# Supplementary material for: The genome of the Hi5 germ cell line from Trichoplusia ni, an agricultural pest and novel model for small RNA biology
Source: eLife. 2018 Jan 29;7:e31628. doi: 10.7554/eLife.31628 (PMC5844692; doi:10.7554/eLife.31628)
Supplement: Supplementary file 9. [file elife-31628-supp9.pdf]

Supplementary File 9. Primers and sequences.

Primer	Sequence	Description
**tracr RNA Core**	GTTTTAGAGCTAGAAATAGCAAGTTAAAATAAGGCTAGTCCGTTATCAACTTGAAAAAGTGGCACCGAGTCGGTGC	Used as template for sgRNA in vitro transcription

ciwi deletion

sgRNA1	CATTTTGTGTTTCTCAACACTGG	PAM
sgRNA2	GGTACGGTGAGAAGCTCTACCGG	PAM
T7-sgRNA1 forward primer	CtaatacgactcactataGGCATTTTGTGTTTCTCAACACgttttagagct	
T7-sgRNA2 forward primer	CtaatacgactcactataGGGGTACGGTGAGAAGCTCTACgttttagagct	
ciwi deletion forward	GCTATTTACCTACACAAACCAATTT	
ciwi Deletion reverse	ACCACGACGTGATCCA	
sgRNA reverse primer	GCACCGACTCGGTGCCACT	

mCherry-*ciwi* knockout

sgRNA5	ATGCTGCAGCTCCGGCGCGTAGG	PAM
sgRNA6	TTTTCAATAACCCAAACATATGG	PAM
T7-sgRNA5 forward primer	CtaatacgactcactataGGATGCTGCAGCTCCGGCGCGTgttttagagct	
T7-sgRNA6 forward primer	CtaatacgactcactataGGTTTTCAATAACCCAAACATAgttttagagct	

vasa HDR

sgRNA3	GCTCAGTAGTAATAGATTTATGG	PAM
sgRNA4	GGATGATGGTGTCGGTGATGTGG	PAM
T7-sgRNA3 forward primer	CtaatacgactcactataGGGCTCAGTAGTAATAGATTTAgttttagagct	
T7-sgRNA4 forward primer	CtaatacgactcactataGGGGATGATGGTGTCGGTGATGgttttagagct	
sgRNA reverse primer	GCACCGACTCGGTGCCACT	

vasa donor amplification

vasa donor forward	/Biotin/CGAATCGAAATCTAAGGCAAG	
vasa donor reverse	ATCTTTGGTGTGAGCTCAAGC	

Primer	Sequence	Description
**Primers to detect *vasa* HDR**		
vasa HR forward	TGACTTGTGAATCCTTGGTTAC	
vasa HR reverse	CATTTTCATAATCCCTTGGTTCTC	
GFP-HA-Vasa N'-Fw	GCGATAAATTGTTGGAAAC	
GFP-HA-Vasa N'-Rv	TCATCCATCCCGCTAC	
GFP-HA-Vasa C'-Fw	GTTTAGAAACATGgtgagcaagg	
GFP-HA-Vasa C'-Rv	CATTTTCATAATCCCTTGGTTCTC	
**Primers for Colony PCR**		
M13 (-20) Fw	GTAAAACGACGGCCAG	
M13 Rv	CAGGAAACAGCTATGAC	

***vasa* genomic sequence**

Exon, **start codon**, sgRNA, PAM

CCGATTCCATTTTCCATTATCTTCTTCCGCCATTGCTAGCGTTATTGGCGGC
TGCTGTCTTTGTTCTATGTGACGCTGTTCACATTTATAAATACTACGCAAATA
CTTCATATAAATTTCAAAGAAGGTAAGATTTCAAAATAGTTATTGTTACTGAA
CAAATATTTACTGGAATGCGCATATTTGCTCTAAACACGTGCAAATAGTTGA
CAAATGCCGGTGCCGGTTGTAGCAAATTTTCAAATTTTTGTGACTGAAAGTT
TTTTTCTGCGATAAATTGTTGGAAACAAATTTATTTGGTCTATATTTTCCTTTT
ATTCATTTGTACTAATATCGAATCGAAATCTAAGGCAAGTTTCAAACCACTTA
GGTGGGTAGGTATTCTGTTTCATGTAGGTAGATAAACTTTTCAATTATTAGCC
TGTACAACTATTGATTGTGTTTATGTTGAAAGATTTATTATGTTTATTATTGAC
TTAGTTGCATTTTCGTAATTCTTGTTAGTTTCTAGTTACCTGCACCATTTTGTT
GATGGCATGGCTGCTTGTAGGTACCTATTACTTTTTGTTACCAAGTGATTTTC
GGTTAAGTTTGGTGGTTTCGATTAAAATAAATTACTTTTTAGGTTTTTTAGTTG
ACTGTAATGTTTTTGCTATTGTGTTATTTCAATTTTGATGGATAGGAAAATGTA
GATAACATACTCATACACAAACAAGATCAAATGCTATGAAATACAAGTTTAAT
AAGTATTGATACTGATTAAAATTGTAGAAGTTGTATAAGTCTCTTAAAAAACT
GTTATGAATTATTCTTAGCTCATAACGTTAAGTTACTATACATGTGAAAAGGA
TCACCTCTGTAGAGCAAAGTGTGACTTGTGAATCCTTGGTTACTTAGATAGA
GTATCTTTCAAATTAATTGTTCTTTAATATGCTATAAAAAAGGAAATATAGGTA
GGAAATTAGGAATGTCAATATTCATGTTAGTCGCCCCAAATTCAGCTAAATTT
TGTAAATAGATTTTATACAACAGTAGCGAACCATTTTGCTGAGCTAAGTCCA
GTAGCCATAAATCTATTACTACTGAGCATTATTATACATTTTATTTTTCTGTTT
AGAAAC**ATG**GATGATGATTGGGATGATGGTGTCGGTGATGTGGTAAGTATT
TAGCGTATAGTCATATTCAAAATTTTAGGGTTTTAAATGGTATATGGCCAATT
GAGCTGACTTGTTGCTATTAACACCTGAGGAATAAATGTATGTCAACAACAA
TGAGAATTATTGTTTATACATTTATGATAATGCTTATTAGTATCCAATGGTGCT
GCTAAACCCAAGATGAGTGACAATTCTGCTCATCATCACATGCTCTTAGTAC
CATAATGAATCAAAAATAATAATAGCCTAGTTTACTTACAGTATCATCTAGAG
AATTAAAATCTAATTGCATTTGTATAATTTGAATAATGTCTTAGTTAAAATGCA
CAAGATTTTTGGGCTCTAGTGATGTACATGATACTTGTTAGCTTTTGACAAAA
GATAGAATAACTTTTATTTTTTAGGTAGTACAATCTCCTGCACTTAACAACTA
CGACTCAAATGTTGATCAAGGGCACAGTCTTTCGAGAGGAAGAGGATTCCA
GTCATTTGATGAAAATGGTAATAATTAATTTGCAAGTCCTGATAATCTGACTT
ATCACTTCTCCCATTTTTTATTGCTGTATTTAGTACTAATAAAATTGCAACATA
ACAAAACTTGAAACTTAGCTAACTCATTTTTCAAGGTATCAGCATTCTTGACT
ACAACACTTCATACACTTATGGCTTTACCACTTAAGTGTTACAAATGAGCCTT
GTTGGTTATACACAGCTTGAGCTCACACCAAAGATATCATGTTACTACTTATA

ATTATAAGTTCTAAGGTCTGCATGTTCATGTTTAGATTATGAAGTGGATAAGG
GCAGCAACGGTTTTGGAGAGAGGCGCGGGCGCGGCGGTAGAGGCGGGCG
GGGAGGAGGCCGTGGGTTCCGTAATGGTGGAACAGGTAGCAGAGAACCAA
GGGATTATGAAAATG

ssDNA donor sequence

Inserted sequence, mutated nucleotide

CGAATCGAAATCTAAGGCAAGTTTCAAACCACTTAGGTGGGTAGGTATTCTG
TTTCATGTAGGTAGATAAACTTTTCAATTATTAGCCTGTACAACTATTGATTGT
GTTTATGTTGAAAGATTTATTATGTTTATTATTGACTTAGTTGCATTTTCGTAA
TTCTTGTTAGTTTCTAGTTACCTGCACCATTTTGTTGATGGCATGGCTGCTTG
TAGGTACCTATTACTTTTTGTTACCAAGTGATTTTCGGTTAAGTTTGGTGGTT
TCGATTAAAATAAATTACTTTTTAGGTTTTTTAGTTGACTGTAATGTTTTTGCT
ATTGTGTTATTTCAATTTTGATGGATAGGAAAATGTAGATAACATACTCATAC
ACAAACAAGATCAAATGCTATGAAATACAAGTTTAATAAGTATTGATACTGAT
TAAAATTGTAGAAGTTGTATAAGTCTCTTAAAAAACTGTTATGAATTATTCTTA
GCTCATAACGTTAAGTTACTATACATGTGAAAAGGATCACCTCTGTAGAGCA
AAGTGTGACTTGTGAATCCTTGGTTACTTAGATAGAGTATCTTTCAAATTAAT
TGTTCTTTAATATGCTATAAAAAAGGAAATATAGGTAGGAAATTAGGAATGTC
AATATTCATGTTAGTCGCCCCAAATTCAGCTAAATTTTGTAAATAGATTTTATA
CAACAGTAGCGAACAATTTTGCTGAGCTAAGTCCAGTAGCAATAAATCTATT
ACTACTGAGCATTATTATACATTTTATTTTTCTGTTTAGAAACatggtgagcaaggg
cgaggagctgttcaccggggtggtgcccatcctggtcgagctggacggcgacgtaaacggccacaagttcag
cgtgtccggcgagggcgagggcgatgccacctacggcaagctgaccctgaagttcatctgcaccaccggcaa
gctgcccgtgccctggcccaccctcgtgaccaccctgacctacggcgtgcagtgcttcagccgctaccccgacc
acatgaagcagcacgacttcttcaagtccgccatgcccgaaggctacgtccaggagcgcaccatcttcttcaag
gacgacggcaactacaagacccgcgccgaggtgaagttcgagggcgacaccctggtgaaccgcatcgagc
tgaagggcatcgacttcaaggaggacggcaacatcctggggcacaagctggagtacaactacaacagccac
aacgtctatatcatggccgacaagcagaagaacggcatcaaggtgaacttcaagatccgccacaacatcgag
gacggcagcgtgcagctcgccgaccactaccagcagaacacccccatcggcgacggccccgtgctgctgcc
cgacaaccactacctgagcacccagtccgccctgagcaaagaccccaacgagaagcgcgatcacatggtcc
tgctggagttcgtgaccgccgccgggatcactctcggcatggacgagctgtacaagggtagtagcggaTACC
CATACGATGTTCCAGATTACGCTggtagtagcggaagtagcgggATGGATGATGATT
GGGATGATGGTGTCGGTGATGTAGTAAGTATTTAGCGTATAGTCATATTCAA
AATTTTAGAGTTTTAAATGGTATATGGCCAATTGAGCTGACTTGTTGCTATTA
ACACCTGAGGAATAAATGTATGTCAACAACAATGAGAATTATTGTTTATACAT
TTATGATAATGCTTATTAGTATCCAATGGTGCTGCTAAACCCAAGATGAGTG
ACAATTCTGCTCATCATCACATGCTCTTAGTACCATAATGAATCAAAAATAAT

AATAGCCTAGTTTACTTACAGTATCATCTAGAGAATTAAAATCTAATTGCATT
TGTATAATTTGAATAATGTCTTAGTTAAAATGCACAAGATTTTTGGGCTCTAG
TGATGTACATGATACTTGTTAGCTTTTGACAAAAGATAGAATAACTTTTATTTT
TTAGGTAGTACAATCTCCTGCACTTAACAACTACGACTCAAATGTTGATCAA
GGGCACAGTCTTTCGAGAGGAAGAGGATTCCAGTCATTTGATGAAAATGGT
AATAATTAATTTGCAAGTCCTGATAATCTGACTTATCACTTCTCCCATTTTTTA
TTGCTGTATTTAGTACTAATAAAATTGCAACATAACAAAACTTGAAACTTAGC
TAACTCATTTTTCAAGGTATCAGCATTCTTGACTACAACACTTCATACACTTA
TGGCTTTACCACTTAAGTGTTACAAATGAGCCTTGTTGGTTATACACAGCTT
GAGCTCACACCAAAGAT

Plasmid template for generating ssDNA donor

Primer binding site, Inserted sequence, Mutated PAM, Plasmid backbone

CGAATCGAAATCTAAGGCAAGTTTCAAACCACTTAGGTGGGTAGGTATTCTG
TTTCATGTAGGTAGATAAACTTTTCAATTATTAGCCTGTACAACTATTGATTGT
GTTTATGTTGAAAGATTTATTATGTTTATTATTGACTTAGTTGCATTTTCGTAA
TTCTTGTTAGTTTCTAGTTACCTGCACCATTTTGTTGATGGCATGGCTGCTTG
TAGGTACCTATTACTTTTTGTTACCAAGTGATTTTCGGTTAAGTTTGGTGGTT
TCGATTAAAATAAATTACTTTTTAGGTTTTTTAGTTGACTGTAATGTTTTTGCT
ATTGTGTTATTTCAATTTTGATGGATAGGAAAATGTAGATAACATACTCATAC
ACAAACAAGATCAAATGCTATGAAATACAAGTTTAATAAGTATTGATACTGAT
TAAAATTGTAGAAGTTGTATAAGTCTCTTAAAAAACTGTTATGAATTATTCTTA
GCTCATAACGTTAAGTTACTATACATGTGAAAAGGATCACCTCTGTAGAGCA
AAGTGTGACTTGTGAATCCTTGGTTACTTAGATAGAGTATCTTTCAAATTAAT
TGTTCTTTAATATGCTATAAAAAAGGAAATATAGGTAGGAAATTAGGAATGTC
AATATTCATGTTAGTCGCCCCAAATTCAGCTAAATTTTGTAAATAGATTTTATA
CAACAGTAGCGAACaATTTTGCTGAGCTAAGTCCAGTAGCaATAAATCTATTA
CTACTGAGCATTATTATACATTTTATTTTTCTGTTTAGAAACATGgtgagcaaggg
cgaggagctgttcaccggggtggtgcccatcctggtcgagctggacggcgacgtaaacggccacaagttcag
cgtgtccggcgagggcgagggcgatgccacctacggcaagctgaccctgaagttcatctgcaccaccggcaa
gctgcccgtgccctggcccaccctcgtgaccaccctgacctacggcgtgcagtgcttcagccgctaccccgacc
acatgaagcagcacgacttcttcaagtccgccatgcccgaaggctacgtccaggagcgcaccatcttcttcaag
gacgacggcaactacaagacccgcgccgaggtgaagttcgagggcgacaccctggtgaaccgcatcgagc
tgaagggcatcgacttcaaggaggacggcaacatcctggggcacaagctggagtacaactacaacagccac
aacgtctatatcatggccgacaagcagaagaacggcatcaaggtgaacttcaagatccgccacaacatcgag
gacggcagcgtgcagctcgccgaccactaccagcagaacacccccatcggcgacggccccgtgctgctgcc
cgacaaccactacctgagcacccagtccgccctgagcaaagaccccaacgagaagcgcgatcacatggtcc
tgctggagttcgtgaccgccgccgggatcactctcggcatggacgagctgtacaagggtagtagcggaTACC

CATACGATGTTCCAGATTACGCTggtagtagcggaagtagcgggATGGATGATGATT
GGGATGATGGTGTCGGTGATGTaGTAAGTATTTAGCGTATAGTCATATTCAA
AATTTTAGaGTTTTAAATGGTATATGGCCAATTGAGCTGACTTGTTGCTATTA
ACACCTGAGGAATAAATGTATGTCAACAACAATGAGAATTATTGTTTATACAT
TTATGATAATGCTTATTAGTATCCAATGGTGCTGCTAAACCCAAGATGAGTG
ACAATTCTGCTCATCATCACATGCTCTTAGTACCATAATGAATCAAAAATAAT
AATAGCCTAGTTTACTTACAGTATCATCTAGAGAATTAAAATCTAATTGCATT
TGTATAATTTGAATAATGTCTTAGTTAAAATGCACAAGATTTTTGGGCTCTAG
TGATGTACATGATACTTGTTAGCTTTTGACAAAAGATAGAATAACTTTTATTTT
TTAGGTAGTACAATCTCCTGCACTTAACAACTACGACTCAAATGTTGATCAA
GGGCACAGTCTTTCGAGAGGAAGAGGATTCCAGTCATTTGATGAAAATGGT
AATAATTAATTTGCAAGTCCTGATAATCTGACTTATCACTTCTCCCATTTTTTA
TTGCTGTATTTAGTACTAATAAAATTGCAACATAACAAAACTTGAAACTTAGC
TAACTCATTTTTCAAGGTATCAGCATTCTTGACTACAACACTTCATACACTTA
TGGCTTTACCACTTAAGTGTTACAAATGAGCCTTGTTGGTTATACACAGCTT
GAGCTCACACCAAAGATATCATGTTACTACTTATAATTATAAGTTCTAATGCA
GATATCCATCACACTGGCGGCCGCTCGAGCATGCATCTAGAGGGCCCAATT
CGCCCTATAGTGAGTCGTATTACAATTCACTGGCCGTCGTTTTACAACGTCG
TGACTGGGAAAACCCTGGCGTTACCCAACTTAATCGCCTTGCAGCACATCC
CCCTTTCGCCAGCTGGCGTAATAGCGAAGAGGCCCGCACCGATCGCCCTT
CCCAACAGTTGCGCAGCCTATACGTACGGCAGTTTAAGGTTTACACCTATAA
AAGAGAGAGCCGTTATCGTCTGTTTGTGGATGTACAGAGTGATATTATTGAC
ACGCCGGGGCGACGGATGGTGATCCCCCTGGCCAGTGCACGTCTGCTGTC
AGATAAAGTCTCCCGTGAACTTTACCCGGTGGTGCATATCGGGGATGAAAG
CTGGCGCATGATGACCACCGATATGGCCAGTGTGCCGGTCTCCGTTATCGG
GGAAGAAGTGGCTGATCTCAGCCACCGCGAAAATGACATCAAAAACGCCAT
TAACCTGATGTTCTGGGGAATATAAATGTCAGGCATGAGATTATCAAAAAGG
ATCTTCACCTAGATCCTTTTCACGTAGAAAGCCAGTCCGCAGAAACGGTGCT
GACCCCGGATGAATGTCAGCTACTGGGCTATCTGGACAAGGGAAAACGCAA
GCGCAAAGAGAAAGCAGGTAGCTTGCAGTGGGCTTACATGGCGATAGCTA
GACTGGGCGGTTTTATGGACAGCAAGCGAACCGGAATTGCCAGCTGGGGC
GCCCTCTGGTAAGGTTGGGAAGCCCTGCAAAGTAAACTGGATGGCTTTCTC
GCCGCCAAGGATCTGATGGCGCAGGGGATCAAGCTCTGATCAAGAGACAG
GATGAGGATCGTTTCGCATGATTGAACAAGATGGATTGCACGCAGGTTCTC
CGGCCGCTTGGGTGGAGAGGCTATTCGGCTATGACTGGGCACAACAGACA
ATCGGCTGCTCTGATGCCGCCGTGTTCCGGCTGTCAGCGCAGGGGCGCCC
GGTTCTTTTTGTCAAGACCGACCTGTCCGGTGCCCTGAATGAACTGCAAGA
CGAGGCAGCGCGGCTATCGTGGCTGGCCACGACGGGCGTTCCTTGCGCAG
CTGTGCTCGACGTTGTCACTGAAGCGGGAAGGGACTGGCTGCTATTGGGC
GAAGTGCCGGGGCAGGATCTCCTGTCATCTCACCTTGCTCCTGCCGAGAAA

GTATCCATCATGGCTGATGCAATGCGGCGGCTGCATACGCTTGATCCGGCT
ACCTGCCCATTCGACCACCAAGCGAAACATCGCATCGAGCGAGCACGTACT
CGGATGGAAGCCGGTCTTGTCGATCAGGATGATCTGGACGAAGAGCATCA
GGGGCTCGCGCCAGCCGAACTGTTCGCCAGGCTCAAGGCGAGCATGCCC
GACGGCGAGGATCTCGTCGTGACCCATGGCGATGCCTGCTTGCCGAATATC
ATGGTGGAAAATGGCCGCTTTTCTGGATTCATCGACTGTGGCCGGCTGGGT
GTGGCGGACCGCTATCAGGACATAGCGTTGGCTACCCGTGATATTGCTGAA
GAGCTTGGCGGCGAATGGGCTGACCGCTTCCTCGTGCTTTACGGTATCGCC
GCTCCCGATTCGCAGCGCATCGCCTTCTATCGCCTTCTTGACGAGTTCTTCT
GAATTATTAACGCTTACAATTTCCTGATGCGGTATTTTCTCCTTACGCATCTG
TGCGGTATTTCACACCGCATACAGGTGGCACTTTTCGGGGAAATGTGCGCG
GAACCCCTATTTGTTTATTTTTCTAAATACATTCAAATATGTATCCGCTCATGA
GACAATAACCCTGATAAATGCTTCAATAATAGCACGTGAGGAGGGCCACCA
TGGCCAAGTTGACCAGTGCCGTTCCGGTGCTCACCGCGCGCGACGTCGCC
GGAGCGGTCGAGTTCTGGACCGACCGGCTCGGGTTCTCCCGGGACTTCGT
GGAGGACGACTTCGCCGGTGTGGTCCGGGACGACGTGACCCTGTTCATCA
GCGCGGTCCAGGACCAGGTGGTGCCGGACAACACCCTGGCCTGGGTGTG
GGTGCGCGGCCTGGACGAGCTGTACGCCGAGTGGTCGGAGGTCGTGTCCA
CGAACTTCCGGGACGCCTCCGGGCCGGCCATGACCGAGATCGGCGAGCA
GCCGTGGGGGCGGGAGTTCGCCCTGCGCGACCCGGCCGGCAACTGCGTG
CACTTCGTGGCCGAGGAGCAGGACTGACACGTGCTAAAACTTCATTTTTAAT
TTAAAAGGATCTAGGTGAAGATCCTTTTTGATAATCTCATGACCAAAATCCCT
TAACGTGAGTTTTCGTTCCACTGAGCGTCAGACCCCGTAGAAAAGATCAAA
GGATCTTCTTGAGATCCTTTTTTTCTGCGCGTAATCTGCTGCTTGCAAACAAA
AAAACCACCGCTACCAGCGGTGGTTTGTTTGCCGGATCAAGAGCTACCAAC
TCTTTTTCCGAAGGTAACTGGCTTCAGCAGAGCGCAGATACCAAATACTGTC
CTTCTAGTGTAGCCGTAGTTAGGCCACCACTTCAAGAACTCTGTAGCACCG
CCTACATACCTCGCTCTGCTAATCCTGTTACCAGTGGCTGCTGCCAGTGGC
GATAAGTCGTGTCTTACCGGGTTGGACTCAAGACGATAGTTACCGGATAAG
GCGCAGCGGTCGGGCTGAACGGGGGGTTCGTGCACACAGCCCAGCTTGG
AGCGAACGACCTACACCGAACTGAGATACCTACAGCGTGAGCTATGAGAAA
GCGCCACGCTTCCCGAAGGGAGAAAGGCGGACAGGTATCCGGTAAGCGGC
AGGGTCGGAACAGGAGAGCGCACGAGGGAGCTTCCAGGGGGAAACGCCT
GGTATCTTTATAGTCCTGTCGGGTTTCGCCACCTCTGACTTGAGCGTCGATT
TTTGTGATGCTCGTCAGGGGGGCGGAGCCTATGGAAAAACGCCAGCAACG
CGGCCTTTTTACGGTTCCTGGGCTTTTGCTGGCCTTTTGCTCACATGTTCTT
TCCTGCGTTATCCCCTGATTCTGTGGATAACCGTATTACCGCCTTTGAGTGA
GCTGATACCGCTCGCCGCAGCCGAACGACCGAGCGCAGCGAGTCAGTGAG
CGAGGAAGCGGAAGAGCGCCCAATACGCAAACCGCCTCTCCCCGCGCGTT
GGCCGATTCATTAATGCAGCTGGCACGACAGGTTTCCCGACTGGAAAGCGG

GCAGTGAGCGCAACGCAATTAATGTGAGTTAGCTCACTCATTAGGCACCCC
AGGCTTTACACTTTATGCTTCCGGCTCGTATGTTGTGTGGAATTGTGAGCGG
ATAACAATTTCACACAGGAAACAGCTATGACCATGATTACGCCAAGCTATTT
AGGTGACACTATAGAATACTCAAGCTATGCATCAAGCTTGGTACCGAGCTC
GGATCCACTAGTAACGGCCGCCAGTGTGCTGGAATTCGCC

Hi5 *ciwi* genomic sequence

Exon, sgRNA for *ciwi* deletion, sgRNA for mCheery-*ciwi* knockout PAM

GCTATTTACCTACACAAACCAATTTAAATATATGAACCGAACACTCATTCGAT
GTTGCCAAGTTAAAATCAAAAATCAATGGGCATTAACAACCAGAAAGTTTATT
GCGTAAAATGATTATTTTTCATTACAATTTATTTCTGATCGAACACAAAAGAT
CAGGGAGATTATTCAAGTAGTTAAAAAGGTAAATAGCCTAATTAGTAATAAAT
GGCATACGCGTTCCAGAAAAAGTGAATATTTTCACATTTCGATTTATAACATT
GCAACATTATTCTGTTATTTTTTTATCGATATTTACTCACAACAAAATCAACAA
ATTATCGACATGTAACATTCGGCTATAGCAATAGGGTAACACAGCTCTAGGA
CTTCTCTTTTTTCTGTTTGCGCGTGCGTACAACGCACGTAGATTTCGCTGTTT
GACTTGTGATAATAATTAAGTTTTGTTTTGCTTATTCTAAGCCGGTAACCTTA
CAATTTCCTAGATTAAGTAACTAAGGTAAGTTACTTTTCGTTGTTATTATAATA
ACTCAGATTGGTATTCTCAGGTCCTCACAGGGTCGCCATTTTTCATTCTTAAT
ATTTTGGCGTATAAACTGACCCACTGAAGAAAAAAACAGATACCTACTTGAA
TATGATGATAACATATTAATTATAACGGTGAGCTTGATACAGCATCTTGATTT
CTATATTTGTTTTATTCAGAATTAAGGAAAGAAACAGGTACCTAGGTATCTCA
ATCGTGTTCATTTTTGTATAAGTATAAATATTGTTTTATAGTATATACCTATCT
AAAACTCACATAATATTATCCTTAATTATCTCAAAAGAGATTTGATATTGAAAT
ACGACGTATATAAGATACATTCTTGGTTTACCTATCTTATTGTTTTGCTTCATA
TATAATAAAATTCTACCTCCATTTTTTTTGTTAAGTCTTGTGTTTCGAAGAATT
CCTTCTAGCGGTTGATGTATCAAATTTCGTATAGTGTGCAAATTTTTTAGTAC
CTACGTAGTCTAGTTATTACAATACGCGACTAAATACTCGGTTTTTACATACA
GCACATACTAATAATATGTGAAAGTGATCTAAATTACTCAAAGCTGGATGGC
CTTAAACAAGATTGACCTTGAAAAACCCTCGCGGTTGTCTCAGTCCCGTTTT
CGTGGAATACACCTTTTTTTTTAAATATTCATTCGTATTCCATACAAAGTCTCT
CAATTTCCGTTACGAAATTGAAAACATAATTTAGATAAGAAATGAAGCGTTTC
TTAATGTAATACATACAAGTTGCAACATGTCTATGTTGTGAACAGCCTGTCAT
ATTATACTGATACTCTATCAACTCAAAATTCTTTACTTGTTATTGGGACTGATA
ATGAACAAAAATGTGGTTAGTGTTCTGGAGTTTCATGAGACACAGATACTTA
AATAGAATAAACAAACAAACAGTTTTCCATTGTTTTGTCCAGTGTTGAGAAAC
ACAAAATGGAAATGTAATGACAGTACCTCATTTAATACTGAATCAGGCTCAC
TATAATTACTATAAAAAGGTCCAGTATAATAAAGAAATTGCAGCAAGTTTTGG

AAATATAGCTTTTTGTCAGACAGTAATGCCTTAGTAAATGGGTACCATTTTTG
GGCTAGGAACCCTAAGAAATCAATCATTTTAATGGTACGCTAAACAATGATC
ACTGGACTATTCATATTACATTTACTCTACTAAAGTGCATTTCTAGAAATGTG
AAATAAAGCTTCCTTATTTCATTGCATGAAACACCAGTTTATCTTATTATTGTA
TAGTTGTGTTCATTTGTGTTTTAAAAAATCATCATACTGCGCACACTAGTTGT
TTTTTTACTGTTAAAACCAGACACTAATAAACATTAGTCTGTTCAAAGCCACA
TTTCAATTTTAACATTTCTCTTGTGATCTTAAAATAAATAAATGAACATCTGAT
ATATTTGTTTTGGTTTTTTTTTAGTGAAGTGACACAATAAGATTGTTTATCTTT
TCTAACAGCATGTAAAATTAGTAACAAAAAAGTTATATGAGCATCCATTATGA
ATAGTTAACCTAGTCCATATGTTTGGGTTATTGAAAACATTTTTAAGCTTATTA
AATTTATCATTTAGATGTCCGAACGCGGACGTGGACGAGCCCGAGGGCTAG
CAGGTAGAGGGGGCGATAATGCTGCAGCTCCGGCGCGTAGGCCTGGGGA
ACGACCCCAAGGCCCACCACAACAACAACAAGGTCCTGTGGGGCCCCGTC
CTCAGCCACCTTCGGCATGGGCACCACCCACTGTTGCCCCTCCGGTCAGA
GCTGGGGTGCCAACGCCAACTGTGCAAGCCGGTAGAGCTTCTCACCGTAC
CACGCCTACCACCCATCAAGATCATCCTGGGGATGTTGATGTTCAACAGAG
GATGCAGGCAATGCAAATTGGTTAGTGATCTTAATTGTAACAATGAATTCAAT
TATTTCCGCTTTATTCCGATTTAACTCATGAAACAACCGTTTCGATCAGGACA
AGCCCAAGCCGGTGCTAGTGGCGATCCAGGTTCGTCAGTGATTGGTCGTG
GATCACGTCGTGGT

mCherry-ciwi **knockout plasmid donor sequence**

sequence inserted, mutated PAM, stop codon, plasmid backbone

CTATCAACTCAAAATTCTTTACTTGTTATTGGGACTGATAATGAACAAAAATG
TGGTTAGTGTTCTGGAGTTTCATGAGACACAGATACTTAAATAGAATAAACA
AACAAACAGTTTTCCATTGTTTTGTCCAGTGTTGAGAAACACAAAATGGAAAT
GTAATGACAGTACCTCATTTAATACTGAATCAGGCTCACTATAATTACTATAA
AAAGGTCCAGTATAATAAAGAAATTGCAGCAAGTTTTGGAAATATAGCTTTTT
GTCAGACAGTAATGCCTTAGTAAATGGGTACCATTTTTGGGCTAGGAACCCT
AAGAAATCAATCATTTTAATGGTACGCTAAACAATGATCACTGGACTATTCAT
ATTACATTTACTCTACTAAAGTGCATTTCTAGAAATGTGAAATAAAGCTTCCT
TATTTCATTGCATGAAACACCAGTTTATCTTATTATTGTATAGTTGTGTTCATT
TGTGTTTTAAAAAATCATCATACTGCGCACACTAGTTGTTTTTTTACTGTTAAA
ACCAGACACTAATAAACATTAGTCTGTTCAAAGCCACATTTCAATTTTAACAT
TTCTCTTGTGATCTTAAAATAAATAAATGAACATCTGATATATTTGTTTTGGTT
TTTTTTTAGTGAAGTGACACAATAAGATTGTTTATCTTTTCTAACAGCATGTAA
AATTAGTAACAAAAAAGTTATATGAGCATCCATTATGAATAGTTAACCTAGTC
AATATGTTTGGGTTATTGAAAACATTTTTAAGCTTATTAAATTTATCATTTAGA
TGGTGAGCAAGGGCGAGGAGGATAACTCCGCCATCATCAAGGAGTTCCTG

CGCTTCAAGGTGCACATGGAGGGCTCCGTGAACGGCCACGAGTTCGAGAT
CGAGGGCGAGGGCGAGGGCCGCCCCTACGAGGGCACCCAGACCGCCAAG
CTGAAGGTGACCAAGGGTGGCCCCCTGCCCTTCGCCTGGGACATCCTGTC
CCCTCAGTTCATGTACGGCTCCAAGGCCTACGTGAAGCACCCCGCCGACAT
CCCCGACTACTTGAAGCTGTCCTTCCCCGAGGGCTTCAAGTGGGAGCGCGT
GATGAACTTCGAGGACGGCGGCGTGGTGACCGTGACCCAGGACTCCTCTC
TGCAGGACGGCGAGTTCATCTACAAGGTGAAGCTGCGCGGCACCAACTTC
CCCTCCGACGGCCCCGTAATGCAGAAGAAGACCATGGGCTGGGAGGCCTC
CTCCGAGCGGATGTACCCCGAGGACGGCGCCCTGAAGGGCGAGATCAAGC
AGAGGCTGAAGCTGAAGGACGGCGGCCACTACGACGCTGAGGTCAAGACC
ACCTACAAGGCCAAGAAGCCCGTGCAGCTGCCCGGCGCCTACAACGTCAA
CATCAAGTTGGACATCACCTCCCACAACGAGGACTACACCATCGTGGAACA
GTACGAACGCGCCGAGGGCCGCCACTCCACCGGCGGCATGGACGAGCTG
TACAAGTAGTCTAGAATGAATCGTTTTTAAAATAACAAATCAATTGTTTTATAA
TATTCGTACGATTCTTTGATTATGTAATAAAATGTGATCATTAGGAAGATTAC
GAAAAATATAAAAAATATGAGTTCTGTGTGTATAACAAATGCTGTAAACGCCA
CAATTGTGTTTGTTGCAAATAAACCCATGATTATTTGATTAAAATTGTTGTTTT
CTTTGTTCATAGACAATAGTGTGTTTTGCCTAAACGTGTACTGCATAAACTCC
ATGCGAGTGTATAGCGAGCTAGTGGCTAACGCTTGCCCCACCAAAGTAGAT
TCGTCAAAATCCTCAATTTCATCACCCTCCTCCAAGTTTAACATTTGGCCGTC
GGAATTAACTTCTAAAGATGCCACATAATCTAATAAATGAAATAGAGATTCAA
ACGTGGCGTCATCGTCCGTTTCGACCATTTCCGAAAAGAACTCGGGCATAA
ACTCTATGATTTCTCTGGACGTGGTGTTGTCGAAACTCTCAAAGTACGCAGT
CAGGAACGTGCGCGACATGTCGTCGGGAAACTCGCGCGGAAACATGTTGT
TGTAACCGAACGGGTCCCATAGCGCCAAAACCAAATCTGCCAGCGTCAATA
GAATGAGCACGATGCCGACAATGGAGCTGGCTTGGATAGCGATTCGAGTTA
ACGGCCGGCCTCCGAACGCGGACGTGGACGAGCCCGAGGGCTAGCAGGT
AGAGGGGGCGATAATGCTGCAGCTCCGGCGCGTAAGCCTGGGGAACGACC
CCAAGGCCCACCACAACAACAACAAGGTCCTGTGGGGCCCCGTCCTCAGC
CACCTTCGGCATGGGCACCACCCACTGTTGCCCCTCCGGTCAGAGCTGGG
GTGCCAACGCCAACTGTGCAAGCCGGTAGAGCTTCTCACCGTACCACGCCT
ACCACCCATCAAGATCATCCTGGGGATGTTGATGTTCAACAGAGGATGCAG
GCAATGCAAATTGGTTAGTGATCTTAATTGTAACAATGAATTCAATTATTTCC
GCTTTATTCCGATTTAACTCATGAAACAACCGTTTCGATCAGGACAAGCCCA
AGCCGGTGCTAGTGGCGATCCAGGTTCGTCAGTGATTGGTCGTGGATCACG
TCGTGGTGGCGGCCGAGTGCTGCCTGAACAAATAACCGTTCTTAGGACTCG
TCCTACTACTATCCAGTCTAAGAAAGGTACTGCTTTGTTGTTTATTATCTACA
TTATAAACTATTTACTACACGTAAATAGAACAAATCTGTATATCCATCTAATTT
AGTGTTGGAAGTTTTTATTATAAAATCTTAATTTTCTCATAGGTTCATCGGGT
ACAGTAATTGATTTGAAAGCCAACTACTTCACTGTGCAGACTACACCACAGT

GGTGCTTATACCAGTATCATGTAGATATTACTCCCGAAGAAGATAGCACCGC
GGTTCGCAAAGGCTTGTTGCGTATTCACGCCAATACACTGGGAGGTGCAGA
TATCCATCACACTGGCGGCCGCTCGAGCATGCATCTAGAGGGCCCAATTCG
CCCTATAGTGAGTCGTATTACAATTCACTGGCCGTCGTTTTACAACGTCGTG
ACTGGGAAAACCCTGGCGTTACCCAACTTAATCGCCTTGCAGCACATCCCC
CTTTCGCCAGCTGGCGTAATAGCGAAGAGGCCCGCACCGATCGCCCTTCC
CAACAGTTGCGCAGCCTATACGTACGGCAGTTTAAGGTTTACACCTATAAAA
GAGAGAGCCGTTATCGTCTGTTTGTGGATGTACAGAGTGATATTATTGACAC
GCCGGGGCGACGGATGGTGATCCCCCTGGCCAGTGCACGTCTGCTGTCAG
ATAAAGTCTCCCGTGAACTTTACCCGGTGGTGCATATCGGGGATGAAAGCT
GGCGCATGATGACCACCGATATGGCCAGTGTGCCGGTCTCCGTTATCGGG
GAAGAAGTGGCTGATCTCAGCCACCGCGAAAATGACATCAAAAACGCCATT
AACCTGATGTTCTGGGGAATATAAATGTCAGGCATGAGATTATCAAAAAGGA
TCTTCACCTAGATCCTTTTCACGTAGAAAGCCAGTCCGCAGAAACGGTGCT
GACCCCGGATGAATGTCAGCTACTGGGCTATCTGGACAAGGGAAAACGCAA
GCGCAAAGAGAAAGCAGGTAGCTTGCAGTGGGCTTACATGGCGATAGCTA
GACTGGGCGGTTTTATGGACAGCAAGCGAACCGGAATTGCCAGCTGGGGC
GCCCTCTGGTAAGGTTGGGAAGCCCTGCAAAGTAAACTGGATGGCTTTCTC
GCCGCCAAGGATCTGATGGCGCAGGGGATCAAGCTCTGATCAAGAGACAG
GATGAGGATCGTTTCGCATGATTGAACAAGATGGATTGCACGCAGGTTCTC
CGGCCGCTTGGGTGGAGAGGCTATTCGGCTATGACTGGGCACAACAGACA
ATCGGCTGCTCTGATGCCGCCGTGTTCCGGCTGTCAGCGCAGGGGCGCCC
GGTTCTTTTTGTCAAGACCGACCTGTCCGGTGCCCTGAATGAACTGCAAGA
CGAGGCAGCGCGGCTATCGTGGCTGGCCACGACGGGCGTTCCTTGCGCAG
CTGTGCTCGACGTTGTCACTGAAGCGGGAAGGGACTGGCTGCTATTGGGC
GAAGTGCCGGGGCAGGATCTCCTGTCATCTCACCTTGCTCCTGCCGAGAAA
GTATCCATCATGGCTGATGCAATGCGGCGGCTGCATACGCTTGATCCGGCT
ACCTGCCCATTCGACCACCAAGCGAAACATCGCATCGAGCGAGCACGTACT
CGGATGGAAGCCGGTCTTGTCGATCAGGATGATCTGGACGAAGAGCATCA
GGGGCTCGCGCCAGCCGAACTGTTCGCCAGGCTCAAGGCGAGCATGCCC
GACGGCGAGGATCTCGTCGTGACCCATGGCGATGCCTGCTTGCCGAATATC
ATGGTGGAAAATGGCCGCTTTTCTGGATTCATCGACTGTGGCCGGCTGGGT
GTGGCGGACCGCTATCAGGACATAGCGTTGGCTACCCGTGATATTGCTGAA
GAGCTTGGCGGCGAATGGGCTGACCGCTTCCTCGTGCTTTACGGTATCGCC
GCTCCCGATTCGCAGCGCATCGCCTTCTATCGCCTTCTTGACGAGTTCTTCT
GAATTATTAACGCTTACAATTTCCTGATGCGGTATTTTCTCCTTACGCATCTG
TGCGGTATTTCACACCGCATACAGGTGGCACTTTTCGGGGAAATGTGCGCG
GAACCCCTATTTGTTTATTTTTCTAAATACATTCAAATATGTATCCGCTCATGA
GACAATAACCCTGATAAATGCTTCAATAATAGCACGTGAGGAGGGCCACCA
TGGCCAAGTTGACCAGTGCCGTTCCGGTGCTCACCGCGCGCGACGTCGCC

GGAGCGGTCGAGTTCTGGACCGACCGGCTCGGGTTCTCCCGGGACTTCGT
GGAGGACGACTTCGCCGGTGTGGTCCGGGACGACGTGACCCTGTTCATCA
GCGCGGTCCAGGACCAGGTGGTGCCGGACAACACCCTGGCCTGGGTGTG
GGTGCGCGGCCTGGACGAGCTGTACGCCGAGTGGTCGGAGGTCGTGTCCA
CGAACTTCCGGGACGCCTCCGGGCCGGCCATGACCGAGATCGGCGAGCA
GCCGTGGGGGCGGGAGTTCGCCCTGCGCGACCCGGCCGGCAACTGCGTG
CACTTCGTGGCCGAGGAGCAGGACTGACACGTGCTAAAACTTCATTTTTAAT
TTAAAAGGATCTAGGTGAAGATCCTTTTTGATAATCTCATGACCAAAATCCCT
TAACGTGAGTTTTCGTTCCACTGAGCGTCAGACCCCGTAGAAAAGATCAAA
GGATCTTCTTGAGATCCTTTTTTTCTGCGCGTAATCTGCTGCTTGCAAACAAA
AAAACCACCGCTACCAGCGGTGGTTTGTTTGCCGGATCAAGAGCTACCAAC
TCTTTTTCCGAAGGTAACTGGCTTCAGCAGAGCGCAGATACCAAATACTGTC
CTTCTAGTGTAGCCGTAGTTAGGCCACCACTTCAAGAACTCTGTAGCACCG
CCTACATACCTCGCTCTGCTAATCCTGTTACCAGTGGCTGCTGCCAGTGGC
GATAAGTCGTGTCTTACCGGGTTGGACTCAAGACGATAGTTACCGGATAAG
GCGCAGCGGTCGGGCTGAACGGGGGGTTCGTGCACACAGCCCAGCTTGG
AGCGAACGACCTACACCGAACTGAGATACCTACAGCGTGAGCTATGAGAAA
GCGCCACGCTTCCCGAAGGGAGAAAGGCGGACAGGTATCCGGTAAGCGGC
AGGGTCGGAACAGGAGAGCGCACGAGGGAGCTTCCAGGGGGAAACGCCT
GGTATCTTTATAGTCCTGTCGGGTTTCGCCACCTCTGACTTGAGCGTCGATT
TTTGTGATGCTCGTCAGGGGGGCGGAGCCTATGGAAAAACGCCAGCAACG
CGGCCTTTTTACGGTTCCTGGCTTTTGCTGGCCTTTTGCTCACATGTTCTT
TCCTGCGTTATCCCCTGATTCTGTGGATAACCGTATTACCGCCTTTGAGTGA
GCTGATACCGCTCGCCGCAGCCGAACGACCGAGCGCAGCGAGTCAGTGAG
CGAGGAAGCGGAAGAGCGCCCAATACGCAAACCGCCTCTCCCCGCGCGTT
GGCCGATTCATTAATGCAGCTGGCACGACAGGTTTCCCGACTGGAAAGCGG
GCAGTGAGCGCAACGCAATTAATGTGAGTTAGCTCACTCATTAGGCACCCC
AGGCTTTACACTTTATGCTTCCGGCTCGTATGTTGTGTGGAATTGTGAGCGG
ATAACAATTTCACACAGGAAACAGCTATGACCATGATTACGCCAAGCTATTT
AGGTGACACTATAGAATACTCAAGCTATGCATCAAGCTTGGTACCGAGCTC
GGATCCACTAGTAACGGCCGCCAGTGTGCTGGAATTCGCC
