# Supplementary material for: The genome of the Hi5 germ cell line from Trichoplusia ni, an agricultural pest and novel model for small RNA biology
Source: eLife. 2018 Jan 29;7:e31628. doi: 10.7554/eLife.31628 (PMC5844692; doi:10.7554/eLife.31628)
Supplement: Supplementary file 10. [file elife-31628-supp10.pdf]

Generating single-stranded DNA (ssDNA) donors
using Streptavidin-coupled Dynabeads

The donor for homology-dependent repair is generated by PCR using one standard and one 5′ biotinylated primer (IDT). In our hands, Phusion High-Fidelity DNA Polymerase (NEB) works well. PCR clean-up steps are not required.

Prepare Dynabeads

1. Resuspend the Dynabeads M-280 streptavidin (ThermoFisher) by vortexing for 1 min.
2. Dispense resuspended Dynabeads into a 1.5 ml tube. Use a volume equivalent to the volume of the PCR reaction to be purified.
3. Add an equal volume of 2× washing buffer (10 mM Tris-HCl [pH 7.5], 1 mM EDTA, 2 M NaCl) to the Dynabeads then vortex 5 sec.
4. Place the tube on a magnetic stand for 1 min, then discard the supernatant.
5. Remove the tube from the magnetic stand and resuspend the washed Dynabeads in 2× washing buffer using a volume equivalent to the volume of the PCR reaction.

Immobilize DNA

1. Add the crude PCR reaction to the washed Dynabeads.
2. Incubate for 30 min at room temperature with gentle rotation.
3. Place the tube in the magnetic stand for 3 min, remove the supernatant containing unbound DNA (Sup 1) to a new tube.
4. Wash the Dynabeads twice with 500 μl 1× washing buffer, mix by pipetting up and down, then collect the beads in the magnetic stand for 2 min.
5. Remove the washing buffer from above the beads.

Denature double-stranded DNA

1. Add 200 μl 0.1 M NaOH to beads, incubate for 5 min at room temperature to denature the biotinylated PCR product .
2. Put tube back in magnetic stand for 3 min, then remove the supernatant (Sup 2) to a new tube. Sup 2 should contain the non-biotinylated DNA strand.
3. Add equal volume of 2× PK buffer (200 mM Tris-HCl [pH7.5], 300 mM NaCl, 25 mM EDTA, 2% w/v SDS) to Sup 2 to neutralize.
4. Wash the Dynabeads twice with 500 μl 1× washing buffer.
5. Resuspend the Dynabeads in 200 μl 10 mM EDTA (pH 8.2).

Release the biotinylated DNA strand

1. Incubate resuspended Dynabeads at 65°C for 5 min.
2. After incubation, put tube back in magnetic stand for 3 min. Collect the supernatant (Sup 3) in a new tube.

Analysis and gel purification of ssDNA donor

1. Add 3 volumes ice-cold absolute ethanol (Decon Laboratories) and 1 µl GlycoBlue coprecipitant (Invitrogen) to each supernatant (Sup 1, Sup 2, and Sup3). Incubate on ice for 1 h, then centrifuge at 15,000× *g* at 4°C for 30 min. Wash DNA pellet with 70% (v/v) ethanol, centrifuge again at 15,000× *g* at 4°C for 5 min to collect pellet. Air dry pellet at room temperature for 5 min. Dissolve pellet in 10 µl dH_2O.
2. Add 2 µl 6× Orange G loading buffer (2.5% [w/v] Ficoll-400, 0.15% [w/v] Orange G) to each pellet, then analyze 12 µl of each sample by agarose gel electrophoresis (1% [w/v] UltraPure [Invitrogen] agarose, 1× TAE buffer [40 mM Tris (pH 8.3), 20 mM acetic acid, 1 mM EDTA] plus 1 µg/µl ethidium bromide [OmniPur]) in 1× TAE buffer at 100 V for 40 min.
3. Excise agarose bands containing the full-length Sup 2 and Sup 3 products. ssDNA will migrate faster than double-stranded DNA of the same length: e.g., a 2,332 bp ssDNA donor runs as if it were ~1 kbp. Extract ssDNA from agarose gel slice using QIAquick Gel Extraction kit (QIAGEN), eluting the ssDNA into water. Store ssDNA donors at –20°C.
