# Supplementary material for: The genome of the Hi5 germ cell line from Trichoplusia ni, an agricultural pest and novel model for small RNA biology
Source: eLife. 2018 Jan 29;7:e31628. doi: 10.7554/eLife.31628 (PMC5844692; doi:10.7554/eLife.31628)
Supplement: Supplementary file 4. [file elife-31628-supp4.pdf]

Provisional ID	:	group0_103
Score total	:	0.8
Score for star read(s)	:	-1.3
Score for read counts	:	0
Score for mfe	:	1.1
Score for randfold	:	1.6
Score for cons. seed	:	-0.6
Total read count	:	312
Mature read count	:	265
Loop read count	:	45
Star read count	:	2

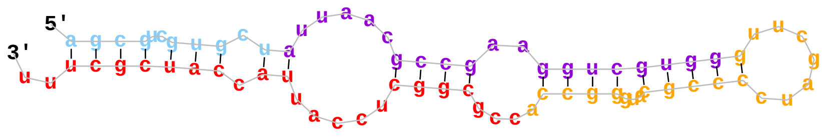

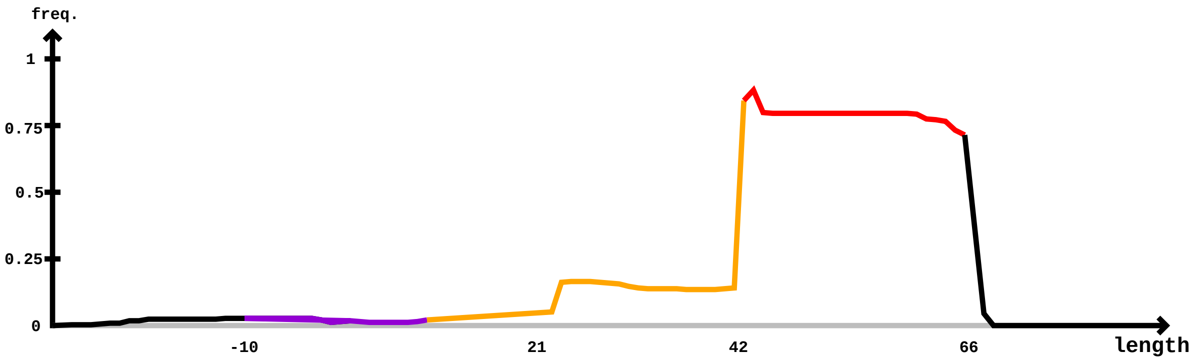

Star Mature

```
5'- cauuagcucaguggcuagagcgucgugcuauuaacgccgaaggucguggguucgauccccgcaugggccaccgcggcuccauuaccaucgcuuuugccauuuuaaucccuuuuu  -3'  obs
    cauuagcucaguggcuagagcgucgugcuauuaacgccgaaggucguggguucgauccccgcaugggccaccgcggcuccauuaccaucgcuuuugccauuuuaaucccuuuuu       exp
    .........((((((.((((((..(((.((.....((((..(((((((((.......)))))...))))....)))).....)).))))))))).)))))).............  reads  mm  sample
    ...................................................ucgauccccUcaugggccacc..........................................  2  1  c15
    ....................................................................caccgcggcuccauuaccaucgcuuuu...................  1  0  c15
    .....................................................................accgcggcuccauuaccaucgcuuuuA..................  1  1  c15
    ......................................................................ccgcggcuccauuaccau..........................  1  0  c15
    ......................................................................ccgcggcuccauuaccauc.........................  3  0  c15
    ......................................................................ccgcggcuccauuaccaucgcu......................  2  0  c15
    ......................................................................ccgcggcuccauuaccaucgcuu.....................  3  0  c15
    ......................................................................ccgcggcuccauuacUaucgcuuu....................  1  1  c15
    ......................................................................cAgcggcuccauuaccaucgcuuu....................  1  1  c15
    ......................................................................ccgcggcuccauuaccaucgcuuu....................  70  0  c15
    ......................................................................ccgcggcuccauuaccaucgcuuA....................  1  1  c15
    ......................................................................ccgcggcuccauuaccaucgcuuuA...................  1  1  c15
    ......................................................................ccgcggcuccauuaccaucgcuuuu...................  43  0  c15
    ......................................................................ccgcggcuccauuaccaucgcuuuuA..................  1  1  c15
    ......................................................................ccgcggcuccauuaccaucgcuuuuU..................  7  1  c15
    .......................................................................cgcggcuccauuaccaucgcuuu....................  11  0  c15
    .......................................................................cgcggcuccauuaccaucgcuuA....................  1  1  c15
    .......................................................................cgcggcuccauuaccaucgcuuuu...................  6  0  c15
    .......................................................................cgcggcuccauuaccaucgcuuuuA..................  1  1  c15
    .......................................................................cgcggcuccauuaccaucgcuuuuU..................  1  1  c15

    .......................................aaggucguggguucgUucc........................................................  1  1  c02
    ........................................aggucguggguucgUucc........................................................  1  1  c02
    ...................................................ucgauccccUcaugggccac...........................................  1  1  c02
    ...................................................ucgauccccUcaugggccacc..........................................  4  1  c02
    ...................................................ucgauccccUcaugggccaccg.........................................  1  1  c02
    ......................................................................ccgcggcuccauuaccaucU........................  1  1  c02
    ......................................................................ccgcggcuccauuaccaucgcuu.....................  2  0  c02
    ......................................................................ccCcggcuccauuaccaucgcuuu....................  1  1  c02
    ......................................................................ccgcggcuccauuaccaucgcuuC....................  1  1  c02
    ......................................................................ccgcggcuccauuaccaucgcuuu....................  4  0  c02
    ......................................................................ccgcggcuccauuaccaucgcuuuu...................  2  0  c02
    .......................................................................cgcggcuccauuaccaucgcu......................  1  0  c02
```

Star **Mature**

cauuagcucaguggcuagagcgucgugcuauuaacgccgaaggucguggguucgauccccgcaugggccaccgcggcuccauuaccaucgcuuuugccauuuuaaucccuuuuu

....ucgauccccUcaugggccacc..	1	1	c04
....ccgcggcuccauuaccauc.........................	1	0	c04
....ccgcggcuccauuaccaucgc.......................	1	0	c04
....ccgcggcuccauuaccaucgcu......................	1	0	c04
....ccgcggcuccauuaccaucgcuuu....................	4	0	c04
....ccgcggcuccauuaccaucgcuuuu...................	1	0	c04
....cgcggcuccauuaccaucgcuuu....................	2	0	c04
cauuagcucaguggcuagagcgucgu..	1	0	c16
...uagcucaguggcuagagcgucgugcua..	1	0	c16
....agcucaguggcuagagcgucgugcuau...	1	0	c16
......cucaguggcuagagcgucgu..	1	0	c16
......cucaguggcuagagcUucgug...	2	1	c16
........caguggcuagagcgucgug...	1	0	c16
........caguggcuagagcgucgugUu...	1	1	c16
................agagcUucgugcuauuaacgccgaaggucguggg..	1	1	c16
............................Gauuaacgccgaaggucgugggu...	1	1	c16
.............................auuaacgccgaaggucgugg...	2	0	c16
....................................ccgaaggucgCggguucg..	1	1	c16
......................................gaaggucguggguucgauccccUcau..	1	1	c16
....aaggucguggguucgaucc..	1	0	c16
....aaggucguggguucgUuccc...	1	1	c16
....gguucgauccccUcaugggccac...	3	1	c16
....ucgauccccUcaugggccacc..	1	1	c16
....ucgauccccgcaugggccaccA...	1	1	c16
....ccgcggcuccauuaccauc.........................	1	0	c16
....ccgcggcuccauuaccaucgc.......................	1	0	c16
....ccgcggcuccauuaccaucgcu......................	3	0	c16
....ccgcggcuccauuaccaucgcuuA....................	1	1	c16
....ccgcggcuccauuaccaucgcuuu....................	25	0	c16
....ccgcggcuccauuaccaucgcuuuu...................	8	0	c16
....ccgcggcuccauuaccaucgcuuuA...................	1	1	c16
....ccgcggcuccauuaccaucgcuuuuA..................	2	1	c16
....ccgcggcuccauuaccaucgcuuuug..................	1	0	c16
....cgcggcuccauuaccaucgcuuu....................	2	0	c16
....cgcggcuccauuaccaucgcuuuu...................	1	0	c16
....cggcuccauuaccaucgcuuu....................	1	0	c16
.....................................cgaaggucguAgguucga...	1	1	c09
.....................................cgaaggucgAggguucgau..	1	1	c09
......................................gaaggucguggguucgUuc...	1	1	c09
....aaggucguggguucgaGcc..	1	1	c09
....aaggucguggguucgUucccc..	1	1	c09
....guucgauccccUcaugggccacc..	1	1	c09
....uucgauccccUcaugggccac...	1	1	c09
....ucgauccccUcaugggccac...	8	1	c09
....ucgauccccUcaugggccacc..	19	1	c09
....cgauccccUcaugggccac...	1	1	c09
....auccccUcaugggccacc..	1	1	c09
....ccgcggcuccauuaccauc.........................	1	0	c09
....ccgcggcuccauuaccaucgcu......................	3	0	c09
....ccgcggcuccauuaccaucgcuu.....................	1	0	c09
....ccgcggcuccauuaccaucgcuuu....................	26	0	c09
....Ucgcggcuccauuaccaucgcuuu....................	1	1	c09
....ccgcggcuccauuaccCucgcuuu....................	1	1	c09
....ccgcggcuccauuaccaucgcuuC....................	1	1	c09
....ccgcggcuccauuaccaucgcuuuA...................	1	1	c09
....ccgcggcuccauuaccaucgcuuuu...................	2	0	c09
....ccgcggcuccauuaccaucgcuuuGg..................	1	1	c09
....cgcggcuccauuaccaucgcu......................	1	0	c09
....Ccggcuccauuaccaucgcuuu....................	1	1	c09

Provisional ID	:	group0_1049
Score total	:	5666.4
Score for star read(s)	:	3.9
Score for read counts	:	5656.1
Score for mfe	:	1.8
Score for randfold	:	1.6
Score for cons. seed	:	3
Total read count	:	11106
Mature read count	:	10516
Loop read count	:	3
Star read count	:	587

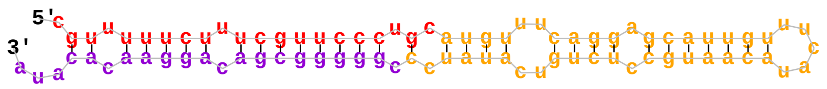

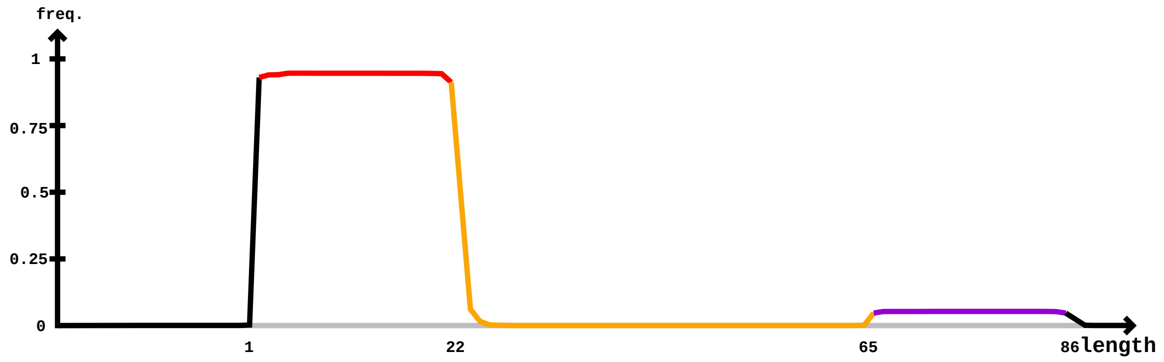


```
                     Mature                                                     Star
5'- aagcauuuggugugguuguacguuuuucuuucguucccugcauguuucaggagcauuguuucauacaaugccucugucauauccccgggggcgacaggaacacauauaacau  -3'   obs
    aagcauuuggugugguuguacguuuuucuuucguucccugcauguuucaggagcauuguuucauacaaugccucugucauauccccgggggcgacaggaacacauauaacau        exp
    ...........((((((((.((.(((((.((((((((.(.((((..((((.(((((((.....))))))).))))..)))).).)))))))).))))).)).))))))))    reads   mm   sample
    ...................cguuuuucuuucguucccugc......................................................................    1       0    c04

    ..................acguuuuucuuucguucccug.......................................................................    1       0    c16
    ...................cguuuuucuuucguucccu........................................................................    8       0    c16
    ...................cguuuuucuuucCuucccug.......................................................................    1       1    c16
    ...................cguuuuucuuucguucccCg.......................................................................    2       1    c16
    ...................cguuuuucuuucguucccug.......................................................................    194     0    c16
    ...................cguuuuucGuucguucccug.......................................................................    1       1    c16
    ...................cCuuuuucuuucguucccug.......................................................................    1       1    c16
    ...................cguuuuucuuucguAcccug.......................................................................    1       1    c16
    ...................cguuCuucuuucguucccug.......................................................................    1       1    c16
    ...................cguuuuucuuucguucAcug.......................................................................    1       1    c16
    ...................cguuuuucuuucguuccAug.......................................................................    2       1    c16
    ...................cguuuuucCuucguucccugc......................................................................    1       1    c16
    ...................cCuuuuucuuucguucccugc......................................................................    1       1    c16
    ...................cguuuuucuuucgAucccugc......................................................................    1       1    c16
    ...................cguuuuucuuucguCcccugc......................................................................    1       1    c16
    ...................cguuuuucuuucguucccugc......................................................................    341     0    c16
    ...................cguAuuucuuucguucccugc......................................................................    1       1    c16
    ...................cguuuuucuuuAguucccugc......................................................................    1       1    c16
    ...................cguuuuucuuucguuccGugc......................................................................    1       1    c16
    ...................cguuuuucuuucguuAccugc......................................................................    1       1    c16
    ...................cguuuuucuuucguucccugU......................................................................    116     1    c16
    ...................cguuuuucuuucguucccugG......................................................................    1       1    c16
    ...................cguuuuAcuuucguucccugc......................................................................    1       1    c16
    ...................cguuuuucuuucguucccAgca.....................................................................    1       1    c16
    ...................cguuuuucuuucguAcccugca.....................................................................    1       1    c16
    ...................cguuuuucuuuAguucccugca.....................................................................    1       1    c16
    ...................cguuuuucuuucguucccugcC.....................................................................    1       1    c16
    ...................cguuuuucuuucguucUcugca.....................................................................    1       1    c16
    ...................cguuuuucuuucguucccugca.....................................................................    119     0    c16
    ...................cguuuuucuuucguucccugcU.....................................................................    61      1    c16
    ...................cguuuuucuuucguucccugUa.....................................................................    4       1    c16
```

Mature Star

aagcauuugguguguuguacguuuuucuuucguucccugcauguuucaggagcauuguuucauacaaugccucugucauaucccgggggcgacaggaacacauauaacau			
...................cguuuuucuAucguucccugcau..	1	1	c16
...................cguuuuucuuucguucccugcaA..	2	1	c16
...................cguuuuucuuucguucccugcUu..	9	1	c16
...................cguuuuucuuucguucccugcau..	21	0	c16
...................cguuuuucuuucguucccugcaC..	3	1	c16
...................cguuuuucuuucguucccugcauA...	2	1	c16
...................cguuuuucuuucguucccugcauU...	4	1	c16
...................cguuuuucuuucguucccugcauUuuu..	1	1	c16
....................guuuuucuuucguucccugc..	3	0	c16
....................guuuuucuuucguucccugcU...	2	1	c16
....................guuuuucuuucguucccugca...	1	0	c16
.....................uuuuucuuucguucccugc..	2	0	c16
......................Guuucuuucguucccugc..	1	1	c16
......................uuuucuuucguucccugU..	5	1	c16
......................uuuucuuucguucccugc..	24	0	c16
......................uuuucuuucguucccugca...	8	0	c16
......................uuuucuuucguucccugcU...	3	1	c16
......................uuuucuuucguucccugcUu..	1	1	c16
......................uuuucuuucguucccugcau..	2	0	c16
......................uuuucuuucguucccugcauU...	1	1	c16
.......................uuucuuucguucccugcU...	1	1	c16
....cgggggcgacaggaacac.........	1	0	c16
....cgggggcgacaggaacacau.......	1	0	c16
...................cguuuuucuuucguucccug...	1	0	c09
...................cguuuuucuuucguucccugc..	4	0	c09
...................cguuuuucuuucguucccugca...	1	0	c09
.agcauuugguguguuguacgu..	1	0	c02
...................cguuuuucuuucguucccu..	1	0	c02
...................cguuuuucuuucguucccug...	37	0	c02
...................cguuuuucuuucguucccuU...	1	1	c02
...................cguuuuucuuucguucccugc..	74	0	c02
...................cguuuuuAuuucguucccugc..	1	1	c02
...................cguuuAucuuucguucccugc..	1	1	c02
...................cguuuuucuuucguucccugA..	1	1	c02
...................cguuuuucuuucguucccugU..	24	1	c02
...................cguuuuucuuuAguucccugc..	1	1	c02
...................cguuuuucuuucguucccugca...	7	0	c02
...................cguuuuucuuucguucccugcU...	5	1	c02
...................cguuuuucuuucguucccugUau..	1	1	c02
...................cguuuuucuuucguucccugcau..	2	0	c02
.....................uuuuucuuucguucccug...	1	0	c02
.....................Guuuucuuucguucccugc..	1	1	c02
.....................uuuuucuuucguucccugc..	2	0	c02
......................uuuucuuucguucccugc..	8	0	c02
......................uuuucuuucguucccugU..	1	1	c02
......................uuuucuuucguucccugca...	1	0	c02
......................uuuucuuucguucccugcauU...	1	1	c02
aagcauuugguguguuguacU...	1	1	c15
...cauuugguguguuguacguuu..	1	0	c15
.....uuugguguguuguacguuuu...	2	0	c15
......uugguguguuguacguuuuucuuucgu...	1	0	c15
..................acguuuuucuuucguucccug...	1	0	c15
..................Ccguuuuucuuucguucccugc..	6	1	c15
..................acguuuuucuuucguucccugc..	7	0	c15
..................Ccguuuuucuuucguucccugca...	1	1	c15
...................cguuuuucuuucguuccc...	5	0	c15
...................cguuuuucuuuUguuccc...	2	1	c15
...................cguuuuucuuucguucccu..	1	0	c15
...................cguuuuucuuucguucccuA...	10	1	c15
...................cguuuuucuuucguucccuC...	1	1	c15
...................cguuuuucuuucguucccug...	99	0	c15
...................cguuuuucuuucguucccuU...	1	1	c15
...................cguCuuucuuucguucccugc..	1	1	c15
...................cguuuuucuuucguucccuAc..	69	1	c15
...................cguuAuucuuucguucccugc..	1	1	c15
...................cguuuuucuuAcguucccugc..	1	1	c15
...................cguuuuucuuucguuGccugc..	1	1	c15

Mature Star

aagcauuugguguguuguacguuuuucuuucguucccugcauguuucaggagcauuguuucauacaaugccucugucauaucccgggggcgacaggaacacauauaacau

...................cguuuuucuuucgAucccugc..	2	1	c15
...................cUuuuuucuuucguucccugc..	1	1	c15
...................cguuuuucuuucguucccugG..	5	1	c15
...................cguuuuucuuucguuccAugc..	1	1	c15
...................cguuuuucuuucguAcccugc..	2	1	c15
...................cguuuuucuuucguuccUugc..	2	1	c15
...................cguuuuucuuucguucccAgc..	1	1	c15
...................cguuuuucuuuUguucccugc..	3	1	c15
...................cguuuuucuuucguuUccugc..	1	1	c15
...................cguuuuucuuucguucccuUc..	4	1	c15
...................cgGuuuucuuucguucccugc..	1	1	c15
...................cguuuuucuuucgGucccugc..	1	1	c15
...................cguuCuucuuucguucccugc..	2	1	c15
...................Aguuuuucuuucguucccugc..	1	1	c15
...................cguuuuuGuuucguucccugc..	1	1	c15
...................cguuuuucuuucgCucccugc..	3	1	c15
...................cguuuuucuuucguucAcugc..	3	1	c15
...................cguuuuucuuCcguucccugc..	2	1	c15
...................cgCuuuucuuucguucccugc..	4	1	c15
...................cguuuuucuAucguucccugc..	1	1	c15
...................cguuuuucGuucguucccugc..	2	1	c15
...................cAuuuuucuuucguucccugc..	3	1	c15
...................Uguuuuucuuucguucccugc..	4	1	c15
...................cguuuGucuuucguucccugc..	5	1	c15
...................cguuuCucuuucguucccugc..	3	1	c15
...................cguuuuCcuuucguucccugc..	1	1	c15
...................cguuuAucuuucguucccugc..	1	1	c15
...................Gguuuuucuuucguucccugc..	1	1	c15
...................cguuuuucuuucguucccugc..	6304	0	c15
...................cguuuuucuuucguucccGgc..	4	1	c15
...................cguuuuuAuuucguucccugc..	1	1	c15
...................cguuuuucuuucguucccugA..	71	1	c15
...................cguuuuuUuuucguucccugc..	3	1	c15
...................cguuuuucuuucguucccugU..	282	1	c15
...................cguuuuucuuucUuucccugc..	2	1	c15
...................cgAuuuucuuucguucccugc..	1	1	c15
...................cguuuuucuuuGguucccugc..	1	1	c15
...................cguuuuucAuucguucccugc..	2	1	c15
...................cguuuuucuuucAuucccugc..	4	1	c15
...................cguuuuucuuucguucccCgc..	4	1	c15
...................cguuuuucuCucguucccugc..	1	1	c15
...................cguuuuucuuucguucUcugc..	3	1	c15
...................cgAuuuucuuucguucccugca...	1	1	c15
...................cguuuuucuuucAuucccugca...	1	1	c15
...................cguuuuucuuucguucccAgca...	1	1	c15
...................cguuuuucuuucguucccugcC...	13	1	c15
...................cguuuAucuuucguucccugca...	1	1	c15
...................cguuuuucuuuUguucccugca...	1	1	c15
...................cguuuuucuuucguucAcugca...	1	1	c15
...................cguuuuucuuucguucUcugca...	1	1	c15
...................cguuuuucuuucguucccuCca...	1	1	c15
...................cguuuuucuuucguucccugUa...	28	1	c15
...................Aguuuuucuuucguucccugca...	1	1	c15
...................cguuuuucuuucguucccugAa...	3	1	c15
...................cguuuuucuuucguucccuAca...	2	1	c15
...................cguuuuucuuucguucccugca...	661	0	c15
...................cguuuuucuuucguucccugcG...	6	1	c15
...................Uguuuuucuuucguucccugca...	1	1	c15
...................cguuuuucuuucguucccugcU...	974	1	c15
...................cguuuuucuuucguucccugcaA..	40	1	c15
...................cguuuuucuuucguucccugcau..	192	0	c15
...................cguuuuucuuucguucccugcaC..	5	1	c15
...................cguuuuucuuucguucccugUau..	8	1	c15
...................cguuuuucuuucguucccugcUu..	211	1	c15
...................cguuuuucuuucguucccugcGu..	1	1	c15
...................cguuuuucuuucguucccugcauC...	2	1	c15
...................cguuuuucuuucguucccugcauA...	9	1	c15
...................cguuuuucuuucguucccugcauU...	119	1	c15
...................cguuuuucuuucguucccugcauCu..	1	1	c15
...................cguuuuucuuucguucccugcauAu..	4	1	c15

Mature **Star**

```
aagcauuugguguguuguacguuuuucuuucguucccugcauguuucaggagcauuguuucauacaaugccucugucauaucccgggggcgacaggaacacauauaacau
...................cguuuuucuuucguucccugcauUu..................................................................  12   1  c15
...................cguuuuucuuucguucccugcauAuu.................................................................  1    1  c15
...................cguuuuucuuucguucccugcauUuu.................................................................  8    1  c15
...................cguuuuucuuucguucccugcauUuuu................................................................  2    1  c15
...................cguuuuucuuucguucccugcauAuuu................................................................  1    1  c15
....................guuuuucuuucguucccugA......................................................................  1    1  c15
....................guuuuucuuucguucccugc......................................................................  59   0  c15
....................guuuuucuuucguucccugU......................................................................  9    1  c15
....................guuuuucuuucguucccugcU.....................................................................  13   1  c15
....................guuuuucuuucguucccugca.....................................................................  10   0  c15
....................guuuuucuuucguucccugcUu....................................................................  1    1  c15
....................guuuuucuuucguucccugcau....................................................................  5    0  c15
....................guuuuucuuucguucccugcauU...................................................................  4    1  c15
.....................Guuuucuuucguucccugc......................................................................  1    1  c15
.....................uuuuucuuucguucccugcU.....................................................................  1    1  c15
.....................uuuuucuuucguucccugcUu....................................................................  1    1  c15
......................uuuucuuucguucccugc......................................................................  3    0  c15
......................uuuucuuucguucccugcU.....................................................................  1    1  c15
......................uuuucuuucguucccugcau....................................................................  1    0  c15
......................uuuucuuucguucccugcauA...................................................................  1    1  c15
.......................uuucuuucguucccugcUu....................................................................  1    1  c15
........................................................guuucauacaaugccucuguc.................................  1    0  c15
........................................................guuucauacaaugccucugucauaucc...........................  1    0  c15
.........................................................uuucauacUaugccucugucauaucc...........................  1    1  c15
.................................................................................cccgggggcgacaggaacac.........  1    0  c15
.................................................................................cccgggggcgacaggaacaca........  1    0  c15
.................................................................................cccgggggcgacaggaacacaua......  1    0  c15
..................................................................................ccgggggcgacaggaacacaua......  4    0  c15
..................................................................................cGgggggcgacaggaacacaua......  1    1  c15
...................................................................................cgggggcgacaggaacac.........  1    0  c15
...................................................................................cgggggcgacaggaacaca........  5    0  c15
...................................................................................cgggggcgacaggaacacUu.......  1    1  c15
...................................................................................cgggggcgacaggaacacGu.......  2    1  c15
...................................................................................cgggggcgacaggaacacau.......  53   0  c15
...................................................................................cgggggcgacagUaacacau.......  1    1  c15
...................................................................................cgggggcgacaggaacacaA.......  1    1  c15
...................................................................................Ugggggcgacaggaacacaua......  1    1  c15
...................................................................................cgggggcgacaggaacacauU......  22   1  c15
...................................................................................cgggggcgacaggaacacaua......  343  0  c15
...................................................................................cgggggcgacaggaacacaCa......  1    1  c15
...................................................................................cggggUcgacaggaacacaua......  1    1  c15
...................................................................................cgggggcgacaggaacacGua......  9    1  c15
...................................................................................cggggAcgacaggaacacaua......  1    1  c15
...................................................................................cgggggcgacaggaacacauaA.....  14   1  c15
...................................................................................cgggggcgacaggaacacGuau.....  5    1  c15
...................................................................................Ugggggcgacaggaacacauau.....  1    1  c15
...................................................................................cUggggcgacaggaacacauau.....  1    1  c15
...................................................................................cgggggcgacaggaacacauau.....  38   0  c15
...................................................................................cgggggcgacaggaacacauUu.....  5    1  c15
...................................................................................cgggggcgacaggaacacauauU....  4    1  c15
...................................................................................cgggggcgacaggaacacauaAa....  1    1  c15
...................................................................................cgggggcgacaggaacacauaua....  1    0  c15
...................................................................................cgggggcgacaggaacacauaAaa...  1    1  c15
....................................................................................gggggcgacaggaacacau.......  1    0  c15
....................................................................................Uggggcgacaggaacacaua......  1    1  c15
....................................................................................gggggcgacaggaacacaua......  57   0  c15
....................................................................................gggggcgacaggaacacGuau.....  1    1  c15
....................................................................................gggggcgacaggaacacauau.....  2    0  c15
....................................................................................gggggcgacaggaacacauauU....  1    1  c15
.....................................................................................ggggcgacaggaacacauaA.....  1    1  c15
.........................................................................................cgacaggaacacauauaacau  4    0  c15
```

Provisional ID	:	group0_1167
Score total	:	169.2
Score for star read(s)	:	3.9
Score for read counts	:	159.2
Score for mfe	:	1.5
Score for randfold	:	1.6
Score for cons. seed	:	3
Total read count	:	324
Mature read count	:	304
Loop read count	:	0
Star read count	:	20

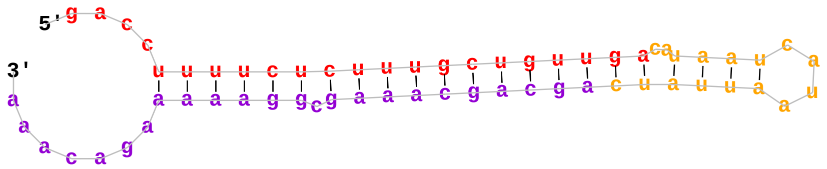

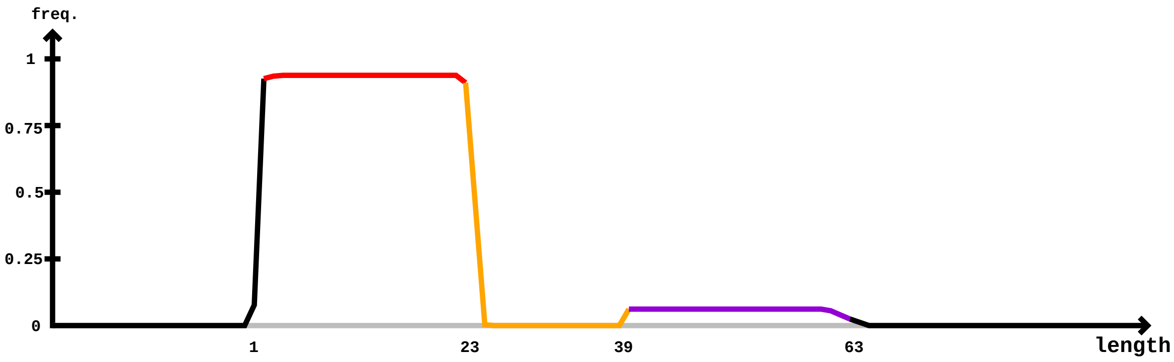

Mature **Star**

```
5'- gaggaaguucucauaaaggagaccuuuucucuuugcuguugacauaaucauaauuaucagcagcaaagcggaaaaagacaaauuuauggagaacuuucgcuucuaucuucug  -3'   obs
    gaggaaguucucauaaaggagaccuuuucucuuugcuguugacauaaucauaauuaucagcagcaaagcggaaaaagacaaauuuauggagaacuuucgcuucuaucuucug         exp
    ..(((((((((((((((.......((((((((((((((((((..((((....)))))))))))))))).)))))).......))))).))))))))))..............   reads   mm   sample
    .....................accuuuucucuuugcuguugac.....................................................................   1       0    c04

    ....................gaccuuuucucuuugcuguuga......................................................................   1       0    c09

    ..................agaccuuuucucuuugcuguuga.......................................................................   25      0    c15
    ....................gaccuuuucucuuugcuguug.......................................................................   9       0    c15
    ....................gaccuuuucucuuugcuAuuga......................................................................   1       1    c15
    ....................gaccGuuucucuuugcuguuga......................................................................   1       1    c15
    ....................gGccuuuucucuuugcuguuga......................................................................   2       1    c15
    ....................gaccuuuucucuuugcuguuAa......................................................................   1       1    c15
    ....................gaccuuuucucuuugcuguuga......................................................................   240     0    c15
    ....................gaccuuuucucuuugcuguugU......................................................................   2       1    c15
    ....................Uaccuuuucucuuugcuguuga......................................................................   2       1    c15
    ....................gaccuuuucucuuugcuguugac.....................................................................   11      0    c15
    ....................gaccuuuucucuuugcuguugaA.....................................................................   2       1    c15
    ....................gaccuuuucucuuugcuguugacC....................................................................   1       1    c15
    .....................accuuuucucuuugcuguuga......................................................................   2       0    c15
    ......................ccuuuucucuuugcuguugac.....................................................................   1       0    c15
    ..........................................................agcagcaaagcggaaaaagac.................................   2       0    c15
    ..........................................................agcagcaaagcggaaaaagaca................................   5       0    c15
    ..........................................................agcagcaaagcggaaaaagacaa...............................   4       0    c15
    ..........................................................Ggcagcaaagcggaaaaagacaa...............................   1       1    c15
    ..........................................................agcagcaaagcggaaaaGgacaaa..............................   1       1    c15
    ..........................................................agcagcaaagcggaaaaagacaaa..............................   7       0    c15

    ....................gaccuuuucucuuugcuguugac.....................................................................   2       0    c02
```

Provisional ID : group0_1265
Score total : 28198.3
Score for star read(s) : 3.9
Score for read counts : 28187.8
Score for mfe : 1.9
Score for randfold : 1.6
Score for cons. seed : 3
Total read count : 55301
Mature read count : 55260
Loop read count : 0
Star read count : 41

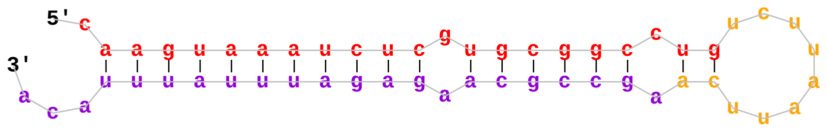

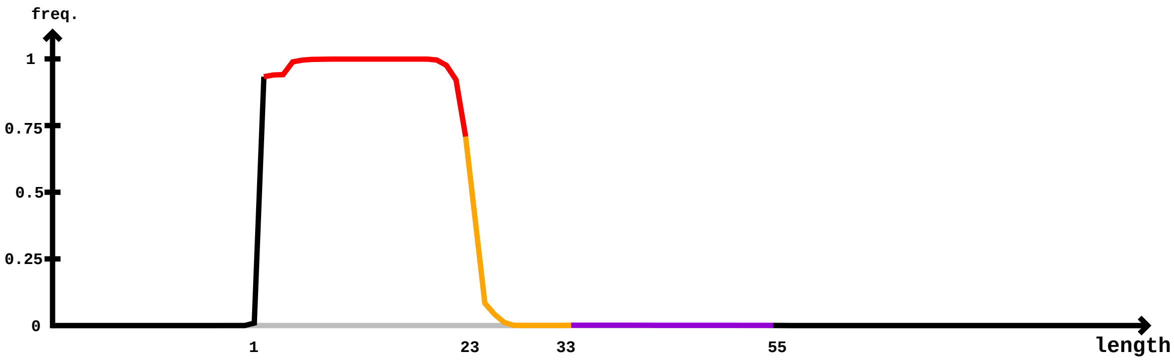

Mature Star

```
5'- aagucgauuuaccgucgacacaaguaaaucucgugcggccugucuuaaauucaagccgcaagagauuuauuuacaucgaugguaaaagaacaucuguuauuaccggcuacgua   -3'   obs
    aagucgauuuaccgucgacacaaguaaaucucgugcggccugucuuaaauucaagccgcaagagauuuauuuacaucgaugguaaaagaacaucuguuauuaccggcuacgua         exp
    .(((((.(((((((((((...(((((((((((.((((((.((.........)).)))))).)))))))))))...)))))))))))..(((....))).....))))).....   reads   mm   sample
    ...................acaaguaaaucucgugcggc..........................................................................   1       0    c15
    ...................acaaguaaaucucgugcggccA........................................................................   1       1    c15
    ...................acaaguaaaucucgugcggccugA......................................................................   1       1    c15
    ...................acaaguaaaucucgugcggccuAu......................................................................   1       1    c15
    ...................acaaguaaaucucgugcggccuguU.....................................................................   1       1    c15
    ...................acaaguaaaucucgugcggccuguUu....................................................................   1       1    c15
    ....................caaguaaaucucgugcggcc.........................................................................   2       0    c15
    ....................caaguaaaucucgugcggccu........................................................................   9       0    c15
    ....................caaguaaaucucgugcggccuU.......................................................................   3       1    c15
    ....................caaguaaaucucgugcggccug.......................................................................   4       0    c15
    ....................caaguaaaucucgugcggccugA......................................................................   2       1    c15
    ....................caaguaaaucucgugcggccugC......................................................................   1       1    c15
    ....................caaguaaaucucgugcggccuUu......................................................................   1       1    c15
    ....................caaguaaaucucgugcggccugu......................................................................   1       0    c15
    ....................caaguaaaucucgugcggccuguc.....................................................................   1       0    c15
    ....................caaguaaaucucgugcggccuguU.....................................................................   6       1    c15
    ....................caaguaaaucucgugcggccuguUu....................................................................   6       1    c15
    ....................caaguaaaucucgugcggccugucu....................................................................   1       0    c15
    ....................caaguaaaucucgugcggccuguAuu...................................................................   1       1    c15
    ....................caaguaaaucucgugcggccuguUuu...................................................................   1       1    c15
    .....................................................agccgcaagagauuuauuua........................................   1       0    c15
    .....................................................agccgcaagagauuuauuuac.......................................   2       0    c15
    .....................................................agccgcaagagauuuauuuaca......................................   5       0    c15

    .................aGacaaguaaaucucgugcggccug.......................................................................   1       1    c02
    ...................acaaguaaaucucgugcgg...........................................................................   7       0    c02
    ...................acaaguaaaucucguUcgg...........................................................................   1       1    c02
    ...................Ccaaguaaaucucgugcgg...........................................................................   3       1    c02
    ...................Ccaaguaaaucucgugcggc..........................................................................   1       1    c02
    ...................acaaguaaaucucgugcggc..........................................................................   12      0    c02
    ...................acaaguaaaucucgAgcggc..........................................................................   1       1    c02
    ...................Ccaaguaaaucucgugcggcc.........................................................................   9       1    c02
    ...................acaaguaaaucucgugcggcc.........................................................................   30      0    c02
```

```
                    Mature                Star
aagucgauuuaccgucgacacaaguaaaucucgugcggccugucuuaauucaagccgcaagagauuuauuuacaucgaugguaaaagaacaucuguuauuaccggcuacgua
...................acaaguaaaucucUugcggcc........................................................................    1     1     c02
...................acaagAaaaucucgugcggccu.......................................................................    1     1     c02
...................acaaguaaaucucgugcggccu.......................................................................    48    0     c02
...................Ccaaguaaaucucgugcggccu.......................................................................    6     1     c02
...................acaaguaaaucucgugcggcAu.......................................................................    1     1     c02
...................acaaguaaaucucgugcggccuU......................................................................    2     1     c02
...................acaaguaaaucucgugcggccug......................................................................    11    0     c02
...................Ccaaguaaaucucgugcggccug......................................................................    3     1     c02
...................acaaguaaaucucgugcggccugu.....................................................................    8     0     c02
...................acaaguaaaucucgugcggccugG.....................................................................    1     1     c02
...................acaaguaaaucucgugcggccuUu.....................................................................    3     1     c02
...................Ccaaguaaaucucgugcggccugu.....................................................................    1     1     c02
...................acaaguaaaucucgugcCgccugu.....................................................................    1     1     c02
....................caaguaaaucucAugcgg..........................................................................    1     1     c02
....................caaguaaaucucgugcgg..........................................................................    75    0     c02
....................caaCuaaaucucgugcgg..........................................................................    1     1     c02
....................caaguaaaucucguUcgg..........................................................................    1     1     c02
....................caaguaaaucuAgugcgg..........................................................................    1     1     c02
....................caaguaaaucuAgugcggc.........................................................................    2     1     c02
....................caaguaaaucucgugcUgc.........................................................................    4     1     c02
....................caaguaaCucucgugcggc.........................................................................    2     1     c02
....................caagAaaaucucgugcggc.........................................................................    1     1     c02
....................caaguaaaucucgugcggc.........................................................................    519   0     c02
....................caaguaaaucucgAgcggc.........................................................................    2     1     c02
....................caaguaaaAcucgugcggc.........................................................................    1     1     c02
....................caaguaaaucucgugcggA.........................................................................    2     1     c02
....................caaguaaaucucgugcAgc.........................................................................    1     1     c02
....................caaguaaaucucgugAggc.........................................................................    3     1     c02
....................caaguaaaucucgugcggU.........................................................................    4     1     c02
....................caGguaaaucucgugcggc.........................................................................    1     1     c02
....................caaguaaaucCcgugcggcc........................................................................    1     1     c02
....................caaguaaaucucCugcggcc........................................................................    2     1     c02
....................caaguaaaucucgugAggcc........................................................................    6     1     c02
....................caaguGaaucucgugcggcc........................................................................    1     1     c02
....................caaguaaaucucgugcUgcc........................................................................    2     1     c02
....................caaguaaaucucgugcCgcc........................................................................    2     1     c02
....................caaguaaaucucgCgcggcc........................................................................    1     1     c02
....................cUaguaaaucucgugcggcc........................................................................    1     1     c02
....................caaguaaaucucguUcggcc........................................................................    5     1     c02
....................caaguaaaucucgugcggcc........................................................................    1346  0     c02
....................caaguaaaucucgugcggGc........................................................................    1     1     c02
....................caaguaaaucucgugcggcU........................................................................    1     1     c02
....................Uaaguaaaucucgugcggcc........................................................................    1     1     c02
....................caaguaaaucucgugcggUc........................................................................    1     1     c02
....................caaguaaaucucguCcggcc........................................................................    2     1     c02
....................caaguaaaucucgugcgUcc........................................................................    2     1     c02
....................caaguaaaucucgAgcggcc........................................................................    1     1     c02
....................caaguaaaucuAgugcggcc........................................................................    7     1     c02
....................caaguaaaucucgugcgCcc........................................................................    1     1     c02
....................caaguaaCucucgugcggcc........................................................................    2     1     c02
....................caaguaaaucucgugcggAc........................................................................    5     1     c02
....................caaguaaaCcucgugcggcc........................................................................    1     1     c02
....................caaguaaaucucAugcggcc........................................................................    4     1     c02
....................caaguaaaGcucgugcggcc........................................................................    1     1     c02
....................caagAaaaucucgugcggcc........................................................................    1     1     c02
....................caaguaaaucucgugcggcA........................................................................    1     1     c02
....................caaguaUaucucgugcggcc........................................................................    1     1     c02
....................caaguaCaucucgugcggccu.......................................................................    1     1     c02
....................caaguaaaucucgAgcggccu.......................................................................    2     1     c02
....................caaguaaauGucgugcggccu.......................................................................    2     1     c02
....................caaguaaaucGcgugcggccu.......................................................................    1     1     c02
....................caaguaaaucucgugAggccu.......................................................................    13    1     c02
....................caagAaaaucucgugcggccu.......................................................................    5     1     c02
....................caaguaaauUucgugcggccu.......................................................................    2     1     c02
....................caaguaaauAucgugcggccu.......................................................................    6     1     c02
....................caaguaaaucucgCgcggccu.......................................................................    3     1     c02
....................caaguaaaucucgugGggccu.......................................................................    1     1     c02
....................caaguaaaucucgugcggUcu.......................................................................    4     1     c02
....................caaguaaaucucgugcggcGu.......................................................................    1     1     c02
....................caaguaaCucucgugcggccu.......................................................................    18    1     c02
```

```
                    Mature                        Star
aagucgauuuaccgucgacacaaguaaaucucgugcggccugucuuaauucaagccgcaagagauuuauuuacaucgaugguaaaagaacaucuguuauuaccggcuacgua
....................caaguGaaucucgugcggccu....................................................................... 1 1 c02
....................caaguaaaucucgugcggcUu....................................................................... 1 1 c02
....................caaguaaaucucgugcgUccu....................................................................... 1 1 c02
....................caaguaaaucucgugUggccu....................................................................... 3 1 c02
....................caaCuaaaucucgugcggccu....................................................................... 6 1 c02
....................caGguaaaucucgugcggccu....................................................................... 2 1 c02
....................caaguaaaucucCugcggccu....................................................................... 8 1 c02
....................caaguaaaucucgugcgCccu....................................................................... 6 1 c02
....................caaguaaaucucgugcggccu....................................................................... 3849 0 c02
....................caaguaaaucucgugcggccA....................................................................... 7 1 c02
....................caaguaaaAcucgugcggccu....................................................................... 2 1 c02
....................cUaguaaaucucgugcggccu....................................................................... 2 1 c02
....................caaguaaaucucgugcggAcu....................................................................... 12 1 c02
....................caaguaaaucucgugcggcAu....................................................................... 2 1 c02
....................caaguaaaucucguCcggccu....................................................................... 7 1 c02
....................caagGaaaucucgugcggccu....................................................................... 1 1 c02
....................caaguaaaucucAugcggccu....................................................................... 1 1 c02
....................caaguaaaucucgugcgAccu....................................................................... 1 1 c02
....................caaguaaaucucgugcUgccu....................................................................... 7 1 c02
....................caaguaaaucucUugcggccu....................................................................... 1 1 c02
....................caaguaaaucucgugcCgccu....................................................................... 1 1 c02
....................Aaaguaaaucucgugcggccu....................................................................... 7 1 c02
....................caaguaaaucuAgugcggccu....................................................................... 6 1 c02
....................caaguaaaucucgugcggccC....................................................................... 6 1 c02
....................caUguaaaucucgugcggccu....................................................................... 1 1 c02
....................cCaguaaaucucgugcggccu....................................................................... 2 1 c02
....................caaguaaaucucguUcggccu....................................................................... 5 1 c02
....................caaguaaaucucgugcAgccu....................................................................... 2 1 c02
....................caaguaaaucucgugcggccuA...................................................................... 9 1 c02
....................caaguaaaucucgugcgUccug...................................................................... 5 1 c02
....................caaguaaaucAcgugcggccug...................................................................... 2 1 c02
....................caaguaaaucGcgugcggccug...................................................................... 3 1 c02
....................Uaaguaaaucucgugcggccug...................................................................... 6 1 c02
....................caaguaaaucucgugUggccug...................................................................... 7 1 c02
....................caGguaaaucucgugcggccug...................................................................... 2 1 c02
....................caaguaaaucucgugcggccug...................................................................... 8284 0 c02
....................caaguaaaucucgugcgCccug...................................................................... 19 1 c02
....................caaguaaaucucgugcAgccug...................................................................... 2 1 c02
....................caaguaaaucucUugcggccug...................................................................... 2 1 c02
....................caaguaaaucucgugcggccAg...................................................................... 6 1 c02
....................caagGaaaucucgugcggccug...................................................................... 4 1 c02
....................caaguaaaucucgugAggccug...................................................................... 25 1 c02
....................caaCuaaaucucgugcggccug...................................................................... 12 1 c02
....................caaguaaaGcucgugcggccug...................................................................... 4 1 c02
....................caaguaaaucucgugcggcUug...................................................................... 4 1 c02
....................caaguaaaucucgugcggcGug...................................................................... 2 1 c02
....................cCaguaaaucucgugcggccug...................................................................... 2 1 c02
....................caaguaaaCcucgugcggccug...................................................................... 3 1 c02
....................caaguaaaucucgugcggccCg...................................................................... 2 1 c02
....................caaguaaaucucguUcggccug...................................................................... 4 1 c02
....................caaguaaaucCcgugcggccug...................................................................... 3 1 c02
....................caaguaaaucucgCgcggccug...................................................................... 5 1 c02
....................caaguaaCucucgugcggccug...................................................................... 24 1 c02
....................caUguaaaucucgugcggccug...................................................................... 1 1 c02
....................cGaguaaaucucgugcggccug...................................................................... 2 1 c02
....................caaguaaaucucCugcggccug...................................................................... 7 1 c02
....................caaguaaaucuUgugcggccug...................................................................... 6 1 c02
....................caaguaaaucucgugcggccuU...................................................................... 40 1 c02
....................caaguaaaucuAgugcggccug...................................................................... 10 1 c02
....................Aaaguaaaucucgugcggccug...................................................................... 4 1 c02
....................caaguaaaucucgugcggccGg...................................................................... 3 1 c02
....................caagAaaaucucgugcggccug...................................................................... 9 1 c02
....................caaguaaaAcucgugcggccug...................................................................... 4 1 c02
....................caaAuaaaucucgugcggccug...................................................................... 2 1 c02
....................caaUuaaaucucgugcggccug...................................................................... 6 1 c02
....................caaguaaauUucgugcggccug...................................................................... 2 1 c02
....................caaguUaaucucgugcggccug...................................................................... 1 1 c02
....................caaguaaaucucgAgcggccug...................................................................... 5 1 c02
....................caaguaaaucucgGgcggccug...................................................................... 3 1 c02
....................caaguaaaucucgugcggccuC...................................................................... 9 1 c02
```

```
                    Mature                   Star
aagucgauuuaccgucgacacaaguaaaucucgugcggccugucuuaauucaagccgcaagagauuuauuuacaucgaugguaaaagaacaucuguuauuaccggcuacgua
....................caaguaaaucucguAcggccug......................................................................   2      1    c02
....................caaguaaaucucgugcggUcug......................................................................   3      1    c02
....................caaguaaauGucgugcggccug......................................................................   2      1    c02
....................cUaguaaaucucgugcggccug......................................................................   3      1    c02
....................caaguaaGucucgugcggccug......................................................................   1      1    c02
....................caaguaaaucucgugcCgccug......................................................................   8      1    c02
....................caaguaaaucucguCcggccug......................................................................   18     1    c02
....................caaguaaaucucAugcggccug......................................................................   3      1    c02
....................caaguaaaucucgugcUgccug......................................................................   11     1    c02
....................caagCaaaucucgugcggccug......................................................................   2      1    c02
....................caaguaaauAucgugcggccug......................................................................   7      1    c02
....................caaguaaaucucgugcggcAug......................................................................   9      1    c02
....................caaguaUaucucgugcggccug......................................................................   1      1    c02
....................caaguaaaucucgugcggAcug......................................................................   13     1    c02
....................caaguaaaucucgugcgAccug......................................................................   1      1    c02
....................caaguaaaucucguAcggccugu.....................................................................   2      1    c02
....................caaguaaaGcucgugcggccugu.....................................................................   1      1    c02
....................caaguaaaucucgugUggccugu.....................................................................   3      1    c02
....................caagCaaaucucgugcggccugu.....................................................................   1      1    c02
....................caaguaaauUucgugcggccugu.....................................................................   1      1    c02
....................caaguaaCucucgugcggccugu.....................................................................   15     1    c02
....................caaguaaauAucgugcggccugu.....................................................................   6      1    c02
....................caaguaaaucucgugcggccuCu.....................................................................   8      1    c02
....................caaguaaaucucgugcAgccugu.....................................................................   2      1    c02
....................caaguUaaucucgugcggccugu.....................................................................   1      1    c02
....................caaguaaaucucgugcggUcugu.....................................................................   3      1    c02
....................caaguaaaucuUgugcggccugu.....................................................................   4      1    c02
....................caaguaaaucucgugcggccugC.....................................................................   89     1    c02
....................cCaguaaaucucgugcggccugu.....................................................................   2      1    c02
....................caaguaaaucucgugcggcGugu.....................................................................   2      1    c02
....................caaguaaaucucgugcggcUugu.....................................................................   2      1    c02
....................caaguaaaucuAgugcggccugu.....................................................................   14     1    c02
....................caaguaaaucucgugcggccuUu.....................................................................   14     1    c02
....................Gaaguaaaucucgugcggccugu.....................................................................   1      1    c02
....................caaAuaaaucucgugcggccugu.....................................................................   1      1    c02
....................caaguaaaucucgugcggccugG.....................................................................   18     1    c02
....................caaguaaaucucguUcggccugu.....................................................................   5      1    c02
....................caUguaaaucucgugcggccugu.....................................................................   1      1    c02
....................caaguaaaucucgugcgAccugu.....................................................................   1      1    c02
....................caaguaaaucucgugcggGcugu.....................................................................   1      1    c02
....................caaguaaaucucgAgcggccugu.....................................................................   5      1    c02
....................caaguaaaucucgugcggccCgu.....................................................................   1      1    c02
....................caaguaaaucucgugcggccuAu.....................................................................   1      1    c02
....................caagGaaaucucgugcggccugu.....................................................................   1      1    c02
....................caaguaGaucucgugcggccugu.....................................................................   1      1    c02
....................caaguaaaucucgugAggccugu.....................................................................   22     1    c02
....................cUaguaaaucucgugcggccugu.....................................................................   1      1    c02
....................caGguaaaucucgugcggccugu.....................................................................   1      1    c02
....................caaguaaaucucgugcCgccugu.....................................................................   9      1    c02
....................caaguaaaucucCugcggccugu.....................................................................   5      1    c02
....................caaguaaaucucUugcggccugu.....................................................................   3      1    c02
....................caaguaaaucucgugcUgccugu.....................................................................   14     1    c02
....................caaguaaaucucgugcggccAgu.....................................................................   5      1    c02
....................caaguaaaucucgugcggccugu.....................................................................   5553   0    c02
....................caaCuaaaucucgugcggccugu.....................................................................   3      1    c02
....................caaguaaauGucgugcggccugu.....................................................................   3      1    c02
....................caaguaaaucucguCcggccugu.....................................................................   16     1    c02
....................caaguaCaucucgugcggccugu.....................................................................   1      1    c02
....................caaguaaaAcucgugcggccugu.....................................................................   2      1    c02
....................Aaaguaaaucucgugcggccugu.....................................................................   2      1    c02
....................caagAaaaucucgugcggccugu.....................................................................   7      1    c02
....................caaguaaGucucgugcggccugu.....................................................................   1      1    c02
....................caaguaaaucucgugcgCccugu.....................................................................   4      1    c02
....................caaguaaaucucgugcggAcugu.....................................................................   19     1    c02
....................caaguaaaucCcgugcggccugu.....................................................................   1      1    c02
....................caaguaaaucucgugcggccugA.....................................................................   112    1    c02
....................caaguaaaucucgugcggcAugu.....................................................................   3      1    c02
....................caaguaaaucucgugcgUccugu.....................................................................   1      1    c02
....................cGaguaaaucucgugcggccugu.....................................................................   1      1    c02
....................caaguaaaucAcgugcggccugu.....................................................................   6      1    c02
```

```
                    Mature                          Star
aagucgauuuaccgucgacacaaguaaaucucgugcggccugucuuaauucaagccgcaagagauuuauuuacaucgaugguaaaagaacaucuguuauuaccggcuacgua
....................caaguaaaucucAugcggccugu.....................................................................  2    1  c02
....................caaguaaaucuAgugcggccuguc....................................................................  1    1  c02
....................caaguaaaucucgugcggccugCc....................................................................  1    1  c02
....................caaguaaaucucguUcggccuguc....................................................................  1    1  c02
....................caaguaaaucucgugcggccuguc....................................................................  67   0  c02
....................caaguaaaucucgAgcggccuguc....................................................................  1    1  c02
....................caaguaaaucucgugcggccugGc....................................................................  5    1  c02
....................caaguaaaucucgugcggccuguG....................................................................  17   1  c02
....................caaguaaaucucgugcggccuguU....................................................................  618  1  c02
....................caaguaaaucucgugcggccuguA....................................................................  100  1  c02
....................caaguaaaucucgugcggccugAcu...................................................................  1    1  c02
....................caaguaaaucucgugcggccugucC...................................................................  1    1  c02
....................caaguaaaucucgugcggccugucA...................................................................  2    1  c02
....................caaguaaaucucgugcggccuguAu...................................................................  58   1  c02
....................caaguaaaucucgugcggccuguUu...................................................................  462  1  c02
....................caaguaaaucucgugcggccugucu...................................................................  40   0  c02
....................caaguaaaucucgugcggccuguAuu..................................................................  22   1  c02
....................caaguaaaucucgugcggccuguUuu..................................................................  142  1  c02
....................caaguaaaucucgugcggccugucuu..................................................................  9    0  c02
....................caaguaaaucucgugcggccugucuA..................................................................  1    1  c02
....................caaguaaaucucgugcggccugucAu..................................................................  1    1  c02
....................caaguaaaucucgugcggccugucuua.................................................................  3    0  c02
....................caaguaaaucucgugcggccugucuuaauucaagccgcaag...................................................  2    0  c02
.....................aaguaaaucucgugcggc.........................................................................  2    0  c02
.....................aaguaaaucucgugcggcA........................................................................  1    1  c02
.....................aaguaaaucucgugcggcc........................................................................  8    0  c02
.....................Caguaaaucucgugcggccu.......................................................................  4    1  c02
.....................aaguaaaucucgugcggccu.......................................................................  23   0  c02
.....................aaguaaaucuAgugcggccu.......................................................................  1    1  c02
.....................Caguaaaucucgugcggccug......................................................................  2    1  c02
.....................aaguaaaucucgugcggccug......................................................................  63   0  c02
.....................aaguaaaucucCugcggccug......................................................................  1    1  c02
.....................aaguaaaucucgugcCgccug......................................................................  1    1  c02
.....................aaguaaaucucgugcggccugu.....................................................................  44   0  c02
.....................aaguaaaucucgugcgCccugu.....................................................................  1    1  c02
.....................aaguaaaucucgugcggccugA.....................................................................  1    1  c02
.....................aaguaaaucucgugcggccugC.....................................................................  1    1  c02
.....................aGguaaaucucgugcggccugu.....................................................................  1    1  c02
.....................aaguaaaucucgugcggccuguU....................................................................  2    1  c02
.....................Caguaaaucucgugcggccuguc....................................................................  1    1  c02
.....................aaguaaaucucgugcggccuguA....................................................................  2    1  c02
.....................aaguaaaucucgugcggccuguUu...................................................................  4    1  c02
......................aguaaaucucgugcggcc........................................................................  3    0  c02
......................aguaaaucucguCcggccu.......................................................................  1    1  c02
......................aguaaaucucgugcggccu.......................................................................  4    0  c02
......................aguaaaucucgugcggccug......................................................................  4    0  c02
......................aguaaaucucgugcggccuU......................................................................  1    1  c02
......................Cguaaaucucgugcggccug......................................................................  1    1  c02
......................aguaaaucucgugcggccugu.....................................................................  7    0  c02
......................aguaaaucucgugcggccugC.....................................................................  1    1  c02
......................aguaaaucucgugcggccuguU....................................................................  1    1  c02
......................aguaaaucucgugcggccuguUu...................................................................  1    1  c02
.......................guaaaucCcgugcggccu.......................................................................  1    1  c02
.......................guaaaucucgugcggccu.......................................................................  237  0  c02
.......................guaaaucuAgugcggccu.......................................................................  1    1  c02
.......................guaaaucucgugcggUcu.......................................................................  1    1  c02
.......................guaaaucucgugAggccu.......................................................................  1    1  c02
.......................Uuaaaucucgugcggccu.......................................................................  2    1  c02
.......................Cuaaaucucgugcggccu.......................................................................  1    1  c02
.......................guaaaucucgAgcggccu.......................................................................  1    1  c02
.......................guaaCucucgugcggccu.......................................................................  1    1  c02
.......................guaaaucucgugcgUccu.......................................................................  1    1  c02
.......................guaaaucucgugcggAcu.......................................................................  2    1  c02
.......................guaaaucuAgugcggccug......................................................................  1    1  c02
.......................guaaCucucgugcggccug......................................................................  1    1  c02
.......................guaaaucucgugcCgccug......................................................................  1    1  c02
.......................gCaaaucucgugcggccug......................................................................  1    1  c02
.......................guaaaucucCugcggccug......................................................................  1    1  c02
.......................Cuaaaucucgugcggccug......................................................................  8    1  c02
.......................guaaaucucAugcggccug......................................................................  1    1  c02
```

Mature Star

```
aagucgauuuaccgucgacacaaguaaaucucgugcggccugucuuaauucaagccgcaagagauuuauuuacaucgaugguaaaagaacaucuguuauuaccggcuacgua
.......................guaaaucucgugAggccug......................................................................	2	1	c02
.......................guaaaucucguUcggccug......................................................................	1	1	c02
.......................Uuaaaucucgugcggccug......................................................................	3	1	c02
.......................guaaaucucgugcggccug......................................................................	479	0	c02
.......................guaaaucucgugcggccuA......................................................................	1	1	c02
.......................guaaaucucgugcggccuU......................................................................	4	1	c02
.......................guaaaucucgugcggAcug......................................................................	2	1	c02
.......................gGaaaucucgugcggccug......................................................................	1	1	c02
.......................guaaaucucgugUggccug......................................................................	1	1	c02
.......................guaaaucucgCgcggccug......................................................................	2	1	c02
.......................guaaaucucgugcgCccug......................................................................	2	1	c02
.......................guaaaucucgugcggccugG.....................................................................	7	1	c02
.......................guaaaucucgugcggccuCu.....................................................................	1	1	c02
.......................guaaaucucgugcggccugC.....................................................................	5	1	c02
.......................guaaaucucgugAggccugu.....................................................................	2	1	c02
.......................guaaauAucgugcggccugu.....................................................................	2	1	c02
.......................guaaaucucgugcggccugA.....................................................................	4	1	c02
.......................guaaaucucgugcgUccugu.....................................................................	1	1	c02
.......................guaaaucucgugcggccuUu.....................................................................	2	1	c02
.......................guaaaucucgugGggccugu.....................................................................	1	1	c02
.......................Cuaaaucucgugcggccugu.....................................................................	2	1	c02
.......................guaaaucuAgugcggccugu.....................................................................	1	1	c02
.......................guaaaucucgugcggcUugu.....................................................................	1	1	c02
.......................guaaaucucgugcggccugu.....................................................................	277	0	c02
.......................guaaaucucgugcggccuguU....................................................................	19	1	c02
.......................guaaaucucgugcggccugCc....................................................................	1	1	c02
.......................guaaaucucgugcggccuguA....................................................................	5	1	c02
.......................guaaaucucgugcggccuguc....................................................................	2	0	c02
.......................Auaaaucucgugcggccuguc....................................................................	1	1	c02
.......................guaaaucucgugcggccuguAu...................................................................	5	1	c02
.......................guaaaucucgugcggccuguUu...................................................................	18	1	c02
.......................guaaaucucgugcggccugucu...................................................................	1	0	c02
.......................guaaaucucgugcggccuguUuu..................................................................	7	1	c02
........................Gaaaucucgugcggccug......................................................................	10	1	c02
........................uaaaucucgugcggccug......................................................................	86	0	c02
........................uaaaucucgAgcggccug......................................................................	1	1	c02
........................uaaaucucgugcggAcug......................................................................	1	1	c02
........................uaaauAucgugcggccug......................................................................	1	1	c02
........................uaaaucucgugcggccuU......................................................................	1	1	c02
........................uaaaucucgugcggccugA.....................................................................	2	1	c02
........................uaaaucucgugcgCccugu.....................................................................	1	1	c02
........................uaaaucucgugUggccugu.....................................................................	1	1	c02
........................uaaaucucgugcggccugu.....................................................................	49	0	c02
........................Gaaaucucgugcggccugu.....................................................................	8	1	c02
........................Aaaaucucgugcggccugu.....................................................................	2	1	c02
........................uaaaucucgugcggccuguc....................................................................	1	0	c02
........................uaaaucucgugcggccuguA....................................................................	2	1	c02
........................uaaaucucgugcggccuguU....................................................................	4	1	c02
........................uaaaucucgugcggccuguAu...................................................................	1	1	c02
........................uaaaucucgugcggccuguUu...................................................................	3	1	c02
........................uaaaucucgugcggccuguAuu..................................................................	1	1	c02
........................uaaaucucgugcggccuguUuu..................................................................	1	1	c02
.........................aaaucucgugcggccugA.....................................................................	3	1	c02
.........................aaaucucgugcggccugu.....................................................................	27	0	c02
.........................aaaucuAgugcggccugu.....................................................................	1	1	c02
.........................aaaucucgugcggccugG.....................................................................	1	1	c02
.........................Caaucucgugcggccugu.....................................................................	1	1	c02
.........................aaaucucgugcggccuCu.....................................................................	1	1	c02
.........................aaaucucgugcggccuguU....................................................................	3	1	c02
.........................aaaucucgugcggccuguc....................................................................	1	0	c02
.........................aaaucucgugcggccuguUu...................................................................	1	1	c02
.........................aaaucucgugcggccuguAu...................................................................	1	1	c02
.........................aaaucucgugcggccuguUuu..................................................................	3	1	c02
..........................aaucucgugcggccuguA....................................................................	4	1	c02
..........................aaucucgugcggccuguU....................................................................	2	1	c02
..........................aaucucgugcggccuguUu...................................................................	3	1	c02
..........................aaucucgugcggccuguUuu..................................................................	2	1	c02
...........................aucucgugcggccuguUu...................................................................	7	1	c02
...........................aucucgugcggccugucu...................................................................	1	0	c02
...........................aucucgugcggccuguUuu..................................................................	3	1	c02
```

```
                    Mature                          Star
aagucgauuuaccgucgacacaaguaaaucucgugcggccugucuuaauucaagccgcaagagauuuauuuacaucgaugguaaaagaacaucuguuauuaccggcuacgua
...........................aucucgugcggccugucuuaauuca............................................................     1     0     c02
.................................................ucaagccgcaagagauuuauuu.........................................     1     0     c02
....................................................Cgccgcaagagauuuauu..........................................     1     1     c02
....................................................agccgcaagagauuuauuua........................................     1     0     c02
....................................................agccgcaagagauuuauuuac.......................................     3     0     c02
....................................................agccgcaagagauuuauuuacG......................................     5     1     c02
....................................................agccgcaagagauuuauuuacGu.....................................     3     1     c02
......................................................ccgcaagagauuuauuuac.......................................     1     0     c02

................gaAacaaguaaaucucgugcggccu.......................................................................     1     1     c16
................gacUcaaguaaaucucgugcggccugu.....................................................................     1     1     c16
...................acaaguaaaucuAgugcgg..........................................................................     1     1     c16
...................acaaguaaaucucgugcgg..........................................................................     11    0     c16
...................Ccaaguaaaucucgugcgg..........................................................................     2     1     c16
...................acaaguaaaucucgugAgg..........................................................................     1     1     c16
...................Ccaaguaaaucucgugcggc.........................................................................     4     1     c16
...................acaaguaaaucucgugcggc.........................................................................     12    0     c16
...................acGaguaaaucucgugcggcc........................................................................     1     1     c16
...................acaaguaaaucAcgugcggcc........................................................................     1     1     c16
...................acaaguaaCucucgugcggcc........................................................................     2     1     c16
...................acaaguaaaucucgugcggcc........................................................................     66    0     c16
...................Ccaaguaaaucucgugcggcc........................................................................     19    1     c16
...................acaaguaaCucucgugcggccu.......................................................................     1     1     c16
...................acaaguaaaucucgugcUgccu.......................................................................     1     1     c16
...................acaaguaaaucucUugcggccu.......................................................................     1     1     c16
...................acaaguaaaCcucgugcggccu.......................................................................     2     1     c16
...................Ccaaguaaaucucgugcggccu.......................................................................     19    1     c16
...................acaaguaaaucucgugcggccC.......................................................................     1     1     c16
...................acaaguaaaucucgugcggccu.......................................................................     57    0     c16
...................acaaguaaauAucgugcggccug......................................................................     1     1     c16
...................Ccaaguaaaucucgugcggccug......................................................................     3     1     c16
...................acaaguaaaucucgugcggccuU......................................................................     3     1     c16
...................acaaguaaaucucgugcggccug......................................................................     9     0     c16
...................Ucaaguaaaucucgugcggccugu.....................................................................     1     1     c16
...................Ccaaguaaaucucgugcggccugu.....................................................................     4     1     c16
...................acaaguaaaucucgugcggAcugu.....................................................................     1     1     c16
...................acaaguaaaucucgugcggccuUu.....................................................................     4     1     c16
...................acaaguaaaucucgugcggccugu.....................................................................     2     0     c16
...................acaaguaaaucucgugcggccuguU....................................................................     2     1     c16
...................acaaguaaaucucgugcggccuguUuu..................................................................     3     1     c16
....................caaguaaaucucCugcgg..........................................................................     1     1     c16
....................caaguaaaucucgugcgg..........................................................................     55    0     c16
....................caaguaaaucucgugcgC..........................................................................     1     1     c16
....................caaguaaaucucguAcggc.........................................................................     1     1     c16
....................caaguaaaucucgugAggc.........................................................................     1     1     c16
....................caaguaaaucucguCcggc.........................................................................     2     1     c16
....................caaguaaaAcucgugcggc.........................................................................     1     1     c16
....................caaguaaaucucgugcggc.........................................................................     394   0     c16
....................caaguaaaucucgugcggU.........................................................................     1     1     c16
....................cUaguaaaucucgugcggc.........................................................................     1     1     c16
....................caaguaaaucucgugcggA.........................................................................     4     1     c16
....................caaguaaaucuAgugcggcc........................................................................     2     1     c16
....................caaguaaaucucgugcCgcc........................................................................     1     1     c16
....................caaguaaaucucgugcgUcc........................................................................     1     1     c16
....................caaguaaUucucgugcggcc........................................................................     1     1     c16
....................caaguaaaucucgugcUgcc........................................................................     2     1     c16
....................caaUuaaaucucgugcggcc........................................................................     3     1     c16
....................caaguaaaucucguCcggcc........................................................................     2     1     c16
....................caagAaaaucucgugcggcc........................................................................     2     1     c16
....................caaguaaauAucgugcggcc........................................................................     2     1     c16
....................caaguaaaucucAugcggcc........................................................................     1     1     c16
....................caaguaaaucucgugcggcA........................................................................     4     1     c16
....................caaguaaaucucCugcggcc........................................................................     2     1     c16
....................caaguaaaucucguUcggcc........................................................................     1     1     c16
....................Aaaguaaaucucgugcggcc........................................................................     1     1     c16
....................caaguaaaucucgugcggAc........................................................................     3     1     c16
....................caaguaaaucucgugcggGc........................................................................     1     1     c16
....................caaguaaaucAcgugcggcc........................................................................     1     1     c16
....................caaguaaCucucgugcggcc........................................................................     2     1     c16
....................caaguaaaucucgugcggcc........................................................................     898   0     c16
```

```
                    Mature          Star
aagucgauuuaccgucgacacaaguaaaucucgugcggccugucuuaauucaagccgcaagagauuuauuuacaucgaugguaaaagaacaucuguuauuaccggcuacgua
....................cUaguaaaucucgugcggcc........................................................................ 1 1 c16
....................caaguaaaucucgugUggcc........................................................................ 1 1 c16
....................caaguaaaucucgugAggcc........................................................................ 6 1 c16
....................caaguaaaucucgGgcggcc........................................................................ 1 1 c16
....................caaguaaaucucgugcggcU........................................................................ 1 1 c16
....................caaguaaaucucgugcgCcc........................................................................ 2 1 c16
....................caaguaaaucucgCgcggcc........................................................................ 1 1 c16
....................caaguaaaGcucgugcggccu....................................................................... 1 1 c16
....................Aaaguaaaucucgugcggccu....................................................................... 6 1 c16
....................caaguaaauGucgugcggccu....................................................................... 1 1 c16
....................caaUuaaaucucgugcggccu....................................................................... 4 1 c16
....................caagCaaaucucgugcggccu....................................................................... 1 1 c16
....................caaguaaaucucgCgcggccu....................................................................... 2 1 c16
....................caaguaaaucucguAcggccu....................................................................... 1 1 c16
....................caaguaaaucucgugcggcAu....................................................................... 6 1 c16
....................caaguaaaucucgugcggccC....................................................................... 5 1 c16
....................caaguaaaucucgugcAgccu....................................................................... 2 1 c16
....................caagGaaaucucgugcggccu....................................................................... 1 1 c16
....................caaguaUaucucgugcggccu....................................................................... 1 1 c16
....................caaguaaaucucgugcCgccu....................................................................... 3 1 c16
....................caaguaaGucucgugcggccu....................................................................... 1 1 c16
....................cCaguaaaucucgugcggccu....................................................................... 1 1 c16
....................caaguaaaucucgugcgCccu....................................................................... 9 1 c16
....................caaguaaaucucCugcggccu....................................................................... 2 1 c16
....................Gaaguaaaucucgugcggccu....................................................................... 1 1 c16
....................caaguaaaucAcgugcggccu....................................................................... 1 1 c16
....................caaguaaaucucgugcgAccu....................................................................... 2 1 c16
....................caaguaaaucucgugcUgccu....................................................................... 8 1 c16
....................caaguaCaucucgugcggccu....................................................................... 2 1 c16
....................caagAaaaucucgugcggccu....................................................................... 4 1 c16
....................caaguaaaucucAugcggccu....................................................................... 1 1 c16
....................cGaguaaaucucgugcggccu....................................................................... 1 1 c16
....................caaguaaaucuAgugcggccu....................................................................... 9 1 c16
....................caaguGaaucucgugcggccu....................................................................... 3 1 c16
....................caaguaaaAcucgugcggccu....................................................................... 1 1 c16
....................caaguaaaucucgugcgUccu....................................................................... 2 1 c16
....................caGguaaaucucgugcggccu....................................................................... 1 1 c16
....................caaguaaaucucUugcggccu....................................................................... 3 1 c16
....................caaguaaaucucgugcggccu....................................................................... 4580 0 c16
....................caaguaaauAucgugcggccu....................................................................... 2 1 c16
....................cUaguaaaucucgugcggccu....................................................................... 2 1 c16
....................caaguaaaucucgugUggccu....................................................................... 4 1 c16
....................caaCuaaaucucgugcggccu....................................................................... 12 1 c16
....................caaguaaaucucgugcggcUu....................................................................... 5 1 c16
....................caaguaaaucucguCcggccu....................................................................... 9 1 c16
....................caaguaaaucucguUcggccu....................................................................... 4 1 c16
....................caaguaaaucucgugAggccu....................................................................... 14 1 c16
....................caaguaaaucucgugcggccG....................................................................... 3 1 c16
....................Uaaguaaaucucgugcggccu....................................................................... 4 1 c16
....................caaguaaaucucgAgcggccu....................................................................... 1 1 c16
....................caaguaaaucucgugGggccu....................................................................... 1 1 c16
....................caaguaaaucucgugcggccA....................................................................... 5 1 c16
....................caaguaaaucucgugcggcGu....................................................................... 1 1 c16
....................caaguCaaucucgugcggccu....................................................................... 1 1 c16
....................caaguaaaucucgugcggAcu....................................................................... 12 1 c16
....................caaguaaaucucgugcggGcu....................................................................... 2 1 c16
....................caaguaaaucCcgugcggccu....................................................................... 1 1 c16
....................caaguaaauUucgugcggccu....................................................................... 3 1 c16
....................caaguaaCucucgugcggccu....................................................................... 15 1 c16
....................caaguaaaucucgugcggccGg...................................................................... 1 1 c16
....................caaguaaaGcucgugcggccug...................................................................... 1 1 c16
....................caaguaaaucucgCgcggccug...................................................................... 2 1 c16
....................caaguaaaucucgugcggcUug...................................................................... 3 1 c16
....................caaguaaaucucgugcggGcug...................................................................... 2 1 c16
....................caaguaaaucucAugcggccug...................................................................... 3 1 c16
....................caaguaaaucucgGgcggccug...................................................................... 3 1 c16
....................caaguaaaucuGgugcggccug...................................................................... 1 1 c16
....................caaguaaaucucgugcggccuU...................................................................... 130 1 c16
....................caaguaaCucucgugcggccug...................................................................... 27 1 c16
....................caaguaaaucucgugcggcAug...................................................................... 7 1 c16
```

```
                    Mature              Star
aagucgauuuaccgucgacacaaguaaaucucgugcggccugucuuaauucaagccgcaagagauuuauuuacaucgaugguaaaagaacaucuguuauuaccggcuacgua
....................caaguaaaucucgugcggccCg......................................................................	3	1	c16
....................caaguaaauUucgugcggccug......................................................................	1	1	c16
....................caaguaaaucucgugcggccAg......................................................................	2	1	c16
....................caaguGaaucucgugcggccug......................................................................	2	1	c16
....................caaguaaaCcucgugcggccug......................................................................	1	1	c16
....................caaguaaaucuUgugcggccug......................................................................	1	1	c16
....................caaguaaaucucgugcggAcug......................................................................	17	1	c16
....................caaguaaaucucgugcggccug......................................................................	6462	0	c16
....................caaguaaaucucUugcggccug......................................................................	1	1	c16
....................caaguaaaucucgugcgUccug......................................................................	6	1	c16
....................cGaguaaaucucgugcggccug......................................................................	1	1	c16
....................caagAaaaucucgugcggccug......................................................................	5	1	c16
....................caaguaaaucucguAcggccug......................................................................	1	1	c16
....................caaguaaaucucgugcggcGug......................................................................	1	1	c16
....................caaUuaaaucucgugcggccug......................................................................	2	1	c16
....................caaguaaaucucgugcggUcug......................................................................	2	1	c16
....................Aaaguaaaucucgugcggccug......................................................................	3	1	c16
....................caaguaaaucucgugcggccuA......................................................................	6	1	c16
....................caaAuaaaucucgugcggccug......................................................................	1	1	c16
....................caaguaaaucucguCcggccug......................................................................	17	1	c16
....................caaguaaaucGcgugcggccug......................................................................	1	1	c16
....................caaguaaaucucgAgcggccug......................................................................	7	1	c16
....................caaCuaaaucucgugcggccug......................................................................	12	1	c16
....................caaguaaaucucgugUggccug......................................................................	1	1	c16
....................caaguaaaucucgugcggccuC......................................................................	13	1	c16
....................caaguaaaucuAgugcggccug......................................................................	11	1	c16
....................caaguaaaAcucgugcggccug......................................................................	1	1	c16
....................Uaaguaaaucucgugcggccug......................................................................	4	1	c16
....................caaguaaaucucCugcggccug......................................................................	10	1	c16
....................caGguaaaucucgugcggccug......................................................................	1	1	c16
....................caaguaaaucucgugcgAccug......................................................................	3	1	c16
....................caaguaaaucucgugcCgccug......................................................................	11	1	c16
....................caaguaaaucCcgugcggccug......................................................................	1	1	c16
....................caaguaaaucucguUcggccug......................................................................	1	1	c16
....................caagGaaaucucgugcggccug......................................................................	4	1	c16
....................caaguaaaucucgugAggccug......................................................................	16	1	c16
....................caaguaaaucucgugcgCccug......................................................................	14	1	c16
....................caaguUaaucucgugcggccug......................................................................	1	1	c16
....................caaguaaaucAcgugcggccug......................................................................	3	1	c16
....................caaguaCaucucgugcggccug......................................................................	1	1	c16
....................caaguaaaucucgugcUgccug......................................................................	11	1	c16
....................caaguaaauAucgugcggccug......................................................................	2	1	c16
....................cUaguaaaucucgugcggccugu.....................................................................	1	1	c16
....................caaguaaaucuUgugcggccugu.....................................................................	1	1	c16
....................caaguaaaucucgugAggccugu.....................................................................	9	1	c16
....................caaguaaaucucgugcggAcugu.....................................................................	6	1	c16
....................caaguaaaucucgugcggccugG.....................................................................	26	1	c16
....................caaguaaaucucgugcCgccugu.....................................................................	4	1	c16
....................caaguaaaucucgugcgUccugu.....................................................................	2	1	c16
....................caaguaaaucucgCgcggccugu.....................................................................	2	1	c16
....................caUguaaaucucgugcggccugu.....................................................................	1	1	c16
....................caaguaaaucucgugcggccugC.....................................................................	71	1	c16
....................caaguaaaucucgugcgCccugu.....................................................................	5	1	c16
....................caaguaaCucucgugcggccugu.....................................................................	12	1	c16
....................caaguaaaucucgugcggccuUu.....................................................................	34	1	c16
....................caaguaaaucCcgugcggccugu.....................................................................	3	1	c16
....................caaguaaaucucguAcggccugu.....................................................................	1	1	c16
....................caaguaaaucuAgugcggccugu.....................................................................	14	1	c16
....................caaguaaaucGcgugcggccugu.....................................................................	1	1	c16
....................caGguaaaucucgugcggccugu.....................................................................	1	1	c16
....................caaguaaaucucCugcggccugu.....................................................................	8	1	c16
....................caaguaaaucucgugcggUcugu.....................................................................	1	1	c16
....................caaguaaaucucgGgcggccugu.....................................................................	1	1	c16
....................caaguaaaucucgugcggcAugu.....................................................................	10	1	c16
....................caaguaaaucucgugcAgccugu.....................................................................	4	1	c16
....................caagAaaaucucgugcggccugu.....................................................................	3	1	c16
....................caaguaaaAcucgugcggccugu.....................................................................	1	1	c16
....................caaguaaaucucguUcggccugu.....................................................................	3	1	c16
....................caaguaaaCcucgugcggccugu.....................................................................	1	1	c16
....................caaguaaaucucAugcggccugu.....................................................................	3	1	c16
```

```
                    Mature                          Star
aagucgauuuaccgucgacacaaguaaaucucgugcggccugucuuaauucaagccgcaagagauuuauuuacaucgaugguaaaagaacaucuguuauuaccggcuacgua
....................caaguaCaucucgugcggccugu.....................................................................   1      1   c16
....................caaguaaaucucgugcggcUugu.....................................................................   1      1   c16
....................caaguaaaucucgugcggGcugu.....................................................................   1      1   c16
....................caaguaaaucucgugcggccCgu.....................................................................   4      1   c16
....................caaCuaaaucucgugcggccugu.....................................................................   6      1   c16
....................Aaaguaaaucucgugcggccugu.....................................................................   3      1   c16
....................caaguaaaucucgAgcggccugu.....................................................................   5      1   c16
....................caaguaaaucucgugcggccugu.....................................................................   4441   0   c16
....................caagCaaaucucgugcggccugu.....................................................................   1      1   c16
....................caaguaaaucuGgugcggccugu.....................................................................   2      1   c16
....................cGaguaaaucucgugcggccugu.....................................................................   1      1   c16
....................caaguaaauUucgugcggccugu.....................................................................   2      1   c16
....................caaguaaaucucgugcggccuAu.....................................................................   2      1   c16
....................caaguaaaucucgugcggccuCu.....................................................................   9      1   c16
....................caaguaaaucAcgugcggccugu.....................................................................   1      1   c16
....................caaguaaaucucUugcggccugu.....................................................................   6      1   c16
....................Uaaguaaaucucgugcggccugu.....................................................................   7      1   c16
....................caaguaaaucucgugcggccugA.....................................................................   57     1   c16
....................caaguaaaucucgugcUgccugu.....................................................................   9      1   c16
....................caagGaaaucucgugcggccugu.....................................................................   1      1   c16
....................caaguaaauAucgugcggccugu.....................................................................   6      1   c16
....................caaguaaaucucguCcggccugu.....................................................................   2      1   c16
....................caaguaaaucucgugcggccuguU....................................................................   908    1   c16
....................caaguaaaucucgugcggccuguc....................................................................   62     0   c16
....................caaguaaaucucgugcggccugGc....................................................................   4      1   c16
....................caaguaaCucucgugcggccuguc....................................................................   1      1   c16
....................caaguaaaucucgugcggAcuguc....................................................................   1      1   c16
....................caaguaaaucucgugcggccugAc....................................................................   1      1   c16
....................caaguaaaucucgugcggccuguA....................................................................   60     1   c16
....................caaguaaaucucgugcggccuguG....................................................................   5      1   c16
....................caaguaaaucucgugcggccuguAu...................................................................   33     1   c16
....................caaguaaaucucgugcUgccugucu...................................................................   1      1   c16
....................caaguaaaucucgugcggccuguUu...................................................................   750    1   c16
....................caaguaaaucucgugcggccugucC...................................................................   1      1   c16
....................caaguaaaucucgugcggccuguGu...................................................................   1      1   c16
....................caaguaaaucucgugcggccugucu...................................................................   25     0   c16
....................caaguaaaucucgugcggccugucuu..................................................................   3      0   c16
....................caaguaaaucucgugcggccuguUuu..................................................................   301    1   c16
....................caaguaaaucucgugcggccuguAuu..................................................................   17     1   c16
....................caaguaaaucucgugcggccugucuua.................................................................   2      0   c16
....................caaguaaaucucgugcggccugucuuaau...............................................................   1      0   c16
....................caaguaaaucucgugcggccugucuuaauucaagc.........................................................   1      0   c16
....................caaguaaaucucgugcggccugucuuaauucaagcc........................................................   1      0   c16
....................caaguaaaucucgugcggccugucuuaauucaagccgca.....................................................   1      0   c16
....................caaguaaaucucgugcggccugucuuaauucaCgccgcaa....................................................   1      1   c16
....................caaguaaaucucgugcggccugucuuaauucaagccgcaa....................................................   8      0   c16
....................caaguaaaucucgugcggccugucuuaauucaagccgcaaU...................................................   1      1   c16
....................caaguaaaucucgugcggccugucuuaauucaagccgcaag...................................................   12     0   c16
.....................aaguaaaucucgugcggc.........................................................................   1      0   c16
.....................aaguaaaucucgugAggc.........................................................................   1      1   c16
.....................aaAuaaaucucgugcggcc........................................................................   1      1   c16
.....................aaguaaaucucgugcggcc........................................................................   4      0   c16
.....................Caguaaaucucgugcggcc........................................................................   1      1   c16
.....................Caguaaaucucgugcggccu.......................................................................   3      1   c16
.....................aaguaaaucucgugcggccu.......................................................................   35     0   c16
.....................aaguaaaucucguCcggccug......................................................................   1      1   c16
.....................aaguaaaucuAgugcggccug......................................................................   1      1   c16
.....................aaguaaaucucgugcggccug......................................................................   46     0   c16
.....................Caguaaaucucgugcggccug......................................................................   2      1   c16
.....................aaguaaaucucgugcggccuC......................................................................   1      1   c16
.....................aaguaaaucucgCgcggccug......................................................................   1      1   c16
.....................Caguaaaucucgugcggccugu.....................................................................   2      1   c16
.....................aaguaaaucucgugcggccugu.....................................................................   23     0   c16
.....................aaguaaaucucgugcggcAugu.....................................................................   1      1   c16
.....................aaguaaauUucgugcggccugu.....................................................................   1      1   c16
.....................aaguaaaucucgugcggccuguU....................................................................   7      1   c16
.....................aaguaaaucucgugcggccuguUu...................................................................   8      1   c16
.....................aaguaaaucucgugcggccuguUuu..................................................................   2      1   c16
.....................aaguaaaucucgugcggccugucuuaa................................................................   1      0   c16
.....................aaguaaaucucgugcggccugucuuaauucaagccgcaa....................................................   1      0   c16
```

Mature **Star**

aagucgauuuaccgucgacacaaguaaaucucgugcggccugucuuaauucaagccgcaagagauuuauuuacaucgaugguaaaagaacaucuguuauuaccggcuacgua			
......................aguaaaucucgugcggcc..	2	0	c16
......................Cguaaaucucgugcggccu...	2	1	c16
......................aguaaaucucgugcggccu...	5	0	c16
......................aguaaaucucgugcggccug..	8	0	c16
......................aguaaaucucgugcggccugu...	3	0	c16
......................aguaaaucucgugcggccuCu...	1	1	c16
......................aguaaaucucgugcggccugC...	1	1	c16
......................aguaaaucucgugcggccuguU..	2	1	c16
......................aguaaaucucgugcggccugucuuaauucaagcc..	1	0	c16
.......................guaaaucucgugcggAcu...	1	1	c16
.......................guaaaucucgugAggccu...	1	1	c16
.......................guaaaucucgugcgUccu...	1	1	c16
.......................guaaaucuUgugcggccu...	1	1	c16
.......................guaaaucucgugGggccu...	1	1	c16
.......................guaaCucucgugcggccu...	1	1	c16
.......................guaaaucucgugcgCccu...	1	1	c16
.......................guaaaucucgugcggccu...	147	0	c16
.......................guaaaucucgugcggccA...	1	1	c16
.......................Auaaaucucgugcggccug..	1	1	c16
.......................guaaaucuAgugcggccug..	2	1	c16
.......................guaaaucucgugcAgccug..	1	1	c16
.......................guaaaucucgugcggccuU..	3	1	c16
.......................guaaaucucgugcggccuC..	1	1	c16
.......................guaaaucucgugcggccuA..	1	1	c16
.......................guaaaucucgugcggccug..	286	0	c16
.......................guaaauUucgugcggccug..	1	1	c16
.......................gCaaaucucgugcggccug..	1	1	c16
.......................guaaaucucgugAggccug..	1	1	c16
.......................Uuaaaucucgugcggccug..	1	1	c16
.......................guaaaucucCugcggccug..	2	1	c16
.......................guaaaucucgugcggAcug..	1	1	c16
.......................guaaaucucguUcggccugu...	1	1	c16
.......................guaaaucucgugcggccugA...	4	1	c16
.......................Uuaaaucucgugcggccugu...	3	1	c16
.......................guaaaucucgugcggccuUu...	3	1	c16
.......................guaaaucuAgugcggccugu...	1	1	c16
.......................guaaaucucgugcggccugu...	173	0	c16
.......................guaaaucucgugcUgccugu...	1	1	c16
.......................guaaaucucgugcggccugC...	4	1	c16
.......................guaaaucucUugcggccugu...	1	1	c16
.......................guaaaucuUgugcggccugu...	1	1	c16
.......................guaaaucucgugAggccugu...	1	1	c16
.......................guaaaucucgugcggcUugu...	1	1	c16
.......................guaaaucucgugcggAcugu...	1	1	c16
.......................guaaaucucgugcggccuguc..	3	0	c16
.......................guaaaucucgugcggccuguU..	29	1	c16
.......................guaaaucucgugcggccugucu...	3	0	c16
.......................guaaaucucgugcggccuguAu...	1	1	c16
.......................guaaaucucgugcggccuguUu...	19	1	c16
.......................guaaaucucgugcggccugucuu..	2	0	c16
.......................guaaaucucgugcggccuguUuu..	11	1	c16
.......................guaaaucucgugcggccugucuuaauucaagccgcaag...	1	0	c16
........................Aaaaucucgugcggccug..	1	1	c16
........................uaaaucucgugcggccuU..	1	1	c16
........................Gaaaucucgugcggccug..	4	1	c16
........................uaaaucucgugcggcAug..	1	1	c16
........................uaaaucucgugcggccug..	35	0	c16
........................Gaaaucucgugcggccugu...	4	1	c16
........................uaaaucucgugcggccugu...	26	0	c16
........................uaaaucucgugcggccuguU..	6	1	c16
........................uaaaucucgugcggccuguc..	2	0	c16
........................uaaaucucgugcggccuguA..	1	1	c16
........................uaaaucucgugcggccuguUu...	3	1	c16
........................uaaaucucgugcggccuguUuu..	4	1	c16
.........................aaaucucgugcggAcugu...	1	1	c16
.........................Caaucucgugcggccugu...	5	1	c16
.........................aaaucucgugcggccugu...	29	0	c16
.........................aaaucucgugcggccuguU..	2	1	c16
.........................aaaucucgugcggccuguUu...	6	1	c16
.........................aaaucucgugcggccuguAu...	2	1	c16

Mature Star

aagucgauuuaccgucgacacaaguaaaucucgugcggccugucuuaauucaagccgcaagagauuuauuuacaucgaugguaaaagaacaucuguuauuaccggcuacgua

.........................aaaucucgugcggccuguUuu..	2	1	c16
..........................aaucucgugcggccuguU..	4	1	c16
..........................aaucucgugcggccuguUu...	2	1	c16
..........................aaucucgugcggccugucu...	2	0	c16
..........................aaucucgugcggccuguUuu..	2	1	c16
...........................aucucgugcggccuguUu...	4	1	c16
...........................aucucgugcggccugucu...	1	0	c16
...........................aucucgugcggccuguUuu..	5	1	c16
............................ucucgugcggccuguUuu..	2	1	c16
....Cgccgcaagagauuuauuuac.......................................	1	1	c16
....agccgcaagagauuuauuuac.......................................	2	0	c16
....agccgcaagagauuuauuuacU......................................	2	1	c16
....agccgcaagagauuuauuuacG......................................	5	1	c16
....agccgcaagagauuuauuuacGu.....................................	1	1	c16
....ccgcaagagauuuauuuacGu.....................................	1	1	c16
....cgcaagagauuuauuuacG......................................	1	1	c16
....gcaagagauuuauuuacGu.....................................	1	1	c16
...................acaaguaaaucucgugcggcc..	2	0	c04
...................acaaguaaaucucgugcggccu...	1	0	c04
...................acaaguaaaucucgugcggccug..	1	0	c04
....................caaguaaaucucgugcggc...	2	0	c04
....................caaguaaaucucgugcggcc..	13	0	c04
....................caaguaaaucucgugcggccu...	30	0	c04
....................caaguaaaucucgugcggccC...	1	1	c04
....................caaguaaaucucgugcggccuU..	3	1	c04
....................caaguaaaucucgugcggccug..	74	0	c04
....................caaguaaaucucgugcggccugC...	1	1	c04
....................caaguaaaucucgugcggccuUu...	1	1	c04
....................caaguaaaucucgugcggccugu...	31	0	c04
....................caaguaaaucucgugcggccuguU..	5	1	c04
....................caaguaaaucucgugcggccuguUu...	2	1	c04
....................caaguaaaucucgugcggccuguAu...	1	1	c04
....................caaguaaaucucgugcggccugucuuaauucaagcc..	1	0	c04
.......................guaaaucucgugcggccu...	1	0	c04
.......................guaaaucucgugcggccug..	1	0	c04
.......................guaaaucucgugcggccugu...	1	0	c04
........................uaaaucucgugcggccug..	1	0	c04
.........................aaaucucgugcggccugu...	1	0	c04
...............cgaGacaaguaaaucucgugcggccugu...	1	1	c09
................gacCcaaguaaaucucgugcggccug..	1	1	c09
...................acaaguaaaucucgugcg...	1	0	c09
...................Ccaaguaaaucucgugcgg..	1	1	c09
...................acaaguaaaucucgugcggc...	2	0	c09
...................acaaguaaCucucgugcggcc..	1	1	c09
...................acaaguaaauAucgugcggcc..	1	1	c09
...................acaaguaaaucucgugcggcc..	37	0	c09
...................acaaguaaaucucgugcggccA...	3	1	c09
...................acaaguaaaucucgugcggccu...	34	0	c09
...................Ccaaguaaaucucgugcggccu...	5	1	c09
...................acaaguaaaucucgugcggccug..	6	0	c09
...................Ccaaguaaaucucgugcggccug..	2	1	c09
...................acaaguaaaucucguCcggccug..	1	1	c09
...................Ucaaguaaaucucgugcggccug..	1	1	c09
...................acaaguaaaucucgugcggccuU..	6	1	c09
...................acaaguaaaucucgugcggccuUu...	1	1	c09
...................acaaguaaaucucgugcggccugu...	3	0	c09
...................acaaguaaaucucgugcggccuguU..	1	1	c09
....................caaguaaaucucgugcgg..	31	0	c09
....................caaguaaaucucguUcggc...	1	1	c09
....................caaguaaaucucgugcggc...	121	0	c09
....................caaguaaaucucgugcCgc...	1	1	c09
....................caaguaaaucucgugcUgc...	1	1	c09
....................caaguaaaucucgugAggc...	1	1	c09
....................caaUuaaaucucgugcggc...	1	1	c09
....................caaguaaaucucgugcggcG..	1	1	c09
....................caaguaaaucucgugcgCcc..	1	1	c09
....................caaguaaaucucAugcggcc..	1	1	c09
....................caaguaaaucucCugcggcc..	1	1	c09

```
                    Mature                        Star
aagucgauuuaccgucgacacaaguaaaucucgugcggccugucuuaauucaagccgcaagagauuuauuuacaucgaugguaaaagaacaucuguuauuaccggcuacgua
....................caaCuaaaucucgugcggcc........................................................................    1      1     c09
....................caaguaaaucucgugcgUcc........................................................................    1      1     c09
....................caaguaaaucucgugcggcA........................................................................    1      1     c09
....................caaguaaaucucgugcggUc........................................................................    1      1     c09
....................caaguaaaucucUugcggcc........................................................................    1      1     c09
....................caaguaaaucucgGgcggcc........................................................................    1      1     c09
....................caaguUaaucucgugcggcc........................................................................    1      1     c09
....................caaguaaaucucgugcggcc........................................................................    443    0     c09
....................caaguaaaucuAgugcggcc........................................................................    1      1     c09
....................caaguaaaucucgugUggccu.......................................................................    3      1     c09
....................caaguaaaucucgugcggcAu.......................................................................    1      1     c09
....................caaguaaaucucgugcggccG.......................................................................    3      1     c09
....................caaguaaaucucgugcCgccu.......................................................................    2      1     c09
....................caagCaaaucucgugcggccu.......................................................................    1      1     c09
....................caaguaaaucucgAgcggccu.......................................................................    3      1     c09
....................caaguaaaucGcgugcggccu.......................................................................    1      1     c09
....................caaguaaaucucgugcggAcu.......................................................................    5      1     c09
....................Aaaguaaaucucgugcggccu.......................................................................    1      1     c09
....................caaguaaaucucgGgcggccu.......................................................................    1      1     c09
....................caaguaaaucuAgugcggccu.......................................................................    6      1     c09
....................caaguaaaAcucgugcggccu.......................................................................    1      1     c09
....................caaguaaauUucgugcggccu.......................................................................    1      1     c09
....................caaCuaaaucucgugcggccu.......................................................................    2      1     c09
....................caaguaaaucucgugcUgccu.......................................................................    4      1     c09
....................caaguaaaucucgugcAgccu.......................................................................    1      1     c09
....................caaguaaaucucgugcggccu.......................................................................    1994   0     c09
....................caaguaaaucucgugAggccu.......................................................................    3      1     c09
....................caaguaaauAucgugcggccu.......................................................................    1      1     c09
....................caaguaaCucucgugcggccu.......................................................................    5      1     c09
....................caaguaaaucucgugcggccA.......................................................................    3      1     c09
....................caaguaaaucucguUcggccu.......................................................................    2      1     c09
....................caaguaaaucucgCgcggccu.......................................................................    1      1     c09
....................caUguaaaucucgugcggccu.......................................................................    2      1     c09
....................caaguaaaucAcgugcggccu.......................................................................    1      1     c09
....................caaguaaaGcucgugcggccu.......................................................................    1      1     c09
....................caaguaaaucucCugcggccu.......................................................................    3      1     c09
....................caaguaaaucucgugcgCccu.......................................................................    1      1     c09
....................caaguaaaucucgugcggccC.......................................................................    7      1     c09
....................caaguaaaucucguCcggccu.......................................................................    8      1     c09
....................caaguaaaucucgAgcggccug......................................................................    4      1     c09
....................caaguaaaucucgugcggccAg......................................................................    1      1     c09
....................caagGaaaucucgugcggccug......................................................................    1      1     c09
....................caaguaaaucuGgugcggccug......................................................................    1      1     c09
....................caaguaaaCcucgugcggccug......................................................................    1      1     c09
....................caaguaaaucucgugcCgccug......................................................................    7      1     c09
....................caaguaaaucucgCgcggccug......................................................................    1      1     c09
....................caaguaaaucucgugcgAccug......................................................................    1      1     c09
....................caaguaaaucucgugcUgccug......................................................................    7      1     c09
....................caaguaaCucucgugcggccug......................................................................    5      1     c09
....................caaguaaaucucguAcggccug......................................................................    1      1     c09
....................caaguaaaucucgugAggccug......................................................................    14     1     c09
....................caaguaaaAcucgugcggccug......................................................................    2      1     c09
....................caagCaaaucucgugcggccug......................................................................    3      1     c09
....................caaguaaaucucgugcggccuC......................................................................    9      1     c09
....................caaguaaaucucgugcggccuU......................................................................    37     1     c09
....................caaguaaaGcucgugcggccug......................................................................    1      1     c09
....................caaguaaaucucUugcggccug......................................................................    2      1     c09
....................caaguaaauAucgugcggccug......................................................................    2      1     c09
....................caaguaaaucuUgugcggccug......................................................................    1      1     c09
....................caaguaaaucucgugcgCccug......................................................................    8      1     c09
....................caaguaaGucucgugcggccug......................................................................    3      1     c09
....................caaguaaaucucgugUggccug......................................................................    2      1     c09
....................Uaaguaaaucucgugcggccug......................................................................    1      1     c09
....................Gaaguaaaucucgugcggccug......................................................................    2      1     c09
....................caaguaaaucucgugcggccuA......................................................................    5      1     c09
....................caaguaaaucucgugcggAcug......................................................................    4      1     c09
....................caaguaaaucucguUcggccug......................................................................    3      1     c09
....................caagAaaaucucgugcggccug......................................................................    5      1     c09
....................caaguaaaucucAugcggccug......................................................................    3      1     c09
....................cUaguaaaucucgugcggccug......................................................................    1      1     c09
```

Mature Star

```
aagucgauuuaccgucgacacaaguaaaucucgugcggccugucuuaauucaagccgcaagagauuuauuuacaucgaugguaaaagaacaucuguuauuaccggcuacgua
....................caaguaaaucuCugcggccug......................................................................   5     1   c09
....................caaguaaaucucgugcggcAug......................................................................   9     1   c09
....................caaguaaUucucgugcggccug......................................................................   3     1   c09
....................caaguCaaucucgugcggccug......................................................................   1     1   c09
....................caaguaaaucucgugcggccug......................................................................   3599  0   c09
....................caaguaaaucucguCcggccug......................................................................   9     1   c09
....................caaguaaaucucgugcggcUug......................................................................   1     1   c09
....................caaguaaaucucgugcggccCg......................................................................   4     1   c09
....................caaguUaaucucgugcggccug......................................................................   1     1   c09
....................caaguaaaucAcgugcggccug......................................................................   2     1   c09
....................Aaaguaaaucucgugcggccug......................................................................   2     1   c09
....................caaUuaaaucucgugcggccug......................................................................   1     1   c09
....................caaguaaaucucgugcggccGg......................................................................   1     1   c09
....................caaguaaaucuAgugcggccug......................................................................   4     1   c09
....................caaguaaaucucgugcgUccug......................................................................   2     1   c09
....................caaguaaaucucgugcggccugC.....................................................................   30    1   c09
....................caaguaaaucucgugcggccugA.....................................................................   49    1   c09
....................caaguaaaucucgugcgCccugu.....................................................................   1     1   c09
....................caaguaaaucucgugcggccuCu.....................................................................   3     1   c09
....................caaguaaaucucgugcggccGgu.....................................................................   3     1   c09
....................caaguaaaucucgugcgUccugu.....................................................................   2     1   c09
....................caaguaaaucucAugcggccugu.....................................................................   2     1   c09
....................caaguaaaucucgugcggccugG.....................................................................   21    1   c09
....................caaguaaaucucgugUggccugu.....................................................................   4     1   c09
....................caaguaaaAcucgugcggccugu.....................................................................   1     1   c09
....................caaguUaaucucgugcggccugu.....................................................................   1     1   c09
....................caaguaaaucucgugcUgccugu.....................................................................   3     1   c09
....................caaguaaaucucgugcggGcugu.....................................................................   1     1   c09
....................caaguaaaucucgugcggcAugu.....................................................................   1     1   c09
....................caagCaaaucucgugcggccugu.....................................................................   1     1   c09
....................caaguaaaucucguUcggccugu.....................................................................   1     1   c09
....................caUguaaaucucgugcggccugu.....................................................................   1     1   c09
....................caaguaaaucucgugcggccuAu.....................................................................   5     1   c09
....................caaguaaaucucgugAggccugu.....................................................................   4     1   c09
....................caaguaaaucucgugcggccuUu.....................................................................   4     1   c09
....................caaguaaaucucgugcggAcugu.....................................................................   4     1   c09
....................caaguaaaucCcgugcggccugu.....................................................................   2     1   c09
....................caaguaaaucucgugcgAccugu.....................................................................   1     1   c09
....................caaguaaCucucgugcggccugu.....................................................................   5     1   c09
....................caaguaaaucucgugcggcUugu.....................................................................   1     1   c09
....................caaguaaaucucguCcggccugu.....................................................................   1     1   c09
....................caaguaaaucucgugcggccugu.....................................................................   1554  0   c09
....................caaguaaaucucgAgcggccugu.....................................................................   1     1   c09
....................caagGaaaucucgugcggccugu.....................................................................   1     1   c09
....................caaguaaaucucgugcggUcugu.....................................................................   1     1   c09
....................cCaguaaaucucgugcggccugu.....................................................................   2     1   c09
....................caaguaaaucucgugcggccuguU....................................................................   193   1   c09
....................caaguaaaucucgugcggUcuguc....................................................................   1     1   c09
....................caaguaaaucucgugcggccuguG....................................................................   7     1   c09
....................caaguaaaucucgugcggccuguA....................................................................   27    1   c09
....................caaguaaaucucgugcggccugGc....................................................................   7     1   c09
....................caaguaaaucucgugcggccuguc....................................................................   29    0   c09
....................caaguaaaucucgugcggccugucu...................................................................   13    0   c09
....................caaguaaaucucgugcggccugucA...................................................................   1     1   c09
....................caaguaaaucucgugcggccuguUu...................................................................   151   1   c09
....................caaguaaaucucgugcCgccugucu...................................................................   1     1   c09
....................caaguaaaucucgugcggccuguAu...................................................................   13    1   c09
....................caaguaaaucucgugcggccugucuu..................................................................   2     0   c09
....................caaguaaaucucgugcggccuguUuu..................................................................   53    1   c09
....................caaguaaaucucgugcggccugucAu..................................................................   1     1   c09
....................caaguaaaucucgugcggccuguAuu..................................................................   8     1   c09
....................caaguaaaucucgugcggccugucuuU.................................................................   2     1   c09
....................caaguaaaucucgugcggccugucuuaauucaagccgcaag...................................................   1     0   c09
.....................aaguaaaucucgugcggc.........................................................................   1     0   c09
.....................Caguaaaucucgugcggcc........................................................................   1     1   c09
.....................aaguaaaucucgugcggcc........................................................................   3     0   c09
.....................aaguaaaucucgugcggccu.......................................................................   9     0   c09
.....................aaguaaaucucgugcggccuU......................................................................   1     1   c09
.....................aUguaaaucucgugcggccug......................................................................   1     1   c09
.....................aaguaaaucucgugcggccug......................................................................   30    0   c09
```

```
                    Mature                 Star
aagucgauuuaccgucgacacaaguaaaaucucgugcggccugucuuaauucaagccgcaagagauuuauuuacaucgaugguaaaagaacaucuguuauuaccggcuacgua
.....................aaguaaaaucucgugcggccuC......................................................................   1    1   c09
.....................Caguaaaaucucgugcggccugu.....................................................................   1    1   c09
.....................aaguaaaaucucgugcggccugu.....................................................................   11   0   c09
.....................aaguaaaaucucgugcggccuguUu...................................................................   1    1   c09
......................aguaaaaucucgugcggcc........................................................................   1    0   c09
......................aguaaaaucucgugcgCccu.......................................................................   1    1   c09
......................aguaaaaucucgugcggccu.......................................................................   4    0   c09
......................aguaaaaucucgugcgCccug......................................................................   1    1   c09
......................aguaaaaucucgugcggccuA......................................................................   1    1   c09
......................aguaaaaucucgugcggccug......................................................................   4    0   c09
......................aguaaaaucucgugcggccugC.....................................................................   1    1   c09
......................aguaaaaucucgugcggccugu.....................................................................   3    0   c09
......................aguaaaaucucgugcggccuguUuu..................................................................   2    1   c09
.......................Cuaaaucucgugcggccu........................................................................   3    1   c09
.......................guaaaucucgugcggccA........................................................................   1    1   c09
.......................guaaaucucgugcggccG........................................................................   1    1   c09
.......................guaaaucucgugcggUcu........................................................................   1    1   c09
.......................guaaaucucgugAggccu........................................................................   2    1   c09
.......................guaaaucucgGgcggccu........................................................................   1    1   c09
.......................guaaaucucgugcggccu........................................................................   213  0   c09
.......................guaaaucucgAgcggccu........................................................................   1    1   c09
.......................guaaaucucgugcggcUug.......................................................................   1    1   c09
.......................guaaaucuAgugcggccug.......................................................................   1    1   c09
.......................Cuaaaucucgugcggccug.......................................................................   7    1   c09
.......................guaaaucucUugcggccug.......................................................................   1    1   c09
.......................guaaCucucgugcggccug.......................................................................   1    1   c09
.......................guaaaucucgugcggccuC.......................................................................   2    1   c09
.......................guaaaucucgugcggccug.......................................................................   347  0   c09
.......................Uuaaaucucgugcggccug.......................................................................   3    1   c09
.......................guaaaucucguUcggccug.......................................................................   2    1   c09
.......................guaaaucucgugcUgccug.......................................................................   1    1   c09
.......................guaaaucAcgugcggccug.......................................................................   1    1   c09
.......................guaaauAucgugcggccug.......................................................................   1    1   c09
.......................guaaaucucgugcggccuU.......................................................................   3    1   c09
.......................guaaaucucgugAggccug.......................................................................   1    1   c09
.......................guaaaucucCugcggccug.......................................................................   1    1   c09
.......................guaaaucucgugcggAcugu......................................................................   1    1   c09
.......................guaaaucucgugcggccugA......................................................................   4    1   c09
.......................guaaaucucgugcggccuUu......................................................................   1    1   c09
.......................Uuaaaucucgugcggccugu......................................................................   1    1   c09
.......................guUaaucucgugcggccugu......................................................................   1    1   c09
.......................guaaaucucgugcggccugu......................................................................   148  0   c09
.......................guaaaucucgugcggccugC......................................................................   3    1   c09
.......................guaaaucucUugcggccugu......................................................................   1    1   c09
.......................gGaaaucucgugcggccugu......................................................................   1    1   c09
.......................guaaaucucgugcggccugG......................................................................   2    1   c09
.......................guaaauUucgugcggccugu......................................................................   1    1   c09
.......................guaaaucucgugAggccugu......................................................................   1    1   c09
.......................guaaaucucgugcggccuguU.....................................................................   15   1   c09
.......................guaaaucucgugcggccuguA.....................................................................   3    1   c09
.......................guaaaucucgugcggccuguc.....................................................................   2    0   c09
.......................guaaaucucgugcggccugGc.....................................................................   1    1   c09
.......................guaaaucucgugcggccugCc.....................................................................   2    1   c09
.......................guaaaucucgugcggccuguAu....................................................................   2    1   c09
.......................guaaaucucgugcggccuguUu....................................................................   7    1   c09
.......................guaaaucucgugcggccugucu....................................................................   2    0   c09
.......................guaaaucucgugcggccuguUuu...................................................................   3    1   c09
.......................guaaaucucgugcggccuguAuu...................................................................   1    1   c09
.......................guaaaucucgugcggccugucuu...................................................................   1    0   c09
........................uGaaucucgugcggccug.......................................................................   1    1   c09
........................uaaaucucgugcggccuU.......................................................................   2    1   c09
........................uaaaucucgugcggccug.......................................................................   54   0   c09
........................uaaaucucgAgcggccug.......................................................................   1    1   c09
........................uaaaCcucgugcggccug.......................................................................   1    1   c09
........................Gaaaucucgugcggccug.......................................................................   1    1   c09
........................uaaaucucgugcAgccug.......................................................................   1    1   c09
........................uaaaucucgugcggccugu......................................................................   31   0   c09
........................uaaaucucgugcggccugC......................................................................   2    1   c09
........................uaaaucucgugcUgccugu......................................................................   1    1   c09
........................uaaaucucgugcggccuguA.....................................................................   1    1   c09
```

Mature Star

```
aagucgauuuaccgucgacacaaguaaaucucgugcggccugucuuaauucaagccgcaagagauuuauuuacaucgaugguaaaagaacaucuguuauuaccggcuacgua
........................uaaaucucgugcggccuguc....................................................................    1    0    c09
........................uaaaucucgugcggccuguU....................................................................    3    1    c09
........................uaaaucucgugcggccuguUu...................................................................    2    1    c09
........................uaaaucucgugcggccuguUuu..................................................................    2    1    c09
.........................Caaucucgugcggccugu.....................................................................    1    1    c09
.........................aaaucucgugcggccugC.....................................................................    3    1    c09
.........................aaaucucgugcggccugA.....................................................................    2    1    c09
.........................aaaucucgugcggccugu.....................................................................   38    0    c09
.........................aaaucucgugcggccugG.....................................................................    2    1    c09
.........................aaaucucgugcggccuguA....................................................................    3    1    c09
.........................aaaucucgugcggccuguG....................................................................    1    1    c09
.........................aaaucucgugcggccuguU....................................................................    5    1    c09
.........................aaaucucgugcggccuguUu...................................................................    2    1    c09
.........................aaaucucgugcggccuguAuu..................................................................    1    1    c09
..........................aaucucgugcggccuguU....................................................................    2    1    c09
..........................aaucucgugcggccuguUu...................................................................    1    1    c09
..........................aaucucgugcggccuguAu...................................................................    1    1    c09
...........................aucucgugcggccuguUu...................................................................    8    1    c09
...........................aucucgugcggccuguAu...................................................................    2    1    c09
...........................aucucgugcggccuguUuu..................................................................    1    1    c09
...........................aucucgugcggccugucuu..................................................................    1    0    c09
....................................................agccgcaagagauuuauuuac.......................................    3    0    c09
....................................................agccgcaagagauuuauuuacG......................................    1    1    c09
......................................................ccgcaagagauuuauuuacGu.....................................    1    1    c09
```

Provisional ID	:	group0_1277
Score total	:	310.4
Score for star read(s)	:	3.9
Score for read counts	:	309.6
Score for mfe	:	-0.2
Score for randfold	:	-2.2
Score for cons. seed	:	-0.6
Total read count	:	619
Mature read count	:	614
Loop read count	:	0
Star read count	:	5

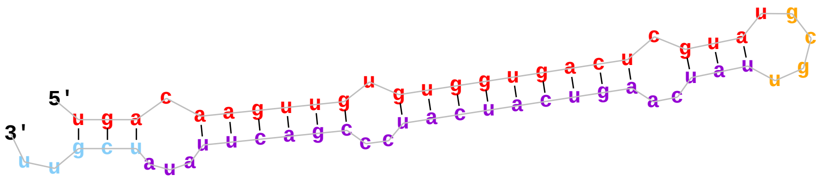

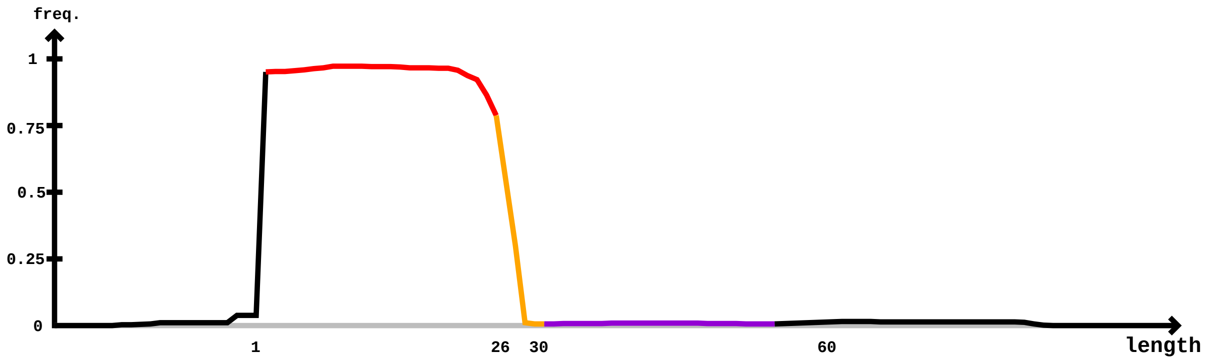

Mature **Star**

```
5'- aguggugcauagaggaaucaugacaaguuguguggugacucguaugcguuaucaagucaucaucccgacuuauaucguuauucccgaccccaacaugcauguucguguccgacag  -3'   obs
    aguggugcauagaggaaucaugacaaguuguguggugacucguaugcguuaucaagucaucaucccgacuuauaucguuauucccgaccccaacaugcauguucguguccgacag         exp
    ..(((.(....(.(((((.((((.((((((.(((((((((.(((.....)))..)))))))))..))))))...)))).))))))...))))(((((......))))).......   reads   mm   sample
    .....ugcauagaggaaucaugacaa.........................................................................................   1       0    c04
    .....ugcauagaggaaucaugacaaguugugu..................................................................................   1       0    c04
    .......cauagaggaaucaugacaag........................................................................................   1       0    c04
    ........auagaggaaucaugacaagu.......................................................................................   1       0    c04
    .........uagaggaaucaugacaaguugugug.................................................................................   1       0    c04
    .........uagaggaaucaugacaaguuguguggg...............................................................................   2       0    c04
    .................ucaugacaaguuguguggugacuc..........................................................................   2       0    c04
    .................ucaugacaaguuguguggugacucg.........................................................................   4       0    c04
    .................ucaugacaaguuguguggugacucgu........................................................................   10      0    c04
    .................ucaugacaaguuguguggugacucguU.......................................................................   1       1    c04
    .................ucaugacaaguuguguggugacucguaugcgu..................................................................   1       0    c04
    ....................ugacaaguugugugguga.............................................................................   1       0    c04
    ....................ugacaaguuguguggugacu...........................................................................   5       0    c04
    ....................ugacaaguuguguggugacuc..........................................................................   9       0    c04
    ....................Ggacaaguuguguggugacuc..........................................................................   2       1    c04
    ....................ugacaaguuguguggugacucg.........................................................................   7       0    c04
    ....................ugacaaguuguguggugacucgu........................................................................   24      0    c04
    ....................Ggacaaguuguguggugacucgu........................................................................   1       1    c04
    ....................ugacaaguuguguggugacucgA........................................................................   1       1    c04
    ....................Ggacaaguuguguggugacucgua.......................................................................   1       1    c04
    ....................ugacaaguuguguggugacucgua.......................................................................   43      0    c04
    ....................ugacaaguuguguggugacucguU.......................................................................   1       1    c04
    ....................ugacaaguuguguggugacucguUu......................................................................   2       1    c04
    ....................ugacaaguuguguggugacAcguau......................................................................   1       1    c04
    ....................ugacaaguuguguggugacucguGu......................................................................   1       1    c04
    ....................Ggacaaguuguguggugacucguau......................................................................   19      1    c04
    ....................ugacaaguuguguggugacucguau......................................................................   232     0    c04
    ....................ugacaaguugugugGgacucguau.......................................................................   1       1    c04
    ....................Agacaaguuguguggugacucguau......................................................................   1       1    c04
    ....................ugacaaguuguguggugacucguauU.....................................................................   2       1    c04
    ....................ugacaaguuguguggugacucgGaug.....................................................................   1       1    c04
    ....................Ggacaaguuguguggugacucguaug.....................................................................   2       1    c04
    ....................ugacaaguuguguggugUcucguaug.....................................................................   1       1    c04
```

Mature Star

```
aguggugcauagaggaaucaugacaaguuguguggugacucguaugcguuaucaagucaucaucccgacuuauaucguuauucccgaccccaacaugcauguucguguccgacag
....................ugacaaguuguguggugacucguauA.....................................................................  6    1  c04
....................ugacaaguuguguggugacucguaug.....................................................................  25   0  c04
....................ugacaaguuguguggugacucguauC.....................................................................  1    1  c04
....................Agacaaguuguguggugacucguaugc....................................................................  1    1  c04
....................ugacaaguuguguggugacucguaugG....................................................................  1    1  c04
....................ugacaaguuguguggugacucguaugc....................................................................  157  0  c04
....................ugacaaguuguguggugacucAuaugc....................................................................  1    1  c04
....................ugacaaguuguguggugacucCuaugc....................................................................  1    1  c04
....................ugacaaCuuguguggugacucguaugc....................................................................  1    1  c04
....................Ggacaaguuguguggugacucguaugc....................................................................  15   1  c04
....................ugacaaguuguguggugacucguaCgc....................................................................  1    1  c04
....................ugacaaguuguguggugacucguaugcC...................................................................  1    1  c04
....................ugacaaguuguguggugacucguaugcA...................................................................  1    1  c04
....................ugacaaguuguguggugacucguaugcU...................................................................  1    1  c04
....................ugacaaguuguguggugacucguaugcgu..................................................................  1    0  c04
....................ugacaaguuguguggugacucguaugcguua................................................................  1    0  c04
.....................gacaaguuguguggugacucguaugc....................................................................  1    0  c04
.......................caaguuguguggugacucguau......................................................................  1    0  c04
.......................caaguuguguggugacucguaugc....................................................................  1    0  c04
........................aaguuguguggugacucguau......................................................................  1    0  c04
........................aaguuguguggugacucguaugc....................................................................  1    0  c04
.........................aguuguguggugacucgua.......................................................................  1    0  c04
.........................aguuguguggugacucguau......................................................................  1    0  c04
.........................aguuguguggugacucguaug.....................................................................  1    0  c04
..........................Cuuguguggugacucguau......................................................................  1    1  c04
..........................guuguguggugacucguaug.....................................................................  1    0  c04
..........................guuguguggugacucguaugc....................................................................  1    0  c04
...........................uuguguggugacucguau......................................................................  3    0  c04
...........................uuguguggugacucguaugc....................................................................  2    0  c04
............................uguguggugacucguaugc....................................................................  1    0  c04
.................................................uaucaagucaucaucccgacu.............................................  1    0  c04
.................................................uaucaagucaucaucccgacuuaua.........................................  1    0  c04
...................................................ucaagucaucaucccgacuuauaucgu.....................................  1    0  c04
...................................................ucaagucaucaucccgacuuauaucguu....................................  1    0  c04
..........................................................................ucguuauucccgaccccaacaugca................  1    0  c04
..........................................................................ucguuauucccgaccccaacaugcau...............  3    0  c04
..........................................................................Gcguuauucccgaccccaacaugcaug..............  1    1  c04
..........................................................................ucguuauucccgaccccaacaugcauA..............  1    1  c04
..........................................................................ucguuauucccgaccccaacaugcaug..............  1    0  c04
..........................................................................ucguuauucccgaccccaacaugcaugC.............  1    1  c04
...............................................................................auucccgacAccaacaugcau...............  1    1  c04

....................ugacaaguuguguggugacucgu........................................................................  2    0  c16
....................ugacaaguuguguggugacucgua.......................................................................  3    0  c16
....................ugacaaguuguguggugacAcguau......................................................................  1    1  c16
....................ugacaaguuguguggugacucguau......................................................................  15   0  c16
....................ugacaaguuguguggugacucguaugc....................................................................  4    0  c16
..........................................uaugcguuaucaagucaucauccc.................................................  1    0  c16
........................................................ucaucaucccgacuuauaucguuauucc...............................  1    0  c16
```

Provisional ID	:	group0_301
Score total	:	133.4
Score for star read(s)	:	3.9
Score for read counts	:	132.2
Score for mfe	:	-3.3
Score for randfold	:	-2.2
Score for cons. seed	:	3
Total read count	:	271
Mature read count	:	168
Loop read count	:	0
Star read count	:	103

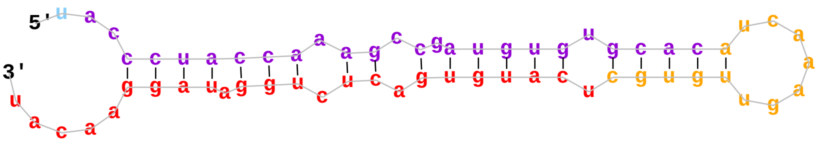

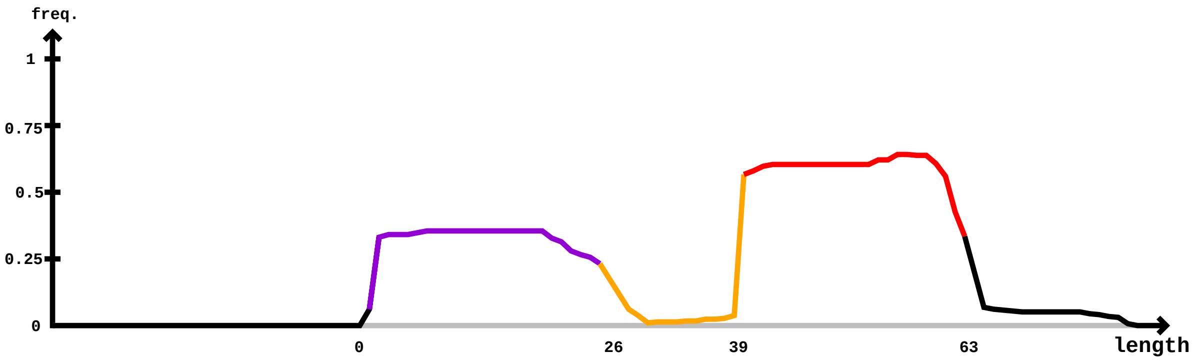

Star **Mature**

```
5'- uucauuuguuauuauuuuuacauauuaaauuuacccuaccaaagccgaugugugcacaucaaaguugugcucaugugacucuggauaggaacauuaucacacucuguguaagac   -3'   obs
    uucauuuguuauuauuuuuacauauuaaauuuacccuaccaaagccgaugugugcacaucaaaguugugcucaugugacucuggauaggaacauuaucacacucuguguaagac         exp
    ...............(((((((((..........(((((((.((.(.(((((.(((((.......))))).)))))).)).))).))))...............))))))))).   reads   mm   sample
    ...............................uacccuaccaaagccgaug................................................................   1   0   c09
    ...............................uacccuaccaaagccgaugugu.............................................................   1   0   c09
    ...............................uacccuaccaaagccgaugugugcac.........................................................   1   0   c09
    ................................Ccccuaccaaagccgaug................................................................   1   1   c09
    ................................Ccccuaccaaagccgaugu...............................................................   1   1   c09
    ................................acccuaccaaagccgaugu...............................................................   1   0   c09
    ................................acccuaccaaagccgaugug..............................................................   1   0   c09
    ................................acccuacAaaagccgaugug..............................................................   1   1   c09
    ................................acccuaccaaagccgaugugugca..........................................................   1   0   c09
    ................................Ccccuaccaaagccgaugugugcac.........................................................   1   1   c09
    ................................acccuaccaaagccgaugugugcac.........................................................   5   0   c09
    ................................acccuaccaaagccgaugugugcaca........................................................   2   0   c09
    ................................Ccccuaccaaagccgaugugugcaca........................................................   1   1   c09
    ................................acccuaccaaagccgaugugugcacauc......................................................   1   0   c09
    ................................acccuaccaaagccgaugugugcacaucaaaguugugcu...........................................   1   0   c09
    .................................cccuaccaaagccgaugugugcaca........................................................   1   0   c09
    .............................................................aaguugugcucaugugacucuggauaggaac.......................   1   0   c09
    ......................................................................ucaugugacucuggauagga........................   3   0   c09
    ......................................................................Gcaugugacucuggauagga........................   1   1   c09
    ......................................................................Gcaugugacucuggauaggaa.......................   1   1   c09
    ......................................................................ucaugugacucuggauaggaa.......................   3   0   c09
    ......................................................................ucaugugacucuggauaggaac......................   9   0   c09
    ......................................................................ucaugugacucuggauaggaaca.....................   6   0   c09
    ......................................................................ucaugugacucuggauaggaacau....................   13  0   c09
    ......................................................................ucaugugacucuggauaggaacauu...................   1   0   c09
    ......................................................................ucaugugacucuggauaggaacauuC..................   1   1   c09
    .......................................................................caugugacucuggauaggaac......................   1   0   c09
    .......................................................................caugugacucuggauaggaacauu...................   2   0   c09
    ........................................................................augugacucuggauaggaaca.....................   1   0   c09
    ........................................................................augugacucuggauaggaacauuaucacacucugugua....   1   0   c09
    .........................................................................ugugacucuggauaggaacauuaucacacucugugua....   1   0   c09
    ....................................................................................auaggaacauuaucacacucug........   1   0   c09
    ....................................................................................auaggaacauuaucacacucugu.......   1   0   c09
```

**Star** / **Mature**			
uucauuuguuauuauuuuuacauauuaaauuuacccuaccaaagccgaugugugcacaucaaaguugugcucaugugacucuggauaggaacauuaucacacucuguguaagac			
....aggaacauuaucacacucugug......	1	0	c09
....aggaacauuaucacacucugugua....	1	0	c09
...............................uacccuaccaaagccgaugugugc...	2	0	c16
...............................uacccuaccaaagccgaugugugca..	1	0	c16
...............................uacccuaccaaagccgaugugugcac...	1	0	c16
................................Ccccuaccaaagccgaug..	1	1	c16
................................acccuaccaaagccgaug..	1	0	c16
................................acccuaccaaagccgaugug..	3	0	c16
................................Ccccuaccaaagccgaugug..	2	1	c16
................................Ccccuaccaaagccgaugugug..	1	1	c16
................................acccuaccaaagccgaugugugca..	2	0	c16
................................acccuaccaaagccgaugugugcac...	2	0	c16
................................Ccccuaccaaagccgaugugugcac...	4	1	c16
................................acccuaccaaagccgaugugugcaca..	1	0	c16
................................acccuaccaaagccgaugugugcacau...	1	0	c16
....................................uaccaaagccgaugugugcac...	2	0	c16
.....................................accaaagccgaugugugca..	1	0	c16
....aaaguugugcucaugugacucuggauaggaac......................	1	0	c16
....ucaugugacucuggauag..........................	1	0	c16
....ucaugugacucuggauagga........................	1	0	c16
....ucaugugacucuggauaggaa.......................	2	0	c16
....ucaugugacucuggauaggaac......................	4	0	c16
....ucaugugCcucuggauaggaac......................	1	1	c16
....ucaugugacucuggauaggaaca.....................	3	0	c16
....ucaugugacucuggauaggaacau....................	9	0	c16
....Gcaugugacucuggauaggaacau....................	1	1	c16
....caugugacucuggauaggaacau....................	2	0	c16
....auaggaacauuaucacacucuguguaa...	1	0	c16
...............................uacccuaccaaagccgaugug..	1	0	c04
...............................uacccuaccaaagccgaugugugcac...	1	0	c04
...............................uacccuaccaaagccgaugugugcaca..	2	0	c04
................................Ccccuaccaaagccgaug..	1	1	c04
................................acccuaccaaagccgaug..	1	0	c04
................................acccuaccaaagccgaugu...	1	0	c04
................................Ccccuaccaaagccgaugu...	1	1	c04
................................Ccccuaccaaagccgaugug..	1	1	c04
................................acccuaccaaagccgauguC..	1	1	c04
................................Ccccuaccaaagccgaugugugc...	2	1	c04
................................Ccccuaccaaagccgaugugugcac...	1	1	c04
................................acccuaccaaagccgaugugugcac...	5	0	c04
.................................cccuaccaaagccgaugugu...	1	0	c04
....uugugcucaugugacucugga.............................	1	0	c04
....ucaugugacucuggauagga........................	1	0	c04
....ucaugugacucuggauaggaac......................	1	0	c04
....ucaugugacucuggauaggaacau....................	2	0	c04
....ucaugugacucuggauaggaacaA....................	2	1	c04
....Uuaggaacauuaucacacucug........	1	1	c04
................................Ccccuaccaaagccgaugugugca..	1	1	c02
................................Ccccuaccaaagccgaugugugcaca..	1	1	c02
.....................................accaaagccgaugugugcac...	1	0	c02
....caucaaaguugugcucaugugacucuggauaggaac......................	1	0	c02
....gugcucaugugacucuggauaggaacauuau.................	1	0	c02
....cucaugugacucuggauaggaac......................	1	0	c02
....ucaugugacucuggauagga........................	2	0	c02
....Gcaugugacucuggauaggaa.......................	1	1	c02
....ucaugugacucuggauaggaa.......................	3	0	c02
....ucaugugacucuggauaggaac......................	5	0	c02
....ucaugugacucuggauaggaaca.....................	3	0	c02
....ucaugugacucuggauaggaacau....................	13	0	c02
....ugugacucuggauaggaacauuaucacacucugugua....	1	0	c02
....uaggaacauuaucacacucugugua....	1	0	c02
...............................uacccuaccaaagccgaugugu...	2	0	c15
...............................uacccuaccaaagccgaugugugc...	1	0	c15
...............................uacccuaccaaagccgaugugugca..	1	0	c15
...............................uacccuaccaaagccgaugugugcaca..	3	0	c15
................................acccuaccaaagccgaug..	1	0	c15

Star Mature

```
uucauuuguuauuauuuuuacauauuaaauuuacccuaccaaagccgaugugugcacaucaaaguugugcucaugugacucuggauaggaacauuaucacacucuguguaagac
................................acccuaccaaagccgauA................................................................ 1 1 c15
................................acccuaccaaagccgaugugug............................................................ 2 0 c15
................................acccuaccaaagccgaugugugc........................................................... 2 0 c15
................................acccuaccaaagccgaugugugca.......................................................... 2 0 c15
................................acccuaccaaagccgaugugugcaca........................................................ 7 0 c15
................................acccuaccaaagccgaugugugcacau....................................................... 5 0 c15
................................acccuaccaaagccgaugugugcacauc...................................................... 7 0 c15
................................acccuaccaaagccgaugugugcacauU...................................................... 1 1 c15
.................................cccuaccaaagccgaugugugcacau....................................................... 1 0 c15
..................................................................gugcucaugugacucuggauaggaac...................... 1 0 c15
....................................................................gcucaugugacucuggauaggaac...................... 1 0 c15
.....................................................................cucaugugacucuggauaggaac...................... 1 0 c15
.....................................................................cucaugugacucuggauaggaacau.................... 1 0 c15
......................................................................ucaugugacucuggauagga........................ 1 0 c15
......................................................................ucaugugacucuggauaggaa....................... 4 0 c15
......................................................................ucaugugacucuggauaggaac...................... 10 0 c15
......................................................................ucaugugacucuggauaggaaA...................... 1 1 c15
......................................................................ucaugugacucuggauaggaaca..................... 14 0 c15
......................................................................uGaugugacucuggauaggaacau.................... 1 1 c15
......................................................................ucaugugacucuggauaggaacau.................... 30 0 c15
......................................................................ucaugugacucuggauaggaacaA.................... 1 1 c15
........................................................................augugacucuggauaggaacauua.................. 1 0 c15
........................................................................augugacucuggauaggaacauuauc................ 1 0 c15
........................................................................augugacucuggauaggaacauuauca............... 1 0 c15
....................................................................................auaggaacauuaucacacucuguguaa... 1 0 c15
......................................................................................aggaacauuaucacacucugug...... 1 0 c15
......................................................................................aggaacauuaucacacucugugu..... 1 0 c15
......................................................................................aggaacauuaucacacucugugua.... 2 0 c15
```

Provisional ID	:	group0_326
Score total	:	1091.3
Score for star read(s)	:	3.9
Score for read counts	:	1087.6
Score for mfe	:	-1
Score for randfold	:	1.6
Score for cons. seed	:	-0.6
Total read count	:	2145
Mature read count	:	2096
Loop read count	:	1
Star read count	:	48

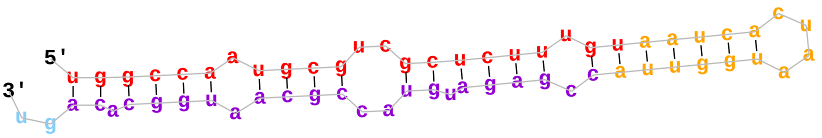

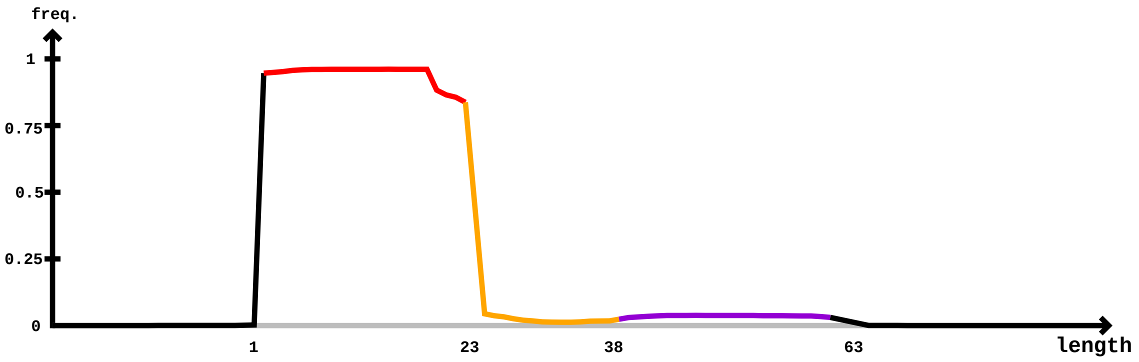

Mature **Star**

```
5'- auaacuagaugcauaaacuauggccaaugcgucgcucuuuguaaucacuaaugguuaccgagauguaccgcaauggcacagucuauuacucaaaccuacuuguaacuu -3'  obs
    auaacuagaugcauaaacuauggccaaugcgucgcucuuuguaaucacuaaugguuaccgagauguaccgcaauggcacagucuauuacucaaaccuacuuguaacuu       exp
    .....((((((..........((((((.((((..((((((.(((((((....))))))).)))).))..)))).)))).)))))))......................... reads  mm  sample
    ....................uggccaaugcgucgcucuuugu..................................................................  15  0  c15
    ....................uAgccaaugcgucgcucuuugu..................................................................  1  1  c15
    ....................Cggccaaugcgucgcucuuugu..................................................................  1  1  c15
    ....................uggccaaugcgucgcucuuugua.................................................................  2  0  c15
    ....................uggccaaugcgucgcucuuuguaa................................................................  1  0  c15
    ....................uggccaaugcgucgcucuuuguaaucac............................................................  1  0  c15
    ....................uggccaaugcgucgcucuuuguaaucacu...........................................................  2  0  c15
    .....................ggccaaugcgucgcucuuugu..................................................................  1  0  c15
    .....................ggccaaugcgucgcucuuugua.................................................................  1  0  c15
    .....................ggccaaugcgucgcucuuuguaaucacC...........................................................  1  1  c15
    .........................aaugcgucgcucuuuguaauc..............................................................  1  0  c15
    .........................aaugcgucgcucuuuguaaucacu...........................................................  1  0  c15
    ...........................ugcgucgcucuuuguaaucacu...........................................................  1  0  c15
    ..........................................aaucacuaaugguuaccgagC.............................................  1  1  c15
    ..................................................augguuaccgagauguaccgcaauggcacagu..........................  1  0  c15
    .....................................................guuaccgagauguaccgcaauggcacag...........................  1  0  c15
    ..........................................................cgagauguaccgcaauggcacaA...........................  1  1  c15
    ..........................................................cgagauguaccgcaauggcacagu..........................  1  0  c15

    ....................Gggccaaugcgucgcucu......................................................................  7  1  c02
    ....................uggccaaugcgucgcucC......................................................................  1  1  c02
    ....................uggccaaugcgucgcucu......................................................................  46  0  c02
    ....................uggccaaugAgucgcucu......................................................................  1  1  c02
    ....................uggccaaugcgucgcucuu.....................................................................  5  0  c02
    ....................uggccaUugcgucgcucuuu....................................................................  1  1  c02
    ....................uggccaaugcgucgcucuuu....................................................................  3  0  c02
    ....................uggccaaugcgucgcucuuug...................................................................  7  0  c02
    ....................Gggccaaugcgucgcucuuug...................................................................  1  1  c02
    ....................uggccaauUcgucgcucuuugu..................................................................  1  1  c02
    ....................ugCccaaugcgucgcucuuugu..................................................................  3  1  c02
    ....................Gggccaaugcgucgcucuuugu..................................................................  38  1  c02
    ....................uggccaaugcgucgAucuuugu..................................................................  1  1  c02
    ....................uggccaaugcgucgcGcuuugu..................................................................  1  1  c02
```

```
                    Mature                               Star
auaacuagaugcauaaacuauggccaaugcgucgcucuuuguaaucacuaaugguuaccgagauguaccgcaauggcacagucuauuacucaaaccuacuuguaacuu
....................Aggccaaugcgucgcucuuugu..................................................................   1     1   c02
....................uggccaaugcgucgcucuuugu..................................................................   504   0   c02
....................uggccaaugcgucCcucuuugu..................................................................   1     1   c02
....................uUgccaaugcgucgcucuuugu..................................................................   1     1   c02
....................uggccaaugcgucAcucuuugu..................................................................   1     1   c02
....................Gggccaaugcgucgcucuuugua.................................................................   2     1   c02
....................uggccaaugcgucgcucuuuguU.................................................................   3     1   c02
....................uggccaaugcgucgcucuuugua.................................................................   25    0   c02
....................uggccaaugcgucgcucuuuguaa................................................................   2     0   c02
....................uggccaaugcgucgcucuuuguaU................................................................   1     1   c02
....................uggccaaugcgucgcucuuuguaau...............................................................   1     0   c02
....................uggccaaugAgucgcucuuuguaau...............................................................   1     1   c02
....................Gggccaaugcgucgcucuuuguaauc..............................................................   1     1   c02
....................uggccaaugcgucgcucuuuguaauc..............................................................   3     0   c02
....................uggccaaugcgucgcucuuuguaauca.............................................................   2     0   c02
....................uggccaaugcgucgcucuuuguaaucac............................................................   3     0   c02
....................uggccaaugcgucgcucuuuguaaucacu...........................................................   4     0   c02
....................uggccaaugcgucgcucuuuguaaucacA...........................................................   1     1   c02
....................uggccaaugcgucgcucuuuguaaucacua..........................................................   1     0   c02
....................uggccaaugcgucgcucuuuguaaucacuaa.........................................................   1     0   c02
.....................ggccaaugcgucgcucuuugu..................................................................   1     0   c02
.....................ggccaaugcgucgcucuuugua.................................................................   1     0   c02
......................gccaaugcgucgcucuuugu..................................................................   3     0   c02
.......................ccaaugcgucgcucuuugu..................................................................   4     0   c02
.........................aaugcgucgcucuuuguaaucacua..........................................................   1     0   c02
...............................................cuaaugguuaccgaCauguaccgcaauggcacaguc.........................   1     1   c02
.................................................aaugguuaccgagauguaccgcaauggcacag...........................   1     0   c02
.................................................aaugguuaccgagauguaccgcaauggcacagu..........................   3     0   c02
.................................................aaugguuaccgagauguacAgcaauggcacagu..........................   1     1   c02
.................................................Caugguuaccgagauguaccgcaauggcacagu..........................   1     1   c02
..................................................augguuaccgagauguaccgcaauggcacaguc.........................   1     0   c02
.......................................................uaccgagauguaccgcaauggcacagu..........................   1     0   c02
.......................................................uaccgagauguaccgcaauggcacagucuauu.....................   1     0   c02
.........................................................ccgagauguaccgcaaugg................................   1     0   c02
.........................................................ccgagauguaccgcaauggca..............................   2     0   c02
.........................................................ccgagauguaccgcaauggcac.............................   1     0   c02
..........................................................cgagauguaccgcaauggcac.............................   1     0   c02
..........................................................cgagauguaccgcaauggcacag...........................   2     0   c02
..........................................................cgagauguaccgcaauggcacagu..........................   2     0   c02
..........................................................cgagauguaccgcaauggcacaguU.........................   1     1   c02
...........................................................gagauguaccgcaauggcacagu..........................   1     0   c02
...........................................................gagauguaccgcaauggcacaguc.........................   3     0   c02
............................................................agauguaccgcaauggcacagu..........................   1     0   c02
............................................................agauguaccgcaauggcacaguc.........................   1     0   c02
..............................................................auguaccgcaauggcacaguc.........................   1     0   c02

.........ugcauaaacuauggccaaugcgucg..........................................................................   1     0   c09
..................uUggccaaugcgucgcucuuugu...................................................................   1     1   c09
....................Gggccaaugcgucgcucu......................................................................   6     1   c09
....................uggccaaugcgucgcucu......................................................................   81    0   c09
....................uggcAaaugcgucgcucu......................................................................   1     1   c09
....................uggccaaugcgucgcucuC.....................................................................   1     1   c09
....................uggccaaugcgucgcucuu.....................................................................   24    0   c09
....................uggccaaugcgucgcucGu.....................................................................   1     1   c09
....................Gggccaaugcgucgcucuu.....................................................................   1     1   c09
....................uggccaaugcgucgcucuuu....................................................................   9     0   c09
....................Gggccaaugcgucgcucuuu....................................................................   1     1   c09
....................uggccaauCcgucgcucuuug...................................................................   1     1   c09
....................uggccaaugcgucgcucuuuA...................................................................   1     1   c09
....................uggccaaugcgucgcucuuug...................................................................   20    0   c09
....................uggUcaaugcgucgcucuuugu..................................................................   1     1   c09
....................uggccaaugcgucgcucCuugu..................................................................   1     1   c09
....................uggccaaugcCucgcucuuugu..................................................................   1     1   c09
....................uggccaaugAgucgcucuuugu..................................................................   2     1   c09
....................uCgccaaugcgucgcucuuugu..................................................................   1     1   c09
....................uggAcaaugcgucgcucuuugu..................................................................   1     1   c09
....................uggccaaCgcgucgcucuuugu..................................................................   1     1   c09
....................uggccaaugcgucgcuUuuugu..................................................................   1     1   c09
....................uggccaaugUgucgcucuuugu..................................................................   1     1   c09
....................uggccaaugcgucgAucuuugu..................................................................   1     1   c09
```

```
                    Mature                                Star
auaacuagaugcauaaacuauggccaaugcgucgcucuuuguaaucacuaaugguuaccgagauguaccgcaauggcacagucuauuacucaaaccuacuuguaacuu
....................uggcAaaugcgucgcucuuugu..................................................................  1    1  c09
....................uggccaaugcgucgcucuuugu..................................................................  614  0  c09
....................ugCccaaugcgucgcucuuugu..................................................................  2    1  c09
....................Gggccaaugcgucgcucuuugu..................................................................  24   1  c09
....................uggccUaugcgucgcucuuugu..................................................................  2    1  c09
....................uggccaaugcgucgcucuuugua.................................................................  18   0  c09
....................uggccaaugcgucgcucuuuguG.................................................................  2    1  c09
....................Gggccaaugcgucgcucuuugua.................................................................  2    1  c09
....................uggccaaugcgucgcucuuuguU.................................................................  3    1  c09
....................uggccaaugcgucgcucuuuguaU................................................................  2    1  c09
....................uggccaaugcgucgcucuuuguaa................................................................  1    0  c09
....................uggccaaugcgucgcucuuuguaau...............................................................  1    0  c09
....................uggccaaugcgucgcucuuuguaauc..............................................................  9    0  c09
....................uggccaaugcgucgcucuuuguaauG..............................................................  1    1  c09
....................uggccaaugcgucgcucuuuguaaCca.............................................................  1    1  c09
....................uggccaaugcgucgcucuuuguaauca.............................................................  4    0  c09
....................Gggccaaugcgucgcucuuuguaaucac............................................................  1    1  c09
....................uggccaaugcgucgcucuuuguaaucac............................................................  1    0  c09
....................Gggccaaugcgucgcucuuuguaaucacu...........................................................  1    1  c09
....................uggccaaugcgucgcucuuuguaaucacu...........................................................  6    0  c09
....................uggccaaugcgucgcucuuuguaaucacua..........................................................  2    0  c09
....................uggccaaugcgucgcucuuAguaaucacua..........................................................  1    1  c09
....................uggccaaugcgucgcucuuuguaaucacuaauggu.....................................................  1    0  c09
.....................ggccaaugcgucgcucuuugua.................................................................  1    0  c09
......................gccaaugcgucgcucuuugu..................................................................  3    0  c09
......................gccaaugcgucgcucuuuguaaucacu...........................................................  1    0  c09
.......................ccaaugcgucgcucuuugu..................................................................  4    0  c09
........................caaugcgucgcucuuugu..................................................................  4    0  c09
.................................gcucuuuguaaucacuaaug.......................................................  1    0  c09
..............................................acuaaugguuaccgagauguaccgca....................................  1    0  c09
..............................................acuaaugguuaccgagauguaccgcaauggcacagu..........................  1    0  c09
................................................uaaugguuaccgagaugu..........................................  1    0  c09
.................................................aaugguuaccgagauguaccgcaauggcacagu..........................  1    0  c09
.................................................aaugguuaccgagauguaccgcaauggcacaguc.........................  2    0  c09
.................................................aaugguuaccgagauguaccgcaauggcacaguA.........................  1    1  c09
..................................................augguuaccgagauguaccgcaauggcacagu..........................  1    0  c09
.....................................................guuaccgagauguaccgcaauggcaca............................  1    0  c09
.....................................................guuaccgagauguaccgcaauggcacaguA.........................  1    1  c09
......................................................uuaccgagauguaccgca....................................  1    0  c09
......................................................uuaccgagauguaccgcaauggca..............................  1    0  c09
......................................................uuaccgagauguaccgcaauggcacag...........................  1    0  c09
......................................................uuaccgagauguaccgcaauggcacaguA.........................  1    1  c09
......................................................uuaccgagauguaccgcaauggcacaguc.........................  1    0  c09
.........................................................ccgagauguaccgcaaug.................................  1    0  c09
.........................................................ccgagauguaccgcaauggcac.............................  3    0  c09
.........................................................ccgagauguaccgcaauggcacU............................  1    1  c09
.........................................................ccgagauguaccgcaauggcaca............................  2    0  c09
.........................................................ccgagauguaccgcaauggcacagu..........................  1    0  c09
..........................................................cgagauguaccgcaauggcac.............................  1    0  c09
..........................................................cgagauguaccgcaauggcacagu..........................  1    0  c09
..........................................................cgagauguaccgcaauggcacagA..........................  1    1  c09
...........................................................gagauguaccgcaauggcacaguc.........................  1    0  c09
............................................................agauguaccgcaauggcacaguc.........................  1    0  c09
.............................................................gauguaccgcaauggcacaguc.........................  2    0  c09
.............................................................gauguaccgcaauggcacaguA.........................  1    1  c09

..................uauggccaaugcgucgcucuuugu..................................................................  1    0  c04
...................auggccaaugcgucgcucuuuguaaucacu...........................................................  1    0  c04
....................uggccaaugcgucgcucu......................................................................  2    0  c04
....................Gggccaaugcgucgcucuuugu..................................................................  1    1  c04
....................uggccaaugcgucgcucuuugu..................................................................  22   0  c04
....................uggccaaugcgucgcucCuugu..................................................................  1    1  c04
....................uggccaaugcgucgcucuuuguU.................................................................  1    1  c04
....................uggccaaugcgucgcucuuuguaaA...............................................................  2    1  c04
....................uggccaaugcgucgcucuuuguaauc..............................................................  1    0  c04
....................uggccaaugcgucgcucuuuguaauca.............................................................  1    0  c04
....................uggccaaugcgucgcucuuuguaaucacuU..........................................................  1    1  c04
.......................ccaaugcgucgcucuuugu..................................................................  1    0  c04
............................................ucacuaaugguuaccgagauguaccgcaauggcacagu..........................  1    0  c04
.................................................aaugguuaccgagauguaccgcaauggcacaguc.........................  1    0  c04
```

Mature Star

```
auaacuagaugcauaaacuauggccaaugcgucgcucuuuguaaucacuaaugguuaccgagauguaccgcaauggcacagucuauuacucaaaccuacuuguaacuu
..................................................augguuaccgagauguaccgcaauggcacagu..........................	1	0	c04
..........................................................cgagauguaccgcaauggcacaguc.........................	1	0	c04
............................................................agauguaccgcaauggcacaguc.........................	1	0	c04
...............................................................uguaccgcaauggcacaguc.........................	1	0	c04

...................auggccaaugcgucgcucuuugu..................................................................	1	0	c16
....................Gggccaaugcgucgcucu......................................................................	2	1	c16
....................uggccaaugcgucgcucu......................................................................	26	0	c16
....................uggccaaugcgucgcucuu.....................................................................	7	0	c16
....................uggccaaugcgucgcucuC.....................................................................	1	1	c16
....................uggccaaugcgucgcucuuu....................................................................	5	0	c16
....................Gggccaaugcgucgcucuuu....................................................................	1	1	c16
....................uggccaaugcgucgcucuuug...................................................................	10	0	c16
....................ugUccaaugcgucgcucuuug...................................................................	1	1	c16
....................Gggccaaugcgucgcucuuug...................................................................	1	1	c16
....................uggAcaaugcgucgcucuuugu..................................................................	3	1	c16
....................uggccaaAgcgucgcucuuugu..................................................................	1	1	c16
....................uggccCaugcgucgcucuuugu..................................................................	1	1	c16
....................uggccaaugcgucgcuAuuugu..................................................................	1	1	c16
....................Aggccaaugcgucgcucuuugu..................................................................	1	1	c16
....................uggccaaugcgucgcucGuugu..................................................................	1	1	c16
....................uUgccaaugcgucgcucuuugu..................................................................	1	1	c16
....................uggccaaugcgucgcucuuugu..................................................................	373	0	c16
....................Gggccaaugcgucgcucuuugu..................................................................	22	1	c16
....................ugUccaaugcgucgcucuuugu..................................................................	1	1	c16
....................uggccaaugcgucgcucuuugua.................................................................	19	0	c16
....................uggccaaugcgucgcucuuugAa.................................................................	1	1	c16
....................uggccaaugcgucgcucuuuguC.................................................................	1	1	c16
....................ugCccaaugcgucgcucuuugua.................................................................	1	1	c16
....................Gggccaaugcgucgcucuuugua.................................................................	2	1	c16
....................uggccaaugcgucgcucuuuguU.................................................................	1	1	c16
....................uggccaaugcgucgcucuuuguaa................................................................	9	0	c16
....................Gggccaaugcgucgcucuuuguaa................................................................	1	1	c16
....................uggccaaugcgucgcucuuuguaau...............................................................	1	0	c16
....................uggccaaugAgucgcucuuuguaau...............................................................	1	1	c16
....................uggccaaugcgucgcucuuuguaaA...............................................................	2	1	c16
....................uggccaaugcgucgcucuuuguaauc..............................................................	2	0	c16
....................uggccaaugcgucgcucuuuguaauca.............................................................	5	0	c16
....................uggccaaugcgucgcucuuuguaaucac............................................................	1	0	c16
....................Gggccaaugcgucgcucuuuguaaucacu...........................................................	1	1	c16
....................uggccaaugcgucgcucuuuguaaucacu...........................................................	4	0	c16
.......................ccaaugcgucgcucuuugu..................................................................	1	0	c16
........................Uaaugcgucgcucuuugu..................................................................	1	1	c16
.................................................aaugguuaccgagauguaccgcaauggcacag...........................	3	0	c16
.................................................aaugguuaccgagauguaccgcaauggcacagu..........................	1	0	c16
.................................................Caugguuaccgagauguaccgcaauggcacaguc.........................	1	1	c16
.....................................................guuaccgagauguaccgcaauggcacaguc.........................	1	0	c16
......................................................uuaccgagauguaccgcaauggca..............................	1	0	c16
........................................................accgagauguaccgcaauggcacU............................	1	1	c16
.........................................................ccgagauguaccgcaauggcaca............................	2	0	c16
..........................................................cgagauguaccgcaauggca..............................	1	0	c16
..........................................................cgagauguaccgcaauggcac.............................	1	0	c16
............................................................agauguaccgcaauggcacaguc.........................	1	0	c16
.............................................................gauguaccgcaauggcacaguc.........................	1	0	c16
..............................................................auguaccgcaauggcacagu..........................	1	0	c16
..............................................................auguaccgcaauggcacaguc.........................	1	0	c16
.................................................................uaccgcaauggcacaguc.........................	1	0	c16
```

Provisional ID	:	group0_440
Score total	:	6462.3
Score for star read(s)	:	3.9
Score for read counts	:	6452.5
Score for mfe	:	1.3
Score for randfold	:	1.6
Score for cons. seed	:	3
Total read count	:	12668
Mature read count	:	12446
Loop read count	:	1
Star read count	:	221

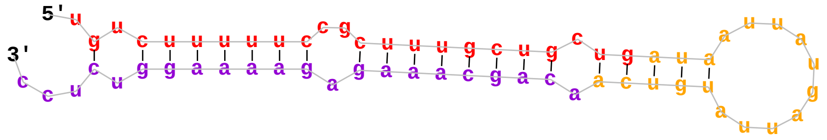

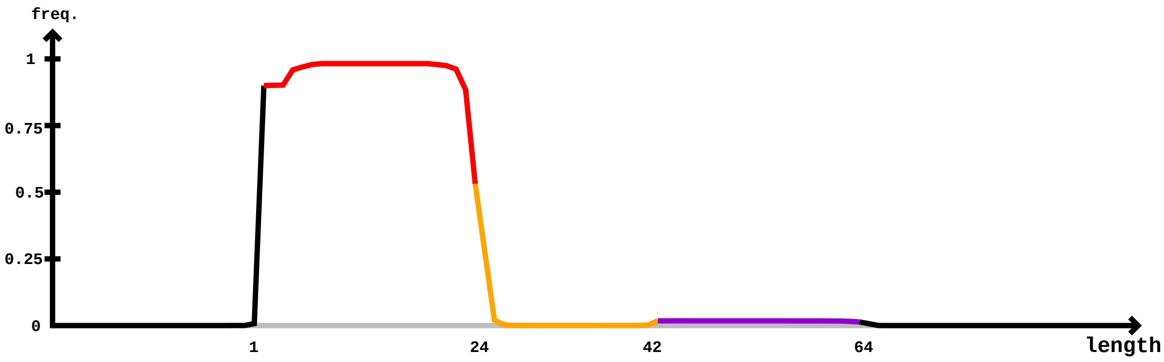


```
                     Mature                                    Star
5'- gcgaaaguucuccauaaauuugucuuuuuccgcuuugcugcugauaauuaugauuaugucaacagcaaagagaaaaggucuccuuuaugagaacuuccuccguggcaacga  -3'   obs
    gcgaaaguucuccauaaauuugucuuuuuccgcuuugcugcugauaauuaugauuaugucaacagcaaagagaaaaggucuccuuuaugagaacuuccuccguggcaacga         exp
    ....(((((((.(((((...(.(((((((..((((((((.(((((.........))))).))))))))).))))))).)...))))))))))))....(((....))).     reads    mm    sample
    .................auuugucuuuuuccgcuuugcugcug....................................................................     1        0     c02
    ...................uugucuuuuuccgcuuugc.........................................................................     1        0     c02
    ...................uugucuuuuuccgcuuugcu........................................................................     4        0     c02
    ...................uugucuuuuuccgcuuugcug.......................................................................     5        0     c02
    ...................Gugucuuuuuccgcuuugcugc......................................................................     1        1     c02
    ...................uugucuuuuuccgcuuugcugc......................................................................     2        0     c02
    ...................Augucuuuuuccgcuuugcugc......................................................................     1        1     c02
    ...................uugucuuuuuccgcuuugcugcu.....................................................................     9        0     c02
    ...................uGgucuuuuuccgcuuugcugcu.....................................................................     2        1     c02
    ...................Gugucuuuuuccgcuuugcugcu.....................................................................     2        1     c02
    ...................uugucuuuuuccgcuuugcugcuU....................................................................     1        1     c02
    ...................uugucuuuuuccgcuuugcugcug....................................................................     4        0     c02
    ....................ugucuuuuuccgcuuugc.........................................................................     7        0     c02
    ....................ugucuuuuuccgcuuugcu........................................................................     5        0     c02
    ....................Ggucuuuuuccgcuuugcug.......................................................................     4        1     c02
    ....................ugucuuuuuccgcuuugcug.......................................................................     39       0     c02
    ....................ugucuuuuuccgcuuugcugU......................................................................     5        1     c02
    ....................uCucuuuuuccgcuuugcugc......................................................................     1        1     c02
    ....................Ggucuuuuuccgcuuugcugc......................................................................     7        1     c02
    ....................ugucuuuuuccgcuuugcCgc......................................................................     1        1     c02
    ....................ugucuAuuuccgcuuugcugc......................................................................     1        1     c02
    ....................ugucuuuuuccgcuuugcugc......................................................................     92       0     c02
    ....................ugucuuuuuccgcGuugcugc......................................................................     1        1     c02
    ....................Agucuuuuuccgcuuugcugcu.....................................................................     4        1     c02
    ....................ugucuuuuuccUcuuugcugcu.....................................................................     1        1     c02
    ....................ugucuuuuuAcgcuuugcugcu.....................................................................     2        1     c02
    ....................ugucuuuuuccgcuuugcAgcu.....................................................................     1        1     c02
    ....................ugucuuuuuccgcuuugcCgcu.....................................................................     2        1     c02
    ....................ugucuuuuuccgcuuugcugcA.....................................................................     5        1     c02
    ....................ugucCuuuuccgcuuugcugcu.....................................................................     7        1     c02
    ....................ugucuuuuuccgcGuugcugcu.....................................................................     1        1     c02
    ....................ugucuuuuuccgcuuAgcugcu.....................................................................     1        1     c02
    ....................ugucuuuuAccgcuuugcugcu.....................................................................     1        1     c02
```

Mature Star

gcgaaaguucuccauaaauuugucuuuuuccgcuuugcugcugauaauuaugauuaugucaacagcaaagagaaaaggucuccuuuaugagaacuuccuccguggcaacga			
....................uAucuuuuuccgcuuugcugcu...	1	1	c02
....................uguAuuuuuccgcuuugcugcu...	1	1	c02
....................ugucuuuuuccgcuuuUcugcu...	1	1	c02
....................ugucuuuuucGgcuuugcugcu...	1	1	c02
....................ugucuuuuuccgcuuugcuCcu...	2	1	c02
....................ugucuuuuuGcgcuuugcugcu...	1	1	c02
....................Cgucuuuuuccgcuuugcugcu...	1	1	c02
....................uCucuuuuuccgcuuugcugcu...	2	1	c02
....................ugucuuuuuccgcuuuCcugcu...	1	1	c02
....................Ggucuuuuuccgcuuugcugcu...	93	1	c02
....................ugucuuuuucUgcuuugcugcu...	1	1	c02
....................ugucuuuuuccCcuuugcugcu...	1	1	c02
....................ugucuuuuuUcgcuuugcugcu...	1	1	c02
....................ugucuuuuuccgcuuugUugcu...	1	1	c02
....................ugucuuuuuccgcuuugcugcu...	898	0	c02
....................uguUuuuuuccgcuuugcugcu...	1	1	c02
....................uUucuuuuuccgcuuugcugcu...	1	1	c02
....................ugucuuuuuccgAuuugcugcug..	1	1	c02
....................uCucuuuuuccgcuuugcugcug..	1	1	c02
....................ugucuuuuuccgcuuugcugcug..	579	0	c02
....................Cgucuuuuuccgcuuugcugcug..	1	1	c02
....................ugucuuuuuccgcuuugcugcuA..	6	1	c02
....................ugucuuuuuAcgcuuugcugcug..	1	1	c02
....................ugucuuuuCccgcuuugcugcug..	1	1	c02
....................ugucuuuuuccgcuuugcCgcug..	1	1	c02
....................Ggucuuuuuccgcuuugcugcug..	64	1	c02
....................ugucCuuuuccgcuuugcugcug..	2	1	c02
....................ugucuuuuuccgcuuugUugcug..	1	1	c02
....................ugucuuuuuccgcuuugcugcAg..	1	1	c02
....................ugucuuuuuccgcuuugcugcuC..	3	1	c02
....................ugucuuuuAccgcuuugcugcug..	1	1	c02
....................ugucuGuuuccgcuuugcugcug..	1	1	c02
....................ugucuuuuuccgcuuuAcugcug..	1	1	c02
....................ugucuuuuuccgUuuugcugcug..	4	1	c02
....................ugucuuuuuccgcuuugcugcuU..	12	1	c02
....................Agucuuuuuccgcuuugcugcug..	1	1	c02
....................ugucuuuuucUgcuuugcugcug..	1	1	c02
....................uguAuuuuuccgcuuugcugcug..	1	1	c02
....................Ggucuuuuuccgcuuugcugcuga...	1	1	c02
....................ugucuuuuuccgcuuugcugcugU...	72	1	c02
....................ugucuuuuuccgcuuugcugcugG...	1	1	c02
....................ugucuuuuuccgcuuugcugcuga...	14	0	c02
....................ugucuuuuuccgcuuugcugcugUu..	2	1	c02
....................ugucuuuuuccgcuuugcugcugau..	1	0	c02
....................ugucuuuuuccgcuuugcugcugaA..	2	1	c02
....................ugucuuuuuccgcuuugcugcugCu..	2	1	c02
.....................gucuuuuuccgcuuugcugc..	1	0	c02
.....................gucuuuuuccgcuuugcugcu...	1	0	c02
.....................gucuuuuuccgcuuugcugcug..	2	0	c02
.....................gucuuuuuccgcuuugcugcugU...	1	1	c02
.......................cuuuuuAcgcuuugcugc..	1	1	c02
.......................cuuuuuccgcuuugcugU..	1	1	c02
.......................cuuuuuccgcuuugcugc..	16	0	c02
.......................cuuuuuccgcGuugcugc..	1	1	c02
.......................cuuuuuccgUuuugcugcu...	1	1	c02
.......................cuuuuuccgcuuugcugcA...	3	1	c02
.......................cuuuAuccgcuuugcugcu...	1	1	c02
.......................cuuuuuccgGuuugcugcu...	1	1	c02
.......................cuuuuuccgcuuugcugcu...	144	0	c02
.......................cuuuuuccgcuuugcugcug..	103	0	c02
.......................Guuuuuccgcuuugcugcug..	1	1	c02
.......................cuuuuuccgcuuugcugcuU..	4	1	c02
.......................cuuuuuccgcuuuCcugcug..	1	1	c02
.......................cuuuuucUgcuuugcugcug..	1	1	c02
.......................cuuuuuccgcuuugcuCcug..	1	1	c02
.......................cuuuuuccgcGuugcugcug..	1	1	c02
.......................cuuuuuccgcuuugcugcuga...	7	0	c02
.......................cuuuuuccgcuuugcugcugC...	2	1	c02
.......................cuuuuuccgcuuugcugcugU...	10	1	c02
.......................cuuuuuccgcuuugcugcugaG..	1	1	c02

```
                    Mature                                  Star
gcgaaaguucuccauaaauuugucuuuuuccgcuuugcugcugauaauuaugauuaugucaacagcaaagagaaaaggucuccuuuaugagaacuuccuccguggcaacga
........................uuuuuccgcuuugcugcu.....................................................................    23    0    c02
........................Guuuuccgcuuugcugcu.....................................................................    3     1    c02
........................uuuuuccgcuuugcugcuU....................................................................    1     1    c02
........................uuuuuccgcuuugcugcug....................................................................    7     0    c02
........................uuuuuccgcuuugcugcuCa...................................................................    1     1    c02
........................uuuuuccgcuuugcugcugU...................................................................    2     1    c02
.........................uuuuUcgcuuugcugcug....................................................................    1     1    c02
.........................uuuuccgcuuugcugcuU....................................................................    1     1    c02
.........................Guuuccgcuuugcugcug....................................................................    5     1    c02
.........................uuuuccgcuuugcugcug....................................................................    24    0    c02
.........................uuuuccgcuuugcugcugU...................................................................    2     1    c02
.........................uuuuccgcuuugcugcuga...................................................................    1     0    c02
.........................uuuuccgcuuugcugcugau..................................................................    1     0    c02
..........................uuuccgcuuugcugcugU...................................................................    6     1    c02
..........................uuuccgcuuugcugcugCu..................................................................    1     1    c02
..........................................................ucaacagcaaagagaaaaggucu..............................    1     0    c02
............................................................Cacagcaaagagaaaaggucuc.............................    1     1    c02
............................................................aacagcaaagagaaaaggucucc............................    1     0    c02
.............................................................acagcaaagagaaaaggucu..............................    3     0    c02
.............................................................Ccagcaaagagaaaaggucuc.............................    2     1    c02
.............................................................acagcaaagagaaaaggucuc.............................    4     0    c02
.............................................................Ccagcaaagagaaaaggucucc............................    6     1    c02
.............................................................acagcaaagagaaaaggucucc............................    17    0    c02
.............................................................acagcaaagagaaaaggucuccu...........................    2     0    c02
.............................................................acagcaaagagaaaaggucuccA...........................    1     1    c02

...................uugucuuuuuccgcuuugcug.......................................................................    4     0    c15
...................Cugucuuuuuccgcuuugcug.......................................................................    1     1    c15
...................uugucuuuuuccgcuuugcugc......................................................................    2     0    c15
...................uugucuuuuuccgcuuugcugU......................................................................    2     1    c15
...................uugucuuuuuccgcuuugcugcu.....................................................................    13    0    c15
...................uugucuuuuuccgcuuugcugcuU....................................................................    2     1    c15
...................uugucuuuuuccgcuuugcugcug....................................................................    3     0    c15
...................uugucuuuuuccgcuuugcugcugU...................................................................    3     1    c15
...................uugucuuuuuccgcuuugcugcugUu..................................................................    1     1    c15
....................ugucuuuuuccgcuuugc.........................................................................    6     0    c15
....................ugucuuuuuccgcuuugcu........................................................................    6     0    c15
....................ugucuuuuuccgGuuugcug.......................................................................    1     1    c15
....................uUucuuuuuccgcuuugcug.......................................................................    1     1    c15
....................ugucuuuuuccgcuuugcuA.......................................................................    6     1    c15
....................ugucuuuuuccgcuuugcug.......................................................................    60    0    c15
....................ugucuuuGuccgcuuugcug.......................................................................    1     1    c15
....................Cgucuuuuuccgcuuugcugc......................................................................    1     1    c15
....................ugucuuuuuccgcuuCgcugc......................................................................    1     1    c15
....................ugucuuuuuccgcuuugcugU......................................................................    25    1    c15
....................ugucuuuuucAgcuuugcugc......................................................................    1     1    c15
....................ugucuuuuuccgcuuuUcugc......................................................................    1     1    c15
....................ugucuuuuucGgcuuugcugc......................................................................    1     1    c15
....................ugucuuuuuccgcuuugcugA......................................................................    3     1    c15
....................ugucuuuuuccgcuuugcugc......................................................................    520   0    c15
....................ugucuuuuucUgcuuugcugc......................................................................    1     1    c15
....................Ggucuuuuuccgcuuugcugc......................................................................    2     1    c15
....................ugucuuuuuUcgcuuugcugc......................................................................    1     1    c15
....................ugucuuuuuccgUuuugcugc......................................................................    1     1    c15
....................ugucuuuuuccgcuuugcuAc......................................................................    9     1    c15
....................ugucuuuuuccgcuuCgcugcu.....................................................................    1     1    c15
....................ugucuuuGuccgcuuugcugcu.....................................................................    1     1    c15
....................ugucuuuuuAcgcuuugcugcu.....................................................................    1     1    c15
....................ugucuuuuuccgcuuugcugAu.....................................................................    2     1    c15
....................ugucuuuuuccgcuuugUugcu.....................................................................    1     1    c15
....................ugucuuuuuccgcuCugcugcu.....................................................................    1     1    c15
....................ugucuuuuuccgcuuugcugcA.....................................................................    49    1    c15
....................ugucuuuuuccgcuAugcugcu.....................................................................    1     1    c15
....................uCucuuuuuccgcuuugcugcu.....................................................................    1     1    c15
....................uAucuuuuuccgcuuugcugcu.....................................................................    2     1    c15
....................ugucuuuuuccgcuuugcuAcu.....................................................................    1     1    c15
....................ugucuuuuucUgcuuugcugcu.....................................................................    2     1    c15
....................ugucuuuuuccgcuuugcugcu.....................................................................    1620  0    c15
....................ugucCuuuuccgcuuugcugcu.....................................................................    1     1    c15
....................ugucuuuuuccgUuuugcugcu.....................................................................    1     1    c15
```

```
                    Mature                                    Star
gcgaaaguucuccauaaauuugucuuuuuccgcuuugcugcugauaauuaugauuaugucaacagcaaagagaaaaggucuccuuuaugagaacuuccuccguggcaacga
....................ugCcuuuuuccgcuuugcugcu.....................................................................    1      1    c15
....................ugucuuuuuccgcuuugcugcC.....................................................................    20     1    c15
....................ugucuuuuuccgcuuugcugUu.....................................................................    4      1    c15
....................ugucuuuuuccgcuuugcugcG.....................................................................    8      1    c15
....................ugucuAuuuccgcuuugcugcu.....................................................................    1      1    c15
....................ugucuuuuuccgcuuugcCgcu.....................................................................    1      1    c15
....................ugucuuuuuccgcuuugcugcuA....................................................................    125    1    c15
....................Cgucuuuuuccgcuuugcugcug....................................................................    4      1    c15
....................ugucuuuuuUcgcuuugcugcug....................................................................    3      1    c15
....................ugucuuuuuccgcuuCgcugcug....................................................................    2      1    c15
....................ugucuuuuuccgcuuugUugcug....................................................................    1      1    c15
....................ugucuuuuuccgcuuGgcugcug....................................................................    1      1    c15
....................ugucuuuuuccgcuuugcugAug....................................................................    1      1    c15
....................uUucuuuuuccgcuuugcugcug....................................................................    1      1    c15
....................ugucuuuuucUgcuuugcugcug....................................................................    2      1    c15
....................ugucuuuuuccgcuuugcuAcug....................................................................    1      1    c15
....................uguUuuuuuccgcuuugcugcug....................................................................    1      1    c15
....................ugucuuuuuccgcuuugcugcuC....................................................................    13     1    c15
....................ugAcuuuuuccgcuuugcugcug....................................................................    2      1    c15
....................ugucuuuuuccgcuuugcugcGg....................................................................    1      1    c15
....................Agucuuuuuccgcuuugcugcug....................................................................    2      1    c15
....................ugucuuuuuccUcuuugcugcug....................................................................    1      1    c15
....................ugucuuuuuccgcuuugcCgcug....................................................................    1      1    c15
....................ugucuuuuuccgUuuugcugcug....................................................................    2      1    c15
....................ugucuuuuuccgGuuugcugcug....................................................................    2      1    c15
....................ugucuuuuuccgcCuugcugcug....................................................................    1      1    c15
....................ugucuuuuuccgcAuugcugcug....................................................................    1      1    c15
....................ugucuuuuuccgcuuugcugcug....................................................................    2139   0    c15
....................ugucuuuuuccgcuuuAcugcug....................................................................    3      1    c15
....................ugucuuuuuccgcuuugcugcCg....................................................................    1      1    c15
....................ugucuuuuuccgcuuugcugcAg....................................................................    2      1    c15
....................ugucuCuuuccgcuuugcugcug....................................................................    1      1    c15
....................ugucuuuuuccgcuuugcugcuU....................................................................    116    1    c15
....................uAucuuuuuccgcuuugcugcug....................................................................    3      1    c15
....................ugucCuuuuccgcuuugcugcuga...................................................................    1      1    c15
....................Agucuuuuuccgcuuugcugcuga...................................................................    1      1    c15
....................ugucuuuuuccgcuuugcugcugU...................................................................    449    1    c15
....................ugucuuuuuccgcuuugcugcuAa...................................................................    3      1    c15
....................ugucuuuuuccgcuuugcugcuga...................................................................    395    0    c15
....................ugucuuuuuccgcuuugcugUuga...................................................................    1      1    c15
....................ugucuuuuuccgcuuugcugcugC...................................................................    9      1    c15
....................ugucuuuuuccgcuuugcugcuUa...................................................................    1      1    c15
....................ugucuuuuuccgcuuugcugcugaC..................................................................    4      1    c15
....................ugucuuuuuccgcuuugcugcugaA..................................................................    21     1    c15
....................ugucuuuuuccgcuuugcugcugau..................................................................    90     0    c15
....................ugucuuuuuccgcuuugcugcugCu..................................................................    3      1    c15
....................ugucuuuuuccgcuuugcugcugUu..................................................................    56     1    c15
....................ugucuuuuuccgcuuugcugcuUau..................................................................    1      1    c15
....................ugucuuuuuccgcuuugcugcugauU.................................................................    25     1    c15
....................ugucuuuuuccgcuuugcugcugauC.................................................................    4      1    c15
....................ugucuuuuuccgcuuugcugcugUua.................................................................    4      1    c15
....................ugucuuuuuccgcuuugcugcugUuaa................................................................    1      1    c15
....................ugucuuuuuccgcuuugcugcugauaU................................................................    5      1    c15
....................ugucuuuuuccgcuuugcugcugauUau...............................................................    1      1    c15
.....................gucuuuuuccgcuuugcugcug....................................................................    3      0    c15
.....................gucuuuuuccgcuuugcugcuga...................................................................    2      0    c15
......................ucuuuuuccgcuuugcugcug....................................................................    1      0    c15
......................ucuuuuuccgcuuugcugcugau..................................................................    2      0    c15
.......................cuuuuuccgcuuugcugcu.....................................................................    1      0    c15
........................uuuuuccgcuuugcugcug....................................................................    1      0    c15
........................uuuuuccgcuuugcugcugU...................................................................    1      1    c15
.........................uuuuccgcuuugcugcuA....................................................................    1      1    c15
............................................uaauuaugauuaugucaacag..............................................    1      0    c15
.......................................................augucaacagcaaagagaaa....................................    1      0    c15
............................................................aacagcaaagagaaaaggucu..............................    1      0    c15
............................................................aacagcaaagagaaaaggucuc.............................    2      0    c15
............................................................aacagcaaagagaaaaggucucc............................    8      0    c15
............................................................aacagcaaagagaaaaggucuccu...........................    1      0    c15
.............................................................acagcaaagagaaaaggucuc.............................    6      0    c15
.............................................................acagcaaagagaaaaggGcucc............................    2      1    c15
```

```
                    Mature                                      Star
gcgaaaguucuccauaaauuugucuuuuuccgcuuugcugcugauaauuaugauuaugucaacagcaaagagaaaaggucuccuuuaugagaacuuccuccguggcaacga
.............................................................Ucagcaaagagaaaaggucucc............................   2     1   c15
.............................................................acagcaaagagaaaaggCcucc............................   1     1   c15
.............................................................acagcaaagagaaaaggucucc............................   74    0   c15
.............................................................acagcaaagagaaaaggucucA............................   1     1   c15
.............................................................acagcaaagagaaaaggucuccu...........................   4     0   c15
.............................................................acagcaaagagaaaaggucuccA...........................   3     1   c15
.............................................................acagcaaagagaaaaggucuccuA..........................   1     1   c15

.................Cuuugucuuuuuccgcuuugcugcu.....................................................................   1     1   c09
..................uCugucuuuuuccgcuuugcugcu.....................................................................   1     1   c09
...................uugucuuuuuccgcuuugcug.......................................................................   3     0   c09
...................Gugucuuuuuccgcuuugcug.......................................................................   1     1   c09
...................uugucuuuuuccgcuuugcugc......................................................................   2     0   c09
...................uugucuuuuuccgcuuugcugcu.....................................................................   6     0   c09
...................Gugucuuuuuccgcuuugcugcu.....................................................................   1     1   c09
...................Cugucuuuuuccgcuuugcugcu.....................................................................   1     1   c09
...................uugucuuuuuccgcuuugcugcuU....................................................................   2     1   c09
...................uugucuuuuuccgcuuugcugcug....................................................................   1     0   c09
....................ugucuuuuuccgcuuugc.........................................................................   18    0   c09
....................Agucuuuuuccgcuuugc.........................................................................   1     1   c09
....................Ggucuuuuuccgcuuugc.........................................................................   1     1   c09
....................ugucuuuuuccgcuuugcu........................................................................   20    0   c09
....................Ggucuuuuuccgcuuugcu........................................................................   3     1   c09
....................ugucuuuuuccgcuuugcug.......................................................................   18    0   c09
....................Ggucuuuuuccgcuuugcugc......................................................................   8     1   c09
....................ugucCuuuuccgcuuugcugc......................................................................   1     1   c09
....................ugucuuuuuccgcuuugcCgc......................................................................   1     1   c09
....................uguUuuuuuccgcuuugcugc......................................................................   1     1   c09
....................ugucuuuuuccgcuuugcAgc......................................................................   1     1   c09
....................ugucuuuuuccgcuuugcugU......................................................................   1     1   c09
....................ugucuuuuuccgcuuugcugc......................................................................   117   0   c09
....................ugucuuuuucUgcuuugcugcu.....................................................................   1     1   c09
....................ugucuuuuuccgcuuugcugcC.....................................................................   1     1   c09
....................ugucuuuuuccgcuuugcugAu.....................................................................   1     1   c09
....................Agucuuuuuccgcuuugcugcu.....................................................................   2     1   c09
....................ugucuuuuuccgcGuugcugcu.....................................................................   1     1   c09
....................ugucuuuCuccgcuuugcugcu.....................................................................   1     1   c09
....................ugucuuuuuccgcAuugcugcu.....................................................................   1     1   c09
....................ugucCuuuuccgcuuugcugcu.....................................................................   6     1   c09
....................ugAcuuuuuccgcuuugcugcu.....................................................................   1     1   c09
....................ugucuuuuuccgcuuugcugcA.....................................................................   12    1   c09
....................ugucuuuuuccgcuuugcugcu.....................................................................   700   0   c09
....................ugucuuuuuUcgcuuugcugcu.....................................................................   2     1   c09
....................ugucuuuuuccgcuuugcuCcu.....................................................................   1     1   c09
....................Cgucuuuuuccgcuuugcugcu.....................................................................   1     1   c09
....................ugucuuuuuccgcuuugcugUu.....................................................................   1     1   c09
....................ugucuuuuucAgcuuugcugcu.....................................................................   2     1   c09
....................ugucuuuuuAcgcuuugcugcu.....................................................................   1     1   c09
....................ugucuuuuuccgcuuugAugcu.....................................................................   1     1   c09
....................uguAuuuuuccgcuuugcugcu.....................................................................   2     1   c09
....................Ggucuuuuuccgcuuugcugcu.....................................................................   48    1   c09
....................ugucuuuuuccgcCuugcugcu.....................................................................   1     1   c09
....................ugucuuuuuccgGuuugcugcug....................................................................   1     1   c09
....................ugucuuuuuccgcuuugcugcug....................................................................   920   0   c09
....................ugucuuuuuccgAuuugcugcug....................................................................   1     1   c09
....................uUucuuuuuccgcuuugcugcug....................................................................   1     1   c09
....................uguAuuuuuccgcuuugcugcug....................................................................   1     1   c09
....................ugucuuuuuccgcuuugcugcuC....................................................................   6     1   c09
....................ugucuuAuuccgcuuugcugcug....................................................................   1     1   c09
....................ugucuuuuuccgcuuugcCgcug....................................................................   1     1   c09
....................ugucuuuuuccgcuuugAugcug....................................................................   2     1   c09
....................ugucuuuuucGgcuuugcugcug....................................................................   1     1   c09
....................ugucuuuuuccgcuuuUcugcug....................................................................   2     1   c09
....................Agucuuuuuccgcuuugcugcug....................................................................   1     1   c09
....................ugucuuuuucAgcuuugcugcug....................................................................   1     1   c09
....................ugucuuuuuccgcuuugcugcuU....................................................................   10    1   c09
....................ugucuuuuuccgcCuugcugcug....................................................................   1     1   c09
....................ugucuuuuuUcgcuuugcugcug....................................................................   1     1   c09
....................ugCcuuuuuccgcuuugcugcug....................................................................   1     1   c09
....................Ggucuuuuuccgcuuugcugcug....................................................................   71    1   c09
```

```
                    Mature                                       Star
gcgaaaguucuccauaaauuugucuuuuuccgcuuugcugcugauaauuaugauuaugucaacagcaaagagaaaaggucuccuuuaugagaacuuccuccguggcaacga
....................uCucuuuuuccgcuuugcugcug....................................................................    1    1    c09
....................ugucuuuuuccgcuuugUugcug....................................................................    1    1    c09
....................ugucuuuuuccgcuuuCcugcug....................................................................    1    1    c09
....................ugucuuuuuccUcuuugcugcug....................................................................    1    1    c09
....................ugucuuuuuccgcuuugcugcuA....................................................................    1    1    c09
....................ugucCuuuuccgcuuugcugcug....................................................................    3    1    c09
....................ugucuuuuuccgcuuugcugcugC...................................................................    2    1    c09
....................ugucuuuuuccgcuuugcugcuga...................................................................    25   0    c09
....................ugucuuuuuccgcuuugcugcugG...................................................................    1    1    c09
....................Ggucuuuuuccgcuuugcugcuga...................................................................    5    1    c09
....................ugucuuuuuccgcuuugcugcugU...................................................................    63   1    c09
....................ugucCuuuuccgcuuugcugcuga...................................................................    1    1    c09
....................ugucuuuuuccgcuuugcugcugUu..................................................................    9    1    c09
....................ugucuuuuuccgcuuugcugcugau..................................................................    2    0    c09
....................ugucuuuuuccgcuuugcugcugGu..................................................................    2    1    c09
......................ucuuuuuccgcuuugcugcu.....................................................................    2    0    c09
.......................cuuuuuccgcuuugcugc......................................................................    25   0    c09
.......................cuuuuuccgcuuugcuCcu.....................................................................    1    1    c09
.......................cuuuuuccgcuuugcugUu.....................................................................    1    1    c09
.......................cuuuuuUcgcuuugcugcu.....................................................................    1    1    c09
.......................cuuuuuccgcuuugcugcu.....................................................................    121  0    c09
.......................cuuuuuccgcuuugcuUcu.....................................................................    1    1    c09
.......................cuuuuuAcgcuuugcugcu.....................................................................    1    1    c09
.......................cuuuuucAgcuuugcugcug....................................................................    2    1    c09
.......................cuuuuuccgcuuuUcugcug....................................................................    1    1    c09
.......................cuuuuuccgcuuugcugcuU....................................................................    3    1    c09
.......................cuuuuuccgcuuugcugcGg....................................................................    1    1    c09
.......................cuuuuuccgGuuugcugcug....................................................................    1    1    c09
.......................cuuuuuccgcuuugcugcug....................................................................    110  0    c09
.......................cCuuuuccgcuuugcugcug....................................................................    1    1    c09
.......................cuuuuuccgUuuugcugcug....................................................................    1    1    c09
.......................cuuuuuccgcuuugcugcuA....................................................................    1    1    c09
.......................cuuuuuccgcuuugUugcug....................................................................    1    1    c09
.......................cuuuuuccgcuuugcugcuAa...................................................................    1    1    c09
.......................cuuuuuccgcuuugcugcugU...................................................................    8    1    c09
.......................cuuuuuccgcuuugcugcuga...................................................................    10   0    c09
.......................cuuuuuccgcuuugcugcugUu..................................................................    1    1    c09
.......................cuuuuuccgcuuugcugcugau..................................................................    1    0    c09
........................Guuuuccgcuuugcugcu.....................................................................    1    1    c09
........................uuuuuccgcuuuCcugcu.....................................................................    1    1    c09
........................uuuuuccgcuuugcugcu.....................................................................    26   0    c09
........................Auuuuccgcuuugcugcug....................................................................    1    1    c09
........................uuuuuccgcuuugcugcug....................................................................    31   0    c09
........................uuuuuccgUuuugcugcug....................................................................    1    1    c09
........................Guuuuccgcuuugcugcug....................................................................    7    1    c09
........................uuuuuccgcuuugcugcuga...................................................................    1    0    c09
........................Guuuuccgcuuugcugcuga...................................................................    1    1    c09
........................uuuuuccgcuuugcugcugU...................................................................    4    1    c09
.........................uuuuccgcuuugcugcuU....................................................................    1    1    c09
.........................Guuuccgcuuugcugcug....................................................................    4    1    c09
.........................uuuuccgcuGugcugcug....................................................................    1    1    c09
.........................uuuuccgcuuuCcugcug....................................................................    1    1    c09
.........................uuuuccgcuuugcugcuA....................................................................    3    1    c09
.........................uuuuccgcuuAgcugcug....................................................................    1    1    c09
.........................uuuuccgcuuugcugcug....................................................................    43   0    c09
.........................uuuuccgcuuugcugcugU...................................................................    4    1    c09
.........................uuuuccgcuuugcugcuga...................................................................    2    0    c09
.........................uuuuccgcuuugcugcugaA..................................................................    1    1    c09
..........................uuuccgcuuugcugcuga...................................................................    11   0    c09
..........................uuuccgcuuugcugcuAa...................................................................    1    1    c09
..........................Guuccgcuuugcugcuga...................................................................    1    1    c09
..........................uuuccgcuuugcugcugG...................................................................    1    1    c09
..........................uuuccgcuuugcugcugU...................................................................    7    1    c09
..........................Guuccgcuuugcugcugau..................................................................    1    1    c09
..........................uuuccgcuuugcugcugUu..................................................................    1    1    c09
............................................................aacagcaaagagaaaaggu................................    3    0    c09
............................................................Cacagcaaagagaaaaggucu..............................    1    1    c09
............................................................aacagcaaagagaaaaggucuc.............................    3    0    c09
............................................................aacagcaaagagaaaaggucucc............................    2    0    c09
............................................................aacagcaaagagaaaaggucucU............................    1    1    c09
```

```
                   Mature                                 Star
gcgaaaguucuccauaaauuugucuuuuuccgcuuugcugcugauaauuaugauuaugucaacagcaaagagaaaaggucuccuuuaugagaacuuccuccguggcaacga
.............................................................acagcaaagagaaaaggucu..............................   6      0      c09
.............................................................acagcaaagagaaaGggucu..............................   1      1      c09
.............................................................acagcaaaCagaaaaggucu..............................   1      1      c09
.............................................................acagcaaagagaaaaggucuc.............................   4      0      c09
.............................................................acagcaaagagaaaaggucucc............................   15     0      c09
.............................................................Ccagcaaagagaaaaggucucc............................   2      1      c09
.............................................................acagcaaagagaaaaggucuccu...........................   2      0      c09
.............................................................acagcaaagagaaaaggucuccA...........................   1      1      c09

..................Cuugucuuuuuccgcuuugcugcu.....................................................................   1      1      c04
...................uugucuuuuuccgcuuugcug.......................................................................   1      0      c04
...................uugucuuuuuccgcuuugcugc......................................................................   1      0      c04
...................uugucuuuuuccgcuuugcugcu.....................................................................   1      0      c04
...................uugucuuuuuccgcuuugcugcuU....................................................................   1      1      c04
....................ugucuuuuuccgcuuugc.........................................................................   3      0      c04
....................Ggucuuuuuccgcuuugc.........................................................................   1      1      c04
....................ugucuuuuuccUcuuugc.........................................................................   1      1      c04
....................Ggucuuuuuccgcuuugcu........................................................................   1      1      c04
....................ugucuuuuuccgcuuugcu........................................................................   9      0      c04
....................ugucuuuuuccgcuuugcug.......................................................................   4      0      c04
....................ugucuuuuuccgcuuugcugc......................................................................   42     0      c04
....................Ggucuuuuuccgcuuugcugc......................................................................   3      1      c04
....................ugucuuuuuccgcuuuCcugc......................................................................   1      1      c04
....................Ggucuuuuuccgcuuugcugcu.....................................................................   12     1      c04
....................ugucuuuuuccgcuuugcugcu.....................................................................   165    0      c04
....................ugucuuuuuccgcuuugcugcA.....................................................................   4      1      c04
....................ugucuuuuuccgcuCugcugcu.....................................................................   1      1      c04
....................ugucAuuuuccgcuuugcugcu.....................................................................   1      1      c04
....................ugucCuuuuccgcuuugcugcu.....................................................................   2      1      c04
....................ugucuuuuuccgcuuugcugcuU....................................................................   8      1      c04
....................ugucuuuuuccgcuuugcugcug....................................................................   149    0      c04
....................Agucuuuuuccgcuuugcugcug....................................................................   1      1      c04
....................ugucCuuuuccgcuuugcugcug....................................................................   1      1      c04
....................ugucuuuuuccgcCuugcugcug....................................................................   1      1      c04
....................Ggucuuuuuccgcuuugcugcug....................................................................   11     1      c04
....................ugucuuuuuccgcuuugcuUcug....................................................................   1      1      c04
....................uguUuuuuuccgcuuugcugcug....................................................................   1      1      c04
....................ugucuuuuucAgcuuugcugcug....................................................................   1      1      c04
....................ugucuuuuuccgcuuugcugcugG...................................................................   2      1      c04
....................ugucuuuuuccgcuuugcugcugU...................................................................   18     1      c04
....................ugucuuuuuccgcuuugcugcuga...................................................................   5      0      c04
....................Ggucuuuuuccgcuuugcugcuga...................................................................   2      1      c04
....................ugucuuuuuccgcuuugcugcugUu..................................................................   2      1      c04
.....................gucuuuuuccgcuuugcugcuga...................................................................   1      0      c04
......................uAuuuuuccgcuuugcugcu.....................................................................   1      1      c04
......................ucuuuuuccgcuuugcugcu.....................................................................   2      0      c04
.......................cuuuuuccgcuuugcugc......................................................................   5      0      c04
.......................cuuuuuccgcuuugcugcA.....................................................................   1      1      c04
.......................cuuuuuccgcuuugcugcu.....................................................................   17     0      c04
.......................cuuuuuccgcuCugcugcug....................................................................   1      1      c04
.......................cuuuuuccgcuuugcugcug....................................................................   22     0      c04
.......................cuuuuuccgcuuugcugcuU....................................................................   2      1      c04
.......................cuuuuuccgcuuugcugcuga...................................................................   1      0      c04
.......................cuuuuuccgcuuugcugcugau..................................................................   1      0      c04
........................uuuuuccgcuuugcugcu.....................................................................   4      0      c04
........................Guuuuccgcuuugcugcu.....................................................................   1      1      c04
........................Guuuuccgcuuugcugcug....................................................................   1      1      c04
........................Cuuuuccgcuuugcugcug....................................................................   1      1      c04
........................uuuuuccgcuuugcugcuU....................................................................   1      1      c04
........................uuuuuccgcuuugcugcugU...................................................................   1      1      c04
.........................uuuuccgcuuugcugcug....................................................................   10     0      c04
.........................uuuuccgcuuugcugcugU...................................................................   2      1      c04
..........................uuuccgcuuugcugcuAa...................................................................   1      1      c04
..........................uuuccgcuuugcugcugU...................................................................   4      1      c04
..........................uuuccgcuuugcugcuga...................................................................   1      0      c04
..........................uuuccgcuuugcugcugaA..................................................................   2      1      c04
...........................................................Uaacagcaaagagaaaaggucuccu...........................   1      1      c04
............................................................aacagcaaagagaaaaggucuc.............................   1      0      c04
............................................................aacagcaaagagaaaaggucucc............................   1      0      c04
.............................................................acagcaaagagaaaagguc...............................   1      0      c04
```

Mature Star

```
gcgaaaguucuccauaaauuugucuuuuuccgcuuugcugcugauaaauuaugauuaugucaacagcaaagagaaaaggucuccuuuaugagaacuuccuccguggcaacga
..............................................................acagcaaagagaaaaggucu..............................	1	0	c04
..............................................................acagcaaagagaaaaggucuc.............................	2	0	c04
..............................................................Ccagcaaagagaaaaggucucc............................	2	1	c04
..............................................................aAagcaaagagaaaaggucucc............................	1	1	c04
..............................................................acagcaaagagaaaaggucucc............................	6	0	c04
..............................................................acagcaaagagaaaaggucuccA...........................	1	1	c04
..............................................................Ccagcaaagagaaaaggucuccu...........................	1	1	c04

...................uugucuuuuuccgcuuugcug........................................................................	1	0	c16
...................Gugucuuuuuccgcuuugcugcu......................................................................	1	1	c16
...................uugucuuuuuccgcuuugcugcu......................................................................	2	0	c16
....................Cgucuuuuuccgcuuugc..........................................................................	1	1	c16
....................ugucuuuuuccgcuuugc..........................................................................	4	0	c16
....................ugucuuuuuccgcuuugcu.........................................................................	5	0	c16
....................ugucuuuuuccgcuuugcug........................................................................	12	0	c16
....................ugucuuuuuccgcuuugcugc.......................................................................	61	0	c16
....................Ggucuuuuuccgcuuugcugc.......................................................................	5	1	c16
....................ugucuuuuuccgcuuugcugU.......................................................................	1	1	c16
....................ugucCuuuuccgcuuugcugc.......................................................................	1	1	c16
....................ugucuuuuuccgcuuugcugAu......................................................................	1	1	c16
....................ugucuuuuuAcgcuuugcugcu......................................................................	2	1	c16
....................ugucuuuuuccgcuuugcugGu......................................................................	1	1	c16
....................ugucAuuuuccgcuuugcugcu......................................................................	1	1	c16
....................ugucuuuuuccgcuuCgcugcu......................................................................	1	1	c16
....................ugucuuuuuccgcuuugcugcA......................................................................	6	1	c16
....................ugucCuuuuccgcuuugcugcu......................................................................	1	1	c16
....................ugucuuuuucAgcuuugcugcu......................................................................	2	1	c16
....................ugucuuuuuccgcuuugcugcu......................................................................	271	0	c16
....................ugucuuuuuccCcuuugcugcu......................................................................	1	1	c16
....................ugucuuuuuccgcuuugAugcu......................................................................	1	1	c16
....................Ggucuuuuuccgcuuugcugcu......................................................................	21	1	c16
....................Ggucuuuuuccgcuuugcugcug.....................................................................	33	1	c16
....................Agucuuuuuccgcuuugcugcug.....................................................................	1	1	c16
....................ugucuuuuuccgAuuugcugcug.....................................................................	1	1	c16
....................ugucuuuuuccgGuuugcugcug.....................................................................	1	1	c16
....................ugucuuuuuccgcuuuUcugcug.....................................................................	1	1	c16
....................ugucuuuuuccgcuuugcugcug.....................................................................	363	0	c16
....................ugucuuuuuccgUuuugcugcug.....................................................................	1	1	c16
....................uCucuuuuuccgcuuugcugcug.....................................................................	1	1	c16
....................ugucCuuuuccgcuuugcugcug.....................................................................	2	1	c16
....................ugucuuuuuccgcuuugcugcuC.....................................................................	1	1	c16
....................ugucuuuuuccgcuuugcugAug.....................................................................	1	1	c16
....................uguUuuuuuccgcuuugcugcug.....................................................................	1	1	c16
....................uguAuuuuuccgcuuugcugcug.....................................................................	1	1	c16
....................ugucuuuuuccgcuuugcugcuA.....................................................................	3	1	c16
....................ugucuuuuuccgcuuugcugcuU.....................................................................	8	1	c16
....................ugucuuuuuccgcuuugcugcugC....................................................................	1	1	c16
....................ugucuuuuuccgcuuugcugcuga....................................................................	14	0	c16
....................Ggucuuuuuccgcuuugcugcuga....................................................................	2	1	c16
....................ugucuuuuuccgcuuugcugcugU....................................................................	49	1	c16
....................ugucuuuuuccgcuuugcugcugUu...................................................................	4	1	c16
....................ugucuuuuuccgcuuugcugcugaA...................................................................	1	1	c16
.....................gucuuuuuccgcuuugcugcu......................................................................	1	0	c16
.....................gucuuuuuccgcuuugcugcugU....................................................................	1	1	c16
.......................cuuuuuccgcuuugcugc.......................................................................	2	0	c16
.......................cuuuuuccgcuuugcugcu......................................................................	23	0	c16
.......................cuuuuuccgcuuugAugcu......................................................................	1	1	c16
.......................cuuuuuccgcuuugcugcA......................................................................	1	1	c16
.......................cuuuuuccgcuuugcugcuU.....................................................................	2	1	c16
.......................cuuuuuccgcuuugcugcug.....................................................................	39	0	c16
.......................cuuuuuccgcuAugcugcug.....................................................................	1	1	c16
.......................cAuuuuccgcuuugcugcug.....................................................................	1	1	c16
.......................cuuuuuccCcuuugcugcug.....................................................................	1	1	c16
.......................cuuuuuccgcuuugcugcuga....................................................................	1	0	c16
.......................cuuuuuccgcuuugcugcugU....................................................................	5	1	c16
........................Guuuuccgcuuugcugcu......................................................................	1	1	c16
........................uuuuuccgcuuugcugcu......................................................................	7	0	c16
........................uuuuuccgcuuugcugcug.....................................................................	5	0	c16
........................uuuuuccgcuuugcugcuU.....................................................................	1	1	c16
........................Guuuuccgcuuugcugcug.....................................................................	1	1	c16
```

```
                    Mature                                  Star
gcgaaaguucuccauaaaauuugucuuuuuccgcuuugcugcugauaauuaugauuaugucaacagcaaagagaaaaggucuccuuuaugagaacuuccuccguggcaacga
.........................Cuuuuccgcuuugcugcug....................................................................    1    1    c16
..........................uuuuccgcuuugcugcug....................................................................    6    0    c16
..........................uuuuccgcuuugcugcugU...................................................................    2    1    c16
...........................uuuccgcuuugcugcugU...................................................................    3    1    c16
...........................uuuccgcuuugcugcuga...................................................................    4    0    c16
..............................................................acagcaaagagaaaaggu................................    1    0    c16
..............................................................acagcaaagagaaaagguc...............................    1    0    c16
..............................................................acagcaaagagaaaaggucu..............................    1    0    c16
..............................................................acagcaaagagaaaaggucuc.............................    2    0    c16
..............................................................Ccagcaaagagaaaaggucuc.............................    1    1    c16
..............................................................acagcaaagagaaaaggucucc............................    10   0    c16
..............................................................Ccagcaaagagaaaaggucucc............................    2    1    c16
```

Provisional ID	:	group0_47
Score total	:	198.5
Score for star read(s)	:	3.9
Score for read counts	:	196.4
Score for mfe	:	-2.7
Score for randfold	:	1.6
Score for cons. seed	:	-0.6
Total read count	:	397
Mature read count	:	380
Loop read count	:	0
Star read count	:	17

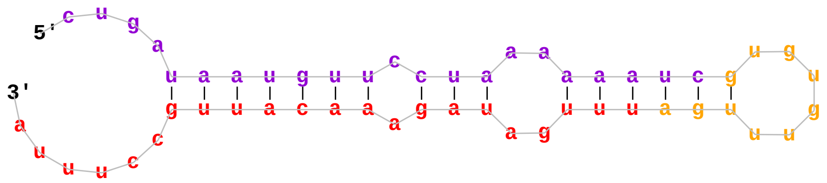

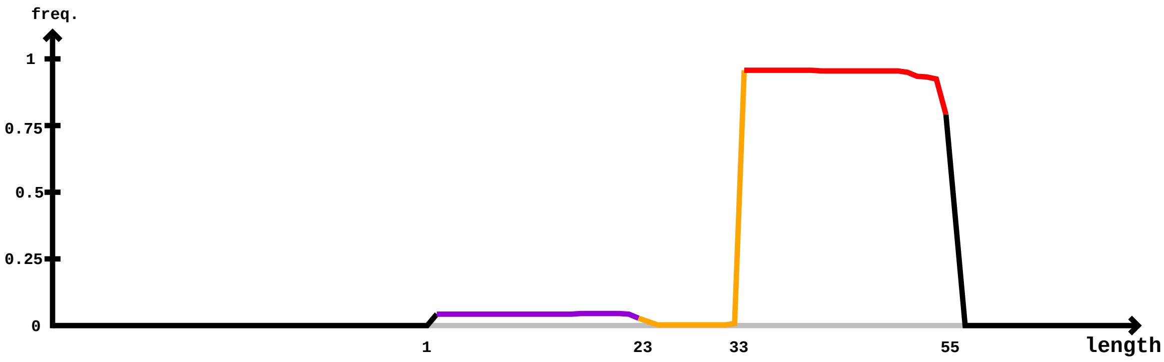

Star Mature

```
5'- gaaggcuaaaagaaaugcgaaagcagucacucguuuuacugauaauguuccuaaaaaaaucguguguuugauuugauagaaacauugccuuuaagaagauugacgagacauc   -3'    obs
    gaaggcuaaaagaaaugcgaaagcagucacucguuuuacugauaauguuccuaaaaaaaucguguguuugauuugauagaaacauugccuuuaagaagauugacgagacauc          exp
    ................(((....)))((((.((.(((((..(.(((((((.(((..((((((......))))))..))).))))))).)..))))).)).))))........     reads      mm         sample
    ......................................cugauaauguuccuaaaaaa......................................................     1          0          c15
    ......................................cugauaauguuccuaaaaaaau.....................................................     6          0          c15
    ......................................cugauaauguuccuaaaaaaauc...................................................     10         0          c15
    ......................................................aaaaaucguguguuugauuugauag.................................     1          0          c15
    .......................................................................uuugauagaaacauugcc.......................     1          0          c15
    .......................................................................uuugauagaaacauugccuuu....................     16         0          c15
    .......................................................................uuugauagaaaAauugccuuua...................     1          1          c15
    .......................................................................uuugauagaaacCuugccuuua...................     1          1          c15
    .......................................................................uuugauagaaacauugccuuuU...................     1          1          c15
    .......................................................................Auugauagaaacauugccuuua...................     1          1          c15
    .......................................................................uuugauagaaacauugccuuua...................     218        0          c15
    .......................................................................uuugauagaaacauugccuuuaa..................     7          0          c15
    .......................................................................uuugauagaaacauugccuuuaU..................     15         1          c15

    .......................................................................uuugauagaaacauugccuu.....................     1          0          c02
    .......................................................................Guugauagaaacauugccuuu....................     1          1          c02
    .......................................................................uuugauagaaacauugccuuu....................     5          0          c02
    .......................................................................Guugauagaaacauugccuuua...................     2          1          c02
    .......................................................................uuugauagaaacauugccuuua...................     12         0          c02

    ......................................................................auuugauagaaacCuugc........................     2          1          c04
    .......................................................................uuugauagaaacauugccuu.....................     1          0          c04
    .......................................................................uuugauagaaacauugccuuua...................     6          0          c04

    .......................................................................Guugauagaaacauugcc.......................     1          1          c16
    .......................................................................uuugauagaaacauugccu......................     1          0          c16
    .......................................................................Guugauagaaacauugccuuu....................     4          1          c16
    .......................................................................uuugauagaaacauugccuuu....................     20         0          c16
    .......................................................................uuugauagaaCcauugccuuua...................     1          1          c16
    .......................................................................uuugauagaaacauugccuuua...................     29         0          c16
    .......................................................................Guugauagaaacauugccuuua...................     1          1          c16
    .......................................................................uuugauagaaacauugccuuuaa..................     1          0          c16
    .......................................................................uuugauagaaacauugccuuuaU..................     1          1          c16
```

```
                                     Star                          Mature
gaaggcuaaaagaaaugcgaaagcagucacucguuuuacugauaauguuccuaaaaaaucguguguuugauuugauagaaacauugccuuuaagaagauugacgagacauc

.....................................................................uuugauagaaacauugcc.......................    4          0          c09
.....................................................................uuugauagaaacauugccuu.....................    1          0          c09
.....................................................................uuugauagaaacauugccuuu....................    7          0          c09
.....................................................................Guugauagaaacauugccuuua...................    2          1          c09
.....................................................................uuugauagaaacauugccuuua...................    14         0          c09
.....................................................................uuugauagaaacauugccuuuaa..................    1          0          c09
.....................................................................uuugauagaaacauugccuuuaU..................    1          1          c09
```

Provisional ID : group0_490
Score total : 78.5
Score for star read(s) : 3.9
Score for read counts : 72
Score for mfe : 1.5
Score for randfold : 1.6
Score for cons. seed : -0.6
Total read count : 153
Mature read count : 81
Loop read count : 0
Star read count : 72

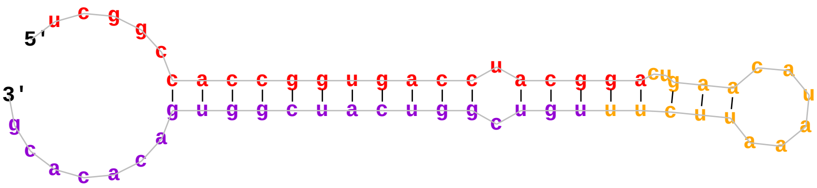

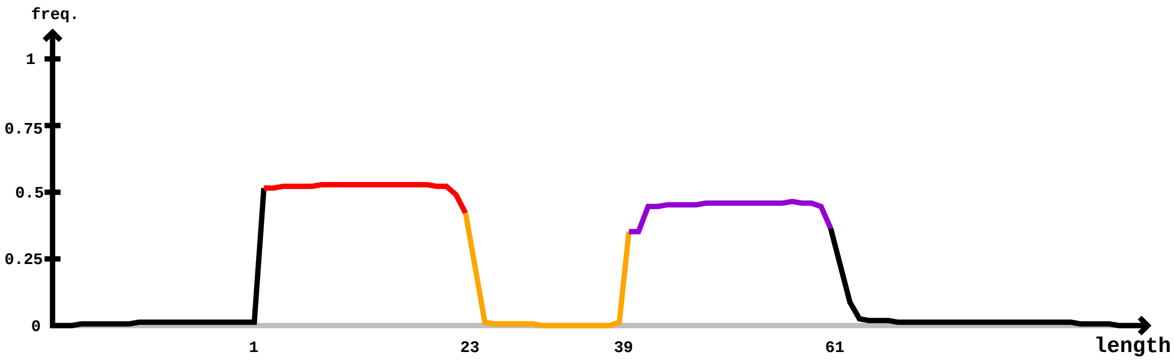

Mature Star

```
5'- aauagcaggucuauaguucgucggccaccggugaccuacggacugaacauaaauucuuugucggucaucggugacacacgaacugaaggucuaacguucgucggucaccagc  -3'  obs
    aauagcaggucuauaguucgucggccaccggugaccuacggacugaacauaaauucuuugucggucaucggugacacacgaacugaaggucuaacguucgucggucaccagc       exp
    ......((((((.((((((((..(.(((((((((((.(((((..(((......)))))))).))))))))))).)..)))))))).))))))....................  reads  mm  sample
    ....................ucggccaccggugaccuacg........................................................................  2  0  c15
    ....................ucggccaccggugaccuacgg.......................................................................  4  0  c15
    ....................ucggccaccggugaccuacgga......................................................................  46  0  c15
    ....................Ccggccaccggugaccuacgga......................................................................  1  1  c15
    ....................ucggccaccggugaccuacggaA.....................................................................  5  1  c15
    ..........................................................ugucggucaucggugacacac.................................  6  0  c15
    ..........................................................ugucggucaucggugacacacU................................  3  1  c15
    ..........................................................ugucggucaucggugacacacg................................  21  0  c15
    ..........................................................ugucggucaucggugacacacgU...............................  1  1  c15
    ..........................................................ugucggucaucggugacacacga...............................  1  0  c15
    ............................................................ucggucaucggugacacacga...............................  3  0  c15
    ............................................................ucggucaucggugacacacgaa..............................  6  0  c15
    ..................................................................aucggugacacacgaacuga..........................  1  0  c15
    ................................................................................aacugaaggucuaacguucgucggucacc...  1  0  c15

    ....................ucggccaccggugaccuacgg.......................................................................  2  0  c02
    ....................ucggccaccggugaccuacgga......................................................................  2  0  c02
    ..........................................................ugucggucaucggugacacac.................................  3  0  c02
    ..........................................................ugucggucaucggugacacacg................................  1  0  c02
    ..........................................................Ggucggucaucggugacacacg................................  1  1  c02
    ..............................................................ggucaucggugacacacgaa..............................  1  0  c02

    .......ggucuauaguucgucggccaccggugaccua..........................................................................  1  0  c16
    ....................ucggccaccggugaccuacg........................................................................  2  0  c16
    ....................ucggccaccggugaccuacgg.......................................................................  4  0  c16
    ....................Gcggccaccggugaccuacgg.......................................................................  1  1  c16
    ....................Gcggccaccggugaccuacgga......................................................................  1  1  c16
    ....................ucggccaccCgugaccuacgga......................................................................  1  1  c16
    ....................ucggccaccggugCccuacgga......................................................................  1  1  c16
    ....................ucggccaccggugaccuacgga......................................................................  2  0  c16
    ....................ucggccaccggugaccuacggaA.....................................................................  1  1  c16
    ..........................................................ugucggucaucggugacaca..................................  2  0  c16
    ..........................................................ugucggucaucggugacacac.................................  3  0  c16
```

```
                    Mature                                Star
aauagcaggucuauaguucgucggccaccggugaccuacggacugaacauaaauucuuugucggucaucggugacacacgaacugaaggucuaacguucgucggucaccagc
..........................................................ugucggucaucggugacacacg................................   6   0   c16
..........................................................ugucggucaucggugCcacacg................................   1   1   c16
............................................................ucggucaucggugacacacgaa..............................   1   0   c16

....................ucggccCccggugaccuacgga......................................................................   1   1   c04
..........................................................ugucggucaucggugacacacg................................   2   0   c04
............................................................ucggucaucggugacacacgaa..............................   1   0   c04

.auagcaggucuauaguucgucggccaccggugaccuacg........................................................................   1   0   c09
....................ucggccaccggugaccuacgga......................................................................   3   0   c09
....................ucggccaccggugaccuacggaA.....................................................................   1   1   c09
......................ggccaccggugaccuacggacu....................................................................   1   0   c09
..........................accggugaccuacggacugaaca...............................................................   1   0   c09
.........................................................uugucggucaucggugacacac.................................   1   0   c09
.........................................................uugucggucaucggugacacacga...............................   1   0   c09
..........................................................ugucggucaucggugaca....................................   1   0   c09
..........................................................ugucggucaucggugacacacg................................   2   0   c09
............................................................ucggucaucggugacacacga...............................   2   0   c09
............................................................ucggucaucggugacacacgaa..............................   1   0   c09
............................................................ucggucaucggugacacacgaaA.............................   1   1   c09
...........................................................................acacgaacugaaggucuaacguucgucggu.......   1   0   c09
```

Provisional ID	:	group0_514
Score total	:	641687.3
Score for star read(s)	:	3.9
Score for read counts	:	641677.2
Score for mfe	:	1.6
Score for randfold	:	1.6
Score for cons. seed	:	3
Total read count	:	1258634
Mature read count	:	836383
Loop read count	:	0
Star read count	:	422251

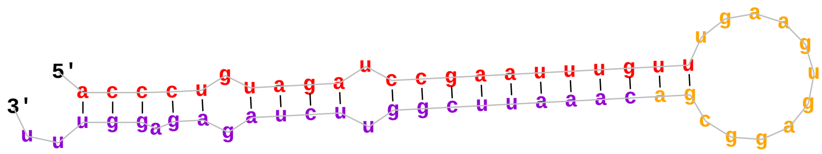

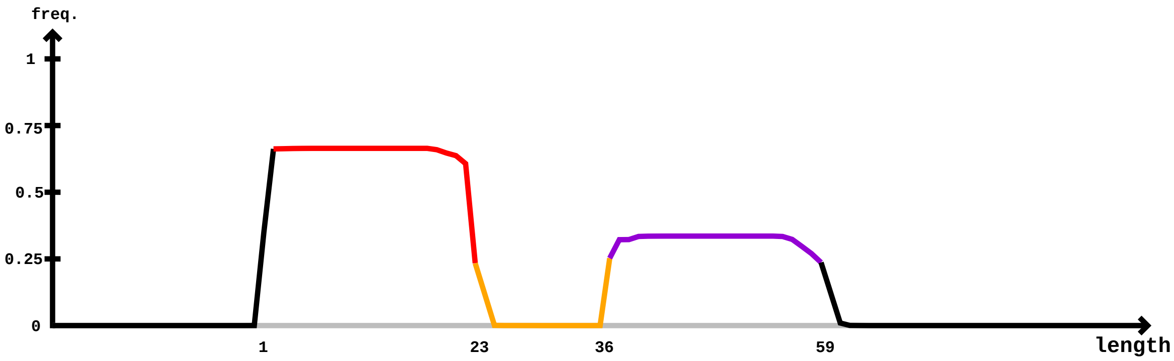

Mature **Star**

```
5'- caauuuuagugcccuacaucuacccuguagauccgaauuuguuugaagugaggcgacaaauucgguucuagagagguuuguguggugcaugcacugcaacguuucaagucug   -3'    obs
    caauuuuagugcccuacaucuacccuguagauccgaauuuguuugaagugaggcgacaaauucgguucuagagagguuuguguggugcaugcacugcaacguuucaagucug          exp
    ......((((((((((((..(((((.((((.((((((((((..........))))))))))).)))).)).)))..)))))).)...))))))...............   reads   mm   sample
    .................Gucuacccuguagauccgaauuugu......................................................................   2       1    c02
    .................Gucuacccuguagauccgaauuuguu.....................................................................   1       1    c02
    .................aAcuacccuguagauccgaauuuguu.....................................................................   1       1    c02
    ..................ucuacccuguagauccgaauuug.......................................................................   3       0    c02
    ..................ucuacccuguagauccgaauuugu......................................................................   2       0    c02
    ..................ucuacccuguagauccgaauuuguu.....................................................................   1       0    c02
    ..................Gcuacccuguagauccgaauuuguu.....................................................................   1       1    c02
    ...................cuacccuguagauccgaauuug.......................................................................   1       0    c02
    ...................cuUcccuguagauccgaauuugu......................................................................   1       1    c02
    ...................cuacccuguagauccgaauuugu......................................................................   4       0    c02
    ...................Auacccuguagauccgaauuugu......................................................................   1       1    c02
    ...................cGacccuguagauccgaauuugu......................................................................   1       1    c02
    ...................Uuacccuguagauccgaauuugu......................................................................   2       1    c02
    ...................cGacccuguagauccgaauuuguu.....................................................................   1       1    c02
    ...................cuacccuguagauccgaauuuguu.....................................................................   1       0    c02
    ....................uGcccuguagauccgaau..........................................................................   1       1    c02
    ....................uaccAuguagauccgaau..........................................................................   1       1    c02
    ....................uacccCguagauccgaau..........................................................................   1       1    c02
    ....................uacccuUuagauccgaau..........................................................................   1       1    c02
    ....................uacccuguagauccgaaC..........................................................................   5       1    c02
    ....................uacccAguagauccgaau..........................................................................   1       1    c02
    ....................uacccuguGgauccgaau..........................................................................   1       1    c02
    ....................uacccugAagauccgaau..........................................................................   1       1    c02
    ....................uacccuCuagauccgaau..........................................................................   5       1    c02
    ....................uacccuguagauccAaau..........................................................................   1       1    c02
    ....................uacccuguagaucUgaau..........................................................................   3       1    c02
    ....................uacccuguagauccgaaA..........................................................................   3       1    c02
    ....................uacccuguagauccgCau..........................................................................   1       1    c02
    ....................uacccuguagauccgaau..........................................................................   2516    0    c02
    ....................uCcccuguagauccgaau..........................................................................   9       1    c02
    ....................uUcccuguagauccgaau..........................................................................   2       1    c02
    ....................uacccuguagauccgaCu..........................................................................   1       1    c02
    ....................uacccuguagauccgaGu..........................................................................   3       1    c02
```

Mature Star

```
caauuuuagugcccuacaucuacccuguagauccgaauuuguuugaagugaggcgacaaauucgguucuagagagguuuguguggugcaugcacugcaacguuucaagucug
....................uacccuguagauccgaaG..........................................................................   8     1   c02
....................uacccuguagaGccgaau..........................................................................   2     1   c02
....................uacUcuguagauccgaau..........................................................................   2     1   c02
....................Gacccuguagauccgaau..........................................................................   174   1   c02
....................uacccuAuagauccgaau..........................................................................   2     1   c02
....................uaUccuguagauccgaau..........................................................................   2     1   c02
....................uacccuguagaucAgaau..........................................................................   4     1   c02
....................Aacccuguagauccgaau..........................................................................   15    1   c02
....................uaAccuguagauccgaau..........................................................................   4     1   c02
....................uacccuguagauAcgaau..........................................................................   3     1   c02
....................uacccuguagaucGgaau..........................................................................   1     1   c02
....................uaccUuguagauccgaau..........................................................................   1     1   c02
....................uacccuguagCuccgaau..........................................................................   10    1   c02
....................uacAcuguagauccgaau..........................................................................   14    1   c02
....................uacccuguagauccCaau..........................................................................   2     1   c02
....................uacccuguUgauccgaau..........................................................................   1     1   c02
....................uacccuguagauccgGau..........................................................................   1     1   c02
....................Cacccuguagauccgaau..........................................................................   1     1   c02
....................uacUcuguagauccgaauu.........................................................................   3     1   c02
....................uacccuguagauGcgaauu.........................................................................   1     1   c02
....................uacccuguagauccgaaAu.........................................................................   5     1   c02
....................uacccuguagauccgaauu.........................................................................   4655  0   c02
....................uacccCguagauccgaauu.........................................................................   1     1   c02
....................uacccuguagauccgaauG.........................................................................   3     1   c02
....................uacccuguagauccgaaCu.........................................................................   1     1   c02
....................uacccuCuagauccgaauu.........................................................................   6     1   c02
....................uacccuguagaucAgaauu.........................................................................   9     1   c02
....................uacAcuguagauccgaauu.........................................................................   25    1   c02
....................uacccugGagauccgaauu.........................................................................   1     1   c02
....................uacccuguagCuccgaauu.........................................................................   9     1   c02
....................Aacccuguagauccgaauu.........................................................................   30    1   c02
....................uaccUuguagauccgaauu.........................................................................   1     1   c02
....................uacccuguagauccgaauA.........................................................................   8     1   c02
....................uCcccuguagauccgaauu.........................................................................   10    1   c02
....................uacccuguagauccCaauu.........................................................................   2     1   c02
....................uacccuguagauccgaauC.........................................................................   4     1   c02
....................uaccAuguagauccgaauu.........................................................................   4     1   c02
....................uacccuguagaucUgaauu.........................................................................   2     1   c02
....................uacccuguagauAcgaauu.........................................................................   7     1   c02
....................uacccuguaCauccgaauu.........................................................................   2     1   c02
....................uacccugAagauccgaauu.........................................................................   2     1   c02
....................uaAccuguagauccgaauu.........................................................................   2     1   c02
....................uaUccuguagauccgaauu.........................................................................   3     1   c02
....................uacccuguagauccgaUuu.........................................................................   1     1   c02
....................Cacccuguagauccgaauu.........................................................................   3     1   c02
....................uacccuguagauUcgaauu.........................................................................   3     1   c02
....................uacccugCagauccgaauu.........................................................................   1     1   c02
....................uacccuAuagauccgaauu.........................................................................   4     1   c02
....................uacccuguagauccAaauu.........................................................................   2     1   c02
....................Gacccuguagauccgaauu.........................................................................   343   1   c02
....................uacccAguagauccgaauu.........................................................................   3     1   c02
....................uacccuguagUuccgaauu.........................................................................   1     1   c02
....................uacccuguagauccUaauu.........................................................................   1     1   c02
....................uacccuguagauccgaGuu.........................................................................   2     1   c02
....................uacccuguagauccgaaGu.........................................................................   7     1   c02
....................uacGcuguagauccgaauu.........................................................................   3     1   c02
....................Cacccuguagauccgaauuu........................................................................   2     1   c02
....................uaccUuguagauccgaauuu........................................................................   1     1   c02
....................uacUcuguagauccgaauuu........................................................................   1     1   c02
....................uacccuguagauccgaaCuu........................................................................   2     1   c02
....................uacccuguagauUcgaauuu........................................................................   1     1   c02
....................uacccuguagauccgaGuuu........................................................................   1     1   c02
....................uacccuguagauccgaauuA........................................................................   11    1   c02
....................uacccuguagauccgaauAu........................................................................   1     1   c02
....................uacccuguaUauccgaauuu........................................................................   1     1   c02
....................Gacccuguagauccgaauuu........................................................................   260   1   c02
....................uacccuguagaucUgaauuu........................................................................   2     1   c02
....................uacccuguagauccgaauuu........................................................................   3557  0   c02
....................uacccuguagauccgaaAuu........................................................................   1     1   c02
....................uacccuguagauccgaauuC........................................................................   6     1   c02
```

```
                    Mature                           Star
caauuuuagugcccuacaucuacccuguagauccgaauuuguuugaagugaggcgacaaauucgguucuagagagguuugugguggugcaugcacugcaacguuucaagucug
....................uacccuguagauccgaauGu........................................................................ 3     1    c02
....................uacccuUuagauccgaauuu........................................................................ 1     1    c02
....................uaccAuguagauccgaauuu........................................................................ 3     1    c02
....................uacccuguagaAccgaauuu........................................................................ 2     1    c02
....................uacccuguagauccgGauuu........................................................................ 1     1    c02
....................uacccCguagauccgaauuu........................................................................ 3     1    c02
....................uacccuguagaucAgaauuu........................................................................ 7     1    c02
....................uacGcuguagauccgaauuu........................................................................ 1     1    c02
....................uaAccuguagauccgaauuu........................................................................ 4     1    c02
....................uacccuguagauccgaauuG........................................................................ 2     1    c02
....................uacccuguagauccgCauuu........................................................................ 1     1    c02
....................uacccuCuagauccgaauuu........................................................................ 5     1    c02
....................uacccuguagauAcgaauuu........................................................................ 3     1    c02
....................Aacccuguagauccgaauuu........................................................................ 11    1    c02
....................uacccuguagaucGgaauuu........................................................................ 1     1    c02
....................uacccugCagauccgaauuu........................................................................ 2     1    c02
....................uacccuguagauccgaaGuu........................................................................ 1     1    c02
....................uacccuguagauccCaauuu........................................................................ 1     1    c02
....................uCcccuguagauccgaauuu........................................................................ 6     1    c02
....................uacccuguagauccgaUuuu........................................................................ 1     1    c02
....................uacccuguagGuccgaauuu........................................................................ 2     1    c02
....................uacAcuguagauccgaauuu........................................................................ 30    1    c02
....................uacccuguagauccUaauuu........................................................................ 1     1    c02
....................uacccuguaCauccgaauuu........................................................................ 3     1    c02
....................uacccGguagauccgaauuu........................................................................ 1     1    c02
....................uacccuguagauccgaauCu........................................................................ 2     1    c02
....................uacccuguGgauccgaauuu........................................................................ 1     1    c02
....................uacccuguagCuccgaauuu........................................................................ 9     1    c02
....................uGcccuguagauccgaauuu........................................................................ 1     1    c02
....................uacccuguagauccgaauuug....................................................................... 9517  0    c02
....................uacccuguagauccgaaAuug....................................................................... 4     1    c02
....................uacccuguagauccgaUuuug....................................................................... 2     1    c02
....................Aacccuguagauccgaauuug....................................................................... 55    1    c02
....................uacccuguagauccgGauug........................................................................ 2     1    c02
....................uacccuguagauccgaauuU........................................................................ 16    1    c02
....................uacccuguagauccgCauug........................................................................ 2     1    c02
....................uacccuguaAauccgaauuug....................................................................... 1     1    c02
....................uacccuguagauccgaauuGg....................................................................... 1     1    c02
....................uaccAuguagauccgaauuug....................................................................... 7     1    c02
....................uaccUuguagauccgaauuug....................................................................... 3     1    c02
....................uacccuguagGuccgaauuug....................................................................... 1     1    c02
....................uUcccuguagauccgaauuug....................................................................... 1     1    c02
....................uacccAguagauccgaauuug....................................................................... 2     1    c02
....................uacccuguagauccgaauGug....................................................................... 1     1    c02
....................uacccuguGgauccgaauuug....................................................................... 3     1    c02
....................Gacccuguagauccgaauuug....................................................................... 689   1    c02
....................uacccuguaUauccgaauuug....................................................................... 1     1    c02
....................uacccGguagauccgaauuug....................................................................... 2     1    c02
....................uacccuAuagauccgaauuug....................................................................... 1     1    c02
....................uacccuUuagauccgaauuug....................................................................... 1     1    c02
....................uacccuguagauccgaaCuug....................................................................... 4     1    c02
....................uacUcuguagauccgaauuug....................................................................... 3     1    c02
....................uaAccuguagauccgaauuug....................................................................... 12    1    c02
....................uacccuguaCauccgaauuug....................................................................... 3     1    c02
....................uacccugAagauccgaauuug....................................................................... 8     1    c02
....................uGcccuguagauccgaauuug....................................................................... 2     1    c02
....................uacccuguCgauccgaauuug....................................................................... 1     1    c02
....................uacccuguagauccUaauuug....................................................................... 4     1    c02
....................uacccuguagauGcgaauuug....................................................................... 1     1    c02
....................uacccuguagauccgaauuCg....................................................................... 1     1    c02
....................uaccGuguagauccgaauuug....................................................................... 1     1    c02
....................uacccuguagUuccgaauuug....................................................................... 1     1    c02
....................uacccCguagauccgaauuug....................................................................... 4     1    c02
....................uacccuguagauccAaauuug....................................................................... 1     1    c02
....................uacccuguagauccCaauuug....................................................................... 2     1    c02
....................uaUccuguagauccgaauuug....................................................................... 3     1    c02
....................uacccuguagauccgaauuuA....................................................................... 8     1    c02
....................uacccuguagauccgaauuuC....................................................................... 5     1    c02
....................uacccuguagCuccgaauuug....................................................................... 39    1    c02
....................uacAcuguagauccgaauuug....................................................................... 46    1    c02
```

```
                    Mature               Star
caauuuuagugcccuacaucuacccuguagauccgaauuuguuugaagugaggcgacaaauucgguucuagagagguuuguguggugcaugcacugcaacguuucaagucug
....................uacccuguagauccgaauAug.......................................................................	3	1	c02
....................uCcccuguagauccgaauuug.......................................................................	20	1	c02
....................uacGcuguagauccgaauuug.......................................................................	3	1	c02
....................uacccuguagauccgaaGuug.......................................................................	8	1	c02
....................uacccuguagauccgaGuuug.......................................................................	2	1	c02
....................uacccuCuagauccgaauuug.......................................................................	9	1	c02
....................uacccugCagauccgaauuug.......................................................................	1	1	c02
....................uacccuguagauccgaauCug.......................................................................	1	1	c02
....................Cacccuguagauccgaauuug.......................................................................	4	1	c02
....................uacccuguagaucUgaauuug.......................................................................	7	1	c02
....................uacccuguagauccgUauuug.......................................................................	2	1	c02
....................uacccuguagaCccgaauuug.......................................................................	1	1	c02
....................uacccuguagaucAgaauuug.......................................................................	18	1	c02
....................uacccugGagauccgaauuug.......................................................................	1	1	c02
....................uacccuguagauccgaauuAg.......................................................................	4	1	c02
....................uacccuguagauAcgaauuug.......................................................................	22	1	c02
....................uacccuguagaucUgaauuugu......................................................................	69	1	c02
....................uacccuguaAauccgaauuugu......................................................................	6	1	c02
....................uacccugGagauccgaauuugu......................................................................	18	1	c02
....................uacccuguagauccgaauuugu......................................................................	148030	0	c02
....................uacccuguagauccgGauuugu......................................................................	23	1	c02
....................uCcccuguagauccgaauuugu......................................................................	368	1	c02
....................uacccuguagauccUaauuugu......................................................................	66	1	c02
....................uacccuguagauccgaauuugG......................................................................	22	1	c02
....................uacccuguagauccgaUuuugu......................................................................	43	1	c02
....................uacAcuguagauccgaauuugu......................................................................	846	1	c02
....................uacccGguagauccgaauuugu......................................................................	23	1	c02
....................uacccuguagUuccgaauuugu......................................................................	18	1	c02
....................uacccuguagauccgaaGuugu......................................................................	39	1	c02
....................uacccugAagauccgaauuugu......................................................................	65	1	c02
....................uacccuguagCuccgaauuugu......................................................................	462	1	c02
....................uUccuguagauccgaauuugu......................................................................	19	1	c02
....................uaccUuguagauccgaauuugu......................................................................	50	1	c02
....................uacccuguagGuccgaauuugu......................................................................	17	1	c02
....................uacccuguagauccgaauAugu......................................................................	63	1	c02
....................uacccuguagauccgaauuuAu......................................................................	28	1	c02
....................uacccuguagauccgaauuuCu......................................................................	143	1	c02
....................uacccuguagauccCaauuugu......................................................................	43	1	c02
....................uacccuguagauccgUauuugu......................................................................	18	1	c02
....................uacccuguagauAcgaauuugu......................................................................	212	1	c02
....................uacccuguUgauccgaauuugu......................................................................	18	1	c02
....................uacccuguGgauccgaauuugu......................................................................	56	1	c02
....................uacccuguagauccgaauuugA......................................................................	75	1	c02
....................uacUcuguagauccgaauuugu......................................................................	50	1	c02
....................uacccuguagauccgaauuCgu......................................................................	36	1	c02
....................uacccuguagauccgaauCugu......................................................................	32	1	c02
....................uacGcuguagauccgaauuugu......................................................................	75	1	c02
....................uacccuguagauccgaauuAgu......................................................................	52	1	c02
....................uacccuguaCauccgaauuugu......................................................................	31	1	c02
....................uaccAuguagauccgaauuugu......................................................................	80	1	c02
....................uacccuguagaucGgaauuugu......................................................................	28	1	c02
....................Aacccuguagauccgaauuugu......................................................................	827	1	c02
....................uacccCguagauccgaauuugu......................................................................	86	1	c02
....................uacccuguaUauccgaauuugu......................................................................	33	1	c02
....................uacccuguagaAccgaauuugu......................................................................	37	1	c02
....................uGcccuguagauccgaauuugu......................................................................	32	1	c02
....................uacccuCuagauccgaauuugu......................................................................	172	1	c02
....................uacccuguagauccgaauuuUu......................................................................	38	1	c02
....................uacccuguagauccgaauuGgu......................................................................	11	1	c02
....................uacccuguagauccgaaCuugu......................................................................	38	1	c02
....................uacccuguagaCccgaauuugu......................................................................	28	1	c02
....................Cacccuguagauccgaauuugu......................................................................	66	1	c02
....................uaAccuguagauccgaauuugu......................................................................	101	1	c02
....................uacccuguagaGccgaauuugu......................................................................	15	1	c02
....................uacccAguagauccgaauuugu......................................................................	77	1	c02
....................uacccuguagauccgaauuugC......................................................................	36	1	c02
....................uacccuguagauccAaauuugu......................................................................	30	1	c02
....................uacccuUuagauccgaauuugu......................................................................	71	1	c02
....................uacccuguagauccgaGuuugu......................................................................	16	1	c02
....................uacccuguagauccgaauGugu......................................................................	8	1	c02
```

```
                     Mature              Star
caauuuuagugcccuacaucuacccuguagauccgaauuuguuugaagugaggcgacaaauucgguucuagagagguuugugguggugcaugcacugcaacguuucaagucug
....................uacccugCagauccgaauuugu......................................................................    28      1    c02
....................uacccuguagauGcgaauuugu......................................................................    24      1    c02
....................uacccuguagauccgaaAuugu......................................................................    55      1    c02
....................uacccuguagauccgaCuuugu......................................................................    7       1    c02
....................uacccuguagauccgCauuugu......................................................................    10      1    c02
....................uaccGuguagauccgaauuugu......................................................................    20      1    c02
....................uacccuguagaucAgaauuugu......................................................................    251     1    c02
....................Gacccuguagauccgaauuugu......................................................................    11725   1    c02
....................uacccuguagauUcgaauuugu......................................................................    38      1    c02
....................uacccuAuagauccgaauuugu......................................................................    42      1    c02
....................uacccuguCgauccgaauuugu......................................................................    3       1    c02
....................uaGccuguagauccgaauuugu......................................................................    15      1    c02
....................uaUccuguagauccgaauuugu......................................................................    53      1    c02
....................uacccuguagauccUaauuuguu.....................................................................    1       1    c02
....................uacccuguagauccgaauuuguA.....................................................................    1067    1    c02
....................uacccuguagauccgaauuugGu.....................................................................    2       1    c02
....................uacccuguagauccgaCuuuguu.....................................................................    1       1    c02
....................uacccuguagauccgaaGuuguu.....................................................................    1       1    c02
....................uaccAuguagauccgaauuuguu.....................................................................    3       1    c02
....................Cacccuguagauccgaauuuguu.....................................................................    1       1    c02
....................uacccuguagaucGgaauuuguu.....................................................................    1       1    c02
....................uaAccuguagauccgaauuuguu.....................................................................    2       1    c02
....................uacccuCuagauccgaauuuguu.....................................................................    5       1    c02
....................uaccGuguagauccgaauuuguu.....................................................................    1       1    c02
....................uacccCguagauccgaauuuguu.....................................................................    2       1    c02
....................Gacccuguagauccgaauuuguu.....................................................................    162     1    c02
....................uacccuguGgauccgaauuuguu.....................................................................    1       1    c02
....................uacccuguagauccgaaCuuguu.....................................................................    2       1    c02
....................uacccuguagauccgaUuuuguu.....................................................................    1       1    c02
....................uacccAguagauccgaauuuguu.....................................................................    1       1    c02
....................uacccuguagauccgaauuuguC.....................................................................    149     1    c02
....................uacccuguagauccgaauuuguG.....................................................................    132     1    c02
....................uacccuguagauAcgaauuuguu.....................................................................    3       1    c02
....................uacccugCagauccgaauuuguu.....................................................................    1       1    c02
....................uacccuguagaAccgaauuuguu.....................................................................    1       1    c02
....................Aacccuguagauccgaauuuguu.....................................................................    16      1    c02
....................uacccuguagauccgaauuuguu.....................................................................    2089    0    c02
....................uCcccuguagauccgaauuuguu.....................................................................    3       1    c02
....................uacccuguagaucAgaauuuguu.....................................................................    2       1    c02
....................uacAcuguagauccgaauuuguu.....................................................................    11      1    c02
....................uacccuguagauccgaauAuguu.....................................................................    2       1    c02
....................uacccugAagauccgaauuuguu.....................................................................    1       1    c02
....................uacccuguagGuccgaauuuguu.....................................................................    1       1    c02
....................uacccuguagaucUgaauuuguu.....................................................................    1       1    c02
....................uacccuguagCuccgaauuuguu.....................................................................    9       1    c02
....................uacccuguagauccgaauuuguuu....................................................................    46      0    c02
....................uacccuguagauccgaauuuguGu....................................................................    3       1    c02
....................uacccuguagauccgCauuuguuu....................................................................    1       1    c02
....................uacccuguagauccgaauuuguAu....................................................................    20      1    c02
....................uacccuguagaucGgaauuuguuu....................................................................    1       1    c02
....................uacccuguagauccgaauuuguuG....................................................................    8       1    c02
....................uacccuguagauccgaauuuguuA....................................................................    16      1    c02
....................Gacccuguagauccgaauuuguuu....................................................................    2       1    c02
....................uacccuguagauccgaauuuguCu....................................................................    2       1    c02
....................uacccuguagauccgaauuuguuuU...................................................................    3       1    c02
....................uacccuguagauAcgaauuuguuug...................................................................    1       1    c02
....................uacccuguagauccgaauuuguuug...................................................................    5       0    c02
....................Gacccuguagauccgaauuuguuug...................................................................    2       1    c02
....................uacccuguagauccgaauuuguuuA...................................................................    1       1    c02
....................uacccuguagauccgaauuuguuugU..................................................................    3       1    c02
....................Gacccuguagauccgaauuuguuuga..................................................................    1       1    c02
....................uacccuguagauccgaauuuguuuga..................................................................    3       0    c02
....................uacccuguagauccgaauuuguuugaa.................................................................    3       0    c02
....................uacccuguagauccgaauuuguuugaag................................................................    1       0    c02
....................uacccuguagauccgaauuuguuugaagu...............................................................    2       0    c02
....................uacccuguagauccgaauuuguuugaagug..............................................................    1       0    c02
.....................accAuguagauccgaauu.........................................................................    2       1    c02
.....................acccuguagCuccgaauu.........................................................................    1       1    c02
.....................acccuCuagauccgaauu.........................................................................    5       1    c02
.....................acccuguagaucAgaauu.........................................................................    8       1    c02
```

Mature **Star**

caauuuuagugcccuacaucuacccuguagauccgaauuuguuugaagugaggcgacaaauucgguucuagagagguuuguguggugcaugcacugcaacguuucaagucug			
.....................acccuguagauccgaauu...	1565	0	c02
.....................acccuguagGuccgaauu...	1	1	c02
.....................acccuguagauccgaUuu...	1	1	c02
.....................acccuguagauccCaauu...	2	1	c02
.....................acccuguagauccgaaAu...	2	1	c02
.....................acccuguaUauccgaauu...	1	1	c02
.....................acccuguagUuccgaauu...	1	1	c02
.....................Ccccuguagauccgaauu...	732	1	c02
.....................acccugCagauccgaauu...	1	1	c02
.....................Gcccuguagauccgaauu...	3	1	c02
.....................acccGguagauccgaauu...	1	1	c02
.....................acccuguagauccgaaCu...	1	1	c02
.....................acccuguagauccgUauu...	2	1	c02
.....................acAcuguagauccgaauu...	9	1	c02
.....................acccuguagauAcgaauu...	4	1	c02
.....................acccAguagauccgaauu...	3	1	c02
.....................acccuguagauccgaauA...	2	1	c02
.....................aAccuguagauccgaauu...	1	1	c02
.....................accUuguagauccgaauu...	1	1	c02
.....................acccuguagaucUgaauu...	1	1	c02
.....................acccuguagauccgaaGu...	1	1	c02
.....................acccugCagauccgaauuu..	1	1	c02
.....................acccuguagauccUaauuu..	1	1	c02
.....................acccuCuagauccgaauuu..	8	1	c02
.....................acGcuguagauccgaauuu..	1	1	c02
.....................acccuguagauccgaauuu..	2245	0	c02
.....................acccuguagauccgaauuC..	1	1	c02
.....................acccuguagauccgaaGuu..	1	1	c02
.....................acccuguagauccgUauuu..	1	1	c02
.....................acccuguagauccgaUuuu..	1	1	c02
.....................aUccuguagauccgaauuu..	1	1	c02
.....................aAccuguagauccgaauuu..	5	1	c02
.....................acccugAagauccgaauuu..	2	1	c02
.....................Gcccuguagauccgaauuu..	4	1	c02
.....................acccuAuagauccgaauuu..	1	1	c02
.....................acccuguagaucUgaauuu..	1	1	c02
.....................acccuguagCuccgaauuu..	9	1	c02
.....................acccuguagauccgaauuA..	2	1	c02
.....................acccuguagauccgaCuuu..	1	1	c02
.....................acccGguagauccgaauuu..	3	1	c02
.....................acAcuguagauccgaauuu..	5	1	c02
.....................accUuguagauccgaauuu..	1	1	c02
.....................Ccccuguagauccgaauuu..	1069	1	c02
.....................acccuguagauccgaaAuu..	1	1	c02
.....................acccuguagaucAgaauuu..	6	1	c02
.....................acccuguagauAcgaauuu..	6	1	c02
.....................acccAguagauccgaauuu..	2	1	c02
.....................acccuguagauccgaauuG..	4	1	c02
.....................acccuguGgauccgaauuu..	2	1	c02
.....................acccuguagUuccgaauuu..	1	1	c02
.....................accAuguagauccgaauuu..	6	1	c02
.....................acccuguagaAccgaauuu..	1	1	c02
.....................acccuguaCauccgaauuu..	2	1	c02
.....................acccuguagauccgaauCug...	1	1	c02
.....................accAuguagauccgaauuug...	8	1	c02
.....................accUuguagauccgaauuug...	2	1	c02
.....................acccuCuagauccgaauuug...	12	1	c02
.....................acccuguGgauccgaauuug...	1	1	c02
.....................acccuguagauccgaauuuC...	6	1	c02
.....................acccuguagauccgaauAug...	4	1	c02
.....................acGcuguagauccgaauuug...	1	1	c02
.....................acccuguagaAccgaauuug...	2	1	c02
.....................acccuguagauccUaauuug...	2	1	c02
.....................acccuguagauccgaaGuug...	2	1	c02
.....................acUcuguagauccgaauuug...	3	1	c02
.....................acccuguagauUcgaauuug...	1	1	c02
.....................acccuguagauccgUauuug...	1	1	c02
.....................acccuguagauccgaauuuA...	3	1	c02
.....................acccuguagauccgaauuAg...	3	1	c02
.....................Ccccuguagauccgaauuug...	1942	1	c02

```
                    Mature              Star
caauuuuagugcccuacaucuacccuguagauccgaauuuguuugaagugaggcgacaaauucgguucuagagagguuugugugguggcaugcacugcaacguuucaagucug
.....................acccuguagauAcgaauuug.......................................................................	4	1	c02
.....................acccuguaUauccgaauuug.......................................................................	3	1	c02
.....................acccuguagaGccgaauuug.......................................................................	1	1	c02
.....................Ucccuguagauccgaauuug.......................................................................	2	1	c02
.....................acccuguagauccAaauuug.......................................................................	1	1	c02
.....................acccGguagauccgaauuug.......................................................................	2	1	c02
.....................aUccuguagauccgaauuug.......................................................................	2	1	c02
.....................acccCguagauccgaauuug.......................................................................	3	1	c02
.....................acccuguagauccgaauuCg.......................................................................	1	1	c02
.....................acccuguagaucUgaauuug.......................................................................	3	1	c02
.....................acccuguaCauccgaauuug.......................................................................	5	1	c02
.....................acccuguagauccgaUuuug.......................................................................	1	1	c02
.....................acccuguagauccgaaCuug.......................................................................	1	1	c02
.....................acccuguagauccgaGuuug.......................................................................	2	1	c02
.....................Gcccuguagauccgaauuug.......................................................................	3	1	c02
.....................acccuUuagauccgaauuug.......................................................................	4	1	c02
.....................acccuguagaucAgaauuug.......................................................................	17	1	c02
.....................acAcuguagauccgaauuug.......................................................................	19	1	c02
.....................acccuguagCuccgaauuug.......................................................................	14	1	c02
.....................acccuguagauccgaaAuug.......................................................................	2	1	c02
.....................acccugAagauccgaauuug.......................................................................	1	1	c02
.....................acccuguagaucGgaauuug.......................................................................	1	1	c02
.....................acccuguagauccgaauuug.......................................................................	3605	0	c02
.....................acccuguagauccgaauuuU.......................................................................	3	1	c02
.....................acccAguagauccgaauuug.......................................................................	4	1	c02
.....................acccuguagaAccgaauuugu......................................................................	5	1	c02
.....................acccuguagaucGgaauuugu......................................................................	3	1	c02
.....................acccuCuagauccgaauuugu......................................................................	38	1	c02
.....................acccuguagauccgaaCuugu......................................................................	5	1	c02
.....................acccAguagauccgaauuugu......................................................................	13	1	c02
.....................acccuguagauUcgaauuugu......................................................................	6	1	c02
.....................acccuguagauccgaauCugu......................................................................	1	1	c02
.....................acccuguagauccgUauuugu......................................................................	1	1	c02
.....................acccuguagauccgGauuugu......................................................................	3	1	c02
.....................aUccuguagauccgaauuugu......................................................................	15	1	c02
.....................acccuguagauccgaauuugA......................................................................	15	1	c02
.....................acccuguGgauccgaauuugu......................................................................	4	1	c02
.....................acccuguagauccgaCuuugu......................................................................	1	1	c02
.....................Ucccuguagauccgaauuugu......................................................................	6	1	c02
.....................acccuguagauccgaUuuugu......................................................................	4	1	c02
.....................acccuguUgauccgaauuugu......................................................................	4	1	c02
.....................aAccuguagauccgaauuugu......................................................................	15	1	c02
.....................acccuguagauccgaauuugG......................................................................	11	1	c02
.....................acUcuguagauccgaauuugu......................................................................	3	1	c02
.....................acccuAuagauccgaauuugu......................................................................	1	1	c02
.....................acccuguagUuccgaauuugu......................................................................	1	1	c02
.....................Gcccuguagauccgaauuugu......................................................................	16	1	c02
.....................acGcuguagauccgaauuugu......................................................................	1	1	c02
.....................accGuguagauccgaauuugu......................................................................	1	1	c02
.....................acccugCagauccgaauuugu......................................................................	1	1	c02
.....................acccuguagauccgaaAuugu......................................................................	5	1	c02
.....................acccGguagauccgaauuugu......................................................................	4	1	c02
.....................acccuguagauccAaauuugu......................................................................	7	1	c02
.....................acccuguagCuccgaauuugu......................................................................	55	1	c02
.....................acccuguagaucUgaauuugu......................................................................	12	1	c02
.....................acccuguagauccCaauuugu......................................................................	6	1	c02
.....................acccCguagauccgaauuugu......................................................................	5	1	c02
.....................aGccuguagauccgaauuugu......................................................................	1	1	c02
.....................accUuguagauccgaauuugu......................................................................	8	1	c02
.....................acccuguaAauccgaauuugu......................................................................	2	1	c02
.....................acccuguagauccgaauuugC......................................................................	6	1	c02
.....................acccuguagauccgaauuuAu......................................................................	1	1	c02
.....................acccuguagauccgaauuugu......................................................................	14750	0	c02
.....................acccugAagauccgaauuugu......................................................................	6	1	c02
.....................acccuguagauccgaGuuugu......................................................................	1	1	c02
.....................acccugGagauccgaauuugu......................................................................	2	1	c02
.....................acccuguaUauccgaauuugu......................................................................	5	1	c02
.....................acccuguagauccgaaGuugu......................................................................	6	1	c02
.....................accAuguagauccgaauuugu......................................................................	24	1	c02
.....................acccuguagauccgaauuuCu......................................................................	25	1	c02
```

```
                     Mature                 Star
caauuuuagugcccuacaucuacccuguagauccgaauuuguuugaagugaggcgacaaauucgguucuagagagguuuguguggugcaugcacugcaacguuucaagucug
.....................acccuguagauccgaauAugu......................................................................   11      1   c02
.....................Ccccuguagauccgaauuugu......................................................................   7193    1   c02
.....................acccuguagGuccgaauuugu......................................................................   2       1   c02
.....................acccuguagauccgaauuCgu......................................................................   3       1   c02
.....................acccuguagauAcgaauuugu......................................................................   36      1   c02
.....................acccuguagauccgaauuuUu......................................................................   11      1   c02
.....................acccuguagauccgaauuAgu......................................................................   6       1   c02
.....................acccuUuagauccgaauuugu......................................................................   10      1   c02
.....................acccuguagaucAgaauuugu......................................................................   67      1   c02
.....................acAcuguagauccgaauuugu......................................................................   63      1   c02
.....................acccuguaCauccgaauuugu......................................................................   9       1   c02
.....................acccuguagauccUaauuugu......................................................................   11      1   c02
.....................acccuguagauccgCauuuguu.....................................................................   3       1   c02
.....................acccuguagauccgaGuuuguu.....................................................................   14      1   c02
.....................acUcuguagauccgaauuuguu.....................................................................   22      1   c02
.....................acccAguagauccgaauuuguu.....................................................................   52      1   c02
.....................acccuguagauccgaauuCguu.....................................................................   10      1   c02
.....................acccuguaCauccgaauuuguu.....................................................................   14      1   c02
.....................accGuguagauccgaauuuguu.....................................................................   2       1   c02
.....................acccuguagauccgaauuuguC.....................................................................   13      1   c02
.....................acccuguagauGcgaauuuguu.....................................................................   2       1   c02
.....................acccuguagauccgaaCuuguu.....................................................................   14      1   c02
.....................acccuguagauccgaauGuguu.....................................................................   3       1   c02
.....................acccuguagauccgaauAuguu.....................................................................   36      1   c02
.....................acGcuguagauccgaauuuguu.....................................................................   2       1   c02
.....................acccuguagauccgaauuuguA.....................................................................   97      1   c02
.....................aUccuguagauccgaauuuguu.....................................................................   31      1   c02
.....................acccuguagauccgaauuugCu.....................................................................   13      1   c02
.....................aAccuguagauccgaauuuguu.....................................................................   51      1   c02
.....................acccugGagauccgaauuuguu.....................................................................   2       1   c02
.....................acccuAuagauccgaauuuguu.....................................................................   9       1   c02
.....................acccuguagauUcgaauuuguu.....................................................................   15      1   c02
.....................acccuguagauccgaauuAguu.....................................................................   35      1   c02
.....................acccuguagauccgaauuuAuu.....................................................................   4       1   c02
.....................acccuguagauccAaauuuguu.....................................................................   5       1   c02
.....................acccuguagauccgaCuuuguu.....................................................................   3       1   c02
.....................Gcccuguagauccgaauuuguu.....................................................................   33      1   c02
.....................acccuguagUuccgaauuuguu.....................................................................   4       1   c02
.....................acccugCagauccgaauuuguu.....................................................................   7       1   c02
.....................acccuguagaucGgaauuuguu.....................................................................   11      1   c02
.....................acccuguagauccgaUuuuguu.....................................................................   8       1   c02
.....................acAcuguagauccgaauuuguu.....................................................................   191     1   c02
.....................acccuCuagauccgaauuuguu.....................................................................   104     1   c02
.....................acccCguagauccgaauuuguu.....................................................................   20      1   c02
.....................Ccccuguagauccgaauuuguu.....................................................................   22367   1   c02
.....................acccuguagauccgaauCuguu.....................................................................   10      1   c02
.....................acccuguagauccgaaAuuguu.....................................................................   21      1   c02
.....................acccuguagaCccgaauuuguu.....................................................................   5       1   c02
.....................acccuguagauccUaauuuguu.....................................................................   23      1   c02
.....................accUuguagauccgaauuuguu.....................................................................   20      1   c02
.....................acccuguagaAccgaauuuguu.....................................................................   9       1   c02
.....................acccuguagaucUgaauuuguu.....................................................................   31      1   c02
.....................acccuguagaGccgaauuuguu.....................................................................   7       1   c02
.....................aGccuguagauccgaauuuguu.....................................................................   1       1   c02
.....................acccuguGgauccgaauuuguu.....................................................................   18      1   c02
.....................Ucccuguagauccgaauuuguu.....................................................................   13      1   c02
.....................acccuUuagauccgaauuuguu.....................................................................   28      1   c02
.....................acccuguaAauccgaauuuguu.....................................................................   3       1   c02
.....................acccuguagauAcgaauuuguu.....................................................................   108     1   c02
.....................acccuguUgauccgaauuuguu.....................................................................   9       1   c02
.....................acccuguagaucAgaauuuguu.....................................................................   183     1   c02
.....................acccugAagauccgaauuuguu.....................................................................   27      1   c02
.....................acccuguagCuccgaauuuguu.....................................................................   149     1   c02
.....................acccuguagauccgaauuuguG.....................................................................   11      1   c02
.....................acccuguagauccgaauuuCuu.....................................................................   75      1   c02
.....................acccuguagauccgaaGuuguu.....................................................................   18      1   c02
.....................acccuguagauccgaauuuguu.....................................................................   49132   0   c02
.....................acccuguagauccgaauuugGu.....................................................................   11      1   c02
.....................acccuguagGuccgaauuuguu.....................................................................   6       1   c02
.....................acccuguagauccgaauuuUuu.....................................................................   16      1   c02
```

Mature Star

```
caauuuuagugcccuacaucuacccuguagauccgaauuuguuugaagugaggcgacaaauucgguucuagagagguuuguguggugcaugcacugcaacguuucaagucug
.....................acccuguagauccgGauuuguu.....................................................................	12	1	c02
.....................acccuguagauccgUauuuguu.....................................................................	6	1	c02
.....................acccuguaUauccgaauuuguu.....................................................................	17	1	c02
.....................acccuguagauccCaauuuguu.....................................................................	20	1	c02
.....................acccuguagauccgaauuGguu.....................................................................	5	1	c02
.....................acccGguagauccgaauuuguu.....................................................................	5	1	c02
.....................accAuguagauccgaauuuguu.....................................................................	78	1	c02
.....................acccuguagauccgaauuugAu.....................................................................	48	1	c02
.....................acccuguagauccgUauuuguuu....................................................................	1	1	c02
.....................acccuguagauccgaauuuguuC....................................................................	61	1	c02
.....................acccuguagauAcgaauuuguuu....................................................................	5	1	c02
.....................acccuguagauccgaauuuguuG....................................................................	47	1	c02
.....................acccuguagauccgGauuuguuu....................................................................	1	1	c02
.....................accAuguagauccgaauuuguuu....................................................................	1	1	c02
.....................acccAguagauccgaauuuguuu....................................................................	2	1	c02
.....................acccuguaCauccgaauuuguuu....................................................................	2	1	c02
.....................acccuguagauccgaauuCguuu....................................................................	1	1	c02
.....................acccuguagauccgaauuuguuA....................................................................	816	1	c02
.....................acccuguagaucAgaauuuguuu....................................................................	3	1	c02
.....................acccuguagauccgaauGuguuu....................................................................	1	1	c02
.....................acccuguagauUcgaauuuguuu....................................................................	1	1	c02
.....................acccuguagauccgaauuGguuu....................................................................	1	1	c02
.....................acccuguagauccCaauuuguuu....................................................................	1	1	c02
.....................acccugCagauccgaauuuguuu....................................................................	2	1	c02
.....................acccuguagauccgaauuuguuu....................................................................	883	0	c02
.....................acccuguagauccgaauuuguAu....................................................................	3	1	c02
.....................acccuCuagauccgaauuuguuu....................................................................	1	1	c02
.....................acccuguagCuccgaauuuguuu....................................................................	2	1	c02
.....................Gcccuguagauccgaauuuguuu....................................................................	1	1	c02
.....................Ccccuguagauccgaauuuguuu....................................................................	370	1	c02
.....................acccuguagauccgaauuuguCu....................................................................	1	1	c02
.....................acAcuguagauccgaauuuguuu....................................................................	4	1	c02
.....................acccuguagauccgaauuuguuGg...................................................................	6	1	c02
.....................acccuguagauccgaauuuguuAg...................................................................	2	1	c02
.....................acccuguagauccgaauuuguuuU...................................................................	22	1	c02
.....................acccuguagauccgaauuuguuuC...................................................................	1	1	c02
.....................acccuguagauccgaauuuguuuA...................................................................	21	1	c02
.....................acccuguagauccgaauuuguuugU..................................................................	1	1	c02
.....................acccuguagauccgaauuuguuCga..................................................................	2	1	c02
.....................acccuguagauccgaauuuguuuUa..................................................................	1	1	c02
.....................acccuguagauccgaauuuguuuAa..................................................................	3	1	c02
.....................acccuguagauccgaauuuguuuga..................................................................	3	0	c02
.....................acccuguagauccgaauuuguuuCa..................................................................	1	1	c02
.....................Ccccuguagauccgaauuuguuuga..................................................................	1	1	c02
.....................acccuguagauccgaauuuguuugaa.................................................................	3	0	c02
.....................acccuguagauccgaauuuguuuAaa.................................................................	1	1	c02
.....................acccuguagauccgaauuuguuugaaA................................................................	1	1	c02
.....................acccuguagauccgaauuuguuugaag................................................................	3	0	c02
.....................acccuguagauccgaauuuguuugaagugaggc..........................................................	1	0	c02
.....................Ccccuguagauccgaauuuguuugaagugaggc..........................................................	1	1	c02
.....................acccuguagauccgaauuuguuugaagugaggcg.........................................................	3	0	c02
.....................acccuguagauccgaauuuguuugaagugaggcga........................................................	3	0	c02
.....................Ccccuguagauccgaauuuguuugaagugaggcga........................................................	1	1	c02
......................cccuguagauccgaauu.........................................................................	17	0	c02
......................Accuguagauccgaauu.........................................................................	1	1	c02
......................cccuCuagauccgaauu.........................................................................	1	1	c02
......................ccGuguagauccgaauuug.......................................................................	1	1	c02
......................cccuguagauccgaauuug.......................................................................	35	0	c02
......................cUcuguagauccgaauuugu......................................................................	2	1	c02
......................cccuguagauccgaaAuugu......................................................................	1	1	c02
......................cccuguagaucAgaauuugu......................................................................	3	1	c02
......................cccuguagauccUaauuugu......................................................................	1	1	c02
......................Accuguagauccgaauuugu......................................................................	1	1	c02
......................cccuguagauccgaauuugu......................................................................	254	0	c02
......................cccuguagauccgaaGuugu......................................................................	1	1	c02
......................ccAuguagauccgaauuugu......................................................................	3	1	c02
......................cccCguagauccgaauuugu......................................................................	1	1	c02
......................cAcuguagauccgaauuugu......................................................................	1	1	c02
......................cccuguagauccgaauAugu......................................................................	1	1	c02
......................cccuguagauccCaauuugu......................................................................	1	1	c02
```

```
                     Mature                          Star
caauuuuagugcccuacaucuacccuguagauccgaauuuguuugaagugaggcgacaaauucgguucuagagagguuuguguggugcaugcacugcaacguuucaagucug
......................cccuguagauccgaauuuguu.....................................................................    217    0    c02
......................ccUuguagauccgaauuuguu.....................................................................    1      1    c02
......................Accuguagauccgaauuuguu.....................................................................    1      1    c02
......................cccuguagauccgaauuuguuA....................................................................    2      1    c02
......................cccuguagauccgaauuuguuC....................................................................    1      1    c02
......................cccuguagauccgaauuuguuu....................................................................    4      0    c02
.......................ccuguagCuccgaauuug.......................................................................    1      1    c02
.......................ccuguagauccgaauuug.......................................................................    15     0    c02
.......................ccuguagaucUgaauuugu......................................................................    1      1    c02
.......................ccuguagaGccgaauuugu......................................................................    1      1    c02
.......................ccuguagaucAgaauuugu......................................................................    1      1    c02
.......................ccuUuagauccgaauuugu......................................................................    2      1    c02
.......................ccuguagauccgaauuugu......................................................................    110    0    c02
.......................ccuguagCuccgaauuuguu.....................................................................    1      1    c02
.......................ccuguagauccgaauuuguu.....................................................................    102    0    c02
.......................ccuguagauccgaaAuuguu.....................................................................    1      1    c02
.......................ccuguagauccgaauuuguuu....................................................................    4      0    c02
.......................ccuguagauccgaauuuguuA....................................................................    2      1    c02
........................cuguagauccgaauuugu......................................................................    28     0    c02
........................Uuguagauccgaauuugu......................................................................    2      1    c02
........................cuCuagauccgaauuugu......................................................................    1      1    c02
........................cuUuagauccgaauuugu......................................................................    1      1    c02
........................cuguagCuccgaauuugu......................................................................    1      1    c02
........................cuguagauccgaauuuguu.....................................................................    31     0    c02
........................cuguGgauccgaauuuguu.....................................................................    1      1    c02
........................Uuguagauccgaauuuguu.....................................................................    1      1    c02
........................cuguagauccgaauuuguuA....................................................................    1      1    c02
.........................uguagauccgaauuuguu.....................................................................    23     0    c02
.........................Gguagauccgaauuuguu.....................................................................    1      1    c02
.........................uguagauccgaauuuguA.....................................................................    1      1    c02
.........................uguagauccgaauuuguuC....................................................................    1      1    c02
.........................uguagauccgaauuuguuA....................................................................    1      1    c02
..........................guagauccgaauuuguuA....................................................................    1      1    c02
.....................................................cUacaaauucgguucuagagagg....................................    1      1    c02
.....................................................Agacaaauucgguucuagagagguuu.................................    1      1    c02
.....................................................cCacaaauucgguucuagagagguuu.................................    1      1    c02
.....................................................cgCcaaauucgguucuagagagguuu.................................    2      1    c02
......................................................gacaaauucgguucuagagagg....................................    1      0    c02
......................................................gacaaauucgguucuagagaggu...................................    1      0    c02
......................................................gacaaauucgguucuagagagUu...................................    1      1    c02
......................................................gCcaaauucgguucuagagagguuu.................................    1      1    c02
.......................................................Ccaaauucgguucuagagagg....................................    1      1    c02
.......................................................Ccaaauucgguucuagagaggu...................................    1      1    c02
.......................................................acaaauucgguucuagagagguu..................................    1      0    c02
.......................................................acaaauucgguucuagagagguuu.................................    7      0    c02
........................................................caaauucgguucuagagC......................................    1      1    c02
........................................................caaauucCguucuagaga......................................    2      1    c02
........................................................caaauucgUuucuagaga......................................    1      1    c02
........................................................caaauucgguucuagaUa......................................    1      1    c02
........................................................caaauucggAucuagaga......................................    1      1    c02
........................................................caaauuAgguucuagaga......................................    4      1    c02
........................................................cGaauucgguucuagaga......................................    1      1    c02
........................................................caaauucgguucuagaCa......................................    1      1    c02
........................................................Uaaauucgguucuagaga......................................    1      1    c02
........................................................caaauucgguucuagagU......................................    1      1    c02
........................................................caaauucUguucuagaga......................................    3      1    c02
........................................................caaauucgguAcuagaga......................................    1      1    c02
........................................................caaauucgguucuagaga......................................    868    0    c02
........................................................caaauAcgguucuagaga......................................    1      1    c02
........................................................caaauucgguucuagGga......................................    1      1    c02
........................................................caaauuUgguucuagaga......................................    1      1    c02
........................................................caaauucgguucAagaga......................................    1      1    c02
........................................................caaauucgguucuagagG......................................    3      1    c02
........................................................caaauucgCuucuagaga......................................    1      1    c02
........................................................Uaaauucgguucuagagag.....................................    3      1    c02
........................................................caaauucgguuUuagagag.....................................    4      1    c02
........................................................caaaAucgguucuagagag.....................................    14     1    c02
........................................................caaauuUgguucuagagag.....................................    2      1    c02
........................................................caaauucgguucuagGgag.....................................    4      1    c02
........................................................caaCuucgguucuagagag.....................................    2      1    c02
```

```
                     Mature                             Star
caauuuuagugcccuacaucuacccuguagauccgaauuuguuugaagugaggcgacaaauucgguucuagagagguuuguguggugcaugcacugcaacguuucaagucug
........................................................caaUuucgguucuagagag.....................................   1      1   c02
........................................................caaauuAgguucuagagag.....................................   35     1   c02
........................................................caaauucCguucuagagag.....................................   11     1   c02
........................................................caaauucggGucuagagag.....................................   8      1   c02
........................................................caaauucgguucuagCgag.....................................   4      1   c02
........................................................caaauucgguucuagaCag.....................................   9      1   c02
........................................................caaauucgUuucuagagag.....................................   8      1   c02
........................................................caaauucgAuucuagagag.....................................   2      1   c02
........................................................cGaauucgguucuagagag.....................................   1      1   c02
........................................................caaauucgguucuagagaU.....................................   11     1   c02
........................................................caaauucggCucuagagag.....................................   1      1   c02
........................................................caaaGucgguucuagagag.....................................   4      1   c02
........................................................cCaauucgguucuagagag.....................................   1      1   c02
........................................................caaauucggAucuagagag.....................................   17     1   c02
........................................................caaauucgguGcuagagag.....................................   7      1   c02
........................................................caaauCcgguucuagagag.....................................   3      1   c02
........................................................caaauucgCuucuagagag.....................................   32     1   c02
........................................................caaauucgguucAagagag.....................................   6      1   c02
........................................................caaauucgguucCagagag.....................................   2      1   c02
........................................................caaauucgguuAuagagag.....................................   10     1   c02
........................................................caGauucgguucuagagag.....................................   1      1   c02
........................................................caaauucgguucuagaUag.....................................   6      1   c02
........................................................caaGuucgguucuagagag.....................................   2      1   c02
........................................................caaauucgguAcuagagag.....................................   7      1   c02
........................................................caaauucgguucuagagGg.....................................   4      1   c02
........................................................Aaaauucgguucuagagag.....................................   19     1   c02
........................................................caaauucgguucuagagaC.....................................   9      1   c02
........................................................caaauucgguucuagagUg.....................................   4      1   c02
........................................................caUauucgguucuagagag.....................................   2      1   c02
........................................................caaauucgguucuagUgag.....................................   6      1   c02
........................................................caaauucgguucuagagCg.....................................   7      1   c02
........................................................caaauucgguucuagagaA.....................................   49     1   c02
........................................................caaauucgguucuagagag.....................................   10540  0   c02
........................................................Gaaauucgguucuagagag.....................................   1      1   c02
........................................................caaauucUguucuagagag.....................................   14     1   c02
........................................................caaauucgguucuCgagag.....................................   1      1   c02
........................................................caCauucgguucuagagag.....................................   2      1   c02
........................................................caaauucAguucuagagag.....................................   4      1   c02
........................................................caaauAcgguucuagagag.....................................   7      1   c02
........................................................caaaCucgguucuagagag.....................................   1      1   c02
........................................................caaauucgguucuaUagag.....................................   8      1   c02
........................................................cUaauucgguucuagagag.....................................   1      1   c02
........................................................caaauucgguuGuagagag.....................................   1      1   c02
........................................................caaauucgguucGagagag.....................................   1      1   c02
........................................................caaauucgguucuaAagag.....................................   2      1   c02
........................................................caaauucgguucuGgagag.....................................   5      1   c02
........................................................caaauucgguucuaCagag.....................................   6      1   c02
........................................................caaauGcgguucuagagagg....................................   1      1   c02
........................................................caaauucgguucuagaCagg....................................   7      1   c02
........................................................caaUuucgguucuagagagg....................................   3      1   c02
........................................................caaauuAgguucuagagagg....................................   46     1   c02
........................................................caaauucgguucuagGgagg....................................   3      1   c02
........................................................caaauucgguucGagagagg....................................   4      1   c02
........................................................caaauucgguAcuagagagg....................................   9      1   c02
........................................................caaauucgguucuagUgagg....................................   7      1   c02
........................................................caaauucgguuUuagagagg....................................   7      1   c02
........................................................caaauucAguucuagagagg....................................   3      1   c02
........................................................caaauucgguucuagaUagg....................................   8      1   c02
........................................................caaauCcgguucuagagagg....................................   2      1   c02
........................................................Gaaauucgguucuagagagg....................................   2      1   c02
........................................................caaauuUgguucuagagagg....................................   6      1   c02
........................................................cUaauucgguucuagagagg....................................   1      1   c02
........................................................Uaaauucgguucuagagagg....................................   3      1   c02
........................................................caaauucgguucuagagaAg....................................   4      1   c02
........................................................caaauucgguCcuagagagg....................................   2      1   c02
........................................................caaauucggAucuagagagg....................................   26     1   c02
........................................................caaauucggCucuagagagg....................................   4      1   c02
........................................................caaauucCguucuagagagg....................................   20     1   c02
........................................................caaauucgguucuagagaCg....................................   15     1   c02
........................................................caaauucgAuucuagagagg....................................   3      1   c02
```

```
                     Mature                                   Star
caauuuuagugcccuacaucuacccuguagauccgaauuuguuugaagugaggcgacaaauucgguucuagagagguuugugguggugcaugcacugcaacguuucaagucug
........................................................caaauucgguucuagagagA....................................  64     1  c02
........................................................caaauucgUuucuagagagg....................................  5      1  c02
........................................................caaauucgguucuagagUgg....................................  1      1  c02
........................................................caaCuucgguucuagagagg....................................  3      1  c02
........................................................caaauAcgguucuagagagg....................................  12     1  c02
........................................................caaauucgguGcuagagagg....................................  5      1  c02
........................................................caaauucgguucuagagagC....................................  32     1  c02
........................................................caaauucgguucAagagagg....................................  13     1  c02
........................................................caaauucgguucuCgagagg....................................  1      1  c02
........................................................Aaaauucgguucuagagagg....................................  24     1  c02
........................................................caaauucgguucuagagCgg....................................  4      1  c02
........................................................caaauucgguucuagCgagg....................................  1      1  c02
........................................................caaauucgguucuaCagagg....................................  9      1  c02
........................................................caaaCucgguucuagagagg....................................  4      1  c02
........................................................caaauucUguucuagagagg....................................  18     1  c02
........................................................caaauucggGucuagagagg....................................  15     1  c02
........................................................caaauucgguucuagagGgg....................................  6      1  c02
........................................................caaauucgguucuagagaUg....................................  10     1  c02
........................................................caaGuucgguucuagagagg....................................  2      1  c02
........................................................caaauucgguucCagagagg....................................  3      1  c02
........................................................caaaGucgguucuagagagg....................................  6      1  c02
........................................................caaauucgguuAuagagagg....................................  27     1  c02
........................................................caaaAucgguucuagagagg....................................  30     1  c02
........................................................cGaauucgguucuagagagg....................................  2      1  c02
........................................................caaauucgguucuagagagU....................................  51     1  c02
........................................................caaauucgguucuaUagagg....................................  7      1  c02
........................................................caaauucgguucuUgagagg....................................  1      1  c02
........................................................caaauucgCuucuagagagg....................................  43     1  c02
........................................................caUauucgguucuagagagg....................................  4      1  c02
........................................................caaauucgguucuagagagg....................................  15592  0  c02
........................................................caaauucgguucuGgagagg....................................  5      1  c02
........................................................caaauucgguucuagagaggu...................................  9697   0  c02
........................................................caaGuucgguucuagagaggu...................................  4      1  c02
........................................................caaauucgguucuagGgaggu...................................  2      1  c02
........................................................caaauuAgguucuagagaggu...................................  25     1  c02
........................................................caaauucCguucuagagaggu...................................  13     1  c02
........................................................caaauucgguucuagUgaggu...................................  1      1  c02
........................................................caaauucgguucCagagaggu...................................  4      1  c02
........................................................cGaauucgguucuagagaggu...................................  2      1  c02
........................................................caaauucgguucuagagaggC...................................  10     1  c02
........................................................caaauucgCuucuagagaggu...................................  16     1  c02
........................................................caaaAucgguucuagagaggu...................................  18     1  c02
........................................................caaauAcgguucuagagaggu...................................  7      1  c02
........................................................caaCuucgguucuagagaggu...................................  3      1  c02
........................................................caaauucgguucuagagaggA...................................  48     1  c02
........................................................cUaauucgguucuagagaggu...................................  2      1  c02
........................................................caaauucgguucuagaCaggu...................................  3      1  c02
........................................................cCaauucgguucuagagaggu...................................  1      1  c02
........................................................caaauucgguucuagagGggu...................................  4      1  c02
........................................................caaauucgguuUuagagaggu...................................  1      1  c02
........................................................caaauucgUuucuagagaggu...................................  5      1  c02
........................................................caaaGucgguucuagagaggu...................................  5      1  c02
........................................................Uaaauucgguucuagagaggu...................................  2      1  c02
........................................................caaauucgguucuagagaCgu...................................  13     1  c02
........................................................caaauucggGucuagagaggu...................................  10     1  c02
........................................................caaauucgguucGagagaggu...................................  4      1  c02
........................................................caaauucgguucuagagagAu...................................  5      1  c02
........................................................caaauucgguucuaCagaggu...................................  10     1  c02
........................................................caCauucgguucuagagaggu...................................  1      1  c02
........................................................Gaaauucgguucuagagaggu...................................  1      1  c02
........................................................caaUuucgguucuagagaggu...................................  1      1  c02
........................................................caaauucgAuucuagagaggu...................................  1      1  c02
........................................................caaauucgguGcuagagaggu...................................  3      1  c02
........................................................caaauucgguucuagagUggu...................................  3      1  c02
........................................................caaauucgguucuaUagaggu...................................  6      1  c02
........................................................caaauucAguucuagagaggu...................................  2      1  c02
........................................................caaauucgguucuagagagCu...................................  24     1  c02
........................................................caaauucgguucuCgagaggu...................................  1      1  c02
........................................................caaauucgguAcuagagaggu...................................  9      1  c02
........................................................caaauucgguucuaAgaggu....................................  1      1  c02
```

```
                     Mature                            Star
caauuuuagugcccuacaucuacccuguagauccgaauuuguuugaagugaggcgacaaauucgguucuagagagguuugugugggugcaugcacugcaacguuucaagucug
........................................................caaauucgguucuGgagaggu...................................  3     1  c02
........................................................caaauGcgguucuagagaggu...................................  1     1  c02
........................................................caaaCucgguucuagagaggu...................................  1     1  c02
........................................................caaauucgguucuagaUaggu...................................  6     1  c02
........................................................caaauucUguucuagagaggu...................................  9     1  c02
........................................................caaauucgguucAagagaggu...................................  2     1  c02
........................................................caaauuUgguucuagagaggu...................................  4     1  c02
........................................................caaauucgguuAuagagaggu...................................  19    1  c02
........................................................caaauucgguCcuagagaggu...................................  2     1  c02
........................................................caaauuGgguucuagagaggu...................................  1     1  c02
........................................................caaauucgguucuagagaUgu...................................  8     1  c02
........................................................caaauucgguucuagagagUu...................................  8     1  c02
........................................................caaauucgguucuagagCggu...................................  2     1  c02
........................................................caaauucgguucuagaAaggu...................................  1     1  c02
........................................................Aaaauucgguucuagagaggu...................................  12    1  c02
........................................................caaauucgguucuagagaAgu...................................  1     1  c02
........................................................caaauucggAucuagagaggu...................................  12    1  c02
........................................................caaauucgguucuagagaggG...................................  30    1  c02
........................................................caaauucgguucuagCgaggu...................................  2     1  c02
........................................................caaauucgguucuagagagUuu..................................  16    1  c02
........................................................caaauucgguGcuagagagguu..................................  2     1  c02
........................................................caaauucgguucuagUgagguu..................................  4     1  c02
........................................................caaauucgguucuagaCagguu..................................  11    1  c02
........................................................caaaCucgguucuagagagguu..................................  2     1  c02
........................................................caaauucggCucuagagagguu..................................  2     1  c02
........................................................caaauucgguuGuagagagguu..................................  2     1  c02
........................................................Uaaauucgguucuagagagguu..................................  1     1  c02
........................................................caaUuucgguucuagagagguu..................................  2     1  c02
........................................................caaaAucgguucuagagagguu..................................  10    1  c02
........................................................caaCuucgguucuagagagguu..................................  2     1  c02
........................................................caaauucgguucuagagaCguu..................................  5     1  c02
........................................................caaauucgguucuagagagguA..................................  141   1  c02
........................................................caaauAcgguucuagagagguu..................................  4     1  c02
........................................................caaauucgguucuagagaggCu..................................  2     1  c02
........................................................caaauucUguucuagagagguu..................................  7     1  c02
........................................................caaauucgguucuagaUagguu..................................  9     1  c02
........................................................caaauuUgguucuagagagguu..................................  2     1  c02
........................................................caaauucgguuUuagagagguu..................................  3     1  c02
........................................................caaauucgguucuagGgagguu..................................  2     1  c02
........................................................Aaaauucgguucuagagagguu..................................  12    1  c02
........................................................caUauucgguucuagagagguu..................................  1     1  c02
........................................................caaauucggGucuagagagguu..................................  13    1  c02
........................................................caaaGucgguucuagagagguu..................................  3     1  c02
........................................................caaauucgguucuaUagagguu..................................  3     1  c02
........................................................caaauucgguucuagagaUguu..................................  1     1  c02
........................................................caaauucgguAcuagagagguu..................................  6     1  c02
........................................................caaauucgguucuagagagguC..................................  11    1  c02
........................................................caaauucgguucGagagagguu..................................  1     1  c02
........................................................caaauucgguucuaCagagguu..................................  2     1  c02
........................................................caaauucgguucuagagGgguu..................................  6     1  c02
........................................................caaauucgguuAuagagagguu..................................  6     1  c02
........................................................caaauucCguucuagagagguu..................................  4     1  c02
........................................................caaauucggAucuagagagguu..................................  12    1  c02
........................................................caaauucgguucCagagagguu..................................  2     1  c02
........................................................caaauucAguucuagagagguu..................................  1     1  c02
........................................................caaauuAgguucuagagagguu..................................  16    1  c02
........................................................caaauucgguucuagagagguG..................................  23    1  c02
........................................................caaauucgguucuagagaggAu..................................  9     1  c02
........................................................caaauucgguucuagagaggGu..................................  2     1  c02
........................................................caaauucgguucuUgagagguu..................................  5     1  c02
........................................................cCaauucgguucuagagagguu..................................  3     1  c02
........................................................caaauucgUuucuagagagguu..................................  4     1  c02
........................................................caaauucgguucuaAagagguu..................................  1     1  c02
........................................................caaauucgguucuagagagguu..................................  8082  0  c02
........................................................caaauucgCuucuagagagguu..................................  21    1  c02
........................................................caaauucgguucuagCgagguu..................................  2     1  c02
........................................................caaauucgguucuagagCgguu..................................  2     1  c02
........................................................caaauucgguucAagagagguu..................................  1     1  c02
........................................................caaauucgguucuagagagCuu..................................  16    1  c02
........................................................caaaCucgguucuagagagguuu.................................  16    1  c02
```

```
                     Mature                               Star
caauuuuagugcccuacaucuacccuguagauccgaauuuguuugaagugaggcgacaaauucgguucuagagagguuugugugguggcaugcacugcaacguuucaagucug
........................................................caaauucgguucuagagaAguuu.................................  7      1  c02
........................................................caaauucgguuUuagagagguuu.................................  22     1  c02
........................................................Gaaauucgguucuagagagguuu.................................  7      1  c02
........................................................caaauucUguucuagagagguuu.................................  50     1  c02
........................................................cUaauucgguucuagagagguuu.................................  11     1  c02
........................................................caaauAcgguucuagagagguuu.................................  45     1  c02
........................................................caaauucgguucuagagaggCuu.................................  17     1  c02
........................................................caaauucgguGcuagagagguuu.................................  16     1  c02
........................................................caaauucCguucuagagagguuu.................................  69     1  c02
........................................................caaauucgCuucuagagagguuu.................................  139    1  c02
........................................................caaauucgguucuagagagguuG.................................  25     1  c02
........................................................caaaGucgguucuagagagguuu.................................  29     1  c02
........................................................caaCuucgguucuagagagguuu.................................  13     1  c02
........................................................caaauucgguucuagagagguuu.................................  61914  0  c02
........................................................cGaauucgguucuagagagguuu.................................  13     1  c02
........................................................caaauucggAucuagagagguuu.................................  61     1  c02
........................................................caaauucgguucuagagGgguuu.................................  18     1  c02
........................................................caaauucgguucuagagagCuuu.................................  115    1  c02
........................................................caaauuUgguucuagagagguuu.................................  24     1  c02
........................................................caaauucgguucAagagagguuu.................................  28     1  c02
........................................................caGauucgguucuagagagguuu.................................  8      1  c02
........................................................caaauucgguucuaUagagguuu.................................  30     1  c02
........................................................caaUuucgguucuagagagguuu.................................  5      1  c02
........................................................caaauucgguucuagagagUuuu.................................  28     1  c02
........................................................caaauucgguuAuagagagguuu.................................  96     1  c02
........................................................caaauucgguucuagaUagguuu.................................  46     1  c02
........................................................caaauGcgguucuagagagguuu.................................  3      1  c02
........................................................caaauucgAuucuagagagguuu.................................  20     1  c02
........................................................caCauucgguucuagagagguuu.................................  3      1  c02
........................................................caaauucgguucuagagagguCu.................................  15     1  c02
........................................................caaauucgguucuGgagagguuu.................................  19     1  c02
........................................................caaauucgguucCagagagguuu.................................  19     1  c02
........................................................caaauucgguuGuagagagguuu.................................  6      1  c02
........................................................caaauucgguucuagagaUguuu.................................  47     1  c02
........................................................caaauucgguucuagagagguAu.................................  54     1  c02
........................................................caaauucgguucuagagaggAuu.................................  63     1  c02
........................................................caaauuGgguucuagagagguuu.................................  4      1  c02
........................................................Uaaauucgguucuagagagguuu.................................  32     1  c02
........................................................caaauucggGucuagagagguuu.................................  63     1  c02
........................................................caaauCcgguucuagagagguuu.................................  9      1  c02
........................................................caaauucgguucuUgagagguuu.................................  11     1  c02
........................................................caaauucgguucuagagagguuC.................................  21     1  c02
........................................................caaauucgguucuaCagagguuu.................................  32     1  c02
........................................................caaauucgguucuCgagagguuu.................................  3      1  c02
........................................................caaauucgguucuagagCgguuu.................................  24     1  c02
........................................................caaauucgguCcuagagagguuu.................................  12     1  c02
........................................................caaauucgguucuaAagagguuu.................................  7      1  c02
........................................................caaauucgguucuagagaggGuu.................................  26     1  c02
........................................................caaGuucgguucuagagagguuu.................................  7      1  c02
........................................................caaauuAgguucuagagagguuu.................................  184    1  c02
........................................................caaauucgguucuagagagguuA.................................  134    1  c02
........................................................cCaauucgguucuagagagguuu.................................  7      1  c02
........................................................caaaAucgguucuagagagguuu.................................  119    1  c02
........................................................caaauucgguucuagagagguGu.................................  17     1  c02
........................................................caaauucggCucuagagagguuu.................................  14     1  c02
........................................................Aaaauucgguucuagagagguuu.................................  75     1  c02
........................................................caaauucgguucuagaCagguuu.................................  56     1  c02
........................................................caaauucgguucGagagagguuu.................................  19     1  c02
........................................................caaauucgguucuagCgagguuu.................................  9      1  c02
........................................................caaauucgguucuagaAagguuu.................................  7      1  c02
........................................................caaauucgguucuagagaCguuu.................................  80     1  c02
........................................................caUauucgguucuagagagguuu.................................  6      1  c02
........................................................caaauucAguucuagagagguuu.................................  12     1  c02
........................................................caaauucgguucuagagAguuu..................................  11     1  c02
........................................................caaauucgUuucuagagagguuu.................................  27     1  c02
........................................................caaauucgguucuagUgagguuu.................................  16     1  c02
........................................................caaauucgguucuagGgagguuu.................................  10     1  c02
........................................................caaauucgguucuagagUgguuu.................................  11     1  c02
........................................................caaauucgguAcuagagagguuu.................................  30     1  c02
........................................................caaauuAgguucuagagagguuug................................  5      1  c02
```

```
                     Mature                   Star
caauuuuagugcccuacaucuacccuguagauccgaauuuguuugaagugaggcgacaaauucgguucuagagagguuugugguggugcaugcacugcaacguuucaagucug
........................................................caaauucgCuucuagagagguuug................................ 5     1  c02
........................................................caaauucgguucuagagaggCuug................................ 2     1  c02
........................................................caaauucgUuucuagagagguuug................................ 1     1  c02
........................................................caaauucCguucuagagagguuug................................ 4     1  c02
........................................................caaauucUguucuagagagguuug................................ 2     1  c02
........................................................caaauucgguucuagagagguuuC................................ 112   1  c02
........................................................caaauucAguucuagagagguuug................................ 1     1  c02
........................................................Aaaauucgguucuagagagguuug................................ 4     1  c02
........................................................caaauucgguucuagagagUuuug................................ 2     1  c02
........................................................caaauucgguucuagagGgguuug................................ 1     1  c02
........................................................caaauucgguucuagagagguuuA................................ 518   1  c02
........................................................caaauucgguuAuagagagguuug................................ 4     1  c02
........................................................caaauucgguAcuagagagguuug................................ 1     1  c02
........................................................caaauucgguucuagagaggGuug................................ 1     1  c02
........................................................caaauucgguucuagagagguuuU................................ 5285  1  c02
........................................................caaauucgguucuagagagguuAg................................ 2     1  c02
........................................................caaauucgguucuagaCagguuug................................ 1     1  c02
........................................................caaauucgguucuagagagguuug................................ 1504  0  c02
........................................................caaauucgguucuagagagCuuug................................ 4     1  c02
........................................................caaauuUgguucuagagagguuug................................ 1     1  c02
........................................................caaauucgguucuaAagagguuug................................ 1     1  c02
........................................................caaauucgguucuagagagguAug................................ 1     1  c02
........................................................caaauucggCucuagagagguuug................................ 1     1  c02
........................................................caaUuucgguucuagagagguuug................................ 1     1  c02
........................................................caaauucggAucuagagagguuug................................ 1     1  c02
........................................................caaauucggGucuagagagguuug................................ 1     1  c02
........................................................caaaAucgguucuagagagguuug................................ 2     1  c02
........................................................caaauucgguucuagagaCguuug................................ 2     1  c02
........................................................caaauucgguucuagCgagguuug................................ 1     1  c02
........................................................caaauucgguuGuagagagguuug................................ 1     1  c02
........................................................caUauucgguucuagagagguuug................................ 1     1  c02
........................................................caaauucgguucuagagaUguuug................................ 1     1  c02
........................................................caaauucgguucuagagagguuuAu............................... 44    1  c02
........................................................caaauucgguucuagagagguuugA............................... 66    1  c02
........................................................caaauucgguucuagagagguuugu............................... 287   0  c02
........................................................caaauucgguucuagagagguuGgu............................... 2     1  c02
........................................................caaauucgguucuagagagguuuCu............................... 17    1  c02
........................................................caaauucgguucuagagagguuugG............................... 16    1  c02
........................................................caaauucgCuucuagagagguuugu............................... 4     1  c02
........................................................Aaaauucgguucuagagagguuugu............................... 1     1  c02
........................................................caaauucgguucuagagagguuuUu............................... 527   1  c02
........................................................caaauucgguucuagagagguuugC............................... 7     1  c02
........................................................caaauuAgguucuagagagguuugu............................... 1     1  c02
........................................................caaauuUgguucuagagagguuugu............................... 1     1  c02
........................................................caaauucgguucuagagagguuuguU.............................. 21    1  c02
........................................................caaauucgguucuagagagguuugGg.............................. 1     1  c02
........................................................caaauucgguucuagagagguuuAug.............................. 1     1  c02
........................................................caaauucgguucuagagagguuuguA.............................. 3     1  c02
........................................................caaauucgguucuagagagguuugug.............................. 3     0  c02
........................................................caaauucgguucuagagagguuuUug.............................. 1     1  c02
........................................................caaauucgguucuagagagguuuguC.............................. 2     1  c02
........................................................caaauucgguucuagagagguuugCgu............................. 1     1  c02
........................................................caaauucgguucuagagagguuuguUu............................. 3     1  c02
........................................................caaauucgguucuagagagguuuguAu............................. 4     1  c02
........................................................caaauucgguucuagagagguuugGgu............................. 2     1  c02
........................................................caaauucgguucuagagagguuuguUugg........................... 1     1  c02
........................................................caaauucgguucuagagagguuugugugg........................... 1     0  c02
........................................................caaauucgguucuagagagguuugCgugg........................... 1     1  c02
.........................................................aaauucgUuucuagagag..................................... 2     1  c02
.........................................................Gaauucgguucuagagag..................................... 1     1  c02
.........................................................aaauucgguucuaUagag..................................... 2     1  c02
.........................................................aaauucgguucuagagag..................................... 288   0  c02
.........................................................aaauucgguucuagagaA..................................... 2     1  c02
.........................................................aaauucgguAcuagagag..................................... 1     1  c02
.........................................................Caauucgguucuagagag..................................... 9     1  c02
.........................................................aaauucgguucuagagaU..................................... 1     1  c02
.........................................................aaauucgguucuUgagag..................................... 1     1  c02
.........................................................aaauucggCucuagagag..................................... 1     1  c02
.........................................................aaauucgguucuagagagC.................................... 3     1  c02
.........................................................aaauucgguucuagagagU.................................... 4     1  c02
```

Mature Star

```
caauuuuagugcccuacaucuacccuguagauccgaauuuguuugaagugaggcgacaaauucgguucuagagagguuuguguggugcaugcacugcaacguuucaagucug
.........................................................aaauucgguucuGgagagg....................................    3       1       c02
.........................................................aaauAcgguucuagagagg....................................    2       1       c02
.........................................................aaauucgguucuagagagg....................................    1792    0       c02
.........................................................aaauucgguucuagaCagg....................................    2       1       c02
.........................................................aaauucCguucuagagagg....................................    2       1       c02
.........................................................aaauuAgguucuagagagg....................................    5       1       c02
.........................................................aaauucUguucuagagagg....................................    2       1       c02
.........................................................aaauucgguucuagagCgg....................................    1       1       c02
.........................................................aaauuUgguucuagagagg....................................    1       1       c02
.........................................................aaauucgguucuagGgagg....................................    1       1       c02
.........................................................aaauucgCuucuagagagg....................................    4       1       c02
.........................................................aaauucgguucuagagagA....................................    1       1       c02
.........................................................aaauucgguucuagagaCg....................................    2       1       c02
.........................................................aaauucggGucuagagagg....................................    1       1       c02
.........................................................aaauucgguucuagagaUg....................................    2       1       c02
.........................................................aaauucgguucuagCgagg....................................    1       1       c02
.........................................................aaauucgguucuUgagagg....................................    1       1       c02
.........................................................aaaAucgguucuagagagg....................................    2       1       c02
.........................................................aaauucgguucuagUgagg....................................    1       1       c02
.........................................................aGauucgguucuagagagg....................................    1       1       c02
.........................................................Gaauucgguucuagagagg....................................    1       1       c02
.........................................................aUauucgguucuagagagg....................................    1       1       c02
.........................................................Uaauucgguucuagagagg....................................    1       1       c02
.........................................................aaauucgUuucuagagagg....................................    2       1       c02
.........................................................aaauucgguGcuagagagg....................................    3       1       c02
.........................................................Caauucgguucuagagagg....................................    79      1       c02
.........................................................aaauucgguucuagagaggG...................................    3       1       c02
.........................................................aaauAcgguucuagagaggu...................................    1       1       c02
.........................................................aaaCucgguucuagagaggu...................................    1       1       c02
.........................................................aaauucgguucuagaUaggu...................................    1       1       c02
.........................................................aaauucggGucuagagaggu...................................    4       1       c02
.........................................................aaauucCguucuagagaggu...................................    1       1       c02
.........................................................aaauucUguucuagagaggu...................................    3       1       c02
.........................................................aaauucgguucuagagCggu...................................    1       1       c02
.........................................................aaaAucgguucuagagaggu...................................    1       1       c02
.........................................................Caauucgguucuagagaggu...................................    65      1       c02
.........................................................aaauuAgguucuagagaggu...................................    6       1       c02
.........................................................aaauucgCuucuagagaggu...................................    1       1       c02
.........................................................aaauucgguuGuagagaggu...................................    1       1       c02
.........................................................aaauucgguucuagagaggC...................................    1       1       c02
.........................................................aaauucgguucAagagaggu...................................    1       1       c02
.........................................................aaauuUgguucuagagaggu...................................    1       1       c02
.........................................................aaauucgguGcuagagaggu...................................    1       1       c02
.........................................................aaauucgguucGagagaggu...................................    1       1       c02
.........................................................aaauucgguucuaCagaggu...................................    2       1       c02
.........................................................aaauucgguucuagagaggu...................................    1428    0       c02
.........................................................aaauucgguucuagagagCu...................................    3       1       c02
.........................................................aaauucgguuUuagagaggu...................................    1       1       c02
.........................................................aaauucgguuAuagagaggu...................................    3       1       c02
.........................................................aaauucgguucuagaCaggu...................................    1       1       c02
.........................................................aaauucgguucuagagaggA...................................    5       1       c02
.........................................................aaauucgguucuagagaCgu...................................    1       1       c02
.........................................................aaauucggCucuagagaggu...................................    1       1       c02
.........................................................aaauucgguucuagagaUgu...................................    1       1       c02
.........................................................aaauucgguucuagaCagguu..................................    3       1       c02
.........................................................aaauucgguAcuagagagguu..................................    2       1       c02
.........................................................aaauucgguucuagagagguC..................................    3       1       c02
.........................................................aaauucgguucuagagaCguu..................................    3       1       c02
.........................................................aaauucgguuUuagagagguu..................................    1       1       c02
.........................................................aaauucUguucuagagagguu..................................    7       1       c02
.........................................................aaauucgguucuagagaggCu..................................    1       1       c02
.........................................................aaauucgguucuagagagCuu..................................    5       1       c02
.........................................................aaauucgguucAagagagguu..................................    1       1       c02
.........................................................Caauucgguucuagagagguu..................................    111     1       c02
.........................................................aaauucgguucuagagagUuu..................................    2       1       c02
.........................................................aaauucCguucuagagagguu..................................    1       1       c02
.........................................................aaauucgguucuagUgagguu..................................    1       1       c02
.........................................................aaauucgguucuaUagagguu..................................    3       1       c02
.........................................................aaauucgguucGagagagguu..................................    1       1       c02
.........................................................aaaAucgguucuagagagguu..................................    5       1       c02
```

```
                    Mature                       Star
caauuuuagugcccuacaucuacccuguagauccgaauuuguuugaagugaggcgacaaauucgguucuagagagguuuguguggugcaugcacugcaacguuucaagucug
.........................................................aaauucgguucuagagCgguu..................................   1       1   c02
.........................................................aaauucgguucuagagaggGu..................................   2       1   c02
.........................................................aaauucgAuucuagagagguu..................................   1       1   c02
.........................................................aaauucgguucuGgagagguu..................................   1       1   c02
.........................................................aaauucgguucuagagaUguu..................................   1       1   c02
.........................................................aaauucggAucuagagagguu..................................   1       1   c02
.........................................................aaauucgguucuagagagguG..................................   5       1   c02
.........................................................aGauucgguucuagagagguu..................................   1       1   c02
.........................................................aaCuucgguucuagagagguu..................................   1       1   c02
.........................................................aaauucgguGcuagagagguu..................................   2       1   c02
.........................................................aaauucgguuAuagagagguu..................................   3       1   c02
.........................................................aaauucgguucuagagagguA..................................   10      1   c02
.........................................................aaauucgguucuagagaggAu..................................   4       1   c02
.........................................................aaauucgguucuaCagagguu..................................   2       1   c02
.........................................................aaauucgguucuagaUagguu..................................   3       1   c02
.........................................................aaauucgguucuUgagagguu..................................   2       1   c02
.........................................................aaauucgUuucuagagagguu..................................   1       1   c02
.........................................................aaauucgguucuagCgagguu..................................   2       1   c02
.........................................................aaauucgguucuagagagguu..................................   3140    0   c02
.........................................................aaauuAgguucuagagagguu..................................   13      1   c02
.........................................................aaauucgCuucuagagagguu..................................   9       1   c02
.........................................................aaauucgguCcuagagagguu..................................   1       1   c02
.........................................................aaGuucgguucuagagagguu..................................   1       1   c02
.........................................................aaauucggCucuagagagguu..................................   6       1   c02
.........................................................aaauuUgguucuagagagguu..................................   1       1   c02
.........................................................aaauucggGucuagagagguu..................................   4       1   c02
.........................................................aaauucgguucuagagagguuu.................................   16732   0   c02
.........................................................aaauucgguucuagagaggAuu.................................   15      1   c02
.........................................................aaauucgAuucuagagagguuu.................................   3       1   c02
.........................................................aaauCcgguucuagagagguuu.................................   2       1   c02
.........................................................aaauucgguucuagagagguuC.................................   9       1   c02
.........................................................aaauucgguuGuagagagguuu.................................   4       1   c02
.........................................................aaauuUgguucuagagagguuu.................................   6       1   c02
.........................................................aaauucgguucuagagUgguuu.................................   3       1   c02
.........................................................aaauucgguucuagagCgguuu.................................   6       1   c02
.........................................................aaauucggAucuagagagguuu.................................   15      1   c02
.........................................................aaaGucgguucuagagagguuu.................................   3       1   c02
.........................................................aaauucgCuucuagagagguuu.................................   32      1   c02
.........................................................Uaauucgguucuagagagguuu.................................   4       1   c02
.........................................................aaauucgguucuUgagagguuu.................................   5       1   c02
.........................................................aaauucgguAcuagagagguuu.................................   8       1   c02
.........................................................aaauucgguucCagagagguuu.................................   8       1   c02
.........................................................aaauucgguucAagagagguuu.................................   3       1   c02
.........................................................aUauucgguucuagagagguuu.................................   4       1   c02
.........................................................aaauuGgguucuagagagguuu.................................   1       1   c02
.........................................................aaauucgguucuaCagagguuu.................................   13      1   c02
.........................................................aaauucgguucuagagagCuuu.................................   38      1   c02
.........................................................aaauucgguGcuagagagguuu.................................   8       1   c02
.........................................................aaauucgguucuagCgagguuu.................................   5       1   c02
.........................................................aaCuucgguucuagagagguuu.................................   1       1   c02
.........................................................aaauucgguucuagaUagguuu.................................   18      1   c02
.........................................................aaauucgguucuagagaUguuu.................................   5       1   c02
.........................................................aaauucAguucuagagagguuu.................................   1       1   c02
.........................................................aaauucgguucuagagaCguuu.................................   21      1   c02
.........................................................aaauucCguucuagagagguuu.................................   9       1   c02
.........................................................aaauucggCucuagagagguuu.................................   2       1   c02
.........................................................aaaCucgguucuagagagguuu.................................   6       1   c02
.........................................................aaauucUguucuagagagguuu.................................   18      1   c02
.........................................................aaauucggGucuagagagguuu.................................   20      1   c02
.........................................................aaauucgguucuagagagguAu.................................   19      1   c02
.........................................................aaaAucgguucuagagagguuu.................................   7       1   c02
.........................................................aaGuucgguucuagagagguuu.................................   3       1   c02
.........................................................aaauucgguucuGgagagguuu.................................   8       1   c02
.........................................................aaauucgguucuagagagAuuu.................................   2       1   c02
.........................................................aaauucgguucuagagaggCuu.................................   4       1   c02
.........................................................aaauucgguucuagUgagguuu.................................   5       1   c02
.........................................................aaauucgguucuagagagguuA.................................   35      1   c02
.........................................................aaauGcgguucuagagagguuu.................................   1       1   c02
.........................................................aaauucgguucuaUagagguuu.................................   8       1   c02
.........................................................aaauucgguuAuagagagguuu.................................   21      1   c02
```

```
                    Mature                    Star
caauuuuagugcccuacaucuacccuguagauccgaauuuguuugaagugaggcgacaaauucgguucuagagagguuugugugguggcaugcacugcaacguuucaagucug
.........................................................aaauucgguucuagagagguGu.................................    7     1    c02
.........................................................aaauucgguucuagagagUuuu.................................    13    1    c02
.........................................................aaauucgguucuagaCagguuu.................................    12    1    c02
.........................................................aaauucgUuucuagagagguuu.................................    3     1    c02
.........................................................aaauAcgguucuagagagguuu.................................    14    1    c02
.........................................................Gaauucgguucuagagagguuu.................................    3     1    c02
.........................................................Caauucgguucuagagagguuu.................................    607   1    c02
.........................................................aaauucgguucGagagagguuu.................................    2     1    c02
.........................................................aaauucgguucuagagaAguuu.................................    1     1    c02
.........................................................aaauucgguuUuagagagguuu.................................    5     1    c02
.........................................................aaauucgguucuagGgagguuu.................................    3     1    c02
.........................................................aGauucgguucuagagagguuu.................................    5     1    c02
.........................................................aaauucgguucuagagaggGuu.................................    7     1    c02
.........................................................aaauucgguucuagagagguuG.................................    8     1    c02
.........................................................aaUuucgguucuagagagguuu.................................    2     1    c02
.........................................................aaauucgguucuagagagguCu.................................    4     1    c02
.........................................................aaauucgguucuagagGgguuu.................................    3     1    c02
.........................................................aaauuAgguucuagagagguuu.................................    61    1    c02
.........................................................aaauucgguucuagagagguuAg................................    3     1    c02
.........................................................aaauAcgguucuagagagguuug................................    3     1    c02
.........................................................aaauucgguucCagagagguuug................................    4     1    c02
.........................................................aaauucgguucuagGgagguuug................................    2     1    c02
.........................................................aaauucgUuucuagagagguuug................................    1     1    c02
.........................................................aaauucgguucuagUgagguuug................................    1     1    c02
.........................................................aaauucgguuGuagagagguuug................................    2     1    c02
.........................................................aaauucgguucuagagagguAug................................    5     1    c02
.........................................................aaauucgguuAuagagagguuug................................    7     1    c02
.........................................................aaauucgguucuagCgagguuug................................    1     1    c02
.........................................................aaauucgguucuagagagUuuug................................    11    1    c02
.........................................................aaGuucgguucuagagagguuug................................    1     1    c02
.........................................................aaUuucgguucuagagagguuug................................    4     1    c02
.........................................................aaauucgguucuagagagguuuC................................    87    1    c02
.........................................................aaauucgguucuGgagagguuug................................    1     1    c02
.........................................................aaauucgguucuaAagagguuug................................    2     1    c02
.........................................................Caauucgguucuagagagguuug................................    249   1    c02
.........................................................aaauuAgguucuagagagguuug................................    13    1    c02
.........................................................aaauucgguucuagagaCguuug................................    6     1    c02
.........................................................aaauucgguCcuagagagguuug................................    1     1    c02
.........................................................aaauucgCuucuagagagguuug................................    4     1    c02
.........................................................aaauucgguucuagagCgguuug................................    5     1    c02
.........................................................aaauucgguucuagagUgguuug................................    2     1    c02
.........................................................aaauucgguucuagagagguuuU................................    4474  1    c02
.........................................................aaauuUgguucuagagagguuug................................    1     1    c02
.........................................................aaauucggCucuagagagguuug................................    2     1    c02
.........................................................aaauucCguucuagagagguuug................................    6     1    c02
.........................................................aaaCucgguucuagagagguuug................................    2     1    c02
.........................................................aaauucgguucuagagaggAuug................................    8     1    c02
.........................................................aaauucgguucuagagagAuuug................................    1     1    c02
.........................................................aaauucUguucuagagagguuug................................    5     1    c02
.........................................................aaauucgguucuagaAagguuug................................    1     1    c02
.........................................................aaauucgguucuagagagguuCg................................    2     1    c02
.........................................................aaauucgguucuagaCagguuug................................    8     1    c02
.........................................................aaauucAguucuagagagguuug................................    2     1    c02
.........................................................aaaGucgguucuagagagguuug................................    1     1    c02
.........................................................aaauucgguucuagagaUguuug................................    6     1    c02
.........................................................Gaauucgguucuagagagguuug................................    3     1    c02
.........................................................aaauucgguucuaCagagguuug................................    3     1    c02
.........................................................aaauucgguucuaUagagguuug................................    1     1    c02
.........................................................aaauucgguAcuagagagguuug................................    4     1    c02
.........................................................aaauucgguucuUgagagguuug................................    1     1    c02
.........................................................aaauucgguuUuagagagguuug................................    3     1    c02
.........................................................aaauucgguucuagagagguuug................................    6534  0    c02
.........................................................aaauucgguucGagagagguuug................................    1     1    c02
.........................................................aaauCcgguucuagagagguuug................................    2     1    c02
.........................................................aaauucgguucAagagagguuug................................    3     1    c02
.........................................................aaauucggGucuagagagguuug................................    16    1    c02
.........................................................aaauucgguucuagagCuuug................................    11    1    c02
.........................................................aaauucgguucuagaUagguuug................................    4     1    c02
.........................................................aaauucgguucuagagagguuuA................................    354   1    c02
.........................................................aaauucgguucuagagGgguuug................................    3     1    c02
```

Mature **Star**

caauuuuagugcccuacaucuacccuguagauccgaauuuguuugaagugaggcgacaaauucgguucuagagagguuugugugguggugcaugcacugcaacguuucaagucug

....aaCuucgguucuagagagguuug................................	1	1	c02
....aaauucgguucuagagagguCug................................	1	1	c02
....aUauucgguucuagagagguuug................................	1	1	c02
....aaauucgguucuagagaggGuug................................	1	1	c02
....aaauucggAucuagagagguuug................................	2	1	c02
....aaaAucgguucuagagagguuug................................	2	1	c02
....aaauucgguucuagagagguuGg................................	1	1	c02
....aaauucgguucuagagagguuuAu...............................	29	1	c02
....aaauucgguucuagagaggGuugu...............................	1	1	c02
....aaauucgguucuagagagguuugA...............................	152	1	c02
....aaauucgguucuaUagagguuugu...............................	1	1	c02
....aaauucUguucuagagagguuugu...............................	2	1	c02
....aaauucgguucuagagagguuugC...............................	11	1	c02
....aaauucgguucuagagagguuugu...............................	646	0	c02
....aaauucgCuucuagagagguuugu...............................	2	1	c02
....aaauucggGucuagagagguuugu...............................	1	1	c02
....aaauuAgguucuagagagguuugu...............................	3	1	c02
....aaauucgguucuagagagguuugG...............................	28	1	c02
....aaauucCguucuagagagguuugu...............................	1	1	c02
....Caauucgguucuagagagguuugu...............................	27	1	c02
....aaauucgguucuagCgagguuugu...............................	1	1	c02
....aaauucgguucuagagagguuAgu...............................	1	1	c02
....aaauucgguucuagagagguuuUu...............................	305	1	c02
....aaauucgguucuagagagCuuugu...............................	1	1	c02
....aaauucgguucuagagagguuuCu...............................	18	1	c02
....aaauucgguucuagagaggAuugu...............................	1	1	c02
....aaauucgguucuagagagguuuguC..............................	3	1	c02
....aaauucgguucuagagagguuuguA..............................	24	1	c02
....aaauucgguucuagagagguuuAug..............................	1	1	c02
....aaauucgguucuagagagguuuguU..............................	22	1	c02
....aaauucgguucuagagagguuuUug..............................	1	1	c02
....aaauucgguucuagagagguuugAg..............................	1	1	c02
....aaauucgguucuagagagguuugug..............................	1	0	c02
....aaauucgguucuagagagguuuguAu.............................	5	1	c02
....aaauucgguucuagagagguuuguUu.............................	3	1	c02
....aauucgguucuagagagg....................................	18	0	c02
....Cauucgguucuagagaggu...................................	1	1	c02
....aauucgguucuagagaggu...................................	14	0	c02
....aauucgguucuagagagguu..................................	14	0	c02
....aauucgguucuagagagCuuu.................................	1	1	c02
....aauucgguucuagagagguuu.................................	98	0	c02
....Cauucgguucuagagagguuu.................................	7	1	c02
....aUuucgguucuagagagguuu.................................	1	1	c02
....aauucgguAcuagagagguuu.................................	1	1	c02
....aauucgguucuagagagguuuU................................	14	1	c02
....aauucgguucuagagagguuug................................	21	0	c02
....aauucgguucuagagagguuuA................................	2	1	c02
....Cauucgguucuagagagguuugu...............................	1	1	c02
....aauucgguucuagagagguuugC...............................	1	1	c02
....aauucgguucuagagagguuugA...............................	3	1	c02
....aauucgguucuagagagguuuUu...............................	11	1	c02
....aauucgguucuagagagguuugu...............................	10	0	c02
....aauucgguucuagagagguuuCu...............................	2	1	c02
....aauucgguucuagagagguuuAu...............................	1	1	c02
....auucgguucAagagaggu...................................	1	1	c02
....auucggAucuagagaggu...................................	1	1	c02
....auucgguucuagagaggA...................................	3	1	c02
....auucgguucuagagaggG...................................	1	1	c02
....auucgguuAuagagaggu...................................	1	1	c02
....auucgguucuagUgaggu...................................	1	1	c02
....auucgguucuagagaggC...................................	3	1	c02
....auucgguucuagagagCu...................................	2	1	c02
....auucgguucuGgagaggu...................................	2	1	c02
....auucgguucuagaCaggu...................................	1	1	c02
....auucgguucuagagaUgu...................................	1	1	c02
....auuAgguucuagagaggu...................................	2	1	c02
....Guucgguucuagagaggu...................................	1	1	c02
....auucgguucuagagaAgu...................................	1	1	c02
....auucgguucuagagaggu...................................	675	0	c02
....auucggGucuagagaggu...................................	1	1	c02

Mature **Star**

caauuuuagugcccuacaucuacccuguagauccgaauuuguuugaagugaggcgacaaauucgguucuagagagguuuguguggugcaugcacugcaacguuucaagucug			
....auucgguucuagGgaggu...................................	1	1	c02
....Cuucgguucuagagaggu...................................	82	1	c02
....auucgguucuaUagaggu...................................	1	1	c02
....auucgguucuagagagUuu..................................	1	1	c02
....auucgguucuGgagagguu..................................	1	1	c02
....auucgguucuagagagguA..................................	11	1	c02
....auucgCuucuagagagguu..................................	1	1	c02
....auucgguucuagagagguu..................................	788	0	c02
....auuAgguucuagagagguu..................................	3	1	c02
....Cuucgguucuagagagguu..................................	67	1	c02
....auucgguAcuagagagguu..................................	1	1	c02
....auucgguucuagCgagguu..................................	1	1	c02
....auuGgguucuagagagguu..................................	1	1	c02
....auucgguucuagagGgguu..................................	1	1	c02
....auucCguucuagagagguu..................................	2	1	c02
....auucgguucuagagagCuu..................................	3	1	c02
....auucgguucuagagaggAu..................................	1	1	c02
....auucgguucCagagagguu..................................	1	1	c02
....auucgguucuagagagguC..................................	2	1	c02
....auucgguucuagGgagguuu.................................	2	1	c02
....Uuucgguucuagagagguuu.................................	2	1	c02
....auucgguucuagagagguAu.................................	7	1	c02
....auucgguucuagagagUuuu.................................	3	1	c02
....auucgguuAuagagagguuu.................................	5	1	c02
....auucggAucuagagagguuu.................................	6	1	c02
....auucgguucuagagaAguuu.................................	2	1	c02
....auucgCuucuagagagguuu.................................	5	1	c02
....auucUguucuagagagguuu.................................	1	1	c02
....auucgguucuagagagguuu.................................	4955	0	c02
....auucgAuucuagagagguuu.................................	1	1	c02
....auucgguucuagagaCguuu.................................	6	1	c02
....auucCguucuagagagguuu.................................	4	1	c02
....auucgguAcuagagagguuu.................................	4	1	c02
....auucgguucAagagagguuu.................................	1	1	c02
....auucgguucuagagGgguuu.................................	2	1	c02
....auucgguucuagaUagguuu.................................	3	1	c02
....aAucgguucuagagagguuu.................................	6	1	c02
....auuAgguucuagagagguuu.................................	13	1	c02
....auucgguucuagaCagguuu.................................	5	1	c02
....auucgguucuagagagAuuu.................................	1	1	c02
....auucgguucuagaAagguuu.................................	1	1	c02
....auucgguucuagagagCuuu.................................	8	1	c02
....auucgguucuagagagguuA.................................	9	1	c02
....auucgguucuaUagagguuu.................................	1	1	c02
....auucgguucuagagaUguuu.................................	7	1	c02
....auucgguucuagUgagguuu.................................	2	1	c02
....aCucgguucuagagagguuu.................................	2	1	c02
....auucgguucuCgagagguuu.................................	1	1	c02
....auCcgguucuagagagguuu.................................	1	1	c02
....auAcgguucuagagagguuu.................................	3	1	c02
....auucgguucuagagaggCuu.................................	1	1	c02
....auuUgguucuagagagguuu.................................	1	1	c02
....auucgguucuGgagagguuu.................................	2	1	c02
....auucgguucuagagagguuG.................................	2	1	c02
....auucgguucuaCagagguuu.................................	6	1	c02
....auucgguuUuagagagguuu.................................	1	1	c02
....auucgguuGuagagagguuu.................................	1	1	c02
....auucggGucuagagagguuu.................................	1	1	c02
....Guucgguucuagagagguuu.................................	2	1	c02
....Cuucgguucuagagagguuu.................................	529	1	c02
....auucgguucGagagagguuug................................	1	1	c02
....auucgguucuagagagguuuC................................	6	1	c02
....auucgguucuagagagguuug................................	226	0	c02
....aGucgguucuagagagguuug................................	1	1	c02
....auucgguuAuagagagguuug................................	1	1	c02
....auucgguucAagagagguuug................................	1	1	c02
....auucgguucuagagagguuuU................................	480	1	c02
....auucgguucuagagagguuuA................................	59	1	c02
....Uuucgguucuagagagguuug................................	1	1	c02
....auucgCuucuagagagguuug................................	1	1	c02

```
                     Mature                             Star
caauuuuagugcccuacaucuacccuguagauccgaauuuguuugaagugaggcgacaaauucgguucuagagagguuuguguggugcaugcacugcaacguuucaagucug
...........................................................auucgguucuagagagUuuug................................  1    1  c02
...........................................................Cuucgguucuagagagguuug................................  26   1  c02
...........................................................auucgguucuagCgagguuug................................  1    1  c02
...........................................................auucgguucuagagagguuugu...............................  30   0  c02
...........................................................auucgguucuagagagguuugG...............................  1    1  c02
...........................................................Cuucgguucuagagagguuugu...............................  2    1  c02
...........................................................auucgguucuagagagguuuUu...............................  39   1  c02
...........................................................auucgguucuagagagguuugA...............................  10   1  c02
...........................................................auucggAucuagagagguuugu...............................  1    1  c02
...........................................................auucgguucuagagagguuugC...............................  1    1  c02
...........................................................auucgguucuagagagguuuCu...............................  3    1  c02
...........................................................auucgguucuagagagguuuAu...............................  2    1  c02
...........................................................auucgguucuagagaCguuugu...............................  1    1  c02
...........................................................auucgguucuagagagguuuguCu.............................  1    1  c02
...........................................................auucgguucuagagagguuugGgu.............................  1    1  c02
............................................................uucgguuAuagagagguu..................................  1    1  c02
............................................................Gucgguucuagagagguu..................................  6    1  c02
............................................................uucgguucuagagagguA..................................  2    1  c02
............................................................uucgguucuagagagguu..................................  68   0  c02
............................................................Aucgguucuagagagguu..................................  1    1  c02
............................................................uucgguucuagagUgguuu.................................  1    1  c02
............................................................uucgguucuagagagguuu.................................  380  0  c02
............................................................uucgguucuGgagagguuu.................................  1    1  c02
............................................................uuAgguucuagagagguuu.................................  1    1  c02
............................................................uucgguuAuagagagguuu.................................  1    1  c02
............................................................uucgguucuagagagCuuu.................................  1    1  c02
............................................................uCcgguucuagagagguuu.................................  1    1  c02
............................................................Gucgguucuagagagguuu.................................  42   1  c02
............................................................uucggAucuagagagguuu.................................  1    1  c02
............................................................uuGgguucuagagagguuu.................................  1    1  c02
............................................................uucgguucAagagagguuu.................................  1    1  c02
............................................................uucgCuucuagagagguuug................................  1    1  c02
............................................................Gucgguucuagagagguuug................................  18   1  c02
............................................................uucgguucuagagagguuuU................................  83   1  c02
............................................................uucgguucuagagagguuug................................  90   0  c02
............................................................uucgguucuagagagguuuA................................  12   1  c02
............................................................uucgguucuagagagguuuC................................  1    1  c02
............................................................uucgguucuagagagguuugA...............................  1    1  c02
............................................................uucgguucuagagagguuuAu...............................  3    1  c02
............................................................uucgguucuagagagguuugu...............................  7    0  c02
............................................................Gucgguucuagagagguuugu...............................  1    1  c02
............................................................uucgguucuagagagguuuUu...............................  3    1  c02
............................................................uucgguucuagagagguuuguA..............................  2    1  c02
.............................................................Gcgguucuagagagguuu.................................  4    1  c02
.............................................................ucCguucuagagagguuu.................................  1    1  c02
.............................................................ucgguucuagagagguuu.................................  38   0  c02
.............................................................ucgguucuagagagguuuA................................  2    1  c02
.............................................................ucgguucuagagagguuuU................................  5    1  c02
.............................................................ucgguucuagagagguuuC................................  1    1  c02
.............................................................ucgguucuagagagguuug................................  3    0  c02
.............................................................ucgguucuagagagCuuug................................  1    1  c02
..............................................................cgguucuagagagguuuU................................  6    1  c02
..............................................................cgguucuagagagguuug................................  3    0  c02

.................aucuacccuguagauccgaauuugu......................................................................  1    0  c15
..................ucuacccuguagauccgaauuu........................................................................  2    0  c15
...................cuacccuguagauccgaauuugu......................................................................  2    0  c15
...................cCacccuguagauccgaauuuguu.....................................................................  1    1  c15
....................uacccuguagauccgaau..........................................................................  177  0  c15
....................uacAcuguagauccgaau..........................................................................  1    1  c15
....................uacccuguagauccgaauC.........................................................................  1    1  c15
....................uacccugAagauccgaauu.........................................................................  1    1  c15
....................uacccuguagauccgaaAu.........................................................................  1    1  c15
....................uGcccuguagauccgaauu.........................................................................  1    1  c15
....................uacccuguagauccgaauu.........................................................................  473  0  c15
....................uacccuguagauccgaauuu........................................................................  236  0  c15
....................uacccuguagauccgaauuA........................................................................  1    1  c15
....................uacUcuguagauccgaauuu........................................................................  1    1  c15
....................uacccAguagauccgaauuu........................................................................  1    1  c15
....................uGcccuguagauccgaauuug.......................................................................  2    1  c15
```

```
                     Mature                 Star
caauuuuagugcccuacaucuacccuguagauccgaauuuguuugaagugaggcgacaaauucgguucuagagagguuuguguggugcaugcacugcaacguuucaagucug
....................uaUccuguagauccgaauuug.......................................................................  1       1    c15
....................uacccuguagauccgaauuuC.......................................................................  2       1    c15
....................uacccuguagauccgCauuug.......................................................................  2       1    c15
....................uacccuguagauccgaCuuug.......................................................................  2       1    c15
....................uacccugGagauccgaauuug.......................................................................  2       1    c15
....................uacccuguagauccgaaCuug.......................................................................  1       1    c15
....................Gacccuguagauccgaauuug.......................................................................  1       1    c15
....................uacccuguagauccgaUuuug.......................................................................  1       1    c15
....................Aacccuguagauccgaauuug.......................................................................  2       1    c15
....................uacccuguaUauccgaauuug.......................................................................  2       1    c15
....................uacccugCagauccgaauuug.......................................................................  2       1    c15
....................uacccuguagauccgaauuuA.......................................................................  70      1    c15
....................uacccuguagauGcgaauuug.......................................................................  1       1    c15
....................uacccuguagaCccgaauuug.......................................................................  1       1    c15
....................uacccuguagauUcgaauuug.......................................................................  2       1    c15
....................uacccuguagaucUgaauuug.......................................................................  2       1    c15
....................uacccCguagauccgaauuug.......................................................................  1       1    c15
....................uacccuguagauccgaauuug.......................................................................  2779    0    c15
....................uacccuguagauccgaauuuU.......................................................................  6       1    c15
....................uacccuguagauccgaauuCg.......................................................................  1       1    c15
....................uacUcuguagauccgaauuug.......................................................................  2       1    c15
....................uacccuCuagauccgaauuug.......................................................................  1       1    c15
....................uacccuguagauccgaauuGg.......................................................................  1       1    c15
....................uacccuguagauccgaGuuug.......................................................................  2       1    c15
....................uacccAguagauccgaauuug.......................................................................  1       1    c15
....................uacccuguagauccAaauuug.......................................................................  1       1    c15
....................uacccuguagaucAgaauuug.......................................................................  2       1    c15
....................uaAccuguagauccgaauuug.......................................................................  2       1    c15
....................Cacccuguagauccgaauuug.......................................................................  3       1    c15
....................uacccuguagauccgaaCuugu......................................................................  9       1    c15
....................uacccuguagaucAgaauuugu......................................................................  48      1    c15
....................uacccuguUgauccgaauuugu......................................................................  13      1    c15
....................uacccuguagauccCaauuugu......................................................................  6       1    c15
....................uaccAuguagauccgaauuugu......................................................................  38      1    c15
....................uacccuguagauccgaauuGgu......................................................................  100     1    c15
....................uacccugGagauccgaauuugu......................................................................  70      1    c15
....................uacccuguagUuccgaauuugu......................................................................  3       1    c15
....................uacccuguagauccgaaGuugu......................................................................  6       1    c15
....................uacccuguagauGcgaauuugu......................................................................  48      1    c15
....................uacccuguagauccgaauuCgu......................................................................  40      1    c15
....................uacccuUuagauccgaauuugu......................................................................  13      1    c15
....................uacccuguagauccgaUuuugu......................................................................  46      1    c15
....................uacccuguagauccgaaAuugu......................................................................  12      1    c15
....................Cacccuguagauccgaauuugu......................................................................  62      1    c15
....................uacccuguagauccgaauCugu......................................................................  18      1    c15
....................uGcccuguagauccgaauuugu......................................................................  149     1    c15
....................uacccuguagaCccgaauuugu......................................................................  21      1    c15
....................Gacccuguagauccgaauuugu......................................................................  11      1    c15
....................uacccCguagauccgaauuugu......................................................................  65      1    c15
....................uacccuguagauccgaauuugu......................................................................  149644  0    c15
....................uacccuguCgauccgaauuugu......................................................................  2       1    c15
....................uaAccuguagauccgaauuugu......................................................................  11      1    c15
....................uacccugAagauccgaauuugu......................................................................  44      1    c15
....................uacccuguagGuccgaauuugu......................................................................  10      1    c15
....................uacccuguagCuccgaauuugu......................................................................  4       1    c15
....................uacccuguagauccgUauuugu......................................................................  46      1    c15
....................uacccuguGgauccgaauuugu......................................................................  35      1    c15
....................uacccuguagauccgCauuugu......................................................................  20      1    c15
....................uaGccuguagauccgaauuugu......................................................................  9       1    c15
....................uacccuguagauccgaauuuUu......................................................................  16      1    c15
....................Aacccuguagauccgaauuugu......................................................................  90      1    c15
....................uacccuguagaAccgaauuugu......................................................................  26      1    c15
....................uacccuguagaucGgaauuugu......................................................................  5       1    c15
....................uaUccuguagauccgaauuugu......................................................................  54      1    c15
....................uacccuguagauccAaauuugu......................................................................  39      1    c15
....................uacccuguagauccgaauuugA......................................................................  342     1    c15
....................uacccuguagauccgaCuuugu......................................................................  30      1    c15
....................uacccuCuagauccgaauuugu......................................................................  10      1    c15
....................uacccuguagauccUaauuugu......................................................................  8       1    c15
....................uacccuguagauccgaauuuAu......................................................................  66      1    c15
```

```
                     Mature                      Star
caauuuuagugcccuacaucuacccuguagauccgaauuuguuugaagugaggcgacaaauucgguucuagagagguuuguguggugcaugcacugcaacguuucaagucug
....................uacccuguagauccgaauGugu......................................................................    6      1    c15
....................uacccuguagauAcgaauuugu......................................................................    30     1    c15
....................uacccGguagauccgaauuugu......................................................................    65     1    c15
....................uaccUuguagauccgaauuugu......................................................................    48     1    c15
....................uacccuguaCauccgaauuugu......................................................................    3      1    c15
....................uacccuguagauccgaauuugG......................................................................    68     1    c15
....................uCcccuguagauccgaauuugu......................................................................    4      1    c15
....................uacAcuguagauccgaauuugu......................................................................    26     1    c15
....................uUcccuguagauccgaauuugu......................................................................    37     1    c15
....................uacccuguagauccgaauuugC......................................................................    682    1    c15
....................uacccuguagaucUgaauuugu......................................................................    67     1    c15
....................uacccuguagauccgaauuAgu......................................................................    52     1    c15
....................uacccuguaAauccgaauuugu......................................................................    19     1    c15
....................uacccuguagaGccgaauuugu......................................................................    28     1    c15
....................uacccuguagauccgaauuuCu......................................................................    16     1    c15
....................uacccuAuagauccgaauuugu......................................................................    48     1    c15
....................uacccuguagauUcgaauuugu......................................................................    66     1    c15
....................uacccAguagauccgaauuugu......................................................................    66     1    c15
....................uacGcuguagauccgaauuugu......................................................................    9      1    c15
....................uaccGuguagauccgaauuugu......................................................................    4      1    c15
....................uacccuguagauccgaauAugu......................................................................    21     1    c15
....................uacccuguagauccgGauuugu......................................................................    24     1    c15
....................uacUcuguagauccgaauuugu......................................................................    44     1    c15
....................uacccugCagauccgaauuugu......................................................................    39     1    c15
....................uacccuguagauccgaGuuugu......................................................................    53     1    c15
....................uacccuguaUauccgaauuugu......................................................................    23     1    c15
....................uaGccuguagauccgaauuuguu.....................................................................    1      1    c15
....................uacccuguagaucUgaauuuguu.....................................................................    2      1    c15
....................uacccuguUgauccgaauuuguu.....................................................................    1      1    c15
....................Cacccuguagauccgaauuuguu.....................................................................    17     1    c15
....................uacccuguagauccgaauuuguu.....................................................................    2107   0    c15
....................uacccuguagauccgGauuuguu.....................................................................    1      1    c15
....................uacccuUuagauccgaauuuguu.....................................................................    2      1    c15
....................uGcccuguagauccgaauuuguu.....................................................................    3      1    c15
....................Aacccuguagauccgaauuuguu.....................................................................    2      1    c15
....................uacccuguagauccgaauuuUuu.....................................................................    5      1    c15
....................uacccuguagauAcgaauuuguu.....................................................................    1      1    c15
....................uacccuguagaCccgaauuuguu.....................................................................    1      1    c15
....................uacccuguagauccgaauuuguC.....................................................................    26     1    c15
....................uacccuguagauccgaauuuguA.....................................................................    631    1    c15
....................uacccuguagauUcgaauuuguu.....................................................................    1      1    c15
....................uacccAguagauccgaauuuguu.....................................................................    1      1    c15
....................uacccuguagaAccgaauuuguu.....................................................................    2      1    c15
....................uacUcuguagauccgaauuuguu.....................................................................    1      1    c15
....................uacccuguagaucAgaauuuguu.....................................................................    1      1    c15
....................uacccuguGgauccgaauuuguu.....................................................................    1      1    c15
....................uacccuguagauccgaauuGguu.....................................................................    2      1    c15
....................uacccuguagauccgaauuugAu.....................................................................    1      1    c15
....................uacccuguagauccgUauuuguu.....................................................................    1      1    c15
....................uacccuguagauccgaauuuguG.....................................................................    8      1    c15
....................uacccuguaUauccgaauuuguu.....................................................................    1      1    c15
....................uaccAuguagauccgaauuuguu.....................................................................    1      1    c15
....................uacccuguagauccgaauuugCu.....................................................................    1      1    c15
....................uacccuguagaGccgaauuuguu.....................................................................    1      1    c15
....................uaccUuguagauccgaauuuguu.....................................................................    1      1    c15
....................uaccGuguagauccgaauuuguu.....................................................................    1      1    c15
....................uacccuguagauccgaauuuguuu....................................................................    157    0    c15
....................uacUcuguagauccgaauuuguuu....................................................................    1      1    c15
....................uacccuguagauccgaauuuguGu....................................................................    1      1    c15
....................uacccuguagaCccgaauuuguuu....................................................................    1      1    c15
....................uacccuguagauccgaauuuguuC....................................................................    4      1    c15
....................uacccuguagauccgaauuuguAu....................................................................    30     1    c15
....................uacccuAuagauccgaauuuguuu....................................................................    1      1    c15
....................uacccuguagauccgaauuuguCu....................................................................    3      1    c15
....................uacccuguagauccgaGuuuguuu....................................................................    1      1    c15
....................uacccuguagauccgaauuuguuA....................................................................    18     1    c15
....................uacccAguagauccgaauuuguuu....................................................................    1      1    c15
....................uacccuguagauccgaauuuguuuA...................................................................    3      1    c15
....................uacccuguagauccgaauuuguuug...................................................................    1      0    c15
....................uacccuguagauccgaauuuguuuCg..................................................................    1      1    c15
```

Mature Star

caauuuuagugcccuacaucuacccuguagauccgaauuuguuugaagugaggcgacaaauucgguucuagagagguuuguguggugcaugcacugcaacguuucaagucug			
....................uacccuguagauccgaauuuguuuU...	10	1	c15
....................uacccuguagauccgaauuuguuuga..	6	0	c15
....................uacccuguagauccgaauuuguuugaa...	3	0	c15
....................uacccuguagauccgaauuuguuugaU...	1	1	c15
....................uacccuguagauccgaauuuguuugaaU..	1	1	c15
....................uacccuguagauccgaauuuguuugaag..	4	0	c15
....................uacccuguagauccgaauuuguuugaagug..	1	0	c15
....................uacccuguagauccgaauuuguuugaaguga...	2	0	c15
.....................acccuguagauGcgaauu...	1	1	c15
.....................aAccuguagauccgaauu...	1	1	c15
.....................acccugCagauccgaauu...	1	1	c15
.....................acccuguagauccgaauu...	722	0	c15
.....................acccuCuagauccgaauu...	1	1	c15
.....................aGccuguagauccgaauu...	2	1	c15
.....................acccuguagauUcgaauu...	1	1	c15
.....................Ucccuguagauccgaauu...	1	1	c15
.....................acccuguCgauccgaauu...	1	1	c15
.....................acccuguagauccgaGuu...	1	1	c15
.....................acccuguagaucUgaauuu..	1	1	c15
.....................acccugCagauccgaauuu..	1	1	c15
.....................acccuguagauccgaauuu..	362	0	c15
.....................acccuguagauccgaUuuu..	1	1	c15
.....................acccuguagauccgaauuC..	1	1	c15
.....................acUcuguagauccgaauuu..	1	1	c15
.....................acccuguagauccgaCuuug...	1	1	c15
.....................acccuguagauccUaauuug...	2	1	c15
.....................acccuguagauccgaaAuug...	1	1	c15
.....................acccCguagauccgaauuug...	8	1	c15
.....................aAccuguagauccgaauuug...	3	1	c15
.....................acUcuguagauccgaauuug...	4	1	c15
.....................acccuguCgauccgaauuug...	1	1	c15
.....................acccuguagauccAaauuug...	4	1	c15
.....................acccuguagauccgaauuuC...	6	1	c15
.....................acccuguagaucGgaauuug...	7	1	c15
.....................acccuguagaAccgaauuug...	1	1	c15
.....................acccuguagauccgaauuug...	9724	0	c15
.....................acccuguagauccgCauuug...	1	1	c15
.....................acccuguagauGcgaauuug...	1	1	c15
.....................acccugAagauccgaauuug...	2	1	c15
.....................acccuguagaCccgaauuug...	2	1	c15
.....................accAuguagauccgaauuug...	5	1	c15
.....................acccugCagauccgaauuug...	2	1	c15
.....................acccuguagauUcgaauuug...	5	1	c15
.....................acccuguagauccgaGuuug...	2	1	c15
.....................acAcuguagauccgaauuug...	1	1	c15
.....................acccGguagauccgaauuug...	3	1	c15
.....................acccuUuagauccgaauuug...	1	1	c15
.....................accUuguagauccgaauuug...	4	1	c15
.....................acccuguagaucUgaauuug...	7	1	c15
.....................Gcccuguagauccgaauuug...	7	1	c15
.....................acccuguagauccgaauAug...	2	1	c15
.....................acccuAuagauccgaauuug...	8	1	c15
.....................acccuguGgauccgaauuug...	2	1	c15
.....................aGccuguagauccgaauuug...	5	1	c15
.....................acccuguagauccgaauuuU...	15	1	c15
.....................Ucccuguagauccgaauuug...	2	1	c15
.....................acccuguagauccgGauuug...	1	1	c15
.....................Ccccuguagauccgaauuug...	1	1	c15
.....................acccuguagauccgaauuuA...	275	1	c15
.....................acccuguagaucAgaauuug...	1	1	c15
.....................acccuguagauccgaUuuug...	2	1	c15
.....................acccuguagauccgaauuCg...	3	1	c15
.....................acccuguagauccgaauuAg...	1	1	c15
.....................acccuCuagauccgaauuug...	1	1	c15
.....................acccuguagGuccgaauuug...	1	1	c15
.....................aUccuguagauccgaauuug...	2	1	c15
.....................acccAguagauccgaauuug...	3	1	c15
.....................acccuguagauAcgaauuugu..	6	1	c15
.....................acccuguagauccgaauuuCu..	7	1	c15
.....................acccuguagauccgaauAugu..	6	1	c15

```
                     Mature                Star
caauuuuagugcccuacaucuacccuguagauccgaauuuguuugaagugaggcgacaaauucgguucuagagagguuuguguggugcaugcacugcaacguuucaagucug
.....................aGccuguagauccgaauuugu......................................................................   27      1   c15
.....................aAccuguagauccgaauuugu......................................................................   17      1   c15
.....................acccuguagauccgaCuuugu......................................................................   6       1   c15
.....................acccuguagauccgUauuugu......................................................................   5       1   c15
.....................acccuguGgauccgaauuugu......................................................................   17      1   c15
.....................accGuguagauccgaauuugu......................................................................   1       1   c15
.....................acccuguagauccUaauuugu......................................................................   8       1   c15
.....................acccuguagauUcgaauuugu......................................................................   23      1   c15
.....................acccuguCgauccgaauuugu......................................................................   2       1   c15
.....................acccuguagaGccgaauuugu......................................................................   4       1   c15
.....................acccuguagaucGgaauuugu......................................................................   14      1   c15
.....................acccuguagaCccgaauuugu......................................................................   3       1   c15
.....................acUcuguagauccgaauuugu......................................................................   14      1   c15
.....................acccuguagauccgaUuuugu......................................................................   16      1   c15
.....................acccuguaUauccgaauuugu......................................................................   6       1   c15
.....................Gcccuguagauccgaauuugu......................................................................   20      1   c15
.....................acccuguagauccgaaAuugu......................................................................   5       1   c15
.....................acccuguagauccgaauuugC......................................................................   224     1   c15
.....................acccuguaCauccgaauuugu......................................................................   2       1   c15
.....................acccuguagauccgGauuugu......................................................................   4       1   c15
.....................acccuguagauccgaauuGgu......................................................................   1       1   c15
.....................acccugAagauccgaauuugu......................................................................   8       1   c15
.....................acccuguagauccgaauuCgu......................................................................   11      1   c15
.....................accAuguagauccgaauuugu......................................................................   14      1   c15
.....................acccuUuagauccgaauuugu......................................................................   11      1   c15
.....................acccugGagauccgaauuugu......................................................................   5       1   c15
.....................acccuCuagauccgaauuugu......................................................................   4       1   c15
.....................acccuguagauccgaauuugA......................................................................   120     1   c15
.....................acccuguagauccgaGuuugu......................................................................   13      1   c15
.....................acccuguagauccgaauuuUu......................................................................   20      1   c15
.....................acccuguaAauccgaauuugu......................................................................   2       1   c15
.....................acccuguagauccgaauuugG......................................................................   17      1   c15
.....................acccuguagaucUgaauuugu......................................................................   26      1   c15
.....................acAcuguagauccgaauuugu......................................................................   7       1   c15
.....................aUccuguagauccgaauuugu......................................................................   16      1   c15
.....................Ccccuguagauccgaauuugu......................................................................   5       1   c15
.....................acccuguagauccgaauuuAu......................................................................   28      1   c15
.....................acccuguagauccgaaGuugu......................................................................   5       1   c15
.....................acccugCagauccgaauuugu......................................................................   14      1   c15
.....................acccGguagauccgaauuugu......................................................................   6       1   c15
.....................acGcuguagauccgaauuugu......................................................................   2       1   c15
.....................accUuguagauccgaauuugu......................................................................   9       1   c15
.....................acccAguagauccgaauuugu......................................................................   13      1   c15
.....................acccuguagauccAaauuugu......................................................................   16      1   c15
.....................acccuguagauccgaauCugu......................................................................   6       1   c15
.....................acccuguagaAccgaauuugu......................................................................   6       1   c15
.....................acccuguagauccgaaCuugu......................................................................   8       1   c15
.....................acccuguagauccgaauuugu......................................................................   43461   0   c15
.....................acccuguagaucAgaauuugu......................................................................   20      1   c15
.....................acccCguagauccgaauuugu......................................................................   20      1   c15
.....................acccuguagauccgaauGugu......................................................................   1       1   c15
.....................acccuguagauGcgaauuugu......................................................................   7       1   c15
.....................acccuguagauccgCauuugu......................................................................   5       1   c15
.....................acccuguagUuccgaauuugu......................................................................   2       1   c15
.....................Ucccuguagauccgaauuugu......................................................................   29      1   c15
.....................acccuAuagauccgaauuugu......................................................................   15      1   c15
.....................acccuguagGuccgaauuugu......................................................................   3       1   c15
.....................acccuguUgauccgaauuugu......................................................................   5       1   c15
.....................acccuguagauccgaauuAgu......................................................................   5       1   c15
.....................acccGguagauccgaauuuguu.....................................................................   15      1   c15
.....................acccuguagauccgaaCuuguu.....................................................................   9       1   c15
.....................acccuguagauccgGauuuguu.....................................................................   15      1   c15
.....................acccuguagauUcgaauuuguu.....................................................................   75      1   c15
.....................acccuguGgauccgaauuuguu.....................................................................   78      1   c15
.....................acccuUuagauccgaauuuguu.....................................................................   37      1   c15
.....................acccuguaAauccgaauuuguu.....................................................................   11      1   c15
.....................acccuguagauccgCauuuguu.....................................................................   11      1   c15
.....................acccuguagauccgaauuuUuu.....................................................................   33      1   c15
.....................acccuguagaucGgaauuuguu.....................................................................   43      1   c15
.....................acccuguagauccgaGuuuguu.....................................................................   59      1   c15
```

Mature **Star**

```
caauuuuagugcccuacaucuacccuguagauccgaauuuguuugaagugaggcgacaaauucgguucuagagagguuuguguggugcaugcacugcaacguuucaagucug
.....................acccuguagauGcgaauuuguu.....................................................................   28      1    c15
.....................acccuCuagauccgaauuuguu.....................................................................   10      1    c15
.....................acccAguagauccgaauuuguu.....................................................................   36      1    c15
.....................acccuguagauAcgaauuuguu.....................................................................   22      1    c15
.....................accGuguagauccgaauuuguu.....................................................................   3       1    c15
.....................acccuguagauccgaauCuguu.....................................................................   20      1    c15
.....................aUccuguagauccgaauuuguu.....................................................................   94      1    c15
.....................acccuguUgauccgaauuuguu.....................................................................   19      1    c15
.....................acccuguagauccgaauuuCuu.....................................................................   15      1    c15
.....................acUcuguagauccgaauuuguu.....................................................................   29      1    c15
.....................acccuguagauccgaauuugAu.....................................................................   86      1    c15
.....................acccuguagauccgaaAuuguu.....................................................................   12      1    c15
.....................acccuguagaCccgaauuuguu.....................................................................   22      1    c15
.....................aAccuguagauccgaauuuguu.....................................................................   44      1    c15
.....................aGccuguagauccgaauuuguu.....................................................................   104     1    c15
.....................accUuguagauccgaauuuguu.....................................................................   41      1    c15
.....................acccuguagaAccgaauuuguu.....................................................................   18      1    c15
.....................acccuguaCauccgaauuuguu.....................................................................   7       1    c15
.....................acAcuguagauccgaauuuguu.....................................................................   22      1    c15
.....................acGcuguagauccgaauuuguu.....................................................................   7       1    c15
.....................acccuguagauccgaauuuguG.....................................................................   36      1    c15
.....................acccuguagauccgaauAuguu.....................................................................   11      1    c15
.....................acccuguagauccgaauuugCu.....................................................................   115     1    c15
.....................Ucccuguagauccgaauuuguu.....................................................................   93      1    c15
.....................accAuguagauccgaauuuguu.....................................................................   41      1    c15
.....................Ccccuguagauccgaauuuguu.....................................................................   13      1    c15
.....................acccuguagauccCaauuuguu.....................................................................   12      1    c15
.....................acccugAagauccgaauuuguu.....................................................................   30      1    c15
.....................acccuguagauccgaauuuguC.....................................................................   328     1    c15
.....................acccuguagaucAgaauuuguu.....................................................................   49      1    c15
.....................acccugCagauccgaauuuguu.....................................................................   55      1    c15
.....................acccuguagGuccgaauuuguu.....................................................................   8       1    c15
.....................acccuguagauccgaauuGguu.....................................................................   4       1    c15
.....................acccuguagaucUgaauuuguu.....................................................................   85      1    c15
.....................Gcccuguagauccgaauuuguu.....................................................................   103     1    c15
.....................acccugGagauccgaauuuguu.....................................................................   34      1    c15
.....................acccuAuagauccgaauuuguu.....................................................................   76      1    c15
.....................acccuguagUuccgaauuuguu.....................................................................   4       1    c15
.....................acccuguagCuccgaauuuguu.....................................................................   3       1    c15
.....................acccuguagauccgaaGuuguu.....................................................................   18      1    c15
.....................acccuguagauccgaauuuguA.....................................................................   353     1    c15
.....................acccuguagauccgaauuuguu.....................................................................   155875  0    c15
.....................acccuguagauccgaUuuuguu.....................................................................   79      1    c15
.....................acccuguagauccAaauuuguu.....................................................................   36      1    c15
.....................acccuguCgauccgaauuuguu.....................................................................   1       1    c15
.....................acccuguagauccUaauuuguu.....................................................................   34      1    c15
.....................acccuguagauccgaauGuguu.....................................................................   2       1    c15
.....................acccuguagauccgaCuuuguu.....................................................................   33      1    c15
.....................acccuguagauccgaauuAguu.....................................................................   15      1    c15
.....................acccCguagauccgaauuuguu.....................................................................   83      1    c15
.....................acccuguaUauccgaauuuguu.....................................................................   23      1    c15
.....................acccuguagauccgaauuuAuu.....................................................................   59      1    c15
.....................acccuguagauccgaauuugGu.....................................................................   29      1    c15
.....................acccuguagaGccgaauuuguu.....................................................................   5       1    c15
.....................acccuguagauccgaauuCguu.....................................................................   23      1    c15
.....................acccuguagauccgUauuuguu.....................................................................   34      1    c15
.....................acccuguagaucGgaauuuguuu....................................................................   2       1    c15
.....................acccuguagauccgaCuuuguuu....................................................................   1       1    c15
.....................acccuguagaucUgaauuuguuu....................................................................   2       1    c15
.....................acccuguagauccgGauuuguuu....................................................................   1       1    c15
.....................acccuguagauccgaauuCguuu....................................................................   2       1    c15
.....................acccuguagauccgaauuuguuA....................................................................   1285    1    c15
.....................acccuguagauccgaauuuUuuu....................................................................   3       1    c15
.....................aUccuguagauccgaauuuguuu....................................................................   4       1    c15
.....................acccuguagauccgaauuugAuu....................................................................   3       1    c15
.....................acUcuguagauccgaauuuguuu....................................................................   1       1    c15
.....................acccuguagaucAgaauuuguuu....................................................................   4       1    c15
.....................acccuguagauccgaauuuguCu....................................................................   3       1    c15
.....................acccuguagauccgaaGuuguuu....................................................................   1       1    c15
.....................acccuguagauccgaauuAuuu.....................................................................   1       1    c15
```

Mature **Star**

```
caauuuuagugcccuacaucuacccuguagauccgaauuuguuuugaagugaggcgacaaauucgguucuagagagguuugugguggugcaugcacugcaacguuucaagucug
.....................acccAguagauccgaauuuguuu.....................................................................	2	1	c15
.....................acccuguagauccgaGuuuguuu.....................................................................	3	1	c15
.....................acccuguGgauccgaauuuguuu.....................................................................	5	1	c15
.....................acccugGagauccgaauuuguuu.....................................................................	1	1	c15
.....................acccuguaUauccgaauuuguuu.....................................................................	2	1	c15
.....................acccuguagauUcgaauuuguuu.....................................................................	3	1	c15
.....................aGccuguagauccgaauuuguuu.....................................................................	9	1	c15
.....................acccuUuagauccgaauuuguuu.....................................................................	1	1	c15
.....................accGuguagauccgaauuuguuu.....................................................................	1	1	c15
.....................acccuguagauccgUauuuguuu.....................................................................	1	1	c15
.....................acccuguagaAccgaauuuguuu.....................................................................	2	1	c15
.....................aAccuguagauccgaauuuguuu.....................................................................	2	1	c15
.....................acccuguagauccgaauuuguGu.....................................................................	1	1	c15
.....................acccuguagauGcgaauuuguuu.....................................................................	3	1	c15
.....................acccGguagauccgaauuuguuu.....................................................................	1	1	c15
.....................acccugCagauccgaauuuguuu.....................................................................	8	1	c15
.....................acccuguagCuccgaauuuguuu.....................................................................	1	1	c15
.....................acccuguagauccgaauAuguuu.....................................................................	2	1	c15
.....................acccuguagauccAaauuuguuu.....................................................................	2	1	c15
.....................acccuAuagauccgaauuuguuu.....................................................................	4	1	c15
.....................acccuguagauccgaauuugGuu.....................................................................	1	1	c15
.....................acccuguagauccgaauGuguuu.....................................................................	1	1	c15
.....................accAuguagauccgaauuuguuu.....................................................................	1	1	c15
.....................acccuguagauccgaauuuguuC.....................................................................	148	1	c15
.....................acAcuguagauccgaauuuguuu.....................................................................	1	1	c15
.....................acccuguagGuccgaauuuguuu.....................................................................	2	1	c15
.....................acccuguagauccgaaCuuguuu.....................................................................	1	1	c15
.....................acccuguagauccgaUuuuguuu.....................................................................	5	1	c15
.....................Ucccuguagauccgaauuuguuu.....................................................................	8	1	c15
.....................acccCguagauccgaauuuguuu.....................................................................	6	1	c15
.....................acccuguagauccgaauuuguuG.....................................................................	13	1	c15
.....................acccuguagaCccgaauuuguuu.....................................................................	3	1	c15
.....................acccuguagauccgaauuuguAu.....................................................................	7	1	c15
.....................acccuguaAauccgaauuuguuu.....................................................................	1	1	c15
.....................acccuguagauccgaauuugCuu.....................................................................	3	1	c15
.....................acccuguagauAcgaauuuguuu.....................................................................	1	1	c15
.....................Gcccuguagauccgaauuuguuu.....................................................................	8	1	c15
.....................acccuguagauccgaauuuguuu.....................................................................	7561	0	c15
.....................acccuguagauccgaauuuguuuC....................................................................	14	1	c15
.....................acccuguagauccgaauuuguCug....................................................................	1	1	c15
.....................acccuguagauccgaauuuguuuA....................................................................	73	1	c15
.....................acccuguagauccgaauuuguuuU....................................................................	401	1	c15
.....................acccuguagauccgaauuuguuug....................................................................	12	0	c15
.....................acccuguagauccgaauuuguuuga...................................................................	6	0	c15
.....................acccuguagauccgaauuuguuuUa...................................................................	4	1	c15
.....................acccuguagauccgaauuuguuuAa...................................................................	4	1	c15
.....................acccuguagauccgaauuuguuuCa...................................................................	3	1	c15
.....................acccuguagauccgaauuuguuugaa..................................................................	13	0	c15
.....................acccuguagauccgaauuuguuuAaa..................................................................	2	1	c15
.....................acccuguagauccgaauuuguuugGa..................................................................	1	1	c15
.....................acccuguagauccgaauuuguuuUaa..................................................................	1	1	c15
.....................acccuguagauccgaauuuguuugaag.................................................................	44	0	c15
.....................acccuguagauccgaauuuguuugaagu................................................................	71	0	c15
.....................acccuguagauccgaauuuguuugaagA................................................................	3	1	c15
.....................acccuguagauccgaauuuguuugaaguU...............................................................	2	1	c15
.....................acccuguagauccgaauuuguuugaaguA...............................................................	1	1	c15
.....................acccuguagauccgaauuuguuugaagug...............................................................	3	0	c15
.....................acccuguagauccgaauuuguuugaaguUa..............................................................	1	1	c15
.....................acccuguagauccgaauuuguuugaagugagg............................................................	1	0	c15
......................cccuguagauccgaauuug........................................................................	1	0	c15
......................cccuguagauccgaauuugu.......................................................................	17	0	c15
......................cccugGagauccgaauuugu.......................................................................	1	1	c15
......................ccUuguagauccgaauuuguu......................................................................	1	1	c15
......................cccuguagauccgaauuuguu......................................................................	35	0	c15
......................cccCguagauccgaauuuguu......................................................................	1	1	c15
......................Accuguagauccgaauuuguu......................................................................	2	1	c15
.......................ccuguagauccgaauuug........................................................................	11	0	c15
.......................ccuguagauccgaGuuugu.......................................................................	1	1	c15
.......................ccugAagauccgaauuugu.......................................................................	1	1	c15
.......................ccuguagauccgaauuugC.......................................................................	1	1	c15
```

Mature   Star

```
caauuuuagugcccuacaucuacccuguagauccgaauuuguuugaagugaggcgacaaauucgguucuagagagguuuguguggugcaugcacugcaacguuucaagucug
.......................Ucuguagauccgaauuugu......................................................................    1      1      c15
.......................ccuguagauccgaauuugA......................................................................    1      1      c15
.......................ccuguagauUcgaauuugu......................................................................    1      1      c15
.......................ccuguagauccgaauuugu......................................................................    153    0      c15
.......................ccuguagauccgaauGuguu.....................................................................    1      1      c15
.......................ccuguagauccgaauuuguu.....................................................................    119    0      c15
.......................ccuguUgauccgaauuuguu.....................................................................    1      1      c15
.......................ccuguagauccgaauuAguu.....................................................................    1      1      c15
.......................ccuguagauccgaauuugAu.....................................................................    1      1      c15
.......................ccuguagauccgaauuuguA.....................................................................    1      1      c15
.......................ccuguagauccgaauuuguC.....................................................................    1      1      c15
.......................ccuguagauccgaauuuguuA....................................................................    3      1      c15
.......................ccuguagauccgaauuuguuu....................................................................    7      0      c15
.......................ccuguagauccgaauuuguuuA...................................................................    1      1      c15
........................cuguagauccgaauuugu......................................................................    117    0      c15
........................cuUuagauccgaauuuguu.....................................................................    1      1      c15
........................cuguagauccgaauuuguu.....................................................................    72     0      c15
........................cuguagauccgaauuuguuu....................................................................    6      0      c15
.........................uguagauccgaauuuguu.....................................................................    52     0      c15
...........................................ugaagugaggcgacaaauucgguucu...........................................    1      0      c15
.....................................................Ggacaaauucgguucuagagagg....................................    1      1      c15
.....................................................cgacaaauucgguucuagagagguuu.................................    3      0      c15
.....................................................cgacaaauucgguucuagagagguuug................................    1      0      c15
.......................................................acaaauucgguucuagagag.....................................    1      0      c15
.......................................................acaaauucgguucuagagaggu...................................    1      0      c15
.......................................................acaaauucgguucuagagagguu..................................    1      0      c15
.......................................................Ccaaauucgguucuagagagguu..................................    1      1      c15
.......................................................Ucaaauucgguucuagagagguuu.................................    1      1      c15
.......................................................acaaauucgguucuagagagguuu.................................    6      0      c15
.......................................................Ccaaauucgguucuagagagguuu.................................    13     1      c15
.......................................................acaaauucgguucuagagagguuuA................................    1      1      c15
.......................................................acaaauucgguucuagagagguuuU................................    2      1      c15
........................................................caaauucgguucCagaga......................................    1      1      c15
........................................................caaauucgguucuagaga......................................    391    0      c15
........................................................caaauucggAucuagaga......................................    1      1      c15
........................................................caaauucgguucuaAgag......................................    1      1      c15
........................................................Aaaauucgguucuagagag.....................................    2      1      c15
........................................................caaauucgguucuagagaC.....................................    9      1      c15
........................................................caaauGcgguucuagagag.....................................    1      1      c15
........................................................caaauucgguucuagagaU.....................................    2      1      c15
........................................................caaauucgguucuagagaA.....................................    4      1      c15
........................................................caaauucgguucuagagag.....................................    851    0      c15
........................................................caaauucggAucuagagag.....................................    1      1      c15
........................................................cGaauucgguucuagagag.....................................    1      1      c15
........................................................caaauucgguGcuagagag.....................................    1      1      c15
........................................................caaGuucgguucuagagagg....................................    1      1      c15
........................................................caaauucgguuUuagagagg....................................    2      1      c15
........................................................cUaauucgguucuagagagg....................................    1      1      c15
........................................................caaauucgguuAuagagagg....................................    1      1      c15
........................................................caaUuucgguucuagagagg....................................    1      1      c15
........................................................caaauucgguucuagagaUg....................................    3      1      c15
........................................................caaaAucgguucuagagagg....................................    1      1      c15
........................................................caaauucgguucuagagGgg....................................    1      1      c15
........................................................caaauucgguucCagagagg....................................    3      1      c15
........................................................caaauucgguucuGgagagg....................................    4      1      c15
........................................................caaauucggCucuagagagg....................................    2      1      c15
........................................................caaauucgguucuUgagagg....................................    2      1      c15
........................................................caaauGcgguucuagagagg....................................    1      1      c15
........................................................caaauucgguucuagagagU....................................    19     1      c15
........................................................caaauucgguucuagUgagg....................................    2      1      c15
........................................................caaauucggAucuagagagg....................................    6      1      c15
........................................................caaauucUguucuagagagg....................................    1      1      c15
........................................................caaauucgguucuaUagagg....................................    2      1      c15
........................................................Uaaauucgguucuagagagg....................................    10     1      c15
........................................................caaauCcgguucuagagagg....................................    1      1      c15
........................................................caaauucggGucuagagagg....................................    1      1      c15
........................................................caaauucgguucuagGgagg....................................    3      1      c15
........................................................caaauucgguucuagagagg....................................    6846   0      c15
........................................................caaauucAguucuagagagg....................................    2      1      c15
........................................................caaauAcgguucuagagagg....................................    3      1      c15
```

```
                     Mature                           Star
caauuuuagugcccuacaucuacccuguagauccgaauuuguuugaagugaggcgacaaauucgguucuagagagguuugugguggugcaugcacugcaacguuucaagucug
........................................................caaauucgguucuagagagA....................................	56	1	c15
........................................................caaauucgUuucuagagagg....................................	1	1	c15
........................................................caaauucgguAcuagagagg....................................	1	1	c15
........................................................caaauucgguucuagaUagg....................................	3	1	c15
........................................................cGaauucgguucuagagagg....................................	2	1	c15
........................................................caUauucgguucuagagagg....................................	1	1	c15
........................................................caaauuAgguucuagagagg....................................	1	1	c15
........................................................caaauucgguucGagagagg....................................	3	1	c15
........................................................Aaaauucgguucuagagagg....................................	5	1	c15
........................................................caaauucgguucuagagagC....................................	12	1	c15
........................................................Aaaauucgguucuagagaggu...................................	7	1	c15
........................................................caaaAucgguucuagagaggu...................................	2	1	c15
........................................................caaauucgguucuagagGggu...................................	3	1	c15
........................................................caaauucgguucuagagaUgu...................................	1	1	c15
........................................................caaauucgCuucuagagaggu...................................	1	1	c15
........................................................caaauucgguucuagagCggu...................................	2	1	c15
........................................................caaauucgguucAagagaggu...................................	1	1	c15
........................................................caaauucgguuAuagagaggu...................................	1	1	c15
........................................................caaauucggGucuagagaggu...................................	8	1	c15
........................................................caaauucgguucuagagagAu...................................	8	1	c15
........................................................caaauucgguucuagagaCgu...................................	1	1	c15
........................................................caaauucCguucuagagaggu...................................	2	1	c15
........................................................caaauucgguucGagagaggu...................................	9	1	c15
........................................................caaauucgguucuagaUaggu...................................	5	1	c15
........................................................caaauuUgguucuagagaggu...................................	2	1	c15
........................................................caaauucgguucuagagaggC...................................	66	1	c15
........................................................caaauAcgguucuagagaggu...................................	6	1	c15
........................................................caUauucgguucuagagaggu...................................	1	1	c15
........................................................Uaaauucgguucuagagaggu...................................	11	1	c15
........................................................caaauucgguuGuagagaggu...................................	4	1	c15
........................................................caaauucggCucuagagaggu...................................	5	1	c15
........................................................caaauucgguucuagagagCu...................................	9	1	c15
........................................................cGaauucgguucuagagaggu...................................	9	1	c15
........................................................caaauucgguucuaAagaggu...................................	1	1	c15
........................................................caaauucgguucuagCgaggu...................................	1	1	c15
........................................................caaauucgguAcuagagaggu...................................	1	1	c15
........................................................caaauucgguucuagagaAgu...................................	1	1	c15
........................................................caaauucUguucuagagaggu...................................	4	1	c15
........................................................caaauucgAuucuagagaggu...................................	1	1	c15
........................................................caaauucgguucuagagaggG...................................	7	1	c15
........................................................caaauucgguucuaUagaggu...................................	1	1	c15
........................................................caaauucgguucuagagaggu...................................	12757	0	c15
........................................................caaauucgguucuagagagUu...................................	3	1	c15
........................................................caaauucgguucCagagaggu...................................	3	1	c15
........................................................caaUuucgguucuagagaggu...................................	2	1	c15
........................................................caaauucgUuucuagagaggu...................................	4	1	c15
........................................................caaauucgguucuUgagaggu...................................	3	1	c15
........................................................caaauucgguuUuagagaggu...................................	2	1	c15
........................................................caaauGcgguucuagagaggu...................................	3	1	c15
........................................................cCaauucgguucuagagaggu...................................	1	1	c15
........................................................caaauuGgguucuagagaggu...................................	2	1	c15
........................................................caaauucAguucuagagaggu...................................	4	1	c15
........................................................caaauucgguucuagagaggA...................................	42	1	c15
........................................................caaauucgguucuagGgaggu...................................	1	1	c15
........................................................caaauucggAucuagagaggu...................................	9	1	c15
........................................................caaauucgguucuagagUggu...................................	2	1	c15
........................................................caaauucgguCcuagagaggu...................................	1	1	c15
........................................................caaauucgguucuagUgaggu...................................	5	1	c15
........................................................Gaaauucgguucuagagaggu...................................	1	1	c15
........................................................caaauucgguucuGgagaggu...................................	7	1	c15
........................................................cUaauucgguucuagagaggu...................................	4	1	c15
........................................................caaauAcgguucuagagagguu..................................	3	1	c15
........................................................caaauuGgguucuagagagguu..................................	1	1	c15
........................................................caaauucgguucuagagaggGu..................................	12	1	c15
........................................................caaauucgguCcuagagagguu..................................	2	1	c15
........................................................caaauucgguucuagUgagguu..................................	1	1	c15
........................................................caaauuAgguucuagagagguu..................................	1	1	c15
........................................................caaauucggGucuagagagguu..................................	4	1	c15
........................................................caaauucgguucuaCagagguu..................................	1	1	c15
........................................................caaauucgguucuagagagCuu..................................	2	1	c15
```

```
                     Mature                      Star
caauuuuagugcccuacaucuacccuguagauccgaauuuguuugaagugaggcgacaaauucgguucuagagagguuuguguggugcaugcacugcaacguuucaagucug
........................................................caaauucgguucuagagagAuu..................................  6      1  c15
........................................................caaaAucgguucuagagagguu..................................  3      1  c15
........................................................caaauucgguucuagagagguC..................................  47     1  c15
........................................................caaauucgguucuagagaAguu..................................  4      1  c15
........................................................Gaaauucgguucuagagagguu..................................  3      1  c15
........................................................caaauucgguucuagaCagguu..................................  1      1  c15
........................................................cUaauucgguucuagagagguu..................................  11     1  c15
........................................................cGaauucgguucuagagagguu..................................  15     1  c15
........................................................caaauucgguAcuagagagguu..................................  3      1  c15
........................................................caaauucgguucuGgagagguu..................................  8      1  c15
........................................................caaauucgguucuagagGgguu..................................  2      1  c15
........................................................caUauucgguucuagagagguu..................................  1      1  c15
........................................................caaauucgguucuagagagguA..................................  147    1  c15
........................................................caaauucgAuucuagagagguu..................................  1      1  c15
........................................................cCaauucgguucuagagagguu..................................  1      1  c15
........................................................caaauucgguucuagagaggAu..................................  34     1  c15
........................................................caaauucgguucuagagaggCu..................................  23     1  c15
........................................................caaauucgguuUuagagagguu..................................  5      1  c15
........................................................caaauucgguucCagagagguu..................................  5      1  c15
........................................................caaauucgguucuagGgagguu..................................  6      1  c15
........................................................caaauucgguucuagCgagguu..................................  3      1  c15
........................................................caaauGcgguucuagagagguu..................................  12     1  c15
........................................................caaauucgguucuagagagguG..................................  10     1  c15
........................................................caaauucCguucuagagagguu..................................  1      1  c15
........................................................caaauucgCuucuagagagguu..................................  1      1  c15
........................................................caaauucggCucuagagagguu..................................  6      1  c15
........................................................caaauucgguucuagagagUuu..................................  8      1  c15
........................................................caaauucgguucGagagagguu..................................  5      1  c15
........................................................caaauucgguucuagagCgguu..................................  1      1  c15
........................................................caaauucgguucuUgagagguu..................................  6      1  c15
........................................................caaauucAguucuagagagguu..................................  2      1  c15
........................................................caaauucgUuucuagagagguu..................................  2      1  c15
........................................................caGauucgguucuagagagguu..................................  1      1  c15
........................................................caaauucgguucAagagagguu..................................  1      1  c15
........................................................caaauucgguuGuagagagguu..................................  2      1  c15
........................................................caaauucgguucuagaUagguu..................................  6      1  c15
........................................................caaauucgguucuagaAagguu..................................  2      1  c15
........................................................caaauucgguucuagagaCguu..................................  1      1  c15
........................................................caaauucgguucuaUagagguu..................................  3      1  c15
........................................................Uaaauucgguucuagagagguu..................................  30     1  c15
........................................................caaauucgguucuagagagguu..................................  18989  0  c15
........................................................caaauucgguucuaAagagguu..................................  2      1  c15
........................................................caaauucUguucuagagagguu..................................  1      1  c15
........................................................caaauucggAucuagagagguu..................................  17     1  c15
........................................................caaauucgguucuagagUgguu..................................  1      1  c15
........................................................Aaaauucgguucuagagagguu..................................  12     1  c15
........................................................caaauucgguucuagagaUguu..................................  2      1  c15
........................................................caaauuUgguucuagagagguu..................................  3      1  c15
........................................................caaaCucgguucuagagagguu..................................  1      1  c15
........................................................caaauucgguucuagGgagguuu.................................  7      1  c15
........................................................caaauucgguucuagagagguuA.................................  482    1  c15
........................................................caaauucgguucuUgagagguuu.................................  15     1  c15
........................................................caaGuucgguucuagagagguuu.................................  3      1  c15
........................................................caaauucgguucuagUgagguuu.................................  19     1  c15
........................................................caaauucgguCcuagagagguuu.................................  16     1  c15
........................................................caaauucUguucuagagagguuu.................................  16     1  c15
........................................................caaUuucgguucuagagagguuu.................................  4      1  c15
........................................................caaauucgguucAagagagguuu.................................  18     1  c15
........................................................caaauucgguucuagaAagguuu.................................  5      1  c15
........................................................caaauucgguGcuagagagguuu.................................  3      1  c15
........................................................caaauucgUuucuagagagguuu.................................  9      1  c15
........................................................caaauAcgguucuagagagguuu.................................  13     1  c15
........................................................caaauucgguucuagaCagguuu.................................  5      1  c15
........................................................caaauucgguucuagagagAuuu.................................  36     1  c15
........................................................caaauucgguucuagagagCuuu.................................  7      1  c15
........................................................caaauucggGucuagagagguuu.................................  40     1  c15
........................................................caaauucgguucuagagGgguuu.................................  21     1  c15
........................................................caaauucgguucuagagaCguuu.................................  1      1  c15
........................................................caaauucgguuAuagagagguuu.................................  9      1  c15
........................................................caaauucgguucuagagagguGu.................................  5      1  c15
```

Mature Star

```
caauuuuagugcccuacaucuacccuguagauccgaauuuguuugaagugaggcgacaaauucgguucuagagagguuugugugguugcaugcacugcaacguuucaagucug
........................................................caaauucgguucuGgagagguuu.................................  18      1   c15
........................................................caaauucgAuucuagagagguuu.................................  17      1   c15
........................................................caaauucgguucuagagaAguuu.................................  7       1   c15
........................................................caaauucgguucuagagaUguuu.................................  9       1   c15
........................................................caaauucgguuUuagagagguuu.................................  26      1   c15
........................................................caaauucCguucuagagagguuu.................................  3       1   c15
........................................................caaauucgguuGuagagagguuu.................................  11      1   c15
........................................................caaauucgguAcuagagagguuu.................................  10      1   c15
........................................................caaauucgguucuaCagagguuu.................................  5       1   c15
........................................................Uaaauucgguucuagagagguuu.................................  126     1   c15
........................................................caaauucgguucuCgagagguuu.................................  4       1   c15
........................................................caaauucgguucuagagagguuC.................................  253     1   c15
........................................................caaauucgguucuagagaggGuu.................................  50      1   c15
........................................................caaauucgguucuagagagguCu.................................  26      1   c15
........................................................caaauuUgguucuagagagguuu.................................  19      1   c15
........................................................caaaCucgguucuagagagguuu.................................  5       1   c15
........................................................caaauGcgguucuagagagguuu.................................  24      1   c15
........................................................caUauucgguucuagagagguuu.................................  8       1   c15
........................................................caaauCcgguucuagagagguuu.................................  8       1   c15
........................................................caaauucgguucGagagagguuu.................................  19      1   c15
........................................................Gaaauucgguucuagagagguuu.................................  5       1   c15
........................................................caaauucAguucuagagagguuu.................................  20      1   c15
........................................................caaauucgguucuagagUgguuu.................................  4       1   c15
........................................................cUaauucgguucuagagagguuu.................................  43      1   c15
........................................................caaauuGgguucuagagagguuu.................................  3       1   c15
........................................................caaauucgguucCagagagguuu.................................  19      1   c15
........................................................caaauucggAucuagagagguuu.................................  72      1   c15
........................................................caaauucgguucuagaUagguuu.................................  19      1   c15
........................................................cCaauucgguucuagagagguuu.................................  8       1   c15
........................................................caaauucgguucuagagagguuG.................................  23      1   c15
........................................................Aaaauucgguucuagagagguuu.................................  59      1   c15
........................................................caaauucgguucuagagaggCuu.................................  86      1   c15
........................................................caaauucgCuucuagagagguuu.................................  1       1   c15
........................................................caaauucgguucuagCgagguuu.................................  1       1   c15
........................................................caaauucgguucuagagagguuu.................................  85793   0   c15
........................................................caGauucgguucuagagagguuu.................................  7       1   c15
........................................................caaauucgguucuaUagagguuu.................................  22      1   c15
........................................................caaauucgguucuagagaggAuu.................................  132     1   c15
........................................................caaauucgguucuagagagUuuu.................................  43      1   c15
........................................................caaaAucgguucuagagagguuu.................................  4       1   c15
........................................................caaauuAgguucuagagagguuu.................................  11      1   c15
........................................................caaauucgguucuagagagguAu.................................  71      1   c15
........................................................caaauucgguucuaAagagguuu.................................  9       1   c15
........................................................caaauucggCucuagagagguuu.................................  36      1   c15
........................................................cGaauucgguucuagagagguuu.................................  60      1   c15
........................................................caaauucgguucuagagagguuCg................................  1       1   c15
........................................................caaauucgguucuagagagguuuC................................  192     1   c15
........................................................cGaauucgguucuagagagguuug................................  1       1   c15
........................................................caaauucgguucuagGgagguuug................................  2       1   c15
........................................................caaauucgguucuagagaggAuug................................  4       1   c15
........................................................caaauucgguucuagagagguuuU................................  12953   1   c15
........................................................Aaaauucgguucuagagagguuug................................  3       1   c15
........................................................caaauucgguuGuagagagguuug................................  1       1   c15
........................................................Uaaauucgguucuagagagguuug................................  1       1   c15
........................................................caaauucgguucuagagagguCug................................  1       1   c15
........................................................caaauucgguucuagaUagguuug................................  1       1   c15
........................................................caaauucgguucuagagagguuuA................................  559     1   c15
........................................................caaauucgguucuagagagguuug................................  1904    0   c15
........................................................caaauucgguucGagagagguuug................................  1       1   c15
........................................................caaauucgAuucuagagagguuug................................  1       1   c15
........................................................caaauucgguucCagagagguuug................................  2       1   c15
........................................................caaauucggGucuagagagguuug................................  1       1   c15
........................................................caaauucgguucuagagagguuAg................................  2       1   c15
........................................................caaauucgguucuagagagAuuug................................  1       1   c15
........................................................caaauucgguucuCgagagguuug................................  1       1   c15
........................................................caaauucgguucuagagaggCuug................................  4       1   c15
........................................................caaauucgguucuagagagguuugu...............................  762     0   c15
........................................................caaauucgguucuagagagguuuCu...............................  51      1   c15
........................................................caaauAcgguucuagagagguuugu...............................  1       1   c15
........................................................caaauucgguucuagagagguuugA...............................  55      1   c15
```

Mature Star

```
caauuuuagugcccuacaucuacccuguagauccgaauuuguuugaagugaggcgacaaauucgguucuagagagguuuguguggugcaugcacugcaacguuucaagucug
........................................................caaauucggAucuagagagguuugu...............................	1	1	c15
........................................................caaauucgguucuagagaUguuugu...............................	1	1	c15
........................................................caUauucgguucuagagagguuugu...............................	1	1	c15
........................................................cGaauucgguucuagagagguuugu...............................	1	1	c15
........................................................caaauucgguucuagagagguuuUu...............................	2683	1	c15
........................................................caaauucgguucCagagagguuugu...............................	1	1	c15
........................................................caaauucgguucGagagagguuugu...............................	1	1	c15
........................................................caaauucgguucuagagagguuugC...............................	23	1	c15
........................................................caaauGcgguucuagagagguuugu...............................	2	1	c15
........................................................caaauucgguucuagagagguuuAu...............................	222	1	c15
........................................................caaauucgguucuagagagguuugug..............................	1	0	c15
........................................................caaauucgguucuagagagguuugGg..............................	1	1	c15
........................................................caaauucgguucuagagagguuuguA..............................	7	1	c15
........................................................caaauucgguucuagagagguuuUug..............................	2	1	c15
........................................................caaauucgguucuagagagguuuguC..............................	1	1	c15
........................................................caaauucgguucuagagagguuuguU..............................	76	1	c15
........................................................caaauucgguucuagagagguuuguCu.............................	2	1	c15
........................................................caaauucgguucuagagagguuuguAu.............................	11	1	c15
........................................................caaauucgguucuagagagguuuguUu.............................	34	1	c15
........................................................caaauucgguucuagagagguuugugu.............................	1	0	c15
.........................................................aaauucgguucuagagaC.....................................	1	1	c15
.........................................................aaauucgguucuagagag.....................................	26	0	c15
.........................................................aaauucgguucuagagaAg....................................	1	1	c15
.........................................................aaauucgguucuagagagg....................................	167	0	c15
.........................................................aaauucgguucuagagagA....................................	1	1	c15
.........................................................aaauucgCuucuagagagg....................................	1	1	c15
.........................................................aaauucgguucuagagagU....................................	1	1	c15
.........................................................aaauucgAuucuagagagg....................................	1	1	c15
.........................................................Caauucgguucuagagaggu...................................	1	1	c15
.........................................................aaauucggAucuagagaggu...................................	1	1	c15
.........................................................aaauucgguucuagagaggC...................................	2	1	c15
.........................................................aaauucgguuUuagagaggu...................................	1	1	c15
.........................................................aaauucgguucuagagUggu...................................	1	1	c15
.........................................................Uaauucgguucuagagaggu...................................	1	1	c15
.........................................................aaauuAgguucuagagaggu...................................	1	1	c15
.........................................................aaauucgguucuagagaggA...................................	1	1	c15
.........................................................aaauucgguucuGgagaggu...................................	1	1	c15
.........................................................aaauucgguucuagagaggu...................................	352	0	c15
.........................................................aaauucgguucuagagagUu...................................	1	1	c15
.........................................................aaauucgguucuagagagguA..................................	3	1	c15
.........................................................aaauucgguucuagagagguu..................................	640	0	c15
.........................................................aaauucgguucuagagaggAu..................................	1	1	c15
.........................................................aaauucgUuucuagagagguu..................................	1	1	c15
.........................................................Caauucgguucuagagagguu..................................	1	1	c15
.........................................................aaauucgguucuagagaUguu..................................	1	1	c15
.........................................................aaauucgguucuagagaggCuu.................................	5	1	c15
.........................................................aaauucgguucuagUgagguuu.................................	2	1	c15
.........................................................aaauuAgguucuagagagguuu.................................	1	1	c15
.........................................................aaauucgguucuagagCgguuu.................................	1	1	c15
.........................................................aaauucgguucuagagGgguuu.................................	1	1	c15
.........................................................Uaauucgguucuagagagguuu.................................	8	1	c15
.........................................................aaauucgguuUuagagagguuu.................................	2	1	c15
.........................................................aaauucgguucuagagagguuu.................................	7699	0	c15
.........................................................aaaCucgguucuagagagguuu.................................	5	1	c15
.........................................................aaauucgUuucuagagagguuu.................................	3	1	c15
.........................................................aaauucgguucuagagagguuC.................................	12	1	c15
.........................................................aGauucgguucuagagagguuu.................................	3	1	c15
.........................................................aaauAcgguucuagagagguuu.................................	1	1	c15
.........................................................aaauucgguucuagagaggGuu.................................	11	1	c15
.........................................................aaauucggAucuagagagguuu.................................	6	1	c15
.........................................................aaauucgguucuagagagguuG.................................	3	1	c15
.........................................................aaaAucgguucuagagagguuu.................................	2	1	c15
.........................................................aaauucgAuucuagagagguuu.................................	2	1	c15
.........................................................aaauucgguucuagagagAuuu.................................	2	1	c15
.........................................................aaauucgguucuagagagUuuu.................................	1	1	c15
.........................................................aaauucggGucuagagagguuu.................................	4	1	c15
.........................................................aaauucgguucuUgagagguuu.................................	1	1	c15
.........................................................aaauucgguCcuagagagguuu.................................	3	1	c15
.........................................................aaauuUgguucuagagagguuu.................................	5	1	c15
.........................................................aUauucgguucuagagagguuu.................................	2	1	c15
```

Mature Star

caauuuuagugcccuacaucuacccuguagauccgaauuuguuugaagugaggcgacaaauucgguucuagagagguuuguguggugcaugcacugcaacguuucaagucug			
....aaauucgguucuagaUagguuu.................................	1	1	c15
....aaauucgguuAuagagagguuu.................................	2	1	c15
....Caauucgguucuagagagguuu.................................	3	1	c15
....aaauucggCucuagagagguuu.................................	3	1	c15
....aaauucgguucuagagagguAu.................................	2	1	c15
....aaauucgguucuGgagagguuu.................................	7	1	c15
....aaauucgguucuagagagguuA.................................	36	1	c15
....aaauuGgguucuagagagguuu.................................	2	1	c15
....aaauucgguGcuagagagguuu.................................	1	1	c15
....aaauucgguucuaAagagguuu.................................	1	1	c15
....Gaauucgguucuagagagguuu.................................	3	1	c15
....aaauucgguucuagagaUguuu.................................	3	1	c15
....aaauucgCuucuagagagguuu.................................	1	1	c15
....aaauucgguucuagagaggAuu.................................	15	1	c15
....aaauucgguucCagagagguuu.................................	2	1	c15
....aaauucAguucuagagagguuu.................................	2	1	c15
....aaauuGgguucuagagagguuug................................	4	1	c15
....aaauucgguAcuagagagguuug................................	2	1	c15
....aaauucgUuucuagagagguuug................................	2	1	c15
....aaauucgguucuGgagagguuug................................	5	1	c15
....aaauucAguucuagagagguuug................................	3	1	c15
....aaauucgguucuagagagguuCg................................	5	1	c15
....aaauucggAucuagagagguuug................................	6	1	c15
....aaauucgguucuagagaggCuug................................	6	1	c15
....aaauAcgguucuagagagguuug................................	1	1	c15
....aaauucgCuucuagagagguuug................................	1	1	c15
....aaauucgguuAuagagagguuug................................	1	1	c15
....aaauucgguucuagagagguuuA................................	497	1	c15
....aaauucgguucuagagaAguuug................................	1	1	c15
....aaauucgguucuagagagguAug................................	2	1	c15
....aaauucggGucuagagagguuug................................	3	1	c15
....aaauucgguucCagagagguuug................................	1	1	c15
....aaauucgguucuagagaggGuug................................	8	1	c15
....aUauucgguucuagagagguuug................................	1	1	c15
....aaauCcgguucuagagagguuug................................	1	1	c15
....aaauucgguucuagagagguuGg................................	1	1	c15
....aaauucUguucuagagagguuug................................	1	1	c15
....aCauucgguucuagagagguuug................................	1	1	c15
....Uaauucgguucuagagagguuug................................	10	1	c15
....aaauucgguucuagagagguGug................................	2	1	c15
....aaaCucgguucuagagagguuug................................	1	1	c15
....aaauuUgguucuagagagguuug................................	5	1	c15
....aaauucggCucuagagagguuug................................	6	1	c15
....aaauucgguucuagagGgguuug................................	4	1	c15
....aaauucgguucGagagagguuug................................	1	1	c15
....aGauucgguucuagagagguuug................................	4	1	c15
....aaauucgguucuagagagUuuug................................	1	1	c15
....aaauucgguucuagagaUguuug................................	1	1	c15
....aaauucgguucuaUagagguuug................................	3	1	c15
....aaauucgAuucuagagagguuug................................	3	1	c15
....aaauucgguucuagagagguuug................................	8544	0	c15
....aaauuAgguucuagagagguuug................................	1	1	c15
....aaUuucgguucuagagagguuug................................	1	1	c15
....aaauucgguucuagagagguuuU................................	6902	1	c15
....aaauucgguucuagagagguCug................................	3	1	c15
....aaauucgguucuagGgagguuug................................	4	1	c15
....aaauucgguucuagaAagguuug................................	2	1	c15
....aaauucgguuUuagagagguuug................................	2	1	c15
....aaauucgguucuaAagagguuug................................	1	1	c15
....aaauucgguucuagagaggAuug................................	9	1	c15
....aaauucgguucuagaUagguuug................................	2	1	c15
....aaauucgguucuagagagguuAg................................	2	1	c15
....aaauucgguucuagagagguuuC................................	144	1	c15
....aaauucgguucuagCgagguuugu...............................	1	1	c15
....aaauucgguucuagagaCguuugu...............................	1	1	c15
....aaauucgguucuagagagguuugG...............................	1	1	c15
....aaauucgguucuagagaggGuugu...............................	1	1	c15
....Uaauucgguucuagagagguuugu...............................	3	1	c15
....aaauucgguCcuagagagguuugu...............................	1	1	c15
....aaauucgguucuagagagguuuCu...............................	21	1	c15

Mature **Star**

caauuuuagugcccuacaucuacccuguagauccgaauuuguuugaagugaggcgacaaauucgguucuagagagguuugugguggugcaugcacugcaacguuucaagucug			
....aaauucgguucuagagagguuuUu...............................	1813	1	c15
....aaauucgguucuagaAagguuugu...............................	2	1	c15
....aaauucgguucuagagaggAuugu...............................	3	1	c15
....aaauucgguucuagagagguuugA...............................	130	1	c15
....aaauucgguucuagagagguuuAu...............................	107	1	c15
....aaauucgguucuagagaAguuugu...............................	1	1	c15
....aaauucgguucuagagagguuCgu...............................	1	1	c15
....aaauucgguucuagagagguuugC...............................	59	1	c15
....aaauucgguucuagagagguuugu...............................	1769	0	c15
....Gaauucgguucuagagagguuugu...............................	1	1	c15
....aGauucgguucuagagagguuugu...............................	1	1	c15
....aaauuAgguucuagagagguuugu...............................	1	1	c15
....aaauucgguucuagagagguuugCg..............................	1	1	c15
....aaauucgguucuagagagguuuguA..............................	35	1	c15
....aaauucgguucuagagagguuugug..............................	1	0	c15
....aaauucgguucuagagagguuuguC..............................	7	1	c15
....aaauucgguucuagagagguuuguU..............................	137	1	c15
....aaauucgguucuagagagguuuguUu.............................	110	1	c15
....aaauucgguucuagagagguuuguAu.............................	42	1	c15
....aaauucgguucuagagagguuuguCu.............................	6	1	c15
....aauucgguucuagagagg....................................	1	0	c15
....aauucgguucuagagaggu...................................	3	0	c15
....aauucgguucuagGgaggu...................................	1	1	c15
....aauucgguucuagagagguu..................................	7	0	c15
....aauucgguucuagagagguuu.................................	49	0	c15
....aauucgguucuagagaggCuu.................................	1	1	c15
....aGuucgguucuagagagguuu.................................	2	1	c15
....aauucgguucuagagagguuug................................	21	0	c15
....aauucgguucuagagagguuuC................................	1	1	c15
....aauucgguucuagagagguuuU................................	37	1	c15
....aauucgguucuagagagguuuA................................	4	1	c15
....aauucgguucuagagagguuuCu...............................	1	1	c15
....aauucgguucuagagagguuugu...............................	18	0	c15
....aauucgguucuagagagguuuUu...............................	57	1	c15
....aauucgguucuagagagguuuguA..............................	1	1	c15
....aauucgguucuagagagguuuguAu.............................	1	1	c15
....auucgguucuagagaggu...................................	5	0	c15
....auucgguucuagagagguu..................................	4	0	c15
....auucgguucuagagagguuu.................................	27	0	c15
....auucgguucuagagagguuC.................................	1	1	c15
....auAcgguucuagagagguuu.................................	1	1	c15
....auucgguucuagagagguuuU................................	7	1	c15
....auucgguucuagagagguuuUu...............................	5	1	c15
....auucgguucuagagagguuugu...............................	1	0	c15
....uucgguucuagagagguu..................................	2	0	c15
....uucgguucuagagagguuu.................................	22	0	c15
....uucgguucuagagagguuuU................................	1	1	c15
....uucgguucuagagagguuug................................	6	0	c15
....uucgguucuagagagguuuA................................	1	1	c15
....uucgguucuagagagguuugu...............................	2	0	c15
....ucgguucuagagagguuu.................................	22	0	c15
....ucgguucuagagagguuuU................................	1	1	c15
....ucgguucuagagagguuug................................	2	0	c15
....ucgguucuagagagguuugu...............................	2	0	c15
....ucgguucuagagagguuuUu...............................	2	1	c15
....cgguucuagagagguuug................................	4	0	c15
....cgguucuagagagguuuU................................	2	1	c15
....cgguucuagagagguuuAu...............................	1	1	c15
....cgguucuagagagguuuUu...............................	4	1	c15
................Aaucuacccuguagauccgaauuuguu...	1	1	c16
..................Gcuacccuguagauccgaauuugu..	1	1	c16
...................cuacccuguagauccgaauuugu..	2	0	c16
...................cuacAcuguagauccgaauuugu..	1	1	c16
....................uacccuguagaucAgaau..	1	1	c16
....................uacccuguagauccgaau..	208	0	c16
....................uCcccuguagauccgaau..	2	1	c16
....................uacAcuguagauccgaau..	1	1	c16
....................Gacccuguagauccgaau..	18	1	c16
....................Aacccuguagauccgaau..	2	1	c16

Mature Star

```
caauuuuagugcccuacaucuacccuguagauccgaauuuguuugaagugaggcgacaaauucgguucuagagagguuuguguggugcaugcacugcaacguuucaagucug
....................uacccuguagauccgaaG..........................................................................	1	1	c16
....................uacccGguagauccgaau..........................................................................	1	1	c16
....................uaccAuguagauccgaau..........................................................................	1	1	c16
....................uacAcuguagauccgaauu.........................................................................	1	1	c16
....................uacccuguagauccgaauC.........................................................................	2	1	c16
....................Gacccuguagauccgaauu.........................................................................	23	1	c16
....................uacccuCuagauccgaauu.........................................................................	2	1	c16
....................uacccuguagauccgaauG.........................................................................	1	1	c16
....................uacccuguagauccgaauu.........................................................................	422	0	c16
....................Aacccuguagauccgaauu.........................................................................	2	1	c16
....................uaccUuguagauccgaauu.........................................................................	1	1	c16
....................uacccuguagauAcgaauu.........................................................................	1	1	c16
....................uacccuguagCuccgaauu.........................................................................	1	1	c16
....................uCcccuguagauccgaauu.........................................................................	1	1	c16
....................uacccugAagauccgaauu.........................................................................	3	1	c16
....................Gacccuguagauccgaauuu........................................................................	22	1	c16
....................uacccuguagauccgaauuu........................................................................	324	0	c16
....................uacccuguagauccgaauuA........................................................................	1	1	c16
....................uacccuguagCuccgaauuu........................................................................	2	1	c16
....................Cacccuguagauccgaauuu........................................................................	1	1	c16
....................uacccuguagauccgaaGuu........................................................................	1	1	c16
....................uacccuguagaAccgaauuu........................................................................	1	1	c16
....................uCcccuguagauccgaauuu........................................................................	1	1	c16
....................uaccAuguagauccgaauuu........................................................................	1	1	c16
....................uacccuguagauccgaauuC........................................................................	1	1	c16
....................Aacccuguagauccgaauuu........................................................................	1	1	c16
....................uacccuguagauAcgaauuug.......................................................................	1	1	c16
....................Cacccuguagauccgaauuug.......................................................................	2	1	c16
....................uCcccuguagauccgaauuug.......................................................................	2	1	c16
....................uacccuguagCuccgaauuug.......................................................................	5	1	c16
....................uacccuguagauccgaauuuC.......................................................................	1	1	c16
....................Gacccuguagauccgaauuug.......................................................................	69	1	c16
....................uacccuCuagauccgaauuug.......................................................................	2	1	c16
....................uacccuguagauccUaauuug.......................................................................	1	1	c16
....................uacccuguagauccgaauuug.......................................................................	879	0	c16
....................uacUcuguagauccgaauuug.......................................................................	1	1	c16
....................uacccAguagauccgaauuug.......................................................................	1	1	c16
....................Aacccuguagauccgaauuug.......................................................................	3	1	c16
....................uacccuguaCauccgaauuug.......................................................................	1	1	c16
....................uacccuguagauccgaauuGg.......................................................................	1	1	c16
....................uacccuguagauccgaaGuug.......................................................................	1	1	c16
....................uacccuguagauccgaauuuU.......................................................................	3	1	c16
....................uacccuguagaucAgaauuug.......................................................................	1	1	c16
....................uaAccuguagauccgaauuug.......................................................................	2	1	c16
....................uacAcuguagauccgaauuug.......................................................................	6	1	c16
....................uacccuguagauccgaauuuCu......................................................................	18	1	c16
....................uacccuCuagauccgaauuugu......................................................................	15	1	c16
....................uacccuguagaCccgaauuugu......................................................................	4	1	c16
....................uacccuguagauccgaauuugA......................................................................	14	1	c16
....................uacccuguagaucAgaauuugu......................................................................	20	1	c16
....................uaccGuguagauccgaauuugu......................................................................	3	1	c16
....................uacccuguagauccgaGuuugu......................................................................	3	1	c16
....................uacAcuguagauccgaauuugu......................................................................	75	1	c16
....................uacccGguagauccgaauuugu......................................................................	4	1	c16
....................uacccuguagauccgaauuugu......................................................................	13304	0	c16
....................Aacccuguagauccgaauuugu......................................................................	77	1	c16
....................uacccuguagaucUgaauuugu......................................................................	4	1	c16
....................Gacccuguagauccgaauuugu......................................................................	1034	1	c16
....................uacccAguagauccgaauuugu......................................................................	6	1	c16
....................uaUccuguagauccgaauuugu......................................................................	1	1	c16
....................uacccuguagauccgaauuuUu......................................................................	5	1	c16
....................uacccuguagauccgaaAuugu......................................................................	2	1	c16
....................uacccuguagauUcgaauuugu......................................................................	1	1	c16
....................uacccuguagaGccgaauuugu......................................................................	2	1	c16
....................uaccUuguagauccgaauuugu......................................................................	2	1	c16
....................uacccuguagaucGgaauuugu......................................................................	2	1	c16
....................uacccugGagauccgaauuugu......................................................................	1	1	c16
....................uacccuguagauAcgaauuugu......................................................................	21	1	c16
....................uacccuguagauccgaauAugu......................................................................	7	1	c16
....................uacccuguagauccgaauuugC......................................................................	5	1	c16
```

```
                    Mature                Star
caauuuuagugcccuacaucuacccuguagauccgaauuuguuugaagugaggcgacaaauucgguucuagagagguuuguguggugcaugcacugcaacguuucaagucug
....................uacccuguagUuccgaauuugu......................................................................  1    1  c16
....................uacccuguagauccgGauuugu......................................................................  1    1  c16
....................uacccuguagauccUaauuugu......................................................................  10   1  c16
....................uaAccuguagauccgaauuugu......................................................................  9    1  c16
....................uacccCguagauccgaauuugu......................................................................  6    1  c16
....................uacccuguagauccgaauuGgu......................................................................  2    1  c16
....................Cacccuguagauccgaauuugu......................................................................  9    1  c16
....................uacccuguagauccgaUuuugu......................................................................  5    1  c16
....................uCcccuguagauccgaauuugu......................................................................  37   1  c16
....................uacccuguagauGcgaauuugu......................................................................  5    1  c16
....................uacUcuguagauccgaauuugu......................................................................  6    1  c16
....................uacccuguagaAccgaauuugu......................................................................  1    1  c16
....................uacccuguagauccgUauuugu......................................................................  2    1  c16
....................uacccuguagauccgaauuCgu......................................................................  1    1  c16
....................uacGcuguagauccgaauuugu......................................................................  8    1  c16
....................uacccuguagGuccgaauuugu......................................................................  2    1  c16
....................uacccuguaUauccgaauuugu......................................................................  4    1  c16
....................uUcccuguagauccgaauuugu......................................................................  1    1  c16
....................uacccuguagauccAaauuugu......................................................................  2    1  c16
....................uacccuAuagauccgaauuugu......................................................................  4    1  c16
....................uaccAuguagauccgaauuugu......................................................................  9    1  c16
....................uacccuguagCuccgaauuugu......................................................................  39   1  c16
....................uGcccuguagauccgaauuugu......................................................................  6    1  c16
....................uacccuguagauccgaaGuugu......................................................................  3    1  c16
....................uacccugAagauccgaauuugu......................................................................  8    1  c16
....................uacccuguUgauccgaauuugu......................................................................  1    1  c16
....................uacccuguGgauccgaauuugu......................................................................  3    1  c16
....................uacccuguagauccgaauuAgu......................................................................  12   1  c16
....................uacccuguagauccCaauuugu......................................................................  5    1  c16
....................uaGccuguagauccgaauuugu......................................................................  2    1  c16
....................uacccuguagauccgaaCuugu......................................................................  5    1  c16
....................uacccuguaCauccgaauuugu......................................................................  5    1  c16
....................uacccuguaAauccgaauuugu......................................................................  3    1  c16
....................uacccuguagauccgaauuugG......................................................................  4    1  c16
....................uacccuUuagauccgaauuugu......................................................................  6    1  c16
....................uacccuguagauccgaauGugu......................................................................  1    1  c16
....................uacccuguagauccgaauuuguG.....................................................................  10   1  c16
....................uacccuguagauccgaauuAguu.....................................................................  1    1  c16
....................uacccuguagauccgaauuuguu.....................................................................  192  0  c16
....................uacccuguagauccgaauuuguA.....................................................................  208  1  c16
....................uacccuguagCuccgaauuuguu.....................................................................  1    1  c16
....................Gacccuguagauccgaauuuguu.....................................................................  15   1  c16
....................uacccGguagauccgaauuuguu.....................................................................  1    1  c16
....................uacccuguagauccgaauuuguC.....................................................................  14   1  c16
....................Aacccuguagauccgaauuuguu.....................................................................  1    1  c16
....................uacccuAuagauccgaauuuguu.....................................................................  1    1  c16
....................uacAcuguagauccgaauuuguu.....................................................................  2    1  c16
....................uCcccuguagauccgaauuuguu.....................................................................  1    1  c16
....................Gacccuguagauccgaauuuguuu....................................................................  3    1  c16
....................uacccuguagauccgaauuuguAu....................................................................  6    1  c16
....................uacccuguagauccgaauuuguuu....................................................................  2    0  c16
....................uacccuguagauccgaauuuguCu....................................................................  2    1  c16
....................uacccuguagauccgaauuuguuA....................................................................  7    1  c16
....................uacccuguagauccgaauuuguuug...................................................................  3    0  c16
....................uacccuguagauccgaauuuguuuga..................................................................  2    0  c16
....................uacccuguagauccgaauuuguuugaag................................................................  1    0  c16
....................uacccuguagauccgaauuuguuugaagugaggcgac.......................................................  1    0  c16
.....................acccuguagaucUgaauu.........................................................................  1    1  c16
.....................Ucccuguagauccgaauu.........................................................................  1    1  c16
.....................acccuguagauccgaauA.........................................................................  1    1  c16
.....................acccuguagauAcgaauu.........................................................................  1    1  c16
.....................acccuguagauccgaauu.........................................................................  113  0  c16
.....................acccugAagauccgaauu.........................................................................  1    1  c16
.....................Ccccuguagauccgaauu.........................................................................  76   1  c16
.....................acccuguagauccgaauCu........................................................................  1    1  c16
.....................acccuguagCuccgaauuu........................................................................  1    1  c16
.....................acccuguUgauccgaauuu........................................................................  1    1  c16
.....................acccuguagauccgaauuu........................................................................  199  0  c16
.....................acccuguagauccgaauuC........................................................................  1    1  c16
.....................Ccccuguagauccgaauuu........................................................................  105  1  c16
```

Mature Star

```
caauuuuagugcccuacaucuacccuguagauccgaauuuguuugaagugaggcgacaaauucgguucuagagagguuuguguggugcaugcacugcaacguuucaagucug
.....................acccuguagauccgaauuG........................................................................	2	1	c16
.....................acccuguagauccgaauuug.......................................................................	646	0	c16
.....................acccuguagauccgaauuCg.......................................................................	1	1	c16
.....................acccuguagauccgaauuuA.......................................................................	2	1	c16
.....................acccuguagauccgaaAuug.......................................................................	1	1	c16
.....................acccuguagauAcgaauuug.......................................................................	1	1	c16
.....................acccuguagCuccgaauuug.......................................................................	1	1	c16
.....................acccuguagauccgaauuuC.......................................................................	2	1	c16
.....................acAcuguagauccgaauuug.......................................................................	3	1	c16
.....................acccuguagauccgaauuuU.......................................................................	1	1	c16
.....................acccuUuagauccgaauuug.......................................................................	1	1	c16
.....................accAuguagauccgaauuug.......................................................................	2	1	c16
.....................Ccccuguagauccgaauuug.......................................................................	418	1	c16
.....................acccuguagauccgaauuAg.......................................................................	1	1	c16
.....................acccuCuagauccgaauuug.......................................................................	2	1	c16
.....................acccuguagaucAgaauuug.......................................................................	4	1	c16
.....................acccuguUgauccgaauuugu......................................................................	1	1	c16
.....................acccAguagauccgaauuugu......................................................................	1	1	c16
.....................acccuguagauccgaauuuAu......................................................................	2	1	c16
.....................acAcuguagauccgaauuugu......................................................................	7	1	c16
.....................acccuguagauccgaauuugu......................................................................	1471	0	c16
.....................acccuguagauccgaauAugu......................................................................	1	1	c16
.....................acccuguagCuccgaauuugu......................................................................	4	1	c16
.....................acccuguagaAccgaauuugu......................................................................	1	1	c16
.....................acccuguagauccCaauuugu......................................................................	1	1	c16
.....................acccuguagauccgaaAuugu......................................................................	2	1	c16
.....................acccuguaCauccgaauuugu......................................................................	2	1	c16
.....................aUccuguagauccgaauuugu......................................................................	1	1	c16
.....................acUcuguagauccgaauuugu......................................................................	2	1	c16
.....................acccuguagauAcgaauuugu......................................................................	2	1	c16
.....................acccuguagaucAgaauuugu......................................................................	7	1	c16
.....................acccuguagauccAaauuugu......................................................................	1	1	c16
.....................Ccccuguagauccgaauuugu......................................................................	864	1	c16
.....................aAccuguagauccgaauuugu......................................................................	4	1	c16
.....................acccuguagauccgaauuugA......................................................................	1	1	c16
.....................accAuguagauccgaauuugu......................................................................	2	1	c16
.....................acccuguagauccgaauuCgu......................................................................	1	1	c16
.....................acccuguagauccgaauuuCu......................................................................	1	1	c16
.....................acccugGagauccgaauuugu......................................................................	1	1	c16
.....................acccugAagauccgaauuugu......................................................................	1	1	c16
.....................acccuguagauccgaauuugG......................................................................	1	1	c16
.....................acccuguagauccgaauuuUu......................................................................	2	1	c16
.....................acccuguagauccgaUuuugu......................................................................	1	1	c16
.....................acccuCuagauccgaauuugu......................................................................	7	1	c16
.....................acccuguagauccgaauuuguu.....................................................................	4761	0	c16
.....................acccuCuagauccgaauuuguu.....................................................................	14	1	c16
.....................acGcuguagauccgaauuuguu.....................................................................	1	1	c16
.....................acAcuguagauccgaauuuguu.....................................................................	18	1	c16
.....................acccuguagaCccgaauuuguu.....................................................................	1	1	c16
.....................acccuguagaucGgaauuuguu.....................................................................	2	1	c16
.....................acccuguagauccgaauuuCuu.....................................................................	7	1	c16
.....................acccuguagauccCaauuuguu.....................................................................	4	1	c16
.....................acccuguagaucUgaauuuguu.....................................................................	4	1	c16
.....................Ucccuguagauccgaauuuguu.....................................................................	1	1	c16
.....................acccCguagauccgaauuuguu.....................................................................	2	1	c16
.....................acUcuguagauccgaauuuguu.....................................................................	4	1	c16
.....................acccuguagauccgaaCuuguu.....................................................................	1	1	c16
.....................acccuguaAauccgaauuuguu.....................................................................	1	1	c16
.....................acccugAagauccgaauuuguu.....................................................................	2	1	c16
.....................acccuguagaucAgaauuuguu.....................................................................	18	1	c16
.....................acccuguagauccgaauuugCu.....................................................................	3	1	c16
.....................acccuguagauccgaauuGguu.....................................................................	1	1	c16
.....................aUccuguagauccgaauuuguu.....................................................................	1	1	c16
.....................acccGguagauccgaauuuguu.....................................................................	1	1	c16
.....................acccuguagauAcgaauuuguu.....................................................................	10	1	c16
.....................acccuguUgauccgaauuuguu.....................................................................	1	1	c16
.....................acccuguagauccgaauuCguu.....................................................................	1	1	c16
.....................accUuguagauccgaauuuguu.....................................................................	6	1	c16
.....................acccuguagCuccgaauuuguu.....................................................................	12	1	c16
.....................acccuguagauccgaauuuguG.....................................................................	3	1	c16
```

```
                     Mature                              Star
caauuuuagugcccuacaucuacccuguagauccgaauuuguuugaagugaggcgacaaauucgguucuagagagguuugugguggugcaugcacugcaacguuucaagucug
.....................acccuguaCauccgaauuuguu.....................................................................  4     1  c16
.....................acccuUuagauccgaauuuguu.....................................................................  3     1  c16
.....................aGccuguagauccgaauuuguu.....................................................................  1     1  c16
.....................acccuguagauccgaaGuuguu.....................................................................  2     1  c16
.....................acccuguagauccUaauuuguu.....................................................................  3     1  c16
.....................acccuguagauccgaauuuguA.....................................................................  15    1  c16
.....................acccuguagauccgaaAuuguu.....................................................................  4     1  c16
.....................acccuguagauccgaauuugGu.....................................................................  1     1  c16
.....................acccuguGgauccgaauuuguu.....................................................................  2     1  c16
.....................acccuguagauccgaauuuAuu.....................................................................  1     1  c16
.....................accAuguagauccgaauuuguu.....................................................................  10    1  c16
.....................acccuAuagauccgaauuuguu.....................................................................  1     1  c16
.....................aAccuguagauccgaauuuguu.....................................................................  2     1  c16
.....................acccuguagauccgaauuuUuu.....................................................................  4     1  c16
.....................Gcccuguagauccgaauuuguu.....................................................................  5     1  c16
.....................acccuguagauccgaauuuguC.....................................................................  2     1  c16
.....................acccuguagaAccgaauuuguu.....................................................................  1     1  c16
.....................Ccccuguagauccgaauuuguu.....................................................................  2656  1  c16
.....................acccAguagauccgaauuuguu.....................................................................  5     1  c16
.....................acccuguagauccgaauuugAu.....................................................................  3     1  c16
.....................acccuguagauccgaauAuguu.....................................................................  4     1  c16
.....................acccuguaUauccgaauuuguu.....................................................................  1     1  c16
.....................acccuguagauccUaauuuguuu....................................................................  1     1  c16
.....................aUccuguagauccgaauuuguuu....................................................................  1     1  c16
.....................acAcuguagauccgaauuuguuu....................................................................  1     1  c16
.....................acccuguaCauccgaauuuguuu....................................................................  1     1  c16
.....................acccuguagauccgaauuuguuu....................................................................  169   0  c16
.....................acccuguUgauccgaauuuguuu....................................................................  1     1  c16
.....................acccuguagauccgaauuuguuA....................................................................  196   1  c16
.....................acccuguagauccgaUuuuguuu....................................................................  1     1  c16
.....................acccCguagauccgaauuuguuu....................................................................  1     1  c16
.....................acccuguagCuccgaauuuguuu....................................................................  1     1  c16
.....................Ccccuguagauccgaauuuguuu....................................................................  84    1  c16
.....................aAccuguagauccgaauuuguuu....................................................................  1     1  c16
.....................acccuguagauccgaauuuguuG....................................................................  8     1  c16
.....................acccuguagauccgaauuuguuC....................................................................  9     1  c16
.....................acccuguagauccgaauuuguuAg...................................................................  1     1  c16
.....................acccuguagauccgaauuuguuuA...................................................................  1     1  c16
.....................acccuguagauccgaauuuguuug...................................................................  2     0  c16
.....................acccuguagauccgaauuuguuuU...................................................................  10    1  c16
.....................acccuguagauccgaauuuguuuga..................................................................  2     0  c16
.....................acccuguagauccgaauuuguuuAa..................................................................  2     1  c16
.....................acccuguagauccgaauuuguuuUa..................................................................  1     1  c16
.....................Ccccuguagauccgaauuuguuuga..................................................................  1     1  c16
.....................acccuguagauccgaauuuguuugG..................................................................  1     1  c16
.....................Ccccuguagauccgaauuuguuugaagugaggcg..........................................................  1     1  c16
.....................acccuguagauccgaauuuguuugaagugaggcga.........................................................  3     0  c16
.....................Ccccuguagauccgaauuuguuugaagugaggcga.........................................................  3     1  c16
......................cccuguagauccgaauuu........................................................................  1     0  c16
......................cccuguagauccgaauuug.......................................................................  3     0  c16
......................cccuguagaucAgaauuugu......................................................................  1     1  c16
......................cccuguagauccgaauuugu......................................................................  10    0  c16
......................Gccuguagauccgaauuuguu.....................................................................  1     1  c16
......................cccuguagauccgaauuuguu.....................................................................  8     0  c16
......................cAcuguagauccgaauuuguu.....................................................................  1     1  c16
......................cccuguagauccgaauuuguuu....................................................................  1     0  c16
.......................ccuguagauccgaauuug.......................................................................  2     0  c16
.......................ccuguagauccgaauuugu......................................................................  19    0  c16
.......................ccuguagauccgUauuugu......................................................................  1     1  c16
.......................ccuguagauccgaauuuguu.....................................................................  24    0  c16
........................cuCuagauccgaauuugu......................................................................  1     1  c16
........................cuguagauccgaauuugu......................................................................  6     0  c16
........................cuguagauccgaauuuguu.....................................................................  9     0  c16
........................Uuguagauccgaauuuguu.....................................................................  1     1  c16
........................cuguagauccgaauuuguuA....................................................................  1     1  c16
.........................uguagauccgaauuuguA.....................................................................  1     1  c16
.........................uguagauccgaauuuguu.....................................................................  11    0  c16
.........................uguagauccgaauuuguuu....................................................................  1     0  c16
.........................uguagauccgaauuuguuA....................................................................  1     1  c16
...........................................ugaagugaggcgacaaauucgguucuagagagguuu.................................  1     0  c16
```

```
                                        Mature                            Star
caauuuuagugcccuacaucuacccuguagauccgaauuuguuugaagugaggcgacaaauucgguucuagagagguuuguguggugcaugcacugcaacguuucaagucug
..................................................aggcgacaaauucgguucuagagagguuu.................................   1     0   c16
....................................................gGgacaaauucgguucuagagagguuu.................................   1     1   c16
......................................................gGcaaauucgguucuagagaggu...................................   1     1   c16
.......................................................Ucaaauucgguucuagagagguuu.................................   1     1   c16
.......................................................aAaaauucgguucuagagagguuu.................................   2     1   c16
........................................................caaauucgguucuagaga......................................   75    0   c16
........................................................caaauucgguuAuagaga......................................   1     1   c16
........................................................caaauucgguAcuagaga......................................   1     1   c16
........................................................cCaauucgguucuagagag.....................................   1     1   c16
........................................................caaauucggGucuagagag.....................................   1     1   c16
........................................................caaauucgCuucuagagag.....................................   2     1   c16
........................................................caaauuAgguucuagagag.....................................   1     1   c16
........................................................caaauucgguucuaCagag.....................................   1     1   c16
........................................................caaauucgguucuagagag.....................................   341   0   c16
........................................................caaaGucgguucuagagag.....................................   1     1   c16
........................................................caaauucCguucuagagag.....................................   1     1   c16
........................................................caaauucgguuAuagagag.....................................   2     1   c16
........................................................caaauucgguucuagagaA.....................................   5     1   c16
........................................................caaauucgguucuagaCag.....................................   1     1   c16
........................................................caaaAucgguucuagagag.....................................   1     1   c16
........................................................caaauucgguucuagagagU....................................   2     1   c16
........................................................caaauucgUuucuagagagg....................................   2     1   c16
........................................................caaauucUguucuagagagg....................................   1     1   c16
........................................................caaauucgguucuagagagA....................................   1     1   c16
........................................................caaauucgguuAuagagagg....................................   5     1   c16
........................................................caaauucgguucuagagCgg....................................   1     1   c16
........................................................caaauucgguucuagagagg....................................   744   0   c16
........................................................caaauuUgguucuagagagg....................................   2     1   c16
........................................................caaauucgguucuagagaCg....................................   3     1   c16
........................................................caaauucCguucuagagagg....................................   1     1   c16
........................................................caaaAucgguucuagagagg....................................   3     1   c16
........................................................caaauucgguucuagagGgg....................................   1     1   c16
........................................................Aaaauucgguucuagagagg....................................   1     1   c16
........................................................caaauucgguucuagagaUg....................................   1     1   c16
........................................................caaauucgguucuagUgagg....................................   1     1   c16
........................................................caaauucggAucuagagaggu...................................   2     1   c16
........................................................caaauucgguAcuagagaggu...................................   1     1   c16
........................................................caaauucggGucuagagaggu...................................   1     1   c16
........................................................caaauucgguucuagagaggA...................................   4     1   c16
........................................................caaauucAguucuagagaggu...................................   1     1   c16
........................................................caaauucCguucuagagaggu...................................   1     1   c16
........................................................caaauucgUuucuagagaggu...................................   1     1   c16
........................................................caaauucgguucuagagagAu...................................   1     1   c16
........................................................caaauucgguucuGgagaggu...................................   2     1   c16
........................................................caaauucUguucuagagaggu...................................   4     1   c16
........................................................caaauucgguGcuagagaggu...................................   2     1   c16
........................................................caaauuAgguucuagagaggu...................................   4     1   c16
........................................................caaauucgguucuagagaggu...................................   888   0   c16
........................................................caaauucgguCcuagagaggu...................................   1     1   c16
........................................................caaauucgguuAuagagaggu...................................   1     1   c16
........................................................caaaAucgguucuagagaggu...................................   3     1   c16
........................................................caaauucgguucuagagaggG...................................   1     1   c16
........................................................caaauucgguucuagagagCu...................................   2     1   c16
........................................................caaauucgguucCagagaggu...................................   1     1   c16
........................................................cGaauucgguucuagagaggu...................................   1     1   c16
........................................................caaauucgguucuagagagguA..................................   14    1   c16
........................................................Aaaauucgguucuagagagguu..................................   2     1   c16
........................................................caaauucgguuAuagagagguu..................................   1     1   c16
........................................................caaauucgguucuagagagguG..................................   2     1   c16
........................................................caaauucgguucuagaCagguu..................................   1     1   c16
........................................................caaauucgguucuagagagUuu..................................   4     1   c16
........................................................caaauucgguucAagagagguu..................................   2     1   c16
........................................................caaauucgCuucuagagagguu..................................   1     1   c16
........................................................caaaAucgguucuagagagguu..................................   1     1   c16
........................................................caaauucCguucuagagagguu..................................   2     1   c16
........................................................caaauucgguucuagagagCuu..................................   1     1   c16
........................................................caaauucgguucuagagagguu..................................   1314  0   c16
........................................................caaauucggAucuagagagguu..................................   2     1   c16
........................................................caaauucUguucuagagagguu..................................   1     1   c16
........................................................caaauucgguucuaUagagguu..................................   1     1   c16
```

Mature / Star			
caauuuuagugcccuacaucuacccuguagauccgaauuuguuugaagugaggcgacaaauucgguucuagagagguuuguguggugcaugcacugcaacguuucaagucug			
....caaauucgguucuGgagagguu..................................	1	1	c16
....caaauucgguucuagaAagguu..................................	1	1	c16
....caaauucgguucCagagagguu..................................	2	1	c16
....caaauucgUuucuagagagguu..................................	2	1	c16
....caaauucgguAcuagagagguu..................................	1	1	c16
....caaauucgguucuagagaCguu..................................	3	1	c16
....Uaaauucgguucuagagagguu..................................	1	1	c16
....caaauucgguucuagagagguC..................................	4	1	c16
....caaauuAgguucuagagagguu..................................	2	1	c16
....caaauucgguucuagaUagguu..................................	1	1	c16
....caaauucgguGcuagagagguu..................................	1	1	c16
....caaauucgguucuagagaUguuu.................................	7	1	c16
....caaauucgguucuagCgagguuu.................................	1	1	c16
....caaauucgguucuagagGgguuu.................................	3	1	c16
....caaauucgguucuagagagUuuu.................................	1	1	c16
....caaauucgguucuagaAagguuu.................................	1	1	c16
....caaauucgguucuagagaggCuu.................................	3	1	c16
....caaCuucgguucuagagagguuu.................................	1	1	c16
....caaauucgguuAuagagagguuu.................................	10	1	c16
....caaauucgguucuagagagguuC.................................	2	1	c16
....Uaaauucgguucuagagagguuu.................................	2	1	c16
....caaauucgUuucuagagagguuu.................................	4	1	c16
....caaauuGgguucuagagagguuu.................................	1	1	c16
....caaauucgguCcuagagagguuu.................................	1	1	c16
....caaauucgguucuaCagagguuu.................................	5	1	c16
....caaauucgguucuagagaggGuu.................................	4	1	c16
....caaauucgguucCagagagguuu.................................	4	1	c16
....caaauucgguucuagagaCguuu.................................	8	1	c16
....cGaauucgguucuagagagguuu.................................	1	1	c16
....caaauucgguucuagaCagguuu.................................	6	1	c16
....caaauucgguuGuagagagguuu.................................	1	1	c16
....caaauucgguucuagagagguuG.................................	2	1	c16
....caaauucgguucuUgagagguuu.................................	2	1	c16
....caaauucgguucuGgagagguuu.................................	2	1	c16
....caUauucgguucuagagagguuu.................................	1	1	c16
....caaaAucgguucuagagagguuu.................................	10	1	c16
....caaauucgguucuagagCgguuu.................................	1	1	c16
....caaauucgguucGagagagguuu.................................	2	1	c16
....caaauucAguucuagagagguuu.................................	1	1	c16
....caaauucggAucuagagagguuu.................................	4	1	c16
....caaauucgguucuagaUagguuu.................................	4	1	c16
....caaauucgguucuagagUgguuu.................................	2	1	c16
....caaauucgguucuagagagguuA.................................	12	1	c16
....caaauuUgguucuagagagguuu.................................	1	1	c16
....caaauucgguucuagagagguuu.................................	7155	0	c16
....caaauucCguucuagagagguuu.................................	9	1	c16
....caaaCucgguucuagagagguuu.................................	3	1	c16
....caaauucgCuucuagagagguuu.................................	16	1	c16
....caaauucgguucuagagaAguuu.................................	2	1	c16
....caaauucgguucuagGgagguuu.................................	2	1	c16
....cCaauucgguucuagagagguuu.................................	1	1	c16
....Aaaauucgguucuagagagguuu.................................	6	1	c16
....caaauucgguucuagagagguCu.................................	3	1	c16
....caaauucgguucuaUagagguuu.................................	7	1	c16
....caaauucgguucuagagagguAu.................................	7	1	c16
....caaaGucgguucuagagagguuu.................................	3	1	c16
....caaauucgguGcuagagagguuu.................................	1	1	c16
....caaauucgAuucuagagagguuu.................................	1	1	c16
....caaauuAgguucuagagagguuu.................................	18	1	c16
....caaauAcgguucuagagagguuu.................................	6	1	c16
....caaauucggGucuagagagguuu.................................	10	1	c16
....caaGuucgguucuagagagguuu.................................	1	1	c16
....caGauucgguucuagagagguuu.................................	1	1	c16
....caaauucgguucuagagaggAuu.................................	5	1	c16
....caaauucgguucAagagagguuu.................................	2	1	c16
....caaauucgguucuagagagCuuu.................................	22	1	c16
....caaauucgguucuagUgagguuu.................................	2	1	c16
....caaauucgguAcuagagagguuu.................................	8	1	c16
....caaauucUguucuagagagguuu.................................	8	1	c16
....caaauucgguucuagagagguuug................................	191	0	c16

```
                                         Mature                                    Star
caauuuuagugcccuacaucuacccuguagauccgaauuuguuugaagugaggcgacaaauucgguucuagagagguuugugguggugcaugcacugcaacguuucaagucug
........................................................caaauucgguucuagagagCuuug................................   1     1   c16
........................................................caaauucggAucuagagagguuug................................   1     1   c16
........................................................caaauuAgguucuagagagguuug................................   1     1   c16
........................................................caaauucgguCcuagagagguuug................................   1     1   c16
........................................................caaauucAguucuagagagguuug................................   1     1   c16
........................................................caaauucgguucuagagagguuuC................................   14    1   c16
........................................................caaauucgguucuagagagguuuU................................   844   1   c16
........................................................caaauucgguucuagagagguuGg................................   1     1   c16
........................................................caaauucgguucuagagagguuuA................................   75    1   c16
........................................................caaauucgguucuagagagguuugu...............................   41    0   c16
........................................................caaauucgguucuagagagguuuAu...............................   7     1   c16
........................................................caaauucgguucuagagagguuugA...............................   5     1   c16
........................................................caaauucgguucuagagagguuuUu...............................   102   1   c16
........................................................caaauucgguucuagagagguuuCu...............................   7     1   c16
........................................................caaauucgguucuagagagguuugG...............................   4     1   c16
........................................................caaauucgguucuagagagguuuguA..............................   2     1   c16
........................................................caaauucgguucuagagagguuuguU..............................   2     1   c16
........................................................caaauucgguucuagagagguuugug..............................   1     0   c16
........................................................caaauucgguucuagagagguuuguUu.............................   1     1   c16
........................................................caaauucgguucuagagagguuuCugugg...........................   1     1   c16
.........................................................aaauucgguucuagagag.....................................   33    0   c16
.........................................................Caauucgguucuagagag.....................................   2     1   c16
.........................................................Caauucgguucuagagagg....................................   9     1   c16
.........................................................aaauucgguucuagagagg....................................   138   0   c16
.........................................................aaauucgguucuagagaggu...................................   159   0   c16
.........................................................Caauucgguucuagagaggu...................................   9     1   c16
.........................................................aaauuAgguucuagagaggu...................................   1     1   c16
.........................................................aaauucgguucuagagaCgu...................................   1     1   c16
.........................................................aaauucgUuucuagagaggu...................................   1     1   c16
.........................................................aaauuUgguucuagagagguu..................................   1     1   c16
.........................................................aaauucAguucuagagagguu..................................   1     1   c16
.........................................................Caauucgguucuagagagguu..................................   16    1   c16
.........................................................aaauucCguucuagagagguu..................................   1     1   c16
.........................................................aaauucgguAcuagagagguu..................................   1     1   c16
.........................................................aaauucgguucuagagaUguu..................................   1     1   c16
.........................................................aaauucgguuAuagagagguu..................................   1     1   c16
.........................................................aaauucgguucuagagagguu..................................   304   0   c16
.........................................................aaauucgguucuaCagagguu..................................   1     1   c16
.........................................................aaauucgguucuagagagguA..................................   1     1   c16
.........................................................aaauuAgguucuagagagguu..................................   1     1   c16
.........................................................aaauucgguucuagagaggGu..................................   1     1   c16
.........................................................aaaAucgguucuagagagguu..................................   1     1   c16
.........................................................aaauucgguucuagagaggAuu.................................   3     1   c16
.........................................................Caauucgguucuagagagguuu.................................   92    1   c16
.........................................................aaauGcgguucuagagagguuu.................................   1     1   c16
.........................................................aaauCcgguucuagagagguuu.................................   1     1   c16
.........................................................aaauucgguucuagagagguAu.................................   2     1   c16
.........................................................aaauuAgguucuagagagguuu.................................   6     1   c16
.........................................................aaauucgCuucuagagagguuu.................................   4     1   c16
.........................................................aaauAcgguucuagagagguuu.................................   2     1   c16
.........................................................aaauucgguucuagagaggGuu.................................   2     1   c16
.........................................................aaauucgguucuagaUagguuu.................................   1     1   c16
.........................................................aaauucggGucuagagagguuu.................................   4     1   c16
.........................................................aaauucgguucuaCagagguuu.................................   1     1   c16
.........................................................aaauucgguucuagagagCuuu.................................   4     1   c16
.........................................................aaauucgguucuagagGgguuu.................................   2     1   c16
.........................................................aaauucgguAcuagagagguuu.................................   1     1   c16
.........................................................aaauucgguucCagagagguuu.................................   1     1   c16
.........................................................aaauucgguucuagagagAuuu.................................   2     1   c16
.........................................................aaaCucgguucuagagagguuu.................................   1     1   c16
.........................................................aaauucgguucuagagagguuC.................................   2     1   c16
.........................................................aaauucgguucuagagaUguuu.................................   1     1   c16
.........................................................aaauucggAucuagagagguuu.................................   1     1   c16
.........................................................aaauucgguucuagagagguuu.................................   1824  0   c16
.........................................................aaauucgguucuagagaCguuu.................................   1     1   c16
.........................................................aaauucgguucuagagagguuG.................................   1     1   c16
.........................................................aaauucgguuUuagagagguuu.................................   2     1   c16
.........................................................aaauucgguucuagagagUuuu.................................   2     1   c16
.........................................................aaauucgguucuagagCgguuu.................................   3     1   c16
.........................................................aaauucgguucuaUagagguuu.................................   2     1   c16
```

```
                     Mature                              Star
caauuuuagugcccuacaucuacccuguagauccgaauuuguuugaagugaggcgacaaauucgguucuagagagguuugugguggugcaugcacugcaacguuucaagucug
.........................................................aaauucgguucuagagaAguuu.................................	1	1	c16
.........................................................aaaAucgguucuagagagguuu.................................	1	1	c16
.........................................................aaauucUguucuagagagguuu.................................	3	1	c16
.........................................................aaauucgguGcuagagagguuu.................................	3	1	c16
.........................................................aaauucgguucGagagagguuu.................................	1	1	c16
.........................................................aaauucgguucuagagagguuA.................................	4	1	c16
.........................................................aaauucgguucAagagagguuu.................................	1	1	c16
.........................................................aaauucgguucuagaCagguuu.................................	3	1	c16
.........................................................aaauucgguuAuagagagguuu.................................	2	1	c16
.........................................................aaauucgguucuagagagguCu.................................	3	1	c16
.........................................................aaauucgguucuagaUagguuug................................	2	1	c16
.........................................................Caauucgguucuagagagguuug................................	54	1	c16
.........................................................aaauucgguucuagagagguuug................................	986	0	c16
.........................................................aaauucggAucuagagagguuug................................	1	1	c16
.........................................................aaauucgguuUuagagagguuug................................	1	1	c16
.........................................................aaauucgguucuagagagguuuC................................	16	1	c16
.........................................................aaauucgguucuagagagguuuA................................	67	1	c16
.........................................................aaauucgUuucuagagagguuug................................	1	1	c16
.........................................................aaauucgguAcuagagagguuug................................	1	1	c16
.........................................................aaauucAguucuagagagguuug................................	1	1	c16
.........................................................aaauucgguucuagagagguuuU................................	761	1	c16
.........................................................aaUuucgguucuagagagguuug................................	1	1	c16
.........................................................aaauucgCuucuagagagguuug................................	1	1	c16
.........................................................aaauucgguucuagagaCguuug................................	4	1	c16
.........................................................aaauucgguucuagagagguuGg................................	2	1	c16
.........................................................aaauucggGucuagagagguuug................................	1	1	c16
.........................................................aaauucgguucuagagagUuuug................................	2	1	c16
.........................................................aaaCucgguucuagagagguuug................................	1	1	c16
.........................................................aaauuAgguucuagagagguuug................................	2	1	c16
.........................................................aaauucgguucuagagaUguuug................................	1	1	c16
.........................................................aaauucCguucuagagagguuug................................	1	1	c16
.........................................................aaauucgguucuUgagagguuug................................	2	1	c16
.........................................................aaauucgguucuagagagguuuAu...............................	8	1	c16
.........................................................aaauucgguucuagagagguuugC...............................	3	1	c16
.........................................................aaauucgguucuagagagguuugG...............................	1	1	c16
.........................................................aaauucgguucuagagagguuuUu...............................	90	1	c16
.........................................................aaauucggGucuagagagguuugu...............................	1	1	c16
.........................................................aaauucgguucuagagagguuugu...............................	144	0	c16
.........................................................aaauucgCuucuagagagguuugu...............................	1	1	c16
.........................................................Caauucgguucuagagagguuugu...............................	5	1	c16
.........................................................aaauucgguucuagagagguuugA...............................	11	1	c16
.........................................................aaauucgguucuagagagguuuguU..............................	6	1	c16
.........................................................aaauucgguucuagagagguuuguA..............................	4	1	c16
.........................................................aaauucgguucuagagagguuuguUu.............................	4	1	c16
.........................................................aaauucgguucuagagagguuuguAu.............................	4	1	c16
..........................................................aauucgguucuagagagg....................................	1	0	c16
..........................................................aauucgguucuagagaggu...................................	1	0	c16
..........................................................aauucgguucuagagagguu..................................	3	0	c16
..........................................................aauucgguucuagagagguuu.................................	6	0	c16
..........................................................Cauucgguucuagagagguuu.................................	1	1	c16
..........................................................aauucgguucuagagagguuuA................................	2	1	c16
..........................................................aauucgguucuagagagguuuU................................	3	1	c16
..........................................................aauucgguucuagagagguuug................................	3	0	c16
..........................................................aauucgguucuagagagguuugu...............................	2	0	c16
..........................................................aauucgguucuagagagguuuUu...............................	6	1	c16
...........................................................auucgguucuagagagUu...................................	1	1	c16
...........................................................auucgguucuagCgaggu...................................	1	1	c16
...........................................................auucgguucuagagaggu...................................	42	0	c16
...........................................................Cuucgguucuagagaggu...................................	8	1	c16
...........................................................auucgguucuagagagguu..................................	88	0	c16
...........................................................Cuucgguucuagagagguu..................................	12	1	c16
...........................................................auucgguCcuagagagguu..................................	1	1	c16
...........................................................auucgCuucuagagagguu..................................	1	1	c16
...........................................................auucgguucuagagagguA..................................	1	1	c16
...........................................................auuAgguucuagagagguu..................................	1	1	c16
...........................................................Guucgguucuagagagguuu.................................	1	1	c16
...........................................................auucgguucuagagagguuA.................................	1	1	c16
...........................................................auucgguuAuagagagguuu.................................	2	1	c16
...........................................................auucgguucuagagagguuG.................................	1	1	c16
...........................................................auucgguucuagagaggGuu.................................	1	1	c16
```

```
                     Mature                Star
caauuuuagugcccuacaucuacccuguagauccgaauuuguuugaagugaggcgacaaauucgguucuagagagguuugugugugcaugcacugcaacguuucaagucug
...........................................................auucgguucCagagagguuu.................................  1    1    c16
...........................................................auucgCuucuagagagguuu.................................  1    1    c16
...........................................................auucgguucuagagagCuuu.................................  1    1    c16
...........................................................auucgguucuagagaggAuu.................................  1    1    c16
...........................................................auuAgguucuagagagguuu.................................  1    1    c16
...........................................................auucgguucuagagagguuu.................................  456  0    c16
...........................................................auucgguGcuagagagguuu.................................  1    1    c16
...........................................................Cuucgguucuagagagguuu.................................  64   1    c16
...........................................................Cuucgguucuagagagguuug................................  1    1    c16
...........................................................auucgguucuagagagguuug................................  20   0    c16
...........................................................auucgguucuagagagguuuU................................  44   1    c16
...........................................................auucgguucuagagagguuuA................................  10   1    c16
...........................................................auucgguucuagagagguuuAu...............................  1    1    c16
...........................................................auucgguucuagagagguuuUu...............................  8    1    c16
...........................................................auucgguucuagagagguuugA...............................  2    1    c16
...........................................................auucgguucuagagagguuugu...............................  4    0    c16
...........................................................auucgguucuagagagguuugGg..............................  1    1    c16
............................................................uucgguucuagagagCuu..................................  1    1    c16
............................................................uucgguucuagagagguu..................................  6    0    c16
............................................................Gucgguucuagagagguu..................................  1    1    c16
............................................................Gucgguucuagagagguuu.................................  4    1    c16
............................................................uucgguucuagagagCuuu.................................  1    1    c16
............................................................uucgguucuagagagguuu.................................  46   0    c16
............................................................uuAgguucuagagagguuu.................................  1    1    c16
............................................................uucgguucuagagagguuuC................................  1    1    c16
............................................................uucgguucuagagagguuug................................  13   0    c16
............................................................uucgguucuagagagguuuU................................  11   1    c16
............................................................uucgguucuagagagguuuA................................  1    1    c16
............................................................uucgguucuagagCgguuugu...............................  1    1    c16
............................................................uucgguucuagagagguuugA...............................  1    1    c16
............................................................uucgguucuagagagguuugu...............................  2    0    c16
.............................................................Gcgguucuagagagguuu.................................  1    1    c16
.............................................................ucgguucuagagagguuu.................................  11   0    c16
.............................................................ucgguucuagagagguuug................................  1    0    c16
.............................................................Gcgguucuagagagguuug................................  2    1    c16
.............................................................ucgguucuagagagguuuU................................  5    1    c16
..............................................................cgguucuagagagguuuU................................  1    1    c16

....................uacccuguagauccgaau..........................................................................  10   0    c04
....................uacccuguagauccgaauu.........................................................................  18   0    c04
....................uacccuguagauccgaauuu........................................................................  13   0    c04
....................Gacccuguagauccgaauuu........................................................................  1    1    c04
....................uacccuguagauccgaauuug.......................................................................  15   0    c04
....................uacccuguagauccgaauuuU.......................................................................  1    1    c04
....................uacccuguagauccgaauuugu......................................................................  335  0    c04
....................Aacccuguagauccgaauuugu......................................................................  1    1    c04
....................Gacccuguagauccgaauuugu......................................................................  17   1    c04
....................uacccuguagaucAgaauuugu......................................................................  2    1    c04
....................uaAccuguagauccgaauuugu......................................................................  1    1    c04
....................uacAcuguagauccgaauuugu......................................................................  1    1    c04
....................uacccuCuagauccgaauuugu......................................................................  1    1    c04
....................uacccuguagauccgaauuuguC.....................................................................  1    1    c04
....................uacccuguagauccgaauuuguA.....................................................................  3    1    c04
....................Gacccuguagauccgaauuuguu.....................................................................  1    1    c04
....................uacccuguagauccgaauuuguu.....................................................................  2    0    c04
....................uacccuguagauccgaauuuguAu....................................................................  1    1    c04
....................uacccuguagauccgaauuuguuu....................................................................  1    0    c04
.....................acccuguagauccgaauu.........................................................................  9    0    c04
.....................acccuguagaucAgaauu.........................................................................  1    1    c04
.....................Ccccuguagauccgaauu.........................................................................  2    1    c04
.....................Ccccuguagauccgaauuu........................................................................  1    1    c04
.....................acccuguagauccgaauuu........................................................................  9    0    c04
.....................Ccccuguagauccgaauuug.......................................................................  2    1    c04
.....................acccuguagauccgaauuug.......................................................................  8    0    c04
.....................acccuguagauccgaauuugu......................................................................  33   0    c04
.....................aGccuguagauccgaauuugu......................................................................  1    1    c04
.....................acccuguagauccgaaCuugu......................................................................  1    1    c04
.....................Ccccuguagauccgaauuugu......................................................................  7    1    c04
.....................acccuguagauccgaauuugAu.....................................................................  1    1    c04
.....................acccuguagauUcgaauuuguu.....................................................................  1    1    c04
```

```
                     Mature                              Star
caauuuuagugcccuacaucuacccuguagauccgaauuuguuugaagugaggcgacaaauucgguucuagagagguuuguguggugcaugcacugcaacguuucaagucug
.....................acccuguagauccgaauuuAuu.....................................................................	1	1	c04
.....................acccuguagauccgGauuuguu.....................................................................	1	1	c04
.....................Ccccuguagauccgaauuuguu.....................................................................	48	1	c04
.....................acccuguagauccgaauuuguA.....................................................................	1	1	c04
.....................acccuguagauccgaauuuguu.....................................................................	165	0	c04
.....................acccuguagauccgaauuuguuC....................................................................	1	1	c04
.....................acccuguagauccgaauuuguuG....................................................................	1	1	c04
.....................acccuguagauccgaauuuguuA....................................................................	2	1	c04
.....................acccuguagauccgaauuuguuu....................................................................	4	0	c04
.....................acccuguagauccgaauuuguuuU...................................................................	1	1	c04
.....................acccuguagauccgaauuuguuGg...................................................................	1	1	c04
.....................Ccccuguagauccgaauuuguuugaagugaggcga........................................................	1	1	c04
......................cccuguagauccgaauuugu......................................................................	1	0	c04
......................cccuguagauccgaauuuguu.....................................................................	1	0	c04
.......................ccuguagauccgaauuugu......................................................................	1	0	c04
........................cuguagauccgaauuugu......................................................................	1	0	c04
.........................uguagauccgaauuuguu.....................................................................	1	0	c04
........................................................caaauucgguucuagaga......................................	3	0	c04
........................................................caaauucgguucuagagag.....................................	7	0	c04
........................................................caaauucgguucuagagagg....................................	14	0	c04
........................................................caaauucgguucuagagaggu...................................	36	0	c04
........................................................caaauucgguucuagagagguu..................................	34	0	c04
........................................................caaauucUguucuagagagguuu.................................	1	1	c04
........................................................Aaaauucgguucuagagagguuu.................................	1	1	c04
........................................................caaauucgguucuagagagguuC.................................	2	1	c04
........................................................caaauucggGucuagagagguuu.................................	1	1	c04
........................................................caaauucgguucuagagagguuu.................................	172	0	c04
........................................................caaauucgguucuagagagguuug................................	7	0	c04
........................................................caaauucgguucuagagagguuuA................................	1	1	c04
........................................................caaauucgguucuagagagguuuU................................	19	1	c04
........................................................caaauucgguucuagagagguuugA...............................	1	1	c04
........................................................caaauucgguucuagagagguuugu...............................	1	0	c04
........................................................caaauucgguucuagagagguuuUu...............................	1	1	c04
.........................................................aaauucgguucuagagag.....................................	1	0	c04
.........................................................aaauucgguucuagagagg....................................	4	0	c04
.........................................................aaauucgguucuagagaggu...................................	1	0	c04
.........................................................aaauucgguucuagagaggG...................................	1	1	c04
.........................................................aaauucgguucuagagagguu..................................	10	0	c04
.........................................................aaauucgguucuagagagUuuu.................................	1	1	c04
.........................................................aaauucgguucuagagagguuu.................................	29	0	c04
.........................................................aaauucgguucuagagagguuug................................	16	0	c04
.........................................................aaauucgguucuagagagguuuU................................	14	1	c04
.........................................................aaauucgguucuagagagguuugu...............................	3	0	c04
.........................................................aaauucgguucuagagagguuugA...............................	1	1	c04
.........................................................aaauucgguucuagagagUuuugu...............................	1	1	c04
.........................................................aaauucgguucuagagagguuuUu...............................	1	1	c04
...........................................................auucgguucuagagaggu...................................	3	0	c04
...........................................................auucgguucuagagagguu..................................	6	0	c04
...........................................................auucgguucuagagagguuu.................................	15	0	c04
...........................................................auucgguucuagagagguuuU................................	1	1	c04
............................................................uucgguucuagagagguu..................................	1	0	c04
............................................................uucgguucuagagagguuu.................................	3	0	c04

...............Gcaucuacccuguagauccgaauuugu......................................................................	1	1	c09
................cCucuacccuguagauccgaauuugu......................................................................	2	1	c09
................Gaucuacccuguagauccgaauuugu......................................................................	1	1	c09
................cUucuacccuguagauccgaauuugu......................................................................	1	1	c09
.................aGcuacccuguagauccgaauu.........................................................................	1	1	c09
.................Uucuacccuguagauccgaauuu........................................................................	1	1	c09
.................aAcuacccuguagauccgaauuugu......................................................................	2	1	c09
.................aucuacccuguagauccgaauuugu......................................................................	2	0	c09
.................aGcuacccuguagauccgaauuugu......................................................................	2	1	c09
..................uUuacccuguagauccga............................................................................	1	1	c09
..................Gcuacccuguagauccgaauu.........................................................................	1	1	c09
..................ucuacccuguagauccgaauu.........................................................................	1	0	c09
..................ucuacccuguagauccgaauuug.......................................................................	1	0	c09
..................uUuacccuguagauccgaauuugu......................................................................	3	1	c09
..................ucuacccuguagauccgaauuugu......................................................................	1	0	c09
..................Ccuacccuguagauccgaauuugu......................................................................	1	1	c09
..................Gcuacccuguagauccgaauuuguu.....................................................................	1	1	c09
```

Mature **Star**

caauuuuagugcccuacaucuacccuguagauccgaauuuguuugaagugaggcgacaaauucgguucuagagagguuuguguggugcaugcacugcaacguuucaagucug			
...................Uuacccuguagauccgaauuugu..	2	1	c09
...................cuacccuguagauccgaauuugu..	8	0	c09
...................cuGcccuguagauccgaauuugu..	1	1	c09
....................uacccuguagaAccgaau..	1	1	c09
....................uacccuguagaucAgaau..	6	1	c09
....................uacccuCuagauccgaau..	3	1	c09
....................uaAccuguagauccgaau..	3	1	c09
....................uacccuguagauccgaaG..	14	1	c09
....................uacccugAagauccgaau..	1	1	c09
....................Cacccuguagauccgaau..	1	1	c09
....................uacccuguGgauccgaau..	2	1	c09
....................uacccuguagauGcgaau..	1	1	c09
....................uacccuguagauccgaCu..	1	1	c09
....................uacccGguagauccgaau..	1	1	c09
....................uacccuguagauccgaGu..	4	1	c09
....................uacccuguagCuccgaau..	2	1	c09
....................uacccuUuagauccgaau..	4	1	c09
....................uacccuguagGuccgaau..	2	1	c09
....................Gacccuguagauccgaau..	161	1	c09
....................uCcccuguagauccgaau..	10	1	c09
....................uacccuguagauAcgaau..	5	1	c09
....................uacccuguagaucGgaau..	1	1	c09
....................uacAcuguagauccgaau..	9	1	c09
....................uacccuguagauccgUau..	2	1	c09
....................uacUcuguagauccgaau..	2	1	c09
....................uacccuguagauccgaaC..	15	1	c09
....................uacccuguagauccgaau..	2978	0	c09
....................uaUccuguagauccgaau..	1	1	c09
....................uacccuguagauUcgaau..	1	1	c09
....................uacccuguagaucUgaau..	2	1	c09
....................uacccuguagauccgaUu..	2	1	c09
....................Aacccuguagauccgaau..	18	1	c09
....................uGcccuguagauccgaau..	3	1	c09
....................uacccuguagauccgaaA..	20	1	c09
....................uacccuguagauccgaGuu...	1	1	c09
....................uacccuguGgauccgaauu...	2	1	c09
....................uacAcuguagauccgaauu...	13	1	c09
....................Aacccuguagauccgaauu...	18	1	c09
....................uacccugCagauccgaauu...	1	1	c09
....................uacccuguagauccgaaGu...	8	1	c09
....................uacccGguagauccgaauu...	1	1	c09
....................uacccuguagaCccgaauu...	2	1	c09
....................uacccugGagauccgaauu...	2	1	c09
....................uacccugAagauccgaauu...	1	1	c09
....................uacccuguagauccgaCuu...	3	1	c09
....................uacccuguaAauccgaauu...	1	1	c09
....................uacccuguagauccgaauu...	3504	0	c09
....................uacccCguagauccgaauu...	3	1	c09
....................uaccAuguagauccgaauu...	2	1	c09
....................uaUccuguagauccgaauu...	1	1	c09
....................uacccuguagauccgaauC...	24	1	c09
....................uacUcuguagauccgaauu...	1	1	c09
....................uacccuguaUauccgaauu...	2	1	c09
....................uacccuguagauccgGauu...	1	1	c09
....................uacccuguagauccUaauu...	1	1	c09
....................uacccuCuagauccgaauu...	2	1	c09
....................uacccuguagaucUgaauu...	2	1	c09
....................uCcccuguagauccgaauu...	5	1	c09
....................uacccuguagauccgaauG...	7	1	c09
....................uacGcuguagauccgaauu...	1	1	c09
....................uacccuguagaucGgaauu...	2	1	c09
....................uacccuguagauccAaauu...	1	1	c09
....................uacccuguagaucAgaauu...	10	1	c09
....................uacccuguagCuccgaauu...	3	1	c09
....................uaAccuguagauccgaauu...	1	1	c09
....................uacccuguagauccgaaAu...	1	1	c09
....................uacccuguagauccgaaCu...	3	1	c09
....................Cacccuguagauccgaauu...	1	1	c09
....................Gacccuguagauccgaauu...	157	1	c09
....................uacccuguagauccgaauA...	22	1	c09

```
                    Mature                         Star
caauuuuagugcccuacaucuacccuguagauccgaauuuguuugaagugaggcgacaaauucgguucuagagagguuuguguggugcaugcacugcaacguuucaagucug
....................uaccUuguagauccgaauu.........................................................................   1      1   c09
....................uacccuguagauAcgaauu.........................................................................   2      1   c09
....................uacccuguGgauccgaauuu........................................................................   1      1   c09
....................uaccUuguagauccgaauuu........................................................................   1      1   c09
....................uacccuguagauccgGauuu........................................................................   1      1   c09
....................Gacccuguagauccgaauuu........................................................................   87     1   c09
....................uacccuguagauccgaauCu........................................................................   5      1   c09
....................uacccuguagauccgaauuG........................................................................   8      1   c09
....................uaccAuguagauccgaauuu........................................................................   1      1   c09
....................uacccuguagaucUgaauuu........................................................................   1      1   c09
....................uacccuguagauccgaauGu........................................................................   2      1   c09
....................uacccCguagauccgaauuu........................................................................   2      1   c09
....................uaAccuguagauccgaauuu........................................................................   2      1   c09
....................uaccGuguagauccgaauuu........................................................................   1      1   c09
....................uCcccuguagauccgaauuu........................................................................   6      1   c09
....................uacccugCagauccgaauuu........................................................................   1      1   c09
....................uacccuguagaucAgaauuu........................................................................   5      1   c09
....................uacAcuguagauccgaauuu........................................................................   4      1   c09
....................uacccuguagauccgaauuu........................................................................   1813   0   c09
....................uacGcuguagauccgaauuu........................................................................   1      1   c09
....................uacccuguagauAcgaauuu........................................................................   1      1   c09
....................uacccuAuagauccgaauuu........................................................................   1      1   c09
....................uacccuguagauUcgaauuu........................................................................   2      1   c09
....................uaUccuguagauccgaauuu........................................................................   1      1   c09
....................uacccuguagauccgUauuu........................................................................   1      1   c09
....................uacccuguagCuccgaauuu........................................................................   3      1   c09
....................Cacccuguagauccgaauuu........................................................................   2      1   c09
....................Aacccuguagauccgaauuu........................................................................   1      1   c09
....................uacccuguagauccgaaCuu........................................................................   1      1   c09
....................uacccuCuagauccgaauuu........................................................................   6      1   c09
....................uacccGguagauccgaauuu........................................................................   1      1   c09
....................uUcccuguagauccgaauuu........................................................................   1      1   c09
....................uacccuguagauccAaauuu........................................................................   2      1   c09
....................uacccuguaUauccgaauuu........................................................................   1      1   c09
....................uacccuguagauccgaauuA........................................................................   17     1   c09
....................uacccuguagauccgaauuC........................................................................   30     1   c09
....................uacUcuguagauccgaauuu........................................................................   1      1   c09
....................uacccugCagauccgaauuug.......................................................................   2      1   c09
....................uaccUuguagauccgaauuug.......................................................................   1      1   c09
....................uaAccuguagauccgaauuug.......................................................................   7      1   c09
....................uacAcuguagauccgaauuug.......................................................................   20     1   c09
....................uacccuguGgauccgaauuug.......................................................................   4      1   c09
....................uacccCguagauccgaauuug.......................................................................   3      1   c09
....................uacccuguagauccgaaCuug.......................................................................   4      1   c09
....................uCcccuguagauccgaauuug.......................................................................   8      1   c09
....................uacccuguagauccAaauuug.......................................................................   2      1   c09
....................uacccuUuagauccgaauuug.......................................................................   1      1   c09
....................uacccuguagGuccgaauuug.......................................................................   2      1   c09
....................Cacccuguagauccgaauuug.......................................................................   1      1   c09
....................uacccuguagauccgaGuuug.......................................................................   1      1   c09
....................uacccuguagauccgaauuAg.......................................................................   3      1   c09
....................Aacccuguagauccgaauuug.......................................................................   21     1   c09
....................uacccuguaUauccgaauuug.......................................................................   1      1   c09
....................uacccuguUgauccgaauuug.......................................................................   1      1   c09
....................uacccuguagauccgaaAuug.......................................................................   2      1   c09
....................uacccuCuagauccgaauuug.......................................................................   1      1   c09
....................uacccuguagauAcgaauuug.......................................................................   8      1   c09
....................uGcccuguagauccgaauuug.......................................................................   1      1   c09
....................uacccuguagauccCaauuug.......................................................................   2      1   c09
....................uacccuguagCuccgaauuug.......................................................................   7      1   c09
....................uacccuguagaucAgaauuug.......................................................................   8      1   c09
....................uacccuguagauccgaauCug.......................................................................   1      1   c09
....................uacccuguagauccgaauuuC.......................................................................   12     1   c09
....................uacccuguagauccgaauuGg.......................................................................   4      1   c09
....................uacccuguagauccgaauGug.......................................................................   1      1   c09
....................uacccuguagauccgaauAug.......................................................................   2      1   c09
....................uacccuguagauccgaauuCg.......................................................................   1      1   c09
....................uacccuguagauccgaauuuU.......................................................................   11     1   c09
....................uacccuguagauccgaauuug.......................................................................   4083   0   c09
....................Gacccuguagauccgaauuug.......................................................................   217    1   c09
```

Mature / Star			
caauuuuagugcccuacaucuacccuguagauccgaauuuguuugaagugaggcgacaaauucgguucuagagagguuuguguggugcaugcacugcaacguuucaagucug			
....................uacccuguagauccgaauuuA...	10	1	c09
....................uacccugGagauccgaauuug...	1	1	c09
....................uacccuguagaCccgaauuug...	1	1	c09
....................uaccAuguagauccgaauuug...	1	1	c09
....................uacccuguagauccUaauuug...	1	1	c09
....................uaUccuguagauccgaauuug...	1	1	c09
....................uacccugAagauccgaauuug...	1	1	c09
....................Gacccuguagauccgaauuugu..	2801	1	c09
....................uacccuguagauccgaauuugC..	26	1	c09
....................uacccuguagaAccgaauuugu..	6	1	c09
....................uaccGuguagauccgaauuugu..	4	1	c09
....................uacccuguagauccgaGuuugu..	5	1	c09
....................uUccuguagauccgaauuugu..	7	1	c09
....................uacccuguagauccgaaAuugu..	15	1	c09
....................uacccuguagauGcgaauuugu..	8	1	c09
....................uacccuguagauccgaauuugu..	52171	0	c09
....................uacccuguagauccgaauuuUu..	20	1	c09
....................uacccuguagauUcgaauuugu..	16	1	c09
....................uCccuguagauccgaauuugu..	102	1	c09
....................uacccuAuagauccgaauuugu..	14	1	c09
....................uacccuguaCauccgaauuugu..	11	1	c09
....................uacccuCuagauccgaauuugu..	36	1	c09
....................uacccuguagaucGgaauuugu..	7	1	c09
....................uGcccuguagauccgaauuugu..	13	1	c09
....................uacccuguCgauccgaauuugu..	4	1	c09
....................uacccuguagUuccgaauuugu..	7	1	c09
....................uacccuguagaGccgaauuugu..	10	1	c09
....................uacccuguagauccgaCuuugu..	2	1	c09
....................uacccugCagauccgaauuugu..	7	1	c09
....................uacccuguagauccgaauuugA..	42	1	c09
....................uacccugAagauccgaauuugu..	15	1	c09
....................uacccuguagauccgaaCuugu..	14	1	c09
....................uacccuguagauccgaauCugu..	14	1	c09
....................uaccAuguagauccgaauuugu..	36	1	c09
....................uacGcuguagauccgaauuugu..	21	1	c09
....................uacccugGagauccgaauuugu..	4	1	c09
....................uacUcuguagauccgaauuugu..	15	1	c09
....................uacccAguagauccgaauuugu..	15	1	c09
....................uacccuguagauccgaauuAgu..	17	1	c09
....................uacccGguagauccgaauuugu..	11	1	c09
....................uaGccuguagauccgaauuugu..	4	1	c09
....................uacccuguagauccgGauuugu..	3	1	c09
....................uaccUuguagauccgaauuugu..	12	1	c09
....................uacccuguagauccgaauuugG..	26	1	c09
....................uacccuguagaCccgaauuugu..	6	1	c09
....................uaAccuguagauccgaauuugu..	29	1	c09
....................Cacccuguagauccgaauuugu..	25	1	c09
....................Aacccuguagauccgaauuugu..	235	1	c09
....................uaUccuguagauccgaauuugu..	12	1	c09
....................uacccuguagauccCaauuugu..	12	1	c09
....................uacccuguagauccgaauuCgu..	15	1	c09
....................uacccuguagauccgaauGugu..	2	1	c09
....................uacccuguagauAcgaauuugu..	54	1	c09
....................uacccuguaAauccgaauuugu..	6	1	c09
....................uacccuguagauccgaauuuAu..	17	1	c09
....................uacccuguagaucUgaauuugu..	16	1	c09
....................uacccuguagaucAgaauuugu..	82	1	c09
....................uacccuguagauccgaauuuCu..	47	1	c09
....................uacAcuguagauccgaauuugu..	180	1	c09
....................uacccuguagGuccgaauuugu..	5	1	c09
....................uacccCguagauccgaauuugu..	37	1	c09
....................uacccuguagauccgaauAugu..	15	1	c09
....................uacccuguagCuccgaauuugu..	100	1	c09
....................uacccuguagauccgaaGuugu..	9	1	c09
....................uacccuguagauccgaUuuugu..	18	1	c09
....................uacccuguGgauccgaauuugu..	18	1	c09
....................uacccuguUgauccgaauuugu..	12	1	c09
....................uacccuguagauccUaauuugu..	26	1	c09
....................uacccuguagauccgaauuGgu..	3	1	c09
....................uacccuguaUauccgaauuugu..	19	1	c09

```
                     Mature                          Star
caauuuuagugcccuacaucuacccuguagauccgaauuuguuugaagugaggcgacaaauucgguucuagagagguuuguguggugcaugcacugcaacguuucaagucug
....................uacccuguagauccAaauuugu......................................................................   12     1   c09
....................uacccuguagauccgUauuugu......................................................................   8      1   c09
....................uacccuUuagauccgaauuugu......................................................................   31     1   c09
....................uacccuguGgauccgaauuuguu.....................................................................   1      1   c09
....................uacccuguagauccgaauuuguu.....................................................................   777    0   c09
....................Cacccuguagauccgaauuuguu.....................................................................   4      1   c09
....................uacccuguagauccgaauuuguG.....................................................................   94     1   c09
....................uacccuguagauccgaauuuguC.....................................................................   134    1   c09
....................uGcccuguagauccgaauuuguu.....................................................................   1      1   c09
....................uacccuguagCuccgaauuuguu.....................................................................   3      1   c09
....................uacUcuguagauccgaauuuguu.....................................................................   2      1   c09
....................uaccAuguagauccgaauuuguu.....................................................................   1      1   c09
....................uacccuguagauccCaauuuguu.....................................................................   1      1   c09
....................uacccuguagauAcgaauuuguu.....................................................................   2      1   c09
....................Aacccuguagauccgaauuuguu.....................................................................   3      1   c09
....................uacccuguagauccgaauuugGu.....................................................................   2      1   c09
....................uacccuguaCauccgaauuuguu.....................................................................   1      1   c09
....................uacccuguagaucAgaauuuguu.....................................................................   2      1   c09
....................uacccuguagauccgaauuuCuu.....................................................................   1      1   c09
....................uCcccuguagauccgaauuuguu.....................................................................   4      1   c09
....................uacccuguagauccgaauuuguA.....................................................................   403    1   c09
....................uacAcuguagauccgaauuuguu.....................................................................   6      1   c09
....................uaccUuguagauccgaauuuguu.....................................................................   1      1   c09
....................uacccuguagauccgaUuuuguu.....................................................................   1      1   c09
....................uacccuguagauccgaauuugCu.....................................................................   1      1   c09
....................Gacccuguagauccgaauuuguu.....................................................................   40     1   c09
....................uacccuguagauccgaauuuguAu....................................................................   9      1   c09
....................uacccuguagauccgaauuuguuu....................................................................   16     0   c09
....................Gacccuguagauccgaauuuguuu....................................................................   1      1   c09
....................uacccuguagauccgaauuuguCu....................................................................   11     1   c09
....................uacccuguagCuccgaauuuguuu....................................................................   1      1   c09
....................uacccuguagauccgaauuuguuA....................................................................   14     1   c09
....................uacccuguagauccgaauuuguuC....................................................................   10     1   c09
....................uacccuguagauccgaauuuguuG....................................................................   4      1   c09
....................uacccuguagauccgaauuuCuuu....................................................................   1      1   c09
....................uacccuguagauccgaauuuguGu....................................................................   5      1   c09
....................uacccuguagauccgaauuuguuug...................................................................   4      0   c09
....................uacccuguagGuccgaauuuguuug...................................................................   1      1   c09
....................uacccuguagauccgaauuuguuuU...................................................................   1      1   c09
....................uacccuguagauccgaauuuguuuA...................................................................   1      1   c09
....................uacccuguagauccgaauuuguCug...................................................................   1      1   c09
....................uacccuguagauccgaauuuguuugG..................................................................   1      1   c09
....................uacccuguagauccgaauuuguuugU..................................................................   7      1   c09
....................uacccuguagauccgaauuuguuuga..................................................................   3      0   c09
....................uacccuguagauccgaauuuguuugaag................................................................   1      0   c09
....................uacccuguagauccgaauuuguuugaagu...............................................................   2      0   c09
....................uacccuguagauccgaauuuguuugaagug..............................................................   1      0   c09
.....................Ccccuguagauccgaauu.........................................................................   378    1   c09
.....................acccuguagauccgaGuu.........................................................................   2      1   c09
.....................acccAguagauccgaauu.........................................................................   3      1   c09
.....................acccuguagUuccgaauu.........................................................................   1      1   c09
.....................acccuguagauccUaauu.........................................................................   1      1   c09
.....................acUcuguagauccgaauu.........................................................................   1      1   c09
.....................aUccuguagauccgaauu.........................................................................   1      1   c09
.....................acccuguagaucAgaauu.........................................................................   7      1   c09
.....................acccuguagauccgaaGu.........................................................................   3      1   c09
.....................acccuUuagauccgaauu.........................................................................   3      1   c09
.....................acccuguagauGcgaauu.........................................................................   1      1   c09
.....................acccuguagauccgaauG.........................................................................   10     1   c09
.....................acccuguagauccgaaCu.........................................................................   3      1   c09
.....................acccuguagauccAaauu.........................................................................   1      1   c09
.....................acccuguagCuccgaauu.........................................................................   2      1   c09
.....................acccuguagauccgaauu.........................................................................   1912   0   c09
.....................acccuguagauccgaUuu.........................................................................   1      1   c09
.....................acccGguagauccgaauu.........................................................................   2      1   c09
.....................acAcuguagauccgaauu.........................................................................   7      1   c09
.....................acccuguagauccgUauu.........................................................................   1      1   c09
.....................accAuguagauccgaauu.........................................................................   3      1   c09
.....................acccuguagauccgaauC.........................................................................   7      1   c09
.....................acccuguagauccgaCuu.........................................................................   1      1   c09
```

```
                     Mature                Star
caauuuuagugcccuacaucuacccuguagauccgaauuuguuugaagugaggcgacaaauucgguucuagagagguuuguguggugcaugcacugcaacguuucaagucug
.....................Gcccuguagauccgaauu.........................................................................  3     1  c09
.....................acccuguagauccgaauA.........................................................................  9     1  c09
.....................acccuCuagauccgaauu.........................................................................  2     1  c09
.....................acccGguagauccgaauuu........................................................................  1     1  c09
.....................acccuCuagauccgaauuu........................................................................  2     1  c09
.....................acccuguagauAcgaauuu........................................................................  1     1  c09
.....................acccuguagauccgaCuuu........................................................................  1     1  c09
.....................acccuguagauccgaauGu........................................................................  5     1  c09
.....................acAcuguagauccgaauuu........................................................................  4     1  c09
.....................acccuguagauccgaauAu........................................................................  1     1  c09
.....................acccCguagauccgaauuu........................................................................  2     1  c09
.....................acccuguagauccgaauuA........................................................................  7     1  c09
.....................acccuguagauccgaGuuu........................................................................  1     1  c09
.....................accGuguagauccgaauuu........................................................................  1     1  c09
.....................accUuguagauccgaauuu........................................................................  1     1  c09
.....................acccuguagCuccgaauuu........................................................................  4     1  c09
.....................acccuguagaAccgaauuu........................................................................  1     1  c09
.....................acccuguagaucAgaauuu........................................................................  5     1  c09
.....................Ccccuguagauccgaauuu........................................................................  278   1  c09
.....................aAccuguagauccgaauuu........................................................................  1     1  c09
.....................acccuguagauccgaaAuu........................................................................  1     1  c09
.....................Gcccuguagauccgaauuu........................................................................  1     1  c09
.....................acccuguagauccgaaCuu........................................................................  1     1  c09
.....................acUcuguagauccgaauuu........................................................................  1     1  c09
.....................acccuguagauUcgaauuu........................................................................  1     1  c09
.....................acccuguagauccgaauuu........................................................................  1358  0  c09
.....................acccuguagaCccgaauuu........................................................................  1     1  c09
.....................acccuguaCauccgaauuu........................................................................  1     1  c09
.....................acccuguaUauccgaauuu........................................................................  2     1  c09
.....................acccuguagauccgaauCu........................................................................  1     1  c09
.....................accAuguagauccgaauuu........................................................................  3     1  c09
.....................acccuguagauccgaauuG........................................................................  8     1  c09
.....................acccuguagauccgaauuC........................................................................  11    1  c09
.....................Ccccuguagauccgaauuug.......................................................................  334   1  c09
.....................aAccuguagauccgaauuug.......................................................................  1     1  c09
.....................acccuguagauccgaauuug.......................................................................  1401  0  c09
.....................acccuguagCuccgaauuug.......................................................................  2     1  c09
.....................acccugAagauccgaauuug.......................................................................  1     1  c09
.....................acccuguagauccgaauGug.......................................................................  1     1  c09
.....................accGuguagauccgaauuug.......................................................................  1     1  c09
.....................acccuguagaucAgaauuug.......................................................................  3     1  c09
.....................acccuguagauccgaauCug.......................................................................  1     1  c09
.....................acccuguagauccCaauuug.......................................................................  1     1  c09
.....................acccuguagauccgaauuuA.......................................................................  4     1  c09
.....................acccAguagauccgaauuug.......................................................................  2     1  c09
.....................acccCguagauccgaauuug.......................................................................  4     1  c09
.....................accAuguagauccgaauuug.......................................................................  2     1  c09
.....................acccuguagauccgaauuuU.......................................................................  7     1  c09
.....................acccuguagauccgaauuAg.......................................................................  1     1  c09
.....................acccuguagaucUgaauuug.......................................................................  1     1  c09
.....................acccuguagauccgaGuuug.......................................................................  1     1  c09
.....................acccuCuagauccgaauuug.......................................................................  1     1  c09
.....................acccuguagauccgaauuuC.......................................................................  10    1  c09
.....................acccuguagauAcgaauuug.......................................................................  2     1  c09
.....................acccuguGgauccgaauuug.......................................................................  1     1  c09
.....................acAcuguagauccgaauuug.......................................................................  2     1  c09
.....................Gcccuguagauccgaauuugu......................................................................  17    1  c09
.....................acccuguaAauccgaauuugu......................................................................  1     1  c09
.....................accUuguagauccgaauuugu......................................................................  2     1  c09
.....................acccuguagauccgaaAuugu......................................................................  5     1  c09
.....................acccuguagauccgaauuGgu......................................................................  1     1  c09
.....................acccuguagauccgaaCuugu......................................................................  5     1  c09
.....................aUccuguagauccgaauuugu......................................................................  5     1  c09
.....................acccuguUgauccgaauuugu......................................................................  5     1  c09
.....................acccuguagauccgaauuCgu......................................................................  2     1  c09
.....................acccuguagauccAaauuugu......................................................................  2     1  c09
.....................acccuguagauccgaaGuugu......................................................................  4     1  c09
.....................acccugCagauccgaauuugu......................................................................  1     1  c09
.....................acccuguagauccgaauuuUu......................................................................  3     1  c09
.....................acccugAagauccgaauuugu......................................................................  1     1  c09
```

```
                     Mature                           Star
caauuuuagugcccuacaucuacccuguagauccgaauuuguuugaagugaggcgacaaauucgguucuagagagguuuguguggugcaugcacugcaacguuucaagucug
.....................aGccuguagauccgaauuugu.......................................................................	2	1	c09
.....................acccuguagauGcgaauuugu.......................................................................	1	1	c09
.....................Ucccuguagauccgaauuugu.......................................................................	1	1	c09
.....................acAcuguagauccgaauuugu.......................................................................	21	1	c09
.....................acccuguaUauccgaauuugu.......................................................................	1	1	c09
.....................acccuguagauccgaauCugu.......................................................................	1	1	c09
.....................acccuguagauccgaauuuCu.......................................................................	15	1	c09
.....................Ccccuguagauccgaauuugu.......................................................................	1633	1	c09
.....................acccuguGgauccgaauuugu.......................................................................	3	1	c09
.....................acccuguaCauccgaauuugu.......................................................................	1	1	c09
.....................acUcuguagauccgaauuugu.......................................................................	2	1	c09
.....................acccuguagauAcgaauuugu.......................................................................	7	1	c09
.....................acccGguagauccgaauuugu.......................................................................	2	1	c09
.....................acccuCuagauccgaauuugu.......................................................................	12	1	c09
.....................acccAguagauccgaauuugu.......................................................................	5	1	c09
.....................acccuguagauccgaauAugu.......................................................................	2	1	c09
.....................acccuguagauUcgaauuugu.......................................................................	4	1	c09
.....................acccuAuagauccgaauuugu.......................................................................	3	1	c09
.....................acccuguagaucAgaauuugu.......................................................................	21	1	c09
.....................acccuUuagauccgaauuugu.......................................................................	7	1	c09
.....................acccuguagauccgUauuugu.......................................................................	3	1	c09
.....................acccuguagauccUaauuugu.......................................................................	3	1	c09
.....................acccuguagaucGgaauuugu.......................................................................	2	1	c09
.....................acccuguagauccgaauGugu.......................................................................	1	1	c09
.....................acccuguagaAccgaauuugu.......................................................................	2	1	c09
.....................accAuguagauccgaauuugu.......................................................................	10	1	c09
.....................acccuguagaGccgaauuugu.......................................................................	1	1	c09
.....................acccuguagauccgaauuugC.......................................................................	7	1	c09
.....................acccCguagauccgaauuugu.......................................................................	2	1	c09
.....................acccuguagauccgaauuuAu.......................................................................	5	1	c09
.....................aAccuguagauccgaauuugu.......................................................................	10	1	c09
.....................acccuguagCuccgaauuugu.......................................................................	12	1	c09
.....................acccuguagauccgaauuugu.......................................................................	7649	0	c09
.....................acccuguagaucUgaauuugu.......................................................................	1	1	c09
.....................acccuguagauccgaauuugA.......................................................................	10	1	c09
.....................acccuguagauccCaauuugu.......................................................................	2	1	c09
.....................acccuguagauccgaauuugG.......................................................................	12	1	c09
.....................acccuguagauccgaauuuguu.....................................................................	24380	0	c09
.....................acccuguagaAccgaauuuguu.....................................................................	7	1	c09
.....................acUcuguagauccgaauuuguu.....................................................................	11	1	c09
.....................aAccuguagauccgaauuuguu.....................................................................	17	1	c09
.....................acccuguagauccgGauuuguu.....................................................................	2	1	c09
.....................acccuguUgauccgaauuuguu.....................................................................	4	1	c09
.....................acccuguagauccgaGuuuguu.....................................................................	3	1	c09
.....................acGcuguagauccgaauuuguu.....................................................................	2	1	c09
.....................acccuguagauccgaauuAguu.....................................................................	12	1	c09
.....................acccuguagauUcgaauuuguu.....................................................................	6	1	c09
.....................acccuguagGuccgaauuuguu.....................................................................	2	1	c09
.....................acccuguagauccgaauuuUuu.....................................................................	11	1	c09
.....................acccuguagauAcgaauuuguu.....................................................................	36	1	c09
.....................Ccccuguagauccgaauuuguu.....................................................................	5153	1	c09
.....................acccuAuagauccgaauuuguu.....................................................................	6	1	c09
.....................acccuguagauccgaauuugAu.....................................................................	17	1	c09
.....................acccuguagauccgaauuugCu.....................................................................	7	1	c09
.....................acccGguagauccgaauuuguu.....................................................................	5	1	c09
.....................acccugGagauccgaauuuguu.....................................................................	2	1	c09
.....................acccAguagauccgaauuuguu.....................................................................	13	1	c09
.....................aGccuguagauccgaauuuguu.....................................................................	2	1	c09
.....................acccuguGgauccgaauuuguu.....................................................................	12	1	c09
.....................acccuguagaucAgaauuuguu.....................................................................	69	1	c09
.....................acccuguaAauccgaauuuguu.....................................................................	2	1	c09
.....................acccuguagauccgaaGuuguu.....................................................................	1	1	c09
.....................Ucccuguagauccgaauuuguu.....................................................................	4	1	c09
.....................acccuguagauccgUauuuguu.....................................................................	6	1	c09
.....................acccuguagauccgaauuuguG.....................................................................	13	1	c09
.....................acccuguagauccgaaAuuguu.....................................................................	13	1	c09
.....................acccuguagUuccgaauuuguu.....................................................................	3	1	c09
.....................acccuguagauGcgaauuuguu.....................................................................	1	1	c09
.....................accAuguagauccgaauuuguu.....................................................................	23	1	c09
.....................acccuguagaCccgaauuuguu.....................................................................	7	1	c09
```

Mature Star

Sequence	Count	Mismatch	Library
caauuuuagugcccuacaucuacccuguagauccgaauuuguuugaagugaggcgacaaauucgguucuagagagguuugugguggugcaugcacugcaacguuucaagucug			
.....................acccuguagaucGgaauuuguu...	2	1	c09
.....................acccuguagauccgaauuCguu...	9	1	c09
.....................acccugCagauccgaauuuguu...	6	1	c09
.....................acccuguagauccgaauCuguu...	2	1	c09
.....................acccuguagauccgaauuuguC...	24	1	c09
.....................acccuguaUauccgaauuuguu...	2	1	c09
.....................acccuCuagauccgaauuuguu...	28	1	c09
.....................acccuguaCauccgaauuuguu...	2	1	c09
.....................acccuguagauccgaauuugGu...	15	1	c09
.....................acccugAagauccgaauuuguu...	11	1	c09
.....................acccuguagauccgaaCuuguu...	7	1	c09
.....................acccCguagauccgaauuuguu...	5	1	c09
.....................acccuguagauccCaauuuguu...	7	1	c09
.....................acccuguagauccgaauuuguA...	64	1	c09
.....................acccuguagauccgaUuuuguu...	7	1	c09
.....................acccuUuagauccgaauuuguu...	11	1	c09
.....................acccuguagauccgaauAuguu...	9	1	c09
.....................Gcccuguagauccgaauuuguu...	28	1	c09
.....................acccuguagCuccgaauuuguu...	39	1	c09
.....................aUccuguagauccgaauuuguu...	23	1	c09
.....................acccuguagauccAaauuuguu...	5	1	c09
.....................accGuguagauccgaauuuguu...	2	1	c09
.....................acAcuguagauccgaauuuguu...	71	1	c09
.....................accUuguagauccgaauuuguu...	15	1	c09
.....................acccuguagauccgaauuuAuu...	7	1	c09
.....................acccuguagauccgaauuGguu...	3	1	c09
.....................acccuguagaucUgaauuuguu...	19	1	c09
.....................acccuguagauccgaauuuCuu...	24	1	c09
.....................acccuguagauccUaauuuguu...	8	1	c09
.....................acccuguCgauccgaauuuguu...	3	1	c09
.....................acccuguagaGccgaauuuguu...	6	1	c09
.....................acccAguagauccgaauuuguuu..	1	1	c09
.....................acccuguagCuccgaauuuguuu..	3	1	c09
.....................acccuguagaucAgaauuuguuu..	3	1	c09
.....................acccuguCgauccgaauuuguuu..	1	1	c09
.....................Gcccuguagauccgaauuuguuu..	1	1	c09
.....................aAccuguagauccgaauuuguuu..	1	1	c09
.....................acccuguagauccgaauuuguuC..	70	1	c09
.....................acccuguagauccgaauuuguuG..	33	1	c09
.....................acccuguagauccgaauuugGuu..	2	1	c09
.....................acccuguagauccgaauuuguuu..	466	0	c09
.....................Ccccuguagauccgaauuuguuu..	86	1	c09
.....................acccuguagauccgaauuuguuA..	324	1	c09
.....................acAcuguagauccgaauuuguuu..	2	1	c09
.....................acUcuguagauccgaauuuguuu..	1	1	c09
.....................acccuguagauccgaauuuguGu..	1	1	c09
.....................acccuguagauccgaauuuguuuC...	6	1	c09
.....................acccuguagauccgaauuuguuGg...	6	1	c09
.....................acccuguagauccgaauuuguuug...	2	0	c09
.....................acccuguagauccgaauuuguCug...	1	1	c09
.....................Ccccuguagauccgaauuuguuug...	1	1	c09
.....................acccuguagauccgaauuuguuCg...	1	1	c09
.....................acccuguagauccgaauuuguuuA...	8	1	c09
.....................acccuguagauccgaauuuguuuU...	10	1	c09
.....................acccuguagauccgaauuuguuugC..	1	1	c09
.....................acccuguagauccgaauuuguuugG..	3	1	c09
.....................acccuguagauccgaauuuguuuga..	1	0	c09
.....................acccuguagauccgaauuuguuugU..	1	1	c09
.....................acccuguagauccgaauuuguuugaa...	2	0	c09
.....................acccuguagauccgaauuuguuuAaa...	1	1	c09
.....................acccuguagauccgaauuuguuugaag..	2	0	c09
.....................acccuguagauccgaauuuguuugaagG...	1	1	c09
.....................acccuguagauccgaauuuguuugCagu...	1	1	c09
.....................acccuguagauccgaauuuguuugaagC...	1	1	c09
.....................acccuguagauccCaauuuguuugaagugag..	1	1	c09
.....................acccuguagauccgaauuuguuugaagugaggcga..	3	0	c09
.....................acccuguagauccgaauuuguuugaagugaggcgac...	1	0	c09
......................cccuguagauccgaauuu..	15	0	c09
......................cccuguagauccgaauuug...	22	0	c09
......................cccuguagauccgaGuuugu..	1	1	c09

Mature **Star**

```
caauuuuagugcccuacaucuacccuguagauccgaauuuguuugaagugaggcgacaaauucgguucuagagagguuugugguggugcaugcacugcaacguuucaagucug
......................Gccuguagauccgaauuugu......................................................................   5     1   c09
......................cccuguagauccgaauuugu......................................................................   172   0   c09
......................cccuguagauccgaauuugG......................................................................   1     1   c09
......................Uccuguagauccgaauuugu......................................................................   2     1   c09
......................Uccuguagauccgaauuuguu.....................................................................   1     1   c09
......................cccuguagaucAgaauuuguu.....................................................................   1     1   c09
......................cccuguaUauccgaauuuguu.....................................................................   1     1   c09
......................cccuguaCauccgaauuuguu.....................................................................   1     1   c09
......................cccuguagauccgaauuuguu.....................................................................   97    0   c09
......................cccuguagCuccgaauuuguu.....................................................................   2     1   c09
......................Gccuguagauccgaauuuguu.....................................................................   1     1   c09
.......................ccuguagauccgaauuug.......................................................................   18    0   c09
.......................ccCguagauccgaauuugu......................................................................   3     1   c09
.......................cUuguagauccgaauuugu......................................................................   1     1   c09
.......................ccuguagCuccgaauuugu......................................................................   1     1   c09
.......................ccuguagauccgaauuugu......................................................................   202   0   c09
.......................ccuguagauccgaauuuguu.....................................................................   87    0   c09
.......................ccuguagauccgaauuuguA.....................................................................   1     1   c09
.......................ccuguagauccgaauuuguuC....................................................................   1     1   c09
.......................ccuguagauccgaauuuguuu....................................................................   3     0   c09
.......................ccuguagauccgaauuuguuA....................................................................   1     1   c09
........................cuguagauccgaGuuugu......................................................................   1     1   c09
........................cuguagauccgaauuugG......................................................................   1     1   c09
........................cuguagaucAgaauuugu......................................................................   1     1   c09
........................cuguagauccgaauuugu......................................................................   190   0   c09
........................cuguagauccUaauuugu......................................................................   1     1   c09
........................cuguagCuccgaauuugu......................................................................   2     1   c09
........................Uuguagauccgaauuugu......................................................................   4     1   c09
........................Guguagauccgaauuugu......................................................................   1     1   c09
........................Uuguagauccgaauuuguu.....................................................................   1     1   c09
........................Auguagauccgaauuuguu.....................................................................   2     1   c09
........................cuguagauccgaauuGguu.....................................................................   1     1   c09
........................cuguagauAcgaauuuguu.....................................................................   1     1   c09
........................cuguagaucAgaauuuguu.....................................................................   1     1   c09
........................cuguagauccgaauuuguA.....................................................................   3     1   c09
........................cuguagauccgaauuuguu.....................................................................   106   0   c09
........................cuguagauccgaauuuAuu.....................................................................   1     1   c09
........................cuguagauccgaauuuguuA....................................................................   3     1   c09
........................cuguagauccgaauuuguuu....................................................................   6     0   c09
.........................uguagauccgaauuuguu.....................................................................   133   0   c09
.........................uguagauccgaauuugAu.....................................................................   1     1   c09
.........................Gguagauccgaauuuguu.....................................................................   15    1   c09
.........................ugGagauccgaauuuguu.....................................................................   1     1   c09
.........................Aguagauccgaauuuguu.....................................................................   1     1   c09
.........................uguagauccgaauuuguC.....................................................................   1     1   c09
.........................uguagauccgaauuuguuu....................................................................   4     0   c09
.........................uguagauccgaauuuguuA....................................................................   1     1   c09
.........................uguagauccgaauuuguuG....................................................................   2     1   c09
.........................uguagauccgaauuuguuC....................................................................   1     1   c09
.........................uguagauccgaauuuguuug...................................................................   1     0   c09
..........................guagCuccgaauuuguu.....................................................................   1     1   c09
..........................guagauccgaauuuguuG....................................................................   1     1   c09
..........................guagauccgaauuuguuA....................................................................   1     1   c09
.....................................................cUacaaauucgguucuagagagg....................................   1     1   c09
.....................................................Agacaaauucgguucuagagagg....................................   1     1   c09
.....................................................Agacaaauucgguucuagagaggu...................................   1     1   c09
.....................................................cUacaaauucgguucuagagagguuu.................................   1     1   c09
......................................................gCcaaauucgguucuagagagg....................................   1     1   c09
......................................................gCcaaauucgguucuagagagguu..................................   1     1   c09
......................................................gCcaaauucgguucuagagagguuu.................................   1     1   c09
......................................................gacaaauucgguucuagagagguuu.................................   1     0   c09
.......................................................acaaauucgguucuagagagg....................................   2     0   c09
.......................................................Ucaaauucgguucuagagaggu...................................   1     1   c09
.......................................................Gcaaauucgguucuagagaggu...................................   1     1   c09
.......................................................Ucaaauucgguucuagagagguuu.................................   2     1   c09
.......................................................acaaauucgguucuagagagguuu.................................   1     0   c09
.......................................................aAaaauucgguucuagagagguuu.................................   1     1   c09
.......................................................Gcaaauucgguucuagagagguuu.................................   1     1   c09
.......................................................Ccaaauucgguucuagagagguuu.................................   6     1   c09
.......................................................Ccaaauucgguucuagagagguuug................................   1     1   c09
```

```
                     Mature                         Star
caauuuuagugcccuacaucuacccuguagauccgaauuuguuugaagugaggcgacaaauucgguucuagagagguuuguguggugcaugcacugcaacguuucaagucug
........................................................caaauucggAucuagaga......................................    3     1    c09
........................................................caaaCucgguucuagaga......................................    1     1    c09
........................................................caaauucgguucuagaAa......................................    1     1    c09
........................................................caaaAucgguucuagaga......................................    1     1    c09
........................................................caaauucgguuAuagaga......................................    1     1    c09
........................................................caaauucgguucuagaga......................................    486   0    c09
........................................................caaauuAgguucuagaga......................................    1     1    c09
........................................................caaauucgguucuagagC......................................    1     1    c09
........................................................caaGuucgguucuagaga......................................    1     1    c09
........................................................caaauuAgguucuagagag.....................................    2     1    c09
........................................................caaauucgguucuaCagag.....................................    2     1    c09
........................................................caaauucgCuucuagagag.....................................    1     1    c09
........................................................caaauucgguucuagagaA.....................................    3     1    c09
........................................................caaauuUgguucuagagag.....................................    1     1    c09
........................................................caaauucgguucuagaCag.....................................    1     1    c09
........................................................caaaCucgguucuagagag.....................................    1     1    c09
........................................................caaauucgguucuagagag.....................................    893   0    c09
........................................................caaauucgguucCagagag.....................................    2     1    c09
........................................................caaauucgguAcuagagag.....................................    2     1    c09
........................................................caaauucgguucuagagaU.....................................    6     1    c09
........................................................caaauucgguucuaUagag.....................................    1     1    c09
........................................................caaauucgguucuagaUag.....................................    1     1    c09
........................................................caaCuucgguucuagagag.....................................    1     1    c09
........................................................caaauucggGucuagagag.....................................    2     1    c09
........................................................caaauucggCucuagagag.....................................    1     1    c09
........................................................caaGuucgguucuagagag.....................................    1     1    c09
........................................................caaauucgguucuagUgag.....................................    1     1    c09
........................................................caaauucUguucuagagag.....................................    1     1    c09
........................................................caaauucgguucuGgagag.....................................    1     1    c09
........................................................caaauucgguucuagagaC.....................................    3     1    c09
........................................................caaauucCguucuagagagg....................................    6     1    c09
........................................................caaauucgguuGuagagagg....................................    1     1    c09
........................................................caaauucgguuAuagagagg....................................    9     1    c09
........................................................caaauucUguucuagagagg....................................    2     1    c09
........................................................caaauucgAuucuagagagg....................................    1     1    c09
........................................................caaauucgguucuagGgagg....................................    1     1    c09
........................................................caaauucgCuucuagagagg....................................    5     1    c09
........................................................caaauucgguucuagCgagg....................................    1     1    c09
........................................................caaauucggGucuagagagg....................................    7     1    c09
........................................................caaauucgguucuagagaCg....................................    6     1    c09
........................................................caaauucgguucuCgagagg....................................    1     1    c09
........................................................caaauucgguucuagaUagg....................................    5     1    c09
........................................................caaUuucgguucuagagagg....................................    1     1    c09
........................................................caaauucgguucCagagagg....................................    4     1    c09
........................................................caaauucgguuUuagagagg....................................    1     1    c09
........................................................caaauucgguucuagagagA....................................    14    1    c09
........................................................caaaCucgguucuagagagg....................................    1     1    c09
........................................................caaauucgguucuagagagC....................................    9     1    c09
........................................................caaauucgguucuagagCgg....................................    2     1    c09
........................................................caaauucgguucuGgagagg....................................    1     1    c09
........................................................caaauucgguAcuagagagg....................................    5     1    c09
........................................................caaauucgguucuagagGgg....................................    2     1    c09
........................................................caaauucgguucuagagagg....................................    5579  0    c09
........................................................caGauucgguucuagagagg....................................    1     1    c09
........................................................caaauuUgguucuagagagg....................................    3     1    c09
........................................................caaauucggAucuagagagg....................................    5     1    c09
........................................................caaauucgguucuaCagagg....................................    4     1    c09
........................................................caaauucgguucuagagaAg....................................    3     1    c09
........................................................caaauucgUuucuagagagg....................................    1     1    c09
........................................................caaauucgguucuaUagagg....................................    4     1    c09
........................................................caaaGucgguucuagagagg....................................    1     1    c09
........................................................caaauAcgguucuagagagg....................................    3     1    c09
........................................................caaaAucgguucuagagagg....................................    14    1    c09
........................................................caaauucgguucuUgagagg....................................    2     1    c09
........................................................cUaauucgguucuagagagg....................................    3     1    c09
........................................................Aaaauucgguucuagagagg....................................    4     1    c09
........................................................caaauucgguucAagagagg....................................    3     1    c09
........................................................caaauuAgguucuagagagg....................................    10    1    c09
........................................................Gaaauucgguucuagagagg....................................    1     1    c09
........................................................caaauucgguucuagagaUg....................................    3     1    c09
```

```
                    Mature                           Star
caauuuuagugcccuacaucuacccuguagauccgaauuuguuugaagugaggcgacaaauucgguucuagagagguuuguguggugcaugcacugcaacguuucaagucug
........................................................caaGuucgguucuagagagg....................................  1     1  c09
........................................................caaauucgguucGagagagg....................................  1     1  c09
........................................................caaauucgguucuagaCagg....................................  3     1  c09
........................................................caaauucgguucuagaAagg....................................  2     1  c09
........................................................caaauucggCucuagagagg....................................  2     1  c09
........................................................caaauucgguucuagagagU....................................  14    1  c09
........................................................caaauucgguucuagagaggG...................................  9     1  c09
........................................................caaauucgguucuagagUggu...................................  1     1  c09
........................................................caaauAcgguucuagagaggu...................................  3     1  c09
........................................................caGauucgguucuagagaggu...................................  1     1  c09
........................................................caaauucgguGcuagagaggu...................................  1     1  c09
........................................................caaauucgCuucuagagaggu...................................  13    1  c09
........................................................caUauucgguucuagagaggu...................................  1     1  c09
........................................................caaauucggCucuagagaggu...................................  3     1  c09
........................................................caaauucgguucuagCgaggu...................................  1     1  c09
........................................................caaauucgguucuagagCggu...................................  1     1  c09
........................................................caaauucgguuGuagagaggu...................................  1     1  c09
........................................................caaauucgguucuagagagUu...................................  4     1  c09
........................................................caaauucgguucuagGgaggu...................................  1     1  c09
........................................................caaauucgguucuagagaggA...................................  33    1  c09
........................................................caaauuUgguucuagagaggu...................................  2     1  c09
........................................................caaauucgguucuagagagCu...................................  7     1  c09
........................................................caaauucCguucuagagaggu...................................  5     1  c09
........................................................caaauucgguucuagagaggu...................................  4627  0  c09
........................................................caaauucgguucuaUagaggu...................................  3     1  c09
........................................................caaauucgguucuagagaAgu...................................  2     1  c09
........................................................caaauucgguucuagagaUgu...................................  2     1  c09
........................................................caaauucgguuAuagagaggu...................................  6     1  c09
........................................................caaauucgguucuagUgaggu...................................  1     1  c09
........................................................caaaAucgguucuagagaggu...................................  9     1  c09
........................................................caaauucgguCcuagagaggu...................................  4     1  c09
........................................................caaauucgguucAagagaggu...................................  1     1  c09
........................................................caaaCucgguucuagagaggu...................................  1     1  c09
........................................................caaaGucgguucuagagaggu...................................  4     1  c09
........................................................caaauuAgguucuagagaggu...................................  9     1  c09
........................................................caaauucgguuUuagagaggu...................................  1     1  c09
........................................................caaauucgguAcuagagaggu...................................  2     1  c09
........................................................caaauucgguucCagagaggu...................................  2     1  c09
........................................................caaauucgguucuagagaCgu...................................  4     1  c09
........................................................caaauucAguucuagagaggu...................................  1     1  c09
........................................................caaauucgguucuagagGggu...................................  1     1  c09
........................................................Aaaauucgguucuagagaggu...................................  3     1  c09
........................................................caaauucUguucuagagaggu...................................  3     1  c09
........................................................caaUuucgguucuagagaggu...................................  1     1  c09
........................................................caaauucgguucuagaCaggu...................................  2     1  c09
........................................................Uaaauucgguucuagagaggu...................................  1     1  c09
........................................................caaauucgguucGagagaggu...................................  1     1  c09
........................................................caaauucgguucuagaUaggu...................................  3     1  c09
........................................................caaauucggAucuagagaggu...................................  5     1  c09
........................................................caaauucgguucuagagaggC...................................  11    1  c09
........................................................caaauucggGucuagagaggu...................................  4     1  c09
........................................................caaauucgguucuaCagaggu...................................  6     1  c09
........................................................caaauucgguucuagagaUguu..................................  5     1  c09
........................................................caaauucgguucuagaCagguu..................................  1     1  c09
........................................................caaauucCguucuagagagguu..................................  1     1  c09
........................................................caaauucgCuucuagagagguu..................................  6     1  c09
........................................................caaauucgguucuagagaggGu..................................  7     1  c09
........................................................caaauucgguucuagagCgguu..................................  1     1  c09
........................................................caaauucggGucuagagagguu..................................  1     1  c09
........................................................caaauucgguucuagagagguC..................................  19    1  c09
........................................................caaauucgguucuaCagagguu..................................  3     1  c09
........................................................caaauucgguucuagagGgguu..................................  2     1  c09
........................................................caaaAucgguucuagagagguu..................................  4     1  c09
........................................................caaauuAgguucuagagagguu..................................  8     1  c09
........................................................cGaauucgguucuagagagguu..................................  1     1  c09
........................................................caaauucgguucuagagagUuu..................................  7     1  c09
........................................................caaauucgUuucuagagagguu..................................  4     1  c09
........................................................caaauucAguucuagagagguu..................................  1     1  c09
........................................................Uaaauucgguucuagagagguu..................................  5     1  c09
........................................................caaauucgguucuagagagguA..................................  55    1  c09
```

```
                                         Mature                        Star
caauuuuagugcccuacaucuacccuguagauccgaauuuguuugaagugaggcgacaaauucgguucuagagagguuugugguggugcaugcacugcaacguuucaagucug
........................................................caaauucgguucuagagagCuu..................................  8     1  c09
........................................................caaauucgguucuagaAagguu..................................  1     1  c09
........................................................caaauucgguucuagagaggAu..................................  8     1  c09
........................................................caaauucgguucuUgagagguu..................................  1     1  c09
........................................................caaauucgguucGagagagguu..................................  4     1  c09
........................................................caaauucgguucuGgagagguu..................................  1     1  c09
........................................................cCaauucgguucuagagagguu..................................  1     1  c09
........................................................caaauucgguucuagGgagguu..................................  4     1  c09
........................................................caaauucgguAcuagagagguu..................................  1     1  c09
........................................................caaauucgguCcuagagagguu..................................  1     1  c09
........................................................caaauucgguuUuagagagguu..................................  2     1  c09
........................................................caaauucgguucuagagaAguu..................................  1     1  c09
........................................................caaauucgguucuaUagagguu..................................  1     1  c09
........................................................caaUuucgguucuagagagguu..................................  1     1  c09
........................................................caaauucgguucuagagagguu..................................  4005  0  c09
........................................................caaauucggCucuagagagguu..................................  1     1  c09
........................................................caaauucgguucuCgagagguu..................................  1     1  c09
........................................................caaauucgguuAuagagagguu..................................  2     1  c09
........................................................caaauucgguucuagagagguG..................................  17    1  c09
........................................................caaauAcgguucuagagagguu..................................  5     1  c09
........................................................caaauucgguuGuagagagguu..................................  1     1  c09
........................................................caaauucgAuucuagagagguu..................................  1     1  c09
........................................................Aaaauucgguucuagagagguu..................................  6     1  c09
........................................................caaauucgguucuagCgagguu..................................  1     1  c09
........................................................caaauucgguucuagagaCguu..................................  9     1  c09
........................................................caaauucUguucuagagagguu..................................  1     1  c09
........................................................caaauucgguucuagUgagguu..................................  1     1  c09
........................................................caaaCucgguucuagagagguu..................................  1     1  c09
........................................................caaaGucgguucuagagagguu..................................  1     1  c09
........................................................caaauucgguucuagaUagguu..................................  2     1  c09
........................................................caaauucggCucuagagagguuu.................................  5     1  c09
........................................................caaauucAguucuagagagguuu.................................  6     1  c09
........................................................caaauucgguCcuagagagguuu.................................  5     1  c09
........................................................Gaaauucgguucuagagagguuu.................................  4     1  c09
........................................................caaauucgguucuagaUagguuu.................................  9     1  c09
........................................................cCaauucgguucuagagagguuu.................................  2     1  c09
........................................................caaauucgguucuaCagagguuu.................................  5     1  c09
........................................................caaauucgguucuUgagagguuu.................................  5     1  c09
........................................................caaGuucgguucuagagagguuu.................................  1     1  c09
........................................................caaauucgguucuagagagCuuu.................................  31    1  c09
........................................................caaauucgguucuagUgagguuu.................................  5     1  c09
........................................................caaauucgguuUuagagagguuu.................................  3     1  c09
........................................................Aaaauucgguucuagagagguuu.................................  15    1  c09
........................................................caaauucgguuGuagagagguuu.................................  2     1  c09
........................................................caaauucggGucuagagagguuu.................................  14    1  c09
........................................................caaauucgguucuagagCgguuu.................................  2     1  c09
........................................................caaaAucgguucuagagagguuu.................................  23    1  c09
........................................................caaauucgguGcuagagagguuu.................................  6     1  c09
........................................................caaauucgguucGagagagguuu.................................  2     1  c09
........................................................caaauCcgguucuagagagguuu.................................  5     1  c09
........................................................caaauuAgguucuagagagguuu.................................  28    1  c09
........................................................caaauucgguucuGgagagguuu.................................  5     1  c09
........................................................caaauucgguucuagagaUguuu.................................  14    1  c09
........................................................caGauucgguucuagagagguuu.................................  5     1  c09
........................................................caaaGucgguucuagagagguuu.................................  4     1  c09
........................................................caaauucgguucuagagaAguuu.................................  7     1  c09
........................................................caaaCucgguucuagagagguuu.................................  5     1  c09
........................................................caaauucgUuucuagagagguuu.................................  8     1  c09
........................................................caaauucgguucuagagagguuG.................................  17    1  c09
........................................................caaauucgguucuagagagguCu.................................  5     1  c09
........................................................caaauucgguucuagagaCguuu.................................  26    1  c09
........................................................caaauucgguucuagGgagguuu.................................  1     1  c09
........................................................caaUuucgguucuagagagguuu.................................  1     1  c09
........................................................caaauucgguucuagagagguuA.................................  33    1  c09
........................................................caaauucgCuucuagagagguuu.................................  17    1  c09
........................................................caaauucgguucuagagagguAu.................................  9     1  c09
........................................................caaauucgguucuagagaggAuu.................................  10    1  c09
........................................................caaauucgAuucuagagagguuu.................................  3     1  c09
........................................................caaauucgguucuagaAagguuu.................................  1     1  c09
........................................................caaauucgguucuagagUgguuu.................................  4     1  c09
```

```
                    Mature                          Star
caauuuuagugcccuacaucuacccuguagauccgaauuuguuugaagugaggcgacaaauucgguucuagagagguuuguguggugcaugcacugcaacguuucaagucug
........................................................caaauucgguucCagagagguuu.................................	5	1	c09
........................................................caaauucgguucAagagagguuu.................................	3	1	c09
........................................................caaauucgguucuagagagguGu.................................	8	1	c09
........................................................caaauAcgguucuagagagguuu.................................	6	1	c09
........................................................caaauucgguucuaUagagguuu.................................	6	1	c09
........................................................caaauucgguAcuagagagguuu.................................	4	1	c09
........................................................caUauucgguucuagagagguuu.................................	1	1	c09
........................................................caaauucgguucuagagaggGuu.................................	10	1	c09
........................................................Uaaauucgguucuagagagguuu.................................	12	1	c09
........................................................caaauucgguucuagagaggCuu.................................	3	1	c09
........................................................caaauucCguucuagagagguuu.................................	18	1	c09
........................................................caaauucgguucuagagagAuuu.................................	3	1	c09
........................................................caaauucgguucuagagagUuuu.................................	11	1	c09
........................................................cGaauucgguucuagagagguuu.................................	4	1	c09
........................................................caaauuGgguucuagagagguuu.................................	1	1	c09
........................................................caaauuUgguucuagagagguuu.................................	6	1	c09
........................................................caaauucggAucuagagagguuu.................................	10	1	c09
........................................................caaauucgguucuagaCagguuu.................................	16	1	c09
........................................................caaauucgguucuagagGgguuu.................................	6	1	c09
........................................................caaauucgguuAuagagagguuu.................................	24	1	c09
........................................................caaauucUguucuagagagguuu.................................	7	1	c09
........................................................caaauucgguucuagagagguuC.................................	22	1	c09
........................................................caaauGcgguucuagagagguuu.................................	1	1	c09
........................................................cUaauucgguucuagagagguuu.................................	4	1	c09
........................................................caaauucgguucuagagagguuu.................................	18409	0	c09
........................................................caaauucgguucuaAagagguuug................................	1	1	c09
........................................................caaauucggCucuagagagguuug................................	1	1	c09
........................................................caaauucgguucuagagaUguuug................................	1	1	c09
........................................................caaauucgguucuagagagguuuA................................	176	1	c09
........................................................caaauucggGucuagagagguuug................................	1	1	c09
........................................................caaauucgguucuagagagguuGg................................	2	1	c09
........................................................caaauucgguucuagagagguuuU................................	1569	1	c09
........................................................caaauucgguucuagagagguuuC................................	46	1	c09
........................................................caaauucgguucuagagagguuug................................	554	0	c09
........................................................caaauucgguucuagagagUuuug................................	1	1	c09
........................................................caaauucgguucuagagaCguuug................................	2	1	c09
........................................................caaauucgguucuagagagguuAg................................	2	1	c09
........................................................caaauucgguucuagagaggCuug................................	1	1	c09
........................................................caaauucAguucuagagagguuug................................	1	1	c09
........................................................caaauucgAuucuagagagguuug................................	1	1	c09
........................................................caaauucgguucuaUagagguuug................................	1	1	c09
........................................................caaauuAgguucuagagagguuug................................	1	1	c09
........................................................caaauucggAucuagagagguuug................................	1	1	c09
........................................................caaauucgguucuagaUagguuug................................	1	1	c09
........................................................caaauucgguucuagagagguuugA...............................	33	1	c09
........................................................caaauucgguucuagagagguuugC...............................	3	1	c09
........................................................caaaAucgguucuagagagguuugu...............................	1	1	c09
........................................................caaauucgguucuagagagguuugG...............................	15	1	c09
........................................................caaauucgguucuagagagguuuUu...............................	142	1	c09
........................................................caaauucgguucuagagagguuuCu...............................	4	1	c09
........................................................caaauucgguucuagagagguuGgu...............................	2	1	c09
........................................................caaauucgguucuaUagagguuugu...............................	1	1	c09
........................................................caaauucgguucuagagagguuuAu...............................	13	1	c09
........................................................caaauucgguucuagagagguuugu...............................	79	0	c09
........................................................caaauucgguucuagagagguuuguU..............................	7	1	c09
........................................................caaauucgguucuagagagguuugGg..............................	1	1	c09
........................................................caaauucgguucuagagagguuugug..............................	1	0	c09
........................................................caaauucgguucuagagagguuuUug..............................	2	1	c09
........................................................caaauucgguucuagagagguuuguA..............................	2	1	c09
........................................................caaauucgguucuagagagguuuguC..............................	1	1	c09
........................................................caaauucgguucuagagagguuugCgu.............................	1	1	c09
........................................................caaauucgguucuagagagguuuCugu.............................	1	1	c09
........................................................caaauucgguucuagagagguuuguUu.............................	1	1	c09
........................................................caaauucgguucuagagagguuugCgug............................	1	1	c09
........................................................caaauucgguucuagagagguuuguCugg...........................	1	1	c09
........................................................caaauucgguucuagagagguuugugugg...........................	1	0	c09
.........................................................aaauAcgguucuagagag.....................................	1	1	c09
.........................................................aaauucgguucuagagag.....................................	238	0	c09
.........................................................Caauucgguucuagagag.....................................	4	1	c09
.........................................................aaauucgguucuagagaC.....................................	1	1	c09
```

Mature Star

```
caauuuuagugcccuacaucuacccuguagauccgaauuuguuugaagugaggcgacaaauucgguucuagagagguuuguguggugcaugcacugcaacguuucaagucug
.........................................................aaauucgguuAuagagag.....................................	1	1	c09
.........................................................aaauucgCuucuagagag.....................................	1	1	c09
.........................................................aaauucgguucuagagaA.....................................	1	1	c09
.........................................................aaauucgguucuagagaU.....................................	1	1	c09
.........................................................Uaauucgguucuagagag.....................................	1	1	c09
.........................................................aaauucgguuAuagagagg....................................	1	1	c09
.........................................................aaauucUguucuagagagg....................................	1	1	c09
.........................................................aaauucgguucuagagagg....................................	781	0	c09
.........................................................aaauucgguucuagagaUg....................................	1	1	c09
.........................................................aaaAucgguucuagagagg....................................	1	1	c09
.........................................................aaUuucgguucuagagagg....................................	1	1	c09
.........................................................aGauucgguucuagagagg....................................	1	1	c09
.........................................................aaauucgguucuagagagU....................................	1	1	c09
.........................................................aaauAcgguucuagagagg....................................	2	1	c09
.........................................................aaauuAgguucuagagagg....................................	3	1	c09
.........................................................Caauucgguucuagagagg....................................	15	1	c09
.........................................................Gaauucgguucuagagagg....................................	1	1	c09
.........................................................aaauucgUuucuagagagg....................................	1	1	c09
.........................................................aaauucgguucuagagagC....................................	1	1	c09
.........................................................Caauucgguucuagagaggu...................................	16	1	c09
.........................................................aaauucgguucuagagaggC...................................	8	1	c09
.........................................................aaauucgguucAagagaggu...................................	1	1	c09
.........................................................aaauucgguuAuagagaggu...................................	2	1	c09
.........................................................aaauucgguucuUgagaggu...................................	1	1	c09
.........................................................aaauucgguucuaCagaggu...................................	3	1	c09
.........................................................aaauucgguucuagagagCu...................................	1	1	c09
.........................................................aaauucgguucuagagagUu...................................	2	1	c09
.........................................................aaauucgguucuagaUaggu...................................	1	1	c09
.........................................................aaauucgguucuagaCaggu...................................	2	1	c09
.........................................................aaauuAgguucuagagaggu...................................	3	1	c09
.........................................................aaauucgguucGagagaggu...................................	1	1	c09
.........................................................aUauucgguucuagagaggu...................................	1	1	c09
.........................................................aaauucgguucuagagaggu...................................	1178	0	c09
.........................................................aaauucggAucuagagaggu...................................	1	1	c09
.........................................................aaauucgguucuGgagaggu...................................	1	1	c09
.........................................................aaauucgguucuagagaggG...................................	2	1	c09
.........................................................Gaauucgguucuagagaggu...................................	1	1	c09
.........................................................aaauucgguucuagagUggu...................................	1	1	c09
.........................................................aaauucgguucuaUagaggu...................................	1	1	c09
.........................................................aaaCucgguucuagagaggu...................................	1	1	c09
.........................................................aaauucgguAcuagagaggu...................................	2	1	c09
.........................................................aaauucUguucuagagaggu...................................	1	1	c09
.........................................................aaGuucgguucuagagaggu...................................	1	1	c09
.........................................................aaauucgguucuUgagagguu..................................	2	1	c09
.........................................................aaauucgguGcuagagagguu..................................	1	1	c09
.........................................................aaauuAgguucuagagagguu..................................	5	1	c09
.........................................................aaauucgguucuaUagagguu..................................	1	1	c09
.........................................................aaauucgguucGagagagguu..................................	2	1	c09
.........................................................aaauucgguucuagGgagguu..................................	1	1	c09
.........................................................aaauucgguucuagagaCguu..................................	4	1	c09
.........................................................aaauucgguucuagagagguG..................................	5	1	c09
.........................................................aaauucgguuGuagagagguu..................................	2	1	c09
.........................................................aaauucgguucuagagaggCu..................................	2	1	c09
.........................................................aaauucgguucuaCagagguu..................................	5	1	c09
.........................................................aaauucgguucuagUgagguu..................................	1	1	c09
.........................................................aaauucggGucuagagagguu..................................	3	1	c09
.........................................................aaauucgguucuagagagguA..................................	5	1	c09
.........................................................aaauucUguucuagagagguu..................................	1	1	c09
.........................................................aaauucCguucuagagagguu..................................	1	1	c09
.........................................................Caauucgguucuagagagguu..................................	19	1	c09
.........................................................aaauucgguucuagagagguC..................................	7	1	c09
.........................................................aaauucgguuUuagagagguu..................................	1	1	c09
.........................................................aaaAucgguucuagagagguu..................................	2	1	c09
.........................................................aaauAcgguucuagagagguu..................................	1	1	c09
.........................................................aaUuucgguucuagagagguu..................................	1	1	c09
.........................................................aaauucgguucuagagaggAu..................................	2	1	c09
.........................................................aaauucgguucuagagagguu..................................	1936	0	c09
.........................................................aaauucggCucuagagagguu..................................	1	1	c09
.........................................................aaauucgguucuagagaggGu..................................	1	1	c09
.........................................................aaauucgCuucuagagagguu..................................	2	1	c09
```

Mature **Star**

caauuuuagugcccuacaucuacccuguagauccgaauuuguuugaagugaggcgacaaauucgguucuagagagguuuguguggugcaugcacugcaacguuucaagucug			
....aaauucgguucuagagagUuu..................................	1	1	c09
....aaaGucgguucuagagagguu..................................	1	1	c09
....aaauucgguucuUgagagguuu.................................	1	1	c09
....aaauucgguucuagUgagguuu.................................	2	1	c09
....aaauucgguucuagagaUguuu.................................	5	1	c09
....aaauucUguucuagagagguuu.................................	7	1	c09
....aaauucgguucuagagagUuuu.................................	4	1	c09
....aaauucgguucuagagGgguuu.................................	1	1	c09
....aaauucggGucuagagagguuu.................................	7	1	c09
....aaauucgguucCagagagguuu.................................	2	1	c09
....aaauCcgguucuagagagguuu.................................	3	1	c09
....aaauucgguucuaUagagguuu.................................	2	1	c09
....Caauucgguucuagagagguuu.................................	74	1	c09
....aaauucgguucuagagagguuu.................................	5810	0	c09
....Gaauucgguucuagagagguuu.................................	1	1	c09
....aaauucgguucAagagagguuu.................................	1	1	c09
....aaauucgguucuagaUagguuu.................................	4	1	c09
....aaauuUgguucuagagagguuu.................................	1	1	c09
....aaauucCguucuagagagguuu.................................	3	1	c09
....aaauucgguucuagagagguGu.................................	1	1	c09
....aaGuucgguucuagagagguuu.................................	2	1	c09
....aaauucgguucuaCagagguuu.................................	5	1	c09
....aaauucgguucuagagagguuG.................................	2	1	c09
....aaauucgguuAuagagagguuu.................................	2	1	c09
....aaauucgguucuagagagCuuu.................................	14	1	c09
....aaauucgguucuagagaAguuu.................................	1	1	c09
....aaauucgguCcuagagagguuu.................................	1	1	c09
....aaauucgguucuagagaggAuu.................................	1	1	c09
....aaauucgguucuagagagguuC.................................	15	1	c09
....aaauucgguucuagaCagguuu.................................	5	1	c09
....aaauucgguucGagagagguuu.................................	1	1	c09
....aGauucgguucuagagagguuu.................................	1	1	c09
....aaauucgUuucuagagagguuu.................................	1	1	c09
....aaauucggAucuagagagguuu.................................	1	1	c09
....aaauucgguuGuagagagguuu.................................	2	1	c09
....aaauucgguucuGgagagguuu.................................	6	1	c09
....aaauucgguuUuagagagguuu.................................	2	1	c09
....aaaAucgguucuagagagguuu.................................	7	1	c09
....aaauucAguucuagagagguuu.................................	3	1	c09
....aaauucgguucuagagaggCuu.................................	3	1	c09
....aaauucgCuucuagagagguuu.................................	9	1	c09
....aCauucgguucuagagagguuu.................................	1	1	c09
....aaauucgguucuagagCgguuu.................................	3	1	c09
....aaauucgguucuagagagguuA.................................	21	1	c09
....aaauucgguucuagGgagguuu.................................	1	1	c09
....aaauuAgguucuagagagguuu.................................	16	1	c09
....aaauucgguucuagagaCguuu.................................	3	1	c09
....aaauucgguucuagagaggGuu.................................	1	1	c09
....aaauucgguucuagagagguCu.................................	1	1	c09
....aaaCucgguucuagagagguuu.................................	1	1	c09
....aaauucgguucuagagagguAu.................................	5	1	c09
....aaUuucgguucuagagagguuu.................................	4	1	c09
....aaaGucgguucuagagagguuu.................................	1	1	c09
....aaauAcgguucuagagagguuu.................................	2	1	c09
....aaauucggGucuagagagguuug................................	2	1	c09
....aaauucgguucuagagagguuuC................................	43	1	c09
....aaauucgUuucuagagagguuug................................	1	1	c09
....aaauucgguucuaAagagguuug................................	1	1	c09
....aaauucgguucuGgagagguuug................................	2	1	c09
....aaauucgCuucuagagagguuug................................	2	1	c09
....aaauucgguucuagaCagguuug................................	1	1	c09
....Caauucgguucuagagagguuug................................	26	1	c09
....aaauucgguucuagagagguCug................................	2	1	c09
....aaauucgguucuagagaggAuug................................	1	1	c09
....aaauucgguucuagagagguuuU................................	1358	1	c09
....Gaauucgguucuagagagguuug................................	2	1	c09
....aaauuUgguucuagagagguuug................................	1	1	c09
....aaauucgguuAuagagagguuug................................	1	1	c09
....aaauucgguuUuagagagguuug................................	1	1	c09
....aaauucCguucuagagagguuug................................	2	1	c09

Mature **Star**

caauuuuagugcccuacaucuacccuguagauccgaauuuguuugaagugaggcgacaaauucgguucuagagagguuuguguggugcaugcacugcaacguuucaagucug			
....aaauucgguucuagagagguuAg................................	1	1	c09
....aaauucggAucuagagagguuug................................	2	1	c09
....aaauucgguucuagagagguGug................................	1	1	c09
....aaauucggCucuagagagguuug................................	3	1	c09
....aaauAcgguucuagagagguuug................................	1	1	c09
....aaauucgguucuagagagCuuug................................	6	1	c09
....aaauuAgguucuagagagguuug................................	5	1	c09
....aaauucgguucuaCagagguuug................................	1	1	c09
....aaauucgguucuagagaUguuug................................	3	1	c09
....aaauucgguucuagagagguuug................................	2225	0	c09
....aaauucgguucuagagagUuuug................................	3	1	c09
....aaauucUguucuagagagguuug................................	1	1	c09
....aaaCucgguucuagagagguuug................................	1	1	c09
....aaauucgguucuagagGgguuug................................	1	1	c09
....aaauCcgguucuagagagguuug................................	1	1	c09
....aaauucgguucuagagagguuuA................................	103	1	c09
....aaauucgguuGuagagagguuug................................	1	1	c09
....aaaGucgguucuagagagguuug................................	1	1	c09
....aaauucgguucuagagaCguuug................................	1	1	c09
....aaauucgguucuagagagguuCg................................	1	1	c09
....Uaauucgguucuagagagguuug................................	1	1	c09
....aaauucgguucuagagagguuugu...............................	179	0	c09
....aaauucgguucuagagagguuugG...............................	20	1	c09
....Caauucgguucuagagagguuugu...............................	1	1	c09
....aaauucgguucuagagagguuugC...............................	9	1	c09
....aaauucgguucuGgagagguuugu...............................	1	1	c09
....aaauucgguucuagagagguuGgu...............................	1	1	c09
....aaauucggAucuagagagguuugu...............................	1	1	c09
....aaauucgguucuagagagguuuCu...............................	4	1	c09
....aaauucggGucuagagagguuugu...............................	1	1	c09
....aaauuAgguucuagagagguuugu...............................	1	1	c09
....aaauucgguucuagagagguuugA...............................	75	1	c09
....aaauucgguucuagGgagguuugu...............................	1	1	c09
....aaauucgguucuagagagguuuAu...............................	17	1	c09
....aaauucgguucuagagagguuuUu...............................	77	1	c09
....aaauucgguucuagagagguuuguA..............................	7	1	c09
....aaauucgguucuagagagguuuguC..............................	2	1	c09
....aaauucgguucuagagagguuuAug..............................	1	1	c09
....aaauucgguucuagagagguuuguU..............................	6	1	c09
....aaauucgguucuagagagguuuUug..............................	3	1	c09
....aaauucgguucuagagagguuuguUu.............................	1	1	c09
....aaauucgguucuagagagguuuguAu.............................	6	1	c09
....aaauucgguucuagagagguuugugugU...........................	1	1	c09
....aauucgguucuagagagg....................................	11	0	c09
....Cauucgguucuagagagg....................................	2	1	c09
....aauucgguucuagagaggu...................................	10	0	c09
....Gauucgguucuagagaggu...................................	1	1	c09
....Cauucgguucuagagagguu..................................	2	1	c09
....aauucgguucuagagagguu..................................	15	0	c09
....aauucgguuAuagagagguu..................................	1	1	c09
....aaAucgguucuagagagguu..................................	1	1	c09
....aauucgguucuagagagAuu..................................	1	1	c09
....Gauucgguucuagagagguuu.................................	2	1	c09
....aauucgguucuagagagguuu.................................	47	0	c09
....Cauucgguucuagagagguuu.................................	2	1	c09
....aauucgguucuagagagguuug................................	10	0	c09
....aauucgguucuagagagguuuA................................	4	1	c09
....aUuucgguucuagagagguuug................................	1	1	c09
....aauucgguucuagagagguuuU................................	6	1	c09
....aauucgguucuagagagguuugC...............................	1	1	c09
....aauucgguucuagagagguuuUu...............................	1	1	c09
....aauucgguucuagagagguuugu...............................	1	0	c09
....auucgguucuagagagCu...................................	1	1	c09
....aAucgguucuagagaggu...................................	1	1	c09
....aCucgguucuagagaggu...................................	1	1	c09
....auucgUuucuagagaggu...................................	1	1	c09
....auucgguucuagUgaggu...................................	1	1	c09
....Uuucgguucuagagaggu...................................	1	1	c09
....auucgguucuagagaggu...................................	743	0	c09
....auucgguucuagagCggu...................................	1	1	c09

Mature Star

caauuuuagugcccuacaucuacccuguagauccgaauuuguuugaagugaggcgacaaauucgguucuagagagguuugugguggugcaugcacugcaacguuucaagucug			
....auCcgguucuagagaggu....................................	1	1	c09
....auucgguucuaUagaggu....................................	1	1	c09
....auucgguucCagagaggu....................................	1	1	c09
....auuUgguucuagagaggu....................................	2	1	c09
....auucgAuucuagagaggu....................................	1	1	c09
....auucgguuAuagagaggu....................................	2	1	c09
....Cuucgguucuagagaggu....................................	29	1	c09
....auucgguucuagagaUgu....................................	1	1	c09
....auucgguucuagagaggG....................................	3	1	c09
....auucgguucuagaUaggu....................................	1	1	c09
....auucgguucuagagUggu....................................	1	1	c09
....auucgguucuagagaggA....................................	11	1	c09
....auucgCuucuagagaggu....................................	2	1	c09
....auuAgguucuagagaggu....................................	2	1	c09
....auucgguucuagagaAgu....................................	1	1	c09
....auCcgguucuagagagguu...................................	1	1	c09
....auucgguucuagagaggGu...................................	1	1	c09
....auucgguucuGgagagguu...................................	1	1	c09
....auucgguucuagagagguC...................................	2	1	c09
....auucgguucuagagagCuu...................................	3	1	c09
....auucgguucuagagaUguu...................................	1	1	c09
....auuAgguucuagagagguu...................................	2	1	c09
....Guucgguucuagagagguu...................................	1	1	c09
....auucggAucuagagagguu...................................	1	1	c09
....auucgguucuaCagagguu...................................	3	1	c09
....auucgguucuagagaCguu...................................	1	1	c09
....auucgguucuagagagguA...................................	3	1	c09
....auucgguucuagUgagguu...................................	1	1	c09
....Cuucgguucuagagagguu...................................	31	1	c09
....auucggGucuagagagguu...................................	1	1	c09
....auucgguucuagagagguG...................................	1	1	c09
....auucgguucuagagagguu...................................	880	0	c09
....auucgguuGuagagagguuu..................................	1	1	c09
....auuUgguucuagagagguuu..................................	1	1	c09
....auucggGucuagagagguuu..................................	1	1	c09
....auucgguucuagagaCguuu..................................	2	1	c09
....auucgUuucuagagagguuu..................................	2	1	c09
....auucgguucuagagaggAuu..................................	2	1	c09
....auucggAucuagagagguuu..................................	3	1	c09
....auucgguucuagCgagguuu..................................	1	1	c09
....auucgAuucuagagagguuu..................................	1	1	c09
....auucCguucuagagagguuu..................................	3	1	c09
....auucgguucuagagagguCu..................................	1	1	c09
....auucgguCcuagagagguuu..................................	2	1	c09
....auucgguuAuagagagguuu..................................	3	1	c09
....auucgguucuagUgagguuu..................................	1	1	c09
....auucUguucuagagagguuu..................................	2	1	c09
....auucgCuucuagagagguuu..................................	4	1	c09
....aCucgguucuagagagguuu..................................	2	1	c09
....auucgguucuagagaggGuu..................................	2	1	c09
....auucgguucCagagagguuu..................................	1	1	c09
....auucgguucuaUagagguuu..................................	1	1	c09
....auuGgguucuagagagguuu..................................	1	1	c09
....auAcgguucuagagagguuu..................................	1	1	c09
....auucgguucuagagagguuu..................................	3109	0	c09
....auucgguucuCgagagguuu..................................	2	1	c09
....auucgguucuagagagUuuu..................................	3	1	c09
....auucgguucuagGgagguuu..................................	2	1	c09
....auucgguucuagagagCuuu..................................	5	1	c09
....Cuucgguucuagagagguuu..................................	121	1	c09
....auucgguucuaCagagguuu..................................	5	1	c09
....auuAgguucuagagagguuu..................................	5	1	c09
....auucgguucGagagagguuu..................................	2	1	c09
....aAucgguucuagagagguuu..................................	3	1	c09
....auucgguuUuagagagguuu..................................	1	1	c09
....Uuucgguucuagagagguuu..................................	4	1	c09
....auucggCucuagagagguuu..................................	2	1	c09
....Guucgguucuagagagguuu..................................	1	1	c09
....auucgguucuagagagguuG..................................	2	1	c09
....auucgguucuagagagguuC..................................	4	1	c09

Mature Star

caauuuuagugcccuacaucuacccuguagauccgaauuuguuugaagugaggcgacaaauucgguucuagagagguuuguguggugcaugcacugcaacguuucaagucug			
....auucgguucuagagaUguuu.................................	2	1	c09
....auucAguucuagagagguuu.................................	2	1	c09
....auucgguucAagagagguuu.................................	1	1	c09
....auucgguucuagagaggCuu.................................	2	1	c09
....auucgguucuagagaAguuu.................................	1	1	c09
....auucgguucuagagUgguuu.................................	2	1	c09
....auucgguucuagaCagguuu.................................	3	1	c09
....auucgguucuagagagguuA.................................	4	1	c09
....auucgguucuagagagguAu.................................	3	1	c09
....auucgguucAagagagguuug................................	1	1	c09
....auucgguucuagagagguuug................................	154	0	c09
....Guucgguucuagagagguuug................................	1	1	c09
....auucgguucuagagagguuuA................................	36	1	c09
....auucgguucuagagagCuuug................................	1	1	c09
....auucgguucuagaUagguuug................................	1	1	c09
....auucgCuucuagagagguuug................................	1	1	c09
....auucgguucuagagagguuuC................................	17	1	c09
....auucgguucuagagagguuuU................................	273	1	c09
....Cuucgguucuagagagguuug................................	8	1	c09
....auucgguucuagagagguuuAu...............................	5	1	c09
....auucgguucuagagagguuugA...............................	4	1	c09
....auucgguucuagagagguuugG...............................	1	1	c09
....auucgguucuagagagguuuCu...............................	1	1	c09
....auucgguucuagagagguuuUu...............................	21	1	c09
....auucgguucuagagagguuugu...............................	13	0	c09
....auucgguucuagagagguuGgu...............................	1	1	c09
....auucgguucuagagagguuugC...............................	1	1	c09
....auucgguucuagagagguuuguU..............................	1	1	c09
....auucgguucuagagagguuuguCu.............................	1	1	c09
....auucgguucuagagagguuuUugu.............................	1	1	c09
....uucgguucuagagagguA..................................	1	1	c09
....uuGgguucuagagagguu..................................	1	1	c09
....Aucgguucuagagagguu..................................	1	1	c09
....Gucgguucuagagagguu..................................	7	1	c09
....uucgguucuagagagguu..................................	111	0	c09
....uucgguucuagagagguC..................................	1	1	c09
....uucgguucAagagagguuu.................................	1	1	c09
....Aucgguucuagagagguuu.................................	1	1	c09
....uucggCucuagagagguuu.................................	1	1	c09
....uucggAucuagagagguuu.................................	1	1	c09
....Gucgguucuagagagguuu.................................	31	1	c09
....uucgguucuagagagguuu.................................	307	0	c09
....uucgguucuagagagguuA.................................	2	1	c09
....uucgguucuagagagguuuU................................	44	1	c09
....uucgguucuagagagguuug................................	77	0	c09
....Cucgguucuagagagguuug................................	1	1	c09
....Gucgguucuagagagguuug................................	2	1	c09
....uucgguucuagagagguuuA................................	2	1	c09
....uucgguucuagagagguuugu...............................	7	0	c09
....uucgguucuagagagguuuUu...............................	3	1	c09
....uucgguucuagagagguuugG...............................	1	1	c09
....uucgguucuagagagguuuAu...............................	1	1	c09
....uucgguucuagagagguuugA...............................	4	1	c09
....Gucgguucuagagagguuugu...............................	1	1	c09
....uucgguucuagagagguuugAg..............................	1	1	c09
....uucgguucuagagagguuuguC..............................	1	1	c09
....ucgguucuagagagguuu.................................	83	0	c09
....ucgguucuagagaggCuu.................................	1	1	c09
....Ccgguucuagagagguuu.................................	1	1	c09
....Gcgguucuagagagguuu.................................	3	1	c09
....ucgguucuagagagCuuug................................	1	1	c09
....ucgguucuagagagguuug................................	16	0	c09
....ucgguucuagagagguuuA................................	1	1	c09
....ucgguucuagagagguuuU................................	11	1	c09
....ucgguucuagagagguuugu...............................	2	0	c09
....ucgguucuagagagguuuUu...............................	2	1	c09
....cgguucuagagagguuuC................................	1	1	c09
....cgguucuagagagguuuU................................	13	1	c09
....cgguucuagagagguuug................................	9	0	c09
....cgguucuagagagguuugu...............................	2	0	c09

```
                     Mature                              Star
caauuuuagugcccuacaucuacccuguagauccgaauuuguuugaagugaggcgacaaauucgguucuagagagguuuguguggugcaugcacugcaacguuucaagucug
..............................................................cgguucuagagagguuuUu...............................	2	1	c09
..............................................................cgguucuagagagguuugG...............................	1	1	c09
................................................................guucuagagagguuuguA..............................	1	1	c09
```

Provisional ID	:	group0_61
Score total	:	159.6
Score for star read(s)	:	3.9
Score for read counts	:	153.1
Score for mfe	:	1.6
Score for randfold	:	1.6
Score for cons. seed	:	-0.6
Total read count	:	312
Mature read count	:	154
Loop read count	:	0
Star read count	:	158

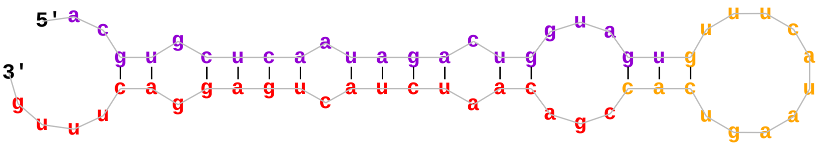

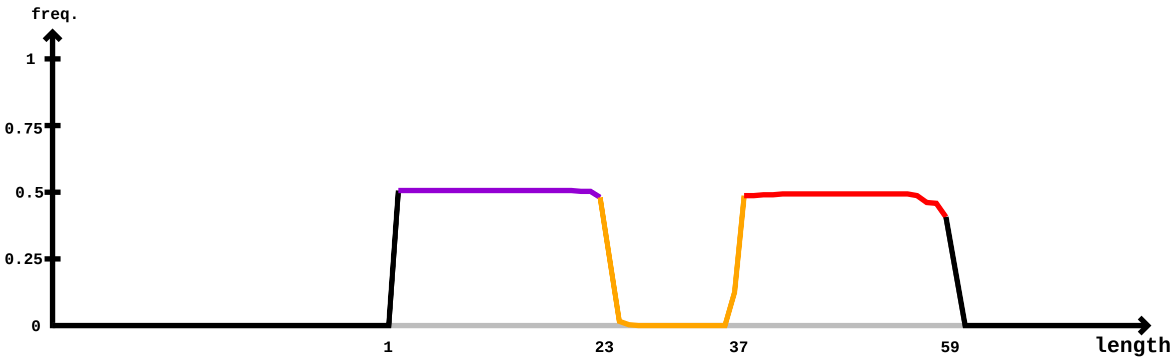


```
                                   Star                              Mature
5'- aaagaagagucuucagcauuuaagugccugcguaacgugcucaauagacugguaguguuucauaagucaccgacaaucuacugaggacuuugcgcagggacuuuauuucugc   -3'   obs
    aaagaagagucuucagcauuuaagugccugcguaacgugcucaauagacugguaguguuucauaagucaccgacaaucuacugaggacuuugcgcagggacuuuauuucugc         exp
    .((((....))))(((.....((((.(((((((((.((.((((.((((.((...(((..........)))...)).)))).)))).)).))))))))).)))).....))).   reads   mm   sample
    ..................................acgugcucaauagacugguag.........................................................   2       0    c02
    ..................................Ccgugcucaauagacugguag.........................................................   1       1    c02
    ..................................acCugcucaauagacugguagu........................................................   1       1    c02
    ..................................acgugcucaauagacugguagu........................................................   29      0    c02
    ..................................Ccgugcucaauagacugguagu........................................................   8       1    c02
    ..................................acgugcuAaauagacugguagu........................................................   1       1    c02
    ..................................aAgugcucaauagacugguagu........................................................   1       1    c02
    ..................................Ccgugcucaauagacugguagug.......................................................   2       1    c02
    ..................................acgugcucaauagacugguagug.......................................................   2       0    c02
    ..................................acgugcucaauagacugguagugu......................................................   1       0    c02
    .....................................................................ccgacaaucuacugaggac........................   2       0    c02
    .....................................................................ccgacaaucuacugaggacu.......................   2       0    c02
    .....................................................................ccgacaaucuacugaggacuuu.....................   5       0    c02
    .....................................................................ccgacaaucuacugaggacuuug....................   20      0    c02
    .....................................................................ccgacaaucuacugagUacuuug....................   1       1    c02
    .....................................................................ccgaGaaucuacugaggacuuug....................   1       1    c02
    ......................................................................cgacaaucuacugaggacu.......................   2       0    c02
    ......................................................................cgacaaucuacugaggacuuu.....................   8       0    c02
    ......................................................................cAacaaucuacugaggacuuug....................   1       1    c02
    ......................................................................cgacaaucuacugaggacuuug....................   65      0    c02
    ......................................................................cgacaaucuacugaggacuuugA...................   1       1    c02
    ......................................................................cgacaaucuacugaggacuuugU...................   1       1    c02
    ........................................................................acaaucuacugaggacuuu.....................   1       0    c02

    ..................................acgugcucaauagacugguag.........................................................   1       0    c15
    ..................................acgugcucaauagacugguagug.......................................................   1       0    c15
    ..................................acgugcucaauagacugguagugu......................................................   1       0    c15
    ..................................acgugcucaauagacugguagugAu.....................................................   1       1    c15

    ..................................acgugcucaauagacugguagu........................................................   12      0    c09
    ..................................Ucgugcucaauagacugguagu........................................................   1       1    c09
    ..................................acgugcucaauagacugguagug.......................................................   1       0    c09
    ..................................acgugcucaauagacugguaguU.......................................................   1       1    c09
```

Star Mature

```
aaagaagagucuucagcauuuaagugccugcguaacgugcucaauagacugguaguguuucauaagucaccgacaaucuacugaggacuuugcgcagggacuuuauuucugc
..................................Ccgugcucaauagacugguagug.......................................................   1   1   c09
.....................................................................ccgacaaucuacugaggacu.......................   3   0   c09
.....................................................................ccgacaaucuacugaggacuuug....................   2   0   c09
......................................................................cgacaaucuacugaggacu.......................   1   0   c09
......................................................................cgacaaucuacugaggacuu......................   1   0   c09
......................................................................cgacaaucuacugaggacuuu.....................   2   0   c09
......................................................................cgacaaucuacugaggacuuug....................   18  0   c09
......................................................................cgacaaucuacugaggacuuugG...................   1   1   c09

..................................acgugcucaauagacuggu...........................................................   1   0   c16
..................................acgugcucaauagacugguag.........................................................   2   0   c16
..................................Ccgugcucaauagacugguag.........................................................   1   1   c16
..................................Ccgugcucaauagacugguagu........................................................   14  1   c16
..................................acgugcucaauagacugguagu........................................................   37  0   c16
..................................acgugcucaauagacugCuagu........................................................   1   1   c16
..................................acgugAucaauagacugguagu........................................................   1   1   c16
..................................acgugcucaauagacugguagug.......................................................   18  0   c16
..................................acgugcuAaauagacugguagug.......................................................   1   1   c16
..................................Ccgugcucaauagacugguagug.......................................................   6   1   c16
..................................acgugcucaauagacugCuagug.......................................................   1   1   c16
..................................acgugcucaauagacugguagugu......................................................   1   0   c16
..................................acgugcucaauagacugguaguUu......................................................   1   1   c16
.....................................................................ccgacaaucuacugaggacuuug....................   3   0   c16
......................................................................cgacaaucuacugaggacuuug....................   11  0   c16
......................................................................cgacaaucuacugaggacuAug....................   1   1   c16
..........................................................................aaucuacugaggacuuug....................   1   0   c16

..................................Ccgugcucaauagacugguagu........................................................   1   1   c04
..................................acgugcucaauagacugguagu........................................................   3   0   c04
..................................acgugcucaauagacugguagug.......................................................   1   0   c04
```

Provisional ID	:	group0_621
Score total	:	132.3
Score for star read(s)	:	3.9
Score for read counts	:	126.1
Score for mfe	:	1.4
Score for randfold	:	1.6
Score for cons. seed	:	-0.6
Total read count	:	259
Mature read count	:	189
Loop read count	:	0
Star read count	:	70

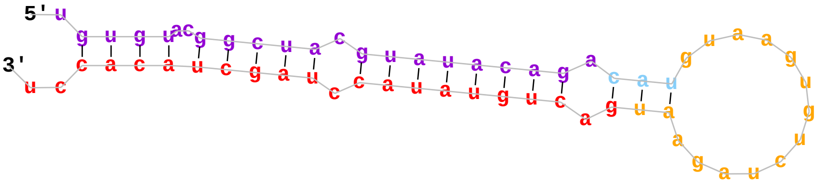

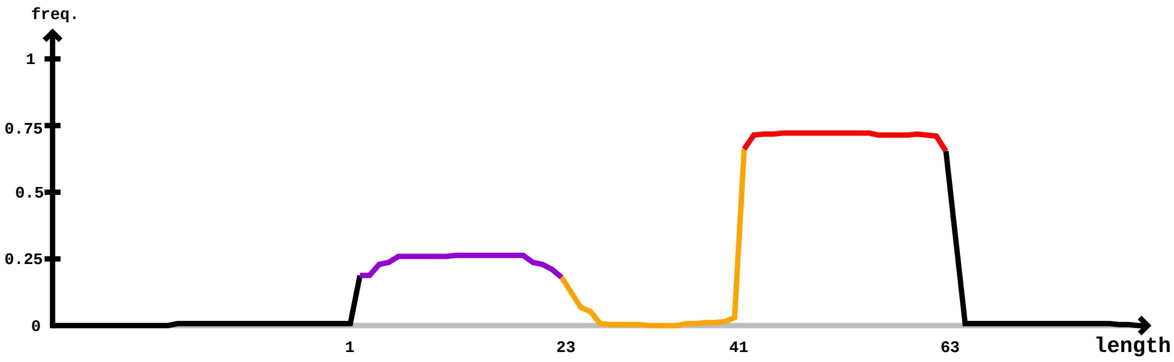


```
                                  Star                                        Mature
5'- ugaagcaagaagcgcuuucuuguguauaaguguguacggcuacguauacagacauguaagugucuagaaugacuguauaccuagcuacaccucguaugcaaggacacacacc   -3'   obs
    ugaagcaagaagcgcuuucuuguguauaaguguguacggcuacguauacagacauguaagugucuagaaugacuguauaccuagcuacaccucguaugcaaggacacacacc         exp
    .(((((.......)))))(((((((((.((.((((..(((((.((((((((.(((..............))).)))))))).))))))))))).))))))))).........   reads   mm   sample
    ..............................uguguacggcuacguaua................................................................   2       0    c04
    ..............................Gguguacggcuacguaua................................................................   1       1    c04
    ..............................uguguacggcuacguauac...............................................................   1       0    c04
    ..............................uguguacggcuacguauacaC.............................................................   1       1    c04
    ..............................uguguacggcuaUguauacag.............................................................   1       1    c04
    ..............................uguguacggcuacguauacag.............................................................   2       0    c04
    ..............................uguguacggcuacguauacaga............................................................   5       0    c04
    ..............................uguguacggcuaAguauacaga............................................................   1       1    c04
    .................................guacggcuacguauacag.............................................................   1       0    c04
    ......................................................................gacuguauaccuagcuaca.......................   1       0    c04
    ......................................................................gacuguauaccuagcuacaccu....................   8       0    c04
    ......................................................................Cacuguauaccuagcuacaccu....................   1       1    c04
    ........................................................................cuguauaccuagcuacaccu....................   1       0    c04

    ...........gcgcuuucuuguguauaag..................................................................................   2       0    c16
    ..............................uguguacggcuacguaua................................................................   1       0    c16
    ..............................uguguacggcuacguauac...............................................................   1       0    c16
    ..............................uguguacggcuacguauaca..............................................................   4       0    c16
    ..............................uguguacggcuacguauacag.............................................................   3       0    c16
    ..............................uguguacggcuacguauacagU............................................................   1       1    c16
    ..............................uguguacggcuacguauacaga............................................................   13      0    c16
    ..............................uguguacggcuacguauacagac...........................................................   1       0    c16
    ................................uguacggcuacguauacagac...........................................................   1       0    c16
    ................................uguacggcuacguauacagacau.........................................................   2       0    c16
    ..................................uacggcuacguauacagaca..........................................................   1       0    c16
    ..................................uacggcuacguauacagacau.........................................................   1       0    c16
    ..................................................................gaUugacuguauaccuagcuacaccu....................   1       1    c16
    .....................................................................ugacuguauaccuagcuacaccu....................   1       0    c16
    ......................................................................gacuguauaccuagcuacacc.....................   4       0    c16
    ......................................................................Cacuguauaccuagcuacaccu....................   1       1    c16
    ......................................................................gacuguauCccuagcuacaccu....................   1       1    c16
    ......................................................................gacAguauaccuagcuacaccu....................   1       1    c16
    ......................................................................gCcuguauaccuagcuacaccu....................   1       1    c16
```

Star **Mature**

```
ugaagcaagaagcgcuuucuuguguauaaguguguacggcuacguauacagacauguaagugucuagaaugacuguauaccuagcuacaccucguaugcaaggacacacacc
......................................................................gacuguauaccuagcuacacAu....................  1   1  c16
......................................................................gacuguauaccuagcuacaccu....................  40  0  c16
......................................................................gacuguauaccuagcuacaccuU...................  1   1  c16
.......................................................................acuguauaccuagcuacaccu....................  2   0  c16
.......................................................................Ccuguauaccuagcuacaccu....................  1   1  c16

..............................uguguacggcuacguaua................................................................  3   0  c09
..............................uguguacggcuacguauaca..............................................................  1   0  c09
..............................uguguacggcuacguauacaga............................................................  4   0  c09
................................uguacggcuacguauacagacau.........................................................  1   0  c09
..................................uacggcuacguauacagacaug........................................................  1   0  c09
....................................................................augacuguauaccuagcuacaccu....................  1   0  c09
.....................................................................ugacuguauaccuagcuacaccu....................  1   0  c09
......................................................................gacuguauaccuagcuacac......................  1   0  c09
......................................................................gacuguauaccuagcuacacc.....................  3   0  c09
......................................................................gacuguauaccuagcuacaccu....................  17  0  c09
......................................................................Cacuguauaccuagcuacaccu....................  1   1  c09
......................................................................gacuguauaccuagcuacaccuA...................  1   1  c09
.......................................................................acuguauaccuagcuacacc.....................  1   0  c09
.......................................................................acuguauaccuagcuacaccu....................  3   0  c09
..........................................................................guauaccuagcuacaccu....................  1   0  c09

..............................uguguacggcuacguauacaga............................................................  1   0  c15
..............................uguguacggcuacguauacagac...........................................................  1   0  c15
................................uguacggcuacguauacagaca..........................................................  1   0  c15
................................uguacggcuacguauacagacau.........................................................  5   0  c15
.................................guacggcuacguauacaga............................................................  1   0  c15
..................................uacggcuGcguauacagaca..........................................................  1   1  c15
..................................uacggcuacguauGcagaca..........................................................  1   1  c15
........................................uGcguauacagacauguaag....................................................  1   1  c15
................................................................uagaaugacuguauaccuag............................  2   0  c15
......................................................................gacuguauaccuagcuacac......................  1   0  c15
......................................................................gacuguauaccuagcuacacc.....................  6   0  c15
......................................................................gacuguauaccuagcuacaccC....................  3   1  c15
......................................................................gacuguauaccuagcuacaccA....................  7   1  c15
......................................................................gacuguauaccuagcuacaccu....................  63  0  c15
......................................................................gacuguauaccuagcuacaccuU...................  3   1  c15
.......................................................................acuguauaccuagcuacaccA....................  1   1  c15
.......................................................................acuguauaccuagcuacaccu....................  4   0  c15
........................................................................................accucguaugcaaggacacGc...  1   1  c15
..........................................................................................cucguaugcaaggacacacac.  1   0  c15

..............................uguCuacggcuacguauacaga............................................................  1   1  c02
..............................uguguacggcuacguauacagacau.........................................................  1   0  c02
................................uguacggcuacguauacagacau.........................................................  1   0  c02
..................................uacggcuacguauacagacau.........................................................  1   0  c02
.....................................................................ugacuguauaccuagcuacaccu....................  2   0  c02
......................................................................gacuguauaccuagcuacacc.....................  1   0  c02
......................................................................gacuguauaccuagcuacaccu....................  1   0  c02
.......................................................................Ccuguauaccuagcuacaccu....................  1   1  c02
.......................................................................acuguauaccuagcuacaccu....................  1   0  c02
```

Provisional ID	: group0_65
Score total	: 62
Score for star read(s)	: 3.9
Score for read counts	: 54.7
Score for mfe	: 2.4
Score for randfold	: 1.6
Score for cons. seed	: -0.6
Total read count	: 119
Mature read count	: 101
Loop read count	: 0
Star read count	: 18

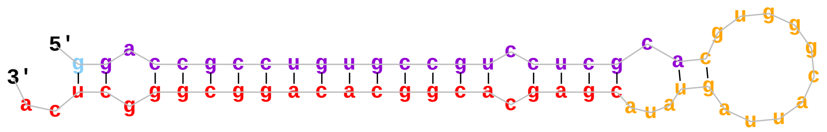

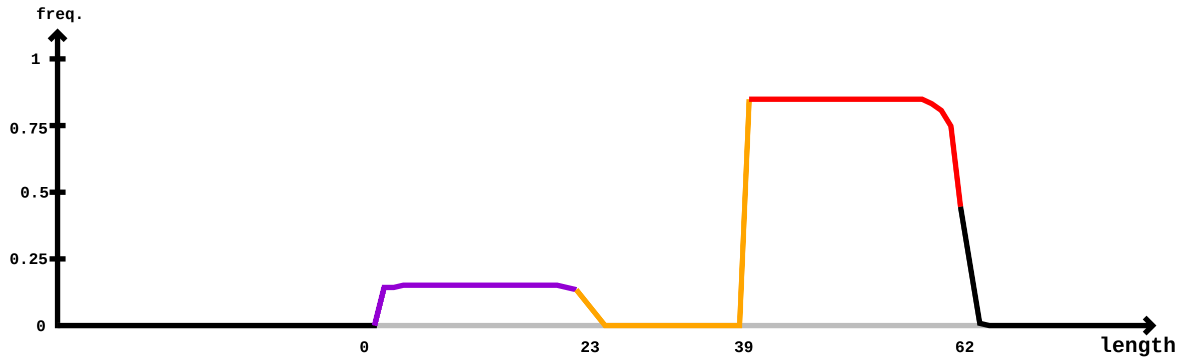

Star **Mature**

```
5'- aacuucaaggagaagaggggugguuggugugggaccgccugugccguccucgcacgugggcauuaguauacgagcacggcacaggcgggcucauacccuccacuauacaacg  -3'   obs
    aacuucaaggagaagaggggugguuggugugggaccgccugugccguccucgcacgugggcauuaguauacgagcacggcacaggcgggcucauacccuccacuauacaacg        exp
    ..((((........))))(((((..((((((((.((((((((((((((.((((.((..........))...)))).)))))))))))))).))))))))..)))))......      reads   mm   sample
    ................................gaccgccugugccguccucg............................................................      1       0    c02
    ................................gCccgccugugccguccucgc...........................................................      1       1    c02
    ................................gaccgccugugccguccucgca..........................................................      3       0    c02
    ................................gaccgccugugccguccucgcaA.........................................................      1       1    c02
    ......................................................................cgagcacggcacaggcggU.......................      1       1    c02
    ......................................................................cgagcacggcacaggcgggcu.....................      2       0    c02
    ......................................................................cgagcUcggcacaggcgggcuc....................      1       1    c02
    ......................................................................cgagcacggcacaggcgggcuA....................      1       1    c02
    ......................................................................cgagcacggcacCggcgggcuc....................      1       1    c02
    ......................................................................cgagcacggcacaggcgggcuc....................      3       0    c02
    ......................................................................cgagcacggcacaggcgggcuU....................      2       1    c02
    ......................................................................cgagcacggcacaggcgggcucU...................      9       1    c02
    ......................................................................cgagcacggcacaggcgggcucGu..................      1       1    c02
    ......................................................................cgagcacggcacaggcgggcucUu..................      4       1    c02

    ................................gaccgccugugccguccucgc...........................................................      1       0    c16
    ......................................................................cgagcacggcacaggcgggcuc....................      1       0    c16
    ......................................................................cgagcacggcacaggcgggcucU...................      2       1    c16
    ......................................................................cgagcacggcacaggcgggcucau..................      1       0    c16
    ......................................................................cgagcacggcacaggcgggcucUu..................      3       1    c16

    ................................gaccgccugugccguccuc.............................................................      1       0    c09
    ................................gaccgccugugccguccucgU...........................................................      1       1    c09
    ................................gaccgccugugccguccucgc...........................................................      1       0    c09
    ................................gaccgccugugccguccucgca..........................................................      7       0    c09
    ..................................ccgccugugccguccucgca..........................................................      1       0    c09
    ......................................................................cgagcacggcacaggcggg.......................      1       0    c09
    ......................................................................cgagcacggcacaggcgggc......................      3       0    c09
    ......................................................................cgagcaUggcacaggcgggcu.....................      1       1    c09
    ......................................................................cgagcacggcacaggcgggcu.....................      3       0    c09
    ......................................................................cCagcacggcacaggcgggcu.....................      1       1    c09
    ......................................................................cgagcacggcacaggcgggcuc....................      24      0    c09
    ......................................................................cgCgcacggcacaggcgggcuc....................      1       1    c09
```

Star Mature

```
aacuucaaggagaagaggggugguuggugugggaccgccugugccguccucgcacgugggcauuaguauacgagcacggcacaggcgggcucauacccuccacuauacaacg
......................................................................cgagcacggcacaggcgggcuU....................	1	1	c09
......................................................................cgagcacggcacaCgcgggcuc....................	1	1	c09
......................................................................cgagcacggcacaggcgggcucU...................	27	1	c09
......................................................................cgagcacggcacaggcgggcucUu..................	4	1	c09
......................................................................cgagcacggcacaggcgggcuUau..................	1	1	c09
......................................................................cgagcacggcacaggcgggcucaua.................	1	0	c09
```

Provisional ID	:	group0_712
Score total	:	1204.3
Score for star read(s)	:	3.9
Score for read counts	:	1198.2
Score for mfe	:	1.2
Score for randfold	:	1.6
Score for cons. seed	:	-0.6
Total read count	:	2362
Mature read count	:	2054
Loop read count	:	0
Star read count	:	308

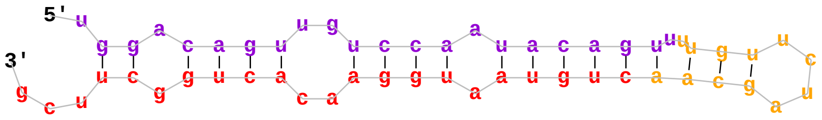

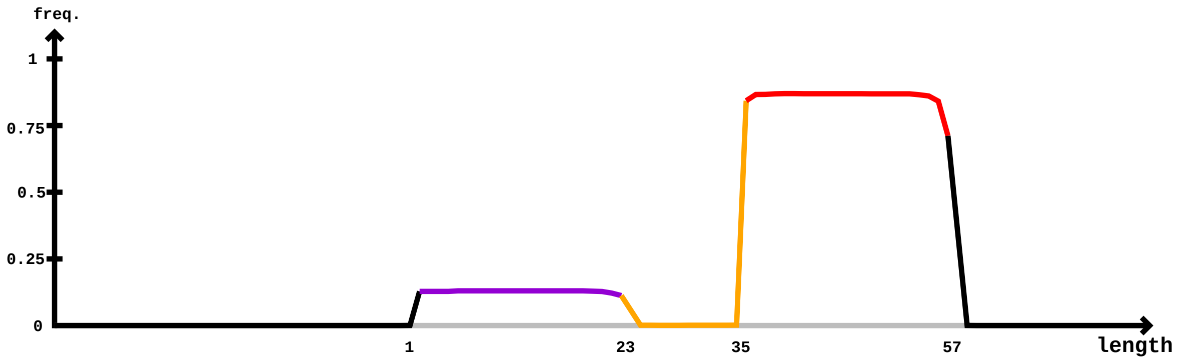

Star **Mature**

```
5'- uuuuugauagucaagauacaugcauaccgauucucguggacaguuguccaauacaguuuuguucuagcaacuguaauggaacacuggcuucgaguguugcguaggugagcau -3'   obs
    uuuuugauagucaagauacaugcauaccgauucucguggacaguuguccaauacaguuuuguucuagcaacuguaauggaacacuggcuucgaguguugcguaggugagcau        exp
    .((((((...))))))...((((.(((((((.((((.((.((((..((((.((((((..(((....))))))))).)))).)).)))).)).)))).))).....)))).))))   reads   mm   sample
    ....................................uggacaguuguccaauac..........................................................   1       0    c09
    ....................................uggacaguuguccaauaca.........................................................   2       0    c09
    ....................................uggacaguuguccaauacag........................................................   2       0    c09
    ....................................Gggacaguuguccaauacagu.......................................................   1       1    c09
    ....................................uggacaguuguccaauacagu.......................................................   2       0    c09
    ....................................uggacaguuguccaauacaguu......................................................   24      0    c09
    ....................................uggacaguugCccaauacaguu......................................................   1       1    c09
    ....................................uggCcaguuguccaauacaguu......................................................   1       1    c09
    ........................................caguuguccaauacaguu......................................................   1       0    c09
    ......................................................................cuguaauggaacacuggc........................   1       0    c09
    ......................................................................cuguaauggaacacuggcu.......................   1       0    c09
    ......................................................................cuguaauggaacacuggcuu......................   1       0    c09
    ......................................................................cuguaauggaacacuggcuuc.....................   6       0    c09
    ......................................................................cuguaauggaacacuggcuucg....................   21      0    c09
    ......................................................................cuguaauggaacacuCgcuucg....................   2       1    c09
    ......................................................................cuguaauggaacacuggcuucgU...................   4       1    c09
    .......................................................................uguaauggaacacuggcuucg....................   1       0    c09
    .......................................................................uguaauggaacacuggcuucgU...................   1       1    c09

    ....................................Gggacaguuguccaauacag........................................................   1       1    c04
    ....................................uggacaguuguccaauacag........................................................   2       0    c04
    ....................................uggacaguuguccaauacagG.......................................................   1       1    c04
    ....................................uggacaguuguccaauacagu.......................................................   1       0    c04
    ....................................uggacaguuguccaUuacaguu......................................................   1       1    c04
    ....................................uggacaguuguccaauacaguu......................................................   17      0    c04
    ........................................caguuguccaauacaguu......................................................   1       0    c04
    ........................................caguuguccaauacaguuA.....................................................   1       1    c04
    ..........................................................uuguucuagcaacuguaauggaaca.............................   1       0    c04
    ......................................................................cuguaauggaacacuggc........................   2       0    c04
    ......................................................................cuguaauggaacacuggcu.......................   1       0    c04
    ......................................................................cuguaauggaacacuggcuuc.....................   5       0    c04
    ......................................................................cuguaauggaaAacuggcuucg....................   1       1    c04
    ......................................................................cGguaauggaacacuggcuucg....................   1       1    c04
```

Star **Mature**

```
uuuuugauagucaagauacaugcauaccgauucucguggacaguuguccaauacaguuuuguucuagcaacuguaauggaacacuggcuucgaguguugcguaggugagcau
......................................................................cuguaauggaacacuggcuucg....................  46  0  c04
......................................................................cuguaauggaacacuggcuucU....................  1  1  c04
......................................................................cuAuaauggaacacuggcuucg....................  1  1  c04
......................................................................cuguaauggaacacuggcuucgU...................  6  1  c04
.......................................................................uguaauggaacacuggcuucg....................  4  0  c04
.........................................................................uaauggaacacuggcuucg....................  1  0  c04
.........................................................................uaauggaacacuggcuucgU...................  1  1  c04
..........................................................................aauggaacacuggcuucg....................  1  0  c04

...................................Uuggacaguuguccaauacaguu......................................................  1  1  c16
....................................uggacaguuguccaauac..........................................................  2  0  c16
....................................uggacaguuguccaauaca.........................................................  1  0  c16
....................................uggacaguuguccaauacaU........................................................  1  1  c16
....................................uggacaguuguccaauacag........................................................  8  0  c16
....................................uggacaguuguccaauacagu.......................................................  15  0  c16
....................................uggacaguugucUaauacaguu......................................................  1  1  c16
....................................Gggacaguuguccaauacaguu......................................................  3  1  c16
....................................uggCcaguuguccaauacaguu......................................................  2  1  c16
....................................uggacaguuguccaauacaguC......................................................  1  1  c16
....................................uggacaguuguUcaauacaguu......................................................  1  1  c16
....................................uggacaguuguccaauacaguu......................................................  124  0  c16
....................................uggacaguuguccaauacaguA......................................................  5  1  c16
....................................uggacaguuguccaauacaguuu.....................................................  7  0  c16
....................................uggacaguuguccaauacaguuA.....................................................  5  1  c16
........................................caAuuguccaauacaguu......................................................  2  1  c16
.........................................................uuuguucuagcaacuguaa....................................  1  0  c16
......................................................................cuguaauggaacacuggc........................  5  0  c16
......................................................................cuguaauggaacacuggcu.......................  8  0  c16
......................................................................cuguaauCgaacacuggcuu......................  1  1  c16
......................................................................cuguaauggaacacuggcuA......................  1  1  c16
......................................................................cuguaauggaacCcuggcuu......................  1  1  c16
......................................................................cuguaauggaacacAggcuu......................  1  1  c16
......................................................................cuguaauggaacacuggcuu......................  27  0  c16
......................................................................cuguaauCgaacacuggcuuc.....................  1  1  c16
......................................................................cuguaauggaacUcuggcuuc.....................  1  1  c16
......................................................................cuguaauggaacacuggcuuA.....................  2  1  c16
......................................................................cuguaauggaacacuggcuuU.....................  1  1  c16
......................................................................cuguaauggaacacuggcuuc.....................  152  0  c16
......................................................................cuguaauggaacUcuggcuucg....................  1  1  c16
......................................................................cuguaaugCaacacuggcuucg....................  1  1  c16
......................................................................cuguaauggaacacuCgcuucg....................  2  1  c16
......................................................................cuguaauggaacacuggcuucU....................  14  1  c16
......................................................................cuguaauggaacaAuggcuucg....................  1  1  c16
......................................................................cuguaaAggaacacuggcuucg....................  1  1  c16
......................................................................cuguaauggaacacuggcuuAg....................  1  1  c16
......................................................................cuguaCuggaacacuggcuucg....................  1  1  c16
......................................................................Auguaauggaacacuggcuucg....................  1  1  c16
......................................................................cuguaauggaacacAggcuucg....................  1  1  c16
......................................................................cuAuaauggaacacuggcuucg....................  1  1  c16
......................................................................cuCuaauggaacacuggcuucg....................  1  1  c16
......................................................................cuguaauggaacacuggcuucg....................  716  0  c16
......................................................................cuguaauggaaUacuggcuucg....................  1  1  c16
......................................................................cuguaauCgaacacuggcuucg....................  3  1  c16
......................................................................cuguaauggaCcacuggcuucg....................  9  1  c16
......................................................................Guguaauggaacacuggcuucg....................  1  1  c16
......................................................................cuguaauggaacacuggcuucC....................  1  1  c16
......................................................................cuguaauggaacCcuggcuucg....................  2  1  c16
......................................................................cuguaauggCacacuggcuucg....................  4  1  c16
......................................................................cuguaauggaacacuggAuucg....................  1  1  c16
......................................................................cuguaauggaacacuggcuucgU...................  82  1  c16
......................................................................cuguaauggaacacuggcuucgC...................  4  1  c16
......................................................................cuguaauggaacacuggcuucga...................  3  0  c16
......................................................................cuguaauggaacacuggcuucgG...................  1  1  c16
......................................................................cuguaauggaacacuggcuucUa...................  2  1  c16
.......................................................................Gguaauggaacacuggcuu......................  1  1  c16
.......................................................................uguaauggaacacuggcuu......................  1  0  c16
.......................................................................uguaauggaacacuggcuuc.....................  5  0  c16
.......................................................................uguaauggaacacuggcuucg....................  13  0  c16
.......................................................................Gguaauggaacacuggcuucg....................  1  1  c16
.......................................................................uguaauggaacacuggcuucgU...................  7  1  c16
```

Star Mature

```
uuuuugauagucaagauacaugcauaccgauucucguggacaguuguccaauacaguuuuguucuagcaacuguaauggaacacuggcuucgaguguugcguaggugagcau
.........................................................................uaauggaacacuggcuuc.....................    1    0    c16
.........................................................................uaauggaacacuggcuucg....................    2    0    c16
..........................................................................aauggaacacuggcuucg....................    1    0    c16

....................................uggacaguuguccaauacag........................................................    1    0    c02
....................................uggacaguuguccaauacagu.......................................................    2    0    c02
....................................uCgacaguuguccaauacaguu......................................................    1    1    c02
....................................uggacaguuguccaauacaguu......................................................    29   0    c02
....................................Gggacaguuguccaauacaguu......................................................    1    1    c02
....................................uggacaguuguccaauacaguuu.....................................................    1    0    c02
................................................................uagcaacuguaauggaacacuggcuucg....................    1    0    c02
......................................................................cuguaauggaacacuggcuu......................    1    0    c02
......................................................................cuguaauggaacacuggcuuc.....................    13   0    c02
......................................................................cuguaauggaacacuggcuuU.....................    1    1    c02
......................................................................cuguUauggaacacuggcuucg....................    1    1    c02
......................................................................cuguaauggaacacuggcuucg....................    62   0    c02
......................................................................cuguaauggCacacuggcuucg....................    1    1    c02
......................................................................cuguaauggaacacAggcuucg....................    1    1    c02
......................................................................cuguaauggaacacugUcuucg....................    1    1    c02
......................................................................cCguaauggaacacuggcuucg....................    1    1    c02
......................................................................cuguaauggaacacuggcuucU....................    1    1    c02
......................................................................cuguaauggaacacuggcuucgU...................    5    1    c02
.......................................................................ugGaauggaacacuggcuuc.....................    1    1    c02
.......................................................................uguaauggaacacuggcuucg....................    1    0    c02

....................................uggacaguuguccaauacaguu......................................................    28   0    c15
....................................uggacaguuguccaauacaguuC.....................................................    1    1    c15
....................................uggacaguuguccaauacaguuu.....................................................    4    0    c15
....................................uggacaguuguccaauacaguuuuU...................................................    1    1    c15
.....................................................................Ccuguaauggaacacuggcuucg....................    1    1    c15
......................................................................cuguaauggaacacuggcu.......................    1    0    c15
......................................................................cuguaauggaacacuggcuA......................    1    1    c15
......................................................................cuguaauggaacacuggcuu......................    10   0    c15
......................................................................cuguaauggaacacuggcuuc.....................    113  0    c15
......................................................................cuguaauggaacacuggcuuA.....................    1    1    c15
......................................................................cuguaauggaacacuggcuGc.....................    1    1    c15
......................................................................cuguaauggaacacuggcuuU.....................    1    1    c15
......................................................................cuguaauggaacacuggcuucg....................    458  0    c15
......................................................................cuguaaAggaacacuggcuucg....................    1    1    c15
......................................................................cuguaauggaacaUuggcuucg....................    1    1    c15
......................................................................cuguaauggaacacuggcuucU....................    13   1    c15
......................................................................cGguaauggaacacuggcuucg....................    1    1    c15
......................................................................cuguaauggaacacuggcuucA....................    49   1    c15
......................................................................Uuguaauggaacacuggcuucg....................    3    1    c15
......................................................................cugAaauggaacacuggcuucg....................    1    1    c15
......................................................................cuguaUuggaacacuggcuucg....................    1    1    c15
......................................................................cuguaauggaacacuggcuucgC...................    3    1    c15
......................................................................cuguaauggaacacuggcuucga...................    7    0    c15
......................................................................cuguaauggaacacuggcuucgU...................    78   1    c15
......................................................................cuguaauggaacacuggcuucgaC..................    1    1    c15
.......................................................................uguaauggaacacuggcuuc.....................    2    0    c15
.......................................................................uguaauggaacacuggcuucg....................    12   0    c15
.......................................................................uguaauggaacacuggcuucgU...................    5    1    c15
........................................................................guaauggaacacuggcuucgU...................    1    1    c15
```

Provisional ID	:	group0_728
Score total	:	175.2
Score for star read(s)	:	3.9
Score for read counts	:	168.4
Score for mfe	:	1.9
Score for randfold	:	1.6
Score for cons. seed	:	-0.6
Total read count	:	342
Mature read count	:	314
Loop read count	:	0
Star read count	:	28

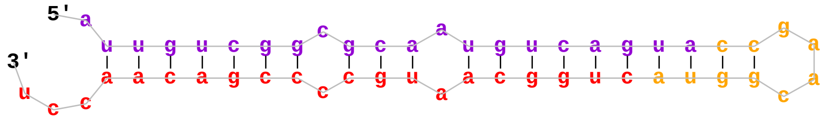

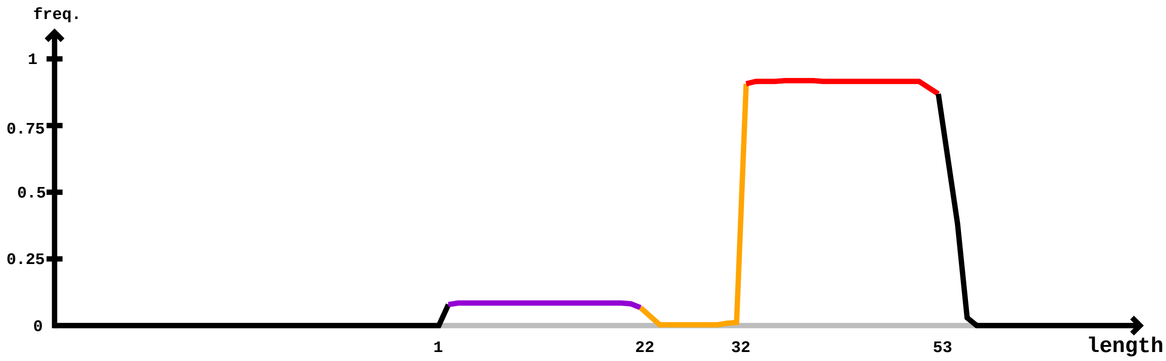


```
                                         Star                              Mature
5'- auauguagcuucgucgucgaaaaccgucugcuucgcggcauugucggcgcaaugucaguaccgaacgguacuggcaaugccccgacaaccuauaaccacccuggcuucaua  -3'   obs
    auauguagcuucgucgucgaaaaccgucugcuucgcggcauugucggcgcaaugucaguaccgaacgguacuggcaaugccccgacaaccuauaaccacccuggcuucaua        exp
    .((((.((((..((((.((((..........)))))))).(((((((.(((.((((((((((....)))))))))).))).)))))))..............)))).))))   reads    mm    sample
    .......................................auugucggcgcaaugucagu....................................................   1        0     c16
    .......................................auugucggcgcaaugucaguaA..................................................   2        1     c16
    .......................................auugucggcgcaaugucaguac..................................................   2        0     c16
    ....................................................................uacuggcaaugccccgacaacU.....................   1        1     c16
    .....................................................................acuggcaaugccccgacaaccu....................   1        0     c16
    ......................................................................cuggcaaugccccgacaac......................   1        0     c16
    ......................................................................cuggcaaugccccgacaacc.....................   3        0     c16
    ......................................................................cuggcaaugccccgacaCccu....................   1        1     c16
    ......................................................................cuggcaaugccccgacaaccu....................   28       0     c16
    ......................................................................cuggcaaugccccgacaaccua...................   4        0     c16
    ......................................................................cuggcaaugccccgacaaccuau..................   19       0     c16
    ......................................................................cuggcaaugccccgacaaccuUu..................   1        1     c16
    ......................................................................cuggcaaugccccgacaaccuauU.................   2        1     c16
    .......................................................................uggcaaugccccgacaaccu....................   1        0     c16

    ......................................................................cuggcaaugccccgacaaccuau..................   3        0     c04

    .......................................auugucggcgcaaugucag.....................................................   1        0     c09
    .......................................auugucggcgcaaugucagu....................................................   3        0     c09
    .......................................auugucggcgcaaugucagua...................................................   3        0     c09
    .......................................auugucggcgcaaugucaguac..................................................   1        0     c09
    ......................................................................cuggcaaugccccgacaac......................   1        0     c09
    ......................................................................cuggcaaugccccgacaacc.....................   1        0     c09
    ......................................................................cuggcaaugccccgacaaccu....................   41       0     c09
    ......................................................................cuggcaaugcccUgacaaccu....................   1        1     c09
    ......................................................................cuggcaaugccccgacaaccuU...................   3        1     c09
    ......................................................................cuggcaaugccccgacaaccua...................   11       0     c09
    ......................................................................cuggcaaugccccgacaaccuau..................   30       0     c09
    ......................................................................cuggcaaugccccgacaaccuUu..................   1        1     c09
    ......................................................................cuggcaaugccccgacaCccuau..................   1        1     c09
    ......................................................................cuggcaaugccccgacaaccuauG.................   1        1     c09
    .......................................................................uggcaaugccccgacaaccu....................   1        0     c09

    .......................................auugucggcgcaaugucagua...................................................   1        0     c15
```

```
                                       Star                         Mature
auauguagcuucgucgucgaaaaccgucugcuucgcggcauugucggcgcaaugucaguaccgaacgguacuggcaaugccccgacaaccuauaaccacccuggcuucaua
......................................................................cuggcaaugccccgacaaccua...................    2    0    c15
......................................................................cuggcaaugccccgacaaccuau..................    16   0    c15
......................................................................cuggcaaugccccgacaaccuauU.................    3    1    c15

.......................................auugucggcgcaaugucagu....................................................    1    0    c02
.......................................auugucggcgcaaugucagua...................................................    8    0    c02
.......................................auugucCgcgcaaugucaguac..................................................    1    1    c02
.......................................auugucggcgcaaugucaguaU..................................................    1    1    c02
.......................................auugucggcgcaaugucaguac..................................................    2    0    c02
........................................uugucggcgcaaugucagua...................................................    1    0    c02
........................................uugucggcgcaaugucaguaccgaacgguacuggcaau.................................    1    0    c02
....................................................................uacuggcaaugccccgacaacU.....................    1    1    c02
......................................................................cuggcaaugccccgacaac......................    5    0    c02
......................................................................cuggcaaugccccgCcaac......................    1    1    c02
......................................................................cuggcaaugccccgacaacc.....................    2    0    c02
......................................................................cuggcaaugccccgacaGccu....................    1    1    c02
......................................................................cuggcaaugccccgacaaAcu....................    1    1    c02
......................................................................cuggcaaugccccgacaaccu....................    60   0    c02
......................................................................cuggcaaugccccCacaaccu....................    1    1    c02
......................................................................cuggcaaugccccgCcaaccu....................    1    1    c02
......................................................................cuggcaaugccccgacaaccua...................    5    0    c02
......................................................................cuggcaaugccccgacaaccuU...................    2    1    c02
......................................................................cuggcaaugccccgaAaaccua...................    1    1    c02
......................................................................cuggcaaugccccCacaaccua...................    1    1    c02
......................................................................cuggcaaugccccgacaCccuau..................    1    1    c02
......................................................................cuggcaaugccccgacaaccuUu..................    3    1    c02
......................................................................cuggcaaugccccgacaaccuau..................    44   0    c02
......................................................................cuggcaaugccccgacaaccuauU.................    4    1    c02
.......................................................................uggcaaugccccgacaaccuau..................    1    0    c02
..........................................................................caaugccccgacaaccuau..................    1    0    c02
```

Provisional ID	:	group0_732
Score total	:	914.5
Score for star read(s)	:	3.9
Score for read counts	:	904.6
Score for mfe	:	1.4
Score for randfold	:	1.6
Score for cons. seed	:	3
Total read count	:	1786
Mature read count	:	1783
Loop read count	:	0
Star read count	:	3

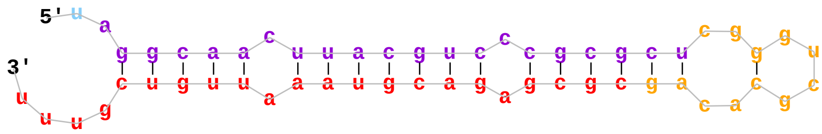

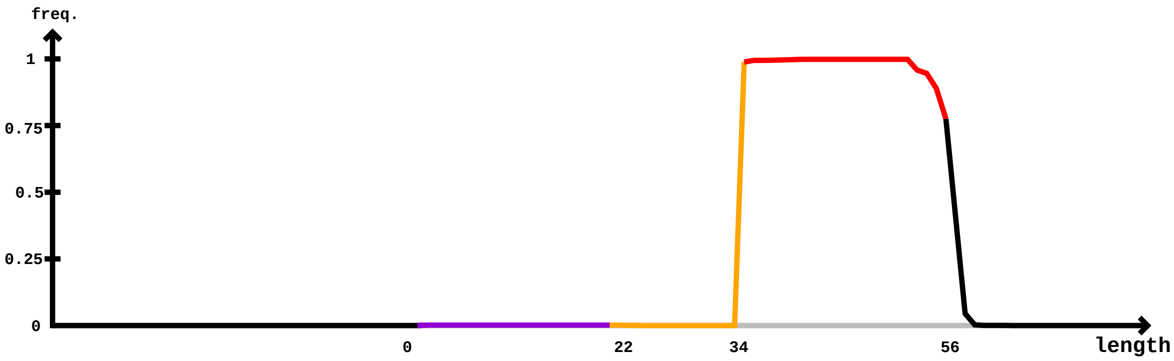

Star Mature

```
5'- uuccccuauaaaaggcgcgucgcuggcuccagccguuaggcaacuuacgucccgcgcucgggucgcacagcgcgagacguaaauugucguuucaaaccuuuggaaauaaaac  -3'  obs
    uuccccuauaaaaggcgcgucgcuggcuccagccguuaggcaacuuacgucccgcgcucgggucgcacagcgcgagacguaaauugucguuucaaaccuuuggaaauaaaac       exp
    ((((......(((((.(((..((((....)))))))..(((((.(((((((.((((((..(....)..)))))).))))))).)))))........))))))))).......  reads  mm  sample
    .....................................aggcaacuuacgucccgcgcu......................................................  1      0   c09
    .....................................aggcaacuuacgucccgcgcucA.....................................................  1      1   c09
    ......................................................................cgcgagacguaaaGuguc........................  1      1   c09
    ......................................................................cgcgagacguaaauuguU........................  2      1   c09
    ......................................................................cgcgagaGguaaauuguc........................  1      1   c09
    ......................................................................cgcgagacguaaauuguc........................  41     0   c09
    ......................................................................cgcgagacguaaauugucg.......................  8      0   c09
    ......................................................................cgcgagaAguaaauugucg.......................  1      1   c09
    ......................................................................cgcgagacguaaauugucgu......................  49     0   c09
    ......................................................................cgcgagacguaaauuguAgu......................  1      1   c09
    ......................................................................cgcgagacguaaauugucgA......................  1      1   c09
    ......................................................................cgcgagacguaaauugucguu.....................  74     0   c09
    ......................................................................cgcgagacguaaauugucgAu.....................  1      1   c09
    ......................................................................cgcgagacguaaauugucguA.....................  2      1   c09
    ......................................................................cAcgagacguaaauugucguuu....................  1      1   c09
    ......................................................................cgcgagacguaaauugucguuu....................  247    0   c09
    ......................................................................cgcgagacguaaauuCucguuu....................  1      1   c09
    ......................................................................cgcgagacguaaauuguUguuu....................  2      1   c09
    ......................................................................cgcgagacguaUauugucguuu....................  1      1   c09
    ......................................................................cgcgagCcguaaauugucguuu....................  2      1   c09
    ......................................................................Ugcgagacguaaauugucguuu....................  1      1   c09
    ......................................................................cgcgagacguaaaCugucguuu....................  1      1   c09
    ......................................................................cgcgagacguaaauugucguuA....................  3      1   c09
    ......................................................................cgcgagacguaaaAugucguuu....................  1      1   c09
    ......................................................................cgcgagacguaaauugucguuC....................  1      1   c09
    ......................................................................cCcgagacguaaauugucguuu....................  1      1   c09
    ......................................................................cgcgagacguaaauuguAguuu....................  1      1   c09
    ......................................................................cgcgagacguaaauugucguuuU...................  29     1   c09
    ......................................................................cgcgagacguaaauugucguuuA...................  10     1   c09
    ......................................................................cgcgagacguaaauugucguuuG...................  1      1   c09
    ......................................................................cgcgagacgGaaauugucguuuc...................  1      1   c09
    ......................................................................cgcgagacguaaauugucguuuc...................  30     0   c09
    ......................................................................cgcgagacguaaauugucguuucU..................  6      1   c09
```

Star **Mature**

```
uuccccuauaaaaggcgcgucgcuggcuccagccguuaggcaacuuacgucccgcgcucgggucgcacagcgcgagacguaaauugucguuucaaaccuuuggaaauaaaac
......................................................................cgcgagacguaaauugucguuucC..................   1   1   c09
......................................................................cgcgagacguaaauugucguuuUa..................   1   1   c09
......................................................................cgcgagacguaaauugucguuuca..................   4   0   c09
.......................................................................gcgagacguaaauugucguuu....................   1   0   c09
.........................................................................gagacguaaauugucguuu....................   1   0   c09
..........................................................................agacguaaauugucguuuA...................   1   1   c09
...........................................................................gaAguaaauugucguuuca..................   1   1   c09
...........................................................................gacguaaauugucguuucU..................   1   1   c09

......................................................................cgcgagacguaaauugucguuu....................   2   0   c04
......................................................................cgcgagacguaaauugucguuuA...................   1   1   c04

..........................................................Cggcaacuuacgucccgcgcu.................................   1   1   c16
......................................................................cgcgagacguaaauuguc........................   9   0   c16
......................................................................cgcgagacguaaauugucg.......................   1   0   c16
......................................................................cgcgagacguaaauuCucg.......................   1   1   c16
......................................................................cgcgagacguaaauugucgu......................   15  0   c16
......................................................................cgcgagacguaaauugucguu.....................   23  0   c16
......................................................................cgcgagacguaaauugucguuA....................   2   1   c16
......................................................................cgcgagacguaaauugucguAu....................   1   1   c16
......................................................................cgcgagCcguaaauugucguuu....................   2   1   c16
......................................................................cgcgagacguaaauugucguuu....................   77  0   c16
......................................................................cgUgagacguaaauugucguuu....................   1   1   c16
......................................................................cgcgCgacguaaauugucguuu....................   1   1   c16
......................................................................cgcgagacguaaauugucUuuu....................   1   1   c16
......................................................................cgcgagacguaaauugucguuuc...................   8   0   c16
......................................................................cgcgagacguaaauugucgAuuc...................   1   1   c16
......................................................................cgcgagacguaaauugucguuuA...................   15  1   c16
......................................................................cgcgagacguaaauugucguuucU..................   2   1   c16
......................................................................cgcgagacguaaauugucguuuca..................   6   0   c16
......................................................................cgcgagacguaaauugucguuucaa.................   1   0   c16
......................................................................cgcgagacguaaauugucguuucaaacc..............   1   0   c16
.......................................................................gcgagacguaaauugucguuuAa..................   1   1   c16

......................................................................cgcgCgacguaaauuguc........................   1   1   c02
......................................................................cgcgagacguaaauuguc........................   14  0   c02
......................................................................cgcgagCcguaaauugucg.......................   1   1   c02
......................................................................cgcgagacguaaauugucg.......................   10  0   c02
......................................................................cgcgagacguaaauuguUgu......................   1   1   c02
......................................................................cgcgagCcguaaauugucgu......................   1   1   c02
......................................................................cgcgagacguaaauugucgu......................   32  0   c02
......................................................................cgcgagaAguaaauugucguu.....................   1   1   c02
......................................................................cgcgagacguaaauugucguA.....................   3   1   c02
......................................................................cUcgagacguaaauugucguu.....................   3   1   c02
......................................................................cgcgagacguaaauugucguu.....................   80  0   c02
......................................................................Ugcgagacguaaauugucguu.....................   1   1   c02
......................................................................cgcgagacguaaCuugucguu.....................   1   1   c02
......................................................................cgGgagacguaaauugucguu.....................   1   1   c02
......................................................................cgcgagacguaaauugucgAuu....................   1   1   c02
......................................................................cgcgagacgCaaauugucguuu....................   1   1   c02
......................................................................cgcgagacguaaauugucguGu....................   1   1   c02
......................................................................cgcgagacguaaauuAucguuu....................   1   1   c02
......................................................................cgcgagCcguaaauugucguuu....................   3   1   c02
......................................................................cgcgagacguaaauugucguuA....................   3   1   c02
......................................................................cgUgagacguaaauugucguuu....................   1   1   c02
......................................................................cgcgagacguaaauuCucguuu....................   2   1   c02
......................................................................cgcgagacgGaaauugucguuu....................   1   1   c02
......................................................................cUcgagacguaaauugucguuu....................   1   1   c02
......................................................................cgcgaUacguaaauugucguuu....................   1   1   c02
......................................................................cgcgagacguaaauugucgGuu....................   1   1   c02
......................................................................cgcgagacguaaauugucguuu....................   322 0   c02
......................................................................cgcgagacguaaauugucguuuA...................   19  1   c02
......................................................................cgcgagacguaaauugucguuuc...................   47  0   c02
......................................................................cgcgagacguaaauugucguuuU...................   28  1   c02
......................................................................cgcgagacguaaauugucguuuG...................   1   1   c02
......................................................................cgcgagacguaaauugucguuuca..................   10  0   c02
......................................................................cgcgagacguaaauugucguuucG..................   1   1   c02
......................................................................cgcgagacguaaauugucguuuAa..................   1   1   c02
......................................................................cgcgagacguaaauugucguuucU..................   15  1   c02
.......................................................................gcgagacguaaauugucguu.....................   1   0   c02
```

Star Mature

```
uuccccuauaaaaggcgcgucgcuggcuccagccguuaggcaacuuacgucccgcgcucgggucgcacagcgcgagacguaaauugucguuucaaaccuuuggaaauaaaac
.......................................................................gcgagacguaaauugucguuu....................    3    0    c02
.......................................................................gcgagacguaaauugucguuuc...................    2    0    c02
.......................................................................gcgagacguaaauugucguuuA...................    1    1    c02
..........................................................................agacguaaauugucguuu....................    1    0    c02
............................................................................acguaaauugucguuucU..................    1    1    c02
............................................................................acguaaauugucguuuca..................    1    0    c02

......................................................................cgcgagacguaaauuguc........................    3    0    c15
......................................................................cgcgagacguaaauugucgu......................    1    0    c15
......................................................................cgcgagacguaaauugucguA.....................    2    1    c15
......................................................................cgcgagacguaaauugucguu.....................    11   0    c15
......................................................................cAcgagacguaaauugucguuu....................    1    1    c15
......................................................................cgcgagacguaaauugucguuA....................    2    1    c15
......................................................................cUcgagacguaaauugucguuu....................    1    1    c15
......................................................................Ugcgagacguaaauugucguuu....................    1    1    c15
......................................................................cgcgagacguaaauugucguuu....................    264  0    c15
......................................................................cgcgagacguaaauugucguuuU...................    29   1    c15
......................................................................cgcgagacguaaCuugucguuuc...................    1    1    c15
......................................................................cgcgagacguaaauugucguuuc...................    103  0    c15
......................................................................cgcgagacguaaauugucguuuA...................    11   1    c15
......................................................................cgcgagacguaaauugucguuuca..................    20   0    c15
......................................................................cgcgagacguaaauugucguuucU..................    3    1    c15
......................................................................cgcgagacguaaauugucguuucaG.................    1    1    c15
......................................................................cgcgagacguaaauugucguuucaa.................    2    0    c15
.......................................................................gcgagacguaaauugucguuu....................    1    0    c15
.......................................................................gcgagacguaaauugucguuuc...................    1    0    c15
```

Provisional ID : group10_14595
Score total : 449083.7
Score for star read(s) : 3.9
Score for read counts : 449074.5
Score for mfe : 0.7
Score for randfold : 1.6
Score for cons. seed : 3
Total read count : 880852
Mature read count : 876820
Loop read count : 0
Star read count : 4032

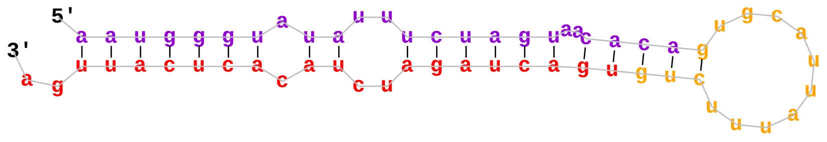

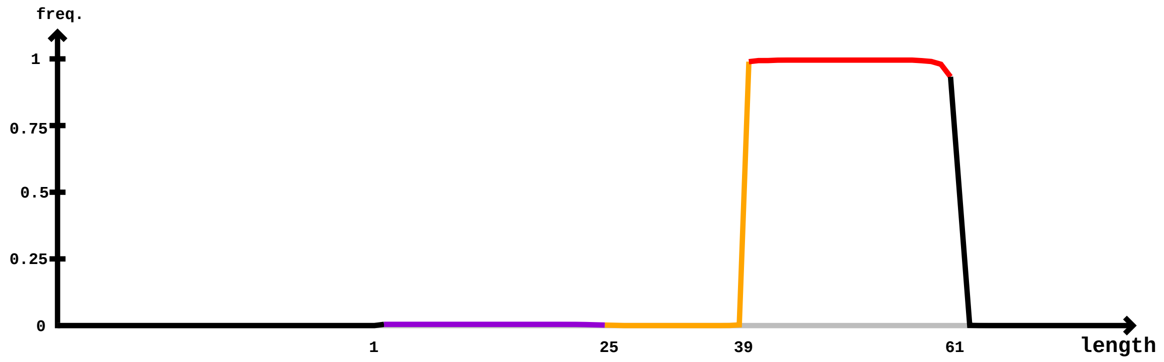

Star Mature

```
5'- aagaaagaaguugaaaguaccuacuuguucuuaauggguauauuucuaguaacacagugcauuauuucugugacuagaucuacacucauugaguacguuuaggacaacau  -3'   obs
    aagaaagaaguugaaaguaccuacuuguucuuaauggguauauuucuaguaacacagugcauuauuucugugacuagaucuacacucauugaguacguuuaggacaacau         exp
    ................((.((((..(((.((((((((((.((..((((((..(((((.........)))))))))))..)).))))))))))).)))..)))))).......   reads   mm   sample
    ................................aauggguauauuuAuagu............................................................   1   1   c09
    ................................aauggguauauuucuagu............................................................   2   0   c09
    ................................aauggguauauuucuagua...........................................................   5   0   c09
    ................................aGuggguauauuucuagua...........................................................   1   1   c09
    ................................aauggguauauuucuaguaac.........................................................   8   0   c09
    ................................aauggguauauuucuaguaaca........................................................   27   0   c09
    ................................aauggguauauuucuaguaacac.......................................................   28   0   c09
    ................................aauggguauauuucuaguaacaca......................................................   127   0   c09
    ................................aaugCguauauuucuaguaacaca......................................................   1   1   c09
    ................................Cauggguauauuucuaguaacaca......................................................   3   1   c09
    ................................aauggguauauuucuaguaCcaca......................................................   1   1   c09
    ................................aauggguauauuucuaguaacacC......................................................   1   1   c09
    ................................aaugggGauauuucuaguaacaca......................................................   1   1   c09
    ................................aauggguauauuucuaguaacacaA.....................................................   3   1   c09
    ................................aauggguauauuucuaguaacacaU.....................................................   1   1   c09
    ..................................uggguauauuucuaguaacac.......................................................   1   0   c09
    ..................................uggguauauuucuaguaacaca......................................................   3   0   c09
    ..................................uggguauauuucuaguaacacag.....................................................   1   0   c09
    ..................................................................Gcugugacuagaucuacacucauuga..................   1   1   c09
    ...................................................................cGgugacuagaucuacacucauuga..................   1   1   c09
    ...................................................................Augugacuagaucuacacucauuga..................   1   1   c09
    ...................................................................cuCugacuagaucuacacucauuga..................   1   1   c09
    ...................................................................cuUugacuagaucuacacucauuga..................   1   1   c09
    ....................................................................Ggugacuagaucuacacuc.......................   1   1   c09
    ....................................................................Ggugacuagaucuacacuca......................   1   1   c09
    ....................................................................ugugacuagaucuacacucau.....................   1   0   c09
    ....................................................................Ggugacuagaucuacacucauu....................   5   1   c09
    ....................................................................uCugacuagaucuacacucauu....................   1   1   c09
    ....................................................................ugugacuagaucuacacucauu....................   6   0   c09
    ....................................................................uCugacuagaucuacacucauug...................   1   1   c09
    ....................................................................ugugacuagaucuacacucauug...................   1   0   c09
    ....................................................................Ggugacuagaucuacacucauuga..................   4   1   c09
    ....................................................................uCugacuagaucuacacucauuga..................   2   1   c09
```

Star **Mature**

aagaaagaaguugaaaguaccuacuuguucuuaauggguauauuucuaguaacacagugcauuauuucugugacuagaucuacacucauugaguacguuuaggacaacau			
....ugugacuagaucuacacucauuga..................	1	0	c09
....Agugacuagaucuacacucauuga..................	2	1	c09
....Cugacuagaucuacacuc.......................	1	1	c09
....Cugacuagaucuacacucauu....................	2	1	c09
....Cugacuagaucuacacucauug...................	6	1	c09
....gugacuagaucuacacucauug...................	1	0	c09
....Cugacuagaucuacacucauuga..................	58	1	c09
....Uugacuagaucuacacucauuga..................	2	1	c09
....gugacuagaucuacacucauuga..................	3	0	c09
....ugacuagGucuacacuca......................	1	1	c09
....ugacuagaucuacacuAa......................	1	1	c09
....ugacuagaucuacacuca......................	142	0	c09
....ugacuagaucuacacucC......................	3	1	c09
....ugacuagCucuacacuca......................	1	1	c09
....ugacuagaucuacacucG......................	1	1	c09
....ugacuagaucGacacuca......................	1	1	c09
....ugacuagaucuacacucU......................	2	1	c09
....Ggacuagaucuacacuca......................	82	1	c09
....ugacCagaucuacacuca......................	1	1	c09
....ugacuagauUuacacuca......................	1	1	c09
....ugacuagaucAacacuca......................	1	1	c09
....ugacuagauGuacacuca......................	1	1	c09
....Agacuagaucuacacuca......................	1	1	c09
....ugacuagaucuacCcucau.....................	1	1	c09
....ugacuagaucuacacucGu.....................	1	1	c09
....ugacuagaucuacacucaG.....................	2	1	c09
....ugacuagaucuacacucau.....................	94	0	c09
....Agacuagaucuacacucau.....................	1	1	c09
....ugacuagCucuacacucau.....................	1	1	c09
....Ggacuagaucuacacucau.....................	40	1	c09
....ugCcuagaucuacacucau.....................	1	1	c09
....ugacuagaucuacacucaA.....................	1	1	c09
....uCacuagaucuacacucauu....................	1	1	c09
....ugacuagaucuaUacucauu....................	1	1	c09
....ugacuagaucuacacucauu....................	643	0	c09
....ugacuagaucuacCcucauu....................	2	1	c09
....ugacuagaucuCcacucauu....................	1	1	c09
....ugacuagUucuacacucauu....................	1	1	c09
....ugacuagaucuacacucauC....................	3	1	c09
....ugacuagauAuacacucauu....................	1	1	c09
....Ggacuagaucuacacucauu....................	341	1	c09
....Cgacuagaucuacacucauu....................	1	1	c09
....ugacuagGucuacacucauu....................	2	1	c09
....ugacGagaucuacacucauu....................	1	1	c09
....ugacuagaucuacacGcauu....................	1	1	c09
....Agacuagaucuacacucauu....................	13	1	c09
....ugacuagaucuacacucaAu....................	1	1	c09
....ugacAagaucuacacucauu....................	1	1	c09
....ugCcuagaucuacacucauu....................	5	1	c09
....ugaUuagaucuacacucauug...................	1	1	c09
....ugacuagaucuCcacucauug...................	3	1	c09
....ugacGagaucuacacucauug...................	1	1	c09
....ugacuagaAcuacacucauug...................	1	1	c09
....ugacuagUucuacacucauug...................	1	1	c09
....Agacuagaucuacacucauug...................	31	1	c09
....ugacuagaucuacacucauuU...................	3	1	c09
....ugacuagaCcuacacucauug...................	1	1	c09
....ugacuagaucuacacucaCug...................	1	1	c09
....ugacuagaucuacaUucauug...................	3	1	c09
....ugacuagaucuacacucaGug...................	1	1	c09
....ugacuagaucuaUacucauug...................	2	1	c09
....ugacuagaucuacacucauug...................	1621	0	c09
....ugacuagauUuacacucauug...................	1	1	c09
....ugacuagaucCacacucauug...................	1	1	c09
....Ggacuagaucuacacucauug...................	794	1	c09
....ugaGuagaucuacacucauug...................	1	1	c09
....ugacuagGucuacacucauug...................	1	1	c09
....ugacuagaucuacacucauuC...................	7	1	c09
....ugacuagaucuacacuAauug...................	5	1	c09
....ugacuagaucuacacucauuA...................	4	1	c09

Star Mature

```
aagaaagaaguugaaaguaccuacuuguucuuaauggguauauuucuaguaacacagugcauuauuucugugacuagaucuacacucauugaguacguuuaggacaacau
......................................................................ugacuagaucuaAacucauug...................	1	1	c09
......................................................................ugacuaUaucuacacucauug...................	3	1	c09
......................................................................ugUcuagaucuacacucauug...................	1	1	c09
......................................................................ugacuGgaucuacacucauug...................	1	1	c09
......................................................................ugacuagaucuacUcucauug...................	2	1	c09
......................................................................ugCcuagaucuacacucauug...................	4	1	c09
......................................................................ugacuagaucAacacucauug...................	1	1	c09
......................................................................ugacuagaucuacacucaAug...................	2	1	c09
......................................................................uUacuagaucuacacucauug...................	1	1	c09
......................................................................ugacuagaucuacCcucauug...................	6	1	c09
......................................................................ugacuagCucuacacucauug...................	4	1	c09
......................................................................ugacAagaucuacacucauuga..................	3	1	c09
......................................................................uUacuagaucuacacucauuga..................	4	1	c09
......................................................................ugacuagCucuacacucauuga..................	23	1	c09
......................................................................ugaUuagaucuacacucauuga..................	5	1	c09
......................................................................ugacuagaGcuacacucauuga..................	9	1	c09
......................................................................ugacuagaucuaGacucauuga..................	2	1	c09
......................................................................ugacuagaucuacacucauuUa..................	5	1	c09
......................................................................ugacuagaucuacacucauugC..................	9	1	c09
......................................................................ugacuGgaucuacacucauuga..................	4	1	c09
......................................................................Ggacuagaucuacacucauuga..................	6105	1	c09
......................................................................ugacuagaucAacacucauuga..................	3	1	c09
......................................................................ugacuagaucuacGcucauuga..................	4	1	c09
......................................................................ugacuagaucuacacucauuAa..................	1	1	c09
......................................................................ugacCagaucuacacucauuga..................	2	1	c09
......................................................................ugUcuagaucuacacucauuga..................	3	1	c09
......................................................................Agacuagaucuacacucauuga..................	229	1	c09
......................................................................ugacuagaucuacacucCuuga..................	1	1	c09
......................................................................ugacuaCaucuacacucauuga..................	6	1	c09
......................................................................ugacuagaucuacacucaGuga..................	6	1	c09
......................................................................ugacuagaucuacacuAauuga..................	11	1	c09
......................................................................ugacuagaucuaAacucauuga..................	9	1	c09
......................................................................ugacuagaucuacacucauuCa..................	12	1	c09
......................................................................ugacuagaucuCcacucauuga..................	8	1	c09
......................................................................ugaAuagaucuacacucauuga..................	8	1	c09
......................................................................ugacuagaucuacaAucauuga..................	7	1	c09
......................................................................ugacuagaucuGcacucauuga..................	6	1	c09
......................................................................ugacuagaucuacacGcauuga..................	3	1	c09
......................................................................uAacuagaucuacacucauuga..................	6	1	c09
......................................................................ugacuagaucuacacucauugU..................	11	1	c09
......................................................................ugacuagaucuacacucaAuga..................	10	1	c09
......................................................................ugacuagaucuacacucauCga..................	5	1	c09
......................................................................ugacuagaucuUcacucauuga..................	7	1	c09
......................................................................ugacuagaucuacacucUuuga..................	6	1	c09
......................................................................ugacuagaucuacacucauugG..................	18	1	c09
......................................................................ugacuagauAuacacucauuga..................	8	1	c09
......................................................................ugacuagaucuacacucauuga..................	13624	0	c09
......................................................................ugacuagaucuacacCcauuga..................	2	1	c09
......................................................................ugacuagaucuacaUucauuga..................	8	1	c09
......................................................................ugacuagaucuacCcucauuga..................	44	1	c09
......................................................................ugCcuagaucuacacucauuga..................	35	1	c09
......................................................................ugacuaUaucuacacucauuga..................	2	1	c09
......................................................................ugaGuagaucuacacucauuga..................	6	1	c09
......................................................................ugacuagaucuaUacucauuga..................	14	1	c09
......................................................................ugacuagaucuacUcucauuga..................	7	1	c09
......................................................................ugacuagaucCacacucauuga..................	13	1	c09
......................................................................ugacuUgaucuacacucauuga..................	1	1	c09
......................................................................ugacuagGucuacacucauuga..................	5	1	c09
......................................................................ugacuagaucuacacuGauuga..................	2	1	c09
......................................................................ugacuagaucuacacucGuuga..................	3	1	c09
......................................................................ugacuagaucuacacucauAga..................	3	1	c09
......................................................................ugacuagaAcuacacucauuga..................	2	1	c09
......................................................................ugacuagaucuacacuUauuga..................	4	1	c09
......................................................................ugacuagaucuacacAcauuga..................	3	1	c09
......................................................................ugacuagaucGacacucauuga..................	1	1	c09
......................................................................ugacuagUucuacacucauuga..................	4	1	c09
......................................................................uCacuagaucuacacucauuga..................	1	1	c09
......................................................................ugacuagauUuacacucauuga..................	9	1	c09
......................................................................ugacuagaucuacacucaCuga..................	1	1	c09
......................................................................ugacuagaCcuacacucauuga..................	2	1	c09
```

Star **Mature**

```
aagaaagaaguugaaaguaccuacuuguucuuaauggguauauuucuaguaacacagugcauuauuucugugacuagaucuacacucauugaguacguuuaggacaacau
......................................................................ugacuagaucuacacucauGga..................  2    1  c09
......................................................................Cgacuagaucuacacucauuga..................  19   1  c09
......................................................................ugGcuagaucuacacucauuga..................  2    1  c09
......................................................................ugacuagaucuacaUucauugag.................  1    1  c09
......................................................................Agacuagaucuacacucauugag.................  1    1  c09
......................................................................ugacuagaucCacacucauugag.................  1    1  c09
......................................................................ugacuagaucuacacucauugaA.................  484  1  c09
......................................................................ugacuagaucuacacucauugaU.................  49   1  c09
......................................................................ugacuagaucuacacucauugaC.................  4    1  c09
......................................................................Ggacuagaucuacacucauugag.................  27   1  c09
......................................................................ugacuagaucuGcacucauugag.................  1    1  c09
......................................................................ugacuagCucuacacucauugag.................  1    1  c09
......................................................................ugacuagaucuacacucauGgag.................  1    1  c09
......................................................................ugacuagaucuacacucauugGg.................  1    1  c09
......................................................................ugacuagaucuacacucauugag.................  46   0  c09
......................................................................ugacuagaucuacacucauugagu................  11   0  c09
......................................................................ugacuagaucuacacucauugagC................  1    1  c09
......................................................................ugacuagaucuacacucauugagG................  3    1  c09
......................................................................ugacuagaucuacacucauugaAu................  6    1  c09
......................................................................ugacuagaucuacacucauugaCu................  1    1  c09
......................................................................ugacuagaucuacacucauugagA................  5    1  c09
......................................................................Ggacuagaucuacacucauugagu................  6    1  c09
......................................................................ugacuagaucuacacucauugaguC...............  1    1  c09
......................................................................ugacuagaucuacacucauugagAa...............  1    1  c09
.......................................................................gacuagaucuacacucau.....................  1    0  c09
.......................................................................gGcuagaucuacacucauu....................  1    1  c09
.......................................................................gacuagaucuacacucauu....................  4    0  c09
.......................................................................gacuagaucuacacucauug...................  4    0  c09
.......................................................................gacuagaucuacacucauuga..................  30   0  c09
.......................................................................gacuagaucuacacAcauuga..................  1    1  c09
.......................................................................gaAuagaucuacacucauuga..................  1    1  c09
.......................................................................gGcuagaucuacacucauuga..................  1    1  c09
.......................................................................gacuagaucuacacGcauuga..................  1    1  c09
........................................................................aAuagaucuacacucauug...................  1    1  c09
........................................................................Gcuagaucuacacucauuga..................  2    1  c09
........................................................................acuagaucuacacucauuga..................  12   0  c09
........................................................................aAuagaucuacacucauuga..................  1    1  c09
........................................................................Ccuagaucuacacucauuga..................  1    1  c09
.........................................................................cuagaucuacacucauug...................  33   0  c09
.........................................................................cuagaucuacacucauuU...................  1    1  c09
.........................................................................cuagCucuacacucauuga..................  7    1  c09
.........................................................................cuaCaucuacacucauuga..................  1    1  c09
.........................................................................cuagaucuCcacucauuga..................  1    1  c09
.........................................................................cuagaucuacacucauuga..................  221  0  c09
.........................................................................Auagaucuacacucauuga..................  1    1  c09
.........................................................................cuagaucuacaAucauuga..................  1    1  c09
.........................................................................cuagaucuacCcucauuga..................  1    1  c09
.........................................................................cuagaAcuacacucauuga..................  1    1  c09
.........................................................................cuagaucuacacGcauuga..................  1    1  c09
.........................................................................cuagaucuacacucaGuga..................  1    1  c09
.........................................................................cuagaucuacacucauugag.................  2    0  c09
.........................................................................cuagaucuacacucauugaA.................  7    1  c09
..........................................................................Gagaucuacacucauuga..................  6    1  c09
..........................................................................uagaucuacacucauugG..................  1    1  c09
..........................................................................uagaucuacacucauuga..................  18   0  c09
..........................................................................Aagaucuacacucauuga..................  1    1  c09

................................aauggguauauuucuagu............................................................  5    0  c16
................................aauggguauauuucuagua...........................................................  23   0  c16
................................aauggguauauuucuaguaa..........................................................  3    0  c16
................................Cauggguauauuucuaguaa..........................................................  1    1  c16
................................aauggguauauuucuaguaac.........................................................  31   0  c16
................................Cauggguauauuucuaguaac.........................................................  1    1  c16
................................Cauggguauauuucuaguaaca........................................................  2    1  c16
................................aauggguauauuucuaguaaca........................................................  65   0  c16
................................aauggguauauuucuaguaacU........................................................  1    1  c16
................................aauggguauauuucuaguaacaU.......................................................  1    1  c16
................................aauggguauauuucuaguaacac.......................................................  73   0  c16
................................aauggguauauuucuaguaacaA.......................................................  4    1  c16
................................Cauggguauauuucuaguaacac.......................................................  5    1  c16
```

Star **Mature**

```
aagaaagaaguugaaaguaccuacuuguucuuaauggguauauuucuaguaacacagugcauuauuucugugacuagaucuacacucauugaguacguuuaggacaacau
................................aauggguauauuucuaguaacacG......................................................    1     1    c16
................................aUuggguauauuucuaguaacaca......................................................    1     1    c16
................................Cauggguauauuucuaguaacaca......................................................    7     1    c16
................................aauggguauauuucuaguaacaca......................................................    262   0    c16
................................aauggguauauuucuaguaacacU......................................................    1     1    c16
................................aauggguauauuucuaguaacaUa......................................................    1     1    c16
................................aauggguauauuuAuaguaacaca......................................................    1     1    c16
................................aaAggguauauuucuaguaacaca......................................................    1     1    c16
................................aauggguauauuucuaguaacacaA.....................................................    7     1    c16
................................aauggguauauuucuaguaacacaU.....................................................    1     1    c16
................................aauggguauauAucuaguaacacag.....................................................    1     1    c16
................................aauggguauauuucuaguaacacag.....................................................    1     0    c16
................................aauggguauauuucuaguaacacaAu....................................................    1     1    c16
.................................auggguauauuucuaguaacaca......................................................    1     0    c16
..................................uggguauauuucuaguaacac.......................................................    1     0    c16
..................................uggguauauuucuaguaacaca......................................................    1     0    c16
...................................ggguauauuucuaguaacac.......................................................    1     0    c16
........................................................gugcauuauuucugugacuagaucuacacucauuga..................    2     0    c16
.................................................................uGcugugacuagaucuacac.........................    1     1    c16
..................................................................ucAgugacuagaucuacac.........................    1     1    c16
...................................................................cGgugacuagaucuacacucauug...................    1     1    c16
...................................................................cugugacuagaucuacacucauuga..................    1     0    c16
...................................................................cGgugacuagaucuacacucauuga..................    2     1    c16
...................................................................cCgugacuagaucuacacucauuga..................    1     1    c16
...................................................................cuCugacuagaucuacacucauuga..................    1     1    c16
...................................................................Gugugacuagaucuacacucauuga..................    1     1    c16
....................................................................ugugacuagaucuacacuc.......................    1     0    c16
....................................................................Ggugacuagaucuacacuca......................    1     1    c16
....................................................................Ggugacuagaucuacacucau.....................    1     1    c16
....................................................................Agugacuagaucuacacucau.....................    1     1    c16
....................................................................ugugacuagaucuacacucau.....................    1     0    c16
....................................................................Agugacuagaucuacacucauu....................    1     1    c16
....................................................................Ggugacuagaucuacacucauu....................    9     1    c16
....................................................................ugugacuagaucuacacucauu....................    13    0    c16
....................................................................Ggugacuagaucuacacucauug...................    1     1    c16
....................................................................uCugacuagaucuacacucauug...................    2     1    c16
....................................................................ugugacuagaucuacacucauuga..................    1     0    c16
....................................................................uUugacuagaucuacacucauuga..................    2     1    c16
....................................................................ugugacuagaucuacacucauuAa..................    1     1    c16
....................................................................uCugacuagaucuacacucauuga..................    15    1    c16
....................................................................Agugacuagaucuacacucauuga..................    1     1    c16
....................................................................Ggugacuagaucuacacucauuga..................    4     1    c16
.....................................................................Cugacuagaucuacacucauu....................    20    1    c16
.....................................................................gugacuagaucuacacucauug...................    1     0    c16
.....................................................................Cugacuagaucuacacucauug...................    32    1    c16
.....................................................................Uugacuagaucuacacucauug...................    1     1    c16
.....................................................................Augacuagaucuacacucauuga..................    2     1    c16
.....................................................................gugacuagaucuacacucauuga..................    2     0    c16
.....................................................................Uugacuagaucuacacucauuga..................    3     1    c16
.....................................................................gGgacuagaucuacacucauuga..................    1     1    c16
.....................................................................Cugacuagaucuacacucauuga..................    307   1    c16
.....................................................................Cugacuagaucuacacucauugag.................    1     1    c16
......................................................................ugCcuagaucuacacuca......................    1     1    c16
......................................................................ugacuagaucuacacuca......................    300   0    c16
......................................................................ugacuagaucAacacuca......................    2     1    c16
......................................................................Agacuagaucuacacuca......................    9     1    c16
......................................................................ugacuagaucuacacuAa......................    1     1    c16
......................................................................Ggacuagaucuacacuca......................    195   1    c16
......................................................................ugacuagCucuacacuca......................    1     1    c16
......................................................................ugacuagaucuacacucG......................    2     1    c16
......................................................................ugacuagaucuacacucC......................    2     1    c16
......................................................................ugacuagaucuacacuGa......................    1     1    c16
......................................................................ugacuaCaucuacacuca......................    1     1    c16
......................................................................ugacuagaucuacacucU......................    1     1    c16
......................................................................ugacuagaucuacGcuca......................    1     1    c16
......................................................................ugacuagauAuacacuca......................    1     1    c16
......................................................................ugaAuagaucuacacucau.....................    1     1    c16
......................................................................ugacuagaucuacacucUu.....................    2     1    c16
......................................................................Ggacuagaucuacacucau.....................    179   1    c16
......................................................................ugacuaCaucuacacucau.....................    1     1    c16
```

Star Mature

```
aagaaagaaguugaaaguaccuacuuguucuuaauggguauauuucuaguaacacagugcauuauuucugugacuagaucuacacucauugaguacguuuaggacaacau
......................................................................Agacuagaucuacacucau.....................   7     1   c16
......................................................................ugacuagaucuacacCcau.....................   1     1   c16
......................................................................ugacuagUucuacacucau.....................   1     1   c16
......................................................................ugacuagaucuacacucau.....................   235   0   c16
......................................................................ugacuaUaucuacacucau.....................   2     1   c16
......................................................................ugacuagauUuacacucau.....................   1     1   c16
......................................................................ugacuagaucuaUacucau.....................   1     1   c16
......................................................................ugacuagaucuCcacucau.....................   1     1   c16
......................................................................ugacuagaucuacacucaA.....................   2     1   c16
......................................................................ugacuagCucuacacucau.....................   1     1   c16
......................................................................ugacuagaucuacacucaC.....................   1     1   c16
......................................................................ugacuagaAcuacacucauu....................   2     1   c16
......................................................................ugacuagaucuacacucauu....................   2764  0   c16
......................................................................ugacuagaucuacacuUauu....................   1     1   c16
......................................................................ugacuagaucuacacucGuu....................   1     1   c16
......................................................................uCacuagaucuacacucauu....................   2     1   c16
......................................................................ugacuagaucuacacucaAu....................   1     1   c16
......................................................................ugacuagaucuacacGcauu....................   1     1   c16
......................................................................ugacuagaGcuacacucauu....................   1     1   c16
......................................................................Ggacuagaucuacacucauu....................   1736  1   c16
......................................................................ugacuagauUuacacucauu....................   4     1   c16
......................................................................ugaAuagaucuacacucauu....................   2     1   c16
......................................................................ugacuagaucuaAacucauu....................   3     1   c16
......................................................................ugacuaUaucuacacucauu....................   2     1   c16
......................................................................ugacuagaucuacacucauC....................   5     1   c16
......................................................................ugaGuagaucuacacucauu....................   2     1   c16
......................................................................ugacGagaucuacacucauu....................   2     1   c16
......................................................................ugacuagCucuacacucauu....................   10    1   c16
......................................................................ugacuagaucuacacucUuu....................   1     1   c16
......................................................................uUacuagaucuacacucauu....................   1     1   c16
......................................................................Agacuagaucuacacucauu....................   55    1   c16
......................................................................ugacuagaucuacGcucauu....................   2     1   c16
......................................................................ugacuagauAuacacucauu....................   4     1   c16
......................................................................uAacuagaucuacacucauu....................   1     1   c16
......................................................................ugacuGgaucuacacucauu....................   1     1   c16
......................................................................ugacuagaucuacacuAauu....................   1     1   c16
......................................................................ugacuagaucuacacucaGu....................   2     1   c16
......................................................................ugCcuagaucuacacucauu....................   8     1   c16
......................................................................Cgacuagaucuacacucauu....................   3     1   c16
......................................................................ugacuagaucuaUacucauu....................   1     1   c16
......................................................................ugacuagaucuacUcucauu....................   2     1   c16
......................................................................ugacuagaucuacacAcauu....................   2     1   c16
......................................................................ugacuagaucAacacucauu....................   1     1   c16
......................................................................ugacuagaucuacacucCuu....................   1     1   c16
......................................................................ugacuagaucuacCcucauu....................   14    1   c16
......................................................................ugacCagaucuacacucauu....................   1     1   c16
......................................................................ugacuagaucuacacucauA....................   2     1   c16
......................................................................ugacuagaucuUcacucauu....................   2     1   c16
......................................................................ugacuaCaucuacacucauu....................   5     1   c16
......................................................................ugacuagaucCacacucauu....................   5     1   c16
......................................................................ugacuagaucuacaAucauu....................   1     1   c16
......................................................................ugaAuagaucuacacucauug...................   3     1   c16
......................................................................uUacuagaucuacacucauug...................   3     1   c16
......................................................................ugacuagaucuacacucauCg...................   1     1   c16
......................................................................ugacuagaucuaGacucauug...................   2     1   c16
......................................................................ugacAagaucuacacucauug...................   3     1   c16
......................................................................ugacuagaucuacacuAauug...................   5     1   c16
......................................................................ugacuagaucuacacuGauug...................   2     1   c16
......................................................................uCacuagaucuacacucauug...................   3     1   c16
......................................................................ugacuagaucuacacucGuug...................   2     1   c16
......................................................................ugacuagaucCacacucauug...................   8     1   c16
......................................................................ugacuagaucGacacucauug...................   2     1   c16
......................................................................ugacuagaucuCcacucauug...................   25    1   c16
......................................................................Agacuagaucuacacucauug...................   154   1   c16
......................................................................ugacuagaucuacacucaCug...................   5     1   c16
......................................................................ugacuagaucuacGcucauug...................   5     1   c16
......................................................................ugacuagauUuacacucauug...................   5     1   c16
......................................................................ugacuagaucuacacucCuug...................   3     1   c16
......................................................................ugacuaUaucuacacucauug...................   3     1   c16
......................................................................ugacuaCaucuacacucauug...................   1     1   c16
```

```
                                Star                                    Mature
aagaaagaaguugaaaguaccuacuuguucuuaauggguauauuucuaguaacacagugcauuauuucugugacuagaucuacacucauugaguacguuuaggacaacau
......................................................................ugacuagauGuacacucauug...................   3      1   c16
......................................................................ugacuagaAcuacacucauug...................   2      1   c16
......................................................................ugacuagaucuacacGcauug...................   1      1   c16
......................................................................Ggacuagaucuacacucauug...................   4525   1   c16
......................................................................ugacuagaucuGcacucauug...................   2      1   c16
......................................................................ugacuagaucAacacucauug...................   4      1   c16
......................................................................ugacuagaucuacacuUauug...................   2      1   c16
......................................................................ugacGagaucuacacucauug...................   3      1   c16
......................................................................ugacuagaucuacacucauAg...................   5      1   c16
......................................................................Cgacuagaucuacacucauug...................   6      1   c16
......................................................................ugacuagaucuacCcucauug...................   38     1   c16
......................................................................ugacuGgaucuacacucauug...................   1      1   c16
......................................................................ugacuagaCcuacacucauug...................   3      1   c16
......................................................................ugacuagaucuacacCcauug...................   2      1   c16
......................................................................ugacuagaucuacaGucauug...................   3      1   c16
......................................................................ugaGuagaucuacacucauug...................   1      1   c16
......................................................................ugacuagUucuacacucauug...................   1      1   c16
......................................................................ugacuagaucuacacucauuC...................   9      1   c16
......................................................................ugacuagaucuacacAcauug...................   5      1   c16
......................................................................ugacuagaucuacaUucauug...................   8      1   c16
......................................................................ugacuagaucuaUacucauug...................   8      1   c16
......................................................................ugacuagaucuaAacucauug...................   6      1   c16
......................................................................ugacuagaucuacUcucauug...................   3      1   c16
......................................................................ugacuagCucuacacucauug...................   24     1   c16
......................................................................ugacuagaucuacacucauug...................   7184   0   c16
......................................................................uAacuagaucuacacucauug...................   3      1   c16
......................................................................ugacuagaucuacaAucauug...................   8      1   c16
......................................................................ugacuagaucuUcacucauug...................   4      1   c16
......................................................................ugacuagGucuacacucauug...................   3      1   c16
......................................................................ugacuagaucuacacucauuA...................   5      1   c16
......................................................................ugCcuagaucuacacucauug...................   33     1   c16
......................................................................ugacuagaucuacacucaGug...................   1      1   c16
......................................................................ugacuagaucuacacucauuU...................   6      1   c16
......................................................................ugUcuagaucuacacucauug...................   2      1   c16
......................................................................ugacuagaGcuacacucauug...................   1      1   c16
......................................................................ugaUuagaucuacacucauug...................   3      1   c16
......................................................................ugacuagauAuacacucauug...................   7      1   c16
......................................................................ugacuagaucuacacucaAug...................   3      1   c16
......................................................................ugacuUgaucuacacucauug...................   1      1   c16
......................................................................ugacuagaucuacacucUuuga..................   14     1   c16
......................................................................ugacuagaucuaGacucauuga..................   18     1   c16
......................................................................ugacuagauGuacacucauuga..................   15     1   c16
......................................................................ugacCagaucuacacucauuga..................   10     1   c16
......................................................................ugacuagaucuacacucauuUa..................   25     1   c16
......................................................................ugacuagaGcuacacucauuga..................   18     1   c16
......................................................................ugacuagaucuacacucaAuga..................   15     1   c16
......................................................................ugacuagUucuacacucauuga..................   22     1   c16
......................................................................ugacuGgaucuacacucauuga..................   15     1   c16
......................................................................ugacuagaucuacaAucauuga..................   53     1   c16
......................................................................ugacuagaucCacacucauuga..................   42     1   c16
......................................................................ugacuagaucuacacucGuuga..................   15     1   c16
......................................................................ugacuagaucuacacGcauuga..................   12     1   c16
......................................................................Ggacuagaucuacacucauuga..................   38760  1   c16
......................................................................ugaUuagaucuacacucauuga..................   24     1   c16
......................................................................ugacuagaucuacacuAauuga..................   52     1   c16
......................................................................ugacuagaucuacacucauAga..................   31     1   c16
......................................................................ugUcuagaucuacacucauuga..................   16     1   c16
......................................................................ugacuagaucuacacucCuuga..................   16     1   c16
......................................................................ugacuagaCcuacacucauuga..................   18     1   c16
......................................................................Agacuagaucuacacucauuga..................   1471   1   c16
......................................................................ugacuagaucuGcacucauuga..................   26     1   c16
......................................................................ugacuagaucuacacuUauuga..................   22     1   c16
......................................................................ugacuagaucuCcacucauuga..................   101    1   c16
......................................................................ugacAagaucuacacucauuga..................   27     1   c16
......................................................................ugacuaAaucuacacucauuga..................   11     1   c16
......................................................................Cgacuagaucuacacucauuga..................   79     1   c16
......................................................................ugacuaCaucuacacucauuga..................   37     1   c16
......................................................................ugacuagauUuacacucauuga..................   34     1   c16
......................................................................ugacuaUaucuacacucauuga..................   35     1   c16
......................................................................ugacuagauAuacacucauuga..................   82     1   c16
```

```
                                Star                                 Mature
aagaaagaaguugaaaguaccuacuuguucuuaauggguauauuucuaguaacacagugcauuauuucugugacuagaucuacacucauugaguacguuuaggacaacau
......................................................................ugaGuagaucuacacucauuga..................   21      1   c16
......................................................................ugacuagaucuacacAcauuga..................   20      1   c16
......................................................................ugacuagaucuacacCcauuga..................   7       1   c16
......................................................................ugacuagaucGacacucauuga..................   8       1   c16
......................................................................uCacuagaucuacacucauuga..................   22      1   c16
......................................................................ugacuagaucuacacucaGuga..................   37      1   c16
......................................................................ugacuagaucuacUcucauuga..................   33      1   c16
......................................................................ugacuagaucuacacucauugG..................   41      1   c16
......................................................................ugacuagaucuacacucauCga..................   31      1   c16
......................................................................ugGcuagaucuacacucauuga..................   13      1   c16
......................................................................ugaAuagaucuacacucauuga..................   50      1   c16
......................................................................ugacuagCucuacacucauuga..................   151     1   c16
......................................................................ugacuagaucuacaUucauuga..................   35      1   c16
......................................................................ugacuagaucuacacucauugU..................   143     1   c16
......................................................................ugacuagaAcuacacucauuga..................   22      1   c16
......................................................................uUacuagaucuacacucauuga..................   20      1   c16
......................................................................ugacuagaucuacGcucauuga..................   25      1   c16
......................................................................ugacuagaucuacaGucauuga..................   17      1   c16
......................................................................ugCcuagaucuacacucauuga..................   210     1   c16
......................................................................ugacuagaucuacacucauGga..................   6       1   c16
......................................................................ugacuagaucuaAcucauuga..................   28      1   c16
......................................................................ugacuCgaucuacacucauuga..................   4       1   c16
......................................................................ugacuUgaucuacacucauuga..................   10      1   c16
......................................................................ugacuagGucuacacucauuga..................   16      1   c16
......................................................................uAacuagaucuacacucauuga..................   19      1   c16
......................................................................ugacuagaucuUcacucauuga..................   21      1   c16
......................................................................ugacuagaucuacacucaCuga..................   14      1   c16
......................................................................ugacuagaucAacacucauuga..................   29      1   c16
......................................................................ugacuagaucuaUacucauuga..................   46      1   c16
......................................................................ugacuagaucuacacuGauuga..................   6       1   c16
......................................................................ugacuagaucuacacucauuCa..................   41      1   c16
......................................................................ugacuagaucuacCcucauuga..................   265     1   c16
......................................................................ugacGagaucuacacucauuga..................   8       1   c16
......................................................................ugacuagaucuacacucauugC..................   58      1   c16
......................................................................ugacuagaucuacacucauuAa..................   7       1   c16
......................................................................ugacuagaucuacacucauuga..................   62364   0   c16
......................................................................Agacuagaucuacacucauugag.................   2       1   c16
......................................................................ugaUuagaucuacacucauugag.................   1       1   c16
......................................................................ugacuagaucuacCcucauugag.................   1       1   c16
......................................................................ugCcuagaucuacacucauugag.................   1       1   c16
......................................................................ugacuagaucuacacucauugGg.................   3       1   c16
......................................................................ugacuagaucuacaAucauugag.................   1       1   c16
......................................................................ugacuagaucuacacucauugaC.................   38      1   c16
......................................................................uUacuagaucuacacucauugag.................   1       1   c16
......................................................................ugacuagaucuacacucauugaA.................   3014    1   c16
......................................................................Ggacuagaucuacacucauugag.................   74      1   c16
......................................................................ugacuagaucuacacucauugUg.................   2       1   c16
......................................................................ugacuagaucuacacucauugag.................   122     0   c16
......................................................................ugacuagaucuacacucauugaU.................   242     1   c16
......................................................................ugacuagUucuacacucauugagu................   1       1   c16
......................................................................ugacuagaucuacacucauugagu................   16      0   c16
......................................................................ugacuagaucuacacucauugaAu................   14      1   c16
......................................................................ugacuagaucuacacucauugagA................   8       1   c16
......................................................................ugacuagaucuacacucauugaUu................   5       1   c16
......................................................................Ggacuagaucuacacucauugagu................   7       1   c16
......................................................................ugacuagaucuacacucauugagC................   4       1   c16
......................................................................ugacuagaucuacacucauugagG................   7       1   c16
......................................................................ugacuagaucuacacucauugaCu................   1       1   c16
......................................................................ugacuagaucuacacucauugagua...............   1       0   c16
......................................................................ugacuagaucuacacucauugagGa...............   2       1   c16
.......................................................................gacuagaucuacacucau.....................   1       0   c16
.......................................................................gGcuagaucuacacucauu....................   1       1   c16
.......................................................................gacuagaucuacacucauu....................   4       0   c16
.......................................................................gacuagaucuacaUucauug...................   1       1   c16
.......................................................................gacuagaucuacacucauug...................   10      0   c16
.......................................................................gacuagaucuacCcucauug...................   1       1   c16
.......................................................................gacuagaucuaUacucauuga..................   1       1   c16
.......................................................................Cacuagaucuacacucauuga..................   1       1   c16
.......................................................................gacuagaucuacacuAauuga..................   1       1   c16
.......................................................................gacuagaucuacCcucauuga..................   1       1   c16
```

Star **Mature**

```
aagaaagaaguugaaaguaccuacuuguucuuaauggguauauuucuaguaacacagugcauuauuucugugacuagaucuacacucauugaguacguuuaggacaacau
.......................................................................gacuagaucuacacucaAuga..................	1	1	c16
.......................................................................gUcuagaucuacacucauuga..................	1	1	c16
.......................................................................Uacuagaucuacacucauuga..................	2	1	c16
.......................................................................gacuagaucuacacucaGuga..................	1	1	c16
.......................................................................gacuagaucuacaUucauuga..................	1	1	c16
.......................................................................gacuagauAuacacucauuga..................	1	1	c16
.......................................................................gacuagaucuacacGcauuga..................	1	1	c16
.......................................................................gGcuagaucuacacucauuga..................	4	1	c16
.......................................................................gacuagaucuacacucauuga..................	150	0	c16
.......................................................................gaAuagaucuacacucauuga..................	1	1	c16
.......................................................................gacuagaucuacacucauugaA.................	2	1	c16
........................................................................aGuagaucuacacucauug...................	1	1	c16
........................................................................acuagaucuacacucauug...................	2	0	c16
........................................................................Ccuagaucuacacucauuga..................	8	1	c16
........................................................................aUuagaucuacacucauuga..................	1	1	c16
........................................................................acuagaucuacacucauuga..................	25	0	c16
........................................................................acuagaucuacacucauuUa..................	1	1	c16
........................................................................acuagaucuacacucauugaA.................	3	1	c16
.........................................................................cuagaucuacacucauug...................	47	0	c16
.........................................................................cuagaucuacCcucauug...................	3	1	c16
.........................................................................cuagaucuaAacucauug...................	1	1	c16
.........................................................................cuagCucuacacucauug...................	3	1	c16
.........................................................................cuagaucuacaAucauuga..................	1	1	c16
.........................................................................cuagaAcuacacucauuga..................	2	1	c16
.........................................................................cuagaucuacacucauuCa..................	1	1	c16
.........................................................................cuagaucuacacucauuga..................	401	0	c16
.........................................................................cuagaucuaAacucauuga..................	1	1	c16
.........................................................................cuagauAuacacucauuga..................	1	1	c16
.........................................................................cGagaucuacacucauuga..................	2	1	c16
.........................................................................cuagaucuacacucauugU..................	1	1	c16
.........................................................................cuagaucuacacucauCga..................	1	1	c16
.........................................................................cuagaucuacacucauuUa..................	1	1	c16
.........................................................................cuagCucuacacucauuga..................	14	1	c16
.........................................................................cuagaucuCcacucauuga..................	1	1	c16
.........................................................................cuagaucuacacucaGuga..................	1	1	c16
.........................................................................cuaUaucuacacucauuga..................	1	1	c16
.........................................................................cuGgaucuacacucauuga..................	1	1	c16
.........................................................................cuagaucuacCcucauuga..................	3	1	c16
.........................................................................cuagauUuacacucauuga..................	1	1	c16
.........................................................................cuagaucuacGcucauuga..................	1	1	c16
.........................................................................Uuagaucuacacucauuga..................	1	1	c16
.........................................................................cuagaucuacacucaCuga..................	1	1	c16
.........................................................................cuagaucuacacucauugaU.................	1	1	c16
.........................................................................cuagaucuacacucauugag.................	2	0	c16
.........................................................................cuagaucuacacucauugaA.................	14	1	c16
..........................................................................uagaucuacacucauuga..................	20	0	c16
..........................................................................Gagaucuacacucauuga..................	10	1	c16
..........................................................................uagaucuacacucauugaA.................	1	1	c16
...........................................................................agaucuacacucauugaA.................	1	1	c16
...........................................................................agaucuacacucauugag.................	1	0	c16
............................................................................................guacguuuaggacaacau	1	0	c16

................................aauggguauauuucuagu............................................................	1	0	c04
................................aauggguauauuucuagua...........................................................	2	0	c04
................................aauggguauauuucuaguaa..........................................................	1	0	c04
................................aauggguauauuucuaguaac.........................................................	6	0	c04
................................aauggguauauuucuaguaaca........................................................	11	0	c04
................................aaugCguauauuucuaguaacac.......................................................	1	1	c04
................................aauggguauauuucuaguaacac.......................................................	20	0	c04
................................aauggguauauuucuaguaacaA.......................................................	1	1	c04
................................aauggguauauuucCaguaacaca......................................................	1	1	c04
................................aauggguauauuucuaguaacacU......................................................	1	1	c04
................................aauggguauauuucuaguaacCca......................................................	1	1	c04
................................aauggguauauCucuaguaacaca......................................................	1	1	c04
................................Cauggguauauuucuaguaacaca......................................................	1	1	c04
................................aauggguauauuucuaguaacaca......................................................	54	0	c04
................................aauggguauauuucuaguaacacaA.....................................................	8	1	c04
..................................uggguauauuucuaguaac.........................................................	1	0	c04
...................................ggguauauuucuaguaacac.......................................................	1	0	c04
........................................................gugcauuauuucugugacuagaucuacacucauuga..................	1	0	c04
```

Star Mature			
aagaaagaaguugaaaguaccuacuuguucuuaauggguauauuucuaguaacacagugcauuauuucugugacuagaucuacacucauugaguacguuuaggacaacau			
....uucGgugacuagaucuacacucauuga..................	1	1	c04
....ucuUugacuagaucuacacucauuga..................	1	1	c04
....Gugugacuagaucuacacucauu....................	1	1	c04
....uCugacuagaucuacacuc.......................	1	1	c04
....uCugacuagaucuacacucauuga..................	5	1	c04
....Ggugacuagaucuacacucauuga..................	2	1	c04
....Cugacuagaucuacacuc.......................	2	1	c04
....Cugacuagaucuacacuca......................	1	1	c04
....Cugacuagaucuacacucauu....................	1	1	c04
....Cugacuagaucuacacucauug...................	1	1	c04
....gugacuagaucuacacucauug...................	1	0	c04
....Cugacuagaucuacacucauuga..................	67	1	c04
....ugacuagaucuacacuAa......................	1	1	c04
....ugacuagaucuacacuGa......................	1	1	c04
....ugacuagaucuacacucG......................	3	1	c04
....ugacuagaucuacacucU......................	1	1	c04
....ugacuagauGuacacuca......................	1	1	c04
....ugacuagaucuacacucC......................	1	1	c04
....Ggacuagaucuacacuca......................	47	1	c04
....ugacuagCucuacacuca......................	1	1	c04
....Agacuagaucuacacuca......................	6	1	c04
....ugacuagaucuacacuUa......................	2	1	c04
....ugacuagaucuacacuca......................	141	0	c04
....ugacuagaucuacacuAau.....................	1	1	c04
....Ggacuagaucuacacucau.....................	53	1	c04
....ugacuagaucuacaAucau.....................	1	1	c04
....ugacuagaucuacacucaC.....................	3	1	c04
....ugacuagaucuacacucaG.....................	1	1	c04
....ugacuagaucuacacucUu.....................	3	1	c04
....ugacuagauUuacacucau.....................	1	1	c04
....ugacuagaucuacacucaA.....................	2	1	c04
....ugacuagaucuacacuUau.....................	1	1	c04
....ugacuagaucuacacucGu.....................	1	1	c04
....ugacuagaucuacacucau.....................	112	0	c04
....ugacuagaucuCcacucau.....................	1	1	c04
....ugacuagaucuacacucauA....................	3	1	c04
....ugacuagaucuacCcucauu....................	4	1	c04
....ugacuagaucuacaAucauu....................	1	1	c04
....ugacuagaucuacacucauC....................	3	1	c04
....ugacuagaucuacacucGuu....................	1	1	c04
....ugacuagaucuGcacucauu....................	1	1	c04
....ugaAuagaucuacacucauu....................	1	1	c04
....ugacuagaucuacacucaGu....................	1	1	c04
....ugacuagauUuacacucauu....................	1	1	c04
....Ggacuagaucuacacucauu....................	275	1	c04
....ugacuagaucuacacuUauu....................	1	1	c04
....ugacuagCucuacacucauu....................	1	1	c04
....ugacuagaucuacacCcauu....................	1	1	c04
....ugacuagaucuacacucauu....................	656	0	c04
....ugacuUgaucuacacucauu....................	1	1	c04
....ugacuagaucuacacucaCu....................	1	1	c04
....ugacuagaGcuacacucauu....................	1	1	c04
....Agacuagaucuacacucauu....................	7	1	c04
....ugacuagaucuaGacucauu....................	1	1	c04
....ugacuagaucAacacucauu....................	1	1	c04
....ugacCagaucuacacucauug...................	1	1	c04
....ugacuagaucuacacuAauug...................	1	1	c04
....ugacuagauAuacacucauug...................	2	1	c04
....ugacuagaucuacaGucauug...................	1	1	c04
....ugacuagaucuacacucauuA...................	5	1	c04
....ugacuagaucuacacAcauug...................	2	1	c04
....ugCcuagaucuacacucauug...................	3	1	c04
....ugacuagaucuacacucauuU...................	9	1	c04
....ugacuagCucuacacucauug...................	4	1	c04
....Ggacuagaucuacacucauug...................	201	1	c04
....ugacuagaucuacacucauug...................	561	0	c04
....ugacuagaucuacaUucauug...................	1	1	c04
....ugacuagaucuacacucauuC...................	2	1	c04
....ugacuagaucuacaAucauug...................	2	1	c04
....ugacuagaucuacacucauAg...................	1	1	c04

Star Mature

```
aagaaagaaguugaaaguaccuacuuguucuuaauggguauauuucuaguaacacagugcauuauuucugugacuagaucuacacucauugaguacguuuaggacaacau
......................................................................ugacuagaucuCcacucauug...................  2      1    c04
......................................................................uUacuagaucuacacucauug...................  1      1    c04
......................................................................ugacuGgaucuacacucauug...................  1      1    c04
......................................................................Agacuagaucuacacucauug...................  6      1    c04
......................................................................ugacuagaucCacacucauug...................  1      1    c04
......................................................................ugacuagaucuacCcucauug...................  2      1    c04
......................................................................ugacuagaucuacacuGauuga..................  1      1    c04
......................................................................ugacuagaucuacacucaGuga..................  4      1    c04
......................................................................ugacuagaucuacacCcauuga..................  2      1    c04
......................................................................ugacuagaucuCcacucauuga..................  11     1    c04
......................................................................ugacuagauUuacacucauuga..................  8      1    c04
......................................................................ugacGagaucuacacucauuga..................  2      1    c04
......................................................................ugacuagaucuacacucGuuga..................  4      1    c04
......................................................................ugacuUgaucuacacucauuga..................  3      1    c04
......................................................................ugacuagCucuacacucauuga..................  17     1    c04
......................................................................ugacuagaucuacaAucauuga..................  8      1    c04
......................................................................ugacuagaucuGcacucauuga..................  5      1    c04
......................................................................ugacuagaucuacacucauuga..................  11297  0    c04
......................................................................ugacuaUaucuacacucauuga..................  3      1    c04
......................................................................Agacuagaucuacacucauuga..................  150    1    c04
......................................................................ugGcuagaucuacacucauuga..................  3      1    c04
......................................................................ugacuagaucuacaUucauuga..................  7      1    c04
......................................................................ugacuagaucuacCcucauuga..................  28     1    c04
......................................................................ugacuagaucuacGcucauuga..................  6      1    c04
......................................................................ugaGuagaucuacacucauuga..................  2      1    c04
......................................................................ugacuagaucuaUacucauuga..................  11     1    c04
......................................................................ugacuagaAcuacacucauuga..................  4      1    c04
......................................................................ugacuagaucuacacucUuuga..................  5      1    c04
......................................................................ugacuagaucuacacucauugU..................  28     1    c04
......................................................................ugacuagauAuacacucauuga..................  12     1    c04
......................................................................ugacuagaGcuacacucauuga..................  5      1    c04
......................................................................ugacuagaucAacacucauuga..................  3      1    c04
......................................................................ugacuagaucuacacucauuCa..................  5      1    c04
......................................................................ugacuagaucuacacucaAuga..................  2      1    c04
......................................................................ugacuagaucuacacGcauuga..................  1      1    c04
......................................................................ugaUuagaucuacacucauuga..................  3      1    c04
......................................................................ugacuagaucuacacuUauuga..................  3      1    c04
......................................................................ugacuagaucuacacucauugC..................  16     1    c04
......................................................................ugacuagaucuacaGucauuga..................  2      1    c04
......................................................................ugacuagaucuacacucauugG..................  6      1    c04
......................................................................ugacuagaucCacacucauuga..................  7      1    c04
......................................................................ugacuagaucuaAacucauuga..................  2      1    c04
......................................................................ugacuagaucuacacucaCuga..................  1      1    c04
......................................................................uAacuagaucuacacucauuga..................  1      1    c04
......................................................................ugacuagGucuacacucauuga..................  9      1    c04
......................................................................ugacuaAaucuacacucauuga..................  1      1    c04
......................................................................ugacuagaucuacacAcauuga..................  2      1    c04
......................................................................ugacuagaCcuacacucauuga..................  3      1    c04
......................................................................ugaAuagaucuacacucauuga..................  3      1    c04
......................................................................ugacuagaucuacacucauCga..................  2      1    c04
......................................................................ugacuagaucuacacucauAga..................  6      1    c04
......................................................................uUacuagaucuacacucauuga..................  4      1    c04
......................................................................Cgacuagaucuacacucauuga..................  7      1    c04
......................................................................ugacAagaucuacacucauuga..................  4      1    c04
......................................................................ugacuaCaucuacacucauuga..................  3      1    c04
......................................................................ugacuagaucuUcacucauuga..................  4      1    c04
......................................................................ugUcuagaucuacacucauuga..................  2      1    c04
......................................................................ugacuagaucuacacucCuuga..................  1      1    c04
......................................................................ugacuagaucuaGacucauuga..................  4      1    c04
......................................................................ugCcuagaucuacacucauuga..................  22     1    c04
......................................................................ugacuagaucuacUcucauuga..................  3      1    c04
......................................................................uCacuagaucuacacucauuga..................  4      1    c04
......................................................................Ggacuagaucuacacucauuga..................  4358   1    c04
......................................................................ugacuagaucuacacucauuUa..................  4      1    c04
......................................................................ugacuagaucuacacuAauuga..................  7      1    c04
......................................................................ugacCagaucuacacucauuga..................  1      1    c04
......................................................................ugacuagUucuacacucauuga..................  4      1    c04
......................................................................ugacuGgaucuacacucauugag.................  1      1    c04
......................................................................ugCcuagaucuacacucauugag.................  1      1    c04
......................................................................Ggacuagaucuacacucauugag.................  29     1    c04
```

Star **Mature**

```
aagaaagaaguugaaaguaccuacuuguucuuaauggguauauuucuaguaacacagugcauuauuucugugacuagaucuacacucauugaguacguuuaggacaacau
......................................................................ugacuagaucuacacucauugaA.................  2231  1  c04
......................................................................Agacuagaucuacacucauugag.................  1     1  c04
......................................................................ugacuagaucuacacucauugaC.................  9     1  c04
......................................................................ugacuagaucuacacucauugaU.................  36    1  c04
......................................................................ugacuagaucuacacucauugag.................  45    0  c04
......................................................................Ggacuagaucuacacucauugagu................  3     1  c04
......................................................................ugacuagaucuacacucauugaUu................  2     1  c04
......................................................................ugacuagaucuacacucauugaCu................  1     1  c04
......................................................................ugacuagaucuacacucauugagG................  2     1  c04
......................................................................ugacuagaucuacacucauugagA................  4     1  c04
......................................................................ugacuagaucuacacucauugagC................  7     1  c04
......................................................................ugacuagaucuacacucauugagu................  9     0  c04
......................................................................ugacuagaucuacacucauugaAu................  8     1  c04
......................................................................ugacuagaucuacacucauugaguU...............  1     1  c04
......................................................................ugacuagaucuacacucauugaguG...............  1     1  c04
......................................................................ugacuagaucuacacucauugaUua...............  1     1  c04
......................................................................ugacuagaucuacacucauugagCa...............  1     1  c04
......................................................................Ggacuagaucuacacucauugaguac..............  1     1  c04
......................................................................ugacuagaucuacacucauugaguaA..............  1     1  c04
......................................................................ugacuagaucuacacucauugaguac..............  1     0  c04
......................................................................ugacuagaucuacacucauugagCac..............  1     1  c04
......................................................................ugacuagaucuacacucauugaguaUg.............  1     1  c04
.......................................................................gacuagaucuacacucauu....................  2     0  c04
.......................................................................gacuagaucuacacucauug...................  1     0  c04
.......................................................................gacuagaucuacacucauuga..................  29    0  c04
.......................................................................Cacuagaucuacacucauuga..................  1     1  c04
.......................................................................gaUuagaucuacacucauuga..................  1     1  c04
.......................................................................gGcuagaucuacacucauuga..................  1     1  c04
.......................................................................gacuagaucuacacucauugaA.................  5     1  c04
........................................................................Ucuagaucuacacucauug...................  1     1  c04
........................................................................acuagaucuacacucauuga..................  4     0  c04
........................................................................Ccuagaucuacacucauuga..................  1     1  c04
........................................................................aUuagaucuacacucauuga..................  1     1  c04
........................................................................acuagaucuacacucauugaA.................  1     1  c04
.........................................................................cuagaucuacacucauug...................  5     0  c04
.........................................................................cuagaucuacacucauuga..................  71    0  c04
.........................................................................cGagaucuacacucauuga..................  1     1  c04
.........................................................................cuagaucuacacucaGuga..................  2     1  c04
.........................................................................cuagCucuacacucauuga..................  3     1  c04
.........................................................................cuagaucuacacucauugaU.................  1     1  c04
.........................................................................cuagaucuacacucauugaA.................  17    1  c04
..........................................................................uagaucuacacucauuga..................  12    0  c04
..........................................................................Gagaucuacacucauuga..................  5     1  c04
..........................................................................Aagaucuacacucauuga..................  1     1  c04
..........................................................................uagaucuacacucauugaA.................  3     1  c04
...........................................................................agaucuacacucauugaA.................  7     1  c04

................................Cauggguauauuucuagu............................................................  1     1  c02
................................aauggguauauuucuagu............................................................  5     0  c02
................................aauggguauauuucuagua...........................................................  2     0  c02
................................aauggguauauuucuaguaa..........................................................  2     0  c02
................................aauggguauauuucuaguaac.........................................................  2     0  c02
................................aauggguauauuucuaguaaA.........................................................  1     1  c02
................................aauggguauauuucuaguaaca........................................................  21    0  c02
................................aauggguauauuucuaguaacac.......................................................  33    0  c02
................................Cauggguauauuucuaguaacac.......................................................  1     1  c02
................................Cauggguauauuucuaguaacaca......................................................  3     1  c02
................................aauCgguauauuucuaguaacaca......................................................  2     1  c02
................................aauggguauauuucuaguaacaca......................................................  109   0  c02
................................aauggguauauuucuaCuaacaca......................................................  1     1  c02
................................aauggguauauuucuaguaacaAa......................................................  1     1  c02
................................aCuggguauauuucuaguaacaca......................................................  1     1  c02
................................aauggguauauuucuaguaacacaA.....................................................  3     1  c02
................................aauggguauauuucuaguaaAacagu....................................................  1     1  c02
.................................auggguauauuucuaguaacaca......................................................  1     0  c02
.................................auggguauauuucuaguaacacag.....................................................  1     0  c02
..................................uggguauauuucuaguaaca........................................................  1     0  c02
..................................uggguauauuucuaguaacaca......................................................  2     0  c02
..................................uggguauauuucuaguaacacag.....................................................  1     0  c02
...................................ggguauauuucuaguaacaca......................................................  1     0  c02
```

Star Mature

```
aagaaagaaguugaaaguaccuacuuguucuuaauggguauauuucuaguaacacagugcauuauuucugugacuagaucuacacucauugaguacguuuaggacaacau
...................................................................cugugacuagaucuacacucauuga..................	1	0	c02
....................................................................uCugacuagaucuacacu........................	1	1	c02
....................................................................Ggugacuagaucuacacuc.......................	2	1	c02
....................................................................uCugacuagaucuacacucauu....................	1	1	c02
....................................................................Ggugacuagaucuacacucauu....................	2	1	c02
....................................................................ugugacuagaucuacacucauu....................	8	0	c02
....................................................................uCugacuagaucuacacucauug...................	1	1	c02
....................................................................ugugacuagaucuacacucauug...................	1	0	c02
....................................................................ugugacuagaucuacacucauuA...................	1	1	c02
....................................................................uCugacuagaucuacacucauuga..................	1	1	c02
.....................................................................Cugacuagaucuacacuc.......................	1	1	c02
.....................................................................Cugacuagaucuacacucauu....................	1	1	c02
.....................................................................Cugacuagaucuacacucauug...................	3	1	c02
.....................................................................Cugacuagaucuacacucauuga..................	27	1	c02
......................................................................Ggacuagaucuacacuca......................	89	1	c02
......................................................................ugacuagaucuacCcuca......................	1	1	c02
......................................................................ugacuagaucuacacuca......................	109	0	c02
......................................................................ugacuagaucuacacucC......................	1	1	c02
......................................................................ugacuagaucuacacucG......................	1	1	c02
......................................................................Agacuagaucuacacuca......................	2	1	c02
......................................................................ugaUuagaucuacacucau.....................	1	1	c02
......................................................................ugacuagaucuacacucGu.....................	1	1	c02
......................................................................ugacuagaucuacacAcau.....................	1	1	c02
......................................................................ugacuagaucuacacucau.....................	60	0	c02
......................................................................Agacuagaucuacacucau.....................	1	1	c02
......................................................................ugacuagaucuacacucaA.....................	1	1	c02
......................................................................Ggacuagaucuacacucau.....................	35	1	c02
......................................................................ugacuagaucuacacucUu.....................	1	1	c02
......................................................................ugacuagaucuacaGucauu....................	1	1	c02
......................................................................ugacGagaucuacacucauu....................	1	1	c02
......................................................................ugacuagaucuacCcucauu....................	2	1	c02
......................................................................ugacuGgaucuacacucauu....................	1	1	c02
......................................................................ugacuagaucuacacuGauu....................	1	1	c02
......................................................................ugCcuagaucuacacucauu....................	1	1	c02
......................................................................ugacuagCucuacacucauu....................	2	1	c02
......................................................................ugacuaCaucuacacucauu....................	1	1	c02
......................................................................ugacuagaucuCcacucauu....................	2	1	c02
......................................................................ugacuagaucuaUacucauu....................	2	1	c02
......................................................................Ggacuagaucuacacucauu....................	400	1	c02
......................................................................Agacuagaucuacacucauu....................	16	1	c02
......................................................................ugacuagaucuacacucauu....................	558	0	c02
......................................................................ugacuagaucuacaAucauu....................	1	1	c02
......................................................................ugacuagaucuacacucaGu....................	6	1	c02
......................................................................ugacuagaucuacacucGuu....................	1	1	c02
......................................................................ugacuagaucuacacucauA....................	2	1	c02
......................................................................ugaAuagaucuacacucauug...................	4	1	c02
......................................................................ugacuagaucCacacucauug...................	1	1	c02
......................................................................ugacuagaucuacacucauuC...................	2	1	c02
......................................................................ugacuagauUuacacucauug...................	1	1	c02
......................................................................Cgacuagaucuacacucauug...................	1	1	c02
......................................................................ugacuagaucuacacucauuA...................	3	1	c02
......................................................................ugacuagaucuacacucaAug...................	1	1	c02
......................................................................ugacuagaucuacacuGauug...................	1	1	c02
......................................................................ugacuagauAuacacucauug...................	2	1	c02
......................................................................ugacuagaucuacaUucauug...................	2	1	c02
......................................................................ugacuagaucuaGacucauug...................	1	1	c02
......................................................................ugacuagaucuGcacucauug...................	1	1	c02
......................................................................ugacuagaucuaUacucauug...................	4	1	c02
......................................................................Ggacuagaucuacacucauug...................	1285	1	c02
......................................................................ugacuagaucuacacucauug...................	1612	0	c02
......................................................................ugCcuagaucuacacucauug...................	10	1	c02
......................................................................ugacuagaucuCcacucauug...................	1	1	c02
......................................................................ugacuagCucuacacucauug...................	6	1	c02
......................................................................ugacuagaucuacUcucauug...................	1	1	c02
......................................................................ugacuagaucuacCcucauug...................	11	1	c02
......................................................................Agacuagaucuacacucauug...................	48	1	c02
......................................................................ugacuagaucuacaGucauug...................	1	1	c02
......................................................................ugacuagaucuacacucGuug...................	1	1	c02
......................................................................ugacuagaucuacacuUauug...................	1	1	c02
......................................................................ugacuagaucuacacucauGg...................	1	1	c02
```

Star Mature

```
aagaaagaaguugaaaguaccuacuuguucuuaauggguauauuucuaguaacacagugcauuauuucugugacuagaucuacacucauugaguacguuuaggacaacau
......................................................................ugacAagaucuacacucauug...................  1      1  c02
......................................................................ugacuagaucuaAacucauug...................  3      1  c02
......................................................................ugacuagaucuacacucauAg...................  1      1  c02
......................................................................uUacuagaucuacacucauug...................  1      1  c02
......................................................................ugacuagaucuacacAcauug...................  2      1  c02
......................................................................ugacuagaucAacacucauug...................  1      1  c02
......................................................................ugacuagaAcuacacucauug...................  1      1  c02
......................................................................ugacuagaucuacGcucauug...................  1      1  c02
......................................................................ugacuagaucuGcacucauuga..................  5      1  c02
......................................................................ugacuagaucuacacucaGuga..................  1      1  c02
......................................................................ugacuagaucuacacAcauuga..................  3      1  c02
......................................................................ugacuagaCcuacacucauuga..................  3      1  c02
......................................................................ugacuagaucuacacucauuga..................  11414  0  c02
......................................................................ugacuagaucuacacucauCga..................  3      1  c02
......................................................................ugacuagauAuacacucauuga..................  15     1  c02
......................................................................ugacuagaucuacacuAauuga..................  11     1  c02
......................................................................ugacuagaGcuacacucauuga..................  3      1  c02
......................................................................ugacuagaucuacacucGuuga..................  1      1  c02
......................................................................ugacuagaucuacGcucauuga..................  8      1  c02
......................................................................ugacuagaucuaGacucauuga..................  5      1  c02
......................................................................ugacCagaucuacacucauuga..................  2      1  c02
......................................................................ugacuagaucuacacucauGga..................  1      1  c02
......................................................................ugacuagaucCacacucauuga..................  6      1  c02
......................................................................Ggacuagaucuacacucauuga..................  9962   1  c02
......................................................................ugacuagaucuacacucauugG..................  8      1  c02
......................................................................ugacuagaucuacacuUauuga..................  5      1  c02
......................................................................ugacuagaucuacacCcauuga..................  2      1  c02
......................................................................ugacuagaucuacacucauugC..................  19     1  c02
......................................................................uCacuagaucuacacucauuga..................  5      1  c02
......................................................................ugCcuagaucuacacucauuga..................  44     1  c02
......................................................................Agacuagaucuacacucauuga..................  453    1  c02
......................................................................ugacuaUaucuacacucauuga..................  9      1  c02
......................................................................ugacuagaucAacacucauuga..................  5      1  c02
......................................................................ugacuagaucuacUcucauuga..................  5      1  c02
......................................................................ugacAagaucuacacucauuga..................  4      1  c02
......................................................................ugaUuagaucuacacucauuga..................  2      1  c02
......................................................................ugacuagaucuacacucaAuga..................  5      1  c02
......................................................................ugaAuagaucuacacucauuga..................  9      1  c02
......................................................................ugaGuagaucuacacucauuga..................  3      1  c02
......................................................................ugacuagaucuCcacucauuga..................  19     1  c02
......................................................................Cgacuagaucuacacucauuga..................  11     1  c02
......................................................................uAacuagaucuacacucauuga..................  2      1  c02
......................................................................ugacuagaucuacacucauuUa..................  6      1  c02
......................................................................ugacuagaucuaUacucauuga..................  23     1  c02
......................................................................ugacuagaucuacacucauAga..................  7      1  c02
......................................................................ugacuagaucuacacucauuAa..................  1      1  c02
......................................................................ugacuagaucuacacucauugU..................  10     1  c02
......................................................................ugacuagGucuacacucauuga..................  1      1  c02
......................................................................ugGcuagaucuacacucauuga..................  3      1  c02
......................................................................ugacuagaucuacaGucauuga..................  4      1  c02
......................................................................ugacGagaucuacacucauuga..................  1      1  c02
......................................................................ugacuagaucuacaAucauuga..................  9      1  c02
......................................................................ugacuagaucuacacGcauuga..................  1      1  c02
......................................................................ugacuaAaucuacacucauuga..................  4      1  c02
......................................................................ugacuagaucGacacucauuga..................  1      1  c02
......................................................................ugacuagaucuacaUucauuga..................  5      1  c02
......................................................................ugacuagaucuaAacucauuga..................  10     1  c02
......................................................................ugacuagaAcuacacucauuga..................  9      1  c02
......................................................................ugacuagauUuacacucauuga..................  11     1  c02
......................................................................ugacuagaucuacacucauuCa..................  9      1  c02
......................................................................ugacuagaucuacCcucauuga..................  71     1  c02
......................................................................ugacuagCucuacacucauuga..................  33     1  c02
......................................................................ugacuagaucuacacucCuuga..................  3      1  c02
......................................................................uUacuagaucuacacucauuga..................  7      1  c02
......................................................................ugacuUgaucuacacucauuga..................  2      1  c02
......................................................................ugacuagauGuacacucauuga..................  4      1  c02
......................................................................ugacuaCaucuacacucauuga..................  7      1  c02
......................................................................ugacuagaucuacacucUuuga..................  2      1  c02
......................................................................ugacuagaucuacacucaCuga..................  4      1  c02
......................................................................ugacuGgaucuacacucauuga..................  5      1  c02
```

Star / Mature

```
aagaaagaaguugaaaguaccuacuuguucuuaauggguauauuucuaguaacacagugcauuauuucugugacuagaucuacacucauugaguacguuuaggacaacau
......................................................................ugacuagUucuacacucauuga..................  1    1  c02
......................................................................ugUcuagaucuacacucauuga..................  1    1  c02
......................................................................ugacuagaucuUcacucauuga..................  5    1  c02
......................................................................ugacuagaucuacacucauugaU.................  40   1  c02
......................................................................ugacuagaucuacacucauugag.................  15   0  c02
......................................................................ugacuagaucuacacucauugaA.................  490  1  c02
......................................................................Ggacuagaucuacacucauugag.................  21   1  c02
......................................................................Agacuagaucuacacucauugag.................  1    1  c02
......................................................................ugacuagaucuacacucauugCg.................  2    1  c02
......................................................................ugacuagaucuacacucauugaC.................  7    1  c02
......................................................................ugacuagaucuacacucauugagC................  2    1  c02
......................................................................Ggacuagaucuacacucauugagu................  5    1  c02
......................................................................ugacuagaucuacacucauugagG................  1    1  c02
......................................................................ugacuagaucuacacucauugaAu................  1    1  c02
......................................................................ugacuagaucuacacucauugagu................  3    0  c02
.......................................................................gacuagaucuacacucauu....................  3    0  c02
.......................................................................gacuagaucuacacuAauu....................  1    1  c02
.......................................................................gacuagaucuacacucauug...................  2    0  c02
.......................................................................gacuagaucuaAacucauuga..................  1    1  c02
.......................................................................gacuagaucuacCcucauuga..................  1    1  c02
.......................................................................Aacuagaucuacacucauuga..................  1    1  c02
.......................................................................gacuagaucuacacucauuga..................  31   0  c02
.......................................................................Uacuagaucuacacucauuga..................  1    1  c02
.......................................................................gUcuagaucuacacucauuga..................  1    1  c02
........................................................................acuagaucuacacucauu....................  1    0  c02
........................................................................acuagaucuacacucauug...................  4    0  c02
........................................................................Ccuagaucuacacucauuga..................  3    1  c02
........................................................................acuagaucuacacucauuga..................  5    0  c02
.........................................................................cuagaucuacacucauug...................  31   0  c02
.........................................................................cuagCucuacacucauug...................  2    1  c02
.........................................................................cuagaucuaAacucauuga..................  1    1  c02
.........................................................................cuagCucuacacucauuga..................  4    1  c02
.........................................................................cuagaucuacacucauugU..................  1    1  c02
.........................................................................cuagaucuacacucGuuga..................  1    1  c02
.........................................................................cuagauAuacacucauuga..................  1    1  c02
.........................................................................cuagaucuacacuAauuga..................  1    1  c02
.........................................................................cuagaucuacacucauuga..................  289  0  c02
.........................................................................cuagaGcuacacucauuga..................  1    1  c02
.........................................................................cuagaucuacaGucauuga..................  1    1  c02
.........................................................................cuagaucuacGcucauuga..................  1    1  c02
.........................................................................cuagaucuCcacucauuga..................  1    1  c02
.........................................................................cuagaucGacacucauuga..................  1    1  c02
.........................................................................cuagaucuacCcucauuga..................  4    1  c02
.........................................................................cuagaucuacacucauugaA.................  9    1  c02
..........................................................................uagaucuacacucauuga..................  4    0  c02
..........................................................................Gagaucuacacucauuga..................  1    1  c02
..........................................................................uaUaucuacacucauuga..................  1    1  c02
..........................................................................uagaucuacacucauugaA.................  1    1  c02

..............................uCaauggguauauuucuaguaacaca......................................................  1    1  c15
...............................Caauggguauauuucuaguaacac.......................................................  1    1  c15
................................aauggguauauuucuagu............................................................  3    0  c15
................................aauggguauauuucuagua...........................................................  16   0  c15
................................aauggguauauuucuaguaa..........................................................  75   0  c15
................................aauggguUuauuucuaguaac.........................................................  1    1  c15
................................aauggguauauuucuaguaGc.........................................................  2    1  c15
................................aaCggguauauuucuaguaac.........................................................  1    1  c15
................................aauggguauauuuGuaguaac.........................................................  1    1  c15
................................Uauggguauauuucuaguaac.........................................................  1    1  c15
................................aaugggAauauuucuaguaac.........................................................  1    1  c15
................................aauggguauauuucuaguaaA.........................................................  1    1  c15
................................aauggguauauuucuaguaac.........................................................  456  0  c15
................................aaugggGauauuucuaguaac.........................................................  1    1  c15
................................aaugggCauauuucuaguaac.........................................................  2    1  c15
................................aGuggguauauuucuaguaac.........................................................  1    1  c15
................................aauggguauauuucuaguaaca........................................................  733  0  c15
................................aauggguauauuucuagAaaca........................................................  1    1  c15
................................aauggguauauuucuaguaUca........................................................  1    1  c15
................................aauggguauauuucuaguaacU........................................................  5    1  c15
................................aaugggGauauuucuaguaaca........................................................  4    1  c15
```

```
                                Star                                  Mature
aagaaagaaguugaaaguaccuacuuguucuuaauggguauauuucuaguaacacagugcauuauuucugugacuagaucuacacucauugaguacguuuaggacaacau
................................aauggguauauuucuaguaacG........................................................   2      1    c15
................................aauggguauauuAcuaguaaca........................................................   1      1    c15
................................aGuggguauauuucuaguaaca........................................................   1      1    c15
................................aaugggCauauuucuaguaaca........................................................   2      1    c15
................................aaugggAauauuucuaguaaca........................................................   1      1    c15
................................Gauggguauauuucuaguaacac.......................................................   1      1    c15
................................aaAggguauauuucuaguaacac.......................................................   1      1    c15
................................aauggAuauauuucuaguaacac.......................................................   1      1    c15
................................aauggguauauuucuaguaacac.......................................................   622    0    c15
................................aauggguauauuucuaguaacaU.......................................................   1      1    c15
................................aauggguauauuucuaguaaGac.......................................................   1      1    c15
................................aaugggGauauuucuaguaacac.......................................................   3      1    c15
................................aauggguauauuucuaguaacaA.......................................................   16     1    c15
................................aaugggCauauuucuaguaacac.......................................................   1      1    c15
................................aauggguauauuucuGguaacac.......................................................   1      1    c15
................................aauggguauaAuucuaguaacac.......................................................   1      1    c15
................................aaugCguauauuucuaguaacac.......................................................   1      1    c15
................................aaAggguauauuucuaguaacaca......................................................   1      1    c15
................................aauggguGuauuucuaguaacaca......................................................   1      1    c15
................................aauggguauauuucuagAaacaca......................................................   1      1    c15
................................aUuggguauauuucuaguaacaca......................................................   1      1    c15
................................aGuggguauauuucuaguaacaca......................................................   2      1    c15
................................aaugggGauauuucuaguaacaca......................................................   5      1    c15
................................aauggCuauauuucuaguaacaca......................................................   1      1    c15
................................aauggguauauuucuaguaacacC......................................................   1      1    c15
................................aauggguauauuucuagCaacaca......................................................   1      1    c15
................................aauggguauauuucuaguaacaUa......................................................   1      1    c15
................................aauggguauauuucuaguaGcaca......................................................   2      1    c15
................................aauggguauauuucuaguaacacG......................................................   2      1    c15
................................aaugUguauauuucuaguaacaca......................................................   1      1    c15
................................aauggguauauuucuaguaacaca......................................................   956    0    c15
................................aaugggCauauuucuaguaacaca......................................................   1      1    c15
................................aaugggAauauuucuaguaacaca......................................................   2      1    c15
................................aauggguauUuuucuaguaacaca......................................................   1      1    c15
................................aauggguauauuucuaguaacacU......................................................   11     1    c15
................................aauggguauauuucuaguaUcaca......................................................   1      1    c15
................................aauggguauauuucuaguaacacaA.....................................................   40     1    c15
................................aauggguauauuucuaguaacacaU.....................................................   1      1    c15
................................aauggguauauuucuaguaacacaAu....................................................   1      1    c15
.................................auggguauauuucuaguaaca........................................................   1      0    c15
.................................auggguauauuucuaguaacacag.....................................................   1      0    c15
..................................uggguauauuucuaguaacacag.....................................................   1      0    c15
...................................ggguauauuucuaguaacac.......................................................   1      0    c15
...................................ggguauauuucuaguaacacaU.....................................................   1      1    c15
....................................................................uCugacuagaucuacacu........................   1      1    c15
....................................................................ugugacuagaucuacacucau.....................   1      0    c15
....................................................................ugugacuagaucuacacucauA....................   1      1    c15
....................................................................ugugacuagaucuacacucauu....................   22     0    c15
....................................................................Ggugacuagaucuacacucauug...................   1      1    c15
....................................................................ugugacuagaucuacacucauug...................   1      0    c15
....................................................................uCugacuagaucuacacucauug...................   5      1    c15
....................................................................ugugacuagaucuacacucauuU...................   2      1    c15
....................................................................uCugacuagaucuacacucauuga..................   135    1    c15
....................................................................uAugacuagaucuacacucauuga..................   6      1    c15
.....................................................................Cugacuagaucuacacucau.....................   9      1    c15
.....................................................................Cugacuagaucuacacucauu....................   7      1    c15
.....................................................................Cugacuagaucuacacucauug...................   87     1    c15
.....................................................................gugacuagaucuacacucauug...................   3      0    c15
.....................................................................gugacuagaucuacacucauuA...................   1      1    c15
.....................................................................Uugacuagaucuacacucauug...................   1      1    c15
.....................................................................gugacuagaucuacacucauuga..................   20     0    c15
.....................................................................gugacuagaucuacacucauGga..................   1      1    c15
.....................................................................gugacuagaucuacGcucauuga..................   1      1    c15
.....................................................................Uugacuagaucuacacucauuga..................   6      1    c15
.....................................................................Cugacuagaucuacacucauuga..................   1251   1    c15
.....................................................................Augacuagaucuacacucauuga..................   4      1    c15
......................................................................ugacuagGucuacacuca......................   1      1    c15
......................................................................ugacuagaucuacacucG......................   3      1    c15
......................................................................ugacuagaucuacacuca......................   772    0    c15
......................................................................ugacuagauUuacacuca......................   1      1    c15
```

```
                                Star                                  Mature
aagaaagaaguugaaaguaccuacuuguucuuaauggguauauuucuaguaacacagugcauuauuucugugacuagaucuacacucauugaguacguuuaggacaacau
......................................................................Cgacuagaucuacacuca......................    1      1    c15
......................................................................ugacuagaucuacacucC......................    2      1    c15
......................................................................Agacuagaucuacacuca......................    1      1    c15
......................................................................ugacuagaucuacacucU......................    1      1    c15
......................................................................ugacuagaucuacacuAau.....................    1      1    c15
......................................................................ugacuagaucuacacucaA.....................    19     1    c15
......................................................................ugacuagCucuacacucau.....................    1      1    c15
......................................................................ugacuagaucuacacucaG.....................    1      1    c15
......................................................................ugacuUgaucuacacucau.....................    1      1    c15
......................................................................ugaGuagaucuacacucau.....................    1      1    c15
......................................................................ugacuGgaucuacacucau.....................    3      1    c15
......................................................................uUacuagaucuacacucau.....................    1      1    c15
......................................................................ugacuagaucuacacucUu.....................    1      1    c15
......................................................................ugacuagaucuacacucau.....................    1723   0    c15
......................................................................ugacuagaucuaUacucau.....................    1      1    c15
......................................................................ugacuagaucuacaUucau.....................    1      1    c15
......................................................................ugacuagaucuacacucGu.....................    1      1    c15
......................................................................ugacuagaucuacacucaC.....................    21     1    c15
......................................................................ugacuaUaucuacacucau.....................    1      1    c15
......................................................................Cgacuagaucuacacucau.....................    2      1    c15
......................................................................uCacuagaucuacacucau.....................    1      1    c15
......................................................................ugacuagaucuacacAcau.....................    2      1    c15
......................................................................ugUcuagaucuacacucau.....................    1      1    c15
......................................................................ugacuagaCcuacacucauu....................    1      1    c15
......................................................................uUacuagaucuacacucauu....................    2      1    c15
......................................................................ugacuagaucuGcacucauu....................    3      1    c15
......................................................................ugacuagaucuacacucauG....................    3      1    c15
......................................................................ugacuagaucCacacucauu....................    1      1    c15
......................................................................ugacuagaucuacacucauC....................    4      1    c15
......................................................................ugacuagaucuacacucaGu....................    2      1    c15
......................................................................ugacuagaucuacacucauu....................    1225   0    c15
......................................................................ugacuagaucuacacucauA....................    3      1    c15
......................................................................ugUcuagaucuacacucauu....................    1      1    c15
......................................................................Cgacuagaucuacacucauu....................    1      1    c15
......................................................................ugacuagaucuacacucUuu....................    2      1    c15
......................................................................ugaGuagaucuacacucauu....................    1      1    c15
......................................................................ugacuagaucuacacucaCu....................    1      1    c15
......................................................................ugacuagaucuaUacucauu....................    1      1    c15
......................................................................ugacuagCucuacacucauug...................    1      1    c15
......................................................................uAacuagaucuacacucauug...................    20     1    c15
......................................................................ugacuagaucuGcacucauug...................    9      1    c15
......................................................................uCacuagaucuacacucauug...................    4      1    c15
......................................................................ugacuagaucuacacucaCug...................    4      1    c15
......................................................................ugacuagaucuacacucauuU...................    17     1    c15
......................................................................ugacuagaucuaUacucauug...................    6      1    c15
......................................................................ugacuagaucuacacCcauug...................    3      1    c15
......................................................................ugacuagaucuacacucaGug...................    1      1    c15
......................................................................ugUcuagaucuacacucauug...................    5      1    c15
......................................................................ugacuagaucuaGacucauug...................    5      1    c15
......................................................................ugacAagaucuacacucauug...................    2      1    c15
......................................................................ugGcuagaucuacacucauug...................    3      1    c15
......................................................................ugacuagaucuacacucauGg...................    22     1    c15
......................................................................ugacuagaucuacacucauuC...................    20     1    c15
......................................................................ugacuagaCcuacacucauug...................    4      1    c15
......................................................................ugacuagUucuacacucauug...................    4      1    c15
......................................................................ugacuagaucuacacucGuug...................    4      1    c15
......................................................................ugacuagaucCacacucauug...................    6      1    c15
......................................................................ugacuagaAcuacacucauug...................    3      1    c15
......................................................................ugacuagaucuacacucauAg...................    9      1    c15
......................................................................ugacuagaucuacacucUuug...................    2      1    c15
......................................................................Agacuagaucuacacucauug...................    16     1    c15
......................................................................ugacuagaucuacUcucauug...................    3      1    c15
......................................................................ugacuagaucuacacAcauug...................    10     1    c15
......................................................................ugacuagaucuacaAucauug...................    1      1    c15
......................................................................ugacuagaucuUcacucauug...................    3      1    c15
......................................................................ugacuCgaucuacacucauug...................    1      1    c15
......................................................................ugacuagaucuaAacucauug...................    3      1    c15
......................................................................ugacuagaucuacaUucauug...................    5      1    c15
......................................................................ugacuagaucuacacGcauug...................    7      1    c15
......................................................................ugacCagaucuacacucauug...................    3      1    c15
```

```
                                Star                                  Mature
aagaaagaaguugaaaguaccuacuuguucuuaauggguauauuucuaguaacacagugcauuauuucugugacuagaucuacacucauugaguacguuuaggacaacau
......................................................................ugacuagGucuacacucauug...................   6       1   c15
......................................................................ugaUuagaucuacacucauug...................   13      1   c15
......................................................................ugacuagaucuacGcucauug...................   1       1   c15
......................................................................ugacuagaucuacacuAauug...................   1       1   c15
......................................................................ugacuagaucuacacucaAug...................   4       1   c15
......................................................................ugacuagaucAacacucauug...................   1       1   c15
......................................................................ugacuagaucuacacucauug...................   21676   0   c15
......................................................................uUacuagaucuacacucauug...................   5       1   c15
......................................................................ugacuagaucuacacucauuA...................   647     1   c15
......................................................................ugaAuagaucuacacucauug...................   4       1   c15
......................................................................ugacuagauAuacacucauug...................   2       1   c15
......................................................................ugacuUgaucuacacucauug...................   9       1   c15
......................................................................ugacuaAaucuacacucauug...................   2       1   c15
......................................................................ugacuaUaucuacacucauug...................   2       1   c15
......................................................................ugacuagaucuacacucauCg...................   16      1   c15
......................................................................ugacuagaucuacacucCuug...................   2       1   c15
......................................................................ugacGagaucuacacucauug...................   2       1   c15
......................................................................ugaGuagaucuacacucauug...................   3       1   c15
......................................................................Cgacuagaucuacacucauug...................   15      1   c15
......................................................................ugacuagaucuacacuUauug...................   9       1   c15
......................................................................ugacuGgaucuacacucauug...................   13      1   c15
......................................................................ugacuagauUuacacucauug...................   5       1   c15
......................................................................ugacuaCaucuacacucauug...................   1       1   c15
......................................................................ugacuagaucGacacucauuga..................   10      1   c15
......................................................................ugacuagaucuaGacucauuga..................   174     1   c15
......................................................................ugacuagaAcuacacucauuga..................   65      1   c15
......................................................................ugCcuagaucuacacucauuga..................   6       1   c15
......................................................................uUacuagaucuacacucauuga..................   188     1   c15
......................................................................ugGcuagaucuacacucauuga..................   81      1   c15
......................................................................ugacuagaucuacacucauuga..................   609823  0   c15
......................................................................ugacuagaucuacacCcauuga..................   65      1   c15
......................................................................ugacuagaucuacaUucauuga..................   192     1   c15
......................................................................ugacuUgaucuacacucauuga..................   129     1   c15
......................................................................ugacuaUaucuacacucauuga..................   70      1   c15
......................................................................ugacuagaucCacacucauuga..................   262     1   c15
......................................................................ugacuagaucuacacucUuuga..................   91      1   c15
......................................................................ugacuCgaucuacacucauuga..................   18      1   c15
......................................................................ugacuagaucuacacucauuUa..................   85      1   c15
......................................................................ugacuGgaucuacacucauuga..................   317     1   c15
......................................................................ugacuagaucuGcacucauuga..................   200     1   c15
......................................................................uCacuagaucuacacucauuga..................   63      1   c15
......................................................................ugacuagaCcuacacucauuga..................   62      1   c15
......................................................................ugacuagaucuacaGucauuga..................   39      1   c15
......................................................................ugacuagaucuacacuAauuga..................   121     1   c15
......................................................................ugacuagaucuacacucaAuga..................   66      1   c15
......................................................................ugacuagaucuacacucauugU..................   2791    1   c15
......................................................................ugacuagaucuacacucauugC..................   455     1   c15
......................................................................ugacuagauUuacacucauuga..................   175     1   c15
......................................................................ugacuagaucuacUcucauuga..................   36      1   c15
......................................................................ugaUuagaucuacacucauuga..................   176     1   c15
......................................................................ugaAuagaucuacacucauuga..................   39      1   c15
......................................................................ugacuagaucuacacucauugG..................   195     1   c15
......................................................................ugacuagaucuacacuGauuga..................   74      1   c15
......................................................................ugacuagaucuaUacucauuga..................   242     1   c15
......................................................................ugacuagaucuacacGcauuga..................   99      1   c15
......................................................................ugUcuagaucuacacucauuga..................   91      1   c15
......................................................................ugaGuagaucuacacucauuga..................   32      1   c15
......................................................................ugacuagaucuacacucaCuga..................   71      1   c15
......................................................................ugacuagaucuacacucauCga..................   271     1   c15
......................................................................ugacuagaucuacacuUauuga..................   241     1   c15
......................................................................ugacuagaucuacacucCuuga..................   9       1   c15
......................................................................ugacuaCaucuacacucauuga..................   16      1   c15
......................................................................Agacuagaucuacacucauuga..................   445     1   c15
......................................................................ugacuagaucuacaAucauuga..................   28      1   c15
......................................................................ugacuagGucuacacucauuga..................   193     1   c15
......................................................................ugacuagaucuacacucauAga..................   317     1   c15
......................................................................ugacuagaucuacacAcauuga..................   112     1   c15
......................................................................ugacuagaucuacacucauuAa..................   1523    1   c15
......................................................................ugacuagaGcuacacucauuga..................   7       1   c15
......................................................................ugacuagCucuacacucauuga..................   48      1   c15
```

Star **Mature**

```
aagaaagaaguugaaaguaccuacuuguucuuaauggguauauuucuaguaacacagugcauuauuucugugacuagaucuacacucauugaguacguuuaggacaacau
......................................................................ugacuagaucuCcacucauuga..................  10     1  c15
......................................................................ugacuaAaucuacacucauuga..................  45     1  c15
......................................................................uAacuagaucuacacucauuga..................  335    1  c15
......................................................................ugacuagaucuacGcucauuga..................  88     1  c15
......................................................................ugacuagaucuacacucGuuga..................  106    1  c15
......................................................................ugacuagaucAacacucauuga..................  64     1  c15
......................................................................ugacCagaucuacacucauuga..................  130    1  c15
......................................................................ugacuagaucuUcacucauuga..................  79     1  c15
......................................................................ugacuagaucuacacucauGga..................  425    1  c15
......................................................................ugacuagaucuaAacucauuga..................  111    1  c15
......................................................................ugacuagaucuacacucaGuga..................  15     1  c15
......................................................................ugacuagaucuacCcucauuga..................  9      1  c15
......................................................................ugacuagUucuacacucauuga..................  158    1  c15
......................................................................ugacuagaucuacacucauuCa..................  50     1  c15
......................................................................ugacGagaucuacacucauuga..................  19     1  c15
......................................................................ugacAagaucuacacucauuga..................  87     1  c15
......................................................................ugacuagauGuacacucauuga..................  27     1  c15
......................................................................Ggacuagaucuacacucauuga..................  63     1  c15
......................................................................ugacuagauAuacacucauuga..................  68     1  c15
......................................................................Cgacuagaucuacacucauuga..................  377    1  c15
......................................................................ugacuagaucuacacucauugaA.................  16642  1  c15
......................................................................ugacuagaucuacacucauugag.................  76     0  c15
......................................................................ugacuagaucuacacucauugaU.................  4152   1  c15
......................................................................ugacuagaucuacacucauugGg.................  2      1  c15
......................................................................ugacuagaucuacacucauugaC.................  466    1  c15
......................................................................ugacuagaucuacacucauugagu................  3      0  c15
......................................................................ugacuagaucuacacucauugagA................  20     1  c15
......................................................................ugacuagaucuacacucauugaUu................  148    1  c15
......................................................................ugacuagaucuacacucauugaAu................  112    1  c15
......................................................................ugacuagaucuacacucauugaCu................  7      1  c15
......................................................................ugacuagaucuacacucauugagG................  1      1  c15
......................................................................ugacuagaucuacacucauugagC................  1      1  c15
......................................................................ugacuagaucuacacucauugagAa...............  8      1  c15
......................................................................ugacuagaucuacacucauugaCua...............  1      1  c15
......................................................................ugacuagaucuacacucauugagCa...............  1      1  c15
......................................................................ugacuagaucuacacucauugaUua...............  3      1  c15
......................................................................ugacuagaucuacacucauugagua...............  3      0  c15
......................................................................ugacuagaucuacacucauugaAua...............  6      1  c15
......................................................................ugacuagaucuacacucauugagGa...............  8      1  c15
......................................................................ugacuagaucuacacucauugaguC...............  1      1  c15
......................................................................ugacuagaucuacacucauugaguac..............  1      0  c15
......................................................................ugacuagaucuacacucauugaguUc..............  1      1  c15
......................................................................ugacuagaucuacacucauugaguaA..............  1      1  c15
......................................................................ugacuagaucuacacucauugagAacg.............  1      1  c15
......................................................................ugacuagaucuacacucauugaguaAg.............  1      1  c15
.......................................................................gacuagaucuacacucaA.....................  1      1  c15
.......................................................................gacuagaucuacacucau.....................  11     0  c15
.......................................................................gacuagaucCacacucauu....................  1      1  c15
.......................................................................gacuagaucuacacucauu....................  1      0  c15
.......................................................................gacuagaucuacacucauuU...................  1      1  c15
.......................................................................gacuagaucuacacucauuA...................  4      1  c15
.......................................................................gacuagaucuacacucauug...................  106    0  c15
.......................................................................gacuagaucuacacCcauug...................  1      1  c15
.......................................................................gacuCgaucuacacucauuga..................  1      1  c15
.......................................................................gacuagauAuacacucauuga..................  1      1  c15
.......................................................................Uacuagaucuacacucauuga..................  15     1  c15
.......................................................................gacuagaucuacacucauuCa..................  1      1  c15
.......................................................................gaUuagaucuacacucauuga..................  1      1  c15
.......................................................................gacuagaucCacacucauuga..................  1      1  c15
.......................................................................gacuagaucuacacucCuuga..................  1      1  c15
.......................................................................gGcuagaucuacacucauuga..................  2      1  c15
.......................................................................gacuagaucuacacucauugG..................  2      1  c15
.......................................................................gacuagaucuacacucauugU..................  10     1  c15
.......................................................................gacuagaucuacacuAauuga..................  1      1  c15
.......................................................................gacuagaucuaGacucauuga..................  1      1  c15
.......................................................................gacuagaucuaUacucauuga..................  2      1  c15
.......................................................................Aacuagaucuacacucauuga..................  1      1  c15
.......................................................................gacuagaucuacacucauCga..................  1      1  c15
.......................................................................gUcuagaucuacacucauuga..................  1      1  c15
.......................................................................gacuagaucuacacucGuuga..................  2      1  c15
```

```
                                Star                                  Mature
aagaaagaaguugaaaguaccuacuuguucuuaauggguauauuucuaguaacacagugcauuauuucugugacuagaucuacacucauugaguacguuuaggacaacau
.......................................................................gacuagaucuacacucaAuga..................    1       1       c15
.......................................................................gacuagaucuacacucauuAa..................    12      1       c15
.......................................................................gacuaAaucuacacucauuga..................    2       1       c15
.......................................................................gacuagaucuacacucauuUa..................    15      1       c15
.......................................................................gacuagaAcuacacucauuga..................    1       1       c15
.......................................................................gacuagaucuacacucauuga..................    2972    0       c15
.......................................................................gacuagaucuacacucauugC..................    4       1       c15
.......................................................................gacuagauUuacacucauuga..................    1       1       c15
.......................................................................gacuagaucuacGcucauuga..................    1       1       c15
.......................................................................gacCagaucuacacucauuga..................    4       1       c15
.......................................................................gacuagaucuacacCcauuga..................    1       1       c15
.......................................................................gacuagaucuacaUucauuga..................    1       1       c15
.......................................................................gacuagaucuacacucauAga..................    1       1       c15
.......................................................................gacuagaucuacacucauugaU.................    16      1       c15
.......................................................................gacuagaucuacacucauugaC.................    6       1       c15
.......................................................................gacuagaucuacacucauugaA.................    86      1       c15
.......................................................................gacuagaucuacacucauugaUu................    2       1       c15
........................................................................aAuagaucuacacucauug...................    1       1       c15
........................................................................acuagaucuacacucauuga..................    33      0       c15
........................................................................acuagaucuacacucauugaA.................    1       1       c15
.........................................................................cuagaucuacacucauug...................    2       0       c15
.........................................................................cuagaucuacacucauuga..................    76      0       c15
.........................................................................cCagaucuacacucauuga..................    1       1       c15
.........................................................................cuagaucuacacucauuAa..................    1       1       c15
.........................................................................cuagaucuacacucauugaA.................    4       1       c15
..........................................................................uagaucuacacucauuga..................    97      0       c15
..........................................................................uagaucuacacGcauuga..................    1       1       c15
..........................................................................Cagaucuacacucauuga..................    1       1       c15
..........................................................................uGgaucuacacucauuga..................    1       1       c15
..........................................................................uagaucuacacucauugaA.................    1       1       c15
...........................................................................agaucuacacucauugaA.................    4       1       c15
```

Provisional ID	:	group10_14597
Score total	:	152136.9
Score for star read(s)	:	3.9
Score for read counts	:	152127.4
Score for mfe	:	1.1
Score for randfold	:	1.6
Score for cons. seed	:	3
Total read count	:	298403
Mature read count	:	297578
Loop read count	:	0
Star read count	:	825

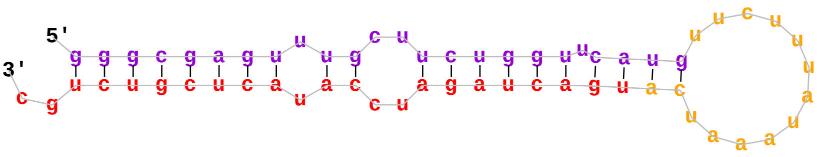

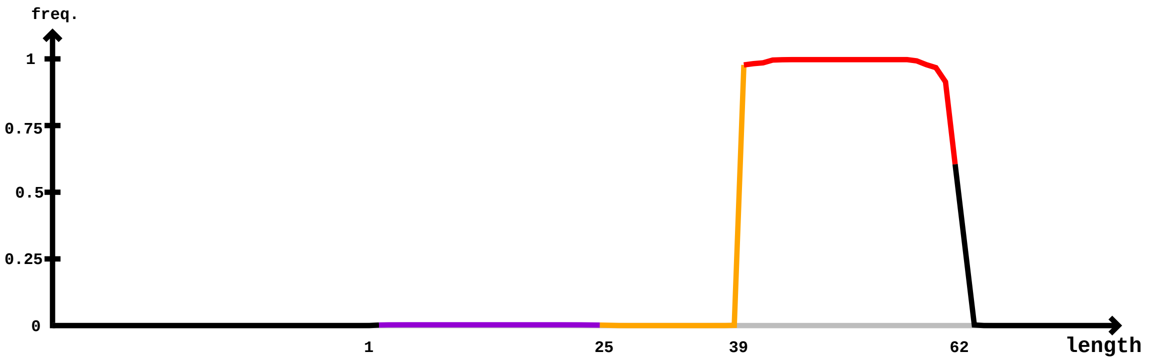

Star　　　　　　　　Mature

```
5'- cuuacuacuauauauaacuugaugauauuugugggcgaguuugcuucugguucauguucuuuauaaaaucaugacuagauccauacucgucugcagccaucaacuuaaauc  -3'  obs
    cuuacuacuauauauaacuugaugauauuugugggcgaguuugcuucugguucauguucuuuauaaaaucaugacuagauccauacucgucugcagccaucaacuuaaauc        exp
    ..............(((.((((((....((((((((((((.((..((((((.((((.............))))))))))..)).)))))))))))).)))))).)))....       reads  mm  sample
    ...............................ugggcgaguuugcuucugguu...........................................................       1      0   c16
    ...............................ugggcgaguuugcuucugguuc..........................................................       1      0   c16
    ...............................ugggcgaguuugcuucugguuca.........................................................       1      0   c16
    ................................gggcgaguuugcuucugguu...........................................................       2      0   c16
    ................................gggcgaguuugcuucugguuc..........................................................       2      0   c16
    ................................gggcgaguuugcuucugguuca.........................................................       3      0   c16
    ................................gggcgaguuugcuucugguucU.........................................................       1      1   c16
    ................................gggcgaguuugcuucugguucau........................................................       9      0   c16
    ................................gggcgaguuugcuucugguucaug.......................................................       8      0   c16
    .................................ggcgaguuugcuucugguuca.........................................................       2      0   c16
    .................................ggcgaguuugcuucugguucau........................................................       2      0   c16
    .................................ggcgaguuugcuucugguucaug.......................................................       14     0   c16
    .................................ggcgaguuugcuucugguucauguucuuu.................................................       1      0   c16
    ..................................gcgaguuugcuucugguucaug.......................................................       1      0   c16
    ...................................cgaguuugcuucugguucau........................................................       1      0   c16
    ...................................cgaguuugcuucugguucaug.......................................................       1      0   c16
    ...................................cgaguuugcuucugguucaugu......................................................       2      0   c16
    ...................................cgaguuugcuucugguucauguu.....................................................       4      0   c16
    ..............................................................Gauaaaucaugacuagauccauacucgucugc.................       1      1   c16
    ....................................................................ucaugacuagauccauacuG.......................       1      1   c16
    ....................................................................ucaugacuagauccauacucgu.....................       1      0   c16
    ....................................................................ucUugacuagauccauacucguc....................       1      1   c16
    ....................................................................Acaugacuagauccauacucgucugc.................       1      1   c16
    .....................................................................caugacuagauccauacucg......................       1      0   c16
    .....................................................................caugacuagauccauacucgu.....................       4      0   c16
    .....................................................................caugacuagauccauacucguc....................       1      0   c16
    ......................................................................augacuagauccauacuc.......................       2      0   c16
    ......................................................................Cugacuagauccauacuc.......................       1      1   c16
    ......................................................................Cugacuagauccauacucg......................       2      1   c16
    ......................................................................Cugacuagauccauacucgu.....................       4      1   c16
    ......................................................................augacuagauccauacucguc....................       1      0   c16
    ......................................................................Cugacuagauccauacucguc....................       2      1   c16
    ......................................................................Cugacuagauccauacucgucu...................       2      1   c16
```

Star **Mature**

cuuacuacuauauauaacuugaugauauuugugggcgaguuugcuucugguucauguucuuuauaaaucaugacuagauccauacucgucugcagccaucaacuuaauc			
....augacuagauccauacucgucu..................	1	0	c16
....augacuagauccauacucguUu..................	1	1	c16
....Uugacuagauccauacucgucug.................	1	1	c16
....augacuagauccauacucgucug.................	8	0	c16
....Cugacuagauccauacucgucug.................	13	1	c16
....augaAuagauccauacucgucugc................	1	1	c16
....Cugacuagauccauacucgucugc................	12	1	c16
....augacuagauccauacucgucugc................	1	0	c16
....augacuagauccauacucgucugU................	1	1	c16
....Uugacuagauccauacucgucugc................	1	1	c16
....augacuagauccauacucgucugcU...............	2	1	c16
....ugacuagauccauacuC......................	1	1	c16
....ugacuagauccauacuU......................	1	1	c16
....ugacuagauccaCacucg.....................	1	1	c16
....ugacuagauccauacuGg.....................	1	1	c16
....uUacuagauccauacucg.....................	1	1	c16
....ugacuagauccauacuUg.....................	1	1	c16
....ugacuagaucAauacucg.....................	1	1	c16
....ugacuagauAcauacucg.....................	3	1	c16
....ugacuagauccauacucA.....................	1	1	c16
....Ggacuagauccauacucg.....................	41	1	c16
....ugCcuagauccauacucg.....................	2	1	c16
....ugacuUgauccauacucg.....................	1	1	c16
....ugaAuagauccauacucg.....................	1	1	c16
....ugacuagauccauaAucg.....................	1	1	c16
....ugacuaAauccauacucg.....................	1	1	c16
....Agacuagauccauacucg.....................	6	1	c16
....ugacuagauccauacucg.....................	594	0	c16
....uCacuagauccauacucg.....................	1	1	c16
....ugacuagauccauacucgC....................	4	1	c16
....ugGcuagauccauacucgu....................	1	1	c16
....ugacuaUauccauacucgu....................	1	1	c16
....ugacuagauccauacuAgu....................	3	1	c16
....ugacuagauccauacucgu....................	2258	0	c16
....ugacuagauccauacucgG....................	3	1	c16
....ugacuagaucAauacucgu....................	3	1	c16
....uCacuagauccauacucgu....................	2	1	c16
....ugacuGgauccauacucgu....................	2	1	c16
....ugacCagauccauacucgu....................	1	1	c16
....ugacuagauccauGcucgu....................	1	1	c16
....ugacuagauccauacucCu....................	3	1	c16
....ugacuagauccauacucgA....................	3	1	c16
....ugaGuagauccauacucgu....................	1	1	c16
....ugacuagauccUuacucgu....................	1	1	c16
....ugacuagauccauacuUgu....................	1	1	c16
....ugacAagauccauacucgu....................	1	1	c16
....ugacuagauccauCcucgu....................	1	1	c16
....ugacuagaucUauacucgu....................	1	1	c16
....ugCcuagauccauacucgu....................	1	1	c16
....ugacuagauccaCacucgu....................	1	1	c16
....uUacuagauccauacucgu....................	1	1	c16
....Ggacuagauccauacucgu....................	211	1	c16
....ugacuagCuccauacucgu....................	3	1	c16
....ugacuagauAcauacucgu....................	3	1	c16
....Cgacuagauccauacucgu....................	1	1	c16
....Agacuagauccauacucgu....................	10	1	c16
....ugacuagauccauaAucgu....................	2	1	c16
....ugaAuagauccauacucguc...................	1	1	c16
....ugacuagauccauacucUuc...................	1	1	c16
....ugacuGgauccauacucguc...................	1	1	c16
....Cgacuagauccauacucguc...................	1	1	c16
....ugacuagauccCuacucguc...................	5	1	c16
....ugacuagGuccauacucguc...................	1	1	c16
....ugacuagauAcauacucguc...................	3	1	c16
....ugacuagauccauacCcguc...................	1	1	c16
....ugacuagauccauacuAguc...................	2	1	c16
....uCacuagauccauacucguc...................	1	1	c16
....ugacuagauccauaUucguc...................	1	1	c16
....ugacuagauccauCcucguc...................	1	1	c16
....ugacuagauccauaGucguc...................	2	1	c16

Star **Mature**

```
cuuacuacuauauauaacuugaugauauuugugggcgaguuugcuucugguucauguucuuuauaaaucaugacuagauccauacucgucugcagccaucaacuuaauc
......................................................................ugCcuagauccauacucguc...................	1	1	c16
......................................................................ugacuagauccauacucguG...................	9	1	c16
......................................................................ugacuagauccauacucguA...................	7	1	c16
......................................................................ugacuagauccauacucAuc...................	2	1	c16
......................................................................Ggacuagauccauacucguc...................	115	1	c16
......................................................................ugacAagauccauacucguc...................	1	1	c16
......................................................................Agacuagauccauacucguc...................	5	1	c16
......................................................................ugacuagauccGuacucguc...................	1	1	c16
......................................................................ugacuagauccaCacucguc...................	1	1	c16
......................................................................ugacuagauccauaAucguc...................	1	1	c16
......................................................................ugacuagCuccauacucguc...................	4	1	c16
......................................................................ugUcuagauccauacucguc...................	1	1	c16
......................................................................ugacCagauccauacucguc...................	1	1	c16
......................................................................ugacuagauccauacucgCc...................	1	1	c16
......................................................................ugacuagaCccauacucguc...................	1	1	c16
......................................................................ugacuagauccauacucCuc...................	1	1	c16
......................................................................ugacuagaAccauacucguc...................	1	1	c16
......................................................................ugacuagauccauacucguU...................	7	1	c16
......................................................................ugacuagauccauacucguc...................	1406	0	c16
......................................................................ugacuagauccauacucAucu..................	2	1	c16
......................................................................ugacuagauccauacuGgucu..................	1	1	c16
......................................................................uCacuagauccauacucgucu..................	6	1	c16
......................................................................ugacuagauccauacucgucG..................	2	1	c16
......................................................................ugacuagauccaAacucgucu..................	1	1	c16
......................................................................ugacuagauccauacAcgucu..................	2	1	c16
......................................................................ugacuagauccauacucgucA..................	1	1	c16
......................................................................ugacuagauccCuacucgucu..................	8	1	c16
......................................................................ugacuagCuccauacucgucu..................	12	1	c16
......................................................................ugacuagauccaGacucgucu..................	1	1	c16
......................................................................ugacuagaucUauacucgucu..................	3	1	c16
......................................................................ugacuagauccauGcucgucu..................	3	1	c16
......................................................................ugacuagauccauacucgucC..................	8	1	c16
......................................................................ugacuagaucAauacucgucu..................	6	1	c16
......................................................................ugacuagauGcauacucgucu..................	1	1	c16
......................................................................ugacuagauccauacucgAcu..................	3	1	c16
......................................................................ugCcuagauccauacucgucu..................	10	1	c16
......................................................................ugacuagaAccauacucgucu..................	3	1	c16
......................................................................ugacuagUuccauacucgucu..................	1	1	c16
......................................................................Ggacuagauccauacucgucu..................	519	1	c16
......................................................................ugacuagauccauacuAgucu..................	9	1	c16
......................................................................ugacuagauccauCcucgucu..................	6	1	c16
......................................................................ugaAuagauccauacucgucu..................	5	1	c16
......................................................................ugacuagauccauacucUucu..................	8	1	c16
......................................................................ugacuagauccauaAucgucu..................	3	1	c16
......................................................................ugacuagaucGauacucgucu..................	1	1	c16
......................................................................ugacuaUauccauacucgucu..................	3	1	c16
......................................................................ugacuagauccauacucCucu..................	9	1	c16
......................................................................uAacuagauccauacucgucu..................	1	1	c16
......................................................................ugacuagauccauUcucgucu..................	3	1	c16
......................................................................ugacuagauccauacucgGcu..................	3	1	c16
......................................................................ugacuagauccauacucguAu..................	2	1	c16
......................................................................ugacAagauccauacucgucu..................	1	1	c16
......................................................................ugacuagaCccauacucgucu..................	2	1	c16
......................................................................Agacuagauccauacucgucu..................	19	1	c16
......................................................................ugacuagauccauacuUgucu..................	4	1	c16
......................................................................ugacuagauccauacucguUu..................	8	1	c16
......................................................................ugacGagauccauacucgucu..................	1	1	c16
......................................................................ugacuagauccauacGcgucu..................	1	1	c16
......................................................................ugacuagauUcauacucgucu..................	4	1	c16
......................................................................ugacuagauccauacucgucu..................	6140	0	c16
......................................................................ugaUuagauccauacucgucu..................	3	1	c16
......................................................................ugacuagauccauaGucgucu..................	1	1	c16
......................................................................Cgacuagauccauacucgucu..................	3	1	c16
......................................................................ugacuagauccGuacucgucu..................	1	1	c16
......................................................................ugacuaAauccauacucgucu..................	1	1	c16
......................................................................ugacCagauccauacucgucu..................	1	1	c16
......................................................................uUacuagauccauacucgucu..................	2	1	c16
......................................................................ugacuagauAcauacucgucu..................	17	1	c16
......................................................................ugacuagauccauaUucgucu..................	1	1	c16
......................................................................ugacuaCauccauacucgucu..................	2	1	c16
```

Star **Mature**

cuuacuacuauauauaacuugaugauauuugugggcgaguuugcuucugguucauguucuuuauaaaucaugacuagauccauacucgucugcagccaucaacuuaauc			
....ugacuagCuccauacucgucug.................	58	1	c16
....ugaUuagauccauacucgucug.................	14	1	c16
....ugacuagauccauGcucgucug.................	6	1	c16
....ugacuagaCccauacucgucug.................	4	1	c16
....ugacuagauUcauacucgucug.................	11	1	c16
....ugCcuagauccauacucgucug.................	34	1	c16
....ugacuagaucAauacucgucug.................	24	1	c16
....ugacuagauccauUcucgucug.................	2	1	c16
....uUacuagauccauacucgucug.................	17	1	c16
....ugacuagauccauacucCucug.................	24	1	c16
....ugacuagauccauacuUgucug.................	9	1	c16
....ugacuagauccauacucgucCg.................	11	1	c16
....ugacuagauccaAacucgucug.................	9	1	c16
....ugacuaAauccauacucgucug.................	1	1	c16
....ugacuagaucUauacucgucug.................	9	1	c16
....Cgacuagauccauacucgucug.................	18	1	c16
....ugacuagauccauCcucgucug.................	24	1	c16
....ugacuagauccauacucgucuC.................	36	1	c16
....ugacCagauccauacucgucug.................	6	1	c16
....ugacuagauccauacucgucAg.................	10	1	c16
....uCacuagauccauacucgucug.................	11	1	c16
....ugaGuagauccauacucgucug.................	3	1	c16
....ugacuagauccauacucUucug.................	19	1	c16
....ugacuagauccauacuAgucug.................	26	1	c16
....ugacuagauccauacucAucug.................	10	1	c16
....ugacuagauccauacucgCcug.................	6	1	c16
....ugacuagauccauacucgAcug.................	3	1	c16
....ugacuagauccauacucgucuA.................	7	1	c16
....ugacuagauccauacucguAug.................	16	1	c16
....ugacuagauGcauacucgucug.................	5	1	c16
....uAacuagauccauacucgucug.................	5	1	c16
....ugacuagaAccauacucgucug.................	7	1	c16
....ugaAuagauccauacucgucug.................	14	1	c16
....ugacGagauccauacucgucug.................	7	1	c16
....ugGcuagauccauacucgucug.................	3	1	c16
....ugacuagUuccauacucgucug.................	2	1	c16
....ugacuagauccCuacucgucug.................	28	1	c16
....ugacuUgauccauacucgucug.................	4	1	c16
....ugacuagauccauacucgucGg.................	2	1	c16
....ugacuagauccauacCcgucug.................	1	1	c16
....Ggacuagauccauacucgucug.................	1790	1	c16
....ugacuagaucGauacucgucug.................	3	1	c16
....ugacuagauccauacAcgucug.................	5	1	c16
....ugacuagauccauacuGgucug.................	1	1	c16
....ugacuagaGccauacucgucug.................	6	1	c16
....ugacuaCauccauacucgucug.................	11	1	c16
....ugacuagGuccauacucgucug.................	1	1	c16
....ugacAagauccauacucgucug.................	10	1	c16
....ugacuaUauccauacucgucug.................	9	1	c16
....ugacuagauccauaGucgucug.................	4	1	c16
....ugacuagauccaGacucgucug.................	6	1	c16
....ugacuagauAcauacucgucug.................	34	1	c16
....ugacuagauccauacucguGug.................	4	1	c16
....ugacuagauccauaAucgucug.................	14	1	c16
....ugacuagauccauacucgucug.................	22443	0	c16
....ugacuagauccauaUucgucug.................	6	1	c16
....ugacuagauccGuacucgucug.................	2	1	c16
....ugUcuagauccauacucgucug.................	4	1	c16
....ugacuagauccauacGcgucug.................	3	1	c16
....ugacuagauccUuacucgucug.................	2	1	c16
....ugacuagauccaCacucgucug.................	8	1	c16
....Agacuagauccauacucgucug.................	71	1	c16
....ugacuGgauccauacucgucug.................	6	1	c16
....ugacuagauccauacucguUug.................	8	1	c16
....ugacuagauccauacucgGcug.................	2	1	c16
....ugacuagauccauacucgucuU.................	79	1	c16
....ugacuagauccauacucgGcugc................	2	1	c16
....ugacuagaucUauacucgucugc................	7	1	c16
....ugacuagaCccauacucgucugc................	4	1	c16
....ugacuagauccauaUucgucugc................	8	1	c16

Star **Mature**

```
cuuacuacuauauauaacuugaugauauuugugggcgaguuugcuucugguucauguucuuuauaaaucaugacuagauccauacucgucugcagccaucaacuuaauc
......................................................................ugaGuagauccauacucgucugc................ 4 1 c16
......................................................................ugacuagGuccauacucgucugc................ 4 1 c16
......................................................................ugacuGgauccauacucgucugc................ 2 1 c16
......................................................................ugacuagauccauacucgucugU................ 2970 1 c16
......................................................................ugacuagauccGuacucgucugc................ 1 1 c16
......................................................................ugacuagauccauacCcgucugc................ 5 1 c16
......................................................................ugacuagauccauacGcgucugc................ 2 1 c16
......................................................................ugacuagauUcauacucgucugc................ 10 1 c16
......................................................................ugUcuagauccauacucgucugc................ 2 1 c16
......................................................................ugacuagauccauacucguAugc................ 22 1 c16
......................................................................ugacuaUauccauacucgucugc................ 10 1 c16
......................................................................uAacuagauccauacucgucugc................ 5 1 c16
......................................................................ugacuagauccaCacucgucugc................ 6 1 c16
......................................................................ugacuagauccCuacucgucugc................ 29 1 c16
......................................................................ugacuagauccauacuCucugc................ 48 1 c16
......................................................................ugacAagauccauacucgucugc................ 5 1 c16
......................................................................ugacuagauccUuacucgucugc................ 4 1 c16
......................................................................ugacCagauccauacucgucugc................ 5 1 c16
......................................................................ugGcuagauccauacucgucugc................ 1 1 c16
......................................................................ugacuagauccauacucgucugA................ 348 1 c16
......................................................................ugacuagauccauacucgucCgc................ 13 1 c16
......................................................................ugacuagauccauUcucgucugc................ 4 1 c16
......................................................................ugacuagauccauacucgCcugc................ 8 1 c16
......................................................................ugacuagCuccauacucgucugc................ 54 1 c16
......................................................................ugacGagauccauacucgucugc................ 2 1 c16
......................................................................Cgacuagauccauacucgucugc................ 22 1 c16
......................................................................ugacuagauccauacucgucuCc................ 20 1 c16
......................................................................ugacuagauccauacucAucugc................ 10 1 c16
......................................................................ugacuagauccauacucgucugc................ 21821 0 c16
......................................................................ugacuagauccauGcucgucugc................ 3 1 c16
......................................................................ugaUuagauccauacucgucugc................ 16 1 c16
......................................................................Agacuagauccauacucgucugc................ 71 1 c16
......................................................................ugacuagauccauacucgucuAc................ 4 1 c16
......................................................................ugacuaAauccauacucgucugc................ 1 1 c16
......................................................................ugacuagauccauaAucgucugc................ 10 1 c16
......................................................................ugaAuagauccauacucgucugc................ 8 1 c16
......................................................................ugCcuagauccauacucgucugc................ 39 1 c16
......................................................................ugacuagauccauaGucgucugc................ 1 1 c16
......................................................................Ggacuagauccauacucgucugc................ 1637 1 c16
......................................................................uCacuagauccauacucgucugc................ 11 1 c16
......................................................................ugacuagauccauacuAgucugc................ 36 1 c16
......................................................................ugacuUgauccauacucgucugc................ 2 1 c16
......................................................................ugacuagaAccauacucgucugc................ 1 1 c16
......................................................................ugacuagauccauacucgucugG................ 74 1 c16
......................................................................ugacuagUuccauacucgucugc................ 1 1 c16
......................................................................ugacuagauccauacucgucGgc................ 9 1 c16
......................................................................ugacuagauccauacucgucuUc................ 9 1 c16
......................................................................ugacuagaGccauacucgucugc................ 6 1 c16
......................................................................ugacuagauccauacAcgucugc................ 11 1 c16
......................................................................ugacuagaucGauacucgucugc................ 9 1 c16
......................................................................ugacuagauccauCcucgucugc................ 23 1 c16
......................................................................ugacuagauccaGacucgucugc................ 5 1 c16
......................................................................ugacuagauccauacucgAcugc................ 7 1 c16
......................................................................ugacuagauccauacucguUugc................ 15 1 c16
......................................................................ugacuagauAcauacucgucugc................ 45 1 c16
......................................................................ugacuagaucAauacucgucugc................ 28 1 c16
......................................................................ugacuaCauccauacucgucugc................ 9 1 c16
......................................................................ugacuagauccauacucgucAgc................ 8 1 c16
......................................................................ugacuagauccaAacucgucugc................ 1 1 c16
......................................................................uUacuagauccauacucgucugc................ 20 1 c16
......................................................................ugacuagauccauacuUgucugc................ 14 1 c16
......................................................................ugacuagauGcauacucgucugc................ 5 1 c16
......................................................................ugacuagauccauacucUucugc................ 24 1 c16
......................................................................ugacuagauccauacucguGugc................ 6 1 c16
......................................................................ugacuagauccauacucgucugca............... 1010 0 c16
......................................................................ugacuagauccauacucgucugAa............... 8 1 c16
......................................................................ugacuagauccauCcucgucugca............... 2 1 c16
......................................................................ugacuagauccCuacucgucugca............... 1 1 c16
......................................................................ugacuagaucUauacucgucugca............... 2 1 c16
......................................................................ugacuagauccaCacucgucugca............... 1 1 c16
```

Star Mature

```
cuuacuacuauauauaacuugaugauauuugugggcgaguuugcuucugguucauguucuuuauaaaucaugacuagauccauacucgucugcagccaucaacuuaauc
......................................................................ugacGagauccauacucgucugca...............   1     1   c16
......................................................................ugacuagauAcauacucgucugca...............   1     1   c16
......................................................................ugacuagauccauacucgGcugca...............   1     1   c16
......................................................................Ggacuagauccauacucgucugca...............   87    1   c16
......................................................................ugacuagauccauacucgucugUa...............   128   1   c16
......................................................................ugacuagauccauacucgucugcU...............   3626  1   c16
......................................................................ugacuagauccauacucgucugcG...............   16    1   c16
......................................................................ugacuagaGccauacucgucugca...............   1     1   c16
......................................................................ugacuaAauccauacucgucugca...............   1     1   c16
......................................................................ugacuagCuccauacucgucugca...............   3     1   c16
......................................................................ugacuagauccauUcucgucugca...............   1     1   c16
......................................................................ugacuagUuccauacucgucugca...............   1     1   c16
......................................................................ugacuaUauccauacucgucugca...............   2     1   c16
......................................................................ugacuagauccauaAucgucugca...............   1     1   c16
......................................................................ugacuUgauccauacucgucugca...............   1     1   c16
......................................................................ugacuagauccaAacucgucugca...............   2     1   c16
......................................................................Agacuagauccauacucgucugca...............   3     1   c16
......................................................................ugacuagauccauacuAgucugca...............   1     1   c16
......................................................................ugacuagauccauacucgucuAca...............   1     1   c16
......................................................................ugacuagauccauacucgucugGa...............   3     1   c16
......................................................................ugacuagauccauacucAucugca...............   1     1   c16
......................................................................ugacuagauccauacucgucugcC...............   81    1   c16
......................................................................ugacuagaucAauacucgucugca...............   1     1   c16
......................................................................Ggacuagauccauacucgucugcag..............   1     1   c16
......................................................................ugacuagauccauacucgucugcaA..............   83    1   c16
......................................................................ugacuagauccauacucgucugcaC..............   8     1   c16
......................................................................ugacuagauccauacucgucugcaU..............   43    1   c16
......................................................................ugacuagauccauacucgucugcag..............   3     0   c16
......................................................................ugacuagauccauacucgucugcUg..............   13    1   c16
......................................................................ugacuagauccauacucgucugcaUc.............   1     1   c16
......................................................................ugacuagauccauacucgucugcagG.............   1     1   c16
......................................................................ugacuagauccauacucgucugcagA.............   1     1   c16
......................................................................ugacuagauccauacucgucugcUgc.............   2     1   c16
......................................................................ugacuagauccauacucgucugcaCcc............   2     1   c16
.......................................................................gacuagauccauacucgu....................   5     0   c16
.......................................................................gGcuagauccauacucguc...................   1     1   c16
.......................................................................gacuagauccauacucguc...................   2     0   c16
.......................................................................gacuagaAccauacucgucu..................   1     1   c16
.......................................................................gacuagauccauacucgucu..................   10    0   c16
.......................................................................gacuagauccauacucgucA..................   1     1   c16
.......................................................................Uacuagauccauacucgucu..................   1     1   c16
.......................................................................Cacuagauccauacucgucug.................   1     1   c16
.......................................................................gacuagauccauacucgucAg.................   1     1   c16
.......................................................................gacuagauGcauacucgucug.................   1     1   c16
.......................................................................gGcuagauccauacucgucug.................   2     1   c16
.......................................................................gacuagauccauacucgucug.................   68    0   c16
.......................................................................gacuagauccauacucCucug.................   1     1   c16
.......................................................................gacuagauccauacucgGcug.................   1     1   c16
.......................................................................gacuagauccauacuAgucug.................   1     1   c16
.......................................................................gCcuagauccauacucgucug.................   1     1   c16
.......................................................................gUcuagauccauacucgucug.................   1     1   c16
.......................................................................Uacuagauccauacucgucug.................   1     1   c16
.......................................................................gacGagauccauacucgucug.................   1     1   c16
.......................................................................gacuaCauccauacucgucug.................   1     1   c16
.......................................................................gUcuagauccauacucgucugc................   2     1   c16
.......................................................................gacuagauccauacucguUugc................   1     1   c16
.......................................................................gacuagauccauaUucgucugc................   1     1   c16
.......................................................................gacuagauccauacucgucugc................   54    0   c16
.......................................................................gGcuagauccauacucgucugc................   1     1   c16
.......................................................................Uacuagauccauacucgucugc................   2     1   c16
.......................................................................gacuagauccauacucgucugU................   1     1   c16
.......................................................................gacuaCauccauacucgucugc................   1     1   c16
.......................................................................gacuagauccauGcucgucugc................   1     1   c16
.......................................................................gacuagauccauacucgucugA................   1     1   c16
.......................................................................gacuagauccauacAcgucugc................   2     1   c16
.......................................................................gacuagauccauacucgucugcU...............   22    1   c16
.......................................................................gacuagauccauacucgucugca...............   2     0   c16
.......................................................................gacuagauccauacucgucugcaU..............   1     1   c16
........................................................................Ccuagauccauacucguc...................   3     1   c16
........................................................................acuagauccauacucgucu..................   4     0   c16
```

Star Mature

```
cuuacuacuauauauaacuugaugauauuugugggcgaguuugcuucugguucauguucuuuauaaaaucaugacuagauccauacucgucugcagccaucaacuuaauc
.........................................................................Ccuagauccauacucgucu..................  2    1  c16
.........................................................................acuUgauccauacucgucu..................  1    1  c16
.........................................................................Ucuagauccauacucgucug.................  1    1  c16
.........................................................................acuagauccauacucgucug.................  14   0  c16
.........................................................................Gcuagauccauacucgucug.................  1    1  c16
.........................................................................aGuagauccauacucgucug.................  1    1  c16
.........................................................................Ccuagauccauacucgucug.................  6    1  c16
.........................................................................acuagauccauacucgucugc................  51   0  c16
.........................................................................acuagaucAauacucgucugc................  2    1  c16
.........................................................................Ccuagauccauacucgucugc................  15   1  c16
.........................................................................aAuagauccauacucgucugc................  1    1  c16
.........................................................................acuagaGccauacucgucugc................  1    1  c16
.........................................................................acuagauccauacucgucugU................  7    1  c16
.........................................................................acuagauAcauacucgucugc................  1    1  c16
.........................................................................acuagauccCuacucgucugc................  1    1  c16
.........................................................................acuagauccauacuAgucugc................  1    1  c16
.........................................................................acuagauccauacucgucugcC...............  5    1  c16
.........................................................................acuagauccauacucgucugcU...............  82   1  c16
.........................................................................acuagaucUauacucgucugca...............  1    1  c16
.........................................................................acuagauccauacucgucugca...............  13   0  c16
.........................................................................acuagauccauacucgucugUa...............  3    1  c16
.........................................................................acuagCuccauacucgucugca...............  1    1  c16
.........................................................................acuagauccauacucgucugcaA..............  2    1  c16
.........................................................................acuagauccauacucgucugcaU..............  1    1  c16
..........................................................................cuagCuccauacucgucu..................  2    1  c16
..........................................................................cuagauccauacucgucu..................  88   0  c16
..........................................................................cuaCauccauacucgucu..................  1    1  c16
..........................................................................cuagaucUauacucgucu..................  1    1  c16
..........................................................................cuagCuccauacucgucug.................  6    1  c16
..........................................................................cuagauccauacucCucug.................  1    1  c16
..........................................................................cuagaucAauacucgucug.................  1    1  c16
..........................................................................Auagauccauacucgucug.................  1    1  c16
..........................................................................cuagauccauacucgucuU.................  6    1  c16
..........................................................................cuagauccauCcucgucug.................  2    1  c16
..........................................................................cuagauccauaAucgucug.................  1    1  c16
..........................................................................cuagauccauGcucgucug.................  1    1  c16
..........................................................................cuagaucGauacucgucug.................  1    1  c16
..........................................................................cuagauccauacuUgucug.................  1    1  c16
..........................................................................cuagauccauacucgucuC.................  2    1  c16
..........................................................................cCagauccauacucgucug.................  1    1  c16
..........................................................................Uuagauccauacucgucug.................  1    1  c16
..........................................................................cuagauccauacucgucuA.................  1    1  c16
..........................................................................cuagauccauacucgucug.................  519  0  c16
..........................................................................cuagauccaGacucgucug.................  1    1  c16
..........................................................................cuagauccCuacucgucug.................  3    1  c16
..........................................................................cuagauccauacuAgucugc................  1    1  c16
..........................................................................cuagauAcauacucgucugc................  3    1  c16
..........................................................................cuagCuccauacucgucugc................  11   1  c16
..........................................................................Uuagauccauacucgucugc................  1    1  c16
..........................................................................cuaCauccauacucgucugc................  1    1  c16
..........................................................................cuagaCccauacucgucugc................  1    1  c16
..........................................................................cuagauccCuacucgucugc................  1    1  c16
..........................................................................cuagauccauacucgucuCc................  2    1  c16
..........................................................................cuagauccauacucgucuUc................  1    1  c16
..........................................................................cuagauccauacucgucugG................  1    1  c16
..........................................................................cuagauccauacucCucugc................  1    1  c16
..........................................................................cuagauccauacucgucugU................  35   1  c16
..........................................................................cuagauccauacucgucugc................  562  0  c16
..........................................................................cuGgauccauacucgucugc................  2    1  c16
..........................................................................cuagauccauaAucgucugc................  1    1  c16
..........................................................................cuagaAccauacucgucugc................  1    1  c16
..........................................................................cuagUuccauacucgucugc................  1    1  c16
..........................................................................cuagauccauacucgucugA................  5    1  c16
..........................................................................cuagauccauacAcgucugc................  2    1  c16
..........................................................................cuagauccauacucgucugca...............  23   0  c16
..........................................................................cuagauccauacucgucugcC...............  2    1  c16
..........................................................................cuagauccauacucgucugUa...............  2    1  c16
..........................................................................cuagauccauacucgucugcU...............  44   1  c16
..........................................................................cuagauccauacucgucugcaA..............  2    1  c16
..........................................................................cuagauccauacucgucugcaU..............  2    1  c16
```

Star **Mature**

```
cuuacuacuauauauaacuugaugauauuugugggcgaguuugcuucugguucauguucuuuauaaaucaugacuagauccauacucgucugcagccaucaacuuaauc
..........................................................................Gagauccauacucgucug.................  6    1  c16
..........................................................................uagauccaGacucgucug.................  1    1  c16
..........................................................................uagauccauCcucgucug.................  1    1  c16
..........................................................................uagCuccauacucgucug.................  1    1  c16
..........................................................................uagauccauacucgucug.................  45   0  c16
..........................................................................uaUauccauacucgucug.................  1    1  c16
..........................................................................uagauccauacucgucugc................  50   0  c16
..........................................................................uagauccauacucgucugU................  6    1  c16
..........................................................................uUgauccauacucgucugc................  1    1  c16
..........................................................................Gagauccauacucgucugc................  5    1  c16
..........................................................................uagauccauacucgucugca...............  3    0  c16
..........................................................................uagauccauacucgucugcU...............  6    1  c16
..........................................................................uagauccauacucgucugcaA..............  1    1  c16
...........................................................................agauccauacucgucugU................  3    1  c16
...........................................................................aCauccauacucgucugc................  1    1  c16
...........................................................................agauccauaAucgucugc................  1    1  c16
...........................................................................agauccauacucgucugc................  28   0  c16
...........................................................................agauccauacucgucugca...............  2    0  c16
...........................................................................agauccauacucgucugcU...............  6    1  c16
............................................................................gauccauacucgucugcU...............  2    1  c16
.............................................................................auccauacucgucugcaA..............  1    1  c16

................................gggcgaguuugcuucugguucau......................................................  1    0  c04
................................gggcgaguuugcuucugguucaug.....................................................  1    0  c04
.................................ggcgaguuugcuucugguucaug.....................................................  5    0  c04
...................................................................ucCugacuagauccauacucgucug.................  1    1  c04
.....................................................................Cugacuagauccauacuc......................  1    1  c04
.....................................................................Cugacuagauccauacucguc...................  1    1  c04
.....................................................................Cugacuagauccauacucgucug.................  5    1  c04
.....................................................................augacuagauccauacucgucug.................  1    0  c04
.....................................................................Cugacuagauccauacucgucugc................  1    1  c04
......................................................................ugacuagauccauacucU.....................  3    1  c04
......................................................................ugacuagauccauacucA.....................  1    1  c04
......................................................................Ggacuagauccauacucg.....................  10   1  c04
......................................................................ugacuagauccauacucC.....................  2    1  c04
......................................................................ugacuagauccauacucg.....................  207  0  c04
......................................................................ugacuaUauccauacucg.....................  2    1  c04
......................................................................ugacuagaucUauacucgu....................  1    1  c04
......................................................................ugacuagauccauacucgu....................  418  0  c04
......................................................................Agacuagauccauacucgu....................  1    1  c04
......................................................................ugacGagauccauacucgu....................  1    1  c04
......................................................................ugacuagauccauacucCu....................  1    1  c04
......................................................................ugacuagauccauacucgG....................  3    1  c04
......................................................................ugacuagauccaGacucgu....................  1    1  c04
......................................................................ugacuagaucAauacucgu....................  1    1  c04
......................................................................ugUcuagauccauacucgu....................  1    1  c04
......................................................................ugacuagauccauacuGgu....................  1    1  c04
......................................................................Ggacuagauccauacucgu....................  31   1  c04
......................................................................ugacuagauccauacucgA....................  4    1  c04
......................................................................Cgacuagauccauacucgu....................  2    1  c04
......................................................................uUacuagauccauacucgu....................  1    1  c04
......................................................................ugacuagauccauacucgC....................  2    1  c04
......................................................................ugacCagauccauacucgu....................  1    1  c04
......................................................................ugacuagauccaAacucgu....................  1    1  c04
......................................................................ugacuagauccauacucguG...................  5    1  c04
......................................................................ugacuagauccauacucgAc...................  1    1  c04
......................................................................ugacuaUauccauacucguc...................  1    1  c04
......................................................................ugacuagauccCuacucguc...................  1    1  c04
......................................................................ugacAagauccauacucguc...................  1    1  c04
......................................................................ugacuagauccauacucgGc...................  3    1  c04
......................................................................ugacuagauccauacucguA...................  4    1  c04
......................................................................Ggacuagauccauacucguc...................  15   1  c04
......................................................................ugacuagauccauacucguU...................  5    1  c04
......................................................................ugacCagauccauacucguc...................  1    1  c04
......................................................................ugacuagauAcauacucguc...................  1    1  c04
......................................................................ugacuagaucUauacucguc...................  1    1  c04
......................................................................ugacuGgauccauacucguc...................  1    1  c04
......................................................................ugacuagauccaCacucguc...................  1    1  c04
......................................................................Cgacuagauccauacucguc...................  1    1  c04
......................................................................ugacuagauccauacucguc...................  419  0  c04
```

Star **Mature**

cuuacuacuauauauaacuugaugauauuugugggcgaguuugcuucugguucauguucuuuauaaaucaugacuagauccauacucgucugcagccaucaacuuaaauc

....ugacGagauccauacucguc....................	1	1	c04
....ugacuagGuccauacucgucu...................	1	1	c04
....ugacuagauccaGacucgucu...................	1	1	c04
....ugacuagauccauacucgucA...................	6	1	c04
....ugacuagauccauacucguUu...................	1	1	c04
....ugacuagauccauacucgucu...................	555	0	c04
....ugacuagauccCuacucgucu...................	1	1	c04
....ugCcuagauccauacucgucu...................	1	1	c04
....ugacuagauccauacucCucu...................	1	1	c04
....uUacuagauccauacucgucu...................	2	1	c04
....ugacuagaCccauacucgucu...................	1	1	c04
....ugacuagauccauacucUucu...................	1	1	c04
....Ggacuagauccauacucgucu...................	24	1	c04
....ugacuagauccUuacucgucu...................	1	1	c04
....ugacuagauccauacucgucG...................	3	1	c04
....ugacuagauccauacucgucC...................	3	1	c04
....Agacuagauccauacucgucu...................	3	1	c04
....ugacuagaAccauacucgucug..................	1	1	c04
....ugacuagauccauacucgucCg..................	2	1	c04
....ugacuagauccauacucguUug..................	4	1	c04
....ugacuagauccauacucgucuC..................	11	1	c04
....ugacuagauccauacAcgucug..................	2	1	c04
....ugacuagauccauacucgucuA..................	8	1	c04
....ugacuagauccauacuUgucug..................	3	1	c04
....ugacuagauccauacucAucug..................	1	1	c04
....ugacuagaucGauacucgucug..................	1	1	c04
....ugacuagauccauacGcgucug..................	1	1	c04
....ugacuagauccauUcucgucug..................	1	1	c04
....ugacuaCauccauacucgucug..................	1	1	c04
....ugacuagaucAauacucgucug..................	5	1	c04
....ugacuagauccauacucgCcug..................	1	1	c04
....ugacuagauccauaAucgucug..................	2	1	c04
....ugGcuagauccauacucgucug..................	1	1	c04
....ugacuagauUcauacucgucug..................	1	1	c04
....ugCcuagauccauacucgucug..................	11	1	c04
....ugacuagCuccauacucgucug..................	11	1	c04
....ugaAuagauccauacucgucug..................	4	1	c04
....ugacuagauAcauacucgucug..................	10	1	c04
....Agacuagauccauacucgucug..................	14	1	c04
....ugacuagauccauacucgucGg..................	1	1	c04
....Ggacuagauccauacucgucug..................	256	1	c04
....ugacuagauccauacucgucug..................	5052	0	c04
....uCacuagauccauacucgucug..................	1	1	c04
....ugacuagauccauacucguAug..................	2	1	c04
....ugacuagauccauacucgucuU..................	19	1	c04
....Cgacuagauccauacucgucug..................	1	1	c04
....ugacuagauccGuacucgucug..................	2	1	c04
....ugacuaUauccauacucgucug..................	2	1	c04
....ugacuagauccauacucCucug..................	6	1	c04
....ugacuaAauccauacucgucug..................	3	1	c04
....ugacuagaucUauacucgucug..................	1	1	c04
....ugacuagauccauacuAgucug..................	3	1	c04
....ugaUuagauccauacucgucug..................	4	1	c04
....ugacuGgauccauacucgucug..................	2	1	c04
....ugacuagauccauacucgucAg..................	2	1	c04
....ugacuagauccauCcucgucug..................	1	1	c04
....ugUcuagauccauacucgucug..................	1	1	c04
....uAacuagauccauacucgucug..................	3	1	c04
....ugacuagauccauacucUucug..................	4	1	c04
....ugacuagauccaGacucgucug..................	2	1	c04
....ugacuagauccCuacucgucug..................	4	1	c04
....ugacuagauccauacucgAcugc.................	2	1	c04
....Agacuagauccauacucgucugc.................	12	1	c04
....Ggacuagauccauacucgucugc.................	137	1	c04
....ugacuagauccauaAucgucugc.................	2	1	c04
....uCacuagauccauacucgucugc.................	8	1	c04
....ugacuagauccauacucgucuCc.................	3	1	c04
....ugacuaUauccauacucgucugc.................	3	1	c04
....ugacuagaucUauacucgucugc.................	1	1	c04
....ugacuaCauccauacucgucugc.................	1	1	c04

```
                           Star                                  Mature
cuuacuacuauauauaacuugaugauauuugugggcgaguuugcuucugguucauguucuuuauaaaucaugacuagauccauacucgucugcagccaucaacuuaauc
......................................................................ugCcuagauccauacucgucugc................  4     1  c04
......................................................................ugacuagauccUuacucgucugc................  1     1  c04
......................................................................ugacuagauccauacucgucugA................  95    1  c04
......................................................................ugacuagauccauacCcgucugc................  1     1  c04
......................................................................ugacuagCuccauacucgucugc................  8     1  c04
......................................................................ugacuagauccauaUucgucugc................  1     1  c04
......................................................................ugacuagauccauacucguGugc................  1     1  c04
......................................................................ugacuagauccauGcucgucugc................  1     1  c04
......................................................................ugacuCgauccauacucgucugc................  1     1  c04
......................................................................ugacuagauccauacucgCcugc................  2     1  c04
......................................................................ugacuagaucAauacucgucugc................  6     1  c04
......................................................................ugacuagaGccauacucgucugc................  1     1  c04
......................................................................ugacuagauccauacucUucugc................  3     1  c04
......................................................................ugacuagauccauacucgucGgc................  2     1  c04
......................................................................ugacuagauccauacucgucuAc................  3     1  c04
......................................................................ugacuagauccauacucgucuUc................  1     1  c04
......................................................................ugacuagauccaGacucgucugc................  2     1  c04
......................................................................ugacuagauccauacucgucugc................  2794  0  c04
......................................................................ugacuUgauccauacucgucugc................  1     1  c04
......................................................................ugacuagauccauacucgucugU................  274   1  c04
......................................................................ugacuagauAcauacucgucugc................  2     1  c04
......................................................................ugacuagauccauCcucgucugc................  3     1  c04
......................................................................ugGcuagauccauacucgucugc................  1     1  c04
......................................................................uUacuagauccauacucgucugc................  1     1  c04
......................................................................ugacuagauccauacucgucugG................  26    1  c04
......................................................................ugacuagauUcauacucgucugc................  1     1  c04
......................................................................ugacuagauccCuacucgucugc................  1     1  c04
......................................................................ugacuagauccauacucguAugc................  1     1  c04
......................................................................ugacuagauccauacAcgucugc................  1     1  c04
......................................................................ugacuagauccauacuAgucugc................  2     1  c04
......................................................................ugacuagauccauacucCucugc................  3     1  c04
......................................................................uAacuagauccauacucgucugc................  1     1  c04
......................................................................ugacuagauccauacucguUugc................  6     1  c04
......................................................................ugacuagauccauacucgucugcU...............  168   1  c04
......................................................................ugacGagauccauacucgucugca...............  1     1  c04
......................................................................ugacuagauccauacucgucugca...............  665   0  c04
......................................................................ugacuagauAcauacucgucugca...............  1     1  c04
......................................................................ugCcuagauccauacucgucugca...............  1     1  c04
......................................................................ugacuagaucUauacucgucugca...............  1     1  c04
......................................................................ugacuagauccauaUucgucugca...............  1     1  c04
......................................................................ugaGuagauccauacucgucugca...............  1     1  c04
......................................................................ugacuagauccauacuUgucugca...............  1     1  c04
......................................................................ugacuagauccauacuAgucugca...............  1     1  c04
......................................................................ugacuagauccauaAucgucugca...............  1     1  c04
......................................................................ugacuagaucGauacucgucugca...............  1     1  c04
......................................................................ugacuagauccauacucgCcugca...............  1     1  c04
......................................................................ugacuagauccauacucgucugGa...............  1     1  c04
......................................................................ugacuagauccauacucgucugUa...............  33    1  c04
......................................................................Ggacuagauccauacucgucugca...............  38    1  c04
......................................................................Agacuagauccauacucgucugca...............  3     1  c04
......................................................................ugacuagCuccauacucgucugca...............  1     1  c04
......................................................................ugacuagauccauacucgucugAa...............  14    1  c04
......................................................................ugacuagauccauacucgucugcG...............  2     1  c04
......................................................................ugacuagauccCuacucgucugca...............  1     1  c04
......................................................................ugaUuagauccauacucgucugca...............  1     1  c04
......................................................................ugacuagaAccauacucgucugca...............  1     1  c04
......................................................................ugacuagauccauacucgucugcC...............  11    1  c04
......................................................................ugacuagauccauacucgucugcag..............  6     0  c04
......................................................................ugacuagauccauacucgucugcaU..............  13    1  c04
......................................................................ugacuagauccauacucgucugcaA..............  317   1  c04
......................................................................ugacuagauccauacucgucugAag..............  1     1  c04
......................................................................ugacuagauccauacucgucugcaC..............  4     1  c04
......................................................................ugacuagauccauacucgucugcaCc.............  1     1  c04
......................................................................ugacuagauccauacucgucugcagU.............  3     1  c04
......................................................................ugacuagauccauacucgucugcagc.............  1     0  c04
......................................................................ugacuagauccauacucgucugcaCcc............  1     1  c04
.......................................................................gacuagauccauacucguc...................  2     0  c04
.......................................................................gCcuagauccauacucguc...................  1     1  c04
.......................................................................gacuagauccauacucgucu..................  3     0  c04
.......................................................................gaGuagauccauacucgucu..................  1     1  c04
```

```
                              Star                                    Mature
cuuacuacuauauauaacuugaugauauuugugggcgaguuugcuucugguucauguucuuuauaaaucaugacuagauccauacucgucugcagccaucaacuuaauc
.......................................................................Uacuagauccauacucgucug.................	1	1	c04
.......................................................................gacuagauccauacAcgucug.................	1	1	c04
.......................................................................gCcuagauccauacucgucug.................	2	1	c04
.......................................................................gacuagauccauacucgucug.................	12	0	c04
.......................................................................gacuagauccauacucgucugc................	14	0	c04
.......................................................................gacuagauccauacucgucugcU...............	1	1	c04
.......................................................................gacuagauccauacucgucugcC...............	1	1	c04
.......................................................................gacuagauccauacucgucugcaA..............	1	1	c04
........................................................................aUuagauccauacucgucu..................	1	1	c04
........................................................................aGuagauccauacucgucu..................	1	1	c04
........................................................................aUuagauccauacucgucug.................	2	1	c04
........................................................................Ccuagauccauacucgucug.................	1	1	c04
........................................................................Ucuagauccauacucgucug.................	1	1	c04
........................................................................acuagauccauacucgucug.................	2	0	c04
........................................................................acuagauccauacucgucugc................	4	0	c04
........................................................................acuagauccauacucgucugU................	1	1	c04
........................................................................acuagauccauacucgucugcC...............	3	1	c04
........................................................................acuagauccauacucgucugUa...............	1	1	c04
........................................................................acuagauccauacucgucugca...............	3	0	c04
........................................................................acuagauccauacucgucugcU...............	6	1	c04
........................................................................acuagauccauacucgucugcaA..............	3	1	c04
.........................................................................cuagauccauCcucgucu..................	1	1	c04
.........................................................................cuagauccauacucgucu..................	15	0	c04
.........................................................................cuagauccauacucgucuC.................	1	1	c04
.........................................................................cuagauccauacucgucuA.................	1	1	c04
.........................................................................cuagauccauacucgucug.................	120	0	c04
.........................................................................cuagauccauacucguUug.................	1	1	c04
.........................................................................cuagauccauacuAgucug.................	2	1	c04
.........................................................................cuagauccauacucgucugU................	3	1	c04
.........................................................................cuagauccauacucgucugG................	1	1	c04
.........................................................................cuagauccauaGucgucugc................	1	1	c04
.........................................................................cuagauccauacucgucugc................	64	0	c04
.........................................................................cuagauAcauacucgucugca...............	1	1	c04
.........................................................................cuagauccauacucgucugcU...............	1	1	c04
.........................................................................cuagauccauacucgucugca...............	13	0	c04
.........................................................................cuagauccauacucgucugcaA..............	4	1	c04
.........................................................................cuagauccauacucgucugcagU.............	1	1	c04
..........................................................................uagauccauacucgucug.................	24	0	c04
..........................................................................Gagauccauacucgucug.................	2	1	c04
..........................................................................uagauccauacucgucugc................	19	0	c04
..........................................................................uagauccauacucgucugU................	3	1	c04
..........................................................................uagauccauacucgucugca...............	6	0	c04
..........................................................................uagauccauacucgucugcU...............	1	1	c04
..........................................................................uagauccauacucgucugcaA..............	4	1	c04
...........................................................................agauccauacucgucugc................	10	0	c04
...........................................................................agauccauacucgucugU................	2	1	c04
...........................................................................agauccauacucgucugcaA..............	1	1	c04
............................................................................gauccauacucgucugca...............	4	0	c04
.............................................................................auccauacucgucugcaA..............	1	1	c04

..............................gugggcgaguuugcuucugguucaug.....................................................	1	0	c09
................................gggcgaguuugcuucugguu.........................................................	2	0	c09
................................gggcgaguuugcuucugguuc........................................................	2	0	c09
................................gggcgaguuugcuucugguuca.......................................................	4	0	c09
................................gggcgaguuugcuucugguucau......................................................	3	0	c09
................................gggcgaguuugcuucugguucaug.....................................................	6	0	c09
................................gggcgaguuugcuucugguucaugu....................................................	2	0	c09
.................................ggcgaguuugcuucugguuca.......................................................	1	0	c09
.................................ggcgaguuugcuucugguucau......................................................	2	0	c09
.................................ggcgaguuugcuucugguucaug.....................................................	6	0	c09
..................................gcgaguuugcuucugguucaug.....................................................	1	0	c09
...................................cgaguuugcuucugguucauguu...................................................	2	0	c09
...................................cgaguuugcuucugguucauguuU..................................................	3	1	c09
............................................................uuauaaaucaugacuagauccauacucgucug.................	1	0	c09
.................................................................aaucaugacuagauccauacu.......................	1	0	c09
..................................................................aucaugacuagauccauacucgucug.................	1	0	c09
..................................................................aucaugacuagauccauacucgucugc................	1	0	c09
....................................................................caugacuagauccauacuc......................	2	0	c09
....................................................................caugacuagauccauacucgu....................	1	0	c09
.....................................................................augacuagauccauacuc......................	1	0	c09
```

Star **Mature**

```
cuuacuacuauauauaacuugaugauauuugugggcgaguuugcuucugguucauguucuuuauaaaucaugacuagauccauacucgucugcagccaucaacuuaauc
.....................................................................augacuagauccauacucgu....................    2       0       c09
.....................................................................Cugacuagauccauacucgu....................    1       1       c09
.....................................................................Cugacuagauccauacucguc...................    1       1       c09
.....................................................................augacuagauccauacucgucu..................    1       0       c09
.....................................................................Cugacuagauccauacucgucug.................    6       1       c09
.....................................................................augacuagauccauacucgucug.................    3       0       c09
.....................................................................Cugacuagauccauacucgucugc................    3       1       c09
.....................................................................augacuagauccauacucgucugc................    1       0       c09
......................................................................ugacuagauccCuacucg.....................    1       1       c09
......................................................................ugacuagauccauacuGg.....................    1       1       c09
......................................................................ugacuagauccauacucg.....................    256     0       c09
......................................................................Ggacuagauccauacucg.....................    16      1       c09
......................................................................ugacuagauccaCacucg.....................    1       1       c09
......................................................................ugacuagauUcauacucg.....................    1       1       c09
......................................................................ugacuagauccauacucU.....................    3       1       c09
......................................................................Agacuagauccauacucg.....................    1       1       c09
......................................................................ugacuagauccauCcucgu....................    1       1       c09
......................................................................ugacuagauAcauacucgu....................    2       1       c09
......................................................................Ggacuagauccauacucgu....................    41      1       c09
......................................................................ugacuagaucUauacucgu....................    1       1       c09
......................................................................ugacuagCuccauacucgu....................    1       1       c09
......................................................................ugacuagauccGuacucgu....................    1       1       c09
......................................................................ugacuagauccauacuAgu....................    1       1       c09
......................................................................ugacuagauccauacucgu....................    523     0       c09
......................................................................ugacuaUauccauacucgu....................    1       1       c09
......................................................................ugacuagauccauacucgG....................    2       1       c09
......................................................................ugacuagaCccauacucgu....................    1       1       c09
......................................................................ugacuagauccauacucUu....................    1       1       c09
......................................................................Agacuagauccauacucgu....................    1       1       c09
......................................................................uUacuagauccauacucgu....................    1       1       c09
......................................................................Agacuagauccauacucguc...................    2       1       c09
......................................................................ugacAagauccauacucguc...................    1       1       c09
......................................................................ugacuagauccauacucguc...................    472     0       c09
......................................................................ugacuagauccauacuAguc...................    1       1       c09
......................................................................ugacuagauUcauacucguc...................    1       1       c09
......................................................................Ggacuagauccauacucguc...................    31      1       c09
......................................................................ugacuagauccauacucguG...................    2       1       c09
......................................................................ugacuagauccauacucguU...................    7       1       c09
......................................................................ugacuagauccauCcucguc...................    1       1       c09
......................................................................ugacuagauccauacucguA...................    3       1       c09
......................................................................ugacuagauccauacucguAu..................    2       1       c09
......................................................................ugacuagauccauUcucgucu..................    1       1       c09
......................................................................Cgacuagauccauacucgucu..................    1       1       c09
......................................................................ugacAagauccauacucgucu..................    3       1       c09
......................................................................ugacuagaucUauacucgucu..................    1       1       c09
......................................................................ugacuagauccauacucAucu..................    1       1       c09
......................................................................ugacuagauccaCacucgucu..................    2       1       c09
......................................................................uCacuagauccauacucgucu..................    1       1       c09
......................................................................Ggacuagauccauacucgucu..................    106     1       c09
......................................................................ugacuagauccaAacucgucu..................    3       1       c09
......................................................................ugacuagauccauacucguUu..................    2       1       c09
......................................................................ugacuagauccGuacucgucu..................    2       1       c09
......................................................................ugacuagaCccauacucgucu..................    1       1       c09
......................................................................ugacuagauccauCcucgucu..................    1       1       c09
......................................................................ugacuagauccauacAcgucu..................    1       1       c09
......................................................................ugacuagaucAauacucgucu..................    2       1       c09
......................................................................ugacuagauccauacGcgucu..................    1       1       c09
......................................................................uUacuagauccauacucgucu..................    1       1       c09
......................................................................ugacuagauccaGacucgucu..................    1       1       c09
......................................................................ugacuaCauccauacucgucu..................    1       1       c09
......................................................................ugacuagauccauacucgucG..................    2       1       c09
......................................................................uAacuagauccauacucgucu..................    2       1       c09
......................................................................ugacuagauccauacucgucu..................    1625    0       c09
......................................................................ugacuagaucGauacucgucu..................    2       1       c09
......................................................................ugacuagCuccauacucgucu..................    3       1       c09
......................................................................ugacuagauccCuacucgucu..................    2       1       c09
......................................................................ugacuagauAcauacucgucu..................    1       1       c09
......................................................................ugaUuagauccauacucgucu..................    1       1       c09
......................................................................ugCcuagauccauacucgucu..................    4       1       c09
......................................................................ugacuagauUcauacucgucu..................    1       1       c09
```

	Star	Mature			
cuuacuacuauauauaacuugaugauauuugugggcgaguuugcuucugguucauguucuuuauaaaucaugacuagauccauacucgucugcagccaucaacuuaauc					
....ugacuagauccauaAucgucu..................			2	1	c09
....ugacuagauccauacucUucu..................			2	1	c09
....ugacuagauccauacucgucC..................			3	1	c09
....ugacuagauccauacucgAcu..................			3	1	c09
....Agacuagauccauacucgucu..................			14	1	c09
....ugacuagauccauacuAgucu..................			3	1	c09
....ugacuagauccauacucguAug.................			4	1	c09
....ugacuagauccauacucgCcug.................			3	1	c09
....ugacuagaCccauacucgucug.................			1	1	c09
....ugacuagaucUauacucgucug.................			3	1	c09
....ugacuagauccauacucgucug.................			3904	0	c09
....ugacuaCauccauacucgucug.................			1	1	c09
....ugUcuagauccauacucgucug.................			2	1	c09
....ugCcuagauccauacucgucug.................			3	1	c09
....ugacuagauccauacCcgucug.................			2	1	c09
....ugacuaAauccauacucgucug.................			3	1	c09
....ugacuagauccauacucgucuC.................			15	1	c09
....ugacuagauccauacuAgucug.................			3	1	c09
....ugacuagauccCuacucgucug.................			1	1	c09
....ugacCagauccauacucgucug.................			2	1	c09
....Cgacuagauccauacucgucug.................			1	1	c09
....ugacuagauccauCcucgucug.................			2	1	c09
....Agacuagauccauacucgucug.................			16	1	c09
....Ggacuagauccauacucgucug.................			229	1	c09
....ugacuagaAccauacucgucug.................			2	1	c09
....ugacuagaucAauacucgucug.................			3	1	c09
....ugacuagauccauaUucgucug.................			5	1	c09
....ugacuagauccauacucgucAg.................			2	1	c09
....uUacuagauccauacucgucug.................			1	1	c09
....ugacuagUuccauacucgucug.................			1	1	c09
....ugaAuagauccauacucgucug.................			1	1	c09
....ugacAagauccauacucgucug.................			2	1	c09
....ugacuagauUcauacucgucug.................			1	1	c09
....uAacuagauccauacucgucug.................			2	1	c09
....ugacuagauccauacucguUug.................			1	1	c09
....ugacuagauccauacucUucug.................			6	1	c09
....ugacuagauccauaAucgucug.................			1	1	c09
....ugacuagauccaAacucgucug.................			1	1	c09
....ugacuagauccauacucgucCg.................			1	1	c09
....ugacuagauccauacucAucug.................			3	1	c09
....uCacuagauccauacucgucug.................			1	1	c09
....ugacuagCuccauacucgucug.................			7	1	c09
....ugacuaUauccauacucgucug.................			1	1	c09
....ugacuagauccauacuUgucug.................			5	1	c09
....ugacuGgauccauacucgucug.................			1	1	c09
....ugaUuagauccauacucgucug.................			1	1	c09
....ugGcuagauccauacucgucug.................			1	1	c09
....ugacuagauccauacucCucug.................			8	1	c09
....ugacuagauAcauacucgucug.................			4	1	c09
....ugacuagauccauacucgucuU.................			22	1	c09
....ugacuagauccUuacucgucug.................			1	1	c09
....ugacuagauccauacucgucuA.................			3	1	c09
....ugCcuagauccauacucgucugc................			7	1	c09
....ugaUuagauccauacucgucugc................			4	1	c09
....ugacuagaucAauacucgucugc................			2	1	c09
....ugaAuagauccauacucgucugc................			1	1	c09
....ugacAagauccauacucgucugc................			1	1	c09
....ugacuagauccauacucgucAgc................			3	1	c09
....Agacuagauccauacucgucugc................			17	1	c09
....ugacuagauccCuacucgucugc................			6	1	c09
....uCacuagauccauacucgucugc................			1	1	c09
....ugacuaUauccauacucgucugc................			1	1	c09
....Ggacuagauccauacucgucugc................			227	1	c09
....ugUcuagauccauacucgucugc................			1	1	c09
....ugacuagauccauacucgucugG................			26	1	c09
....ugGcuagauccauacucgucugc................			1	1	c09
....ugacuagGuccauacucgucugc................			1	1	c09
....ugacuagauccauacucCucugc................			4	1	c09
....ugacuagauccauacucguUugc................			7	1	c09
....ugacuaCauccauacucgucugc................			1	1	c09

Star Mature

cuuacuacuauauauaacuugaugauauuugugggcgaguuugcuucugguucauguucuuuauaaaucaugacuagauccauacucgucugcagccaucaacuuaauc			
....ugacuagauccauacucgucugA................	82	1	c09
....ugacuagauccauacucgucuUc................	2	1	c09
....ugacuagauccauacucgucuCc................	6	1	c09
....ugacuagauccauacuGgucugc................	1	1	c09
....ugacuagauccauUcucgucugc................	1	1	c09
....ugacuagauccauacucAucugc................	3	1	c09
....ugacuagaCccauacucgucugc................	1	1	c09
....ugacuagauccauacucgAcugc................	1	1	c09
....ugacuagauccauacuAgucugc................	6	1	c09
....ugacuagauccauaAucgucugc................	1	1	c09
....Cgacuagauccauacucgucugc................	7	1	c09
....ugacuagauccauacucgCcugc................	1	1	c09
....ugacCagauccauacucgucugc................	2	1	c09
....ugacuagCuccauacucgucugc................	6	1	c09
....uUacuagauccauacucgucugc................	1	1	c09
....ugacuagauAcauacucgucugc................	7	1	c09
....ugacuagauccauacucgucuAc................	1	1	c09
....ugacuagauccauCcucgucugc................	1	1	c09
....ugacuagauccauacCcgucugc................	1	1	c09
....ugacuagauccauacucgucGgc................	1	1	c09
....ugacuagauccauacucguAugc................	2	1	c09
....ugacuagauUcauacucgucugc................	3	1	c09
....ugacuagauccauacuUgucugc................	1	1	c09
....ugacuagauccauacucgucugc................	3767	0	c09
....ugacuagaucUauacucgucugc................	2	1	c09
....ugacuagaucGauacucgucugc................	1	1	c09
....ugacuagauccaCacucgucugc................	1	1	c09
....ugacuGgauccauacucgucugc................	1	1	c09
....ugacuagauccaAacucgucugc................	1	1	c09
....ugacuagauccauacucgucugU................	428	1	c09
....ugacuCgauccauacucgucugc................	1	1	c09
....ugacuagauccauaUucgucugc................	1	1	c09
....ugacuagauccauacucgucugUa...............	20	1	c09
....ugacuagauccauacucgucugcC...............	9	1	c09
....ugacuagauAcauacucgucugca...............	1	1	c09
....ugacuagauccauacucgucugAa...............	6	1	c09
....ugacuagauccauacucgucugcU...............	448	1	c09
....ugacuagauccauacucgucugcG...............	1	1	c09
....Agacuagauccauacucgucugca...............	2	1	c09
....ugacuagauccauacucgucugca...............	119	0	c09
....Ggacuagauccauacucgucugca...............	5	1	c09
....ugacuagaucUauacucgucugca...............	2	1	c09
....ugacuagauccauacucgucugcUg..............	5	1	c09
....ugacuagauccauacucgucugcaU..............	3	1	c09
....ugacuagauccauacucgucugcaA..............	2	1	c09
....ugacuagauccauacucgucugcaC..............	1	1	c09
....ugacuagauccauacucgucugAagc.............	1	1	c09
....ugacuagauccauacucgucugcagG.............	1	1	c09
....ugacuagauccauacucgucugcUgc.............	1	1	c09
....gacuagauccCuacucgu....................	1	1	c09
....gCcuagauccauacucguc...................	1	1	c09
....gUcuagauccauacucgucu..................	1	1	c09
....gacuagauccauacucgucu..................	6	0	c09
....gacuagauccauacucgucug.................	9	0	c09
....gacuagauccauacucgucugc................	17	0	c09
....gacuagauccauacucgucugU................	1	1	c09
....gGcuagauccauacucgucugc................	1	1	c09
....gacuagauAcauacucgucugc................	1	1	c09
....gacuagauccauacucgucugcU...............	1	1	c09
....gacuagauccauacucgucugcaU..............	1	1	c09
....Ccuagauccauacucguc...................	1	1	c09
....acuagauccauacucguc...................	3	0	c09
....acuagauccauacucgucu..................	5	0	c09
....acuagauccauacucgucug.................	5	0	c09
....aGuagauccauacucgucug.................	1	1	c09
....Ccuagauccauacucgucug.................	2	1	c09
....acuagauccauacucgucugc................	31	0	c09
....Ccuagauccauacucgucugc................	5	1	c09
....acuagauccauacucgucugU................	3	1	c09
....aUuagauccauacucgucugc................	1	1	c09

```
                                Star                                    Mature
cuuacuacuauauauaacuugaugauauuugugggcgaguuugcuucugguucauguucuuuauaaaucaugacuagauccauacucgucugcagccaucaacuuaauc
........................................................................acuagauccauacucgCcugca...............    1     1     c09
........................................................................acuagauccauacucgucugca...............    9     0     c09
........................................................................acuagCuccauacucgucugca...............    1     1     c09
........................................................................acuagauccauacucgucugcC...............    5     1     c09
........................................................................acuagauccauacucgucugcU...............    27    1     c09
........................................................................acuagauccauacucgucugcaA..............    1     1     c09
.........................................................................cuagauccauacucCucu..................    1     1     c09
.........................................................................cuagauccauacucgucu..................    83    0     c09
.........................................................................cuagaAccauacucgucu..................    1     1     c09
.........................................................................cuagauccauacAcgucu..................    1     1     c09
.........................................................................cuagauccCuacucgucu..................    2     1     c09
.........................................................................cuagauccauacucgucA..................    1     1     c09
.........................................................................cuagaucAauacucgucug.................    2     1     c09
.........................................................................cuagauccauacucgucuC.................    2     1     c09
.........................................................................cuagauccauacucgucug.................    364   0     c09
.........................................................................cuagauccauCcucgucug.................    1     1     c09
.........................................................................cuagCuccauacucgucug.................    2     1     c09
.........................................................................cuagauccauacuAgucug.................    1     1     c09
.........................................................................cuagauccauacuUgucug.................    1     1     c09
.........................................................................cuagauccauacucCucug.................    1     1     c09
.........................................................................cuagauccauacucgucuU.................    6     1     c09
.........................................................................cuagauccauacucgGcugc................    1     1     c09
.........................................................................Uuagauccauacucgucugc................    3     1     c09
.........................................................................cuagauccauacucgucugc................    289   0     c09
.........................................................................cuagauAcauacucgucugc................    1     1     c09
.........................................................................cuagauccauacucCucugc................    1     1     c09
.........................................................................cuagauccauacucgucuCc................    1     1     c09
.........................................................................cuagauccauacucgucugU................    32    1     c09
.........................................................................Auagauccauacucgucugc................    1     1     c09
.........................................................................cuagauccauacucgucugG................    1     1     c09
.........................................................................cuagCuccauacucgucugc................    1     1     c09
.........................................................................cuagauccauacucgucugA................    3     1     c09
.........................................................................cuagauccauacucgucugca...............    8     0     c09
.........................................................................cuagauccauacucgucugcC..............    4     1     c09
.........................................................................cuagauccauacucgucugcU..............    24    1     c09
..........................................................................uagauccauacucgucug.................    42    0     c09
..........................................................................Gagauccauacucgucug.................    4     1     c09
..........................................................................uGgauccauacucgucug.................    1     1     c09
..........................................................................uagauccauacucgucugU................    5     1     c09
..........................................................................Gagauccauacucgucugc................    3     1     c09
..........................................................................uagauccauacucgucugc................    33    0     c09
..........................................................................Aagauccauacucgucugc................    1     1     c09
..........................................................................uagauccauacAcgucugc................    1     1     c09
..........................................................................uagauccauacucgucugca...............    1     0     c09
..........................................................................uagauccauacucgucugUa...............    2     1     c09
..........................................................................uagauccauacucgucugcU...............    3     1     c09
...........................................................................aUauccauacucgucugc................    2     1     c09
...........................................................................agauccauacucgucugc................    30    0     c09
...........................................................................agauccauacucgucugU................    4     1     c09
...........................................................................agauccauacucgucugca...............    1     0     c09
...........................................................................agauccauacucgucugcG...............    1     1     c09
...........................................................................agauccauacucgucugcU...............    2     1     c09
............................................................................gauccauacucgucugcG...............    1     1     c09
............................................................................gauccauacucgucugcU...............    1     1     c09

.................................ggcgaguuugcuucugguucaug.....................................................    2     0     c02
...................................cgaguuugcuucugguucaug.....................................................    1     0     c02
...................................cgaguuugcuucugguucaugu....................................................    3     0     c02
...................................cgaguuugcuucugguucauguu...................................................    4     0     c02
...................................................................ucaugacuagauccauacu.......................    1     0     c02
...................................................................ucaugacuagauccauacucg.....................    2     0     c02
....................................................................caugacuagauccauacucgu....................    2     0     c02
....................................................................cUugacuagauccauacucgu....................    1     1     c02
....................................................................cUugacuagauccauacucgucug.................    1     1     c02
....................................................................cCugacuagauccauacucgucugc................    1     1     c02
.....................................................................augacuagauccauacucgu....................    1     0     c02
.....................................................................Cugacuagauccauacucgucug.................    2     1     c02
......................................................................ugacuagaucUauacucg.....................    1     1     c02
......................................................................Ggacuagauccauacucg.....................    16    1     c02
......................................................................ugacuagauccauacucC.....................    1     1     c02
```

```
                                   Star                                    Mature
cuuacuacuauauauaacuugaugauauuugugggcgaguuugcuucugguucauguucuuuauaaaucaugacuagauccauacucgucugcagccaucaacuuaauc
......................................................................ugacuagauccauacucg.....................    104     0     c02
......................................................................ugacuagaucAauacucg.....................    1       1     c02
......................................................................ugacuagauccauacucgA....................    2       1     c02
......................................................................ugacuagauccauaAucgu....................    2       1     c02
......................................................................ugacuagauccauacucUu....................    1       1     c02
......................................................................ugacuagauUcauacucgu....................    1       1     c02
......................................................................ugacuagauccauacucgu....................    491     0     c02
......................................................................ugacuagauccauCcucgu....................    1       1     c02
......................................................................ugacuagaucAauacucgu....................    1       1     c02
......................................................................Cgacuagauccauacucgu....................    1       1     c02
......................................................................Agacuagauccauacucgu....................    2       1     c02
......................................................................ugacuagauccGuacucgu....................    1       1     c02
......................................................................ugacuagaGccauacucgu....................    1       1     c02
......................................................................ugacuagCuccauacucgu....................    1       1     c02
......................................................................Ggacuagauccauacucgu....................    42      1     c02
......................................................................uCacuagauccauacucgu....................    2       1     c02
......................................................................ugacuagCuccauacucguc...................    1       1     c02
......................................................................ugacuagauccauacucguc...................    182     0     c02
......................................................................Ggacuagauccauacucguc...................    23      1     c02
......................................................................ugacuagauccauacucguA...................    1       1     c02
......................................................................ugacuagauccauacucguU...................    3       1     c02
......................................................................ugacuagauccauacucgAc...................    1       1     c02
......................................................................ugacuagauccauacucAuc...................    1       1     c02
......................................................................ugacuaUauccauacucguc...................    1       1     c02
......................................................................ugacuagauAcauacucgucu..................    2       1     c02
......................................................................ugacuagaAccauacucgucu..................    1       1     c02
......................................................................ugacuagauccauacucCucu..................    4       1     c02
......................................................................uCacuagauccauacucgucu..................    1       1     c02
......................................................................ugacuagauccauacuAgucu..................    2       1     c02
......................................................................ugGcuagauccauacucgucu..................    1       1     c02
......................................................................ugacuagauccauacCcgucu..................    1       1     c02
......................................................................ugacuagauccUuacucgucu..................    1       1     c02
......................................................................ugacuagCuccauacucgucu..................    3       1     c02
......................................................................ugacuagauccauacAcgucu..................    1       1     c02
......................................................................ugacuaAauccauacucgucu..................    1       1     c02
......................................................................ugacuaUauccauacucgucu..................    1       1     c02
......................................................................Cgacuagauccauacucgucu..................    2       1     c02
......................................................................ugacuagauccCuacucgucu..................    3       1     c02
......................................................................ugacGagauccauacucgucu..................    1       1     c02
......................................................................ugacuagauccaGacucgucu..................    2       1     c02
......................................................................ugacuagauccauacucUucu..................    2       1     c02
......................................................................ugacuagauccauacucguUu..................    2       1     c02
......................................................................ugacuagauccauCcucgucu..................    5       1     c02
......................................................................ugacuagauccauaAucgucu..................    1       1     c02
......................................................................ugCcuagauccauacucgucu..................    1       1     c02
......................................................................Agacuagauccauacucgucu..................    6       1     c02
......................................................................Ggacuagauccauacucgucu..................    127     1     c02
......................................................................ugacuagauccaCacucgucu..................    1       1     c02
......................................................................ugacuagauccaAacucgucu..................    1       1     c02
......................................................................ugacuagauccauacucgucA..................    2       1     c02
......................................................................ugacuGgauccauacucgucu..................    2       1     c02
......................................................................ugacuagauccauacucgucu..................    1733    0     c02
......................................................................ugacuagauccauacucgucG..................    2       1     c02
......................................................................ugacuaCauccauacucgucu..................    1       1     c02
......................................................................ugaAuagauccauacucgucu..................    2       1     c02
......................................................................ugacuagGuccauacucgucu..................    1       1     c02
......................................................................ugacuagaucAauacucgucu..................    2       1     c02
......................................................................ugacuagauccauacucgucC..................    1       1     c02
......................................................................ugacuagauccauacucAucu..................    3       1     c02
......................................................................ugacuagauccauacucgucuA.................    1       1     c02
......................................................................ugacuagauAcauacucgucug.................    3       1     c02
......................................................................ugacuaCauccauacucgucug.................    3       1     c02
......................................................................ugacuagauccauacucgucuU.................    5       1     c02
......................................................................ugGcuagauccauacucgucug.................    1       1     c02
......................................................................ugacuaUauccauacucgucug.................    1       1     c02
......................................................................ugaAuagauccauacucgucug.................    1       1     c02
......................................................................ugacuagauccauacuAgucug.................    4       1     c02
......................................................................Cgacuagauccauacucgucug.................    2       1     c02
......................................................................ugacuagauccauacucCucug.................    1       1     c02
......................................................................ugacuagauccauaAucgucug.................    2       1     c02
```

Star **Mature**

cuuacuacuauauauaacuugaugauauuugugggcgaguuugcuucugguucauguucuuuauaaaucaugacuagauccauacucgucugcagccaucaacuuaaauc

....uCacuagauccauacucgucug..................	1	1	c02
....ugacuagauccauacucAucug..................	3	1	c02
....ugacuagauGcauacucgucug..................	1	1	c02
....ugacuagaAccauacucgucug..................	2	1	c02
....ugacuagauccauacucgGcug..................	1	1	c02
....ugacuagauccauacCcgucug..................	4	1	c02
....Agacuagauccauacucgucug..................	20	1	c02
....ugacuagauccauacucguUug..................	1	1	c02
....ugaUuagauccauacucgucug..................	2	1	c02
....ugacuGgauccauacucgucug..................	1	1	c02
....ugacuagauccauacucgucug..................	2640	0	c02
....ugacuagauccauacucguAug..................	1	1	c02
....ugacGagauccauacucgucug..................	1	1	c02
....ugacuagauccauacucUucug..................	3	1	c02
....ugacuagauUcauacucgucug..................	2	1	c02
....ugacuagauccauacucgucCg..................	1	1	c02
....ugacuagaucAauacucgucug..................	1	1	c02
....ugCcuagauccauacucgucug..................	2	1	c02
....ugacCagauccauacucgucug..................	2	1	c02
....ugacuagCuccauacucgucug..................	6	1	c02
....ugacAagauccauacucgucug..................	2	1	c02
....uUacuagauccauacucgucug..................	2	1	c02
....ugacuagauccauacuUgucug..................	3	1	c02
....ugacuagauccauacucgucuC..................	4	1	c02
....Ggacuagauccauacucgucug..................	222	1	c02
....ugacuagauccCuacucgucug..................	4	1	c02
....ugacuagaucAauacucgucugc.................	2	1	c02
....ugacuagauccauacuUgucugc.................	1	1	c02
....ugacuagauccauacuAgucugc.................	2	1	c02
....ugacuagauccauacucUucugc.................	12	1	c02
....ugacuagauccauacucgucugU.................	473	1	c02
....ugacuagauccauacucgucugc.................	1261	0	c02
....ugacuaCauccauacucgucugc.................	1	1	c02
....ugacuagauccaAacucgucugc.................	3	1	c02
....ugacuagauccCuacucgucugc.................	1	1	c02
....Agacuagauccauacucgucugc.................	3	1	c02
....ugacuagauccauaUucgucugc.................	3	1	c02
....ugacuagauccauacucgCcugc.................	1	1	c02
....Cgacuagauccauacucgucugc.................	1	1	c02
....ugaGuagauccauacucgucugc.................	2	1	c02
....ugacuagauccauacucgucugG.................	3	1	c02
....ugGcuagauccauacucgucugc.................	1	1	c02
....ugacuagaucUauacucgucugc.................	1	1	c02
....ugacuagauccauacucgucugA.................	53	1	c02
....ugacuagauccauacucAucugc.................	1	1	c02
....ugacuagauccauaAucgucugc.................	1	1	c02
....ugacuagauccauacucgAcugc.................	1	1	c02
....ugacuagauccauacucgucuCc.................	2	1	c02
....ugCcuagauccauacucgucugc.................	3	1	c02
....ugacuagUuccauacucgucugc.................	1	1	c02
....ugacAagauccauacucgucugc.................	1	1	c02
....ugacuagauccauacucguUugc.................	2	1	c02
....ugacuagauccauacucgucAgc.................	1	1	c02
....ugacuagCuccauacucgucugc.................	2	1	c02
....ugacuagauccauacucgGcugc.................	1	1	c02
....ugacuagauAcauacucgucugc.................	3	1	c02
....ugacuagauccauacucgucGgc.................	1	1	c02
....ugacuagauccauCcucgucugc.................	1	1	c02
....ugacuagauccauacucCucugc.................	4	1	c02
....ugacuagauccauacucgucCgc.................	1	1	c02
....ugacuCgauccauacucgucugc.................	1	1	c02
....ugacGagauccauacucgucugc.................	1	1	c02
....Ggacuagauccauacucgucugc.................	110	1	c02
....ugacuagauccauacucgucugUa................	24	1	c02
....ugacuagauccauacucgucugcU................	440	1	c02
....ugacuagauccauacucgucugca................	62	0	c02
....ugacuagauccauacucgucugAa................	2	1	c02
....ugacuagauccauacucgucugcC................	6	1	c02
....ugacuagauccauacucgucuAca................	1	1	c02
....Ggacuagauccauacucgucugca................	5	1	c02

Star **Mature**

cuuacuacuauauauaacuugaugauauuugugggcgaguuugcuucugguucauguucuuuauaaaucaugacuagauccauacucgucugcagccaucaacuuaauc			
....ugacuagauccauacucgucugcG...............	2	1	c02
....ugacuagauccauacucgucugcUg..............	2	1	c02
....ugacuagauccauacucgucugcaA..............	1	1	c02
....gacuagauccauacucgu....................	1	0	c02
....gacuagauccauacucguc...................	1	0	c02
....gacuagauccauacucgucu..................	7	0	c02
....gacuagauccauacucgucA..................	1	1	c02
....gacuagauccauacucgucug.................	6	0	c02
....Aacuagauccauacucgucug.................	1	1	c02
....gacuagauccauacucgucugc................	1	0	c02
....gUcuagauccauacucgucugc................	1	1	c02
....gacuagauccauacucgucugcU...............	1	1	c02
....acuagauccauacucguc...................	1	0	c02
....Ccuagauccauacucgucu..................	1	1	c02
....acuagauccauacucgucu..................	3	0	c02
....acuagauccauacucgucug.................	4	0	c02
....Ccuagauccauacucgucug.................	1	1	c02
....acuagauccauacucgAcugc................	1	1	c02
....Ccuagauccauacucgucugc................	9	1	c02
....acuagauccauacucgucugU................	3	1	c02
....acuagauccauacucgucugc................	46	0	c02
....acuagauccauacucgucugca...............	11	0	c02
....acuagauAcauacucgucugca...............	1	1	c02
....acuagauccauacucgucugcC...............	1	1	c02
....Ccuagauccauacucgucugca...............	3	1	c02
....acuaUauccauacucgucugca...............	1	1	c02
....acuagauccauacucgucugcU...............	58	1	c02
....acuagauccauacucgucugUa...............	6	1	c02
....acuagauccauacucgucugcG...............	2	1	c02
....cuagauAcauacucgucu..................	1	1	c02
....cuagCuccauacucgucu..................	1	1	c02
....cuagauccUuacucgucu..................	1	1	c02
....cuagauccauacucgucu..................	76	0	c02
....cuaCauccauacucgucug.................	1	1	c02
....cuagauccauacuUgucug.................	1	1	c02
....cuagCuccauacucgucug.................	2	1	c02
....cuagauAcauacucgucug.................	1	1	c02
....cAagauccauacucgucug.................	1	1	c02
....cuagauccauacucgucuU.................	3	1	c02
....cuagauccauacucgucuA.................	1	1	c02
....cuagauccauacucgGcug.................	1	1	c02
....cuagauccauacucgucug.................	214	0	c02
....cuagaucAauacucgucugc................	1	1	c02
....cuagauccauacucgucAgc................	1	1	c02
....cuagauccCuacucgucugc................	1	1	c02
....Uuagauccauacucgucugc................	1	1	c02
....cuagauAcauacucgucugc................	2	1	c02
....cuagauccauacucgucuUc................	1	1	c02
....cuagGuccauacucgucugc................	1	1	c02
....cuagauccauacucgucugA................	4	1	c02
....cuagCuccauacucgucugc................	2	1	c02
....cuagauccauacAcgucugc................	2	1	c02
....cuagauccauacucgucugc................	269	0	c02
....cuagauUcauacucgucugc................	1	1	c02
....cuagauccauacucgucugU................	14	1	c02
....cuagauccauacucgucugca...............	5	0	c02
....cuagauccauacucgucugcC...............	7	1	c02
....cuagauccauacucgucugUa...............	1	1	c02
....cuagauccauacucgucugcU...............	27	1	c02
....Gagauccauacucgucug.................	2	1	c02
....uagauccauacucgucuC.................	1	1	c02
....uagauccauacucgucug.................	15	0	c02
....Gagauccauacucgucugc................	1	1	c02
....uagauccauacucguAugc................	1	1	c02
....uagauccauacucgucugc................	16	0	c02
....uagauccauacucgucugcC...............	1	1	c02
....uagauccauacucgucugcU...............	1	1	c02
....agauccauacucgucugc................	7	0	c02
....Cgauccauacucgucugc................	1	1	c02
....gauccauacucgucugcU...............	3	1	c02

Star Mature

```
cuuacuacuauauauaacuugaugauauuugugggcgaguuugcuucugguucauguucuuuauaaaucaugacuagauccauacucgucugcagccaucaacuuaauc

..........uauauaacuugaugauauuugug............................................................................ 1 0 c15
...........auauaacuugaugauauuugu............................................................................. 1 0 c15
...........auauaacuugaugauauuugug............................................................................ 5 0 c15
...........auauaacuugaugauauuuguA............................................................................ 1 1 c15
.............auaacuugaugauauuugug............................................................................ 1 0 c15
...............................ugggcgaguuugcuucugguuc........................................................ 3 0 c15
...............................ugggcgaguuugcuucugguuca....................................................... 6 0 c15
...............................ugggcgagGuugcuucugguuca....................................................... 1 1 c15
...............................ugggcgaguuugcuucugguucau...................................................... 2 0 c15
...............................Cgggcgaguuugcuucugguucaug..................................................... 1 1 c15
................................gggcgaguuugcuucuggu.......................................................... 1 0 c15
................................gggcgaguuugcuucugguu......................................................... 3 0 c15
................................gggcgaguuugcuucugguuc........................................................ 13 0 c15
................................gggcgaguuugcuucugguuU........................................................ 5 1 c15
................................gggcgaguuugcuucugguuca....................................................... 65 0 c15
................................gggcgaguuugcuucugguucC....................................................... 1 1 c15
................................gggcgaguuugcuucugguucU....................................................... 4 1 c15
................................gggcgaguuugcuucugguucau...................................................... 66 0 c15
................................gggcgaguuugcuucugguucUu...................................................... 2 1 c15
................................gggcgaguuugcuucugguucaC...................................................... 2 1 c15
................................gggcgaguuugcuucugguucauU..................................................... 11 1 c15
................................gggcgaguuugcuucugguCcaug..................................................... 1 1 c15
................................gggcgaguuugcuucugguucauA..................................................... 15 1 c15
................................gggcgaguuugcuucuCguucaug..................................................... 1 1 c15
................................gggcgaguuugcuucugguGcaug..................................................... 2 1 c15
................................gggcgaguuugUuucugguucaug..................................................... 1 1 c15
................................Aggcgaguuugcuucugguucaug..................................................... 1 1 c15
................................gggcgaguuugcuucugguucauC..................................................... 4 1 c15
................................gAgcgaguuugcuucugguucaug..................................................... 1 1 c15
................................Uggcgaguuugcuucugguucaug..................................................... 5 1 c15
................................gggcgUguuugcuucugguucaug..................................................... 2 1 c15
................................gggcgaguuugcuucuggCucaug..................................................... 1 1 c15
................................ggAcgaguuugcuucugguucaug..................................................... 1 1 c15
................................gggcgaguuugcuucugguucaug..................................................... 301 0 c15
................................gggcgGguuugcuucugguucaugu.................................................... 1 1 c15
................................gggcgaguuugcuucugguucaugA.................................................... 2 1 c15
................................gggcgaguuugcuucugguucaugu.................................................... 10 0 c15
................................gggcgaguuugcuucugguucauguu................................................... 1 0 c15
.................................ggcgaguuugcuucugguuU........................................................ 1 1 c15
.................................ggcgaguuugcuucugguuc........................................................ 2 0 c15
.................................ggcgaguuugcuucugguucU....................................................... 2 1 c15
.................................ggcgaguuugcuucugguuca....................................................... 28 0 c15
.................................ggcgaguuugcuucugguucaC...................................................... 1 1 c15
.................................ggcgaguuugcuucugguucau...................................................... 25 0 c15
.................................Agcgaguuugcuucugguucaug..................................................... 1 1 c15
.................................Ugcgaguuugcuucugguucaug..................................................... 1 1 c15
.................................ggcgaguuugcuucugguucaug..................................................... 99 0 c15
.................................ggcgaguuugcuucugguucauA..................................................... 5 1 c15
.................................ggcgaguuugcuucugguucauC..................................................... 1 1 c15
.................................ggcgaguuugcuucugguucauguU................................................... 1 1 c15
.................................ggcgaguuugcuucugguucauguucu................................................. 1 0 c15
..................................gcgaguuugcuucugguucau...................................................... 1 0 c15
..................................gcgaguuugcuucugguucaug..................................................... 8 0 c15
...................................cgaguuugcuucugguuc........................................................ 1 0 c15
...................................cgaguuugcuucugguuca....................................................... 1 0 c15
...................................cgaguuugcuucugguucaug..................................................... 1 0 c15
...................................cgaguuugcuucugguucaugu.................................................... 1 0 c15
...................................cgaguuugcuucugguucauguU................................................... 1 1 c15
........................................................uucuuuauaaaucaugacuagauccaua......................... 1 0 c15
...................................................................ucaugacuagauccauacucgu.................... 1 0 c15
....................................................................caugacuagauccauacucguc................... 1 0 c15
....................................................................caugacuagauccauacucgucu.................. 1 0 c15
....................................................................Uaugacuagauccauacucgucu.................. 1 1 c15
....................................................................caugacuagauccauacucguUu.................. 1 1 c15
....................................................................Gaugacuagauccauacucgucug................. 1 1 c15
....................................................................Uaugacuagauccauacucgucugc................ 1 1 c15
.....................................................................augacuagauccauacucgu.................... 1 0 c15
.....................................................................Cugacuagauccauacucgucu.................. 6 1 c15
.....................................................................augacuagauccauacucguUu.................. 1 1 c15
```

Star **Mature**

```
cuuacuacuauauauaacuugaugauauuugugggcgaguuugcuucugguucauguucuuuauaaaucaugacuagauccauacucgucugcagccaucaacuuaauc
.....................................................................Cugacuagauccauacucgucug.................  14     1  c15
.....................................................................augacuagauccauacucgucug.................  11     0  c15
.....................................................................augacuagauccauacucgucugc................  4      0  c15
.....................................................................augacuagauccauacucgucugU................  1      1  c15
.....................................................................Cugacuagauccauacucgucugc................  80     1  c15
......................................................................ugacuagauccauacucg.....................  77     0  c15
......................................................................ugacuagauccauacucA.....................  11     1  c15
......................................................................ugacuagauccauacucgu....................  102    0  c15
......................................................................ugacuagauccauacucAu....................  1      1  c15
......................................................................ugacuagauccauacucgA....................  1      1  c15
......................................................................ugacuagauccaAacucgu....................  1      1  c15
......................................................................ugacuaUauccauacucgu....................  1      1  c15
......................................................................ugacuagauAcauacucguc...................  1      1  c15
......................................................................ugacuagauccauacucguU...................  2      1  c15
......................................................................ugacuagauccauacucguc...................  514    0  c15
......................................................................ugacuagauccauacucguA...................  3      1  c15
......................................................................ugacuagauccauacAcguc...................  1      1  c15
......................................................................Agacuagauccauacucguc...................  1      1  c15
......................................................................ugacuagauccGuacucguc...................  1      1  c15
......................................................................ugacuagauccauacucgAc...................  4      1  c15
......................................................................ugacuagaucUauacucguc...................  2      1  c15
......................................................................ugacuUgauccauacucguc...................  1      1  c15
......................................................................ugacuagauccauacAcgucu..................  2      1  c15
......................................................................ugacuagauccauacucguUu..................  6      1  c15
......................................................................ugacuagauccGuacucgucu..................  1      1  c15
......................................................................ugacuagauccauacucgucC..................  40     1  c15
......................................................................ugacuagauccauacCcgucu..................  2      1  c15
......................................................................ugacuagauccauacucgucu..................  4044   0  c15
......................................................................ugUcuagauccauacucgucu..................  1      1  c15
......................................................................ugacuagauccauGcucgucu..................  2      1  c15
......................................................................ugacuagauccaGacucgucu..................  1      1  c15
......................................................................ugacuagauccauacuGgucu..................  1      1  c15
......................................................................ugacuCgauccauacucgucu..................  1      1  c15
......................................................................ugacuagauccauacucgucA..................  143    1  c15
......................................................................ugacCagauccauacucgucu..................  1      1  c15
......................................................................ugacuagauccauacucAucu..................  1      1  c15
......................................................................ugacuagauccauaUucgucu..................  2      1  c15
......................................................................ugacuaUauccauacucgucu..................  1      1  c15
......................................................................ugacuagauccauacucgGcu..................  1      1  c15
......................................................................ugacuagauccaAacucgucu..................  2      1  c15
......................................................................ugacuagauccauacucUucu..................  4      1  c15
......................................................................ugacuagauccauacucgAcu..................  1      1  c15
......................................................................ugacuagaucUauacucgucu..................  2      1  c15
......................................................................ugGcuagauccauacucgucu..................  1      1  c15
......................................................................uAacuagauccauacucgucu..................  4      1  c15
......................................................................ugacuagauAcauacucgucu..................  1      1  c15
......................................................................uUacuagauccauacucgucu..................  2      1  c15
......................................................................ugacuGgauccauacucgucu..................  2      1  c15
......................................................................ugacuagauUcauacucgucu..................  1      1  c15
......................................................................Agacuagauccauacucgucu..................  2      1  c15
......................................................................ugaUuagauccauacucgucu..................  3      1  c15
......................................................................ugacuagauccaCacucgucu..................  2      1  c15
......................................................................ugacuagauccauacucgCcu..................  1      1  c15
......................................................................Cgacuagauccauacucgucu..................  3      1  c15
......................................................................ugacuagaucAauacucgucu..................  1      1  c15
......................................................................ugacuagauccauacucCucu..................  1      1  c15
......................................................................ugacuagGuccauacucgucu..................  2      1  c15
......................................................................ugacuagauGcauacucgucu..................  1      1  c15
......................................................................ugacuagauccauacucgucG..................  7      1  c15
......................................................................ugacuagauccauacucguGu..................  5      1  c15
......................................................................ugacuagauAcauacucgucug.................  12     1  c15
......................................................................ugacuagauccauaUucgucug.................  18     1  c15
......................................................................ugacuaAauccauacucgucug.................  5      1  c15
......................................................................ugacuagauccCuacucgucug.................  3      1  c15
......................................................................ugacuagauccauacucgucug.................  48057  0  c15
......................................................................ugacuagauccauacucgucGg.................  28     1  c15
......................................................................ugacuagauccauacucguAug.................  14     1  c15
......................................................................ugacCagauccauacucgucug.................  9      1  c15
......................................................................uAacuagauccauacucgucug.................  29     1  c15
......................................................................ugacuagauccauUcucgucug.................  6      1  c15
```

Star Mature

```
cuuacuacuauauauaacuugaugauauuugugggcgaguuugcuucugguucauguucuuuauaaaucaugacuagauccauacucgucugcagccaucaacuuaauc
......................................................................ugacuagUuccauacucgucug.................	6	1	c15
......................................................................ugacuagauccauacCcgucug.................	8	1	c15
......................................................................ugacuagauccauacuUgucug.................	18	1	c15
......................................................................uCacuagauccauacucgucug.................	7	1	c15
......................................................................ugacuagauccauacuGgucug.................	9	1	c15
......................................................................ugacuGgauccauacucgucug.................	22	1	c15
......................................................................ugaAuagauccauacucgucug.................	6	1	c15
......................................................................ugacuagauccauacucgGcug.................	3	1	c15
......................................................................ugacuagauccauacucUucug.................	11	1	c15
......................................................................ugacuagauccauacucguUug.................	26	1	c15
......................................................................Cgacuagauccauacucgucug.................	30	1	c15
......................................................................ugacuagaCccauacucgucug.................	10	1	c15
......................................................................ugacuagauGcauacucgucug.................	2	1	c15
......................................................................ugacuagauccauacucgucAg.................	40	1	c15
......................................................................ugacuagaucAauacucgucug.................	5	1	c15
......................................................................ugacuagauccauacucAucug.................	18	1	c15
......................................................................ugacuagauccauaAucgucug.................	4	1	c15
......................................................................ugacuagauccGuacucgucug.................	13	1	c15
......................................................................ugacuagauccaGacucgucug.................	13	1	c15
......................................................................ugGcuagauccauacucgucug.................	13	1	c15
......................................................................ugacuagauccauacucgucuU.................	153	1	c15
......................................................................ugacuagauccauaGucgucug.................	1	1	c15
......................................................................ugacuagGuccauacucgucug.................	10	1	c15
......................................................................ugacuaCauccauacucgucug.................	3	1	c15
......................................................................ugacGagauccauacucgucug.................	1	1	c15
......................................................................ugacuagaucGauacucgucug.................	2	1	c15
......................................................................uUacuagauccauacucgucug.................	14	1	c15
......................................................................ugacuagauccauacucgCcug.................	11	1	c15
......................................................................Ggacuagauccauacucgucug.................	7	1	c15
......................................................................ugacuagaAccauacucgucug.................	6	1	c15
......................................................................ugacuCgauccauacucgucug.................	5	1	c15
......................................................................ugacuagauccaCacucgucug.................	11	1	c15
......................................................................ugacuagauccauacucguGug.................	30	1	c15
......................................................................ugacuagauccauacucgucuA.................	3379	1	c15
......................................................................ugacuagauccauacAcgucug.................	8	1	c15
......................................................................ugacAagauccauacucgucug.................	3	1	c15
......................................................................ugacuagauUcauacucgucug.................	14	1	c15
......................................................................ugacuagaucUauacucgucug.................	19	1	c15
......................................................................ugacuagauccauacucgucCg.................	47	1	c15
......................................................................ugUcuagauccauacucgucug.................	8	1	c15
......................................................................ugacuagauccUuacucgucug.................	10	1	c15
......................................................................ugacuagauccauacucCucug.................	4	1	c15
......................................................................ugaUuagauccauacucgucug.................	16	1	c15
......................................................................ugaGuagauccauacucgucug.................	4	1	c15
......................................................................ugacuUgauccauacucgucug.................	10	1	c15
......................................................................ugacuagauccaAacucgucug.................	6	1	c15
......................................................................ugacuagauccauacGcgucug.................	9	1	c15
......................................................................Agacuagauccauacucgucug.................	38	1	c15
......................................................................ugacuagaGccauacucgucug.................	2	1	c15
......................................................................ugacuagauccauacuAgucug.................	8	1	c15
......................................................................ugacuagauccauacucgucuC.................	141	1	c15
......................................................................ugacuagauccauacucgAcug.................	9	1	c15
......................................................................ugacuagauccauGcucgucug.................	10	1	c15
......................................................................ugacuaUauccauacucgucug.................	11	1	c15
......................................................................ugacuagauccUuacucgucugc................	23	1	c15
......................................................................ugacuagauccauacucgucugU................	8599	1	c15
......................................................................ugacuagGuccauacucgucugc................	25	1	c15
......................................................................ugacuagauccauGcucgucugc................	20	1	c15
......................................................................ugacuagauccauacuGgucugc................	21	1	c15
......................................................................ugacuagauccauacuAgucugc................	20	1	c15
......................................................................ugacuagaucGauacucgucugc................	8	1	c15
......................................................................ugacuagauccauacucgucugc................	114062	0	c15
......................................................................ugacuagauccauacuUgucugc................	29	1	c15
......................................................................ugacuagauccauacucAucugc................	42	1	c15
......................................................................ugacuagauccaGacucgucugc................	31	1	c15
......................................................................ugacuagauccaAacucgucugc................	38	1	c15
......................................................................ugacuagauccCuacucgucugc................	5	1	c15
......................................................................ugaGuagauccauacucgucugc................	10	1	c15
......................................................................ugacGagauccauacucgucugc................	1	1	c15
......................................................................uUacuagauccauacucgucugc................	28	1	c15
```

```
                              Star                              Mature
cuuacuacuauauauaacuugaugauauuugugggcgaguuugcuucugguucauguucuuuauaaaucaugacuagauccauacucgucugcagccaucaacuuaauc
......................................................................ugacuagauGcauacucgucugc................    10    1   c15
......................................................................ugacuagUuccauacucgucugc................    15    1   c15
......................................................................ugacuagauccauacucgucugG................    23    1   c15
......................................................................ugacuagauccaCacucgucugc................    19    1   c15
......................................................................ugaUuagauccauacucgucugc................    31    1   c15
......................................................................ugacuagauccauaAucgucugc................    9     1   c15
......................................................................ugacuagaAccauacucgucugc................    19    1   c15
......................................................................ugCcuagauccauacucgucugc................    3     1   c15
......................................................................Cgacuagauccauacucgucugc................    73    1   c15
......................................................................ugacuagauccauaGucgucugc................    10    1   c15
......................................................................ugacuagauccauacCcgucugc................    22    1   c15
......................................................................ugacuagaucAauacucgucugc................    20    1   c15
......................................................................ugacuCgauccauacucgucugc................    6     1   c15
......................................................................ugacuaUauccauacucgucugc................    12    1   c15
......................................................................ugaAuagauccauacucgucugc................    11    1   c15
......................................................................uAacuagauccauacucgucugc................    69    1   c15
......................................................................ugacuagCuccauacucgucugc................    3     1   c15
......................................................................ugacuagauAcauacucgucugc................    32    1   c15
......................................................................ugacuagauccauacucgAcugc................    26    1   c15
......................................................................ugacAagauccauacucgucugc................    11    1   c15
......................................................................ugacuagauccauacucguGugc................    75    1   c15
......................................................................ugacuagauccauacucguAugc................    70    1   c15
......................................................................ugacuagaucUauacucgucugc................    37    1   c15
......................................................................ugacuagauccauacucgGcugc................    6     1   c15
......................................................................Agacuagauccauacucgucugc................    91    1   c15
......................................................................ugacuagauccauCcucgucugc................    2     1   c15
......................................................................ugacuaCauccauacucgucugc................    8     1   c15
......................................................................ugacuagauccauacAcgucugc................    34    1   c15
......................................................................ugacuGgauccauacucgucugc................    62    1   c15
......................................................................uCacuagauccauacucgucugc................    11    1   c15
......................................................................ugacuagaCccauacucgucugc................    11    1   c15
......................................................................ugUcuagauccauacucgucugc................    29    1   c15
......................................................................ugacuagauccauacucUucugc................    15    1   c15
......................................................................ugacuagauccauacucgucugA................    1883  1   c15
......................................................................ugacCagauccauacucgucugc................    27    1   c15
......................................................................ugacuagauccauacucgucCgc................    44    1   c15
......................................................................ugacuagauccauacucCucugc................    3     1   c15
......................................................................ugacuagauccauacucgucGgc................    8     1   c15
......................................................................Ggacuagauccauacucgucugc................    14    1   c15
......................................................................ugGcuagauccauacucgucugc................    10    1   c15
......................................................................ugacuagauccauacucgucuAc................    1089  1   c15
......................................................................ugacuagauccauaUucgucugc................    39    1   c15
......................................................................ugacuagauccauacucgCcugc................    26    1   c15
......................................................................ugacuagauccauacucguUugc................    56    1   c15
......................................................................ugacuagauccauacucgucuCc................    16    1   c15
......................................................................ugacuagauUcauacucgucugc................    51    1   c15
......................................................................ugacuagauccauacucgucAgc................    28    1   c15
......................................................................ugacuagauccauacGcgucugc................    28    1   c15
......................................................................ugacuagauccGuacucgucugc................    27    1   c15
......................................................................ugacuaAauccauacucgucugc................    7     1   c15
......................................................................ugacuagauccauacucgucuUc................    15    1   c15
......................................................................ugacuagauccauUcucgucugc................    2     1   c15
......................................................................ugacuUgauccauacucgucugc................    30    1   c15
......................................................................uCacuagauccauacucgucugca...............    1     1   c15
......................................................................ugacuagauAcauacucgucugca...............    1     1   c15
......................................................................ugacuagauccauacucgucugca...............    618   0   c15
......................................................................ugacuagauccauacuUgucugca...............    1     1   c15
......................................................................ugacuGgauccauacucgucugca...............    1     1   c15
......................................................................ugacuagauccauacucgucuAca...............    1     1   c15
......................................................................ugacuagauccauacucgucAgca...............    1     1   c15
......................................................................ugacuagauccauacucgucugcG...............    15    1   c15
......................................................................ugacuagauccauacucgucugAa...............    23    1   c15
......................................................................ugacuagauccauacucgucugcU...............    3051  1   c15
......................................................................ugacuagauccauacucgucugcC...............    173   1   c15
......................................................................ugacuagauccauacucguAugca...............    1     1   c15
......................................................................ugacuagauccauacuAgucugca...............    1     1   c15
......................................................................ugacuagauccauacucgucugUa...............    281   1   c15
......................................................................ugacuagauccaAacucgucugca...............    1     1   c15
......................................................................Agacuagauccauacucgucugca...............    1     1   c15
......................................................................ugacuagauccauacucgucugcaC..............    3     1   c15
```

Star Mature

cuuacuacuauauauaacuugaugauauuugugggcgaguuugcuucugguucauguucuuuauaaaucaugacuagauccauacucgucugcagccaucaacuuaauc

....ugacuagauccauacucgucugcaA..............	34	1	c15
....ugacuagauccauacucgucugcaU..............	86	1	c15
....ugacuagauccauacucgucugAagc.............	1	1	c15
....ugacuagauccauacucgucugcaUc.............	2	1	c15
....ugacuagauccauacucgucugcaAc.............	1	1	c15
....ugacuagauccauacucgucugGagcca...........	1	1	c15
....gacuagauccauacucgu....................	2	0	c15
....gacuagauccauacucguc...................	4	0	c15
....gacuagauccauacucgucC..................	1	1	c15
....gacuagauccauacucgucu..................	34	0	c15
....gacuagauccauacucgucCg.................	1	1	c15
....gacuagauccaCacucgucug.................	1	1	c15
....gacuagauccauacucgucuU.................	3	1	c15
....gacuaAauccauacucgucug.................	1	1	c15
....Cacuagauccauacucgucug.................	1	1	c15
....gacuagauccauacucgucuA.................	27	1	c15
....gacuagauccauacucgucug.................	323	0	c15
....gacuagGuccauacucgucug.................	1	1	c15
....gGcuagauccauacucgucug.................	1	1	c15
....Uacuagauccauacucgucug.................	1	1	c15
....gacuagauccauaAucgucug.................	1	1	c15
....gacuagauccauUcucgucug.................	1	1	c15
....Uacuagauccauacucgucugc................	1	1	c15
....gacuagauccauaAucgucugc................	1	1	c15
....gacCagauccauacucgucugc................	2	1	c15
....gGcuagauccauacucgucugc................	2	1	c15
....gacuagauccauacucgucugc................	649	0	c15
....gacuagauccauacuAgucugc................	1	1	c15
....gacuagauUcauacucgucugc................	2	1	c15
....gacuagauccauacuUgucugc................	1	1	c15
....gacuagauccauacucgucugU................	19	1	c15
....gacuGgauccauacucgucugc................	1	1	c15
....gacuaAauccauacucgucugc................	1	1	c15
....gacuagaAccauacucgucugc................	1	1	c15
....gacuagauccauacucgAcugc................	1	1	c15
....gacuagauccauacucgucugA................	9	1	c15
....gacuagauccauacucgCcugc................	1	1	c15
....gacuagauccauacucgucuAc................	12	1	c15
....gacuagauccauacucgucGgc................	1	1	c15
....gacuagauccauacucgucAgc................	1	1	c15
....gCcuagauccauacucgucugc................	1	1	c15
....gacuagauccauacucCucugc................	1	1	c15
....gacuagauccauacucgucugca...............	4	0	c15
....gacuagauccauacucgucugUa...............	1	1	c15
....gacuagauccauacucgucugcU...............	23	1	c15
....acuagauccauacucgucu..................	1	0	c15
....acuagauccauacucgucug.................	6	0	c15
....acuagauccauacucgucugc................	85	0	c15
....aGuagauccauacucgucugc................	1	1	c15
....acuagauccauacucgucugA................	2	1	c15
....acuagauccauacucgucugU................	4	1	c15
....acuagauccauacucgucugcU...............	157	1	c15
....acuagauccauacucgucugUa...............	35	1	c15
....acuagauccauacucgucugcC...............	7	1	c15
....acuagauccauacucgucugca...............	107	0	c15
....acuagauccauacucgucugcG...............	1	1	c15
....acuagauccauacucgucugcaU..............	9	1	c15
....cuagauccauacucgucu..................	2	0	c15
....cuagauccauacucgucug.................	15	0	c15
....cuagauccauacucUucugc................	1	1	c15
....cuagauccauacucgucugc................	16	0	c15
....cuagauccauacucgucugU................	3	1	c15
....cuagauccauacucgucugcU...............	6	1	c15
....cuagauccauacucgucugcC...............	1	1	c15
....cuagauccauacucgucugca...............	1	0	c15
....cuagauccauacucgucugcaA..............	1	1	c15
....uagauccauacucgucug.................	7	0	c15
....uagauccauacucgucuA.................	1	1	c15
....uagauccauacucgucugU................	1	1	c15
....uagaucUauacucgucugc................	1	1	c15

Star Mature

```
cuuacuacuauauauaacuugaugauauuugugggcgaguuugcuucugguucauguucuuuauaaaucaugacuagauccauacucgucugcagccaucaacuuaauc
..........................................................................uagauccauacucgucugc................	11	0	c15
..........................................................................uagauccauacucgucugcU...............	1	1	c15
...........................................................................aUauccauacucgucugc................	1	1	c15
...........................................................................agauccauacucgucugU................	1	1	c15
...........................................................................agauccauacucgucugc................	13	0	c15
...........................................................................agauccauacucgucugcU...............	1	1	c15
...........................................................................agauccauacucgucugca...............	1	0	c15
............................................................................gauccauacucgucugcC...............	1	1	c15
............................................................................gauccauacucgucugca...............	1	0	c15
```

Provisional ID	:	group10_14867
Score total	:	131166.5
Score for star read(s)	:	3.9
Score for read counts	:	131156.2
Score for mfe	:	1.8
Score for randfold	:	1.6
Score for cons. seed	:	3
Total read count	:	257269
Mature read count	:	246722
Loop read count	:	0
Star read count	:	10547

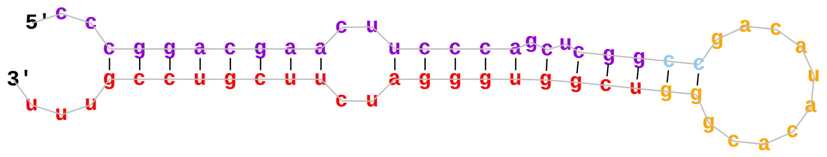

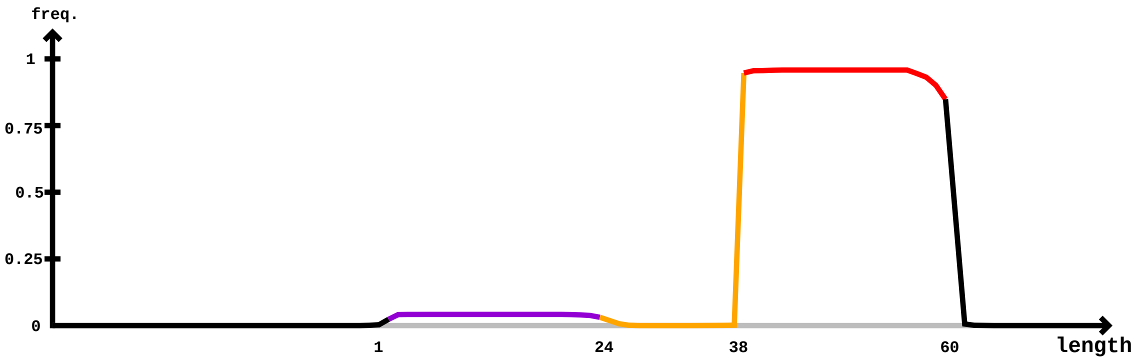


```
                                                 Star                                Mature
5'- cuggaagaaagaagaacgguaucaagugcuacgcccggacgaacuucccagcucggccgacauacacgggucggugggaucuucguccguuuguaucgcuuauacguu   -3'   obs
    cuggaagaaagaagaacgguaucaagugcuacgcccggacgaacuucccagcucggccgacauacacgggucggugggaucuucguccguuuguaucgcuuauacguu         exp
    ..................((((.(((((.((((..((((((((..(((((.(.(((((..........)))))))))))..))))))))..)))).)))))))))...   reads   mm   sample
    ...............................cgcccggacgaacuucccag.........................................................   1       0    c15
    ...............................cgcccggacgaacuucccagc........................................................   6       0    c15
    ...............................cgcccggacgaacuucccagcu.......................................................   9       0    c15
    ...............................cgcccggaGgaacuucccagcu.......................................................   1       1    c15
    ...............................cgcccggacgaacuucccagcuc......................................................   8       0    c15
    ...............................cgcccggacgaacuucccagcucg.....................................................   26      0    c15
    ...............................cgcccggacgaacuucccagcucA.....................................................   3       1    c15
    ...............................cgcccggacgaacuucccagcucgA....................................................   2       1    c15
    ...............................cgcccggacgaacuucccagcucgU....................................................   1       1    c15
    ...............................cgcccggacgaacuucccagcucgg....................................................   16      0    c15
    ...............................cgcccggacgaacuucccagcucggc...................................................   24      0    c15
    ...............................cgcccggacgaacuucccagcucggU...................................................   2       1    c15
    ...............................cUcccggacgaacuucccagcucggc...................................................   1       1    c15
    ...............................cgcccggacgaacuucccagcucggcc..................................................   27      0    c15
    ...............................cgcccggacgaacuucccagcucgAcc..................................................   1       1    c15
    ...............................cgcccggacgaacuucccagcucggcA..................................................   1       1    c15
    ...............................cgcccggacgaacuucccagcucggcU..................................................   18      1    c15
    ................................gcccggacgaacuucccagc........................................................   4       0    c15
    ................................gcccggacgaacuucccagA........................................................   1       1    c15
    ................................Ucccggacgaacuucccagcu.......................................................   2       1    c15
    ................................gcccggacgaacAucccagcu.......................................................   1       1    c15
    ................................gcccggacgaacuucccagcA.......................................................   2       1    c15
    ................................gcccggacgaacuucccagcu.......................................................   66      0    c15
    ................................gccUggacgaacuucccagcuc......................................................   1       1    c15
    ................................gcccggaAgaacuucccagcuc......................................................   1       1    c15
    ................................gcccggacgaacuuccGagcuc......................................................   1       1    c15
    ................................gcccggacgaacuucccagcuA......................................................   1       1    c15
    ................................gcccggacgaacuucccagcuc......................................................   39      0    c15
    ................................gcccggacgaacuucccagcuU......................................................   2       1    c15
    ................................Ucccggacgaacuucccagcucg.....................................................   2       1    c15
    ................................gcccggacgaacuucccagcucU.....................................................   5       1    c15
    ................................gcccggacgaacuucccagcucC.....................................................   1       1    c15
    ................................gcccggacgaacuucccagcucA.....................................................   10      1    c15
```

Star **Mature**

```
cuggaagaaagaagaacgguaucaagugcuacgcccggacgaacuucccagcucggccgacauacacgggucggugggaucuucguccguuuguaucgcuuauacguu
................................gcccggacgaacuucccagcucg.....................................................	84	0	c15
................................gcccggacgaacuucccagcucgg....................................................	39	0	c15
................................Acccggacgaacuucccagcucgg....................................................	1	1	c15
................................Ccccggacgaacuucccagcucgg....................................................	1	1	c15
................................gcccggacgaacuucccagcucgU....................................................	13	1	c15
................................Ucccggacgaacuucccagcucgg....................................................	1	1	c15
................................gcccggacgaacuucccagcucgA....................................................	1	1	c15
................................Ucccggacgaacuucccagcucggc...................................................	1	1	c15
................................gcccggacgaacuucccagcucggU...................................................	2	1	c15
................................gcccggacAaacuucccagcucggc...................................................	1	1	c15
................................gcccggacgaacuucccagcucggc...................................................	36	0	c15
................................gcccggacgaacuAcccagcucggcc..................................................	1	1	c15
................................gcccggacgaacuucccagcucggcA..................................................	1	1	c15
................................gcccggacgaacuucccagcucggcc..................................................	17	0	c15
................................gcccggacgaacuucccagcucggcU..................................................	14	1	c15
................................gcccggacgaacuucccagcucggccU.................................................	3	1	c15
................................gcccggacgaacuucccagcucggccA.................................................	1	1	c15
.................................cccggacgaacuucccag.........................................................	1	0	c15
.................................cccggacgaacuucccagc........................................................	5	0	c15
.................................cccggacgaacuucccagcu.......................................................	27	0	c15
.................................cccggacgaacuucccagcA.......................................................	1	1	c15
.................................cccggacgaacuucccagcuA......................................................	2	1	c15
.................................cccggacgaacuucccagcAc......................................................	2	1	c15
.................................cccggacgaacuucccagcuU......................................................	3	1	c15
.................................cccggacgaacuucccagcuc......................................................	52	0	c15
.................................cccggacgaacuCcccagcucg.....................................................	1	1	c15
.................................cccggacgaacuucccagcucg.....................................................	187	0	c15
.................................ccUggacgaacuucccagcucg.....................................................	1	1	c15
.................................cccggacgaacuucccagcucA.....................................................	15	1	c15
.................................cccggacgaacuucccagcucU.....................................................	2	1	c15
.................................cccggGcgaacuucccagcucg.....................................................	1	1	c15
.................................cccggacgaacuucccaUcucgg....................................................	1	1	c15
.................................cccggacgaacuucccagcAcgg....................................................	1	1	c15
.................................cccggacgaacuucccagcGcgg....................................................	1	1	c15
.................................ccGggacgaacuucccagcucgg....................................................	1	1	c15
.................................cccggacgaacuucccagcucgC....................................................	1	1	c15
.................................cccggacgaacuGcccagcucgg....................................................	1	1	c15
.................................cUcggacgaacuucccagcucgg....................................................	1	1	c15
.................................cccggacgaacuucccagcucgU....................................................	8	1	c15
.................................cGcggacgaacuucccagcucgg....................................................	1	1	c15
.................................Uccggacgaacuucccagcucgg....................................................	1	1	c15
.................................cccggacgaacuucccagcucgg....................................................	250	0	c15
.................................cccggacgaacuucccagcucgA....................................................	5	1	c15
.................................cccggacgaacuucccagcucggA...................................................	7	1	c15
.................................cccggacgaacuucccagcucgAc...................................................	8	1	c15
.................................cccggacgaacuucccagcucggc...................................................	212	0	c15
.................................cccggacgaacuAcccagcucggc...................................................	1	1	c15
.................................cccggacgaacuGcccagcucggc...................................................	1	1	c15
.................................cccggacAaacuucccagcucggc...................................................	1	1	c15
.................................cccggacgaacuucccagUucggc...................................................	1	1	c15
.................................cccggacgaacuucccagcucggU...................................................	23	1	c15
.................................cccggacgaacuuccAagcucggc...................................................	1	1	c15
.................................cccggacgaacuuccUagcucggc...................................................	1	1	c15
.................................cccggacgaacuucccagcucggUc..................................................	1	1	c15
.................................cccggacgaacuucccagcucggcU..................................................	85	1	c15
.................................cccggacgaacuucccagcucggcc..................................................	2	0	c15
.................................cccggacgaacuucccagcucggcA..................................................	13	1	c15
..................................ccggacgaacuucccagc........................................................	10	0	c15
..................................ccggacgaacuucccagcu.......................................................	24	0	c15
..................................ccggacgaacuucccagcuU......................................................	3	1	c15
..................................ccUgacgaacuucccagcuc......................................................	1	1	c15
..................................ccggacgaacuucccagcuc......................................................	90	0	c15
..................................ccggacgaacuucccagcAc......................................................	1	1	c15
..................................ccggaUgaacuucccagcuc......................................................	1	1	c15
..................................ccggacgaacuucccagcuA......................................................	1	1	c15
..................................ccggUcgaacuucccagcucg.....................................................	1	1	c15
..................................ccggacUaacuucccagcucg.....................................................	1	1	c15
..................................ccggacgaacuucccagcucU.....................................................	18	1	c15
..................................ccggGcgaacuucccagcucg.....................................................	1	1	c15
..................................Ucggacgaacuucccagcucg.....................................................	1	1	c15
```

```
                                  Star                                   Mature
cuggaagaaagaagaacgguaucaagugcuacgcccggacgaacuucccagcucggccgacauacacgggucggugggaucuucguccguuuguaucgcuuauacguu
..................................ccggacgaacuucccGgcucg.....................................................	1	1	c15
..................................ccggacgaacuucccagcuUg.....................................................	1	1	c15
..................................ccggacgaacuucccagcucC.....................................................	2	1	c15
..................................ccggacgaacuucccagcucg.....................................................	338	0	c15
..................................ccggacgaacuucccagcucA.....................................................	28	1	c15
..................................ccggaAgaacuucccagcucgg....................................................	2	1	c15
..................................ccggacgaacuucccagcucgg....................................................	342	0	c15
..................................ccggacgaacuucccagcucgC....................................................	2	1	c15
..................................ccggacgaacuucccagcuUgg....................................................	1	1	c15
..................................ccggacgaacuucccagcucAg....................................................	1	1	c15
..................................ccggacgaacuucccagcucgU....................................................	15	1	c15
..................................ccggacgUacuucccagcucgg....................................................	1	1	c15
..................................ccggacgaacuucccagcucgA....................................................	15	1	c15
..................................ccUgacgaacuucccagcucgg....................................................	1	1	c15
..................................ccggacgaacuuccAagcucgg....................................................	1	1	c15
..................................ccggacgaacuucccUgcucggc...................................................	1	1	c15
..................................cAggacgaacuucccagcucggc...................................................	1	1	c15
..................................Ucggacgaacuucccagcucggc...................................................	1	1	c15
..................................ccggacgaacuucccagcucggA...................................................	1	1	c15
..................................ccggaUgaacuucccagcucggc...................................................	1	1	c15
..................................ccggacgaacuucUcagcucggc...................................................	1	1	c15
..................................ccggacgaaUuucccagcucggc...................................................	1	1	c15
..................................ccggacgaacAucccagcucggc...................................................	1	1	c15
..................................ccggacgaacuucccagcucgAc...................................................	7	1	c15
..................................cGggacgaacuucccagcucggc...................................................	2	1	c15
..................................cUggacgaacuucccagcucggc...................................................	1	1	c15
..................................ccggacgaacuucccaUcucggc...................................................	1	1	c15
..................................ccggacgaacuucccagcucggc...................................................	429	0	c15
..................................ccggacgaacuucccagcucggU...................................................	8	1	c15
..................................Gcggacgaacuucccagcucggc...................................................	1	1	c15
..................................ccggacgaaAuucccagcucggc...................................................	1	1	c15
..................................cGggacgaacuucccagcucggcc..................................................	1	1	c15
..................................ccggacgaacuucccagcucggcA..................................................	49	1	c15
..................................ccggacgaacuuUccagcucggcc..................................................	1	1	c15
..................................ccggacgaacuucccagcucggcU..................................................	53	1	c15
..................................ccUgacgaacuucccagcucggcc..................................................	1	1	c15
..................................ccggaUgaacuucccagcucggcc..................................................	2	1	c15
..................................ccggacgaacuucccagcuUggcc..................................................	2	1	c15
..................................ccggacgaacuucccagcucAgcc..................................................	1	1	c15
..................................Ucggacgaacuucccagcucggcc..................................................	2	1	c15
..................................ccggacgaacuucccaAcucggcc..................................................	1	1	c15
..................................ccgCacgaacuucccagcucggcc..................................................	1	1	c15
..................................cUggacgaacuucccagcucggcc..................................................	2	1	c15
..................................ccggacgaacuucccagcucggcc..................................................	638	0	c15
..................................ccggacgaacuucccagcCcggcc..................................................	1	1	c15
..................................ccggacgaacuAcccagcucggcc..................................................	1	1	c15
..................................ccggacgaacuucccagcucggAc..................................................	4	1	c15
..................................ccggacgaacuucccagcuGggcc..................................................	1	1	c15
..................................ccggacAaacuucccagcucggcc..................................................	1	1	c15
..................................ccggacgaacuucccagcucggccA.................................................	34	1	c15
..................................ccggacgaacuucccagcucggccU.................................................	83	1	c15
..................................ccggacgaacuucccagcucggccC.................................................	6	1	c15
..................................ccggacgaacuucccagcucggccCa................................................	1	1	c15
..................................ccggacgaacuucccagcucggccUa................................................	2	1	c15
...................................cggacgaacuucccagcuc......................................................	2	0	c15
...................................cggacgaacuucccagcucg.....................................................	8	0	c15
...................................cggacgaacuucccagcucA.....................................................	1	1	c15
...................................cggacgaacuucccagcucgA....................................................	1	1	c15
...................................cggacgaacuucccagcucgg....................................................	11	0	c15
...................................cggacgaacuucccagcucggU...................................................	1	1	c15
...................................cggacgaacuucccagcucggA...................................................	1	1	c15
...................................cggacgaacuucccagcucggc...................................................	16	0	c15
...................................cggacgaacuucccagcucggcA..................................................	3	1	c15
...................................cggacgaacuucccagcucggcc..................................................	19	0	c15
...................................cggacgaacuucccagcucggcG..................................................	1	1	c15
...................................cCgacgaacuucccagcucggcc..................................................	1	1	c15
...................................Uggacgaacuucccagcucggcc..................................................	1	1	c15
...................................cggacgaacuucccagcucggcU..................................................	3	1	c15
....................................ggacgaacuucccagcucggcc..................................................	1	0	c15
.....................................................................gAucggugggaucuucguccgu.................	1	1	c15
```

```
                                 Star                                 Mature
cuggaagaaagaagaacgguaucaagugcuacgcccggacgaacuucccagcucggccgacauacacgggucggugggaucuucguccguuuguaucgcuuauacguu
....................................................................gUucggugggaucuucguccguuu................   1     1     c15
.....................................................................Cucggugggaucuucguccguu.................   1     1     c15
.....................................................................Cucggugggaucuucguccguuu................   40    1     c15
......................................................................ucggugggaucuucgucA....................   1     1     c15
......................................................................ucggugggaucuucgucc....................   67    0     c15
......................................................................ucggugggaucuucguccA...................   1     1     c15
......................................................................ucggugggaucuucgucUg...................   1     1     c15
......................................................................ucggugggaucuucguccg...................   15    0     c15
......................................................................ucggugggaucuucguccgu..................   54    0     c15
......................................................................ucggugggaucuucguccAu..................   1     1     c15
......................................................................ucggugggaucuucguccguu.................   581   0     c15
......................................................................ucggugggaucuucguccguC.................   2     1     c15
......................................................................ucggugggaucuucguccgAu.................   1     1     c15
......................................................................Acggugggaucuucguccguu.................   2     1     c15
......................................................................ucggugggaucuucguccguA.................   1     1     c15
......................................................................ucggugggaucuucguccguG.................   1     1     c15
......................................................................ucggugggaucuucguccAuu.................   2     1     c15
......................................................................ucggugggaucuucguccUuu.................   1     1     c15
......................................................................ucggugggaucuucguccgCu.................   1     1     c15
......................................................................ucUgugggaucuucguccguuu................   7     1     c15
......................................................................ucggugggaucuAcguccguuu................   4     1     c15
......................................................................ucggugggaucuucgucUguuu................   22    1     c15
......................................................................uGggugggaucuucguccguuu................   17    1     c15
......................................................................ucggGgggaucuucguccguuu................   13    1     c15
......................................................................ucgguggUaucuucguccguuu................   2     1     c15
......................................................................ucggAgggaucuucguccguuu................   18    1     c15
......................................................................ucgAugggaucuucguccguuu................   16    1     c15
......................................................................ucggugAgaucuucguccguuu................   3     1     c15
......................................................................Gcggugggaucuucguccguuu................   5     1     c15
......................................................................ucggugggauUuucguccguuu................   7     1     c15
......................................................................ucggugggaucuucguccAuuu................   26    1     c15
......................................................................ucggugggaucuuGguccguuu................   3     1     c15
......................................................................ucggugggaucuucguAcguuu................   6     1     c15
......................................................................ucgguAggaucuucguccguuu................   8     1     c15
......................................................................ucggugggaucuucCuccguuu................   3     1     c15
......................................................................ucggugggaucuucguccguuA................   223   1     c15
......................................................................ucggugggGucuucguccguuu................   5     1     c15
......................................................................ucAgugggaucuucguccguuu................   28    1     c15
......................................................................ucgguggCaucuucguccguuu................   2     1     c15
......................................................................ucggugggaucuucguccgGuu................   15    1     c15
......................................................................ucgguggAaucuucguccguuu................   4     1     c15
......................................................................ucggugggaucuuAguccguuu................   5     1     c15
......................................................................Ccggugggaucuucguccguuu................   10    1     c15
......................................................................ucggugggaucGucguccguuu................   5     1     c15
......................................................................ucggugggaucuucguccguAu................   14    1     c15
......................................................................ucggugggaucuucguGcguuu................   6     1     c15
......................................................................ucggugggaucuGcguccguuu................   9     1     c15
......................................................................ucgguCggaucuucguccguuu................   1     1     c15
......................................................................ucggugggaucuuUguccguuu................   25    1     c15
......................................................................ucggugggaucuucguccguuC................   96    1     c15
......................................................................ucggCgggaucuucguccguuu................   21    1     c15
......................................................................ucggugggUucuucguccguuu................   6     1     c15
......................................................................ucgguUggaucuucguccguuu................   9     1     c15
......................................................................ucggugggaucuucgAccguuu................   10    1     c15
......................................................................ucggugUgaucuucguccguuu................   3     1     c15
......................................................................Acggugggaucuucguccguuu................   15    1     c15
......................................................................ucgUugggaucuucguccguuu................   4     1     c15
......................................................................ucggugggaucuucgucAguuu................   12    1     c15
......................................................................ucggugggaucuucguccguGu................   4     1     c15
......................................................................ucggugggaucuucguccgAuu................   28    1     c15
......................................................................ucggugggaucuCcguccguuu................   8     1     c15
......................................................................ucggugggauAuucguccguuu................   3     1     c15
......................................................................ucggugggaucCucguccguuu................   4     1     c15
......................................................................ucggugggaucuucAuccguuu................   9     1     c15
......................................................................ucggugggaucAucguccguuu................   16    1     c15
......................................................................uAggugggaucuucguccguuu................   14    1     c15
......................................................................ucggugggaucuucguccUuuu................   6     1     c15
......................................................................ucggugggaucuucguUcguuu................   11    1     c15
......................................................................ucggugggaucuucUuccguuu................   4     1     c15
......................................................................ucggugggaucuucguccCuuu................   5     1     c15
```

```
                                Star                                Mature
cuggaagaaagaagaacgguaucaagugcuacgcccggacgaacuucccagcucggccgacauacacgggucggugggaucuucguccguuuguaucgcuuauacguu
......................................................................ucggugggaucuucguccguuG................   9       1   c15
......................................................................ucggugggaAcuucguccguuu................   2       1   c15
......................................................................ucggugggaucuucguccguCu................   13      1   c15
......................................................................ucggugggaucuucgGccguuu................   3       1   c15
......................................................................ucggugggaCcuucguccguuu................   8       1   c15
......................................................................ucggugggaucuucguccgCuu................   15      1   c15
......................................................................ucggugggCucuucguccguuu................   2       1   c15
......................................................................uUggugggaucuucguccguuu................   32      1   c15
......................................................................ucggugCgaucuucguccguuu................   3       1   c15
......................................................................ucggugggauGuucguccguuu................   3       1   c15
......................................................................ucggugggaucuucgucGguuu................   2       1   c15
......................................................................ucggugggaucuucgCccguuu................   9       1   c15
......................................................................ucggugggaucuucguccguuu................   35157   0   c15
......................................................................ucggugggaucuucguccguuuU...............   2021    1   c15
......................................................................ucggugggaucuucguccguuug...............   144     0   c15
......................................................................ucggugggaucuGcguccguuug...............   1       1   c15
......................................................................ucggugggaucuucguccAuuug...............   1       1   c15
......................................................................ucCgugggaucuucguccguuug...............   1       1   c15
......................................................................ucggugggaucuucguccguuuC...............   46      1   c15
......................................................................ucggugggaucuucguccguuuA...............   140     1   c15
......................................................................ucggugggaucuucguccguuuAu..............   15      1   c15
......................................................................ucggugggaucuucguccguuugC..............   1       1   c15
......................................................................ucggugggaucuucguccguuugu..............   64      0   c15
......................................................................ucggugggaucuucguccguuuCu..............   5       1   c15
......................................................................ucggugggaucuucguccguuuUu..............   214     1   c15
......................................................................ucggugggaucuucguccguuugG..............   1       1   c15
......................................................................ucggugggaucuucguccguuugA..............   1       1   c15
......................................................................ucggugggaucuucguccguuuguU.............   5       1   c15
......................................................................ucggugggaucuucguccguuuUua.............   4       1   c15
......................................................................ucggugggaucuucguccguuuAua.............   1       1   c15
......................................................................ucggugggaucuucguccguuugua.............   31      0   c15
......................................................................ucggugggaucuucguccguuuguUu............   2       1   c15
......................................................................ucggugggaucuucguccguuuUuau............   3       1   c15
......................................................................ucggugggaucuucguccguuuguaA............   3       1   c15
......................................................................ucggugggaucuucguccguuuguau............   15      0   c15
......................................................................ucggugggaucuucguccguuuguauA...........   1       1   c15
.......................................................................cggugggaucuucguccguu.................   4       0   c15
.......................................................................cggGgggaucuucguccguuu................   1       1   c15
.......................................................................cggugggaucuucguccguuC................   1       1   c15
.......................................................................cgguAggaucuucguccguuu................   1       1   c15
.......................................................................cggugggaucuucguccguuu................   79      0   c15
.......................................................................cggugggaucuucguccguuA................   1       1   c15
.......................................................................cggugggauAuucguccguuu................   1       1   c15
.......................................................................cggugggaucuucguccguuug...............   40      0   c15
.......................................................................cggugggaucuucguccguuuU...............   22      1   c15
.......................................................................cggugggaucuucguccguuuA...............   5       1   c15
.......................................................................cggugggaucuucgucUguuug...............   1       1   c15
.......................................................................cggugggaucuucguccguuuUu..............   3       1   c15
.......................................................................cggugggaucuucguccguuuAu..............   1       1   c15
.......................................................................cggugggaucuucguccguuugu..............   19      0   c15
.......................................................................cggugggaucuucguccguuugua.............   17      0   c15
.......................................................................cggugggaucuucguccguuuguU.............   4       1   c15
.......................................................................Uggugggaucuucguccguuugua.............   1       1   c15
.......................................................................cggugggaucuucguccguuuguUu............   2       1   c15
.......................................................................cggugggaucuucguccCuuuguau............   1       1   c15
.......................................................................Uggugggaucuucguccguuuguau............   1       1   c15
.......................................................................cggugggaucuucguccguuuguau............   41      0   c15
.......................................................................cggugggaucuucguccguGuguau............   1       1   c15
.......................................................................cggugggaucuucguccguuuguaA............   10      1   c15
.......................................................................cggugggaucuucguccguuuguauU...........   3       1   c15
........................................................................ggugggaucuucguccguuu................   2       0   c15
.........................................................................gugggaucuucguccguuu................   1       0   c15
.........................................................................gugggaucuucguccguuuU...............   1       1   c15
.........................................................................gugggaucuucguccguuugua.............   2       0   c15
.........................................................................gugggaucuucguccguuuguau............   5       0   c15
.........................................................................gugggaucuucguccguuuguauU...........   1       1   c15
..........................................................................ugggaucuucguccguuu................   2       0   c15
..........................................................................ugggaucuucguccguuugua.............   1       0   c15

...............................cgcccggacgaacuucccagc........................................................   1       0   c02
```

Star **Mature**

cuggaagaaagaagaacgguaucaagugcuacgcccggacgaacuucccagcucggccgacauacacgggucggugggaucuucguccguuuguaucgcuuauacguu			
...............................cgcccggacgaacuucccagcu...	1	0	c02
...............................cgcccggacgaacuucccagcuc..	3	0	c02
...............................cgcccggacgaacuucccagcuUg...	1	1	c02
...............................cgcccggacgaacuucccagcucgg..	3	0	c02
...............................cgcccggacgaacuucccagcucggc...	4	0	c02
...............................cgcccggacgaacuucccagcucggcA..	1	1	c02
................................gcccggacgaacuucccag...	1	0	c02
................................gcccggacgaacuucccagc..	2	0	c02
................................gcccggacgaacuucccagcucg...	2	0	c02
................................gcccggacgaacuucccagcucU...	1	1	c02
................................gcccggacgaacuucccagcucgg..	2	0	c02
................................Ucccggacgaacuucccagcucggc...	1	1	c02
................................gcccggacgaacuucccagcucggc...	13	0	c02
.................................cccggacgaacuucccag...	1	0	c02
.................................cccggacgaacuucccagc..	17	0	c02
.................................cccggacgaacuucccagcu...	46	0	c02
.................................cccggacgaacuucccagcA...	1	1	c02
.................................Gccggacgaacuucccagcu...	1	1	c02
.................................cccggacgCacuucccagcuc..	1	1	c02
.................................cccggacgaCcuucccagcuc..	1	1	c02
.................................cccggacgaacuucccagcuc..	52	0	c02
.................................cccggacgaaAuucccagcuc..	1	1	c02
.................................cccggCcgaacuucccagcuc..	1	1	c02
.................................cccggacgaacuucccagcucg...	153	0	c02
.................................cccggacgCacuucccagcucg...	4	1	c02
.................................cccggacgaCcuucccagcucg...	4	1	c02
.................................cccggacgaacuucccagcucU...	1	1	c02
.................................cccggacgaacuucAcagcucg...	1	1	c02
.................................cccggacgaacuucccagcucA...	1	1	c02
.................................Accggacgaacuucccagcucgg..	1	1	c02
.................................cccggacgaaUuucccagcucgg..	1	1	c02
.................................cccggacAaacuucccagcucgg..	1	1	c02
.................................cccggacgaacuuccAagcucgg..	1	1	c02
.................................cccggacgaacuucccaUcucgg..	1	1	c02
.................................cccggacgaacuucccagcucgg..	626	0	c02
.................................cccggacgaacuucccagcucCg..	1	1	c02
.................................cccggacgaacuucccagcuUgg..	1	1	c02
.................................cccggacgaacuucccUgcucgg..	2	1	c02
.................................cccggacgCacuucccagcucgg..	7	1	c02
.................................cccggacgaacuucccaCcucgg..	1	1	c02
.................................cccggCcgaacuucccagcucgg..	3	1	c02
.................................cccggacgaCcuucccagcucgg..	3	1	c02
.................................cccggacgaacuAcccagcucgg..	1	1	c02
.................................cUcggacgaacuucccagcucgg..	1	1	c02
.................................cccggacgaacuucccCgcucgg..	1	1	c02
.................................cccggacgaacuucccagcucUg..	1	1	c02
.................................cccUgacgaacuucccagcucgg..	1	1	c02
.................................cccggacgaacuucccagAucgg..	1	1	c02
.................................cccggacgaacuucccagcucgU..	2	1	c02
.................................cccAgacgaacuucccagcucggc...	1	1	c02
.................................cccggacgaacuucccagcucgCc...	2	1	c02
.................................cccggacgaacGucccagcucggc...	1	1	c02
.................................cccggacgaacuucccCgcucggc...	1	1	c02
.................................cccggacgaCcuucccagcucggc...	4	1	c02
.................................cccggacgaacuucccagcucggU...	6	1	c02
.................................cccggacgCacuucccagcucggc...	4	1	c02
.................................cccggacgaacuucccagcucCgc...	1	1	c02
.................................ccUggacgaacuucccagcucggc...	1	1	c02
.................................cccggacgaacuucccagcuGggc...	1	1	c02
.................................cccggacgaacuucccaCcucggc...	1	1	c02
.................................cAcggacgaacuucccagcucggc...	1	1	c02
.................................ccAggacgaacuucccagcucggc...	2	1	c02
.................................cccggacgaacuucccagcucggc...	340	0	c02
.................................cccggacgaacuucccaAcucggc...	1	1	c02
.................................cccggacgaacuucccagcucggA...	8	1	c02
.................................cccggCcgaacuucccagcucggc...	3	1	c02
.................................cccggacgaacuucccagcuUggc...	1	1	c02
.................................cccgCacgaacuucccagcucggc...	1	1	c02
.................................cccggacgaacuucccagcucggcA..	7	1	c02
.................................cccggacgaacuucccagcucggcc..	1	0	c02

Star   Mature

cuggaagaaagaagaacgguaucaagugcuacgcccggacgaacuucccagcucggccgacauacacgggucggugggaucuucguccguuuguaucgcuuauacguu			
.................................cccggacgaacuucccagcucggcU..	18	1	c02
.................................cccggacgaacuucccagcucggccU...	1	1	c02
.................................cccggacgaacuucccagcucggccgG..	1	1	c02
..................................ccggacgaacuucccagc..	18	0	c02
..................................ccggacgaacuucccagcu...	25	0	c02
..................................ccggCcgaacuucccagcuc..	1	1	c02
..................................ccggacgaacuucccagcuA..	1	1	c02
..................................ccggacgaCcuucccagcuc..	2	1	c02
..................................ccggacgaacuucccagcuc..	73	0	c02
..................................ccggacgaacuucccCgcuc..	1	1	c02
..................................ccggacgaacuucccagcucU...	1	1	c02
..................................ccggacgaacuuAccagcucg...	1	1	c02
..................................ccggacgaacuCcccagcucg...	1	1	c02
..................................ccggacgaacuucccagcucg...	108	0	c02
..................................cUggacgaacuucccagcucg...	1	1	c02
..................................ccggacgCacuucccagcucg...	1	1	c02
..................................ccggacgaacuucAcagcucg...	1	1	c02
..................................ccggacgaacuucccCgcucgg..	1	1	c02
..................................ccggacgaacuucAcagcucgg..	1	1	c02
..................................ccggacgaacuAcccagcucgg..	1	1	c02
..................................ccggCcgaacuucccagcucgg..	3	1	c02
..................................cUggacgaacuucccagcucgg..	1	1	c02
..................................ccggacgaacuucccagcCcgg..	1	1	c02
..................................ccggaAgaacuucccagcucgg..	1	1	c02
..................................ccggacgaacuucccagcucgA..	1	1	c02
..................................ccggacgaCcuucccagcucgg..	5	1	c02
..................................ccggacgaacuucccagcucgg..	319	0	c02
..................................ccggacgaGcuucccagcucgg..	1	1	c02
..................................ccggacgaGcuucccagcucggc...	1	1	c02
..................................ccggacgaacuucccagcucggU...	5	1	c02
..................................ccggCcgaacuucccagcucggc...	1	1	c02
..................................ccggacgaacuucccagcucggc...	79	0	c02
..................................ccggacgaacuucccagcucCgcc..	1	1	c02
..................................ccggacgaacuucccagcucggcc..	58	0	c02
..................................ccggacgaacuucccagcucggcA..	4	1	c02
..................................Acggacgaacuucccagcucggcc..	1	1	c02
..................................ccggacgaCcuucccagcucggcc..	2	1	c02
..................................cUggacgaacuucccagcucggcc..	1	1	c02
..................................ccggacgaacuucccagcucggcU..	7	1	c02
..................................ccggacgaacuucccagcucggccU...	6	1	c02
..................................ccggacgaGcuucccagcucggccg...	1	1	c02
..................................ccggacgaacuucccagcucggccA...	5	1	c02
..................................ccggacgaacuucccagcucggccAa..	1	1	c02
...................................cggacgaacuucccagcucg...	1	0	c02
...................................cggacgaacuucccagcucgg..	4	0	c02
...................................cggacgaacuucAcagcucgg..	1	1	c02
...................................cggacgaacuucccagcucggc...	3	0	c02
...................................cggacgaacuucccagcucggcc..	5	0	c02
......................................acgaacuucccagcucggcc..	2	0	c02
....cgaacuucccagcucggG...	1	1	c02
....acgggucggugggaucuucguccguuug...............	1	0	c02
....cgggucggugggaucuucguccgC..................	1	1	c02
....cgggucggugggaucuucguccguu.................	2	0	c02
....cgAgucggugggaucuucguccguuu................	3	1	c02
....cUggucggugggaucuucguccguuu................	1	1	c02
....gAgucggugggaucuucguccguuu................	2	1	c02
....ggUucggugggaucuucguccguuug...............	1	1	c02
....Cucggugggaucuucguccguu.................	2	1	c02
....Cucggugggaucuucguccguuu................	6	1	c02
....Uucggugggaucuucguccguuu................	1	1	c02
....Aucggugggaucuucguccguuu................	3	1	c02
....ucggugggaucuucAucc....................	1	1	c02
....ucggugggaucuucgucU....................	1	1	c02
....Gcggugggaucuucgucc....................	22	1	c02
....ucgguggggCucuucgucc....................	1	1	c02
....uUggugggaucuucgucc....................	1	1	c02
....uGggugggaucuucgucc....................	1	1	c02
....ucggugggaucuuAgucc....................	1	1	c02
....ucggugggaucuucgucc....................	229	0	c02
....ucggugggaucuucgGcc....................	1	1	c02

```
                                Star                                Mature
cuggaagaaagaagaacgguaucaagugcuacgcccggacgaacuucccagcucggccgacauacacgggucggugggaucuucguccguuuguaucgcuuauacguu
......................................................................ucggugUgaucuucguccg...................	1	1	c02
......................................................................ucUgugggaucuucguccg...................	1	1	c02
......................................................................ucggugggaucuucguUcg...................	1	1	c02
......................................................................ucggugggCucuucguccg...................	2	1	c02
......................................................................ucCgugggaucuucguccg...................	2	1	c02
......................................................................Gcggugggaucuucguccg...................	49	1	c02
......................................................................ucggugggaucuucguccA...................	1	1	c02
......................................................................ucggugggaucuuUguccg...................	1	1	c02
......................................................................ucggugggaucuucUuccg...................	1	1	c02
......................................................................Ccggugggaucuucguccg...................	1	1	c02
......................................................................ucgguUggaucuucguccg...................	1	1	c02
......................................................................ucggugggaucuucgCccg...................	1	1	c02
......................................................................ucggugggaucuucguAcg...................	1	1	c02
......................................................................ucgCugggaucuucguccg...................	2	1	c02
......................................................................ucggugggaucuucguccg...................	486	0	c02
......................................................................ucggugggaucuucguccgG..................	2	1	c02
......................................................................ucggugggaucuucguccgu..................	1263	0	c02
......................................................................ucAgugggaucuucguccgu..................	2	1	c02
......................................................................ucggugggaucuuUguccgu..................	1	1	c02
......................................................................ucggugggaucuucguAcgu..................	1	1	c02
......................................................................ucggugggaucuucCuccgu..................	1	1	c02
......................................................................ucggugggaucCucguccgu..................	1	1	c02
......................................................................ucgguCggaucuucguccgu..................	1	1	c02
......................................................................ucggugggauAuucguccgu..................	2	1	c02
......................................................................uAggugggaucuucguccgu..................	3	1	c02
......................................................................ucggugggaucuucguccAu..................	1	1	c02
......................................................................ucggugggaucuuAguccgu..................	1	1	c02
......................................................................ucgUugggaucuucguccgu..................	1	1	c02
......................................................................ucUgugggaucuucguccgu..................	4	1	c02
......................................................................ucggugggCucuucguccgu..................	7	1	c02
......................................................................ucgguggCaucuucguccgu..................	1	1	c02
......................................................................ucggugUgaucuucguccgu..................	2	1	c02
......................................................................uUggugggaucuucguccgu..................	5	1	c02
......................................................................ucggugggaucuucAuccgu..................	2	1	c02
......................................................................ucggugggaAcuucguccgu..................	1	1	c02
......................................................................ucggugggaucuucgucAgu..................	3	1	c02
......................................................................ucgguggUaucuucguccgu..................	1	1	c02
......................................................................ucggAgggaucuucguccgu..................	1	1	c02
......................................................................Gcggugggaucuucguccgu..................	130	1	c02
......................................................................ucggugggaucuucgucUgu..................	3	1	c02
......................................................................ucggugggaucuucgAccgu..................	1	1	c02
......................................................................ucggugggaucuucguUcgu..................	1	1	c02
......................................................................ucggugggaucuucguccgA..................	2	1	c02
......................................................................ucggugCgaucuucguccgu..................	2	1	c02
......................................................................ucggugggaCcuucguccgu..................	1	1	c02
......................................................................ucgguAggaucuucguccgu..................	1	1	c02
......................................................................ucggGgggaucuucguccgu..................	1	1	c02
......................................................................ucgCugggaucuucguccgu..................	6	1	c02
......................................................................ucCgugggaucuucguccgu..................	1	1	c02
......................................................................ucggugggaucuAcguccguu.................	1	1	c02
......................................................................ucggugggaAcuucguccguu.................	1	1	c02
......................................................................ucgCugggaucuucguccguu.................	5	1	c02
......................................................................ucggugggaucCucguccguu.................	1	1	c02
......................................................................ucggugggaucuucgCccguu.................	1	1	c02
......................................................................ucggugggaucuucguUcguu.................	1	1	c02
......................................................................ucgguUggaucuucguccguu.................	5	1	c02
......................................................................uAggugggaucuucguccguu.................	2	1	c02
......................................................................ucCgugggaucuucguccguu.................	2	1	c02
......................................................................ucggugggaucuucCuccguu.................	1	1	c02
......................................................................uGggugggaucuucguccguu.................	1	1	c02
......................................................................ucgguggCaucuucguccguu.................	1	1	c02
......................................................................uUggugggaucuucguccguu.................	3	1	c02
......................................................................ucggugggaucuucguccUuu.................	8	1	c02
......................................................................ucgguggUaucuucguccguu.................	1	1	c02
......................................................................ucggugggaucuuAguccguu.................	3	1	c02
......................................................................ucgAugggaucuucguccguu.................	1	1	c02
......................................................................ucggugggCucuucguccguu.................	4	1	c02
......................................................................ucggugCgaucuucguccguu.................	2	1	c02
......................................................................ucggugggauAuucguccguu.................	1	1	c02
......................................................................ucgUugggaucuucguccguu.................	1	1	c02
```

Star Mature

```
cuggaagaaagaagaacgguaucaagugcuacgcccggacgaacuucccagcucggccgacauacacgggucggugggaucuucguccguuuguaucgcuuauacguu
......................................................................ucggugggaucuucguccgAu.................	2	1	c02
......................................................................Gcggugggaucuucguccguu.................	191	1	c02
......................................................................ucggugggaucuucUuccguu.................	1	1	c02
......................................................................Ccggugggaucuucguccguu.................	3	1	c02
......................................................................ucUgugggaucuucguccguu.................	4	1	c02
......................................................................ucggugggaucuucguccCuu.................	1	1	c02
......................................................................ucggugggaucuucguccguu.................	1904	0	c02
......................................................................ucggugggaucuucgucGguu.................	1	1	c02
......................................................................ucAgugggaucuucguccguu.................	2	1	c02
......................................................................ucggugggaGcuucguccguu.................	1	1	c02
......................................................................ucggugggaucuucgucAguu.................	5	1	c02
......................................................................Acggugggaucuucguccguu.................	3	1	c02
......................................................................ucggugggaucuucguccguA.................	4	1	c02
......................................................................ucggugggaucuCcguccguuu................	6	1	c02
......................................................................ucggugggaucuucguccguuu................	43055	0	c02
......................................................................ucggugggaucuucguccguuA................	47	1	c02
......................................................................ucggugggaucuucguccUuuu................	31	1	c02
......................................................................ucggugAgaucuucguccguuu................	14	1	c02
......................................................................ucggugggUucuucguccguuu................	7	1	c02
......................................................................ucggugggaucuucguUcguuu................	18	1	c02
......................................................................ucggugggaucuucCuccguuu................	36	1	c02
......................................................................ucgguggUaucuucguccguuu................	14	1	c02
......................................................................ucggugggaucuuAguccguuu................	45	1	c02
......................................................................ucgguUggaucuucguccguuu................	28	1	c02
......................................................................ucgCugggaucuucguccguuu................	72	1	c02
......................................................................ucggugUgaucuucguccguuu................	37	1	c02
......................................................................ucggugggaucuAcguccguuu................	8	1	c02
......................................................................ucggugggaucuucguccguCu................	15	1	c02
......................................................................ucggugggaucAucguccguuu................	16	1	c02
......................................................................ucggugggauAuucguccguuu................	28	1	c02
......................................................................ucgguggCaucuucguccguuu................	27	1	c02
......................................................................ucggugggauUuucguccguuu................	9	1	c02
......................................................................ucggugggaCcuucguccguuu................	8	1	c02
......................................................................ucggugggaucuucguccguuG................	9	1	c02
......................................................................ucggugggaucuucguccgGuu................	20	1	c02
......................................................................ucAgugggaucuucguccguuu................	37	1	c02
......................................................................ucggugggaucuucgCccguuu................	9	1	c02
......................................................................ucggugggaucuucguccgCuu................	15	1	c02
......................................................................ucggugggauGuucguccguuu................	4	1	c02
......................................................................ucgguCggaucuucguccguuu................	54	1	c02
......................................................................ucggCgggaucuucguccguuu................	14	1	c02
......................................................................ucggugggaucuucguGcguuu................	8	1	c02
......................................................................ucggugggaAcuucguccguuu................	13	1	c02
......................................................................ucggGgggaucuucguccguuu................	8	1	c02
......................................................................ucgguAggaucuucguccguuu................	11	1	c02
......................................................................ucgUugggaucuucguccguuu................	36	1	c02
......................................................................ucggAgggaucuucguccguuu................	32	1	c02
......................................................................Ccggugggaucuucguccguuu................	27	1	c02
......................................................................ucUgugggaucuucguccguuu................	61	1	c02
......................................................................uGggugggaucuucguccguuu................	10	1	c02
......................................................................ucggugggaucuucUuccguuu................	17	1	c02
......................................................................ucggugggaucuucguccguuC................	10	1	c02
......................................................................ucggugggaucuucAuccguuu................	13	1	c02
......................................................................ucggugggaucCucguccguuu................	18	1	c02
......................................................................Gcggugggaucuucguccguuu................	4256	1	c02
......................................................................ucggugggaGcuucguccguuu................	10	1	c02
......................................................................uUggugggaucuucguccguuu................	59	1	c02
......................................................................ucgguggAaucuucguccguuu................	2	1	c02
......................................................................ucggugggaucuucgucGguuu................	10	1	c02
......................................................................Acggugggaucuucguccguuu................	86	1	c02
......................................................................ucggugggCucuucguccguuu................	153	1	c02
......................................................................ucggugggGucuucguccguuu................	11	1	c02
......................................................................ucggugggaucuucguccgAuu................	24	1	c02
......................................................................ucggugggaucuGcguccguuu................	6	1	c02
......................................................................ucggugggaucuucgAccguuu................	22	1	c02
......................................................................ucggugggaucuucguccAuuu................	13	1	c02
......................................................................ucggugggaucuucguAcguuu................	22	1	c02
......................................................................ucggugggaucGucguccguuu................	11	1	c02
......................................................................ucgAugggaucuucguccguuu................	19	1	c02
......................................................................ucggugCgaucuucguccguuu................	42	1	c02
```

```
                               Star                                  Mature
cuggaagaaagaagaacgguaucaagugcuacgcccggacgaacuucccagcucggccgacauacacgggucggugggaucuucguccguuuguaucgcuuauacguu
.....................................................................ucggugggaucuucgGccguuu.................  12    1   c02
.....................................................................ucggugggaucuuGguccguuu.................  3     1   c02
.....................................................................ucCgugggaucuucguccguuu.................  41    1   c02
.....................................................................ucggugggaucuucguccguGu.................  11    1   c02
.....................................................................ucggugggaucuucguccCuuu.................  42    1   c02
.....................................................................ucggugggaucuucgucAguuu.................  73    1   c02
.....................................................................ucggugggaucuuUguccguuu.................  23    1   c02
.....................................................................ucggugggaucuucgucUguuu.................  42    1   c02
.....................................................................uAggugggaucuucguccguuu.................  32    1   c02
.....................................................................ucggugggaucuucguccguAu.................  17    1   c02
.....................................................................ucgCugggaucuucguccguuug................  2     1   c02
.....................................................................Gcggugggaucuucguccguuug................  19    1   c02
.....................................................................ucggugggaucCucguccguuug................  1     1   c02
.....................................................................ucggugggaucuucguccguuuU................  1326  1   c02
.....................................................................ucggugggaucuucguccguuug................  237   0   c02
.....................................................................ucggugggaucuucguccguuuC................  20    1   c02
.....................................................................ucggugggauAuucguccguuug................  1     1   c02
.....................................................................ucggugggaucuucguccguuuA................  450   1   c02
.....................................................................ucggugggCucuucguccguuug................  1     1   c02
.....................................................................ucggugggaCcuucguccguuug................  1     1   c02
.....................................................................ucggugggaucuucguccguuugA...............  2     1   c02
.....................................................................ucggugggauAuucguccguuugu...............  1     1   c02
.....................................................................ucggugggaucuucguUcguuugu...............  1     1   c02
.....................................................................ucggugggaucuucguccguuuAu...............  11    1   c02
.....................................................................ucggugggaucuucguccguuugu...............  53    0   c02
.....................................................................ucggugggaucuucguccguuGgu...............  1     1   c02
.....................................................................ucggugggaucuucguccguuuUu...............  50    1   c02
.....................................................................ucggugggaucuucguccguuuCu...............  3     1   c02
.....................................................................ucggugggaucuucguccguuugC...............  3     1   c02
.....................................................................Gcggugggaucuucguccguuugu...............  4     1   c02
.....................................................................ucggugggaucuucguccguuugG...............  10    1   c02
.....................................................................ucggugggaucuucguccguuugGa..............  1     1   c02
.....................................................................ucggugggaucuucguccguuuguU..............  1     1   c02
.....................................................................ucggugggaucuucguccguuuguC..............  1     1   c02
.....................................................................ucggugggaucuucguccguuugua..............  2     0   c02
.....................................................................ucggugggaucuucguccguuuUuau.............  1     1   c02
.....................................................................ucggugggaucuucguccguuuguau.............  2     0   c02
......................................................................cggugggaucuucguccg....................  4     0   c02
......................................................................cggugggaucuucguccgu...................  12    0   c02
......................................................................cgCugggaucuucguccguu..................  1     1   c02
......................................................................cggugggaucuucguccguu..................  12    0   c02
......................................................................cggugggaucuucCuccguuu.................  1     1   c02
......................................................................Uggugggaucuucguccguuu.................  1     1   c02
......................................................................cgguUggaucuucguccguuu.................  1     1   c02
......................................................................cAgugggaucuucguccguuu.................  1     1   c02
......................................................................cggugggaucuucguccUuuu.................  1     1   c02
......................................................................cggugggaucuucguccguuG.................  1     1   c02
......................................................................cggugggaucuuUguccguuu.................  1     1   c02
......................................................................cggugggaucuucguccguuu.................  340   0   c02
......................................................................cggugggaucuucguccguuA.................  1     1   c02
......................................................................cggugggaucuucguccguuug................  173   0   c02
......................................................................cggugggaucuucguccguuuC................  1     1   c02
......................................................................cggugggaucuucguccguuuU................  48    1   c02
......................................................................cggugggaucuucguccguuuA................  12    1   c02
......................................................................cggugggaucuucguccguuugA...............  3     1   c02
......................................................................cggugggaucuucguccguuugu...............  20    0   c02
......................................................................cggugggaucuucguccguuuUu...............  1     1   c02
......................................................................cggugggaucuucguccguuugua..............  5     0   c02
......................................................................cggugggaucuucguccguuuguaA.............  3     1   c02
......................................................................cggugggaucuucguccguuuguauU............  1     1   c02
.......................................................................ggugggaucuucguccgu...................  1     0   c02
.......................................................................ggugggaucuucguccguu..................  1     0   c02
.......................................................................Ugugggaucuucguccguuu.................  1     1   c02
.......................................................................ggugggaucuucguccguuu.................  25    0   c02
.......................................................................gUugggaucuucguccguuu.................  1     1   c02
........................................................................gugggaucuucguccguu..................  5     0   c02
........................................................................gugggaucuucguccguuu.................  40    0   c02
........................................................................Uugggaucuucguccguuu.................  1     1   c02
........................................................................gugAgaucuucguccguuu.................  1     1   c02
........................................................................gugggaucuucguccguGu.................  1     1   c02
```

Star **Mature**

cuggaagaaagaagaacgguaucaagugcuacgcccggacgaacuucccagcucggccgacauacacgggucggugggaucuucguccguuuguaucgcuuauacguu			
....gCgggaucuucguccguuu................	1	1	c02
....gugggaucuucguAcguuu................	1	1	c02
....gugggaucuucguccguuuA...............	3	1	c02
....gugggaucuucguccguuuU...............	1	1	c02
....Ggggaucuucguccguuu................	7	1	c02
....ugggaucuucgucUguuu................	2	1	c02
....uAggaucuucguccguuu................	1	1	c02
....ugggaucuucguccguuu................	69	0	c02
....ugggaucuucguccguuuU...............	1	1	c02
...............................cgcccggacgaacuucccagcA...	1	1	c09
...............................cgcccggacgaacuucccagcu...	1	0	c09
...............................cgcccggacgaacuucccagcuc..	2	0	c09
...............................cgcccggacgaacuucccagcucgg..	13	0	c09
...............................cgcccggacgaacuucccagcucggc...	9	0	c09
...............................cgcccggacgaacuucccagcucggAc..	1	1	c09
...............................cgcccggacgaacuucccagcucggcc..	3	0	c09
................................gcccggacgaacuuccca..	1	0	c09
................................gcccggacgaacuucccag...	1	0	c09
................................gcccggacgaacuucccagc..	4	0	c09
................................gcccggacgaacuucccagcu...	4	0	c09
................................gcccggacgaacuucccagcuc..	4	0	c09
................................gcccggacgaacuucccagcucg...	3	0	c09
................................gcccggacgaacuucccagcucA...	1	1	c09
................................gcccggacgCacuucccagcucgg..	1	1	c09
................................gcccggacgaacuucccagcucgg..	12	0	c09
................................gcccggacgaacuucccagcuAggc...	1	1	c09
................................gcccggCcgaacuucccagcucggc...	1	1	c09
................................gcccggacgCacuucccagcucggc...	1	1	c09
................................gcccggacgaacuucccagcucggc...	55	0	c09
................................gcccggacgaacuucccagcucggU...	1	1	c09
................................gcccggacgaacuucccagcucggcc..	9	0	c09
................................gcccggacgaacuucccagcucggcU..	6	1	c09
................................gcccggacgaacuucccagcucggcA..	2	1	c09
................................gcccggacgaacuucccagcucggccA...	1	1	c09
.................................cccggacgaacuucccag...	2	0	c09
.................................cccggacgaacuucccaCc..	1	1	c09
.................................cccggacgaacuucccagc..	34	0	c09
.................................cccggacgaacuucccGgcu...	1	1	c09
.................................cccggacgaacuucccagcu...	32	0	c09
.................................cccggacgaacuucccagcuc..	76	0	c09
.................................cccggacgaacuucccaCcuc..	1	1	c09
.................................cccggacgaacuucccagcuU..	1	1	c09
.................................cccggacgaacuucccagcuA..	1	1	c09
.................................cccggacgaCcuucccagcuc..	1	1	c09
.................................cccggacgaacuucccagcucC...	1	1	c09
.................................cccggacCaacuucccagcucg...	1	1	c09
.................................cAcggacgaacuucccagcucg...	1	1	c09
.................................cccggacgaacuucccagcucU...	3	1	c09
.................................cccggCcgaacuucccagcucg...	2	1	c09
.................................cccggacgCacuucccagcucg...	1	1	c09
.................................cccggacgaCcuucccagcucg...	4	1	c09
.................................cccggacgaacuucccagcuAg...	1	1	c09
.................................cccggacgaacuucccagcucg...	261	0	c09
.................................cccggacgaCcuucccagcucgg..	12	1	c09
.................................cccggacgaacuucccagcucgU..	3	1	c09
.................................cAcggacgaacuucccagcucgg..	1	1	c09
.................................ccGggacgaacuucccagcucgg..	1	1	c09
.................................cccggacgaacAucccagcucgg..	1	1	c09
.................................cccggacgaacuucGcagcucgg..	1	1	c09
.................................ccUggacgaacuucccagcucgg..	1	1	c09
.................................cccUgacgaacuucccagcucgg..	1	1	c09
.................................cccgCacgaacuucccagcucgg..	1	1	c09
.................................cccggacgaacuuccAagcucgg..	1	1	c09
.................................cccggacgGacuucccagcucgg..	1	1	c09
.................................cccgUacgaacuucccagcucgg..	1	1	c09
.................................cccggacgaacuucccagcucgA..	1	1	c09
.................................cccggacgaacuucccagcuAgg..	1	1	c09
.................................cccggacgaacuucccagUucgg..	1	1	c09
.................................cccggacgCacuucccagcucgg..	8	1	c09

Star Mature

```
cuggaagaaagaagaacgguaucaagugcuacgcccggacgaacuucccagcucggccgacauacacgggucggugggaucuucguccguuuguaucgcuuauacguu
.................................cccggacgaacuucccUgcucgg....................................................  1     1  c09
.................................cccggacgaacuucccagcucgg....................................................  1058  0  c09
.................................cccggacgaacuucccagcucgC....................................................  1     1  c09
.................................cccgAacgaacuucccagcucgg....................................................  1     1  c09
.................................cUcggacgaacuucccagcucgg....................................................  2     1  c09
.................................cccggCcgaacuucccagcucgg....................................................  5     1  c09
.................................cccggacgaacuucccagAucgg....................................................  1     1  c09
.................................cccggacgaacuucccCgcucgg....................................................  2     1  c09
.................................ccAggacgaacuucccagcucgg....................................................  2     1  c09
.................................cccggacgaacuucccaUcucggc...................................................  1     1  c09
.................................cccggacgaCcuucccagcucggc...................................................  1     1  c09
.................................cccggacgaacuucccagcuUggc...................................................  1     1  c09
.................................cccggacgaacuucccagcucggc...................................................  771   0  c09
.................................ccAggacgaacuucccagcucggc...................................................  2     1  c09
.................................cccggacgaacuucccaCcucggc...................................................  2     1  c09
.................................cAcggacgaacuucccagcucggc...................................................  1     1  c09
.................................cccgUacgaacuucccagcucggc...................................................  1     1  c09
.................................cccggacgaacuuccGagcucggc...................................................  1     1  c09
.................................cccAgacgaacuucccagcucggc...................................................  1     1  c09
.................................cccggacgaacuucccagcucUgc...................................................  1     1  c09
.................................cccggacgaacuucAcagcucggc...................................................  1     1  c09
.................................cccggacgaacuucccagcucggA...................................................  6     1  c09
.................................cccggaUgaacuucccagcucggc...................................................  1     1  c09
.................................cccggacgaacuuAccagcucggc...................................................  1     1  c09
.................................cccggacgaacuucccCgcucggc...................................................  1     1  c09
.................................cccggacgaacuucccagcuAggc...................................................  1     1  c09
.................................cccggacgCacuucccagcucggc...................................................  9     1  c09
.................................cccggacgaacuucccagcucggU...................................................  12    1  c09
.................................cccggacgaacuucccagcucggG...................................................  4     1  c09
.................................cccgAacgaacuucccagcucggc...................................................  1     1  c09
.................................cccggacgaacuucccagcucggcG..................................................  1     1  c09
.................................cccggacgaacuucccagcucggcA..................................................  52    1  c09
.................................cccggacgaacuucccagcucggcc..................................................  5     0  c09
.................................cccggacgaacuucccagcucggcU..................................................  58    1  c09
.................................cccggacgaacuucccagcucggccU.................................................  1     1  c09
.................................cccggacgaacuucccagcucggccA.................................................  1     1  c09
..................................Ucggacgaacuucccagc........................................................  1     1  c09
..................................ccggacgaacuucccagc........................................................  11    0  c09
..................................ccggacgaacuucccagcu.......................................................  27    0  c09
..................................ccggacgCacuucccagcu.......................................................  1     1  c09
..................................ccggacgaacuucccagcuA......................................................  1     1  c09
..................................ccggCcgaacuucccagcuc......................................................  1     1  c09
..................................ccggacgCacuucccagcuc......................................................  1     1  c09
..................................ccggacgaacuucccagcuc......................................................  22    0  c09
..................................ccggacgaacuuGccagcucg.....................................................  1     1  c09
..................................ccggacgaacuucccagcucC.....................................................  1     1  c09
..................................ccggacgaacuucAcagcucg.....................................................  1     1  c09
..................................ccggacgaacuucccCgcucg.....................................................  1     1  c09
..................................ccggacgaacuucccaCcucg.....................................................  1     1  c09
..................................ccggacgaacuucccagcucA.....................................................  1     1  c09
..................................ccggacgaacuucccagcucg.....................................................  108   0  c09
..................................ccggacgaCcuucccagcucgg....................................................  2     1  c09
..................................ccggacgaacuucccagcucgU....................................................  2     1  c09
..................................ccCgacgaacuucccagcucgg....................................................  1     1  c09
..................................ccggacgaacuucccagcucUg....................................................  1     1  c09
..................................ccggacgaacuAcccagcucgg....................................................  1     1  c09
..................................ccggacgaacuucccagcucCg....................................................  1     1  c09
..................................Ucggacgaacuucccagcucgg....................................................  1     1  c09
..................................ccggCcgaacuucccagcucgg....................................................  1     1  c09
..................................ccggacgaaUuucccagcucgg....................................................  1     1  c09
..................................ccggacgaacuucccagcucgg....................................................  224   0  c09
..................................ccggacgaacuucccagcucggc...................................................  131   0  c09
..................................ccggacgaCcuucccagcucggc...................................................  2     1  c09
..................................ccggCcgaacuucccagcucggc...................................................  1     1  c09
..................................ccggacgaacuAcccagcucggc...................................................  2     1  c09
..................................ccggacgaUcuucccagcucggc...................................................  1     1  c09
..................................ccggacgaacuucccagcucggU...................................................  5     1  c09
..................................ccggacgaacuucccaAcucggc...................................................  1     1  c09
..................................ccggacgaacuucccagcucggA...................................................  3     1  c09
..................................ccAgacgaacuucccagcucggcc..................................................  1     1  c09
```

```
                                 Star                          Mature
cuggaagaaagaagaacgguaucaagugcuacgcccggacgaacuucccagcucggccgacauacacgggucggugggaucuucguccguuuguaucgcuuauacguu
..................................cUggacgaacuucccagcucggcc..................................................	1	1	c09
..................................ccggacgaacuucAcagcucggcc..................................................	1	1	c09
..................................ccggacgaacuucccagcucggcU..................................................	11	1	c09
..................................ccggacgaacuucccagcucggcA..................................................	5	1	c09
..................................ccggacgaacuucccagcuUggcc..................................................	1	1	c09
..................................ccggacgaacuucccagcucUgcc..................................................	1	1	c09
..................................ccggacgaacuucccagcucggcc..................................................	66	0	c09
..................................ccggacgGacuucccagcucggcc..................................................	1	1	c09
..................................ccggacgaacuucccagcucggccA.................................................	24	1	c09
..................................ccggacgaacuucccagcucggccU.................................................	53	1	c09
..................................ccggacgaacuucccagcucggccAa................................................	1	1	c09
..................................ccggacgaacuucccagcucggccUa................................................	2	1	c09
..................................ccggacgaacuucccagcucggccgac...............................................	1	0	c09
..................................ccggacgaacuucccagcucggccgacau.............................................	1	0	c09
..................................ccggacgaacuucccagcucggccgacauacacggg......................................	1	0	c09
...................................cggacgaacuucccagcucg.....................................................	2	0	c09
...................................cggacgaacuucccagcucgg....................................................	11	0	c09
...................................cggacgaacuucccagcucggc...................................................	6	0	c09
...................................cggacgaacuucccagcucCgcc..................................................	2	1	c09
...................................cggacgaacuucccagcucggcc..................................................	11	0	c09
...................................cggacgaacuucccagcucggccA.................................................	1	1	c09
...................................cggacgaacuucccagcucggccgaca..............................................	1	0	c09
...................................cggacgaacuucccagcucggccgacauaca..........................................	1	0	c09
....................................ggacgaacuucccagcuc......................................................	1	0	c09
....................................Agacgaacuucccagcuc......................................................	1	1	c09
....................................Cgacgaacuucccagcucggcc..................................................	1	1	c09
....................................ggacgaacuucccagcucggcc..................................................	3	0	c09
....................................ggacgaacuucccagcucggcA..................................................	1	1	c09
.....................................gacgaacuucccagcucgg....................................................	2	0	c09
.....................................gacgaacuucccagcucggc...................................................	1	0	c09
.....................................gacgaacuucccagcucggcc..................................................	2	0	c09
.....................................gacgaacuucccagcucggccgacauacacgggucgg..................................	1	0	c09
......................................acgaacuucccagcucgg....................................................	2	0	c09
......................................Ccgaacuucccagcucgg....................................................	1	1	c09
......................................acgaacuucccagcucggc...................................................	2	0	c09
......................................acgaacuucccagcucggcc..................................................	1	0	c09
......................................acgaacuucccagcucggccgac...............................................	1	0	c09
..........................................................gacauacacgggucggugggauc...........................	1	0	c09
.............................................................auacacgggucggugggaucuucguccguuu................	1	0	c09
................................................................cacgggucggugggaucuucguccguuu................	1	0	c09
................................................................cCcgggucggugggaucuucguccguuu................	1	1	c09
.................................................................acgggucggugggaucuucguccgu..................	1	0	c09
.................................................................acgggucggugggaucuucguccguuu................	1	0	c09
..................................................................cCggucggugggaucuucguc.....................	1	1	c09
..................................................................cgggucggugggaucuucgucc....................	2	0	c09
..................................................................cUggucggugggaucuucgucc....................	1	1	c09
..................................................................cgggucggugggaucuucguccg...................	2	0	c09
..................................................................cgggucggugggaucuucguccgu..................	3	0	c09
..................................................................cgggucggugggaucuucCuccgu..................	1	1	c09
..................................................................cgggucggugggaucuucguccguu.................	2	0	c09
..................................................................cgggucggugggaucuucguccguA.................	2	1	c09
..................................................................cggCucggugggaucuucguccguu.................	1	1	c09
..................................................................cggUucggugggaucuucguccguu.................	1	1	c09
..................................................................cggAucggugggaucuucguccguuu................	2	1	c09
..................................................................cggCucggugggaucuucguccguuu................	3	1	c09
..................................................................cUggucggugggaucuucguccguuu................	5	1	c09
..................................................................cCggucggugggaucuucguccguuu................	13	1	c09
..................................................................cgggucggugggaucuucguccguuu................	6	0	c09
..................................................................cgCgucggugggaucuucguccguuu................	1	1	c09
..................................................................cgggucggugggaucuucguccguuugAa.............	1	1	c09
...................................................................gggucggugggaucuucgu.......................	1	0	c09
...................................................................gUgucggugggaucuucguc.....................	2	1	c09
...................................................................Cggucggugggaucuucguccgu..................	2	1	c09
...................................................................gggucggugggaucuucguccguu.................	1	0	c09
...................................................................Aggucggugggaucuucguccguu.................	1	1	c09
...................................................................gUgucggugggaucuucguccguuu................	2	1	c09
...................................................................gAgucggugggaucuucguccguuu................	3	1	c09
...................................................................Cggucggugggaucuucguccguuu................	7	1	c09
...................................................................Aggucggugggaucuucguccguuu................	3	1	c09
...................................................................ggCucggugggaucuucguccguuu................	1	1	c09
```

Star **Mature**

```
cuggaagaaagaagaacgguaucaagugcuacgcccggacgaacuucccagcucggccgacauacacgggucggugggaucuucguccguuuguaucgcuuauacguu
...................................................................ggUucggugggaucuucguccguuu................    4      1      c09
...................................................................gggucggugggaucuucguccguuu................    6      0      c09
....................................................................gCucggugggaucuucguccg...................    1      1      c09
....................................................................ggucggugggaucuucguccgu..................    1      0      c09
....................................................................Cgucggugggaucuucguccgu..................    2      1      c09
....................................................................Cgucggugggaucuucguccguu.................    1      1      c09
....................................................................ggucggugggaucuucguccguu.................    1      0      c09
....................................................................Ugucggugggaucuucguccguu.................    1      1      c09
....................................................................ggucggugggaucuucguccguuu................    6      0      c09
....................................................................gUucggugggaucuucguccguuu................    2      1      c09
....................................................................Agucggugggaucuucguccguuu................    2      1      c09
....................................................................Cgucggugggaucuucguccguuu................    28     1      c09
....................................................................ggucggugggaucuucgucAguuu................    1      1      c09
.....................................................................Cucggugggaucuucguc.....................    1      1      c09
.....................................................................Aucggugggaucuucgucc....................    1      1      c09
.....................................................................Cucggugggaucuucguccgu..................    4      1      c09
.....................................................................gucggugggaucuucguccgu..................    2      0      c09
.....................................................................Cucggugggaucuucguccguu.................    6      1      c09
.....................................................................gucggugggaucuucguccguA.................    1      1      c09
.....................................................................Uucggugggaucuucguccguu.................    1      1      c09
.....................................................................Cucggugggaucuucguccguuu................    63     1      c09
.....................................................................Aucggugggaucuucguccguuu................    4      1      c09
.....................................................................Uucggugggaucuucguccguuu................    1      1      c09
.....................................................................Cucggugggaucuucguccguuug...............    1      1      c09
......................................................................ucggugggaucuucgucU....................    9      1      c09
......................................................................ucgCugggaucuucgucc....................    2      1      c09
......................................................................ucUgugggaucuucgucc....................    2      1      c09
......................................................................Acggugggaucuucgucc....................    2      1      c09
......................................................................ucggugggaucuucgucG....................    3      1      c09
......................................................................uUggugggaucuucgucc....................    3      1      c09
......................................................................ucgguUggaucuucgucc....................    3      1      c09
......................................................................ucgguAggaucuucgucc....................    1      1      c09
......................................................................ucggugggaGcuucgucc....................    1      1      c09
......................................................................uAggugggaucuucgucc....................    2      1      c09
......................................................................ucggugUgaucuucgucc....................    2      1      c09
......................................................................ucggugggaucuucguUc....................    4      1      c09
......................................................................ucgUugggaucuucgucc....................    1      1      c09
......................................................................ucggugggaucuCcgucc....................    1      1      c09
......................................................................ucggugggauAuucgucc....................    3      1      c09
......................................................................ucggugggaucuucguAc....................    2      1      c09
......................................................................ucggugggaucuucUucc....................    4      1      c09
......................................................................ucggugAgaucuucgucc....................    1      1      c09
......................................................................ucggugggaucuuAgucc....................    3      1      c09
......................................................................ucggugggUucuucgucc....................    1      1      c09
......................................................................ucggugggaCcuucgucc....................    1      1      c09
......................................................................ucggugggaucuucguGc....................    1      1      c09
......................................................................ucggugggaucuuUgucc....................    1      1      c09
......................................................................ucggugggaucuucgCcc....................    1      1      c09
......................................................................ucggugggaucuucgucA....................    13     1      c09
......................................................................Gcggugggaucuucgucc....................    185    1      c09
......................................................................ucggugggaucAucgucc....................    1      1      c09
......................................................................ucAgugggaucuucgucc....................    3      1      c09
......................................................................ucggugggaucuucgGcc....................    3      1      c09
......................................................................ucggAgggaucuucgucc....................    2      1      c09
......................................................................ucggugggaucuucgAcc....................    2      1      c09
......................................................................ucggugggaucuucCucc....................    1      1      c09
......................................................................ucgguggUaucuucgucc....................    1      1      c09
......................................................................ucggugggCucuucgucc....................    2      1      c09
......................................................................ucggugggaucCucgucc....................    1      1      c09
......................................................................ucggugggaucuucgucc....................    2449   0      c09
......................................................................Ccggugggaucuucgucc....................    1      1      c09
......................................................................ucgAugggaucuucguccg...................    1      1      c09
......................................................................Gcggugggaucuucguccg...................    170    1      c09
......................................................................ucggugAgaucuucguccg...................    1      1      c09
......................................................................ucgguggUaucuucguccg...................    1      1      c09
......................................................................ucgguggAaucuucguccg...................    2      1      c09
......................................................................ucggAgggaucuucguccg...................    2      1      c09
......................................................................ucggugggaucuucgucUg...................    5      1      c09
......................................................................ucggugggaucCucguccg...................    1      1      c09
......................................................................uUggugggaucuucguccg...................    4      1      c09
```

```
                  Star                        Mature
cuggaagaaagaagaacgguaucaagugcuacgcccggacgaacuucccagcucggccgacauacacgggucggugggaucuucguccguuuguaucgcuuauacguu
......................................................................ucggugggaucuucguccA...................   10     1     c09
......................................................................ucggugggaucuucguAcg...................   2      1     c09
......................................................................ucggugggaucAucguccg...................   1      1     c09
......................................................................ucgguggGCucuucguccg...................   7      1     c09
......................................................................ucggugggaucuucgucAg...................   4      1     c09
......................................................................ucgUugggaucuucguccg...................   1      1     c09
......................................................................ucggugggaucuucCuccg...................   3      1     c09
......................................................................ucggugggaucuGcguccg...................   1      1     c09
......................................................................ucAgugggaucuucguccg...................   2      1     c09
......................................................................Ccggugggaucuucguccg...................   1      1     c09
......................................................................ucggugggaucuucguccU...................   27     1     c09
......................................................................Acggugggaucuucguccg...................   7      1     c09
......................................................................ucUgugggaucuucguccg...................   3      1     c09
......................................................................ucggugggaucuucgAccg...................   1      1     c09
......................................................................ucggugggaucuucguUcg...................   2      1     c09
......................................................................ucggugggaucuucguccg...................   2353   0     c09
......................................................................ucggugggaucuucguccC...................   1      1     c09
......................................................................ucgguAggaucuucguccg...................   1      1     c09
......................................................................ucggugggaucuuUguccg...................   2      1     c09
......................................................................ucggugUgaucuucguccg...................   1      1     c09
......................................................................ucggugggaucuAcguccg...................   2      1     c09
......................................................................ucggugggaucuuAguccg...................   3      1     c09
......................................................................ucggugggaucuucUuccg...................   2      1     c09
......................................................................ucgguCggaucuucguccg...................   5      1     c09
......................................................................uGggugggaucuucguccg...................   2      1     c09
......................................................................uAggugggaucuucguccg...................   1      1     c09
......................................................................ucggugggaucGucguccg...................   1      1     c09
......................................................................ucgCugggaucuucguccg...................   1      1     c09
......................................................................ucggCgggaucuucguccg...................   2      1     c09
......................................................................ucggugggauUuucguccg...................   1      1     c09
......................................................................ucggugggauAuucguccg...................   2      1     c09
......................................................................ucggugggaAcuucguccg...................   2      1     c09
......................................................................ucCgugggaucuucguccg...................   1      1     c09
......................................................................ucggugggaucuucAuccgu..................   1      1     c09
......................................................................ucgAugggaucuucguccgu..................   1      1     c09
......................................................................ucggugAgaucuucguccgu..................   4      1     c09
......................................................................ucggGgggaucuucguccgu..................   1      1     c09
......................................................................ucggugggaucuuUguccgu..................   3      1     c09
......................................................................ucgguggGCucuucguccgu..................   8      1     c09
......................................................................ucggugggaucCucguccgu..................   3      1     c09
......................................................................ucggugggaucuucgAccgu..................   1      1     c09
......................................................................ucggugggaucuucgucAgu..................   3      1     c09
......................................................................ucggugCgaucuucguccgu..................   1      1     c09
......................................................................ucggugggaucuucguAcgu..................   1      1     c09
......................................................................ucggugggaucuucUuccgu..................   3      1     c09
......................................................................ucggAgggaucuucguccgu..................   4      1     c09
......................................................................ucggugggaucuucguccUu..................   2      1     c09
......................................................................uAggugggaucuucguccgu..................   4      1     c09
......................................................................ucggugggauAuucguccgu..................   4      1     c09
......................................................................ucAgugggaucuucguccgu..................   3      1     c09
......................................................................ucggugggaucuucguccAu..................   3      1     c09
......................................................................ucgguCggaucuucguccgu..................   4      1     c09
......................................................................ucggugggaucuucgucUgu..................   5      1     c09
......................................................................ucggugggaucuucguccgC..................   8      1     c09
......................................................................ucgguggGGucuucguccgu..................   1      1     c09
......................................................................Ccggugggaucuucguccgu..................   7      1     c09
......................................................................ucggugggaucuucCuccgu..................   5      1     c09
......................................................................ucggugggaucuAcguccgu..................   2      1     c09
......................................................................ucggugUgaucuucguccgu..................   2      1     c09
......................................................................ucgguUggaucuucguccgu..................   5      1     c09
......................................................................ucgCugggaucuucguccgu..................   6      1     c09
......................................................................ucggugggaucuucguccgu..................   4520   0     c09
......................................................................ucggugggaucuucguGcgu..................   1      1     c09
......................................................................ucggugggaucuucguccgA..................   7      1     c09
......................................................................Acggugggaucuucguccgu..................   11     1     c09
......................................................................ucggugggaucuucguccgG..................   9      1     c09
......................................................................ucggugggaucuCcguccgu..................   2      1     c09
......................................................................ucggugggaucGucguccgu..................   1      1     c09
......................................................................uUggugggaucuucguccgu..................   11     1     c09
......................................................................ucUgugggaucuucguccgu..................   3      1     c09
```

Star Mature

```
cuggaagaaagaagaacgguaucaagugcuacgcccggacgaacuucccagcucggccgacauacacgggucggugggaucuucguccguuuguaucgcuuauacguu
......................................................................ucgUugggaucuucguccgu..................	4	1	c09
......................................................................ucggugggaucAucguccgu..................	2	1	c09
......................................................................ucggugggaucuuAguccgu..................	4	1	c09
......................................................................ucCgugggaucuucguccgu..................	4	1	c09
......................................................................ucggugggaucuucguUcgu..................	1	1	c09
......................................................................ucggugggaucuucguccCu..................	3	1	c09
......................................................................Gcggugggaucuucguccgu..................	332	1	c09
......................................................................ucggCgggaucuucguccgu..................	1	1	c09
......................................................................ucggugggaucuucguccUuu.................	18	1	c09
......................................................................ucggugUgaucuucguccguu.................	3	1	c09
......................................................................ucggugAgaucuucguccguu.................	2	1	c09
......................................................................ucgguggGaGcuucguccguu.................	2	1	c09
......................................................................ucggugggaucuucguccAuu.................	2	1	c09
......................................................................ucggugggaucuucguccguC.................	14	1	c09
......................................................................ucggugggaucuAcguccguu.................	4	1	c09
......................................................................ucggugggaucuucguGcguu.................	1	1	c09
......................................................................ucggugggaucuuAguccguu.................	5	1	c09
......................................................................ucAgugggaucuucguccguu.................	9	1	c09
......................................................................ucgguUggaucuucguccguu.................	1	1	c09
......................................................................ucggugggauAuucguccguu.................	4	1	c09
......................................................................ucggugggaucAucguccguu.................	3	1	c09
......................................................................ucgguggGucuucguccguu.................	2	1	c09
......................................................................uAggugggaucuucguccguu.................	2	1	c09
......................................................................ucggugggaucuucgCccguu.................	3	1	c09
......................................................................ucgguCggaucuucguccguu.................	4	1	c09
......................................................................ucggugggaucuucguccCuu.................	2	1	c09
......................................................................ucgguAggaucuucguccguu.................	2	1	c09
......................................................................ucggugggaucuucguccguA.................	40	1	c09
......................................................................ucggugggaucuucCuccguu.................	9	1	c09
......................................................................ucgUugggaucuucguccguu.................	1	1	c09
......................................................................ucUgugggaucuucguccguu.................	5	1	c09
......................................................................ucggAgggaucuucguccguu.................	1	1	c09
......................................................................ucggugggauGuucguccguu.................	1	1	c09
......................................................................ucggugggaucuucguccgGu.................	4	1	c09
......................................................................ucgguggCucuucguccguu.................	9	1	c09
......................................................................ucggGgggaucuucguccguu.................	1	1	c09
......................................................................ucggugggaucuuUguccguu.................	4	1	c09
......................................................................ucggugggaucuucgucGguu.................	1	1	c09
......................................................................ucggugggaucuucguccguG.................	18	1	c09
......................................................................ucggugggaucuucguUcguu.................	1	1	c09
......................................................................uUggugggaucuucguccguu.................	12	1	c09
......................................................................ucCgugggaucuucguccguu.................	2	1	c09
......................................................................Gcggugggaucuucguccguu.................	488	1	c09
......................................................................ucgCugggaucuucguccguu.................	10	1	c09
......................................................................ucggugggaucuucguccgCu.................	1	1	c09
......................................................................ucggugggaucuGcguccguu.................	1	1	c09
......................................................................ucgguggGaAcuucguccguu.................	1	1	c09
......................................................................ucggugggauUuucguccguu.................	1	1	c09
......................................................................ucggugggaucuucgGccguu.................	3	1	c09
......................................................................ucggugggaucuucguccgAu.................	3	1	c09
......................................................................ucggugggaucuucgucUguu.................	7	1	c09
......................................................................ucgguggGaCcuucguccguu.................	1	1	c09
......................................................................ucggugggaucuucguAcguu.................	3	1	c09
......................................................................Ccggugggaucuucguccguu.................	3	1	c09
......................................................................ucgguggUaucuucguccguu.................	2	1	c09
......................................................................Acggugggaucuucguccguu.................	13	1	c09
......................................................................ucggugggaucCucguccguu.................	3	1	c09
......................................................................ucggugggaucuucgAccguu.................	1	1	c09
......................................................................ucgguggUucuucguccguu.................	1	1	c09
......................................................................ucgguggCaucuucguccguu.................	3	1	c09
......................................................................ucgguggAaucuucguccguu.................	1	1	c09
......................................................................ucggugggaucuucgucAguu.................	11	1	c09
......................................................................ucggugggaucuucguccguu.................	6797	0	c09
......................................................................ucgCugggaucuucguccguuu................	114	1	c09
......................................................................ucggCgggaucuucguccguuu................	26	1	c09
......................................................................Ccggugggaucuucguccguuu................	57	1	c09
......................................................................ucggugggaucuucguccgCuu................	11	1	c09
......................................................................ucgguUggaucuucguccguuu................	41	1	c09
......................................................................ucggugggaucuucguccguCu................	23	1	c09
......................................................................ucggAgggaucuucguccguuu................	56	1	c09
```

```
                               Star                                 Mature
cuggaagaaagaagaacgguaucaagugcuacgcccggacgaacuucccagcucggccgacauacacgggucggugggaucuucguccguuuguaucgcuuauacguu
......................................................................ucggugggauUuucguccguuu................   19      1    c09
......................................................................ucggugggaucuucgCccguuu................   24      1    c09
......................................................................ucggugggaucuucguccguuC................   54      1    c09
......................................................................ucggugggaucuucguccguuG................   32      1    c09
......................................................................ucggugggaucuucguAcguuu................   50      1    c09
......................................................................ucggGgggaucuucguccguuu................   10      1    c09
......................................................................ucggugggaucuucguccguuu................   82713   0    c09
......................................................................ucggugggUucuucguccguuu................   14      1    c09
......................................................................uAggugggaucuucguccguuu................   60      1    c09
......................................................................ucggugggaucuucgAccguuu................   17      1    c09
......................................................................ucggugggaucuucguccAuuu................   34      1    c09
......................................................................ucggugggaucuucgucGguuu................   11      1    c09
......................................................................ucCgugggaucuucguccguuu................   38      1    c09
......................................................................ucggugggaucuucguGcguuu................   9       1    c09
......................................................................ucggugggaucuucCuccguuu................   65      1    c09
......................................................................ucggugggaucuucguccguAu................   32      1    c09
......................................................................ucggugggaucuucgucUguuu................   55      1    c09
......................................................................ucggugAgaucuucguccguuu................   17      1    c09
......................................................................ucggugggaucGucguccguuu................   30      1    c09
......................................................................ucggugggaucuucguUcguuu................   37      1    c09
......................................................................ucggugggauGuucguccguuu................   2       1    c09
......................................................................ucggugggaucuAcguccguuu................   16      1    c09
......................................................................ucggugggaucuucguccCuuu................   89      1    c09
......................................................................ucggugCaucuucguccguuu................   28      1    c09
......................................................................uGggugggaucuucguccguuu................   10      1    c09
......................................................................ucggugggaAcuucguccguuu................   17      1    c09
......................................................................ucggugggauAuucguccguuu................   40      1    c09
......................................................................ucggugggaucuucguccUuuu................   38      1    c09
......................................................................ucggugggaucuucAuccguuu................   33      1    c09
......................................................................ucggugUgaucuucguccguuu................   45      1    c09
......................................................................ucgAugggaucuucguccguuu................   39      1    c09
......................................................................ucAgugggaucuucguccguuu................   71      1    c09
......................................................................ucgguCggaucuucguccguuu................   57      1    c09
......................................................................Acggugggaucuucguccguuu................   211     1    c09
......................................................................ucggugggaGcuucguccguuu................   15      1    c09
......................................................................Gcggugggaucuucguccguuu................   6184    1    c09
......................................................................ucggugggaucuucUuccguuu................   36      1    c09
......................................................................ucggugggaucAucguccguuu................   22      1    c09
......................................................................ucggugggaucuucguccgGuu................   31      1    c09
......................................................................ucggugCgaucuucguccguuu................   57      1    c09
......................................................................ucggugggaCcuucguccguuu................   22      1    c09
......................................................................ucgUugggaucuucguccguuu................   30      1    c09
......................................................................ucggugggCucuucguccguuu................   143     1    c09
......................................................................ucggugggaucuucguccguGu................   32      1    c09
......................................................................ucggugggaucuuUguccguuu................   51      1    c09
......................................................................ucgguggAaucuucguccguuu................   14      1    c09
......................................................................ucggugggaucuuGguccguuu................   5       1    c09
......................................................................uUggugggaucuucguccguuu................   116     1    c09
......................................................................ucggugggaucuuAguccguuu................   71      1    c09
......................................................................ucggugggaucuucguccgAuu................   28      1    c09
......................................................................ucggugggaucuGcguccguuu................   17      1    c09
......................................................................ucggugggaucuucgucAguuu................   145     1    c09
......................................................................ucggugggaucuucguccguuA................   143     1    c09
......................................................................ucgguAggaucuucguccguuu................   18      1    c09
......................................................................ucggugggaucCucguccguuu................   37      1    c09
......................................................................ucUgugggaucuucguccguuu................   63      1    c09
......................................................................ucgguggUaucuucguccguuu................   11      1    c09
......................................................................ucggugggGucuucguccguuu................   13      1    c09
......................................................................ucggugggaucuucgGccguuu................   5       1    c09
......................................................................ucggugggaucuCcguccguuu................   15      1    c09
......................................................................ucggugggaucuucguccguuuU...............   1916    1    c09
......................................................................ucggugggaucCucguccguuug...............   1       1    c09
......................................................................ucggugggaucuucguccguCug...............   1       1    c09
......................................................................uAggugggaucuucguccguuug...............   1       1    c09
......................................................................ucggugggaucuucgucUguuug...............   2       1    c09
......................................................................ucggugggaucGucguccguuug...............   1       1    c09
......................................................................Acggugggaucuucguccguuug...............   1       1    c09
......................................................................Gcggugggaucuucguccguuug...............   47      1    c09
......................................................................ucggugggaucuucguccguuCg...............   1       1    c09
......................................................................ucggugggauUuucguccguuug...............   1       1    c09
```

```
                          Star                             Mature
cuggaagaaagaagaacgguaucaagugcuacgcccggacgaacuucccagcucggccgacauacacggggucggugggaucuucguccguuuguaucgcuuauacguu
......................................................................ucggAgggaucuucguccguuug...............  1    1  c09
......................................................................ucggugggaucuucguccgCuug...............  1    1  c09
......................................................................ucggugggaucuuUguccguuug...............  2    1  c09
......................................................................ucgAugggaucuucguccguuug...............  1    1  c09
......................................................................ucggugggaucuucgucAguuug...............  1    1  c09
......................................................................ucggugCgaucuucguccguuug...............  1    1  c09
......................................................................ucggugggaucuucguccAuuug...............  1    1  c09
......................................................................ucggugggaucuucguccguuGg...............  1    1  c09
......................................................................ucgguggUaucuucguccguuug...............  1    1  c09
......................................................................ucggugggaucuucCuccguuug...............  1    1  c09
......................................................................ucggugggaucuucguccguuuA...............  688  1  c09
......................................................................ucggugggaucuucguccguGug...............  1    1  c09
......................................................................ucggGgggaucuucguccguuug...............  1    1  c09
......................................................................ucggugggauAuucguccguuug...............  2    1  c09
......................................................................ucggugggaucuucguccguuuC...............  113  1  c09
......................................................................ucgguAggaucuucguccguuug...............  1    1  c09
......................................................................ucggugggaucuucguccgGuug...............  2    1  c09
......................................................................ucggugggaucuucguccCuuug...............  1    1  c09
......................................................................ucggugggaucuucguccguuug...............  608  0  c09
......................................................................ucggugggaucuucguccUuuugu..............  1    1  c09
......................................................................ucggugggaucuucguccguuCgu..............  1    1  c09
......................................................................ucggugggaucuucguccguuugA..............  11   1  c09
......................................................................ucggugggaucuucguccguuuUu..............  80   1  c09
......................................................................ucggugggaucuucguccguuugG..............  49   1  c09
......................................................................ucggugggaucuucguccguuuCu..............  13   1  c09
......................................................................ucggugggaucuucguccguuugC..............  18   1  c09
......................................................................Acggugggaucuucguccguuugu..............  1    1  c09
......................................................................ucggugggaucuucguccguuuAu..............  10   1  c09
......................................................................ucggugggaucuucguccguuugu..............  123  0  c09
......................................................................Gcggugggaucuucguccguuugu..............  5    1  c09
......................................................................ucggugggaucuucguccguuuUua.............  4    1  c09
......................................................................Gcggugggaucuucguccguuugua.............  1    1  c09
......................................................................ucggugggaucuucguccguuuAua.............  2    1  c09
......................................................................ucggugggaucuucguccguuuguG.............  1    1  c09
......................................................................ucggugggaucuucguccguuugGa.............  3    1  c09
......................................................................ucggugggaucuucguccguuuguC.............  3    1  c09
......................................................................ucggugggaucuucguccguGugua.............  1    1  c09
......................................................................ucggugggaucuucguccguuuCua.............  2    1  c09
......................................................................ucggugggaucuucguccguuugAa.............  1    1  c09
......................................................................ucggugggaucuucguccguuugua.............  9    0  c09
......................................................................ucggugggaucuucguccguuugCa.............  3    1  c09
......................................................................ucggugggaucuucguccguuuguU.............  2    1  c09
......................................................................ucggugggaucuucguccguuuguGu............  1    1  c09
......................................................................Gcggugggaucuucguccguuuguau............  1    1  c09
......................................................................ucggugggaucuucguccguuugCau............  1    1  c09
......................................................................ucggugggaucuucguccguuuguau............  7    0  c09
......................................................................ucggugggaucuucguccguuugCauc...........  1    1  c09
......................................................................ucggugggaucuucguccguuuguauGg..........  1    1  c09
.......................................................................cggugggaucuucguccg...................  18   0  c09
.......................................................................cggugggaucuucguccgu..................  29   0  c09
.......................................................................cUgugggaucuucguccgu..................  1    1  c09
.......................................................................cggugggaucuucguccguu.................  42   0  c09
.......................................................................cCgugggaucuucguccguu.................  1    1  c09
.......................................................................cUgugggaucuucguccguu.................  1    1  c09
.......................................................................Uggugggaucuucguccguu.................  1    1  c09
.......................................................................cggugggUucuucguccguu.................  1    1  c09
.......................................................................cgguggCucuucguccguuu................  2    1  c09
.......................................................................cggugggaucuucguccguGu................  1    1  c09
.......................................................................cggugggaucuucguccAuuu................  1    1  c09
.......................................................................cgUugggaucuucguccguuu................  1    1  c09
.......................................................................cggugggaucuucguccguuC................  1    1  c09
.......................................................................cggugggaucuucguccguuu................  483  0  c09
.......................................................................cUgugggaucuucguccguuu................  1    1  c09
.......................................................................cggugggaucuucguccgCuu................  1    1  c09
.......................................................................cggugggaucuucgucAguuu................  1    1  c09
.......................................................................cggugUgaucuucguccguuu................  1    1  c09
.......................................................................cgguggUaucuucguccguuu................  1    1  c09
.......................................................................cggugggaucuucguccguuG................  1    1  c09
.......................................................................cggugggaucuucgCccguuu................  2    1  c09
.......................................................................cCgugggaucuucguccguuu................  1    1  c09
```

```
                                 Star                                  Mature
cuggaagaaagaagaacgguaucaagugcuacgcccggacgaacuucccagcucggccgacauacacgggucggugggaucuucguccguuuguaucgcuuauacguu
.......................................................................Uggugggaucuucguccguuu................	2	1	c09
.......................................................................cggugggaucuucguccguCu................	1	1	c09
.......................................................................Gggugggaucuucguccguuu................	1	1	c09
.......................................................................cggugggaucuucguccguuA................	1	1	c09
.......................................................................cAgugggaucuucguccguuu................	1	1	c09
.......................................................................cggugggaucuucguccgAuug...............	1	1	c09
.......................................................................cggugggaucuucguccguuuA...............	19	1	c09
.......................................................................cgUugggaucuucguccguuug...............	1	1	c09
.......................................................................cggugggaucuucguccguuug...............	227	0	c09
.......................................................................cggAgggaucuucguccguuug...............	1	1	c09
.......................................................................cggugggaucuucguccguuuU...............	32	1	c09
.......................................................................cggugggaucuucguccguuuC...............	2	1	c09
.......................................................................cggugggaucuucguccguuugu..............	36	0	c09
.......................................................................cggugggaucuucguccguuugG..............	1	1	c09
.......................................................................cggugggaucuucguccguuuUu..............	1	1	c09
.......................................................................cggugggaucuucguccguuugA..............	2	1	c09
.......................................................................cggugggaucuucguccCuuugu..............	1	1	c09
.......................................................................cggugggaucuucguccguuugua.............	1	0	c09
.......................................................................cggugggaucuucguccguuuguau............	3	0	c09
.......................................................................cggugggaucuucguccguuuguaA............	1	1	c09
........................................................................Agugggaucuucguccgu..................	1	1	c09
........................................................................ggugggaucuucguccgu..................	1	0	c09
........................................................................ggugggaucuucguccgA..................	1	1	c09
........................................................................ggugggaucuucguccguu.................	3	0	c09
........................................................................gCugggaucuucguccguu.................	1	1	c09
........................................................................Agugggaucuucguccguuu................	4	1	c09
........................................................................ggugggCucuucguccguuu................	1	1	c09
........................................................................gguUggaucuucguccguuu................	2	1	c09
........................................................................gCugggaucuucguccguuu................	2	1	c09
........................................................................gAugggaucuucguccguuu................	1	1	c09
........................................................................gUugggaucuucguccguuu................	4	1	c09
........................................................................ggugggaucuucguccguuu................	57	0	c09
........................................................................ggugggaucuucguccguuug...............	1	0	c09
........................................................................ggugggaucuucguccguuuUu..............	1	1	c09
.........................................................................gugggaucuucguccguu.................	13	0	c09
.........................................................................gugggaucuucguccguuA................	1	1	c09
.........................................................................Uugggaucuucguccguuu................	1	1	c09
.........................................................................gugggaucuucCuccguuu................	1	1	c09
.........................................................................gugggCucuucguccguuu................	1	1	c09
.........................................................................gugggaucuucgucAguuu................	2	1	c09
.........................................................................Augggaucuucguccguuu................	1	1	c09
.........................................................................gAgggaucuucguccguuu................	1	1	c09
.........................................................................gugggaucuucguccguuu................	206	0	c09
.........................................................................gugggaucuucUuccguuu................	1	1	c09
.........................................................................Cugggaucuucguccguuu................	4	1	c09
.........................................................................gugggaucuucguccguuug...............	2	0	c09
.........................................................................gugggaucuucguccguuuU...............	6	1	c09
..........................................................................uUggaucuucguccguuu................	1	1	c09
..........................................................................ugggaucuucguccguuu................	96	0	c09
..........................................................................ugggaucuAcguccguuu................	1	1	c09
..........................................................................ugAgaucuucguccguuu................	1	1	c09
..........................................................................uCggaucuucguccguuu................	1	1	c09
..........................................................................Ggggaucuucguccguuu................	6	1	c09
..........................................................................uAggaucuucguccguuu................	1	1	c09
..........................................................................ugggaucuucguccguuuU...............	5	1	c09
..........................................................................ugggaucuucguccguuug...............	2	0	c09
..........................................................................ugggaucuucguccguuugu..............	1	0	c09
...........................................................................gggaucuucguccguuugGa.............	1	1	c09
..............................................................................aucuucguccguuuguaA............	1	1	c09

...............................cgcccggacgaacuucccag.........................................................	1	0	c16
...............................cgcccggacgaacuucccagcu.......................................................	1	0	c16
...............................cgcccggacgaacuucccagcuc......................................................	1	0	c16
...............................cgcccggacgaacuucccagcucg.....................................................	4	0	c16
...............................cgcccggacgaacuucccagcucU.....................................................	1	1	c16
...............................cgcccggacgaacuucccagcucgg....................................................	8	0	c16
...............................cgcccggacgCacuucccagcucgg....................................................	1	1	c16
...............................cgcccggacgaacuucccagcucggc...................................................	4	0	c16
...............................Ggcccggacgaacuucccagcucggc...................................................	1	1	c16
...............................cgcccggacgaacuucccagcucggcU..................................................	4	1	c16
```

Star Mature

```
cuggaagaaagaagaacgguaucaagugcuacgcccggacgaacuucccagcucggccgacauacacgggucggugggaucuucguccguuuguaucgcuuauacguu
................................gcccggacgaacuuccca..........................................................	2	0	c16
................................gcccggacgaacuucccag.........................................................	2	0	c16
................................gcccggacgaacuucccagc........................................................	2	0	c16
................................gcccggacgaacuucccagcu.......................................................	10	0	c16
................................Ucccggacgaacuucccagcuc......................................................	2	1	c16
................................gcccggacgaCcuucccagcuc......................................................	1	1	c16
................................gcccggacgaacuucccagcuc......................................................	8	0	c16
................................gcccggacgaacuucccagcucU.....................................................	1	1	c16
................................gcccggacgaacuucccagcucg.....................................................	13	0	c16
................................gcccCgacgaacuucccagcucg.....................................................	1	1	c16
................................gcccggacgaGcuucccagcucg.....................................................	1	1	c16
................................Ucccggacgaacuucccagcucgg....................................................	1	1	c16
................................gcccggacgaacuuccAagcucgg....................................................	1	1	c16
................................gcccggacgaacuucccagcucgA....................................................	1	1	c16
................................gcccggacgaacuucccagcucgg....................................................	17	0	c16
................................gcccggacgaacuuccUagcucgg....................................................	1	1	c16
................................gcccggacgaacuucccagcucggc...................................................	17	0	c16
................................gcccggacgaacuucccagcucggG...................................................	1	1	c16
................................Ucccggacgaacuucccagcucggc...................................................	2	1	c16
................................gcccggacgaacuucccagcucggcc..................................................	1	0	c16
................................gcccggacgaacuucccagcucggcU..................................................	3	1	c16
................................gcccggacgaacuucccagcucggccU.................................................	1	1	c16
.................................cccggacgUacuucccag.........................................................	1	1	c16
.................................cccggacgaacuucccagc........................................................	3	0	c16
.................................ccUggacgaacuucccagc........................................................	1	1	c16
.................................cccggacgaacuucccagcu.......................................................	11	0	c16
.................................cccggacgaacuucccagcuc......................................................	24	0	c16
.................................cccggCcgaacuucccagcuc......................................................	1	1	c16
.................................cccggacgaacuucccCgcuc......................................................	1	1	c16
.................................cccggacgaacuucccagcucg.....................................................	76	0	c16
.................................cAcggacgaacuucccagcucg.....................................................	1	1	c16
.................................cUcggacgaacuucccagcucg.....................................................	1	1	c16
.................................cccggacgaacuuccAagcucg.....................................................	1	1	c16
.................................cccggacgaCcuucccagcucg.....................................................	1	1	c16
.................................cccggacgaacuuGccagcucg.....................................................	1	1	c16
.................................cccggacgCacuucccagcucg.....................................................	2	1	c16
.................................cccggacgaacuuccUagcucgg....................................................	1	1	c16
.................................cccggacgaacuuAccagcucgg....................................................	1	1	c16
.................................cccggaUgaacuucccagcucgg....................................................	1	1	c16
.................................cccggacgaacuucccagcucgg....................................................	162	0	c16
.................................cccggacgCacuucccagcucgg....................................................	3	1	c16
.................................cccggacgaacuucccagcucgU....................................................	3	1	c16
.................................cccggacgaacuucccagAucgg....................................................	1	1	c16
.................................cccggacgaCcuucccagcucgg....................................................	1	1	c16
.................................cccggacgaacuucccagcucUg....................................................	1	1	c16
.................................cccggacCaacuucccagcucgg....................................................	1	1	c16
.................................cccggCcgaacuucccagcucgg....................................................	1	1	c16
.................................cccggacgaacuucccGgcucgg....................................................	1	1	c16
.................................cccggacgaacuuccAagcucgg....................................................	1	1	c16
.................................cccggacgaacuucccagcucUgc...................................................	1	1	c16
.................................cccggacgaCcuucccagcucggc...................................................	3	1	c16
.................................cccggacgaacuucccagcucggU...................................................	11	1	c16
.................................cccggCcgaacuucccagcucggc...................................................	1	1	c16
.................................cccggacgCacuucccagcucggc...................................................	2	1	c16
.................................cccggacgaacuucccagcucggA...................................................	3	1	c16
.................................cccggacgUacuucccagcucggc...................................................	1	1	c16
.................................cccggacgaacuucccagcucggc...................................................	67	0	c16
.................................cccggacgaacuucccagcucggcU..................................................	17	1	c16
.................................cccggacgaacuucccagcucggcA..................................................	5	1	c16
.................................cccggacgaacuucccagcucggcGg.................................................	1	1	c16
..................................ccggCcgaacuucccagc........................................................	1	1	c16
..................................ccggacgaUcuucccagc........................................................	1	1	c16
..................................ccggacgaacuucccagc........................................................	8	0	c16
..................................ccggacgaacuucccagcu.......................................................	15	0	c16
..................................ccggaAgaacuucccagcu.......................................................	1	1	c16
..................................ccggacgaacuucccagcC.......................................................	1	1	c16
..................................ccggacgaacuucccagcuU......................................................	1	1	c16
..................................ccggacgCacuucccagcuc......................................................	1	1	c16
..................................ccggacgaacGucccagcuc......................................................	1	1	c16
..................................cAggacgaacuucccagcuc......................................................	1	1	c16
```

Star Mature

```
cuggaagaaagaagaacgguaucaagugcuacgcccggacgaacuucccagcucggccgacauacacgggucggugggaucuucguccguuuguaucgcuuauacguu
..................................ccggacgaacuucccagcuc......................................................  64   0   c16
..................................ccCgacgaacuucccagcuc......................................................  1    1   c16
..................................ccggaAgaacuucccagcucg.....................................................  2    1   c16
..................................ccggacgaacuucccagcuGg.....................................................  1    1   c16
..................................ccggacgCacuucccagcucg.....................................................  1    1   c16
..................................ccggacgaacuucccagcucg.....................................................  159  0   c16
..................................ccggacgaacuucccagcucU.....................................................  3    1   c16
..................................ccggacgaacuuGccagcucg.....................................................  1    1   c16
..................................ccggCcgaacuucccagcucg.....................................................  1    1   c16
..................................ccggacgaacuucAcagcucg.....................................................  1    1   c16
..................................ccggacgaCcuucccagcucg.....................................................  6    1   c16
..................................ccggacgaacuucccagcucgU....................................................  4    1   c16
..................................ccggacgaacuucccagcucgg....................................................  231  0   c16
..................................ccggacgaacuucAcagcucgg....................................................  1    1   c16
..................................ccggacgaCcuucccagcucgg....................................................  2    1   c16
..................................cAggacgaacuucccagcucgg....................................................  1    1   c16
..................................ccggacgaacuucccagcucUg....................................................  1    1   c16
..................................ccggCcgaacuucccagcucgg....................................................  2    1   c16
..................................ccggacgaacuucccagcucgA....................................................  2    1   c16
..................................ccggacgaacuucccagcucggU...................................................  16   1   c16
..................................ccggacgaCcuucccagcucggc...................................................  1    1   c16
..................................Ucggacgaacuucccagcucggc...................................................  1    1   c16
..................................ccggacgaacuucccagcucggc...................................................  83   0   c16
..................................ccggacgaacuucccagcucggA...................................................  1    1   c16
..................................ccggCcgaacuucccagcucggc...................................................  3    1   c16
..................................ccggacgaacuucccCgcucggc...................................................  1    1   c16
..................................ccggacgGacuucccagcucggc...................................................  1    1   c16
..................................ccggaAgaacuucccagcucggc...................................................  1    1   c16
..................................ccggacgaacuucccagcucggcA..................................................  3    1   c16
..................................ccggacAaacuucccagcucggcc..................................................  1    1   c16
..................................ccggCcgaacuucccagcucggcc..................................................  2    1   c16
..................................ccggacgaacuucccagcucggcc..................................................  32   0   c16
..................................ccggacgaacuucccagcucggcU..................................................  15   1   c16
..................................ccggacgaacuucccagcucggccA.................................................  3    1   c16
..................................ccggacgaacuucccagcucggccU.................................................  80   1   c16
...................................cggacgaacuucccagcuc......................................................  1    0   c16
...................................cggacgaacuucccagcucg.....................................................  2    0   c16
...................................cggacgaacuucccagcucgg....................................................  2    0   c16
...................................cggacgaacuucccagcucggU...................................................  1    1   c16
...................................cggacgaacuucccagcucggc...................................................  3    0   c16
...................................cggacgaacuucccagcucggcU..................................................  2    1   c16
...................................cggacgaacuucccagcucggcc..................................................  2    0   c16
...................................cggacgaacuucccagcucggccU.................................................  1    1   c16
....................................ggacgaacuucccagcuc......................................................  1    0   c16
....................................ggacgaacuucccagcucggcc..................................................  1    0   c16
.........................................aacuucccagcucggccU.................................................  1    1   c16
.................................................................aAgggucggugggaucuucguccguuu................  1    1   c16
..................................................................cCggucggugggaucuucguccgu..................  2    1   c16
..................................................................cCggucggugggaucuucguccguu.................  1    1   c16
..................................................................cUggucggugggaucuucguccguu.................  1    1   c16
..................................................................cCggucggugggaucuucguccguuu................  3    1   c16
..................................................................cUggucggugggaucuucguccguuu................  2    1   c16
..................................................................cggCucggugggaucuucguccguuu................  2    1   c16
...................................................................gAgucggugggaucuucguccguuu................  1    1   c16
...................................................................Cggucggugggaucuucguccguuu................  2    1   c16
...................................................................gggucggugggaucuucguccguuu................  2    0   c16
...................................................................Aggucggugggaucuucguccguuu................  1    1   c16
....................................................................Cgucggugggaucuucguccguu.................  2    1   c16
....................................................................Agucggugggaucuucguccguuu................  1    1   c16
....................................................................gAucggugggaucuucguccguuu................  1    1   c16
....................................................................ggucggugggaucuucguccguuu................  3    0   c16
....................................................................gUucggugggaucuucguccguuu................  2    1   c16
....................................................................gCucggugggaucuucguccguuu................  1    1   c16
....................................................................Cgucggugggaucuucguccguuu................  4    1   c16
.....................................................................Cucggugggaucuucguccguu.................  3    1   c16
.....................................................................Cucggugggaucuucguccguuu................  18   1   c16
.....................................................................Uucggugggaucuucguccguuu................  1    1   c16
......................................................................ucggugggaucuucgGcc....................  1    1   c16
......................................................................Gcggugggaucuucgucc....................  17   1   c16
......................................................................ucggugggaucuucgucc....................  144  0   c16
```

Star **Mature**

cuggaagaaagaagaacgguaucaagugcuacgcccggacgaacuucccagcucggccgacauacacgggucggugggaucuucguccguuuguaucgcuuauacguu

....ucggugggaucuucCucc....................	1	1	c16
....ucAgugggaucuucgucc....................	1	1	c16
....ucggugggaucAucgucc....................	1	1	c16
....uUggugggaucuucgucc....................	1	1	c16
....uAggugggaucuucgucc....................	3	1	c16
....ucggugggaucuucguAc....................	1	1	c16
....ucggugggaucuucCuccg...................	1	1	c16
....ucggugggaucuucguccU...................	2	1	c16
....Gcggugggaucuucguccg...................	21	1	c16
....ucggugUgaucuucguccg...................	1	1	c16
....ucggugAgaucuucguccg...................	1	1	c16
....ucgguUggaucuucguccg...................	1	1	c16
....ucggugggaucuucguccg...................	236	0	c16
....ucggugggauAuucguccg...................	1	1	c16
....ucggugggaucuucgucUg...................	1	1	c16
....ucgUugggaucuucguccgu..................	1	1	c16
....ucggugggaucuucgucAgu..................	1	1	c16
....uGggugggaucuucguccgu..................	1	1	c16
....ucgguUggaucuucguccgu..................	2	1	c16
....ucggugggaucuucguAcgu..................	2	1	c16
....ucggugggaucuucguccgA..................	1	1	c16
....ucggugggaucuAcguccgu..................	1	1	c16
....uAggugggaucuucguccgu..................	3	1	c16
....ucUgugggaucuucguccgu..................	1	1	c16
....ucggugUgaucuucguccgu..................	1	1	c16
....Gcggugggaucuucguccgu..................	88	1	c16
....Acggugggaucuucguccgu..................	2	1	c16
....ucCgugggaucuucguccgu..................	1	1	c16
....ucggugggaucuuAguccgu..................	1	1	c16
....ucggugggaucAucguccgu..................	1	1	c16
....ucAgugggaucuucguccgu..................	1	1	c16
....ucggugggaucuucguccgu..................	807	0	c16
....ucgCugggaucuucguccgu..................	1	1	c16
....ucggugggaCcuucguccgu..................	1	1	c16
....ucggugggaucuucguccUu..................	2	1	c16
....ucggugggaucuucgucUgu..................	1	1	c16
....ucggugggaucuucgCccgu..................	1	1	c16
....uUggugggaucuucguccgu..................	2	1	c16
....ucggCgggaucuucguccgu..................	1	1	c16
....ucggugggaucGucguccgu..................	1	1	c16
....ucggugggaucuuUguccguu.................	2	1	c16
....ucgguUggaucuucguccguu.................	1	1	c16
....ucggugggaucuucguccUuu.................	4	1	c16
....ucggCgggaucuucguccguu.................	1	1	c16
....ucggugggaucuucguccguA.................	1	1	c16
....ucggugggaucGucguccguu.................	1	1	c16
....ucggugggaucuucgucUguu.................	1	1	c16
....ucggAgggaucuucguccguu.................	1	1	c16
....ucggugggaucCucguccguu.................	1	1	c16
....Acggugggaucuucguccguu.................	3	1	c16
....ucgguCggaucuucguccguu.................	4	1	c16
....ucgguAggaucuucguccguu.................	1	1	c16
....ucgguggCaucuucguccguu.................	2	1	c16
....ucCgugggaucuucguccguu.................	2	1	c16
....ucggugCgaucuucguccguu.................	1	1	c16
....ucUgugggaucuucguccguu.................	1	1	c16
....ucggugggaCcuucguccguu.................	1	1	c16
....ucggugggaucuuAguccguu.................	1	1	c16
....ucggugggaucuucguAcguu.................	4	1	c16
....ucggugggaucuucguccgAu.................	1	1	c16
....ucAgugggaucuucguccguu.................	2	1	c16
....uUggugggaucuucguccguu.................	3	1	c16
....Gcggugggaucuucguccguu.................	218	1	c16
....ucgUugggaucuucguccguu.................	5	1	c16
....ucggugggaucuucgucAguu.................	2	1	c16
....uGggugggaucuucguccguu.................	2	1	c16
....ucggugggaucuucguccguu.................	2212	0	c16
....ucggugggaucuucguccguC.................	3	1	c16
....ucggugggaucuucguccCuu.................	2	1	c16
....ucggugggCucuucguccguu.................	7	1	c16

```
                                 Star                       Mature
cuggaagaaagaagaacgguaucaagugcuacgcccggacgaacuucccagcucggccgacauacacgggucggugggaucuucguccguuuguaucgcuuauacguu
......................................................................ucggugggaucuucAuccguu.................	1	1	c16
......................................................................ucggugggaucuucguccgGu.................	1	1	c16
......................................................................ucggugggauUuucguccguu.................	1	1	c16
......................................................................uAggugggaucuucguccguu.................	1	1	c16
......................................................................ucgguggAaucuucguccguu.................	1	1	c16
......................................................................ucgCugggaucuucguccguu.................	6	1	c16
......................................................................ucggugggaucuucgGccguu.................	1	1	c16
......................................................................Ccggugggaucuucguccguu.................	1	1	c16
......................................................................ucggugUgaucuucguccguu.................	5	1	c16
......................................................................ucggAgggaucuucguccguuu................	16	1	c16
......................................................................ucggugggaucuucgAccguuu................	3	1	c16
......................................................................ucggugggauUuucguccguuu................	7	1	c16
......................................................................ucUgugggaucuucguccguuu................	19	1	c16
......................................................................ucggugggaCcuucguccguuu................	2	1	c16
......................................................................ucggugggaucuucguccgGuu................	9	1	c16
......................................................................Acggugggaucuucguccguuu................	45	1	c16
......................................................................ucggugggCucuucguccguuu................	64	1	c16
......................................................................ucggugggaucuucgGccguuu................	7	1	c16
......................................................................ucggugggaucuucCuccguuu................	14	1	c16
......................................................................ucggugggaucCucguccguuu................	7	1	c16
......................................................................ucggugCgaucuucguccguuu................	16	1	c16
......................................................................ucggugggaucuucgucGguuu................	3	1	c16
......................................................................ucggugggaucuuAguccguuu................	23	1	c16
......................................................................uGggugggaucuucguccguuu................	2	1	c16
......................................................................ucggugggaucuGcguccguuu................	3	1	c16
......................................................................ucggugggaucuucguccguuu................	18866	0	c16
......................................................................ucCgugggaucuucguccguuu................	17	1	c16
......................................................................ucggCgggaucuucguccguuu................	6	1	c16
......................................................................ucggugggaucuucguGcguuu................	3	1	c16
......................................................................ucggugggaucAucguccguuu................	7	1	c16
......................................................................uAggugggaucuucguccguuu................	17	1	c16
......................................................................ucggugggaucuucAuccguuu................	5	1	c16
......................................................................ucggugAgaucuucguccguuu................	2	1	c16
......................................................................ucggugggaucuucguccgCuu................	3	1	c16
......................................................................ucggugggauGuucguccguuu................	1	1	c16
......................................................................ucggugggUucuucguccguuu................	1	1	c16
......................................................................ucggugggaAcuucguccguuu................	7	1	c16
......................................................................Ccggugggaucuucguccguuu................	15	1	c16
......................................................................ucggugggaucuucguccAuuu................	5	1	c16
......................................................................ucgguCggaucuucguccguuu................	22	1	c16
......................................................................ucgUugggaucuucguccguuu................	10	1	c16
......................................................................ucggugggaucuucguccguCu................	6	1	c16
......................................................................ucggugggGucuucguccguuu................	1	1	c16
......................................................................ucggugggaucuucguAcguuu................	20	1	c16
......................................................................ucggGgggaucuucguccguuu................	1	1	c16
......................................................................ucggugggaucuucgCccguuu................	7	1	c16
......................................................................ucgguggAaucuucguccguuu................	4	1	c16
......................................................................ucggugggaucuucguccguuG................	10	1	c16
......................................................................ucggugggaucuucguccguuA................	36	1	c16
......................................................................ucggugggaucuAcguccguuu................	9	1	c16
......................................................................ucggugggauAuucguccguuu................	8	1	c16
......................................................................ucggugggaucuucguccguGu................	14	1	c16
......................................................................ucggugggaucGucguccguuu................	4	1	c16
......................................................................ucggugggaucuCcguccguuu................	1	1	c16
......................................................................ucggugggaucuucguccCuuu................	17	1	c16
......................................................................ucgAugggaucuucguccguuu................	5	1	c16
......................................................................ucgguAggaucuucguccguuu................	2	1	c16
......................................................................ucgCugggaucuucguccguuu................	33	1	c16
......................................................................ucgguUggaucuucguccguuu................	7	1	c16
......................................................................uUggugggaucuucguccguuu................	23	1	c16
......................................................................ucAgugggaucuucguccguuu................	14	1	c16
......................................................................ucgguggUaucuucguccguuu................	3	1	c16
......................................................................ucggugggaucuucguccUuuu................	19	1	c16
......................................................................ucggugggaucuucgucAguuu................	28	1	c16
......................................................................ucggugggaucuucUuccguuu................	13	1	c16
......................................................................ucggugggaucuucguUcguuu................	5	1	c16
......................................................................ucggugggaucuucguccguAu................	12	1	c16
......................................................................ucgguggCaucuucguccguuu................	17	1	c16
......................................................................ucggugggaucuucguccguuC................	8	1	c16
......................................................................ucggugggaucuucguccgAuu................	8	1	c16
```

Star   Mature

```
cuggaagaaagaagaacgguaucaagugcuacgcccggacgaacuucccagcucggccgacauacacgggucggugggaucuucguccguuuguaucgcuuauacguu
......................................................................ucggugggaucuuUguccguuu................	18	1	c16
......................................................................Gcggugggaucuucguccguuu................	1870	1	c16
......................................................................ucggugggaucuuGguccguuu................	4	1	c16
......................................................................ucggugUgaucuucguccguuu................	15	1	c16
......................................................................ucggugggaucuucgucUguuu................	11	1	c16
......................................................................ucggugggaGcuucguccguuu................	6	1	c16
......................................................................ucggugggaucuucguccguuGg...............	1	1	c16
......................................................................ucggugggaucuucguccguuuC...............	23	1	c16
......................................................................ucggugggaucuucguccCuuug...............	1	1	c16
......................................................................Gcggugggaucuucguccguuug...............	8	1	c16
......................................................................ucggugggaucuucguccguuuU...............	2384	1	c16
......................................................................ucggugggaucuucguccguuuA...............	96	1	c16
......................................................................ucggugggaucuucguccguuug...............	71	0	c16
......................................................................ucggugggaucuucguccguuuUu..............	256	1	c16
......................................................................ucggugggaucuucguccguuugA..............	2	1	c16
......................................................................ucggugggaucuucguccguuuAu..............	10	1	c16
......................................................................ucggugggaucuucguccguuugG..............	3	1	c16
......................................................................ucggugggaucuucguccguuugu..............	22	0	c16
......................................................................ucggugggaucuucguccguuuCu..............	10	1	c16
......................................................................ucggugggaucuucguccguuugC..............	1	1	c16
......................................................................Gcggugggaucuucguccguuugu..............	2	1	c16
......................................................................ucggugggaucuucguccguuAgua.............	1	1	c16
......................................................................ucggugggaucuucguccUuuugua.............	1	1	c16
......................................................................ucggugggaucuucguccguuugCa.............	1	1	c16
......................................................................ucggugggaucuucguccguuuUua.............	4	1	c16
......................................................................ucggugggaucuucguccguuugua.............	1	0	c16
......................................................................ucggugggaucuucguccguuuguU............	2	1	c16
......................................................................ucggugggaucuucguccguuuguUu...........	1	1	c16
......................................................................ucggugggaucuucguccguuuguau...........	1	0	c16
......................................................................ucggugggaucuucguccguuugGau...........	1	1	c16
......................................................................Gcggugggaucuucguccguuuguau...........	1	1	c16
......................................................................ucggugggaucuucguccguuuguauU..........	1	1	c16
.......................................................................cggugggaucuucguccg...................	4	0	c16
.......................................................................cggugggaucuucguccgu..................	3	0	c16
.......................................................................cggugggaucuucguccguu.................	12	0	c16
.......................................................................cggugggaucuucguccguuu................	142	0	c16
.......................................................................Uggugggaucuucguccguuu................	2	1	c16
.......................................................................cggugggCucuucguccguuu................	1	1	c16
.......................................................................cgCugggaucuucguccguuug...............	1	1	c16
.......................................................................cggugggaucuucguccguuug...............	73	0	c16
.......................................................................cggugggaucuucguccguuuU...............	26	1	c16
.......................................................................cggugggaucuucguccguuuC...............	1	1	c16
.......................................................................cggugggaucuucguccguuuA...............	2	1	c16
.......................................................................cggugggaucuucguccguuugA..............	1	1	c16
.......................................................................cggAgggaucuucguccguuugu..............	1	1	c16
.......................................................................cggugggaucuucguccguuugu..............	16	0	c16
.......................................................................cggugggaucuucguccguuuUu..............	6	1	c16
.......................................................................cggugggaucuucguccguuugua.............	5	0	c16
.......................................................................cggugggaucuucguccguuuguau............	6	0	c16
.......................................................................cggugggaucuucguccguuuguaA............	2	1	c16
........................................................................ggugggaucuucguccguuu................	11	0	c16
........................................................................ggugggaucuucgucUguuu................	1	1	c16
........................................................................ggugggaucuucguccguuug...............	1	0	c16
.........................................................................gugggaucuucCuccguuu................	1	1	c16
.........................................................................gugggaucuucguccguuu................	11	0	c16
.........................................................................gugggaucuucguccguuuU...............	3	1	c16
.........................................................................gugggaucuucguccguuuUu..............	1	1	c16
..........................................................................ugggCucuucguccguuu................	1	1	c16
..........................................................................ugggaucuucguccguuu................	7	0	c16
..........................................................................ugggaucuucguccguuuU...............	2	1	c16

...............................cgcccggacgaacuucccagc........................................................	1	0	c04
...............................cgcccggacgaacuucccagcucg.....................................................	1	0	c04
...............................cgcccggacgaacuucccagcucgg....................................................	2	0	c04
................................gcccggacgaacuucccagc........................................................	2	0	c04
................................gcccggacgaacuucccagcucg.....................................................	1	0	c04
................................gcccggacgaacuucccagcucgg....................................................	3	0	c04
................................gcccggacgaacuucccagcucggc...................................................	6	0	c04
................................gcccggacgaacuucccagcucggcA..................................................	1	1	c04
................................gcccggacgaacuucccagcucggcU..................................................	1	1	c04
```

Star Mature

```
cuggaagaaagaagaacgguaucaagugcuacgcccggacgaacuucccagcucggccgacauacacgggucggugggaucuucguccguuuguaucgcuuauacguu
................................gcccggacgaacuucccagcucggcc..................................................   1    0   c04
.................................cccggacgaacuucccag.........................................................   1    0   c04
.................................cccggacgaacuucccagcuG......................................................   1    1   c04
.................................cccggacgaacuucccagcuc......................................................   3    0   c04
.................................cccggacgaacuucccagcucg.....................................................   12   0   c04
.................................cccggacgaacuucccagcucgU....................................................   1    1   c04
.................................cccggacgaacuucccagcuUgg....................................................   1    1   c04
.................................cccggacgaacuucccagcucCg....................................................   1    1   c04
.................................cccggacgaacuucccCgcucgg....................................................   1    1   c04
.................................cccggacgaacuucccagcucgC....................................................   1    1   c04
.................................cccggacgaacuucccagcucgg....................................................   46   0   c04
.................................cccggacgaacuucccagcucggc...................................................   10   0   c04
.................................cccggacgaacuucccagcucggU...................................................   2    1   c04
.................................ccGggacgaacuucccagcucggc...................................................   1    1   c04
.................................cccggacgaacuucccagcucggcU..................................................   5    1   c04
..................................ccggacgaacuucccagc........................................................   2    0   c04
..................................ccggacgaacuucccagcu.......................................................   5    0   c04
..................................ccggacgaacuucccagcuc......................................................   2    0   c04
..................................ccggacgaacuucccagcucg.....................................................   7    0   c04
..................................ccggacgaacuucccagcucgg....................................................   20   0   c04
..................................ccggacgaCcuucccagcucgg....................................................   1    1   c04
..................................ccggacgaacuucccagcucggU...................................................   1    1   c04
..................................ccggacgaacuucccagcucggc...................................................   5    0   c04
..................................ccggacgaacuucccagcucggcU..................................................   1    1   c04
..................................ccggacgaacuucccagcucggcA..................................................   2    1   c04
..................................ccggacgaacuucccagcucggcc..................................................   6    0   c04
..................................ccggacgaacuucccagcucggccU.................................................   6    1   c04
..................................ccggacgaacuucccagcucggccA.................................................   5    1   c04
..................................ccggacgaacuucccagcucggccUa................................................   1    1   c04
..................................ccggacgaacuucccagcucggccgacauacacggg......................................   1    0   c04
...................................cggacgaacuucccagcucggcc..................................................   1    0   c04
.................................................................Ccgggucggugggaucuucguccg...................   1    1   c04
..................................................................cgAgucggugggaucuucguccguuu................   1    1   c04
...................................................................Cggucggugggaucuucguccguuu................   1    1   c04
....................................................................Cgucggugggaucuucguccgu..................   1    1   c04
....................................................................Agucggugggaucuucguccguuu................   1    1   c04
.....................................................................Cucggugggaucuucguccg...................   1    1   c04
.....................................................................Cucggugggaucuucguccguu.................   1    1   c04
.....................................................................gucAgugggaucuucguccguuu................   1    1   c04
.....................................................................Cucggugggaucuucguccguuu................   7    1   c04
......................................................................ucgguggCaucuucgucc....................   1    1   c04
......................................................................ucggCgggaucuucgucc....................   1    1   c04
......................................................................ucggugggaucuucgucc....................   106  0   c04
......................................................................ucggugggaucuucgucU....................   1    1   c04
......................................................................ucggugggaucuucgucA....................   1    1   c04
......................................................................ucggugggaAcuucgucc....................   1    1   c04
......................................................................ucggugggaucuucguUc....................   1    1   c04
......................................................................ucggugggaucuuUgucc....................   1    1   c04
......................................................................Gcggugggaucuucgucc....................   6    1   c04
......................................................................ucggugggaucuucgucAg...................   2    1   c04
......................................................................Ccggugggaucuucguccg...................   1    1   c04
......................................................................ucgguCggaucuucguccg...................   1    1   c04
......................................................................ucggugggCucuucguccg...................   1    1   c04
......................................................................ucggugggaucuucguccU...................   3    1   c04
......................................................................ucgCugggaucuucguccg...................   1    1   c04
......................................................................ucggugggaucuucguccg...................   78   0   c04
......................................................................Gcggugggaucuucguccg...................   12   1   c04
......................................................................ucggugggaucuucguccA...................   1    1   c04
......................................................................ucgCugggaucuucguccgu..................   2    1   c04
......................................................................ucggugAgaucuucguccgu..................   1    1   c04
......................................................................ucCgugggaucuucguccgu..................   1    1   c04
......................................................................ucggugggaucCucguccgu..................   2    1   c04
......................................................................ucggugggaucuucguccgG..................   1    1   c04
......................................................................uAggugggaucuucguccgu..................   1    1   c04
......................................................................ucggugggaucuucguccgA..................   1    1   c04
......................................................................Gcggugggaucuucguccgu..................   16   1   c04
......................................................................ucggugggaucuucguccgu..................   264  0   c04
......................................................................ucgguggUaucuucguccguu.................   1    1   c04
......................................................................Acggugggaucuucguccguu.................   1    1   c04
......................................................................ucggugggCucuucguccguu.................   2    1   c04
```

```
                                 Star                   Mature
cuggaagaaagaagaacgguaucaagugcuacgcccggacgaacuucccagcucggccgacauacacgggucggugggaucuucguccguuuguaucgcuuauacguu
......................................................................ucggugggaucuucguUcguu.................   1      1   c04
......................................................................ucggugggaucuucguccguu.................   479    0   c04
......................................................................ucggugggaucuucguccguC.................   3      1   c04
......................................................................Ccggugggaucuucguccguu.................   1      1   c04
......................................................................ucUgugggaucuucguccguu.................   1      1   c04
......................................................................ucggugggaucuucgucAguu.................   3      1   c04
......................................................................ucggugggaucuuAguccguu.................   1      1   c04
......................................................................Gcggugggaucuucguccguu.................   33     1   c04
......................................................................ucggugggaucuucguccguA.................   1      1   c04
......................................................................ucggugggGucuucguccguu.................   1      1   c04
......................................................................ucggAgggaucuucguccguu.................   1      1   c04
......................................................................ucggugggaucuucguccgGu.................   1      1   c04
......................................................................ucggugggaucuCcguccguuu................   1      1   c04
......................................................................ucggugggaucuucguccCuuu................   1      1   c04
......................................................................ucggugggaucuucguUcguuu................   3      1   c04
......................................................................ucggugggaucuuAguccguuu................   2      1   c04
......................................................................ucggugggUucuucguccguuu................   3      1   c04
......................................................................ucggugggaucuucguccguuC................   4      1   c04
......................................................................ucggugggaucuucguAcguuu................   6      1   c04
......................................................................ucgguUggaucuucguccguuu................   4      1   c04
......................................................................ucggugggaucuucguccgAuu................   2      1   c04
......................................................................ucggugggaucuucCuccguuu................   5      1   c04
......................................................................ucggugggaucuucgAccguuu................   3      1   c04
......................................................................uAggugggaucuucguccguuu................   5      1   c04
......................................................................ucggugggaucuuUguccguuu................   2      1   c04
......................................................................uGggugggaucuucguccguuu................   1      1   c04
......................................................................ucggugggaucGucguccguuu................   3      1   c04
......................................................................ucggugggauAuucguccguuu................   3      1   c04
......................................................................ucgguCggaucuucguccguuu................   5      1   c04
......................................................................ucgAugggaucuucguccguuu................   2      1   c04
......................................................................ucggugggaucuucgucGguuu................   2      1   c04
......................................................................ucggugggaucuuGguccguuu................   1      1   c04
......................................................................ucggugggaucuAcguccguuu................   2      1   c04
......................................................................ucggugggaucuucguccAuuu................   2      1   c04
......................................................................ucggugggaucuucguGcguuu................   3      1   c04
......................................................................ucggAgggaucuucguccguuu................   6      1   c04
......................................................................ucggugggaucuucgCccguuu................   1      1   c04
......................................................................ucggugAgaucuucguccguuu................   2      1   c04
......................................................................ucggugggaucuucguccguGu................   1      1   c04
......................................................................Ccggugggaucuucguccguuu................   2      1   c04
......................................................................ucggugggaucuucguccgCuu................   4      1   c04
......................................................................ucggugggaucuucguccguAu................   3      1   c04
......................................................................ucggugggaucuucguccguCu................   3      1   c04
......................................................................ucggugggaucuucguccgGuu................   2      1   c04
......................................................................ucggugggCucuucguccguuu................   11     1   c04
......................................................................ucgUugggaucuucguccguuu................   3      1   c04
......................................................................ucggugggaucuucguccUuuu................   6      1   c04
......................................................................ucUgugggaucuucguccguuu................   3      1   c04
......................................................................ucggGgggaucuucguccguuu................   1      1   c04
......................................................................Acggugggaucuucguccguuu................   7      1   c04
......................................................................ucggugggaucuucguccguuA................   11     1   c04
......................................................................ucCgugggaucuucguccguuu................   1      1   c04
......................................................................ucAgugggaucuucguccguuu................   1      1   c04
......................................................................Gcggugggaucuucguccguuu................   366    1   c04
......................................................................ucgCugggaucuucguccguuu................   2      1   c04
......................................................................ucgguggUaucuucguccguuu................   2      1   c04
......................................................................uUggugggaucuucguccguuu................   8      1   c04
......................................................................ucggugggaucuucgucUguuu................   5      1   c04
......................................................................ucggugggaucuucguccguuu................   4890   0   c04
......................................................................ucggugCgaucuucguccguuu................   4      1   c04
......................................................................ucggugggGucuucguccguuu................   3      1   c04
......................................................................ucggugggaucuucgucAguuu................   12     1   c04
......................................................................ucggugUgaucuucguccguuu................   4      1   c04
......................................................................ucggugggaucuucAuccguuu................   4      1   c04
......................................................................ucggugggauUuucguccguuu................   1      1   c04
......................................................................ucggugggaucAucguccguuu................   2      1   c04
......................................................................ucggugggaucCucguccguuu................   2      1   c04
......................................................................ucggugggaucuucUuccguuu................   2      1   c04
......................................................................ucggugggaucuucguccguuG................   3      1   c04
......................................................................ucggugggaucuucguccAuuug...............   1      1   c04
```

Star Mature

```
cuggaagaaagaagaacgguaucaagugcuacgcccggacgaacuucccagcucggccgacauacacgggucggugggaucuucguccguuuguaucgcuuauacguu
......................................................................ucggugggaucuucguccguuuC...............	2	1	c04
......................................................................ucggugggaucuucguccguuuA...............	32	1	c04
......................................................................Gcggugggaucuucguccguuug...............	4	1	c04
......................................................................Ccggugggaucuucguccguuug...............	1	1	c04
......................................................................ucggugggaucuucguccguuug...............	32	0	c04
......................................................................ucggugggaucuucguccguuuU...............	283	1	c04
......................................................................ucggugggaucuucguccguuGgu..............	1	1	c04
......................................................................ucggugggaucuucguccguuuUu..............	34	1	c04
......................................................................ucggugggaucuucguccguuugG..............	2	1	c04
......................................................................ucggugggaucuucguccguuuAu..............	2	1	c04
......................................................................ucggugggaucuucguccguuuCu..............	3	1	c04
......................................................................ucggugggaucuucguccguuugu..............	7	0	c04
......................................................................ucggugggaucuucguccguuugC..............	3	1	c04
......................................................................Gcggugggaucuucguccguuugu..............	1	1	c04
......................................................................ucggugggaucuucguccguuugua.............	1	0	c04
......................................................................ucggugggaucuucguccguuugAau............	1	1	c04
.......................................................................cggugggaucuucguccg...................	2	0	c04
.......................................................................cggugggaucuucguccgu..................	1	0	c04
.......................................................................cggugggaucuucguccguA.................	1	1	c04
.......................................................................Gggugggaucuucguccguu.................	1	1	c04
.......................................................................cggugggaucuucguccguu.................	2	0	c04
.......................................................................cggugggaucuucguccguuu................	27	0	c04
.......................................................................cggugggaucuucguccguuug...............	5	0	c04
.......................................................................cggugggaucuucguccguuuU...............	3	1	c04
.......................................................................cggugggaucuucguccguuuA...............	1	1	c04
.......................................................................cggugggaucuucguccguuugA..............	1	1	c04
.......................................................................cggugggaucuucguccguuugu..............	2	0	c04
.......................................................................cggugggaucuucguccguuugua.............	2	0	c04
.......................................................................cggugggaucuucguccguuuguau............	2	0	c04
........................................................................ggugggaucuucguccguu.................	1	0	c04
........................................................................ggugggaucuucguccguuu................	4	0	c04
.........................................................................gugggaucuucguccguu.................	1	0	c04
.........................................................................gugggaucuucguccguuu................	8	0	c04
.........................................................................gugggaucuucguccguuuU...............	1	1	c04
..........................................................................ugggaucuucguccguuu................	5	0	c04
..........................................................................ugggaucuucguccguuuU...............	1	1	c04
```

Provisional ID : group10_15089
Score total : 288715.2
Score for star read(s) : 3.9
Score for read counts : 288704.9
Score for mfe : 1.8
Score for randfold : 1.6
Score for cons. seed : 3
Total read count : 566294
Mature read count : 558955
Loop read count : 0
Star read count : 7339

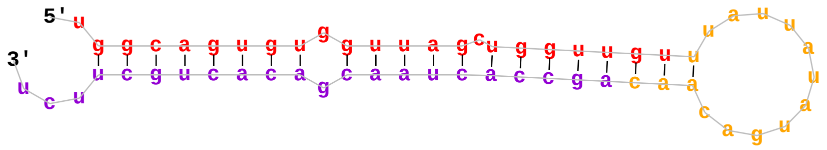

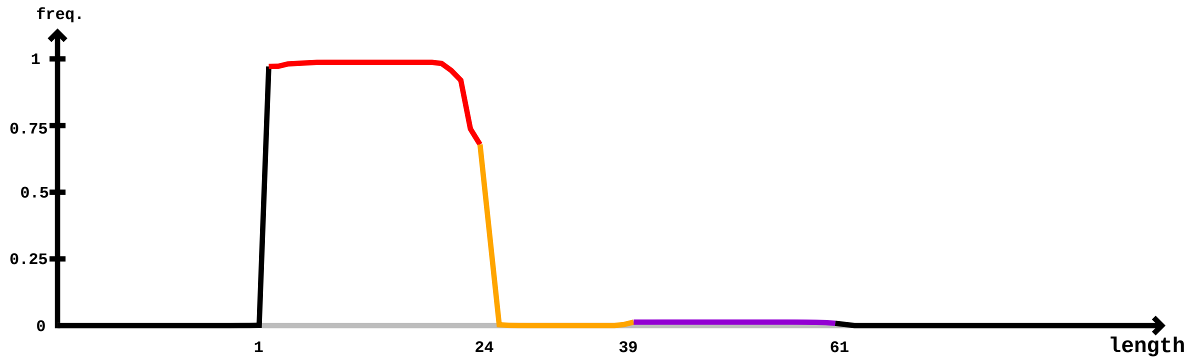

Mature Star

```
5'- gaaaauggguuagaccgcguuggcagugugguuagcugguuguuuauuauaugacaacagccacuaacgacacugcuucugcguacacccugauccuacauaucgacugacag  -3'  obs
    gaaaauggguuagaccgcguuggcagugugguuagcugguuguuuauuauaugacaacagccacuaacgacacugcuucugcguacacccugauccuacauaucgacugacag        exp
    ......(((((((...((((.((((((((.(((((.((((((((...........))))))))))))).))))))))...)))).....))))))).................   reads  mm  sample
    ....auggguuagaccgcguuggcagug.....................................................................................   1      0   c09
    ..............ccgcgCuggcagugugguuagcugguugu......................................................................   1      1   c09
    ...............cUcguuggcagugugguuagcugguu........................................................................   1      1   c09
    ...............cgcCuuggcagugugguuagcugguu........................................................................   1      1   c09
    ...............cCcguuggcagugugguuagcugguugu......................................................................   1      1   c09
    ................gcUuuggcagugugguuagcug...........................................................................   1      1   c09
    ................gcUuuggcagugugguuagcugg..........................................................................   1      1   c09
    ................Ccguuggcagugugguuagcugguu........................................................................   1      1   c09
    ................gcUuuggcagugugguuagcugguugu......................................................................   8      1   c09
    ................Ccguuggcagugugguuagcugguugu......................................................................   1      1   c09
    ................Acguuggcagugugguuagcugguugu......................................................................   1      1   c09
    ................gUguuggcagugugguuagcugguuguu.....................................................................   1      1   c09
    ................gcUuuggcagugugguuagcugguuguu.....................................................................   1      1   c09
    .................cCuuggcagugugguuagcugguu........................................................................   1      1   c09
    .................cgAuggcagugugguuagcugguu........................................................................   1      1   c09
    .................cgGuggcagugugguuagcugguu........................................................................   1      1   c09
    .................cgCuggcagugugguuagcugguu........................................................................   3      1   c09
    .................cUuuggcagugugguuagcugguu........................................................................   2      1   c09
    .................Gguuggcagugugguuagcugguug.......................................................................   1      1   c09
    .................cgCuggcagugugguuagcugguug.......................................................................   1      1   c09
    .................cguuggcagugugguuagcugguuU.......................................................................   1      1   c09
    .................cCuuggcagugugguuagcugguugu......................................................................   4      1   c09
    .................Gguuggcagugugguuagcugguugu......................................................................   3      1   c09
    .................Uguuggcagugugguuagcugguugu......................................................................   1      1   c09
    .................cUuuggcagugugguuagcugguugu......................................................................   5      1   c09
    .................cgCuggcagugugguuagcugguugu......................................................................   25     1   c09
    .................Aguuggcagugugguuagcugguugu......................................................................   6      1   c09
    .................cgGuggcagugugguuagcugguugu......................................................................   3      1   c09
    .................Gguuggcagugugguuagcugguuguu.....................................................................   1      1   c09
    ..................Auuggcagugugguuagcugg..........................................................................   1      1   c09
    ..................gCuggcagugugguuagcuggu.........................................................................   1      1   c09
    ..................Auuggcagugugguuagcugguu........................................................................   1      1   c09
    ..................Uuuggcagugugguuagcugguu........................................................................   3      1   c09
```

Mature Star

gaaaauggguuagaccgcguuggcagugugguuagcugguuguuuauuauaugacaacagccacuaacgacacugcuucugcguacacccugauccuacauaucgacugacag			
..................Cuuggcagugugguuagcugguu..	1	1	c09
..................gCuggcagugugguuagcugguu..	3	1	c09
..................guAggcagugugguuagcugguug...	1	1	c09
..................gAuggcagugugguuagcugguug...	2	1	c09
..................Auuggcagugugguuagcugguugu..	1	1	c09
..................Uuuggcagugugguuagcugguugu..	1	1	c09
..................Cuuggcagugugguuagcugguugu..	1	1	c09
..................gAuggcagugugguuagcugguugu..	3	1	c09
..................gCuggcagugugguuagcugguugu..	13	1	c09
..................guuggcagugugguuagcugguugu..	2	0	c09
..................gGuggcagugugguuagcugguugu..	6	1	c09
..................gGuggcagugugguuagcugguuguu...	1	1	c09
...................Guggcagugugguuagcu..	2	1	c09
...................uuggcagugugguuagcu..	1	0	c09
...................Cuggcagugugguuagcu..	4	1	c09
...................uuggcagugugguuagcug...	1	0	c09
...................uuggcagugugguuagcugg..	2	0	c09
...................Cuggcagugugguuagcugg..	1	1	c09
...................Cuggcagugugguuagcuggu...	5	1	c09
...................uuggcagugugguuagcuggu...	6	0	c09
...................uuggcagugugguuagcugguu..	14	0	c09
...................Guggcagugugguuagcugguu..	1	1	c09
...................Cuggcagugugguuagcugguu..	39	1	c09
...................uuggcagugugguuagcugguug...	5	0	c09
...................uuggcagugugguuagcugguuA...	1	1	c09
...................Cuggcagugugguuagcugguug...	9	1	c09
...................uuggcagugugguuagcugguugu..	37	0	c09
...................Cuggcagugugguuagcugguugu..	98	1	c09
...................Auggcagugugguuagcugguugu..	6	1	c09
...................Guggcagugugguuagcugguugu..	3	1	c09
...................Cuggcagugugguuagcugguuguu...	3	1	c09
...................uuggcagugugguuagcugguuguu...	12	0	c09
...................Guggcagugugguuagcugguuguu...	1	1	c09
...................uuggcagugugguuagcugguuguA...	3	1	c09
...................uuggcagugugguuagcugguuguG...	1	1	c09
...................uuggcagugugguuagcugguuguuu..	1	0	c09
....................uggcagugugguuagcGg...	1	1	c09
....................uggcaAugugguuagcug...	1	1	c09
....................uggcagugugguuagcuC...	52	1	c09
....................uggAagugugguuagcug...	2	1	c09
....................Gggcagugugguuagcug...	131	1	c09
....................uggcagugugguuagUug...	1	1	c09
....................uggcagugugguAagcug...	1	1	c09
....................Cggcagugugguuagcug...	2	1	c09
....................uggcagugugguuaCcug...	2	1	c09
....................uggcagugugguuagcCg...	2	1	c09
....................ugUcagugugguuagcug...	1	1	c09
....................uggcagugugguuagcug...	1571	0	c09
....................uggcagugugguuagcuU...	40	1	c09
....................uggcagugugCuuagcug...	2	1	c09
....................Aggcagugugguuagcug...	4	1	c09
....................uggcagugCgguuagcug...	1	1	c09
....................ugAcagugugguuagcug...	1	1	c09
....................uggcaguguUguuagcug...	1	1	c09
....................uggcagugugguuagcuA...	20	1	c09
....................uggcaguUugguuagcug...	1	1	c09
....................uUgcagugugguuagcug...	3	1	c09
....................uggcaguCugguuagcug...	1	1	c09
....................uggcGgugugguuagcug...	1	1	c09
....................uggcagugugguuagAug...	2	1	c09
....................uggUagugugguuagcug...	1	1	c09
....................uggcagugugguuagcuAg..	3	1	c09
....................ugCcagugugguuagcugg..	1	1	c09
....................uggcaguguCguuagcugg..	4	1	c09
....................uggcagugugguuCgcugg..	1	1	c09
....................uggcagugugguuagUugg..	3	1	c09
....................uggcagugugguAagcugg..	2	1	c09
....................uggcaCugugguuagcugg..	3	1	c09
....................uggcaguAugguuagcugg..	2	1	c09
....................uggcagugugguuagcugC..	4	1	c09

```
                    Mature                                        Star
gaaaauggguuagaccgcguuggcagugugguuagcugguuguuuauuauaugacaacagccacuaacgacacugcuucugcguacacccugauccuacauaucgacugacag
....................uggcUgugugguuagcugg..........................................................................	1	1	c09
....................uggcagugugguuagcGgg..........................................................................	1	1	c09
....................uggcagugugguuUgcugg..........................................................................	2	1	c09
....................uggcagCgugguuagcugg..........................................................................	2	1	c09
....................uggcagugugguuagcugg..........................................................................	6784	0	c09
....................uggAagugugguuagcugg..........................................................................	3	1	c09
....................uUgcagugugguuagcugg..........................................................................	5	1	c09
....................uggcaguguAguuagcugg..........................................................................	1	1	c09
....................ugAcagugugguuagcugg..........................................................................	1	1	c09
....................Gggcagugugguuagcugg..........................................................................	474	1	c09
....................uggcaguguggAuagcugg..........................................................................	3	1	c09
....................uggcagugugguuaAcugg..........................................................................	4	1	c09
....................uggcagugugguuaCcugg..........................................................................	6	1	c09
....................ugUcagugugguuagcugg..........................................................................	1	1	c09
....................uggGagugugguuagcugg..........................................................................	2	1	c09
....................uggcagugugCuuagcugg..........................................................................	8	1	c09
....................uggcagugugguuagcAgg..........................................................................	1	1	c09
....................uggcagugugguuagcuCg..........................................................................	2	1	c09
....................uggcagugugAuuagcugg..........................................................................	1	1	c09
....................uggcagugugguCagcugg..........................................................................	5	1	c09
....................uggcagugugguuaUcugg..........................................................................	6	1	c09
....................uCgcagugugguuagcugg..........................................................................	4	1	c09
....................uggcagugAgguuagcugg..........................................................................	2	1	c09
....................uggcagugugguuagAugg..........................................................................	6	1	c09
....................uggcaguCugguuagcugg..........................................................................	4	1	c09
....................uggcagugugguuagcugU..........................................................................	12	1	c09
....................uggcaAugugguuagcugg..........................................................................	2	1	c09
....................uggcagugugguuagcuUg..........................................................................	2	1	c09
....................uggcagugugguuagcugA..........................................................................	16	1	c09
....................uggcagugugguuagcCgg..........................................................................	1	1	c09
....................uggcagAgugguuagcugg..........................................................................	3	1	c09
....................uggcGgugugguuagcugg..........................................................................	1	1	c09
....................uggcaguUugguuagcugg..........................................................................	1	1	c09
....................uggcagugugUuuagcugg..........................................................................	3	1	c09
....................uAgcagugugguuagcugg..........................................................................	2	1	c09
....................Cggcagugugguuagcugg..........................................................................	7	1	c09
....................uggUagugugguuagcugg..........................................................................	7	1	c09
....................Aggcagugugguuagcugg..........................................................................	22	1	c09
....................uggcaguguUguuagcugg..........................................................................	1	1	c09
....................uggcaguguAguuagcuggu.........................................................................	5	1	c09
....................uCgcagugugguuagcuggu.........................................................................	10	1	c09
....................uggcagugugguuagcuggA.........................................................................	43	1	c09
....................uggcagugugguuGgcuggu.........................................................................	2	1	c09
....................uggAagugugguuagcuggu.........................................................................	5	1	c09
....................uggcUgugugguuagcuggu.........................................................................	1	1	c09
....................uggcaguAugguuagcuggu.........................................................................	2	1	c09
....................uggcagugugguuagcuCgu.........................................................................	2	1	c09
....................uggcagugugguuagcuggu.........................................................................	12708	0	c09
....................uggcagugugguuagcCggu.........................................................................	6	1	c09
....................uggcagugugguuaUcuggu.........................................................................	11	1	c09
....................uggcagugugguuagcuggG.........................................................................	12	1	c09
....................uggcagugugguuUgcuggu.........................................................................	2	1	c09
....................uggcagugugguuagcugAu.........................................................................	7	1	c09
....................uggcagugugguuagcGggu.........................................................................	1	1	c09
....................Cggcagugugguuagcuggu.........................................................................	12	1	c09
....................uggcagugugAuuagcuggu.........................................................................	3	1	c09
....................uUgcagugugguuagcuggu.........................................................................	4	1	c09
....................Gggcagugugguuagcuggu.........................................................................	936	1	c09
....................uggcaguguggCuagcuggu.........................................................................	3	1	c09
....................Aggcagugugguuagcuggu.........................................................................	47	1	c09
....................uggcagugugguuaCcuggu.........................................................................	7	1	c09
....................uggcagCgugguuagcuggu.........................................................................	6	1	c09
....................uggcaAugugguuagcuggu.........................................................................	1	1	c09
....................uggcagugugguuagAuggu.........................................................................	15	1	c09
....................uggcagugugguuagcugCu.........................................................................	11	1	c09
....................uggcagugGgguuagcuggu.........................................................................	3	1	c09
....................uggcagugugguuagcAggu.........................................................................	2	1	c09
....................uggcaguguggGuagcuggu.........................................................................	7	1	c09
....................uggcagugugguuagcuggC.........................................................................	32	1	c09
....................uggcagugugCuuagcuggu.........................................................................	10	1	c09
```

```
                    Mature                                     Star
gaaaauggguuagaccgcguuggcagugugguuagcugguuguuuauuauaugacaacagccacuaacgacacugcuucugcguacacccugauccuacauaucgacugacag
....................uggcaguguggAuagcuggu.........................................................................   7      1   c09
....................uggcagugAgguuagcuggu.........................................................................   7      1   c09
....................uggcagugugguuagcuAgu.........................................................................   2      1   c09
....................uggcaCugugguuagcuggu.........................................................................   4      1   c09
....................uggcagugCgguuagcuggu.........................................................................   4      1   c09
....................ugUcagugugguuagcuggu.........................................................................   2      1   c09
....................uggcaguUugguuagcuggu.........................................................................   3      1   c09
....................uggcagGgugguuagcuggu.........................................................................   2      1   c09
....................uggcaguguCguuagcuggu.........................................................................   7      1   c09
....................uAgcagugugguuagcuggu.........................................................................   9      1   c09
....................ugAcagugugguuagcuggu.........................................................................   6      1   c09
....................uggGagugugguuagcuggu.........................................................................   4      1   c09
....................uggcagAgugguuagcuggu.........................................................................   3      1   c09
....................uggUagugugguuagcuggu.........................................................................   14     1   c09
....................uggcagugugguuagcugUu.........................................................................   19     1   c09
....................uggcagugugguuagUuggu.........................................................................   4      1   c09
....................uggcagugugguuCgcuggu.........................................................................   1      1   c09
....................uggcagugugguCagcuggu.........................................................................   6      1   c09
....................uggcaguCugguuagcuggu.........................................................................   17     1   c09
....................uggcagugugguGagcuggu.........................................................................   1      1   c09
....................uggcagugugguuagGuggu.........................................................................   1      1   c09
....................uggcagugugguuagcuUgu.........................................................................   9      1   c09
....................ugCcagugugguuagcuggu.........................................................................   7      1   c09
....................uggcGgugugguuagcuggu.........................................................................   2      1   c09
....................uggcagugugguAagcuggu.........................................................................   2      1   c09
....................uggcagugugUuuagcugguu........................................................................   8      1   c09
....................uggcaAugugguuagcugguu........................................................................   7      1   c09
....................uggcaguguggGuagcugguu........................................................................   20     1   c09
....................uggcagCgugguuagcugguu........................................................................   14     1   c09
....................uggcagugugguCagcugguu........................................................................   19     1   c09
....................uAgcagugugguuagcugguu........................................................................   20     1   c09
....................uggcagugugguuagcAgguu........................................................................   15     1   c09
....................Aggcagugugguuagcugguu........................................................................   165    1   c09
....................ugAcagugugguuagcugguu........................................................................   18     1   c09
....................uggcaguUugguuagcugguu........................................................................   11     1   c09
....................uggcaCugugguuagcugguu........................................................................   26     1   c09
....................uUgcagugugguuagcugguu........................................................................   24     1   c09
....................uggcagugugguuaCcugguu........................................................................   66     1   c09
....................uggcagugugguuaUcugguu........................................................................   37     1   c09
....................uggcagugugguuagcugguC........................................................................   42     1   c09
....................uggcagugugguuCgcugguu........................................................................   5      1   c09
....................uggcagugugguuagcuggCu........................................................................   16     1   c09
....................uggcagugugguuagcugUuu........................................................................   25     1   c09
....................uggcaguguUguuagcugguu........................................................................   15     1   c09
....................uggcaUugugguuagcugguu........................................................................   21     1   c09
....................uggcaguguggCuagcugguu........................................................................   13     1   c09
....................uggcaguCugguuagcugguu........................................................................   56     1   c09
....................uggcagugugguuagcuUguu........................................................................   10     1   c09
....................uggcCgugugguuagcugguu........................................................................   3      1   c09
....................uggcagugugguuUgcugguu........................................................................   14     1   c09
....................uggAagugugguuagcugguu........................................................................   37     1   c09
....................uggcaguguCguuagcugguu........................................................................   28     1   c09
....................uggcagugugguuagcuAguu........................................................................   12     1   c09
....................uggcagugugguuagcugguu........................................................................   56655  0   c09
....................Gggcagugugguuagcugguu........................................................................   4076   1   c09
....................uggcagGgugguuagcugguu........................................................................   12     1   c09
....................uggcagugugguuaAcugguu........................................................................   3      1   c09
....................uggcagugugguuagcuggAu........................................................................   33     1   c09
....................uggUagugugguuagcugguu........................................................................   48     1   c09
....................uggcagugugCuuagcugguu........................................................................   37     1   c09
....................uggcagugugguuagGugguu........................................................................   4      1   c09
....................uggcagAgugguuagcugguu........................................................................   16     1   c09
....................uggcagugugguuagcCgguu........................................................................   15     1   c09
....................uggcagugugAuuagcugguu........................................................................   7      1   c09
....................uggcagugugguuagcugCuu........................................................................   61     1   c09
....................uggcagugAgguuagcugguu........................................................................   20     1   c09
....................uggcagugCgguuagcugguu........................................................................   12     1   c09
....................uggcagugGgguuagcugguu........................................................................   16     1   c09
....................ugCcagugugguuagcugguu........................................................................   24     1   c09
....................uggcagugugguuagcugguG........................................................................   57     1   c09
```

Mature Star

gaaaauggguuagaccgcguuggcagugugguuagcugguuguuuauuauaugacaacagccacuaacgacacugcuucugcguacacccugauccuacauaucgacugacag			
....................uggcagugugguAagcugguu..	16	1	c09
....................uggcagugugguuagcugguA..	107	1	c09
....................uggcagugugguGagcugguu..	11	1	c09
....................uggcaguguggAuagcugguu..	26	1	c09
....................uggcaguAugguuagcugguu..	14	1	c09
....................uggcUgugugguuagcugguu..	4	1	c09
....................ugUcagugugguuagcugguu..	8	1	c09
....................uggGagugugguuagcugguu..	11	1	c09
....................uggcagugugguuagcugAuu..	18	1	c09
....................Cggcagugugguuagcugguu..	57	1	c09
....................uggcagugugguuagAugguu..	60	1	c09
....................uggcagugugguuGgcugguu..	9	1	c09
....................uggcagugugguuagcuggGu..	15	1	c09
....................uggcGgugugguuagcugguu..	19	1	c09
....................uggcagugugguuagcGgguu..	7	1	c09
....................uCgcagugugguuagcugguu..	24	1	c09
....................uggcaguguAguuagcugguu..	19	1	c09
....................uggcagugugguuagcuCguu..	40	1	c09
....................uggcagugugguuagUugguu..	26	1	c09
....................uggcagugugguCagcugguug...	1	1	c09
....................uggcaguguCguuagcugguug...	8	1	c09
....................uggcagugugguuagcuAguug...	4	1	c09
....................uCgcagugugguuagcugguug...	6	1	c09
....................uggcagugugguuagcugguuU...	1777	1	c09
....................uggcaguguggAuagcugguug...	6	1	c09
....................uggcaguguUguuagcugguug...	4	1	c09
....................uggcagugugguuagcGgguug...	2	1	c09
....................uggcagugCgguuagcugguug...	2	1	c09
....................Cggcagugugguuagcugguug...	12	1	c09
....................ugUcagugugguuagcugguug...	2	1	c09
....................uggcagugugguuagcuggAug...	5	1	c09
....................uggcagugugguuGgcugguug...	2	1	c09
....................uggcagugugguuagcuUguug...	4	1	c09
....................uggcagugugguuagcCgguug...	4	1	c09
....................uggcagugGgguuagcugguug...	3	1	c09
....................uggcaguguAguuagcugguug...	5	1	c09
....................uggcagugugguuaUcugguug...	8	1	c09
....................uggcagAgugguuagcugguug...	2	1	c09
....................uggcagugugguuagcugUuug...	8	1	c09
....................uggcagugugguuagcugguug...	14436	0	c09
....................uggcagugugguuagcuggCug...	4	1	c09
....................uggcagugugguuagcugguuA...	436	1	c09
....................uggcagugugguuagcugguAg...	6	1	c09
....................uggcagugugguuUgcugguug...	3	1	c09
....................uggcagugugguuagcugguuC...	186	1	c09
....................uggcagugAgguuagcugguug...	3	1	c09
....................uUgcagugugguuagcugguug...	4	1	c09
....................uggcaguguggGuagcugguug...	5	1	c09
....................uggcaguCugguuagcugguug...	10	1	c09
....................uggcagugugguAagcugguug...	4	1	c09
....................uggcagugugguuagcugCuug...	15	1	c09
....................uggcUgugugguuagcugguug...	1	1	c09
....................uggcagGgugguuagcugguug...	2	1	c09
....................uggcagCgugguuagcugguug...	2	1	c09
....................uggcagugugguGagcugguug...	2	1	c09
....................uggcagugugguuagcuCguug...	10	1	c09
....................uggcagugugAuuagcugguug...	5	1	c09
....................uggcagugugguuagcugguCg...	5	1	c09
....................uggcagugugguuagcuggGug...	3	1	c09
....................uggcaguUugguuagcugguug...	4	1	c09
....................uggcaUugugguuagcugguug...	4	1	c09
....................Gggcagugugguuagcugguug...	1095	1	c09
....................uAgcagugugguuagcugguug...	3	1	c09
....................uggcaCugugguuagcugguug...	9	1	c09
....................uggcagugugguuagUugguug...	6	1	c09
....................uggGagugugguuagcugguug...	4	1	c09
....................uggcaguAugguuagcugguug...	7	1	c09
....................uggcagugugUuuagcugguug...	1	1	c09
....................Aggcagugugguuagcugguug...	50	1	c09
....................uggcagugugguuagcugAuug...	5	1	c09

Mature Star

```
gaaaaugggguuagaccgcguuggcagugugguuagcugguuguuuauuauaugacaacagccacuaacgacacugcuucugcguacacccugauccuacauaucgacugacag
....................uggcaAugugguuagcugguug.......................................................................  4       1  c09
....................uggcagugugCuuagcugguug.......................................................................  15      1  c09
....................ugAcagugugguuagcugguug.......................................................................  6       1  c09
....................uggcaguguggCuagcugguug.......................................................................  2       1  c09
....................uggcagugugguuagcAgguug.......................................................................  1       1  c09
....................ugCcagugugguuagcugguug.......................................................................  2       1  c09
....................uggcagugugguuagAugguug.......................................................................  15      1  c09
....................uggUagugugguuagcugguug.......................................................................  14      1  c09
....................uggcagugugguuaAcugguug.......................................................................  5       1  c09
....................uggcGgugugguuagcugguug.......................................................................  2       1  c09
....................uggcagugugguuaCcugguug.......................................................................  10      1  c09
....................uggcagugugguuagcugguGg.......................................................................  6       1  c09
....................uggAagugugguuagcugguug.......................................................................  10      1  c09
....................uggcagugugguuagcAgguugu......................................................................  45      1  c09
....................uggcaguCugguuagcugguugu......................................................................  193     1  c09
....................uggcagugugguuaUcugguugu......................................................................  87      1  c09
....................uggcagugugguuagcugguugA......................................................................  290     1  c09
....................ugUcagugugguuagcugguugu......................................................................  62      1  c09
....................uggcaguguggGuagcugguugu......................................................................  86      1  c09
....................uggcagugugguuagcuUguugu......................................................................  48      1  c09
....................uggcaAugugguuagcugguugu......................................................................  27      1  c09
....................uggcaguguggCuagcugguugu......................................................................  34      1  c09
....................uAgcagugugguuagcugguugu......................................................................  66      1  c09
....................Gggcagugugguuagcugguugu......................................................................  13048   1  c09
....................uggcagugugguuagcugguugG......................................................................  130     1  c09
....................uggcagugugguuagcGgguugu......................................................................  20      1  c09
....................Aggcagugugguuagcugguugu......................................................................  539     1  c09
....................uggcagugugguAagcugguugu......................................................................  25      1  c09
....................uggcagugugguuUgcugguugu......................................................................  16      1  c09
....................uggcagugCgguuagcugguugu......................................................................  32      1  c09
....................uggcagugugguuagcugguGgu......................................................................  23      1  c09
....................uggcagugugguuagcugguugu......................................................................  174710  0  c09
....................uggcaUugugguuagcugguugu......................................................................  52      1  c09
....................uggcagugugguuagUugguugu......................................................................  102     1  c09
....................uggcaguguggAuagcugguugu......................................................................  73      1  c09
....................uggcagugugguuagAugguugu......................................................................  159     1  c09
....................uggcagugugguuaCcugguugu......................................................................  131     1  c09
....................uggcagAgugguuagcugguugu......................................................................  64      1  c09
....................uggcaguAugguuagcugguugu......................................................................  75      1  c09
....................uggcagugugguuaAcugguugu......................................................................  30      1  c09
....................uggcagugugguuagcugguAgu......................................................................  55      1  c09
....................uggcagugugguGagcugguugu......................................................................  31      1  c09
....................uggcGgugugguuagcugguugu......................................................................  46      1  c09
....................uggcagugugguuagcuggAugu......................................................................  56      1  c09
....................uggcagugugguuagcuAguugu......................................................................  27      1  c09
....................uggcaguguAguuagcugguugu......................................................................  36      1  c09
....................ugCcagugugguuagcugguugu......................................................................  89      1  c09
....................uggcagugugguuagcCgguugu......................................................................  67      1  c09
....................uggcagugugguuagcugCuugu......................................................................  231     1  c09
....................uggcagugugguuCgcugguugu......................................................................  14      1  c09
....................ugAcagugugguuagcugguugu......................................................................  59      1  c09
....................uggcagugugguuGgcugguugu......................................................................  22      1  c09
....................uggcaCugugguuagcugguugu......................................................................  112     1  c09
....................uggcaguguCguuagcugguugu......................................................................  102     1  c09
....................uggcagugugCuuagcugguugu......................................................................  133     1  c09
....................uggcaguguUguuagcugguugu......................................................................  36      1  c09
....................uggcagugugguCagcugguugu......................................................................  35      1  c09
....................uggcagCgugguuagcugguugu......................................................................  33      1  c09
....................uggcagugugguuagcuggCugu......................................................................  53      1  c09
....................uggcagugugguuagGugguugu......................................................................  21      1  c09
....................uggcagugugguuagcugUuugu......................................................................  64      1  c09
....................uggcagugugAuuagcugguugu......................................................................  34      1  c09
....................uggcagugugUuuagcugguugu......................................................................  35      1  c09
....................uggcagugugguuagcugguCgu......................................................................  54      1  c09
....................uCgcagugugguuagcugguugu......................................................................  99      1  c09
....................uggcagugugguuagcugguuUu......................................................................  229     1  c09
....................uggcagugugguuagcuCguugu......................................................................  123     1  c09
....................uggcagGgugguuagcugguugu......................................................................  27      1  c09
....................uggAagugugguuagcugguugu......................................................................  103     1  c09
....................uggGagugugguuagcugguugu......................................................................  39      1  c09
```

Mature Star

gaaaauggguuagaccgcguuggcagugugguuagcugguuguuuauuauaugacaacagccacuaacgacacugcuucugcguacacccugauccuacauaucgacugacag			
....................uggcaguUugguuagcugguugu..	48	1	c09
....................uggcagugGgguuagcugguugu..	41	1	c09
....................uggcagugugguuagcuggGugu..	27	1	c09
....................uggcagugugguuagcugguugC..	85	1	c09
....................Cggcagugugguuagcugguugu..	164	1	c09
....................uggcagugAgguuagcugguugu..	64	1	c09
....................uggcUgugugguuagcugguugu..	25	1	c09
....................uggcCgugugguuagcugguugu..	4	1	c09
....................uUgcagugugguuagcugguugu..	65	1	c09
....................uggUagugugguuagcugguugu..	150	1	c09
....................uggcagugugguuagcugAuugu..	37	1	c09
....................uggcagugugguuagcugguuAu..	55	1	c09
....................uggcagugugguuagcugguuCu..	194	1	c09
....................uggcagugugguuagcugguAguu...	3	1	c09
....................ugCcagugugguuagcugguuguu...	3	1	c09
....................uggcaguguCguuagcugguuguu...	1	1	c09
....................Aggcagugugguuagcugguuguu...	17	1	c09
....................uggcagugugguuagcugguuAuu...	2	1	c09
....................uggcagugugguuagcugguuguu...	6025	0	c09
....................uggcagugugguuagcuAguuguu...	1	1	c09
....................uggcagGgugguuagcugguuguu...	1	1	c09
....................ugAcagugugguuagcugguuguu...	3	1	c09
....................uggcagugugguuagcugAuuguu...	2	1	c09
....................uggcagugAgguuagcugguuguu...	1	1	c09
....................uggcagugugguuagAugguuguu...	4	1	c09
....................uggcagugugguuagcuggAuguu...	1	1	c09
....................uggcUgugugguuagcugguuguu...	2	1	c09
....................uggcagugugguuagcuCguuguu...	6	1	c09
....................uggcagugugguuagcuggCuguu...	2	1	c09
....................Gggcagugugguuagcugguuguu...	464	1	c09
....................uggcaguguggGuagcugguuguu...	4	1	c09
....................uggcagugugguuaCcugguuguu...	4	1	c09
....................uAgcagugugguuagcugguuguu...	2	1	c09
....................uggcagugugguuagcugguugGu...	11	1	c09
....................uggcagugugguuagcugguugCu...	8	1	c09
....................uggcagugugguuagcugUuuguu...	1	1	c09
....................uggcaguguggCuagcugguuguu...	1	1	c09
....................uggcagugugCuuagcugguuguu...	5	1	c09
....................uggcagugugguuagcugCuuguu...	12	1	c09
....................uggcagugugguuagcugguCguu...	3	1	c09
....................uggcagugugguuagcugguuUuu...	17	1	c09
....................uggcGgugugguuagcugguuguu...	2	1	c09
....................uggcaguUugguuagcugguuguu...	1	1	c09
....................uggUagugugguuagcugguuguu...	5	1	c09
....................Cggcagugugguuagcugguuguu...	7	1	c09
....................uggAagugugguuagcugguuguu...	3	1	c09
....................uggcagugugguCagcugguuguu...	1	1	c09
....................uggcagugugguuagcuUguuguu...	1	1	c09
....................uggcagugugguuagcugguuguA...	1948	1	c09
....................uggcaCugugguuagcugguuguu...	5	1	c09
....................uggcagugugguuagcGgguuguu...	2	1	c09
....................uggcagugugguuaUcugguuguu...	3	1	c09
....................uggcagugGgguuagcugguuguu...	1	1	c09
....................uggcagugugguuagcugguGguu...	4	1	c09
....................ugUcagugugguuagcugguuguu...	1	1	c09
....................uggGagugugguuagcugguuguu...	2	1	c09
....................uggcaAugugguuagcugguuguu...	2	1	c09
....................uggcagugugguuagcugguuguC...	479	1	c09
....................uggcagugugguuagcugguugAu...	4	1	c09
....................uUgcagugugguuagcugguuguu...	1	1	c09
....................uCgcagugugguuagcugguuguu...	2	1	c09
....................uggcagugugguuagcAgguuguu...	1	1	c09
....................uggcagugugguuagcuggGuguu...	1	1	c09
....................uggcagugugguuagcugguuCuu...	14	1	c09
....................uggcagugCgguuagcugguuguu...	1	1	c09
....................uggcaguguggAuagcugguuguu...	2	1	c09
....................uggcaguCugguuagcugguuguu...	2	1	c09
....................uggcagugugguGagcugguuguu...	1	1	c09
....................uggcagugugguuagcugguuguG...	477	1	c09
....................uggcaguguUguuagcugguuguu...	1	1	c09

Mature **Star**

```
gaaaauggguuagaccgcguuggcagugugguuagcugguuguuuauuauaugacaacagccacuaacgacacugcuucugcguacacccugauccuacauaucgacugacag
....................uggcaguAugguuagcugguuguu.....................................................................   1     1   c09
....................uggcagugugUuuagcugguuguu.....................................................................   2     1   c09
....................uggcagugugguuGgcugguuguu.....................................................................   1     1   c09
....................uggcagugugguuagUugguuguu.....................................................................   4     1   c09
....................uggcagugugguuagcugguuguuG....................................................................   21    1   c09
....................uggcagugugguuagcugguuguuA....................................................................   145   1   c09
....................Gggcagugugguuagcugguuguuu....................................................................   26    1   c09
....................uggcagugugUuuagcugguuguuu....................................................................   1     1   c09
....................Aggcagugugguuagcugguuguuu....................................................................   3     1   c09
....................uggcagugugguuagcugguuguCu....................................................................   24    1   c09
....................uggcagugugguuagcugguuAuuu....................................................................   1     1   c09
....................uggcagugugguuagcugguuguuu....................................................................   296   0   c09
....................uggcagugugguuagcugguuguAu....................................................................   70    1   c09
....................uggUagugugguuagcugguuguuu....................................................................   1     1   c09
....................uggcagugugguuagcugguuUuuu....................................................................   1     1   c09
....................uggcagugugguuagcugguuguGu....................................................................   34    1   c09
....................uggcagugugguuagcugguugGuu....................................................................   8     1   c09
....................uggcagugugguuagcugguugAuu....................................................................   2     1   c09
....................uggcagugugguuagcugguuCuuu....................................................................   1     1   c09
....................uggcagugugguuagcugguugCuu....................................................................   3     1   c09
....................uggcagugugguuagcugguuguuC....................................................................   37    1   c09
....................uggcagugugguuagcugguuguuuG...................................................................   4     1   c09
....................uggcagugugguuagcugguuguuGa...................................................................   3     1   c09
....................uggcagugugguuagcugguuguuua...................................................................   27    0   c09
....................Gggcagugugguuagcugguuguuua...................................................................   4     1   c09
....................uggcagugugguuagcugguuguuuU...................................................................   42    1   c09
....................uggcagugugguuagcugguuguuuC...................................................................   9     1   c09
....................uggcagugugguuagcugguuguuAa...................................................................   15    1   c09
....................uggcagugugguuagcugguuguuCa...................................................................   2     1   c09
....................uggcagugugguuagcugguugCuua...................................................................   1     1   c09
....................uggcagugugguuagcugguuguAua...................................................................   4     1   c09
....................uggcagugugguuagcugguuguCua...................................................................   2     1   c09
....................uggcagugugguuagcugguuguGua...................................................................   3     1   c09
....................uggcagugugguuagcugguuguuAau..................................................................   4     1   c09
....................uggcagugugguuagcugguuguuuUu..................................................................   1     1   c09
....................uggcagugugguuagcugguuguuuaA..................................................................   3     1   c09
....................uggcagugugguuagcugguuguuuGu..................................................................   3     1   c09
....................uggcagugugguuagcugguuguuGau..................................................................   2     1   c09
....................uggcagugugguuagcugguuguuuau..................................................................   5     0   c09
....................uggcagugugguuagcugguuguuuCu..................................................................   1     1   c09
....................uggcagugugguuagcugguuguuuauA.................................................................   1     1   c09
....................uggcagugugguuagcugguuguuAauu.................................................................   1     1   c09
....................uggcagugugguuagcugguuguuuauu.................................................................   2     0   c09
....................uggcagugugguuagcugguuguuuUuua................................................................   1     1   c09
....................Gggcagugugguuagcugguuguuuauua................................................................   1     1   c09
....................uggcagugugguuagcugguuguuuauua................................................................   2     0   c09
....................uggcagugugguuagcugguuguuuauuaua..............................................................   4     0   c09
....................uggcagugugguuagcugguuguuuauuauau.............................................................   1     0   c09
....................uggcagugugguuagcugguuguuuauuauaugaca.........................................................   3     0   c09
....................uggcagugugguuagcugguuguuuauuauaugacaac.......................................................   5     0   c09
....................uggcagugugguuagcugguuguuuauuauaugacaacU......................................................   1     1   c09
.....................Ugcagugugguuagcugg..........................................................................   1     1   c09
.....................ggcagugugguuagcugg..........................................................................   4     0   c09
.....................ggUagugugguuagcugg..........................................................................   1     1   c09
.....................ggcagugugguuagcuggu.........................................................................   2     0   c09
.....................ggGagugugguuagcugguu........................................................................   1     1   c09
.....................ggcagugugguuagcugguu........................................................................   10    0   c09
.....................ggcaguguAguuagcugguu........................................................................   1     1   c09
.....................Ugcagugugguuagcugguu........................................................................   5     1   c09
.....................gUcagugugguuagcugguug.......................................................................   1     1   c09
.....................ggcagugugguuagcugguug.......................................................................   9     0   c09
.....................Ugcagugugguuagcugguug.......................................................................   1     1   c09
.....................ggcagugugguuagcugguuU.......................................................................   1     1   c09
.....................ggcagugugguuagcugguuC.......................................................................   1     1   c09
.....................gAcagugugguuagcugguugu......................................................................   4     1   c09
.....................ggUagugugguuagcugguugu......................................................................   1     1   c09
.....................ggcaguCugguuagcugguugu......................................................................   1     1   c09
.....................Ugcagugugguuagcugguugu......................................................................   21    1   c09
.....................ggcUgugugguuagcugguugu......................................................................   1     1   c09
.....................Agcagugugguuagcugguugu......................................................................   1     1   c09
```

```
                             Mature                                    Star
gaaaauggguuagaccgcguuggcagugugguuagcugguuguuuauuauaugacaacagccacuaacgacacugcuucugcguacacccugauccuacauaucgacugacag
.....................Cgcagugugguuagcugguugu......................................................................   2      1   c09
.....................ggcagugugguuagcugguCgu......................................................................   1      1   c09
.....................ggcagugugguuagcugguuUu......................................................................   1      1   c09
.....................ggcagugugguuagcugguugu......................................................................   80     0   c09
.....................Cgcagugugguuagcugguuguu.....................................................................   1      1   c09
.....................ggcagugugguuagcugguuAuu.....................................................................   1      1   c09
.....................ggcagugugguuagcugguuguu.....................................................................   86     0   c09
.....................ggcaguguggCuagcugguuguu.....................................................................   1      1   c09
.....................Ugcagugugguuagcugguuguu.....................................................................   2      1   c09
.....................ggcagugugguuagcugguuguA.....................................................................   2      1   c09
.....................ggcagugugguuagcugguuguuC....................................................................   1      1   c09
.....................ggcagugugguuagcugguuguuu....................................................................   1      0   c09
.....................ggcagugGgguuagcugguuguuu....................................................................   1      1   c09
.....................ggcagugugguuagcugguuguuAa...................................................................   2      1   c09
.....................ggcagugugguuagcugguuguuAau..................................................................   1      1   c09
......................gcagugugguuagcugCu.........................................................................   1      1   c09
......................gcagugugguuagcuggu.........................................................................   36     0   c09
......................gcaguguggAuagcuggu.........................................................................   1      1   c09
......................gcagugugguuagcugguu........................................................................   63     0   c09
......................Ucagugugguuagcugguug.......................................................................   1      1   c09
......................Ccagugugguuagcugguug.......................................................................   1      1   c09
......................gcagugugguuagcugguug.......................................................................   55     0   c09
......................Ucagugugguuagcugguugu......................................................................   4      1   c09
......................gcagugugguuagAugguugu......................................................................   1      1   c09
......................gcagugugguuagcugguugu......................................................................   256    0   c09
......................gcaguguCguuagcugguugu......................................................................   2      1   c09
......................gcagugugCuuagcugguugu......................................................................   1      1   c09
......................gcaguCugguuagcugguugu......................................................................   1      1   c09
......................gcagugugguuagcugguugC......................................................................   1      1   c09
......................gGagugugguuagcugguugu......................................................................   1      1   c09
......................gcUgugugguuagcugguugu......................................................................   1      1   c09
......................gcCgugugguuagcugguugu......................................................................   2      1   c09
......................gcagugugguuagcugguugA......................................................................   1      1   c09
......................gcagugugguuagcuCguugu......................................................................   1      1   c09
......................gcagugugguCagcugguugu......................................................................   1      1   c09
......................gUagugugguuagcugguugu......................................................................   1      1   c09
......................gAagugugguuagcugguugu......................................................................   4      1   c09
......................gcagugugguuagcugUuugu......................................................................   1      1   c09
......................Acagugugguuagcugguuguu.....................................................................   5      1   c09
......................gUagugugguuagcugguuguu.....................................................................   3      1   c09
......................gcagugugCuuagcugguuguu.....................................................................   2      1   c09
......................gcagGgugguuagcugguuguu.....................................................................   1      1   c09
......................gcaCugugguuagcugguuguu.....................................................................   1      1   c09
......................gcaUugugguuagcugguuguu.....................................................................   2      1   c09
......................gcagugugguuagcugCuuguu.....................................................................   1      1   c09
......................gcagugugguuagcugguuCuu.....................................................................   2      1   c09
......................Ucagugugguuagcugguuguu.....................................................................   11     1   c09
......................gcagugugguuagcugguugAu.....................................................................   2      1   c09
......................gcagugugguuagcuCguuguu.....................................................................   4      1   c09
......................gcagugugguuCgcugguuguu.....................................................................   1      1   c09
......................gcagAgugguuagcugguuguu.....................................................................   2      1   c09
......................gcagugugguuagcugguuguG.....................................................................   1      1   c09
......................gcagugugguuagcugUuuguu.....................................................................   1      1   c09
......................Ccagugugguuagcugguuguu.....................................................................   9      1   c09
......................gcagugugguuagcCgguuguu.....................................................................   1      1   c09
......................gcagugugguuagcugguuguu.....................................................................   1521   0   c09
......................gcagugugguuagcuggAuguu.....................................................................   1      1   c09
......................gcagugugguuagcugguuguA.....................................................................   4      1   c09
......................gcaguguggGuagcugguuguu.....................................................................   1      1   c09
......................gcagugugguuagAugguuguu.....................................................................   3      1   c09
......................gcagugugAuuagcugguuguu.....................................................................   2      1   c09
......................gcagugugguuagcugguuguC.....................................................................   2      1   c09
......................gcagugugguuaAcugguuguu.....................................................................   1      1   c09
......................gcagugugguuagcugguuguuA....................................................................   10     1   c09
......................gcagugugguuagcugguuguuC....................................................................   2      1   c09
......................gcagugugguuagcugguuguuG....................................................................   6      1   c09
......................gcagugugguuagcugguuguuu....................................................................   57     0   c09
......................gcagugugguuagcugguuguuuU...................................................................   6      1   c09
......................gcagugugguuagcugguuguuuC...................................................................   1      1   c09
......................gcagugugguuagcugguuguuGa...................................................................   1      1   c09
```

Mature   Star

```
gaaaauggguuagaccgcguuggcagugugguuagcugguuguuuauuauaugacaacagccacuaacgacacugcuucugcguacacccugauccuacauaucgacugacag
......................gcagugugguuagcugguuguuAa...................................................................    6     1    c09
......................gcagugugguuagcugguuguuuau..................................................................    1     0    c09
......................gcagugugguuagcugguuguuuUu..................................................................    1     1    c09
......................gcagugugguuagcugguuguuuauua................................................................    2     0    c09
.......................Gagugugguuagcugguu........................................................................    1     1    c09
.......................cagugugguuagcugguu........................................................................    82    0    c09
.......................cagugugguuagcugAuu........................................................................    1     1    c09
.......................cagugugguuagcugguA........................................................................    1     1    c09
.......................cagugugguuagcugguuU.......................................................................    3     1    c09
.......................cagugugguuagcugguuA.......................................................................    1     1    c09
.......................cagugugguuagcugguug.......................................................................    19    0    c09
.......................caguUugguuagcugguug.......................................................................    1     1    c09
.......................cagCgugguuagcugguugu......................................................................    1     1    c09
.......................caguguggGuagcugguugu......................................................................    1     1    c09
.......................caguUugguuagcugguugu......................................................................    1     1    c09
.......................cagugGgguuagcugguugu......................................................................    1     1    c09
.......................caguAugguuagcugguugu......................................................................    1     1    c09
.......................cagugugguuagAugguugu......................................................................    2     1    c09
.......................cagugugguuagUugguugu......................................................................    2     1    c09
.......................cagugugguuagcugguugu......................................................................    404   0    c09
.......................Aagugugguuagcugguugu......................................................................    1     1    c09
.......................caguguggAuagcugguugu......................................................................    1     1    c09
.......................cagugugUuuagcugguugu......................................................................    2     1    c09
.......................caUugugguuagcugguugu......................................................................    4     1    c09
.......................cUgugugguuagcugguugu......................................................................    2     1    c09
.......................cagugugguuaUcugguugu......................................................................    1     1    c09
.......................cagugugguuagcugguuCu......................................................................    1     1    c09
.......................caguguggGuagcugguuguu.....................................................................    1     1    c09
.......................Aagugugguuagcugguuguu.....................................................................    1     1    c09
.......................cagugugguuagcugguuguu.....................................................................    15    0    c09
.......................cagugugguuagcugguuguA.....................................................................    4     1    c09
.......................cagugugguuagcugguuguuu....................................................................    1     0    c09
.......................cagugugguuagcugguuguuA....................................................................    2     1    c09
........................agugugguuagcugguuA.......................................................................    1     1    c09
........................aCugugguuagcugguug.......................................................................    1     1    c09
........................Cgugugguuagcugguug.......................................................................    1     1    c09
........................agugugguuagcugguuC.......................................................................    1     1    c09
........................aUugugguuagcugguug.......................................................................    1     1    c09
........................agugugguuagcugguuU.......................................................................    6     1    c09
........................agugugguuagcugguug.......................................................................    47    0    c09
........................Cgugugguuagcugguugu......................................................................    15    1    c09
........................agugugguuagcugguugG......................................................................    2     1    c09
........................agugugguuagUugguugu......................................................................    1     1    c09
........................agGgugguuagcugguugu......................................................................    2     1    c09
........................agugugguuagcugguugu......................................................................    596   0    c09
........................Ggugugguuagcugguugu......................................................................    4     1    c09
........................agugugAuuagcugguugu......................................................................    1     1    c09
........................aguAugguuagcugguugu......................................................................    1     1    c09
........................agugugguuagcugguCgu......................................................................    1     1    c09
........................agugugguuagcuggAugu......................................................................    1     1    c09
........................agugugguuagcugguugC......................................................................    1     1    c09
........................aguguCguuagcugguugu......................................................................    1     1    c09
........................aUugugguuagcugguugu......................................................................    11    1    c09
........................agugugguuagcugguugA......................................................................    1     1    c09
........................agugugguuagcuggCugu......................................................................    1     1    c09
........................agugugguuagAugguugu......................................................................    2     1    c09
........................agugugguuagcugUuugu......................................................................    1     1    c09
........................agugAgguuagcugguugu......................................................................    1     1    c09
........................agugugCuuagcugguugu......................................................................    1     1    c09
........................agugugguuaCcugguugu......................................................................    1     1    c09
........................aCugugguuagcugguugu......................................................................    3     1    c09
........................aguguUguuagcugguugu......................................................................    1     1    c09
........................agugugguuagcugguuguG.....................................................................    2     1    c09
........................agugugguuagcugguuguA.....................................................................    7     1    c09
........................agugugguuGgcugguuguu.....................................................................    1     1    c09
........................aUugugguuagcugguuguu.....................................................................    1     1    c09
........................agugugguuagcugguuguC.....................................................................    3     1    c09
........................agugugguuagcugguuguu.....................................................................    35    0    c09
........................agugugguuagcugguuguuu....................................................................    1     0    c09
........................agugugguuagcugguuguuA....................................................................    2     1    c09
```

```
                    Mature                          Star
gaaaaugggguuagaccgcguuggcagugugguuagcugguuguuuauuauaugacaacagccacuaacgacacugcuucugcguacacccugauccuacauaucgacugacag
........................agugugguuagcugguuguuua...................................................................   2     0    c09
........................agugugguuagcugguuguuuU...................................................................   1     1    c09
........................agugugguuagcugguuguuuau..................................................................   1     0    c09
........................agugugguuagcugguuguuuauu.................................................................   1     0    c09
........................agugugguuagcugguuguuuauuauaugaca.........................................................   1     0    c09
.........................gugugguuagcugguuUu......................................................................   1     1    c09
.........................Uugugguuagcugguugu......................................................................   2     1    c09
.........................guUugguuagcugguugu......................................................................   1     1    c09
.........................gugugguuagcugguugu......................................................................   562   0    c09
.........................gugGgguuagcugguugu......................................................................   2     1    c09
.........................gugugguuagcugUuugu......................................................................   1     1    c09
.........................gGgugguuagcugguugu......................................................................   3     1    c09
.........................gugugguuGgcugguugu......................................................................   1     1    c09
.........................gugugguuagcugCuugu......................................................................   1     1    c09
.........................Augugguuagcugguugu......................................................................   1     1    c09
.........................gAgugguuagcugguugu......................................................................   3     1    c09
.........................Cugugguuagcugguugu......................................................................   2     1    c09
.........................guguCguuagcugguugu......................................................................   1     1    c09
.........................guguAguuagcugguugu......................................................................   1     1    c09
.........................guCugguuagcugguugu......................................................................   3     1    c09
.........................gugugguuagcugguGgu......................................................................   1     1    c09
.........................gAgugguuagcugguuguu.....................................................................   1     1    c09
.........................gugugguuagcugguuguG.....................................................................   1     1    c09
.........................gugugguuagcugguuguA.....................................................................   5     1    c09
.........................gugugguuagcugguuguu.....................................................................   35    0    c09
.........................gugugguuagcugguugGuu....................................................................   2     1    c09
.........................gugugguuagcugguuguAu....................................................................   1     1    c09
.........................gugugguuagcugguuguuA....................................................................   1     1    c09
.........................gugugguuagcugguuguuG....................................................................   1     1    c09
.........................gugugguuagcugguuguuC....................................................................   1     1    c09
.........................gugugguuagcugguuguuu....................................................................   4     0    c09
.........................gugugguuagcugguuguuuau..................................................................   1     0    c09
..........................ugugguuagcugguuguu.....................................................................   12    0    c09
..........................ugugguuagcugguuguC.....................................................................   1     1    c09
..........................ugugguuagcugguuguA.....................................................................   1     1    c09
..........................ugugguuagcugguugCu.....................................................................   1     1    c09
...........................gugguuagcugguCguuu....................................................................   1     1    c09
...........................gugguuagcugguuguuu....................................................................   1     0    c09
...........................gugguuagcugguuguuG....................................................................   1     1    c09
...........................gugguuagcugguuguuC....................................................................   2     1    c09
...........................gugguuagcugguuguuA....................................................................   1     1    c09
...........................gugguuagcugguuguGu....................................................................   1     1    c09
..............................................uuauaugacaacagccacuaacgacacu.......................................   1     0    c09
...............................................uauaugacaacagccacuaacgacacugc.....................................   1     0    c09
...............................................uauaugacaacagccacuaacgacacugcuu...................................   2     0    c09
...............................................uauaugacaacagccacuaacgacacugcuucu.................................   1     0    c09
................................................auaugacaacagccacuaacgacacugcuucu.................................   1     0    c09
.................................................uaugacaacagccacuaacgac..........................................   1     0    c09
..................................................augacaacCgccacuaacgacacugcuucu.................................   1     1    c09
...................................................Ggacaacagccacuaacgacacugcu....................................   1     1    c09
...................................................ugacaacagccacuaacgacacugcuucugc...............................   1     0    c09
......................................................caacagccacuaacgacacugcu....................................   1     0    c09
.......................................................aacagccacuaacgacacugcuuc..................................   1     0    c09
.......................................................aacagccacuaacgacacugcuucu.................................   3     0    c09
........................................................acagccacuaacgacacugc.....................................   1     0    c09
........................................................acagccacuaacgacacugcu....................................   1     0    c09
........................................................acagccacuaacgacacugcuu...................................   9     0    c09
........................................................Ccagccacuaacgacacugcuu...................................   2     1    c09
........................................................acagccacuaacgacacugcuuc..................................   2     0    c09
........................................................acagccacuaacgacacugcuucu.................................   12    0    c09
........................................................acagccCcuaacgacacugcuucu.................................   1     1    c09
........................................................Ccagccacuaacgacacugcuucu.................................   3     1    c09
........................................................acagccacuaaAgacacugcuucu.................................   1     1    c09
.........................................................cagccacuaacgacacug......................................   15    0    c09
.........................................................cagccacuaacgacacuU......................................   1     1    c09
.........................................................cagccacuaacgacacugc.....................................   50    0    c09
.........................................................cagccacuaacgacacuCc.....................................   2     1    c09
.........................................................caCccacuaacgacacugcu....................................   1     1    c09
.........................................................cagccacuaacgCcacugcu....................................   1     1    c09
.........................................................cagccacuaacgacacugcu....................................   64    0    c09
```

```
                     Mature                                      Star
gaaaaugguuagaccgcguuggcagugugguuagcugguuguuuauuauaugacaacagccacuaacgacacugcuucugcguacacccugauccuacauaucgacugacag
........................................................cagccacuaCcgacacugcuu...................................  1    1  c09
........................................................cagccacuaacgacacugcCu...................................  2    1  c09
........................................................cagccacuaacgacacugcuC...................................  2    1  c09
........................................................cagAcacuaacgacacugcuu...................................  1    1  c09
........................................................cagccacuCacgacacugcuu...................................  1    1  c09
........................................................cCgccacuaacgacacugcuu...................................  1    1  c09
........................................................cagccacuaacgacacugcuu...................................  124  0  c09
........................................................cCgccacuaacgacacugcuuc..................................  7    1  c09
........................................................cagcGacuaacgacacugcuuc..................................  1    1  c09
........................................................cagccacuaacgacacugcuuc..................................  533  0  c09
........................................................cagccacuaacgaUacugcuuc..................................  1    1  c09
........................................................cGgccacuaacgacacugcuuc..................................  1    1  c09
........................................................cagccacuaGcgacacugcuuc..................................  1    1  c09
........................................................cagccacuaacgacacugcuuU..................................  24   1  c09
........................................................cagccacuaacgacacugcuuA..................................  2    1  c09
........................................................cagUcacuaacgacacugcuuc..................................  1    1  c09
........................................................cagccacuaCcgacacugcuuc..................................  4    1  c09
........................................................cagcAacuaacgacacugcuuc..................................  1    1  c09
........................................................Gagccacuaacgacacugcuuc..................................  1    1  c09
........................................................cagccacuaacgacacuAcuuc..................................  1    1  c09
........................................................cagccacuaacgCcacugcuuc..................................  3    1  c09
........................................................cagccacuaacgacacugcGuc..................................  1    1  c09
........................................................cagccacuaacgacacCgcuuc..................................  1    1  c09
........................................................Uagccacuaacgacacugcuuc..................................  1    1  c09
........................................................cagAcacuaacgacacugcuuc..................................  1    1  c09
........................................................cagAcacuaacgacacugcuucu.................................  1    1  c09
........................................................cagccacuaacgacacugcuucu.................................  153  0  c09
........................................................cagccacuaacUacacugcuucu.................................  1    1  c09
........................................................cagccacuaacgacacugcuucG.................................  1    1  c09
........................................................cagccacuaacgacacugcuucA.................................  4    1  c09
........................................................cagccacuaacgacacugcGucu.................................  1    1  c09
........................................................cagccacuaacgacacugcuucC.................................  4    1  c09
........................................................cagccacuaacgCcacugcuucu.................................  3    1  c09
........................................................cagccacuaacgacacugcuucug................................  1    0  c09
........................................................cagccacuaacgacacugcuucuU................................  3    1  c09
........................................................cagccacuaacgacacugcuucuA................................  1    1  c09
.........................................................agccacuaacgacacugc.....................................  71   0  c09
.........................................................agccacuaCcgacacugc.....................................  1    1  c09
.........................................................Cgccacuaacgacacugc.....................................  9    1  c09
.........................................................agccacuaacgacacugcu....................................  128  0  c09
.........................................................agccacuaacgacacugcC....................................  3    1  c09
.........................................................Cgccacuaacgacacugcu....................................  12   1  c09
.........................................................Cgccacuaacgacacugcuu...................................  13   1  c09
.........................................................agccacuaacgacacugAuu...................................  1    1  c09
.........................................................agccacuaacgacacAgcuu...................................  1    1  c09
.........................................................agccacuaacgGcacugcuu...................................  1    1  c09
.........................................................agccacuaacgacaUugcuu...................................  1    1  c09
.........................................................agccacuaacgacacugcuu...................................  113  0  c09
.........................................................Ggccacuaacgacacugcuuc..................................  1    1  c09
.........................................................agccacuaacgacacugcuuU..................................  2    1  c09
.........................................................agccacuaacgacacugcuuc..................................  111  0  c09
.........................................................agccUcuaacgacacugcuuc..................................  1    1  c09
.........................................................Cgccacuaacgacacugcuuc..................................  12   1  c09
.........................................................agccacuaacgacacugcuuA..................................  1    1  c09
.........................................................aCccacuaacgacacugcuucu.................................  2    1  c09
.........................................................agccacuaacCacacugcuucu.................................  1    1  c09
.........................................................agccacuaacgacaAugcuucu.................................  1    1  c09
.........................................................agccacuaacgacCcugcuucu.................................  3    1  c09
.........................................................agccacCaacgacacugcuucu.................................  1    1  c09
.........................................................agccacuaacgaUacugcuucu.................................  1    1  c09
.........................................................agccacuaacgacacugcuuAu.................................  1    1  c09
.........................................................Cgccacuaacgacacugcuucu.................................  195  1  c09
.........................................................agAcacuaacgacacugcuucu.................................  1    1  c09
.........................................................agccacuaacgacacuCcuucu.................................  1    1  c09
.........................................................agccacuaacgacacugcuucA.................................  4    1  c09
.........................................................agccacuaacgGcacugcuucu.................................  1    1  c09
.........................................................agccaUuaacgacacugcuucu.................................  2    1  c09
.........................................................agccacuaacgacacugcuucC.................................  4    1  c09
.........................................................agcAacuaacgacacugcuucu.................................  2    1  c09
.........................................................aUccacuaacgacacugcuucu.................................  2    1  c09
```

```
                    Mature                                  Star
gaaaauggguuagaccgcguuggcagugugguuagcugguuguuuauuauaugacaacagccacuaacgacacugcuucugcguacacccugauccuacauaucgacugacag
..........................................................agUcacuaacgacacugcuucu.................................   1     1  c09
..........................................................agccacuaacgacacugcuucu.................................   1529  0  c09
..........................................................Ugccacuaacgacacugcuucu.................................   1     1  c09
..........................................................agccacuaacgCcacugcuucu.................................   8     1  c09
..........................................................agccacuaCcgacacugcuucu.................................   2     1  c09
..........................................................Ggccacuaacgacacugcuucu.................................   1     1  c09
..........................................................agccacuaacgacacugAuucu.................................   2     1  c09
..........................................................agccacuaacgacacugcuAcu.................................   1     1  c09
..........................................................agccacuaacgacacCgcuucu.................................   1     1  c09
..........................................................agccacuCacgacacugcuucu.................................   1     1  c09
..........................................................agccacuaacgacacugcuucuC................................   3     1  c09
..........................................................agccacuaacgacacugcuucuA................................   11    1  c09
..........................................................agccacuaacgacacugUuucug................................   1     1  c09
..........................................................agccacuaacgCcacugcuucug................................   2     1  c09
..........................................................agccacuaacgacacugcuucuU................................   7     1  c09
..........................................................agccacuaacgacacugcuucug................................   3     0  c09
..........................................................agccacuaacgacacugcuucuAc...............................   1     1  c09
...........................................................gccacuaacgacacugcu....................................   1     0  c09
...........................................................gccacuaacgacacugcuuc..................................   1     0  c09
...........................................................gccacuaacgacacugcuucu.................................   8     0  c09
...........................................................gccacuaacgCcacugcuucu.................................   1     1  c09
............................................................ccacuaacgacacugcuucu.................................   3     0  c09
.............................................................cacuaacgacacugcuucu.................................   5     0  c09
..............................................................acuaacgacacugcuucu.................................   8     0  c09
..............................................................acuaacgacacugcuucug................................   1     0  c09

.................cUuuggcagugugguuagcugguu........................................................................   1     1  c16
.................cguuggcagugugguuagcugguug.......................................................................   1     0  c16
...................Cuggcagugugguuagcugguu........................................................................   6     1  c16
...................uuggcagugugguuagcugguu........................................................................   3     0  c16
...................Cuggcagugugguuagcugguug.......................................................................   2     1  c16
...................uuggcagugugguuagcugguugu......................................................................   3     0  c16
...................Auggcagugugguuagcugguugu......................................................................   1     1  c16
...................Guggcagugugguuagcugguugu......................................................................   1     1  c16
...................Cuggcagugugguuagcugguugu......................................................................   6     1  c16
...................uuggcagugugguuagcugguuguu.....................................................................   1     0  c16
...................Guggcagugugguuagcugguuguu.....................................................................   1     1  c16
...................Cuggcagugugguuagcugguuguuua...................................................................   1     1  c16
....................Gggcagugugguuagcug...........................................................................   8     1  c16
....................uggcagugugguuagcug...........................................................................   83    0  c16
....................uggcagugugguAagcugg..........................................................................   3     1  c16
....................Cggcagugugguuagcugg..........................................................................   3     1  c16
....................uggGagugugguuagcugg..........................................................................   1     1  c16
....................ugCcagugugguuagcugg..........................................................................   1     1  c16
....................uggcaguCugguuagcugg..........................................................................   1     1  c16
....................uggcagugugguuagcuUg..........................................................................   1     1  c16
....................uggUagugugguuagcugg..........................................................................   1     1  c16
....................uggcagugugAuuagcugg..........................................................................   2     1  c16
....................uggcagugugguuagcugg..........................................................................   1501  0  c16
....................uCgcagugugguuagcugg..........................................................................   2     1  c16
....................uggcagugugguuagcuCg..........................................................................   2     1  c16
....................uggcaguAugguuagcugg..........................................................................   1     1  c16
....................uggcGgugugguuagcugg..........................................................................   1     1  c16
....................uggAagugugguuagcugg..........................................................................   1     1  c16
....................uggcaCugugguuagcugg..........................................................................   2     1  c16
....................Aggcagugugguuagcugg..........................................................................   4     1  c16
....................uggcagugugguuaUcugg..........................................................................   1     1  c16
....................Gggcagugugguuagcugg..........................................................................   157   1  c16
....................uggcagugugguuagcugA..........................................................................   2     1  c16
....................uggcagugugguuaCcugg..........................................................................   1     1  c16
....................uggcagugugguuagcugU..........................................................................   4     1  c16
....................uggcUgugugguuagcugg..........................................................................   1     1  c16
....................uggcagugugCuuagcugg..........................................................................   2     1  c16
....................uggcagugugguuagcCgg..........................................................................   1     1  c16
....................uggcagugugguuagcAgg..........................................................................   1     1  c16
....................ugAcagugugguuagcugg..........................................................................   1     1  c16
....................uggcagugugguuagAugg..........................................................................   2     1  c16
....................uggcagugugguGagcuggu.........................................................................   1     1  c16
....................ugUcagugugguuagcuggu.........................................................................   1     1  c16
....................uggcagugugAuuagcuggu.........................................................................   1     1  c16
```

```
                    Mature                             Star
gaaaauggguuagaccgcguuggcagugugguuagcugguuguuuauuauaugacaacagccacuaacgacacugcuucugcguacacccugauccuacauaucgacugacag
....................ugCcagugugguuagcuggu.........................................................................  2     1  c16
....................Aggcagugugguuagcuggu.........................................................................  2     1  c16
....................uggcagugugguuagcuCgu.........................................................................  1     1  c16
....................uUgcagugugguuagcuggu.........................................................................  1     1  c16
....................uggcaguguAguuagcuggu.........................................................................  1     1  c16
....................uggcagugugguuagcuggu.........................................................................  1324  0  c16
....................uggcaguguggUAagcuggu.........................................................................  1     1  c16
....................uggcagAgugguuagcuggu.........................................................................  2     1  c16
....................uggcaguguCguuagcuggu.........................................................................  2     1  c16
....................Cggcagugugguuagcuggu.........................................................................  1     1  c16
....................Gggcagugugguuagcuggu.........................................................................  140   1  c16
....................uggcaguCugguuagcuggu.........................................................................  3     1  c16
....................uggAagugugguuagcuggu.........................................................................  2     1  c16
....................uggcaUugugguuagcuggu.........................................................................  1     1  c16
....................uggcaCugugguuagcuggu.........................................................................  2     1  c16
....................uggcagugugguuaCcuggu.........................................................................  3     1  c16
....................uCgcagugugguuagcuggu.........................................................................  2     1  c16
....................uggUagugugguuagcuggu.........................................................................  1     1  c16
....................uggcagugugguuagUuggu.........................................................................  1     1  c16
....................uggcagugugguuaUcuggu.........................................................................  1     1  c16
....................uggcagugugguuagcuggC.........................................................................  2     1  c16
....................uggcagugugguuagAuggu.........................................................................  2     1  c16
....................uggcagugugguuagcuggA.........................................................................  3     1  c16
....................uggcagugugCuagcuggu..........................................................................  1     1  c16
....................uggcagugugguuagcugUu.........................................................................  2     1  c16
....................uggcagGgugguuagcuggu.........................................................................  1     1  c16
....................uggcCgugugguuagcuggu.........................................................................  1     1  c16
....................uggcagugugguuagcuggG.........................................................................  2     1  c16
....................uggcaCugugguuagcugguu........................................................................  5     1  c16
....................uggcCgugugguuagcugguu........................................................................  1     1  c16
....................uggcaguguggAuagcugguu........................................................................  4     1  c16
....................uggcagugugguuagcugguA........................................................................  13    1  c16
....................uggcGgugugguuagcugguu........................................................................  5     1  c16
....................uggcagugugCuuagcugguu........................................................................  7     1  c16
....................uggcagugugguuagcugguu........................................................................  8614  0  c16
....................uggUagugugguuagcugguu........................................................................  8     1  c16
....................uUgcagugugguuagcugguu........................................................................  4     1  c16
....................uggcaguUugguuagcugguu........................................................................  5     1  c16
....................uggcaguAugguuagcugguu........................................................................  1     1  c16
....................uggcaguguCguuagcugguu........................................................................  3     1  c16
....................uggcagugGgguuagcugguu........................................................................  1     1  c16
....................uggcagugugguuagcGgguu........................................................................  2     1  c16
....................ugUcagugugguuagcugguu........................................................................  5     1  c16
....................Cggcagugugguuagcugguu........................................................................  14    1  c16
....................uAgcagugugguuagcugguu........................................................................  3     1  c16
....................uggcagugugguuagcAgguu........................................................................  2     1  c16
....................uggcagugugguuGgcugguu........................................................................  2     1  c16
....................Gggcagugugguuagcugguu........................................................................  833   1  c16
....................uggcaguguggCagcugguu.........................................................................  3     1  c16
....................uggcagGgugguuagcugguu........................................................................  1     1  c16
....................uggcaguguggUAagcugguu........................................................................  4     1  c16
....................uggcagugugguuagcugguG........................................................................  4     1  c16
....................uggcagugugguuaCcugguu........................................................................  9     1  c16
....................uggAagugugguuagcugguu........................................................................  2     1  c16
....................ugAcagugugguuagcugguu........................................................................  1     1  c16
....................uggcagugugGuagcugguu.........................................................................  5     1  c16
....................uggcagugugguuagGugguu........................................................................  2     1  c16
....................uggcagugugguuagcugguC........................................................................  4     1  c16
....................uggGagugugguuagcugguu........................................................................  2     1  c16
....................uggcaguUguuagcugguu..........................................................................  2     1  c16
....................uggcaAugugguuagcugguu........................................................................  2     1  c16
....................uggcagugugUuuagcugguu........................................................................  2     1  c16
....................uggcUgugugguuagcugguu........................................................................  4     1  c16
....................uggcaguAguuagcugguu..........................................................................  2     1  c16
....................uggcagugugguuCgcugguu........................................................................  1     1  c16
....................uggcagugugguuagcuggAu........................................................................  6     1  c16
....................ugCcagugugguuagcugguu........................................................................  8     1  c16
....................uggcagugugguuagcuUguu........................................................................  3     1  c16
....................uggcagugAgguuagcugguu........................................................................  7     1  c16
....................uggcaUugugguuagcugguu........................................................................  3     1  c16
```

Mature Star			
gaaaauggguuagaccgcguuggcagugugguuagcugguuguuuauuauaugacaacagccacuaacgacacugcuucugcguacacccugauccuacauaucgacugacag			
....................uggcagugugguuagUugguu..	5	1	c16
....................uCgcagugugguuagcugguu..	5	1	c16
....................uggcagugugguuUgcugguu..	1	1	c16
....................uggcagugugguuaUcugguu..	13	1	c16
....................uggcaguCugguuagcugguu..	20	1	c16
....................uggcagugugguuagcugUuu..	6	1	c16
....................uggcagAgugguuagcugguu..	5	1	c16
....................Aggcagugugguuagcugguu..	12	1	c16
....................uggcagugugguuagcuggGu..	3	1	c16
....................uggcagugugguuagcCgguu..	2	1	c16
....................uggcagugugguuagcugCuu..	7	1	c16
....................uggcagugugguGagcugguu..	1	1	c16
....................uggcagugugguuagAugguu..	9	1	c16
....................uggcaguguggCuagcugguu..	1	1	c16
....................uggcagugugguuagcugAuu..	1	1	c16
....................uggcagugugguuagcuCguu..	12	1	c16
....................uggUagugugguuagcugguug...	2	1	c16
....................uggcagugugguuagcuCguug...	2	1	c16
....................uggcagAgugguuagcugguug...	2	1	c16
....................uggGagugugguuagcugguug...	1	1	c16
....................uggcaguguggCuagcugguug...	1	1	c16
....................uggcagugugguuaCcugguug...	3	1	c16
....................uggcagugugguuaUcugguug...	1	1	c16
....................uggcagugAgguuagcugguug...	3	1	c16
....................uggcaguguggAuagcugguug...	1	1	c16
....................uggcagugugguGagcugguug...	1	1	c16
....................uAgcagugugguuagcugguug...	1	1	c16
....................ugCcagugugguuagcugguug...	1	1	c16
....................uCgcagugugguuagcugguug...	1	1	c16
....................uggcagugugguuagAugguug...	2	1	c16
....................uggcaguUugguuagcugguug...	1	1	c16
....................uggcGgugugguuagcugguug...	1	1	c16
....................uggcagugugUuuagcugguug...	1	1	c16
....................uggcagugugguuagcugCuug...	5	1	c16
....................uggcagugugCuuagcugguug...	2	1	c16
....................uggcagugugguuagcugguug...	1334	0	c16
....................uggcagugugguuagGugguug...	1	1	c16
....................uggcaguguCguuagcugguug...	2	1	c16
....................uggcaguguAguuagcugguug...	1	1	c16
....................uggcagGgugguuagcugguug...	1	1	c16
....................ugAcagugugguuagcugguug...	1	1	c16
....................uggcagugugguuagcugguGg...	1	1	c16
....................uggcagugugguuagcugguuA...	38	1	c16
....................uggcagugugguuagcugguuU...	1190	1	c16
....................Aggcagugugguuagcugguug...	1	1	c16
....................uggcagugugguuagcuggGug...	1	1	c16
....................Cggcagugugguuagcugguug...	1	1	c16
....................Gggcagugugguuagcugguug...	150	1	c16
....................uggcagugugguuagcugguuC...	17	1	c16
....................ugUcagugugguuagcugguug...	2	1	c16
....................uggcagugugguuagcuggAug...	2	1	c16
....................uggcaguCugguuagcugguug...	3	1	c16
....................uggcagugugguuagcugUuug...	1	1	c16
....................Cggcagugugguuagcugguugu..	17	1	c16
....................uggcagugugCuuagcugguugu..	12	1	c16
....................uggcagugugguuaCcugguugu..	7	1	c16
....................uggcagugugguAagcugguugu..	2	1	c16
....................uggcagugugguuagcugguugG..	7	1	c16
....................uggcagugugguuagcuggCugu..	4	1	c16
....................uggcagugugguuagcugCuugu..	18	1	c16
....................uggcagugugguuUgcugguugu..	1	1	c16
....................uggcagugugguuagcugUuugu..	7	1	c16
....................uggcagugugguuagcGgguugu..	4	1	c16
....................uggcagugugguuagGugguugu..	2	1	c16
....................uggcagugugguuagcugguAgu..	6	1	c16
....................uggcagugugguuagcuCguugu..	8	1	c16
....................uCgcagugugguuagcugguugu..	11	1	c16
....................uggcagugugguuaUcugguugu..	13	1	c16
....................uAgcagugugguuagcugguugu..	5	1	c16
....................uggcaAugugguuagcugguugu..	2	1	c16

```
                    Mature                 Star
gaaaauggguuagaccgcguuggcagugugguuagcugguuguuuauuauaugacaacagccacuaacgacacugcuucugcguacacccugauccuacauaucgacugacag
....................uggcagugugguuagcugguuUu......................................................................  188    1  c16
....................uggcaguguggAuagcugguugu......................................................................  2      1  c16
....................uggcagugugguuagcCgguugu......................................................................  4      1  c16
....................uggcaguguUguuagcugguugu......................................................................  2      1  c16
....................uggcaguguCguuagcugguugu......................................................................  14     1  c16
....................uggcagugugguuagcugguugu......................................................................  12947  0  c16
....................uggcaguCugguuagcugguugu......................................................................  17     1  c16
....................uggcaguAugguuagcugguugu......................................................................  4      1  c16
....................uggcagugugguuagcugAuugu......................................................................  1      1  c16
....................uggcGgugugguuagcugguugu......................................................................  5      1  c16
....................uggcagugugguuagcAgguugu......................................................................  4      1  c16
....................uggcagugugguuagcugguuAu......................................................................  3      1  c16
....................uggcaguguAguuagcugguugu......................................................................  3      1  c16
....................uggcaguguggCuagcugguugu......................................................................  7      1  c16
....................uggcagugugguuCgcugguugu......................................................................  1      1  c16
....................ugAcagugugguuagcugguugu......................................................................  4      1  c16
....................uggcagugugguuagcugguugA......................................................................  3      1  c16
....................uggAagugugguuagcugguugu......................................................................  7      1  c16
....................uggcagugCgguuagcugguugu......................................................................  3      1  c16
....................Aggcagugugguuagcugguugu......................................................................  27     1  c16
....................ugUcagugugguuagcugguugu......................................................................  5      1  c16
....................uggcaCugugguuagcugguugu......................................................................  12     1  c16
....................uggcaguUugguuagcugguugu......................................................................  3      1  c16
....................uggcagCgugguuagcugguugu......................................................................  11     1  c16
....................uggcagugugguuagcuggAugu......................................................................  9      1  c16
....................uUgcagugugguuagcugguugu......................................................................  1      1  c16
....................uggUagugugguuagcugguugu......................................................................  7      1  c16
....................uggcagugugAuuagcugguugu......................................................................  2      1  c16
....................uggcaguguggGuagcugguugu......................................................................  9      1  c16
....................uggcagGgugguuagcugguugu......................................................................  4      1  c16
....................ugCcagugugguuagcugguugu......................................................................  7      1  c16
....................Gggcagugugguuagcugguugu......................................................................  1346   1  c16
....................uggcagugAgguuagcugguugu......................................................................  7      1  c16
....................uggcagugugguuagUugguugu......................................................................  5      1  c16
....................uggcagugugguuagcugguugC......................................................................  1      1  c16
....................uggcagugugguuagcugguuCu......................................................................  25     1  c16
....................uggcagugugguCagcugguugu......................................................................  5      1  c16
....................uggcagugugguuagcuUguugu......................................................................  7      1  c16
....................uggcagugugguuagcugguCgu......................................................................  2      1  c16
....................uggcagugugguuagcuAguugu......................................................................  3      1  c16
....................uggGagugugguuagcugguugu......................................................................  6      1  c16
....................uggcagAgugguuagcugguugu......................................................................  11     1  c16
....................uggcagugugguGagcugguugu......................................................................  1      1  c16
....................uggcagugGgguuagcugguugu......................................................................  3      1  c16
....................uggcagugugguuGgcugguugu......................................................................  3      1  c16
....................uggcagugugUuuagcugguugu......................................................................  2      1  c16
....................uggcaUugugguuagcugguugu......................................................................  8      1  c16
....................uggcagugugguuagAugguugu......................................................................  17     1  c16
....................uggcagugugguuagcugguugGu.....................................................................  2      1  c16
....................uggcagugAgguuagcugguuguu.....................................................................  1      1  c16
....................Cggcagugugguuagcugguuguu.....................................................................  3      1  c16
....................uggcagugugguuagUugguuguu.....................................................................  1      1  c16
....................uggUagugugguuagcugguuguu.....................................................................  2      1  c16
....................uggcagugugguuagcugguAguu.....................................................................  1      1  c16
....................uggcagugugguuagcugguuguA.....................................................................  64     1  c16
....................uggcagugugguuagcuCguuguu.....................................................................  1      1  c16
....................uggcaguguCguuagcugguuguu.....................................................................  1      1  c16
....................uggcagugugguuagAugguuguu.....................................................................  2      1  c16
....................uggcagugugguuagcugguuguC.....................................................................  12     1  c16
....................uggcagugugguuagcugUuuguu.....................................................................  1      1  c16
....................uggcagugugguuagcugguuCuu.....................................................................  1      1  c16
....................uggcaCugugguuagcugguuguu.....................................................................  1      1  c16
....................uggcUgugugguuagcugguuguu.....................................................................  1      1  c16
....................Aggcagugugguuagcugguuguu.....................................................................  2      1  c16
....................uggcagugugguuagcugguuguu.....................................................................  685    0  c16
....................uggcagugugguuagcugguuguG.....................................................................  17     1  c16
....................Gggcagugugguuagcugguuguu.....................................................................  69     1  c16
....................uggcagugugguuagcugguuUuu.....................................................................  29     1  c16
....................uggcagugugguuagcuUguuguu.....................................................................  1      1  c16
....................uggcagugugguuagcugguuguuu....................................................................  99     0  c16
```

```
                    Mature                                      Star
gaaaauggguuagaccgcguuggcagugugguuagcugguuguuuauuauaugacaacagccacuaacgacacugcuucugcguacacccugauccuacauaucgacugacag
....................uggcagugugguuagcugguuguuC....................................................................    5     1     c16
....................uggcaguguCguuagcugguuguuu....................................................................    1     1     c16
....................uAgcagugugguuagcugguuguuu....................................................................    1     1     c16
....................uUgcagugugguuagcugguuguuu....................................................................    1     1     c16
....................Gggcagugugguuagcugguuguuu....................................................................    12    1     c16
....................uggcagugugguuagcugguuguCu....................................................................    3     1     c16
....................ugCcagugugguuagcugguuguuu....................................................................    1     1     c16
....................uggcagugugguuagcugguuguGu....................................................................    1     1     c16
....................uggcagugugguuaCcugguuguuu....................................................................    1     1     c16
....................uggcagugugguuagcugguuguuA....................................................................    7     1     c16
....................uggcagugugguuagcuggAuguuu....................................................................    1     1     c16
....................uggAagugugguuagcugguuguuu....................................................................    1     1     c16
....................uggcagugugguuagcugguuguuG....................................................................    2     1     c16
....................uggcagugugguuagcugguuguAu....................................................................    13    1     c16
....................uggcagugugguuagcugguuguuGa...................................................................    1     1     c16
....................uggcagugugguuagcugguuguuAa...................................................................    1     1     c16
....................uggcagugugguuagcugguuguuuU...................................................................    31    1     c16
....................uggcagugugguuagcugguuguuua...................................................................    4     0     c16
....................uggcagugugguuagcugguuguuAau..................................................................    1     1     c16
....................uggcagugugguuagcugguuguuuCu..................................................................    1     1     c16
....................uggcagugugguuagcugguuguuuUu..................................................................    11    1     c16
....................uggcagugugguuagcugguuguuCau..................................................................    1     1     c16
....................uggcagugugguuagcugguuguuuUuu.................................................................    2     1     c16
....................uggcagugugguuagcugguuguuAauu.................................................................    1     1     c16
.....................Ugcagugugguuagcugg..........................................................................    1     1     c16
.....................Ugcagugugguuagcuggu.........................................................................    1     1     c16
.....................ggcagugugguuagcugguu........................................................................    3     0     c16
.....................Ugcagugugguuagcugguugu......................................................................    3     1     c16
.....................ggcagugugguuagcugguugu......................................................................    3     0     c16
.....................ggUagugugguuagcugguugu......................................................................    1     1     c16
.....................ggcagugugguuagcugguuguu.....................................................................    9     0     c16
......................gcagugugguuagcuggu.........................................................................    2     0     c16
......................gcagugugguuagcugguu........................................................................    8     0     c16
......................gcagugugguuagcugguug.......................................................................    9     0     c16
......................gcaguguggGuagcugguugu......................................................................    1     1     c16
......................gcagugugCuuagcugguugu......................................................................    1     1     c16
......................gcagugugguuagcugguugu......................................................................    35    0     c16
......................gAagugugguuagcugguugu......................................................................    2     1     c16
......................gcagugugguuagcugguuguA.....................................................................    1     1     c16
......................Ccagugugguuagcugguuguu.....................................................................    1     1     c16
......................Ucagugugguuagcugguuguu.....................................................................    2     1     c16
......................gcagugugguuagcugCuuguu.....................................................................    1     1     c16
......................gcagugugguuagcugguuguu.....................................................................    183   0     c16
......................gcagugugguuagUugguuguu.....................................................................    1     1     c16
......................gcaguCugguuagcugguuguu.....................................................................    2     1     c16
......................Ucagugugguuagcugguuguuu....................................................................    2     1     c16
......................gcagugugguuagcugguuguuA....................................................................    1     1     c16
......................gcagugugguuagcugguAguuu....................................................................    1     1     c16
......................gcagugugCuuagcugguuguuu....................................................................    1     1     c16
......................gcagugugguuagcugguuguuu....................................................................    54    0     c16
......................gcagugugguuagcugguuguuC....................................................................    1     1     c16
......................gcagugugguuagcuUguuguuu....................................................................    1     1     c16
......................gcagugugguuagcGgguuguuu....................................................................    1     1     c16
......................gcagugugguuagcugguuguuuU...................................................................    13    1     c16
......................gcagugugguuagcugguuguuuC...................................................................    1     1     c16
......................gcagugugguuagcugguuguuAa...................................................................    2     1     c16
......................gcagugugguuagcugguuguuuUu..................................................................    2     1     c16
......................gcagugugguuagcugguuguuuaA..................................................................    2     1     c16
......................gcagugugguuagcugguuguuuUuu.................................................................    1     1     c16
.......................cagugugguuagcugguu........................................................................    2     0     c16
.......................cagugugguuagcuggCug.......................................................................    1     1     c16
.......................cagugugguuagcugguug.......................................................................    3     0     c16
.......................caCugugguuagcugguugu......................................................................    1     1     c16
.......................caUugugguuagcugguugu......................................................................    1     1     c16
.......................cagugugguuagcugguugu......................................................................    25    0     c16
.......................cagugugguuagcugguuguu.....................................................................    1     0     c16
........................agugugguuagcugguug.......................................................................    6     0     c16
........................aguUgugguuagcugguugu.....................................................................    1     1     c16
........................agugugguuagcugguugu......................................................................    25    0     c16
........................agugugguuagcugguuUu......................................................................    1     1     c16
```

Mature **Star**

```
gaaaauggguuagaccgcguuggcagugugguuagcugguuguuuauuauaugacaacagccacuaacgacacugcuucugcguacacccugauccuacauaucgacugacag
........................Cgugugguuagcugguugu......................................................................  1    1  c16
........................agugugguuagcugguuguu.....................................................................  9    0  c16
........................agugugguuagcugguuguuuau..................................................................  1    0  c16
.........................gugugguuagcugguuUu......................................................................  2    1  c16
.........................gugugguuagcugguugu......................................................................  16   0  c16
.........................gugugguuagcugguuguu.....................................................................  1    0  c16
..........................ugugguuagcugguuguu.....................................................................  1    0  c16
...........................gugguuagcugguugAuu....................................................................  1    1  c16
...........................gugguuagcugguuguuu....................................................................  2    0  c16
...........................................uuauuauaugacaacagUcacuaacgacacugcuucu.................................  1    1  c16
.....................................................acaacagccacuaacgacacugcuucu.................................  1    0  c16
........................................................acagccacuaacgacacugcuu...................................  1    0  c16
.........................................................cagccacuaacgacacugc.....................................  13   0  c16
.........................................................cagccacuaacgacacugcu....................................  7    0  c16
.........................................................cagccacuaacgacacugcuu...................................  64   0  c16
.........................................................cagccacuaacgacCcugcuu...................................  1    1  c16
.........................................................cagcUacuaacgacacugcuu...................................  2    1  c16
.........................................................cagccCcuaacgacacugcuu...................................  1    1  c16
.........................................................cagccacuaacgacacugcuuc..................................  184  0  c16
.........................................................cagcAacuaacgacacugcuuc..................................  1    1  c16
.........................................................cagccacuaacgacacugcuuU..................................  21   1  c16
.........................................................cagccacuaacgacacugAuuc..................................  1    1  c16
.........................................................caUccacuaacgacacugcuuc..................................  1    1  c16
.........................................................cagccacuaacgacCcugcuuc..................................  1    1  c16
.........................................................cagccacuaacgCcacugcuuc..................................  3    1  c16
.........................................................cCgccacuaacgacacugcuuc..................................  1    1  c16
.........................................................Uagccacuaacgacacugcuuc..................................  1    1  c16
.........................................................cagccacuaacgacUcugcuuc..................................  1    1  c16
.........................................................cagAcacuaacgacacugcuuc..................................  1    1  c16
.........................................................cagccacuaCcgacacugcuucu.................................  1    1  c16
.........................................................cagccacuaacgacacugcuucA.................................  2    1  c16
.........................................................cagccacuaacgacacugcuucC.................................  4    1  c16
.........................................................cagccacuaacgCcacugcuucu.................................  2    1  c16
.........................................................cagccacuaacgacacugcuucu.................................  64   0  c16
.........................................................cagccacuaacgacacugcuucug................................  1    0  c16
.........................................................cagccacuaacgacacugcuucuA................................  1    1  c16
.........................................................cagccacuaacgacacugcuucAg................................  1    1  c16
.........................................................cagccacuaacgacacugcuucuC................................  2    1  c16
..........................................................agccacuaacgacacugU.....................................  1    1  c16
..........................................................Cgccacuaacgacacugc.....................................  4    1  c16
..........................................................agccacuaacgacacugc.....................................  9    0  c16
..........................................................Cgccacuaacgacacugcu....................................  2    1  c16
..........................................................agccacuaacgacacugcu....................................  7    0  c16
..........................................................agccacuaacgacacugcuu...................................  11   0  c16
..........................................................agccacuaacgacacugcuA...................................  1    1  c16
..........................................................Cgccacuaacgacacugcuu...................................  10   1  c16
..........................................................agccacuaacgacacugcuuc..................................  23   0  c16
..........................................................Cgccacuaacgacacugcuuc..................................  8    1  c16
..........................................................Ggccacuaacgacacugcuuc..................................  1    1  c16
..........................................................aCccacuaacgacacugcuucu.................................  3    1  c16
..........................................................agccacuaacgacaUugcuucu.................................  1    1  c16
..........................................................Ugccacuaacgacacugcuucu.................................  1    1  c16
..........................................................agccacuaacgacacugcuGcu.................................  1    1  c16
..........................................................agccacuaacgacacugcuucu.................................  570  0  c16
..........................................................agccacuaacgacCcugcuucu.................................  2    1  c16
..........................................................agUcacuaacgacacugcuucu.................................  1    1  c16
..........................................................agccacuaacgacacugGuucu.................................  1    1  c16
..........................................................agccacuaCcgacacugcuucu.................................  2    1  c16
..........................................................agAcacuaacgacacugcuucu.................................  1    1  c16
..........................................................agccacuaacgacacugAuucu.................................  2    1  c16
..........................................................agccacuaacUacacugcuucu.................................  1    1  c16
..........................................................agccacuaacgacacugcuuGu.................................  1    1  c16
..........................................................Cgccacuaacgacacugcuucu.................................  217  1  c16
..........................................................agccacuaacgacacugcuucC.................................  1    1  c16
..........................................................Ggccacuaacgacacugcuucu.................................  1    1  c16
..........................................................agccaAuaacgacacugcuucu.................................  1    1  c16
..........................................................agccacuaacgacaGugcuucu.................................  1    1  c16
..........................................................agccacuaacgaAacugcuucu.................................  1    1  c16
..........................................................agccacuaacgCcacugcuucu.................................  2    1  c16
..........................................................agccacuaaAgacacugcuucu.................................  1    1  c16
```

Mature **Star**

gaaaauggguuagaccgcguuggcagugugguuagcugguuguuuauuauaugacaacagccacuaacgacacugcuucugcguacacccugauccuacauaucgacugacag			
....agccaAuaacgacacugcuucug................................	1	1	c16
....agccacuaacgacacugcuucuA................................	1	1	c16
....agccacuaacgacacugcuucug................................	1	0	c16
....agccacuaacgacacugcuucuU................................	3	1	c16
....agccacuaacgacacugcuucuC................................	1	1	c16
....Cgccacuaacgacacugcuucugcg..............................	1	1	c16
....gccacuaacgacacugcuu...................................	1	0	c16
....gccacuaacgacacugcuuc..................................	2	0	c16
....gccacuaacgacacugcuucu.................................	4	0	c16
....ccacuaacgacacugcuucu.................................	1	0	c16
....acuaacgacacugcuucu.................................	1	0	c16
................gcUuuggcagugugguuagcuggu...	1	1	c04
.................cgCuggcagugugguuagcugguug...	1	1	c04
.................cUuuggcagugugguuagcugguugu..	1	1	c04
...................Cuggcagugugguuagcu..	1	1	c04
...................Cuggcagugugguuagcugguu..	2	1	c04
...................Auggcagugugguuagcugguugu..	1	1	c04
...................uuggcagugugguuagcugguuguuuU...	1	1	c04
....................Gggcagugugguuagcug...	1	1	c04
....................uggcagugugguuagcug...	14	0	c04
....................uggcagugugguuagcuU...	1	1	c04
....................Gggcagugugguuagcugg..	5	1	c04
....................uggcagugugguuagcugg..	98	0	c04
....................uggcaguguAguuagcugg..	1	1	c04
....................uggcagugugguuagcugA..	1	1	c04
....................uggcagugugCuuagcugg..	1	1	c04
....................Aggcagugugguuagcugg..	1	1	c04
....................ugUcagugugguuagcuggu...	1	1	c04
....................Aggcagugugguuagcuggu...	1	1	c04
....................uggcagugugCuuagcuggu...	1	1	c04
....................uggcagugugguuagcuggA...	3	1	c04
....................uggcagugugguGagcuggu...	1	1	c04
....................uggcagugugguuagcuggu...	182	0	c04
....................uUgcagugugguuagcuggu...	1	1	c04
....................Gggcagugugguuagcuggu...	6	1	c04
....................uggcagugugguuaCcuggu...	1	1	c04
....................uCgcagugugguuagcuggu...	1	1	c04
....................uggcagugugguuaCcugguu..	1	1	c04
....................uggcagugugguuagcuggAu..	2	1	c04
....................uggcagugugguAagcugguu..	1	1	c04
....................Gggcagugugguuagcugguu..	49	1	c04
....................uggcagugugguuagcugCuu..	1	1	c04
....................uggcagugugguuagcugguA..	13	1	c04
....................uggcagugugguuagcuggGu..	1	1	c04
....................uggcagugugguuagcuCguu..	2	1	c04
....................uUgcagugugguuagcugguu..	1	1	c04
....................uggcaguguggCuagcugguu..	1	1	c04
....................uggcaCugugguuagcugguu..	1	1	c04
....................uggcagugugguuagcugguG..	2	1	c04
....................uggcagugugguuagcugguu..	750	0	c04
....................uggcaguguUguuagcugguu..	1	1	c04
....................uggcaguCugguuagcugguu..	1	1	c04
....................uCgcagugugguuagcugguu..	1	1	c04
....................uggcagugugguuagcugguC..	1	1	c04
....................uggcagAgugguuagcugguu..	1	1	c04
....................uAgcagugugguuagcugguu..	1	1	c04
....................uggcaguguCguuagcugguu..	1	1	c04
....................Aggcagugugguuagcugguug...	1	1	c04
....................uggcagugugguuagcugguug...	196	0	c04
....................uggcagugugguuagcugguuA...	24	1	c04
....................uggcagCgugguuagcugguug...	1	1	c04
....................uggcagugugguuagcGgguug...	1	1	c04
....................uggUagugugguuagcugguug...	2	1	c04
....................uggcagugugguuagcugguuU...	82	1	c04
....................uUgcagugugguuagcugguug...	1	1	c04
....................Gggcagugugguuagcugguug...	14	1	c04
....................uggcagugugguuagcugguuC...	1	1	c04
....................ugUcagugugguuagcugguug...	1	1	c04
....................uggcagugugguuaUcugguug...	1	1	c04

```
                    Mature                                      Star
gaaaauggguuagaccgcguuggcagugugguuagcugguuguuuauuauaugacaacagccacuaacgacacugcuucugcguacacccugauccuacauaucgacugacag
....................uggcaUugugguuagcugguug.......................................................................    1      1      c04
....................uggcagugugguuagcugAuugu......................................................................    1      1      c04
....................uggcaguUugguuagcugguugu......................................................................    2      1      c04
....................uCgcagugugguuagcugguugu......................................................................    2      1      c04
....................uAgcagugugguuagcugguugu......................................................................    1      1      c04
....................uggcagugugguuagcugguuCu......................................................................    1      1      c04
....................uggcagugugguuagcugguuAu......................................................................    1      1      c04
....................uggcagugugguuaCcugguugu......................................................................    2      1      c04
....................uggcagugugguuUgcugguugu......................................................................    1      1      c04
....................uggcaguguCguuagcugguugu......................................................................    1      1      c04
....................Aggcagugugguuagcugguugu......................................................................    3      1      c04
....................uggcagugugCuuagcugguugu......................................................................    2      1      c04
....................uggcagAgugguuagcugguugu......................................................................    1      1      c04
....................uggcagugugguuagcugguugC......................................................................    3      1      c04
....................uggcaguguggCuagcugguugu......................................................................    1      1      c04
....................uggcaguguggGuagcugguugu......................................................................    1      1      c04
....................uggcagugugguuagcuCguugu......................................................................    3      1      c04
....................uggcagugugguuagcugUuugu......................................................................    3      1      c04
....................uggcagugugguuagcugguCgu......................................................................    1      1      c04
....................uggcaguguAguuagcugguugu......................................................................    1      1      c04
....................uggcagugugguuagcugguugG......................................................................    1      1      c04
....................ugUcagugugguuagcugguugu......................................................................    1      1      c04
....................uggUagugugguuagcugguugu......................................................................    2      1      c04
....................uggcagugugguuCgcugguugu......................................................................    2      1      c04
....................uggcagugugguuagcugguugA......................................................................    2      1      c04
....................Cggcagugugguuagcugguugu......................................................................    1      1      c04
....................uggcagCgugguuagcugguugu......................................................................    1      1      c04
....................uggcagugugguuagUugguugu......................................................................    4      1      c04
....................uggcagugugguuagcugCuugu......................................................................    2      1      c04
....................uggcaguguggAuagcugguugu......................................................................    1      1      c04
....................uggAagugugguuagcugguugu......................................................................    1      1      c04
....................uggcagugugguAagcugguugu......................................................................    1      1      c04
....................uggcagugugguuagcugguAgu......................................................................    2      1      c04
....................uggcagugugguuagcugguugu......................................................................    1905   0      c04
....................uggcagugugguuaUcugguugu......................................................................    1      1      c04
....................uggcaCugugguuagcugguugu......................................................................    1      1      c04
....................uggcagugugguuGgcugguugu......................................................................    1      1      c04
....................uggcaguCugguuagcugguugu......................................................................    1      1      c04
....................uggcagugugguuagcugguuUu......................................................................    15     1      c04
....................uggGagugugguuagcugguugu......................................................................    1      1      c04
....................uggcagugugguuagcuggAugu......................................................................    3      1      c04
....................uUgcagugugguuagcugguugu......................................................................    1      1      c04
....................Gggcagugugguuagcugguugu......................................................................    114    1      c04
....................uggcagugugAuuagcugguugu......................................................................    1      1      c04
....................uggcaguCugguuagcugguuguu.....................................................................    1      1      c04
....................Gggcagugugguuagcugguuguu.....................................................................    5      1      c04
....................uggcagugugguuagcuAguuguu.....................................................................    2      1      c04
....................uggcagugugguuagcugguuguA.....................................................................    172    1      c04
....................uggcagugugguuagcugguuUuu.....................................................................    2      1      c04
....................uggcagugugguuagcugguuguG.....................................................................    3      1      c04
....................uggcagugugguuagcugguugCu.....................................................................    1      1      c04
....................uggcagugugguuagcugguuCuu.....................................................................    1      1      c04
....................uggcagugugguuagcugguuguu.....................................................................    58     0      c04
....................uggcagugugguuagcugguuguC.....................................................................    2      1      c04
....................uggcagugugguuagcugguuguuC....................................................................    3      1      c04
....................uggcagugugguuagcugguuguuu....................................................................    13     0      c04
....................uggcagugugguuagcugguuguuG....................................................................    3      1      c04
....................uggcagugugguuagcugguuguGu....................................................................    1      1      c04
....................uggcagugugguuagcugguuguuA....................................................................    11     1      c04
....................uggcagugugguuagcugguuguAu....................................................................    11     1      c04
....................uggcagugugguuagcugguuguAua...................................................................    2      1      c04
....................uggcagugugguuagcugguuguuGa...................................................................    1      1      c04
....................uggcagugugguuagcugguuguuAa...................................................................    4      1      c04
....................uggcagugugguuagcugguuguuuUu..................................................................    3      1      c04
.....................Ugcagugugguuagcuggu.........................................................................    1      1      c04
.....................Ugcagugugguuagcugguugu......................................................................    1      1      c04
.....................ggcagugugguuagcugguugu......................................................................    3      0      c04
.....................ggcagugugguuagcugguuguu.....................................................................    1      0      c04
......................gcagugugguuagcuggu.........................................................................    1      0      c04
......................gcagugugguuagcugguu........................................................................    2      0      c04
```

```
                    Mature                                  Star
gaaaauggguuagaccgcguuggcagugugguuagcugguuguuuauuauaugacaacagccacuaacgacacugcuucugcguacacccugauccuacauaucgacugacag
......................gcagugugguuagcugguugu......................................................................   12   0   c04
......................gcagugugguuagcugCuuguu.....................................................................   1    1   c04
......................gcagugugguuagcugguuguu.....................................................................   16   0   c04
......................gcagugugguuagcugguuguuu....................................................................   2    0   c04
......................gcagugugguuagcugguuguuA....................................................................   7    1   c04
......................gcagugugguuagcugguuguuAa...................................................................   2    1   c04
.......................cagugugguuagcugguu........................................................................   1    0   c04
.......................cagugugguuagcugguugu......................................................................   2    0   c04
.......................cagugugguuagcugguuguA.....................................................................   1    1   c04
........................agugugguuagcugguuA.......................................................................   1    1   c04
........................agugugguuagcugguugu......................................................................   1    0   c04
........................agAgugguuagcugguugu......................................................................   1    1   c04
.........................gugugguuagcugguugu......................................................................   4    0   c04
.........................gugugguuagcugguuguA.....................................................................   1    1   c04
.........................gugugguuagcugguuguuuU...................................................................   1    1   c04
.........................................................cagccacuaacgacacugcu....................................   1    0   c04
.........................................................cagccacuaacgacacugcuu...................................   3    0   c04
.........................................................cagccacuaacgacacugcuuU..................................   2    1   c04
.........................................................cagccacuaacgacacugcuuc..................................   12   0   c04
.........................................................cagccacuaacUacacugcuuc..................................   1    1   c04
.........................................................cagccacuaacgacacugcuucu.................................   4    0   c04
..........................................................agccacuaacgacacuUc.....................................   1    1   c04
..........................................................agccacuaacgacacugc.....................................   5    0   c04
..........................................................agccacuaacgacacugcu....................................   2    0   c04
..........................................................Cgccacuaacgacacugcuu...................................   1    1   c04
..........................................................agccacuaacgacacugcuu...................................   4    0   c04
..........................................................agccacuaacgacacugcuuc..................................   4    0   c04
..........................................................agccacuaacgacacugcAucu.................................   1    1   c04
..........................................................agccacuaacgacacugcuAcu.................................   1    1   c04
..........................................................Cgccacuaacgacacugcuucu.................................   16   1   c04
..........................................................agccacuaacgacacugAuucu.................................   1    1   c04
..........................................................agccacuaacgacacugcuucu.................................   83   0   c04
..........................................................agccacuaacgacacugcuGcu.................................   1    1   c04
..........................................................agGcacuaacgacacugcuucu.................................   1    1   c04
..........................................................agccacuaacgacacugcuucuU................................   3    1   c04

...................uuggcagugugguuagcugg..........................................................................   1    0   c15
...................Cuggcagugugguuagcuggu.........................................................................   1    1   c15
....................uggcagugugguuagcug...........................................................................   1    0   c15
....................uggcagugugguuagcugg..........................................................................   145  0   c15
....................uggcagugugguuagUugg..........................................................................   1    1   c15
....................uggcagugugguuagcugA..........................................................................   2    1   c15
....................uggcagugugguuagcugU..........................................................................   1    1   c15
....................uggcGgugugguuagcugg..........................................................................   1    1   c15
....................uggcagCgugguuagcugg..........................................................................   1    1   c15
....................uggcagugugguuagAugg..........................................................................   1    1   c15
....................uggcagugugguuagcuggC.........................................................................   1    1   c15
....................uggcagugugguuagAuggu.........................................................................   1    1   c15
....................uggcagugugguuagcuggA.........................................................................   1    1   c15
....................uggcagugugguuagcuggu.........................................................................   116  0   c15
....................Aggcagugugguuagcuggu.........................................................................   1    1   c15
....................uggcaUugugguuagcugguu........................................................................   1    1   c15
....................uggcagugugguuagcugguu........................................................................   245  0   c15
....................uggcagugugguuagcugAuu........................................................................   3    1   c15
....................uggcagCgugguuagcugguu........................................................................   1    1   c15
....................ugAcagugugguuagcugguu........................................................................   1    1   c15
....................uggcagugugguuagcuggAu........................................................................   1    1   c15
....................uggAagugugguuagcugguug.......................................................................   1    1   c15
....................uggcagugGgguuagcugguug.......................................................................   1    1   c15
....................uggcagugugguuagcugguuC.......................................................................   3    1   c15
....................uggcagugugguuagcugguuU.......................................................................   24   1   c15
....................uggcaguAugguuagcugguug.......................................................................   1    1   c15
....................uggcagugugguuagcugguug.......................................................................   80   0   c15
....................uggcagugugguuagcugguuA.......................................................................   2    1   c15
....................uggcagGgugguuagcugguugu......................................................................   4    1   c15
....................uggcagugugguuagcCgguugu......................................................................   3    1   c15
....................uggcagugugguuagcuggAugu......................................................................   1    1   c15
....................uggcagugugguuagcugguuAu......................................................................   2    1   c15
....................uggcagugGgguuagcugguugu......................................................................   2    1   c15
....................uggcagugugguuagUugguugu......................................................................   1    1   c15
```

Mature **Star**

```
gaaaauggguuagaccgcguuggcagugugguuagcugguuguuuauuauaugacaacagccacuaacgacacugcuucugcguacacccugauccuacauaucgacugacag
....................uggcaguguUguuagcugguugu......................................................................  1     1  c15
....................uggcagugugguuagcuUguugu......................................................................  1     1  c15
....................uggcagugugguuagcuggCugu......................................................................  3     1  c15
....................Gggcagugugguuagcugguugu......................................................................  1     1  c15
....................uUgcagugugguuagcugguugu......................................................................  1     1  c15
....................uggcaguguggAuagcugguugu......................................................................  1     1  c15
....................uggcagugCgguuagcugguugu......................................................................  1     1  c15
....................ugAcagugugguuagcugguugu......................................................................  1     1  c15
....................uggcagugugguuagcuggGugu......................................................................  2     1  c15
....................uggcagCgugguuagcugguugu......................................................................  1     1  c15
....................uggcGgugugguuagcugguugu......................................................................  2     1  c15
....................uggcagugugguuaUcugguugu......................................................................  2     1  c15
....................uggcagugugguCagcugguugu......................................................................  1     1  c15
....................uggcagugugguuaAcugguugu......................................................................  1     1  c15
....................uggcagugugguuagcugguugA......................................................................  13    1  c15
....................Cggcagugugguuagcugguugu......................................................................  4     1  c15
....................uggcagugugAuuagcugguugu......................................................................  1     1  c15
....................uggcagugugguuagAugguugu......................................................................  2     1  c15
....................uggcagugugguuagcugAuugu......................................................................  1     1  c15
....................uggcagugugguAagcugguugu......................................................................  1     1  c15
....................uggcagugugguuagcugguugu......................................................................  2779  0  c15
....................uggcagugugguuagcugguuUu......................................................................  10    1  c15
....................Aggcagugugguuagcugguugu......................................................................  2     1  c15
....................uggcagugAgguuagcugguugu......................................................................  4     1  c15
....................uggcaguAugguuagcugguugu......................................................................  1     1  c15
....................uggcagugugguuGgcugguugu......................................................................  2     1  c15
....................uggUagugugguuagcugguugu......................................................................  1     1  c15
....................uggcagugugguuagcugguugC......................................................................  9     1  c15
....................uAgcagugugguuagcugguugu......................................................................  3     1  c15
....................ugAcagugugguuagcugguuguu.....................................................................  1     1  c15
....................uggcagugugguuagcugguuguA.....................................................................  7     1  c15
....................uggcagugugguuaUcugguuguu.....................................................................  1     1  c15
....................uggcagugugguuagcugguugAu.....................................................................  1     1  c15
....................uggcagugugguuagcugguuguu.....................................................................  152   0  c15
....................uggcagugugguuagcugguuguC.....................................................................  3     1  c15
....................uggcagugugguuagcugguuguuu....................................................................  26    0  c15
....................uggcagugugguuagcugguuguuA....................................................................  8     1  c15
....................uggcagugugguuagcugguuguAu....................................................................  1     1  c15
....................uggcagugugguuagcugguuguuuC...................................................................  1     1  c15
....................uggcagugugguuagcugguuguuuU...................................................................  5     1  c15
....................uggcagugugguuagcugguuguuuUu..................................................................  1     1  c15
....................uggcagugugguuagcugguuguuuau..................................................................  1     0  c15
....................uggcagugugguuagcugguuguuuaAu.................................................................  1     1  c15
.....................ggcagugugguuagcugguugu......................................................................  3     0  c15
.....................ggcagugugguuagcugguuguu.....................................................................  1     0  c15
......................gcagugugguuagcugguugu......................................................................  2     0  c15
......................gcagugugguuagcugguuguu.....................................................................  3     0  c15
......................gcagugugguuagcugguuguuC....................................................................  1     1  c15
...........................gugguuagcugguugA......................................................................  1     1  c15
.........................................................cagccacuaacgacacugcuu...................................  3     0  c15
.........................................................cagccacuaacgacaGugcuuc..................................  1     1  c15
.........................................................Uagccacuaacgacacugcuuc..................................  1     1  c15
.........................................................cagccacuaacgacacugcuuU..................................  8     1  c15
.........................................................cagccacuaacgacacugcuuc..................................  45    0  c15
.........................................................cagccacuaacgacacugcuucA.................................  1     1  c15
.........................................................cagccacuaacgacacugcuucu.................................  21    0  c15
.........................................................cagccacuaacgacacugcuucuU................................  2     1  c15
..........................................................agccacuaacgacacugc.....................................  1     0  c15
..........................................................agccacuaacgacacugcuu...................................  2     0  c15
..........................................................agccacuaacgacacugcuuc..................................  33    0  c15
..........................................................Ugccacuaacgacacugcuucu.................................  1     1  c15
..........................................................agUcacuaacgacacugcuucu.................................  1     1  c15
..........................................................aUccacuaacgacacugcuucu.................................  1     1  c15
..........................................................Ggccacuaacgacacugcuucu.................................  1     1  c15
..........................................................agccacuaacgacacugcuucA.................................  12    1  c15
..........................................................agccacuaacgacacugcuucG.................................  1     1  c15
..........................................................agccacuaaAgacacugcuucu.................................  1     1  c15
..........................................................agccacuaacgacacugcuGcu.................................  1     1  c15
..........................................................agccacuaacgacacugcuucu.................................  467   0  c15
..........................................................agccacuaacgacacugcuuUu.................................  1     1  c15
```

Mature Star

```
gaaaauggguuagaccgcguuggcagugugguuagcugguuguuuauuauaugacaacagccacuaacgacacugcuucugcguacacccugauccuacauaucgacugacag
..........................................................agccacuaacgacacugcuucC.................................   1      1      c15
..........................................................agccacuaacgacacugcuucuU................................   5      1      c15
..........................................................agccacuaacgacacugcuucuA................................   1      1      c15
..........................................................agccacuaacgacacugcuucugU...............................   1      1      c15
...........................................................gccacuaacgacacugcuuc..................................   1      0      c15
...........................................................gccacuaacgacacugcuucu.................................   4      0      c15
.............................................................cacuaacgacacugcuucu.................................   2      0      c15

................gcUuuggcagugugguuagcugg..........................................................................   1      1      c02
................gcUuuggcagugugguuagcugguu........................................................................   1      1      c02
................gcUuuggcagugugguuagcugguugu......................................................................   1      1      c02
................gcgCuggcagugugguuagcugguugu......................................................................   1      1      c02
................gGguuggcagugugguuagcugguugu......................................................................   1      1      c02
.................cUuuggcagugugguuagcugguug.......................................................................   1      1      c02
.................cCuuggcagugugguuagcugguugu......................................................................   1      1      c02
.................cgCuggcagugugguuagcugguugu......................................................................   1      1      c02
.................cUuuggcagugugguuagcugguugu......................................................................   1      1      c02
..................gCuggcagugugguuagcugguug.......................................................................   1      1      c02
..................gGuggcagugugguuagcugguugu......................................................................   3      1      c02
..................Auuggcagugugguuagcugguugu......................................................................   2      1      c02
..................Cuuggcagugugguuagcugguugu......................................................................   3      1      c02
..................gGuggcagugugguuagcugguuguu.....................................................................   1      1      c02
...................uuggcagugugguuagcuggu.........................................................................   4      0      c02
...................Cuggcagugugguuagcugguu........................................................................   1      1      c02
...................uuggcagugugguuagcugguu........................................................................   8      0      c02
...................Guggcagugugguuagcugguu........................................................................   1      1      c02
...................Guggcagugugguuagcugguug.......................................................................   4      1      c02
...................uuggcagugugguuagcugguug.......................................................................   4      0      c02
...................Cuggcagugugguuagcugguug.......................................................................   3      1      c02
...................uuggcagugugguuagcugguuU.......................................................................   2      1      c02
...................Guggcagugugguuagcugguugu......................................................................   4      1      c02
...................uuggcagugugCuuagcugguugu......................................................................   1      1      c02
...................uuggcagugugguuagcugguugG......................................................................   1      1      c02
...................Auggcagugugguuagcugguugu......................................................................   7      1      c02
...................Cuggcagugugguuagcugguugu......................................................................   11     1      c02
...................uuggcagugugguuagcugguugu......................................................................   35     0      c02
...................uuggcaUugugguuagcugguugu......................................................................   1      1      c02
...................Guggcagugugguuagcugguuguu.....................................................................   1      1      c02
...................uuggcagugugguuagcugguuguu.....................................................................   11     0      c02
...................uuggcagugugguuagcugguuguuu....................................................................   1      0      c02
....................uggUagugugguuagcug...........................................................................   1      1      c02
....................uggcaguguAguuagcug...........................................................................   1      1      c02
....................uggcaguguCguuagcug...........................................................................   1      1      c02
....................uggcagugugguuagcuC...........................................................................   5      1      c02
....................uggcagugugguuagcug...........................................................................   376    0      c02
....................uggcagugugUuuagcug...........................................................................   1      1      c02
....................uggcaCugugguuagcug...........................................................................   6      1      c02
....................ugUcagugugguuagcug...........................................................................   1      1      c02
....................uggcagugugguuGgcug...........................................................................   1      1      c02
....................uggcagugugguuaUcug...........................................................................   1      1      c02
....................uUgcagugugguuagcug...........................................................................   1      1      c02
....................Cggcagugugguuagcug...........................................................................   1      1      c02
....................Gggcagugugguuagcug...........................................................................   30     1      c02
....................uggcagugugguuCgcug...........................................................................   1      1      c02
....................uggcagugugguuaUcugg..........................................................................   5      1      c02
....................uggcagugugAuuagcugg..........................................................................   2      1      c02
....................uggcagugugguuagcAgg..........................................................................   1      1      c02
....................uggcaAugugguuagcugg..........................................................................   2      1      c02
....................uggcaCugugguuagcugg..........................................................................   4      1      c02
....................uggcagugugguuagcugg..........................................................................   4803   0      c02
....................uggcagugugguuagcugU..........................................................................   3      1      c02
....................uggcaguAugguuagcugg..........................................................................   2      1      c02
....................uggcagugugguuagcCgg..........................................................................   3      1      c02
....................Cggcagugugguuagcugg..........................................................................   3      1      c02
....................uggcagAgugguuagcugg..........................................................................   3      1      c02
....................uggcaUugugguuagcugg..........................................................................   1      1      c02
....................ugUcagugugguuagcugg..........................................................................   1      1      c02
....................uggGagugugguuagcugg..........................................................................   2      1      c02
....................Gggcagugugguuagcugg..........................................................................   457    1      c02
....................uggcagugAgguuagcugg..........................................................................   1      1      c02
```

```
                    Mature                 Star
gaaaauggguuagaccgcguuggcagugugguuagcugguuguuuauuauaugacaacagccacuaacgacacugcuucugcguacacccugauccuacauaucgacugacag
....................uggcagugugguGagcugg..........................................................................  1     1  c02
....................uggcagugugguuagcugA..........................................................................  1     1  c02
....................uggcaguguAguuagcugg..........................................................................  1     1  c02
....................uggcagugugguuagUugg..........................................................................  2     1  c02
....................uggcaguCugguuagcugg..........................................................................  6     1  c02
....................uggUagugugguuagcugg..........................................................................  3     1  c02
....................uggAagugugguuagcugg..........................................................................  1     1  c02
....................uggcagugugUuuagcugg..........................................................................  2     1  c02
....................uggcagugugCuuagcugg..........................................................................  3     1  c02
....................Aggcagugugguuagcugg..........................................................................  11    1  c02
....................uggcagugCgguuagcugg..........................................................................  2     1  c02
....................uAgcagugugguuagcugg..........................................................................  1     1  c02
....................uggcaguguggGuagcugg..........................................................................  1     1  c02
....................uggcagugugguuagcugC..........................................................................  3     1  c02
....................uggcagugugguuaCcugg..........................................................................  2     1  c02
....................uggcagugugguCagcugg..........................................................................  2     1  c02
....................uggcUgugugguuagcugg..........................................................................  1     1  c02
....................uggcaguguUguuagcugg..........................................................................  3     1  c02
....................uggcaguguggAuagcugg..........................................................................  5     1  c02
....................uggcaguguCguuagcugg..........................................................................  5     1  c02
....................uggcaguguggCuagcugg..........................................................................  2     1  c02
....................uggcagugugguuGgcugg..........................................................................  1     1  c02
....................uggcagugugguuagAugg..........................................................................  7     1  c02
....................uggcGgugugguuagcugg..........................................................................  2     1  c02
....................uggcagugugguuagcuCg..........................................................................  2     1  c02
....................uggcagugugguuagcuAg..........................................................................  1     1  c02
....................uCgcagugugguuagcugg..........................................................................  3     1  c02
....................uggcagugugguuagcuggC.........................................................................  3     1  c02
....................uggcagugugAuuagcuggu.........................................................................  1     1  c02
....................Aggcagugugguuagcuggu.........................................................................  7     1  c02
....................uggcaguguggAuagcuggu.........................................................................  1     1  c02
....................uCgcagugugguuagcuggu.........................................................................  3     1  c02
....................uggcagCgugguuagcuggu.........................................................................  1     1  c02
....................uggcagugGgguuagcuggu.........................................................................  3     1  c02
....................uggcagugugguuagGuggu.........................................................................  2     1  c02
....................uggcagugugguuagcuCgu.........................................................................  2     1  c02
....................uggcGgugugguuagcuggu.........................................................................  2     1  c02
....................uggcaguAugguuagcuggu.........................................................................  1     1  c02
....................uggcagugugguuagcuggA.........................................................................  25    1  c02
....................uUgcagugugguuagcuggu.........................................................................  1     1  c02
....................uggcaguCugguuagcuggu.........................................................................  6     1  c02
....................uggcagugugguuagcugUu.........................................................................  10    1  c02
....................uggcagugugguuaCcuggu.........................................................................  4     1  c02
....................uggcagugugguuagcugAu.........................................................................  1     1  c02
....................uggcagugugguuagcuggu.........................................................................  4306  0  c02
....................Cggcagugugguuagcuggu.........................................................................  9     1  c02
....................uggcagugugguuagcGggu.........................................................................  1     1  c02
....................uggcaguUugguuagcuggu.........................................................................  1     1  c02
....................uAgcagugugguuagcuggu.........................................................................  1     1  c02
....................ugCcagugugguuagcuggu.........................................................................  3     1  c02
....................uggcagugugguuagcuUgu.........................................................................  4     1  c02
....................uggcagugugguuagAuggu.........................................................................  8     1  c02
....................uggcaguguggCuagcuggu.........................................................................  2     1  c02
....................uggcagugAgguuagcuggu.........................................................................  1     1  c02
....................uggcagugugCuuagcuggu.........................................................................  1     1  c02
....................uggcagGgugguuagcuggu.........................................................................  2     1  c02
....................uggcaguguggGuagcuggu.........................................................................  2     1  c02
....................uggcUgugugguuagcuggu.........................................................................  2     1  c02
....................uggUagugugguuagcuggu.........................................................................  4     1  c02
....................uggcagugugguuagUuggu.........................................................................  2     1  c02
....................uggcagugugUuuagcuggu.........................................................................  1     1  c02
....................uggcagugugguuagcugCu.........................................................................  2     1  c02
....................uggcagugugguuagcuggG.........................................................................  3     1  c02
....................uggcagugugguuaAcuggu.........................................................................  1     1  c02
....................uggcagugugguuagcAggu.........................................................................  2     1  c02
....................uggGagugugguuagcuggu.........................................................................  1     1  c02
....................uggcCgugugguuagcuggu.........................................................................  1     1  c02
....................uggcaguguCguuagcuggu.........................................................................  6     1  c02
....................Gggcagugugguuagcuggu.........................................................................  431   1  c02
....................uggcagAgugguuagcuggu.........................................................................  3     1  c02
```

Mature **Star**

```
gaaaauggguuagaccgcguuggcagugugguuagcugguuguuuauuauaugacaacagccacuaacgacacugcuucugcguacacccugauccuacauaucgacugacag
....................uggcaUugugguuagcuggu.........................................................................   7       1   c02
....................uggcagugugguuGgcuggu.........................................................................   1       1   c02
....................uggcagugugguuaUcuggu.........................................................................   3       1   c02
....................uggcagugugguuagcCggu.........................................................................   4       1   c02
....................uggcaCugugguuagcuggu.........................................................................   2       1   c02
....................uggcagugugguuagcuAgu.........................................................................   5       1   c02
....................uggAagugugguuagcuggu.........................................................................   1       1   c02
....................uggcagugugguuaUcugguu........................................................................   29      1   c02
....................uggcagugugguuagcuggGu........................................................................   7       1   c02
....................uggcagugugguuagcugCuu........................................................................   27      1   c02
....................uggcagugugguuagcugguA........................................................................   42      1   c02
....................uggcagugugguuagcuggAu........................................................................   17      1   c02
....................uggcagugugguuagGugguu........................................................................   2       1   c02
....................uggcagugugguuCgcugguu........................................................................   4       1   c02
....................uggcaAugugguuagcugguu........................................................................   2       1   c02
....................uggcagugugUuuagcugguu........................................................................   13      1   c02
....................uggcaguguggAuagcugguu........................................................................   18      1   c02
....................uggcaguguCguuagcugguu........................................................................   25      1   c02
....................uggcagugugguuagcuCguu........................................................................   34      1   c02
....................uggcagugugguuagcuggCu........................................................................   5       1   c02
....................uggcagugugguuGgcugguu........................................................................   7       1   c02
....................uggcagugugguuagcugguG........................................................................   7       1   c02
....................uggGagugugguuagcugguu........................................................................   12      1   c02
....................uggcagugugguuagcugguu........................................................................   26179   0   c02
....................uggcagugugguuUgcugguu........................................................................   4       1   c02
....................uggcaguguggGuagcugguu........................................................................   14      1   c02
....................ugUcagugugguuagcugguu........................................................................   14      1   c02
....................uggcagugugCuuagcugguu........................................................................   31      1   c02
....................uggUagugugguuagcugguu........................................................................   36      1   c02
....................uggcaguCugguuagcugguu........................................................................   33      1   c02
....................uggcaguguUguuagcugguu........................................................................   9       1   c02
....................uggcagugGgguuagcugguu........................................................................   7       1   c02
....................uggcagugugguuaCcugguu........................................................................   25      1   c02
....................Cggcagugugguuagcugguu........................................................................   29      1   c02
....................uggcaguguAguuagcugguu........................................................................   5       1   c02
....................uggcagugugguuagcuAguu........................................................................   5       1   c02
....................uggcagCgugguuagcugguu........................................................................   5       1   c02
....................uggcaguUugguuagcugguu........................................................................   10      1   c02
....................uggcagugugguAagcugguu........................................................................   6       1   c02
....................uggAagugugguuagcugguu........................................................................   14      1   c02
....................uggcaUugugguuagcugguu........................................................................   22      1   c02
....................uggcagugugguuagAugguu........................................................................   34      1   c02
....................uggcagugugguuagUugguu........................................................................   23      1   c02
....................uggcaguAugguuagcugguu........................................................................   13      1   c02
....................uggcagugAgguuagcugguu........................................................................   15      1   c02
....................uggcagGgugguuagcugguu........................................................................   8       1   c02
....................uggcagugCgguuagcugguu........................................................................   9       1   c02
....................uggcaCugugguuagcugguu........................................................................   14      1   c02
....................uggcagugugguuagcuUguu........................................................................   4       1   c02
....................uggcagugugguuagcCgguu........................................................................   10      1   c02
....................uggcagugugguuagcugAuu........................................................................   12      1   c02
....................uggcagugugguuagcugguC........................................................................   10      1   c02
....................uggcaguguggCuagcugguu........................................................................   3       1   c02
....................uggcagugugAuuagcugguu........................................................................   3       1   c02
....................uCgcagugugguuagcugguu........................................................................   25      1   c02
....................uAgcagugugguuagcugguu........................................................................   18      1   c02
....................uggcagugugguuaAcugguu........................................................................   2       1   c02
....................ugCcagugugguuagcugguu........................................................................   22      1   c02
....................uggcGgugugguuagcugguu........................................................................   8       1   c02
....................uggcagAgugguuagcugguu........................................................................   15      1   c02
....................uggcUgugugguuagcugguu........................................................................   5       1   c02
....................uggcagugugguuagcAgguu........................................................................   3       1   c02
....................uggcagugugguGagcugguu........................................................................   7       1   c02
....................uUgcagugugguuagcugguu........................................................................   20      1   c02
....................Aggcagugugguuagcugguu........................................................................   42      1   c02
....................ugAcagugugguuagcugguu........................................................................   10      1   c02
....................uggcCgugugguuagcugguu........................................................................   1       1   c02
....................Gggcagugugguuagcugguu........................................................................   2789    1   c02
....................uggcagugugguCagcugguu........................................................................   1       1   c02
....................uggcagugugguuagcugUuu........................................................................   11      1   c02
```

```
                    Mature                                    Star
gaaaauggguuagaccgcguuggcagugugguuagcugguuguuuauuauaugacaacagccacuaacgacacugcuucugcguacacccugauccuacauaucgacugacag
....................uggcagugugguuagcGgguu........................................................................ 2     1  c02
....................Aggcagugugguuagcugguug....................................................................... 15    1  c02
....................Gggcagugugguuagcugguug....................................................................... 898   1  c02
....................uggcagugugAuuagcugguug....................................................................... 2     1  c02
....................uggcagugugguuagcugguuU....................................................................... 798   1  c02
....................ugCcagugugguuagcugguug....................................................................... 7     1  c02
....................ugAcagugugguuagcugguug....................................................................... 5     1  c02
....................uggcagGgugguuagcugguug....................................................................... 2     1  c02
....................uggcaUugugguuagcugguug....................................................................... 7     1  c02
....................uggcaAugugguuagcugguug....................................................................... 1     1  c02
....................uggcagugugguuaUcugguug....................................................................... 5     1  c02
....................uggcagugugguuagcCgguug....................................................................... 3     1  c02
....................uggcagugGgguuagcugguug....................................................................... 3     1  c02
....................uggcagugugguuagAugguug....................................................................... 9     1  c02
....................uggcagugugguuagcuAguug....................................................................... 1     1  c02
....................uggcaguguCguuagcugguug....................................................................... 4     1  c02
....................uCgcagugugguuagcugguug....................................................................... 2     1  c02
....................uggGagugugguuagcugguug....................................................................... 5     1  c02
....................uggcagugugguuagcuUguug....................................................................... 2     1  c02
....................uggcaguguggGuagcugguug....................................................................... 2     1  c02
....................uggcUgugugguuagcugguug....................................................................... 2     1  c02
....................uggcagugugguuGgcugguug....................................................................... 5     1  c02
....................uggcagAgugguuagcugguug....................................................................... 7     1  c02
....................uggAagugugguuagcugguug....................................................................... 4     1  c02
....................uggcagugugguuUgcugguug....................................................................... 2     1  c02
....................uggcagugugguuagcugUuug....................................................................... 1     1  c02
....................uggcagugAgguuagcugguug....................................................................... 4     1  c02
....................uggcagugugguuagcuCguug....................................................................... 5     1  c02
....................uggcagugugguuagcugguug....................................................................... 9154  0  c02
....................uggcagugugCuuagcugguug....................................................................... 7     1  c02
....................Cggcagugugguuagcugguug....................................................................... 5     1  c02
....................uUgcagugugguuagcugguug....................................................................... 3     1  c02
....................uggcaguguAguuagcugguug....................................................................... 1     1  c02
....................uggcagugugguuaAcugguug....................................................................... 2     1  c02
....................uggcagugugguuagcugAuug....................................................................... 1     1  c02
....................uggcagugugguuagcugguuA....................................................................... 163   1  c02
....................uggcagugugguuagcuggGug....................................................................... 3     1  c02
....................uggcagugugguuaCcugguug....................................................................... 6     1  c02
....................uggcagugugguuCgcugguug....................................................................... 1     1  c02
....................uggcagugugguuagcugguAg....................................................................... 3     1  c02
....................uAgcagugugguuagcugguug....................................................................... 5     1  c02
....................uggcagugugguuagcAgguug....................................................................... 2     1  c02
....................uggcaCugugguuagcugguug....................................................................... 10    1  c02
....................uggcaguAugguuagcugguug....................................................................... 3     1  c02
....................uggcaguguggAuagcugguug....................................................................... 7     1  c02
....................uggcagugugguuagcugguCg....................................................................... 1     1  c02
....................uggcagugugUuuagcugguug....................................................................... 2     1  c02
....................ugUcagugugguuagcugguug....................................................................... 5     1  c02
....................uggcagugugguuagcugCuug....................................................................... 18    1  c02
....................uggcaguguUguuagcugguug....................................................................... 2     1  c02
....................uggUagugugguuagcugguug....................................................................... 14    1  c02
....................uggcagCgugguuagcugguug....................................................................... 2     1  c02
....................uggcagugCgguuagcugguug....................................................................... 2     1  c02
....................uggcaguCugguuagcugguug....................................................................... 9     1  c02
....................uggcagugugguuagcuggAug....................................................................... 4     1  c02
....................uggcagugugguGagcugguug....................................................................... 3     1  c02
....................uggcagugugguuagUugguug....................................................................... 6     1  c02
....................uggcagugugguuagcugguGg....................................................................... 3     1  c02
....................uggcGgugugguuagcugguug....................................................................... 7     1  c02
....................uggcagugugguuagcugguuC....................................................................... 46    1  c02
....................uggcagugugguAagcugguug....................................................................... 2     1  c02
....................uggcagugugguuagcuggCug....................................................................... 1     1  c02
....................uggcaguUugguuagcugguug....................................................................... 5     1  c02
....................uggcagugugguuaUcugguugu...................................................................... 88    1  c02
....................uggcagugugguuagcGgguugu...................................................................... 28    1  c02
....................uggcagugugguuCgcugguugu...................................................................... 4     1  c02
....................uggcCgugugguuagcugguugu...................................................................... 10    1  c02
....................uggcagugugguuagcuCguugu...................................................................... 108   1  c02
....................uUgcagugugguuagcugguugu...................................................................... 69    1  c02
....................uggcagugugguuagcugCuugu...................................................................... 161   1  c02
```

```
                    Mature                 Star
gaaaauggguuagaccgcguuggcagugugguuagcugguuguuuauuauaugacaacagccacuaacgacacugcuucugcguacacccugauccuacauaucgacugacag
....................uggcagugugguAagcugguugu......................................................................   25      1   c02
....................uggcagugugguuagcuUguugu......................................................................   28      1   c02
....................uggcagugugguuagcAgguugu......................................................................   38      1   c02
....................uggcagugugguGagcugguugu......................................................................   29      1   c02
....................uggcaguUugguuagcugguugu......................................................................   54      1   c02
....................uggcagugAgguuagcugguugu......................................................................   71      1   c02
....................uggcagugugguuagcugguugA......................................................................   156     1   c02
....................uggcaUugugguuagcugguugu......................................................................   76      1   c02
....................Gggcagugugguuagcugguugu......................................................................   13200   1   c02
....................uggcagGgugguuagcugguugu......................................................................   31      1   c02
....................uggcaCugugguuagcugguugu......................................................................   108     1   c02
....................uggcagugugUuuagcugguugu......................................................................   33      1   c02
....................uggcaguguCguuagcugguugu......................................................................   94      1   c02
....................uggcagugugguuGgcugguugu......................................................................   22      1   c02
....................uggcagugugguuagcuggCugu......................................................................   28      1   c02
....................uggcagugugguuagcugguCgu......................................................................   31      1   c02
....................uggcagugugguCagcugguugu......................................................................   23      1   c02
....................uggcagugugguuagGugguugu......................................................................   11      1   c02
....................Cggcagugugguuagcugguugu......................................................................   115     1   c02
....................uAgcagugugguuagcugguugu......................................................................   61      1   c02
....................uggcagugugAuuagcugguugu......................................................................   27      1   c02
....................uggcagugugguuagcugguugC......................................................................   37      1   c02
....................uggcagugugguuagcugguuAu......................................................................   27      1   c02
....................uggcagugugguuagcugguugG......................................................................   44      1   c02
....................uggcagugugguuagcCgguugu......................................................................   48      1   c02
....................uggcagCgugguuagcugguugu......................................................................   29      1   c02
....................uCgcagugugguuagcugguugu......................................................................   103     1   c02
....................uggcaguguggAuagcugguugu......................................................................   83      1   c02
....................uggcagugugguuagcugguuUu......................................................................   133     1   c02
....................uggcUgugugguuagcugguugu......................................................................   31      1   c02
....................uggcagugugguuaAcugguugu......................................................................   21      1   c02
....................uggUagugugguuagcugguugu......................................................................   138     1   c02
....................uggcaguguUguuagcugguugu......................................................................   39      1   c02
....................uggcagugugguuagAugguugu......................................................................   166     1   c02
....................uggcagugugguuagUugguugu......................................................................   78      1   c02
....................uggcagugGgguuagcugguugu......................................................................   29      1   c02
....................uggcagugugguuagcugUuugu......................................................................   71      1   c02
....................uggcGgugugguuagcugguugu......................................................................   41      1   c02
....................uggcaguCugguuagcugguugu......................................................................   157     1   c02
....................uggcagugugguuagcugguAgu......................................................................   41      1   c02
....................uggcaAugugguuagcugguugu......................................................................   20      1   c02
....................uggcagugugguuagcuggGugu......................................................................   32      1   c02
....................ugCcagugugguuagcugguugu......................................................................   87      1   c02
....................uggcagugugguuagcugguuCu......................................................................   118     1   c02
....................ugUcagugugguuagcugguugu......................................................................   81      1   c02
....................uggcaguAugguuagcugguugu......................................................................   59      1   c02
....................uggcagAgugguuagcugguugu......................................................................   56      1   c02
....................uggcagugugguuagcuggAugu......................................................................   65      1   c02
....................uggcagugugCuuagcugguugu......................................................................   110     1   c02
....................uggcagugugguuagcugguGgu......................................................................   14      1   c02
....................Aggcagugugguuagcugguugu......................................................................   290     1   c02
....................uggcagugugguuaCcugguugu......................................................................   96      1   c02
....................uggcaguguggCuagcugguugu......................................................................   19      1   c02
....................uggcagugugguuagcuAguugu......................................................................   22      1   c02
....................uggGagugugguuagcugguugu......................................................................   34      1   c02
....................uggcagugugguuagcugguugu......................................................................   127723  0   c02
....................ugAcagugugguuagcugguugu......................................................................   45      1   c02
....................uggcagugugguuUgcugguugu......................................................................   16      1   c02
....................uggcagugCgguuagcugguugu......................................................................   26      1   c02
....................uggcaguguggGuagcugguugu......................................................................   64      1   c02
....................uggcagugugguuagcugAuugu......................................................................   24      1   c02
....................uggAagugugguuagcugguugu......................................................................   70      1   c02
....................uggcaguguAguuagcugguugu......................................................................   27      1   c02
....................uggcGgugugguuagcugguuguu.....................................................................   2       1   c02
....................uggcagugugguuagcuUguuguu.....................................................................   1       1   c02
....................uggcagugugguuagcugguuUuu.....................................................................   9       1   c02
....................uggUagugugguuagcugguuguu.....................................................................   2       1   c02
....................uggAagugugguuagcugguuguu.....................................................................   2       1   c02
....................uggcagugugguuaUcugguuguu.....................................................................   5       1   c02
....................uggcagugugguuagUugguuguu.....................................................................   1       1   c02
```

```
                    Mature                                      Star
gaaaauggguuagaccgcguuggcagugugguuagcugguuguuuauuauaugacaacagccacuaacgacacugcuucugcguacacccugauccuacauaucgacugacag
....................uggcagugugguuagcCgguuguu.....................................................................    1     1     c02
....................uggcagugugguAagcugguuguu.....................................................................    1     1     c02
....................uggcagugugguuagcugguGguu.....................................................................    1     1     c02
....................uggcagugugguuagcugCuuguu.....................................................................    2     1     c02
....................uggcagugugCuuagcugguuguu.....................................................................    4     1     c02
....................uggcagugugguuagcuggAuguu.....................................................................    1     1     c02
....................uggcagugugguuagcugguuguu.....................................................................    3627  0     c02
....................uggcagugugguuagcuCguuguu.....................................................................    3     1     c02
....................ugCcagugugguuagcugguuguu.....................................................................    4     1     c02
....................uUgcagugugguuagcugguuguu.....................................................................    6     1     c02
....................ugAcagugugguuagcugguuguu.....................................................................    1     1     c02
....................uggcagAgugguuagcugguuguu.....................................................................    4     1     c02
....................Cggcagugugguuagcugguuguu.....................................................................    5     1     c02
....................uCgcagugugguuagcugguuguu.....................................................................    3     1     c02
....................uggGagugugguuagcugguuguu.....................................................................    2     1     c02
....................uggcagugugguuagcugUuuguu.....................................................................    1     1     c02
....................uggcagugugguuagcuAguuguu.....................................................................    2     1     c02
....................uggcagugugguuagcugguugAu.....................................................................    2     1     c02
....................uggcagCgugguuagcugguuguu.....................................................................    3     1     c02
....................uggcagugugguuagcuggCuguu.....................................................................    1     1     c02
....................uggcagugugguuagcGgguuguu.....................................................................    1     1     c02
....................uggcagugugguuagAugguuguu.....................................................................    3     1     c02
....................ugUcagugugguuagcugguuguu.....................................................................    1     1     c02
....................uggcaguUugguuagcugguuguu.....................................................................    2     1     c02
....................uggcagugugguuagcugguugGu.....................................................................    7     1     c02
....................uggcaguguCguuagcugguuguu.....................................................................    4     1     c02
....................uggcagugugguuagcugguuCuu.....................................................................    5     1     c02
....................uggcagugugguuagcugguuguC.....................................................................    113   1     c02
....................uggcagugugguuagcugguAguu.....................................................................    3     1     c02
....................uggcagugugguuagcugguuguA.....................................................................    1117  1     c02
....................uggcagugAgguuagcugguuguu.....................................................................    1     1     c02
....................uggcaCugugguuagcugguuguu.....................................................................    2     1     c02
....................uggcaguguggAuagcugguuguu.....................................................................    4     1     c02
....................uAgcagugugguuagcugguuguu.....................................................................    2     1     c02
....................uggcagugugguuaCcugguuguu.....................................................................    5     1     c02
....................uggcagugugguuagcugguuguG.....................................................................    171   1     c02
....................uggcaguAugguuagcugguuguu.....................................................................    2     1     c02
....................uggcUgugugguuagcugguuguu.....................................................................    1     1     c02
....................uggcaguguAguuagcugguuguu.....................................................................    2     1     c02
....................uggcaguguggGuagcugguuguu.....................................................................    3     1     c02
....................uggcaguguggCuagcugguuguu.....................................................................    1     1     c02
....................uggcaguCugguuagcugguuguu.....................................................................    9     1     c02
....................Gggcagugugguuagcugguuguu.....................................................................    355   1     c02
....................uggcaguguUguuagcugguuguu.....................................................................    1     1     c02
....................uggcagugGgguuagcugguuguu.....................................................................    4     1     c02
....................uggcagugugguuCgcugguuguu.....................................................................    2     1     c02
....................Aggcagugugguuagcugguuguu.....................................................................    9     1     c02
....................uggcagugugguuagcugguAguuu....................................................................    1     1     c02
....................uggcaguguCguuagcugguuguuu....................................................................    1     1     c02
....................uggcagugugguuagcugguuguuA....................................................................    59    1     c02
....................ugAcagugugguuagcugguuguuu....................................................................    1     1     c02
....................Gggcagugugguuagcugguuguuu....................................................................    15    1     c02
....................uggcagugugguuagcugguuUuuu....................................................................    2     1     c02
....................uggcagugugguuagcugguuguuu....................................................................    125   0     c02
....................uggcagugugguuagcugguuguAu....................................................................    34    1     c02
....................uggcaguguggGuagcugguuguuu....................................................................    1     1     c02
....................uggcagugugguuagcugguuguuG....................................................................    10    1     c02
....................uggcaguUugguuagcugguuguuu....................................................................    1     1     c02
....................uggcagugugguuGgcugguuguuu....................................................................    1     1     c02
....................uggcagugugguuagcugguuguuC....................................................................    7     1     c02
....................uggcagugugguuagcugguugAuu....................................................................    2     1     c02
....................uggcagugugguuagcugguuguGu....................................................................    12    1     c02
....................uggcagugugguuagcAgguuguuu....................................................................    1     1     c02
....................uggcagugugguuagcugguuguCu....................................................................    1     1     c02
....................uggcagugugguAagcugguuguuu....................................................................    1     1     c02
....................uggcagugugguuagcugguuguuua...................................................................    5     0     c02
....................uggcagugugguuagcugguuguuuU...................................................................    25    1     c02
....................uggcagugugguuagcugguuguuuG...................................................................    1     1     c02
....................uggcagugugguuagcugguuguAua...................................................................    1     1     c02
....................uggcagugugguuagcuggCuguuua...................................................................    1     1     c02
```

Mature   Star

```
gaaaauggguuagaccgcguuggcagugugguuagcugguuguuuauuauaugacaacagccacuaacgacacugcuucugcguacacccugauccuacauaucgacugacag
....................uggcagugugguuagcugguuguuAa...................................................................  8     1   c02
....................uggcagugugguuagcugguuguuuC...................................................................  1     1   c02
....................uggcagugugguuagcugguuguuuau..................................................................  4     0   c02
....................uggcagugugguuagcugguuguuuGu..................................................................  1     1   c02
....................uggcagugugguuagcugguuguuGau..................................................................  1     1   c02
....................uggcagugugguuagcugguuguuuaA..................................................................  4     1   c02
....................uggcagugugguuagcugguuguuuUu..................................................................  1     1   c02
....................uggcagugugguuagcugguuguuuauuauaugacaac.......................................................  5     0   c02
....................uggcagugugguuagcugguuguuuauuauaugacaacU......................................................  1     1   c02
.....................Ugcagugugguuagcugg..........................................................................  2     1   c02
.....................ggcagugugguuagcuggu.........................................................................  1     0   c02
.....................Ugcagugugguuagcugguu........................................................................  5     1   c02
.....................ggcagugugguuagcugguu........................................................................  4     0   c02
.....................ggcagGgugguuagcugguu........................................................................  1     1   c02
.....................Ugcagugugguuagcugguug.......................................................................  2     1   c02
.....................ggcagugugguuagcugguug.......................................................................  3     0   c02
.....................ggUagugugguuagcugguugu......................................................................  1     1   c02
.....................ggcagugugguuagcugguugu......................................................................  46    0   c02
.....................Ugcagugugguuagcugguugu......................................................................  31    1   c02
.....................Cgcagugugguuagcugguugu......................................................................  1     1   c02
.....................ggcagugugguuagcugguuguu.....................................................................  108   0   c02
.....................ggcagugAgguuagcugguuguu.....................................................................  1     1   c02
.....................Ugcagugugguuagcugguuguu.....................................................................  1     1   c02
.....................gUcagugugguuagcugguuguu.....................................................................  1     1   c02
.....................ggcagugugguuagcugguuguC.....................................................................  1     1   c02
.....................ggcagugugguuagcugAuuguu.....................................................................  1     1   c02
.....................ggcagugugguuagcugguuguuA....................................................................  1     1   c02
.....................ggcagugugguuagcugguuguuu....................................................................  3     0   c02
.....................ggcagugugguuagcugguuguuC....................................................................  1     1   c02
.....................ggcagugugguuagcugguuguuuauuaua..............................................................  1     0   c02
......................gcagugugguuagcuggu.........................................................................  5     0   c02
......................gcagugugguuagcugguu........................................................................  36    0   c02
......................Ucagugugguuagcugguu........................................................................  2     1   c02
......................gcaguCugguuagcugguug.......................................................................  1     1   c02
......................gcagugugguuagcugguug.......................................................................  27    0   c02
......................Ucagugugguuagcugguug.......................................................................  1     1   c02
......................gUagugugguuagcugguugu......................................................................  1     1   c02
......................gcagugugguuagcugguugu......................................................................  240   0   c02
......................Ucagugugguuagcugguugu......................................................................  2     1   c02
......................gcagugAgguuagcugguugu......................................................................  1     1   c02
......................gcUgugugguuagcugguugu......................................................................  1     1   c02
......................gcagugugguuagcugguugA......................................................................  1     1   c02
......................gcagugugguuagcugguAgu......................................................................  1     1   c02
......................Ccagugugguuagcugguugu......................................................................  1     1   c02
......................gcagugugguuagcugguuguu.....................................................................  1910  0   c02
......................gUagugugguuagcugguuguu.....................................................................  1     1   c02
......................gcagugugguuCgcugguuguu.....................................................................  1     1   c02
......................gcagugugguuagcugAuuguu.....................................................................  1     1   c02
......................gcagugugguuagcugguuCuu.....................................................................  1     1   c02
......................gcagugugguuagcuAguuguu.....................................................................  1     1   c02
......................gcaguguggAuagcugguuguu.....................................................................  2     1   c02
......................gcagugugguuagcugCuuguu.....................................................................  3     1   c02
......................Ccagugugguuagcugguuguu.....................................................................  7     1   c02
......................Ucagugugguuagcugguuguu.....................................................................  20    1   c02
......................gcagugugCuuagcugguuguu.....................................................................  2     1   c02
......................gcagugugguuagUugguuguu.....................................................................  1     1   c02
......................gcagCgugguuagcugguuguu.....................................................................  1     1   c02
......................gcagugugguuagcugguuguG.....................................................................  1     1   c02
......................gcagugugAuuagcugguuguu.....................................................................  1     1   c02
......................Acagugugguuagcugguuguu.....................................................................  1     1   c02
......................gcagugugguuagcuggCuguu.....................................................................  1     1   c02
......................gcagugugguuagcuCguuguu.....................................................................  2     1   c02
......................gcaguUugguuagcugguuguu.....................................................................  1     1   c02
......................gcagugugguuagAugguuguu.....................................................................  6     1   c02
......................gcagugCgguuagcugguuguu.....................................................................  1     1   c02
......................gcaCugugguuagcugguuguu.....................................................................  1     1   c02
......................gcaguCugguuagcugguuguu.....................................................................  3     1   c02
......................gcaguguUguuagcugguuguu.....................................................................  1     1   c02
......................gcagugugguuUgcugguuguu.....................................................................  2     1   c02
......................gcagugugguuagcugguuguA.....................................................................  4     1   c02
```

```
                    Mature                                   Star
gaaaauggguuagaccgcguuggcagugugguuagcugguuguuuauuauaugacaacagccacuaacgacacugcuucugcguacacccugauccuacauaucgacugacag
......................gcagAgugguuagcugguuguu.....................................................................    1    1    c02
......................gcagugugguuagcugguuguuC....................................................................    2    1    c02
......................gcagugugguuagcugCuuguuu....................................................................    1    1    c02
......................gcagugugguuagcugguuCuuu....................................................................    2    1    c02
......................gcagugugguuagcugguuguuA....................................................................    17   1    c02
......................gcagugugguuagcugguuguuu....................................................................    76   0    c02
......................Ccagugugguuagcugguuguuu....................................................................    1    1    c02
......................gcagugugguuagcugguuguuG....................................................................    4    1    c02
......................gcagugugguuagcugguuguuua...................................................................    4    0    c02
......................gcagugugguuagcugguuguuuU...................................................................    10   1    c02
......................gcagugugguuagcugguuguuuUu..................................................................    1    1    c02
......................gcagugugguuagcugguuguuuau..................................................................    2    0    c02
.......................cagugugguuagcuCguu........................................................................    1    1    c02
.......................cagugugguuagcugguu........................................................................    19   0    c02
.......................cagugugguuagcugguuU.......................................................................    2    1    c02
.......................cagugugguuagcugguug.......................................................................    11   0    c02
.......................cagugugguuaCcugguugu......................................................................    2    1    c02
.......................cagugugguuagcugguugA......................................................................    1    1    c02
.......................cagAgugguuagcugguugu......................................................................    2    1    c02
.......................cCgugugguuagcugguugu......................................................................    1    1    c02
.......................cagugugguuagcugguuCu......................................................................    1    1    c02
.......................caguCugguuagcugguugu......................................................................    1    1    c02
.......................cagugugguuagcugCuugu......................................................................    1    1    c02
.......................caguguggAuagcugguugu......................................................................    1    1    c02
.......................cagugugguuagcugguugu......................................................................    477  0    c02
.......................cGgugugguuagcugguugu......................................................................    1    1    c02
.......................cagugugguuagcugguuguu.....................................................................    12   0    c02
.......................cagugugguuagcugguuguA.....................................................................    4    1    c02
........................agugugguuagcugguuU.......................................................................    1    1    c02
........................agugugguuagcugguug.......................................................................    8    0    c02
........................agugugguuagcugguugu......................................................................    284  0    c02
........................agugugguuaUcugguugu......................................................................    2    1    c02
........................agAgugguuagcugguugu......................................................................    1    1    c02
........................aUugugguuagcugguugu......................................................................    6    1    c02
........................aguAugguuagcugguugu......................................................................    1    1    c02
........................aAugugguuagcugguugu......................................................................    3    1    c02
........................Ggugugguuagcugguugu......................................................................    4    1    c02
........................aCugugguuagcugguugu......................................................................    1    1    c02
........................aguUugguuagcugguugu......................................................................    3    1    c02
........................aguCugguuagcugguugu......................................................................    4    1    c02
........................aguguggAuagcugguugu......................................................................    1    1    c02
........................agugugguuagcugguuUu......................................................................    1    1    c02
........................Cgugugguuagcugguugu......................................................................    12   1    c02
........................agugugguuagcugguuguu.....................................................................    6    0    c02
........................agugugguuagcugguuguA.....................................................................    2    1    c02
........................agugugguuagcugguuguuu....................................................................    1    0    c02
........................agugugguuagcugguuguuua...................................................................    1    0    c02
........................agugugguuagcugguuguuuauuaua..............................................................    1    0    c02
........................agugugguuagcugguuguuuauuauau.............................................................    1    0    c02
........................agugugguuagcugguuguuuauuauaugaca.........................................................    1    0    c02
........................Cgugugguuagcugguuguuuauuauaugaca.........................................................    1    1    c02
.........................gAgugguuagcugguugu......................................................................    5    1    c02
.........................guUugguuagcugguugu......................................................................    1    1    c02
.........................gugugguuagAugguugu......................................................................    1    1    c02
.........................gugugUuuagcugguugu......................................................................    1    1    c02
.........................Cugugguuagcugguugu......................................................................    3    1    c02
.........................gGgugguuagcugguugu......................................................................    2    1    c02
.........................gugugguuagcugguuUu......................................................................    1    1    c02
.........................Uugugguuagcugguugu......................................................................    3    1    c02
.........................gugugguuagcugguugu......................................................................    331  0    c02
.........................gCgugguuagcugguuguu.....................................................................    1    1    c02
.........................gugugguuagcugguuguu.....................................................................    16   0    c02
.........................gugugguuagcugguuguA.....................................................................    3    1    c02
.........................gugugguuagcugguuguG.....................................................................    1    1    c02
.........................gugugguuagcugguuguuA....................................................................    1    1    c02
.........................gugugguuagcugguuguuuauu.................................................................    1    0    c02
..........................ugugguuagcugguuguA.....................................................................    1    1    c02
..........................ugugguuagcugguuguu.....................................................................    6    0    c02
..........................ugugguuagcugguuguuu....................................................................    1    0    c02
............................................uauuauaugacaacagccacuaacgac..........................................    2    0    c02
```

```
                    Mature                Star
gaaaauggguuagaccgcguuggcagugugguuagcugguuguuuauuauaugacaacagccacuaacgacacugcuucugcguacacccugauccuacauaucgacugacag
...............................................uauaugacaacagccacuaacgacacugcuucu.................................   1     0   c02
.................................................uaugacaacagccacuaacgacacugcuucu.................................   1     0   c02
...................................................ugacaacagccacuaacgacacugcuucu.................................   1     0   c02
.......................................................aUcagccacuaacgacacugcuucu.................................   1     1   c02
........................................................acagccacuaacgacacugcu....................................   1     0   c02
........................................................acagccacuaacgacacugcuA...................................   1     1   c02
........................................................acagccacuaacgacacugcuu...................................   1     0   c02
........................................................acagccacuaacgGcacugcuu...................................   1     1   c02
........................................................Ccagccacuaacgacacugcuu...................................   1     1   c02
........................................................acagccacuaacgacacugcuucu.................................   1     0   c02
.........................................................cagccacuaacgacacug......................................   13    0   c02
.........................................................cagccacuaacgacacugc.....................................   11    0   c02
.........................................................cagccacuaacgacacugcu....................................   17    0   c02
.........................................................Uagccacuaacgacacugcuu...................................   1     1   c02
.........................................................cagccacuaacgCcacugcuu...................................   2     1   c02
.........................................................cCgccacuaacgacacugcuu...................................   1     1   c02
.........................................................cagccacuaacgacacugcuu...................................   66    0   c02
.........................................................cagccacuaCcgacacugcuuc..................................   4     1   c02
.........................................................cagccacuaacgacCcugcuuc..................................   2     1   c02
.........................................................cagccacuaacgacacCgcuuc..................................   2     1   c02
.........................................................cagcAacuaacgacacugcuuc..................................   1     1   c02
.........................................................cagccacuaacgacacugcuuU..................................   14    1   c02
.........................................................cGgccacuaacgacacugcuuc..................................   1     1   c02
.........................................................cagccCcuaacgacacugcuuc..................................   1     1   c02
.........................................................cagccaAuaacgacacugcuuc..................................   2     1   c02
.........................................................cagccacuaacgCcacugcuuc..................................   3     1   c02
.........................................................cagccUcuaacgacacugcuuc..................................   1     1   c02
.........................................................caUccacuaacgacacugcuuc..................................   1     1   c02
.........................................................cCgccacuaacgacacugcuuc..................................   2     1   c02
.........................................................cagAcacuaacgacacugcuuc..................................   2     1   c02
.........................................................cagccacuaacgacacugcuuc..................................   376   0   c02
.........................................................cagccacuaacCacacugcuuc..................................   1     1   c02
.........................................................cagccacuaacgacacugcuuAu.................................   1     1   c02
.........................................................cagccacuaacgacacugcuucC.................................   3     1   c02
.........................................................cagccacuaacgCcacugcuucu.................................   4     1   c02
.........................................................cagccacuaacgacacugcuucA.................................   3     1   c02
.........................................................cagccacuaacgacacuUcuucu.................................   1     1   c02
.........................................................cCgccacuaacgacacugcuucu.................................   1     1   c02
.........................................................cagccacuaacgacacuCcuucu.................................   2     1   c02
.........................................................cagccacuaacgacacugcuucu.................................   144   0   c02
.........................................................cagccacuaacgacacugAuucu.................................   1     1   c02
.........................................................cagccacuaacgacacuAcuucu.................................   1     1   c02
.........................................................cagccacuaacgacacugcuCcu.................................   1     1   c02
.........................................................cagccacuaacgacacugcuucug................................   2     0   c02
.........................................................cagccacuaacgacacugcuucugc...............................   1     0   c02
..........................................................Cgccacuaacgacacugc.....................................   9     1   c02
..........................................................agccacuaacgacacugc.....................................   28    0   c02
..........................................................agccacuaacgCcacugc.....................................   1     1   c02
..........................................................Cgccacuaacgacacugcu....................................   9     1   c02
..........................................................agccacuaacgacacugcu....................................   20    0   c02
..........................................................Cgccacuaacgacacugcuu...................................   8     1   c02
..........................................................agccacuaacgacacugcuu...................................   26    0   c02
..........................................................agccacuaacgacacugAuuc..................................   1     1   c02
..........................................................agccacuaacgCcacugcuuc..................................   1     1   c02
..........................................................agccacuaacgacacugcuuc..................................   37    0   c02
..........................................................Ggccacuaacgacacugcuuc..................................   1     1   c02
..........................................................agccacuaacgacacugcuuU..................................   2     1   c02
..........................................................Cgccacuaacgacacugcuuc..................................   4     1   c02
..........................................................agccacuaacgacacugcuuAu.................................   1     1   c02
..........................................................agccacuaacgacacugAuucu.................................   3     1   c02
..........................................................agccacuaacgacacugcuucu.................................   755   0   c02
..........................................................Ugccacuaacgacacugcuucu.................................   1     1   c02
..........................................................agccacuaCcgacacugcuucu.................................   3     1   c02
..........................................................agccacuaacgGcacugcuucu.................................   1     1   c02
..........................................................agccCcuaacgacacugcuucu.................................   1     1   c02
..........................................................agccaAuaacgacacugcuucu.................................   1     1   c02
..........................................................agccacuaacgaAacugcuucu.................................   1     1   c02
..........................................................agccacuaacgacacugcuucC.................................   1     1   c02
..........................................................agccacuaacgacacuUcuucu.................................   1     1   c02
..........................................................aAccacuaacgacacugcuucu.................................   1     1   c02
```

Mature Star

```
gaaaauggguuagaccgcguuggcagugugguuagcugguuguuuauuauaugacaacagccacuaacgacacugcuucugcguacacccugauccuacauaucgacugacag
..........................................................agccacuaacgacacugcuucA.................................	4	1	c02
..........................................................agUcacuaacgacacugcuucu.................................	1	1	c02
..........................................................agccacuaacgacCcugcuucu.................................	1	1	c02
..........................................................agAcacuaacgacacugcuucu.................................	2	1	c02
..........................................................Cgccacuaacgacacugcuucu.................................	258	1	c02
..........................................................agccacuaacgacacCgcuucu.................................	1	1	c02
..........................................................agcAacuaacgacacugcuucu.................................	3	1	c02
..........................................................agccacuaacgacaAugcuucu.................................	2	1	c02
..........................................................aUccacuaacgacacugcuucu.................................	1	1	c02
..........................................................agccacuaacCacacugcuucu.................................	1	1	c02
..........................................................agccacuaacgaGacugcuucu.................................	1	1	c02
..........................................................agccacuaacgCcacugcuucu.................................	8	1	c02
..........................................................agccacAaacgacacugcuucu.................................	2	1	c02
..........................................................agccacuaaAgacacugcuucu.................................	1	1	c02
..........................................................agccacuaacgUcacugcuucu.................................	1	1	c02
..........................................................Ggccacuaacgacacugcuucu.................................	1	1	c02
..........................................................agccacuaacgacacugcuucuA................................	3	1	c02
..........................................................agccacuaacgacacugcuucuU................................	2	1	c02
...........................................................gccacuaacgacacugcuucu.................................	4	0	c02
............................................................ccacuaacgacacugcuucu.................................	1	0	c02
.............................................................cacuaacgacacugcuuc..................................	1	0	c02
.............................................................cacuaacgacacugcuucu.................................	3	0	c02
.............................................................cacuaacgacaAugcuucu.................................	1	1	c02
..............................................................acuaacgacacugcuucu.................................	4	0	c02
..............................................................Ccuaacgacacugcuucu.................................	1	1	c02
```

Provisional ID	: group10_15092
Score total	: 1902070.4
Score for star read(s)	: 3.9
Score for read counts	: 1902059.8
Score for mfe	: 2.1
Score for randfold	: 1.6
Score for cons. seed	: 3
Total read count	: 3730820
Mature read count	: 3729989
Loop read count	: 294
Star read count	: 537

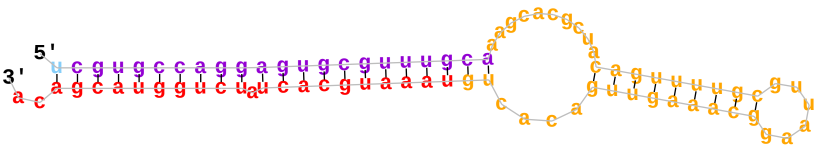

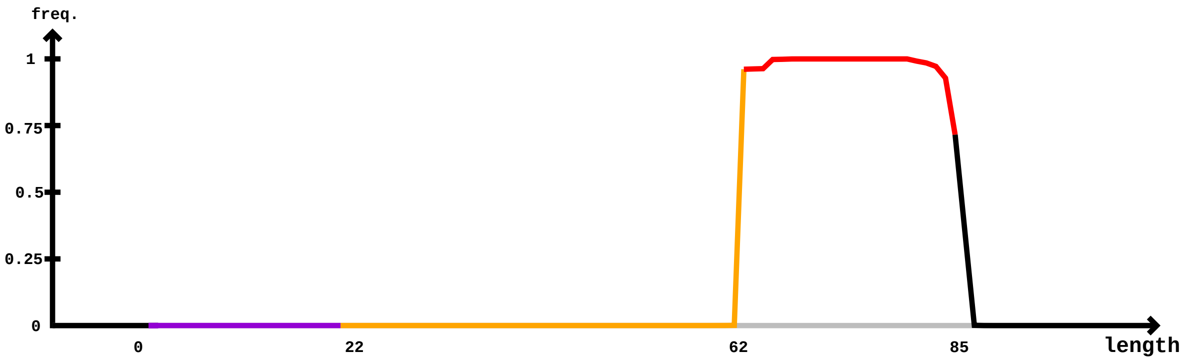

Star **Mature**

5'-	uccggaguucgugccaggagugcguuugcaaagcacgcuacaguuuugcguuaaggcaaaguugacacuguaaaugcacuaucugguacgacaccccggcgcguguagcgacg -3'	obs		
	uccggaguucgugccaggagugcguuugcaaagcacgcuacaguuuugcguuaaggcaaaguugacacuguaaaugcacuaucugguacgacaccccggcgcguguagcgacg	exp		
	.((((.((((((((((((((((((((((((..........(((((((((......)))))))))....)))))))))))).)))))))))).)).))))(((.....)))...	reads	mm	sample
	........ucgugccaggagugcguuugc..	1	0	c16
	........ucgugccaggagugcguuugca...	1	0	c16
	.........cgugccaggagugcguuu..	3	0	c16
	.........cgugccaggagugcguuug...	4	0	c16
	.........cgugccaggagugcguuugc..	5	0	c16
	.........cgugccaggagugcguuugca...	14	0	c16
	.........cgugccaggagugcguuugcU...	1	1	c16
	.........cgugccaggagugcguuugUa...	3	1	c16
	.........cgugccaggaguCcguuugca...	1	1	c16
	..............................aagcacgcuacaguuuugcguuaaggcaaag..	1	0	c16
	..............................aagcacgcuacaguuuugUguuaaggcaaag..	1	1	c16
	..............................aagcacgcuacaguuuugcguuaaggcaaaguugacac...	1	0	c16
	..............................aagcacgcuacaguuuugcguuaaggcaaaguugacacu..	1	0	c16
	..............................aagcacgcuacaguuuugcguuaaggcaaaguugacacug...	47	0	c16
	..............................aagcacgcuacaguuuugcguuaaggcaaaguugacCcug...	1	1	c16
	..............................Cagcacgcuacaguuuugcguuaaggcaaaguugacacug...	1	1	c16
	..............................aagcacgcuacaguuuugcguuaaggcaaaguuAacacug...	1	1	c16
	..............................aagcacUcuacaguuuugcguuaaggcaaaguugacacug...	1	1	c16
	.....................................cuacaguuuugcguuaaggcaaaguugacacug...	1	0	c16
	....acaguuuugcguuaaggcaaaguugacacug...	2	0	c16
	....guuuugcguuaaggcaaaguugacacug...	1	0	c16
	....uuuugcguuaaggcaaaguugacacug...	1	0	c16
	....uuugcguuaaggcaaaguugacacug...	3	0	c16
	....uugcguuaaggcaaaguugacacug...	1	0	c16
	....guuaagUcaaaguugacacug...	1	1	c16
	....guuaaggcaaaguugacacug...	1	0	c16
	....aGuguaaaugcacuaucugguacgaca....................	1	1	c16
	....cGguaaaugcacuaucugguacga......................	1	1	c16
	....cGguaaaugcacuaucugguacgaca....................	2	1	c16
	....Guguaaaugcacuaucugguacgaca....................	3	1	c16
	....uUuaaaugcacuaucugguacgaca....................	1	1	c16
	....uCuaaaugcacuaucugguacgaca....................	2	1	c16
	....Aguaaaugcacuaucugguacgaca....................	2	1	c16

Star **Mature**

uccggaguucgugccaggagugcguuugcaaagcacgcuacaguuuugcguuaaggcaaaguugacacuguaaaugcacuaucugguacgacaccccggcgcguguagcgacg

....Cguaaaugcacuaucugguacgaca....................	1	1	c16
....guaaaugcacuaucugguacgac.....................	1	0	c16
....Cuaaaugcacuaucugguacgac.....................	4	1	c16
....Cuaaaugcacuaucugguacgaca....................	10	1	c16
....Auaaaugcacuaucugguacgaca....................	1	1	c16
....uaaaugcCcuaucuggua.........................	2	1	c16
....uaaaugUacuaucuggua.........................	1	1	c16
....uaaaugcacuaucuggua.........................	152	0	c16
....uaaaugcacuaucuAgua.........................	1	1	c16
....uaaaugcacuaucugCua.........................	1	1	c16
....uaaaugcaAuaucuggua.........................	1	1	c16
....Gaaaugcacuaucuggua.........................	20	1	c16
....uaaaugcacuaucugguaU........................	1	1	c16
....uaaaugcacuaucugguac........................	177	0	c16
....Aaaaugcacuaucugguac........................	1	1	c16
....uaaaugcCcuaucugguac........................	1	1	c16
....Gaaaugcacuaucugguac........................	33	1	c16
....uaaaugcacuaucugguaA........................	1	1	c16
....uaaaugcacuaucugguaUg.......................	1	1	c16
....uaaaugcacuaucugguCcg.......................	1	1	c16
....Gaaaugcacuaucugguacg.......................	57	1	c16
....uaaaugcacuaucugguacg.......................	505	0	c16
....uaaaugcacuaucugguaAg.......................	3	1	c16
....uaaaugcCcuaucugguacg.......................	1	1	c16
....uaaaAgcacuaucugguacg.......................	1	1	c16
....uaaUugcacuaucugguacg.......................	1	1	c16
....Aaaaugcacuaucugguacg.......................	2	1	c16
....uaaauCcacuaucugguacg.......................	1	1	c16
....uaaCugcacuaucugguacg.......................	1	1	c16
....uaaaugcacuaucugguacU.......................	2	1	c16
....uaaaugcacuaucugUuacg.......................	1	1	c16
....uaaaugcacuaucuCguacg.......................	1	1	c16
....uaaaugcacuaucugguacCa......................	3	1	c16
....uaaaugcacuaucuAguacga......................	1	1	c16
....uaaaugcacuauAugguacga......................	2	1	c16
....uaaaugcacuaucugguacgC......................	6	1	c16
....uaaaugcacuaucugguacgG......................	3	1	c16
....uaaaugcCcuaucugguacga......................	2	1	c16
....uaaaugcacuCucugguacga......................	3	1	c16
....uaaaugcacuaucugUuacga......................	1	1	c16
....uaaUugcacuaucugguacga......................	1	1	c16
....uaaaCgcacuaucugguacga......................	1	1	c16
....uaaauUcacuaucugguacga......................	1	1	c16
....uaaaugcacuaucugguaUga......................	1	1	c16
....uaaaugcacuaucugguacga......................	2322	0	c16
....uaaCugcacuaucugguacga......................	2	1	c16
....uaaaugcaUuaucugguacga......................	1	1	c16
....uaaaugAacuaucugguacga......................	3	1	c16
....uaaaugcUcuaucugguacga......................	1	1	c16
....uaaaugcaAuaucugguacga......................	1	1	c16
....uaaaugcacuaucugguacAa......................	1	1	c16
....uaaaugcacuaucugguaGga......................	1	1	c16
....uaaaugcacuaucuCguacga......................	3	1	c16
....uaaaugcacuaucugguCcga......................	1	1	c16
....uaaaugGacuaucugguacga......................	1	1	c16
....uaaaugcacuaucugguacUa......................	1	1	c16
....uaaaugcacuaucugguacgU......................	6	1	c16
....Gaaaugcacuaucugguacga......................	289	1	c16
....uaaaugcacuaucugguaAga......................	5	1	c16
....uaaaugUacuaucugguacga......................	1	1	c16
....uaaaugcacuaucugCuacga......................	6	1	c16
....uaaauCcacuaucugguacga......................	8	1	c16
....uaaaAgcacuaucugguacga......................	2	1	c16
....uaaaugcacuaucuggAacga......................	1	1	c16
....uaaaugcacuaucAgguacga......................	4	1	c16
....Aaaaugcacuaucugguacga......................	17	1	c16
....uaaaugcacuaucugguGcga......................	1	1	c16
....uaaaugcacuaucuggCacga......................	2	1	c16
....uaaaugcacuaucugguaUgac.....................	3	1	c16
....uaaaugcacuauUugguacgac.....................	1	1	c16

Star Mature

```
uccggaguucgugccaggagugcguuugcaaagcacgcuacaguuuugcguuaaggcaaaguugacacuguaaaugcacuaucugguacgacaccccggcgcguguagcgacg
......................................................................uaaaugcacuaucugUuacgac.....................	3	1	c16
......................................................................uaaaugcacuaucugguacAac.....................	3	1	c16
......................................................................uaaaugcacuaAcugguacgac.....................	2	1	c16
......................................................................uaaaAgcacuaucugguacgac.....................	8	1	c16
......................................................................uaaaugcacuaucugguUcgac.....................	1	1	c16
......................................................................uaaaugcaUuaucugguacgac.....................	6	1	c16
......................................................................uaaaugcacuaucugguacgaA.....................	14	1	c16
......................................................................uaaaugcacuGucugguacgac.....................	1	1	c16
......................................................................uaaaugcacuaucugCuacgac.....................	12	1	c16
......................................................................uaaaugcacuauGugguacgac.....................	2	1	c16
......................................................................uaaaugcaGuaucugguacgac.....................	2	1	c16
......................................................................uaaaugcacuUucugguacgac.....................	1	1	c16
......................................................................uaaaugcacuaucuAguacgac.....................	1	1	c16
......................................................................uaaaugcGcuaucugguacgac.....................	1	1	c16
......................................................................uaaauUcacuaucugguacgac.....................	5	1	c16
......................................................................uaaaugAacuaucugguacgac.....................	16	1	c16
......................................................................uaaaugcacuaucugguacgac.....................	7067	0	c16
......................................................................uaUaugcacuaucugguacgac.....................	2	1	c16
......................................................................uaaaugcacuauAugguacgac.....................	5	1	c16
......................................................................uaaaugcacuaucugguacgGc.....................	2	1	c16
......................................................................uaaaugcacuaucugguacgUc.....................	3	1	c16
......................................................................uaaaugcacuaucCgguacgac.....................	5	1	c16
......................................................................uaaaugcacuaucuggAacgac.....................	2	1	c16
......................................................................uaaaugcacuaucugguaGgac.....................	1	1	c16
......................................................................uaaaugcacuaucugguGcgac.....................	2	1	c16
......................................................................uaaaugcaAuaucugguacgac.....................	1	1	c16
......................................................................uaaaugcUcuaucugguacgac.....................	1	1	c16
......................................................................Aaaaugcacuaucugguacgac.....................	35	1	c16
......................................................................uaaauAcacuaucugguacgac.....................	1	1	c16
......................................................................uaaaugcacuaucuggCacgac.....................	4	1	c16
......................................................................Caaaugcacuaucugguacgac.....................	5	1	c16
......................................................................uaaaugcacuaucugguacgaG.....................	8	1	c16
......................................................................uaaaugcacGaucugguacgac.....................	1	1	c16
......................................................................uaaaugcacuaucugguacgCc.....................	27	1	c16
......................................................................Gaaaugcacuaucugguacgac.....................	786	1	c16
......................................................................uaaaugcCcuaucugguacgac.....................	21	1	c16
......................................................................uaaaCgcacuaucugguacgac.....................	3	1	c16
......................................................................uaaaugcacuaucugguacCac.....................	5	1	c16
......................................................................uaaaugcacuaucuCguacgac.....................	14	1	c16
......................................................................uaaaugcacuaucAgguacgac.....................	8	1	c16
......................................................................uaaaugcacuaucugguacUac.....................	9	1	c16
......................................................................uaaaugUacuaucugguacgac.....................	2	1	c16
......................................................................uaaaugcacAaucugguacgac.....................	1	1	c16
......................................................................uaaaugcacuaucGgguacgac.....................	3	1	c16
......................................................................uaCaugcacuaucugguacgac.....................	1	1	c16
......................................................................uaaaugcacuaucugguacgaU.....................	5	1	c16
......................................................................uaaaugcacuaucugAuacgac.....................	2	1	c16
......................................................................uaaauCcacuaucugguacgac.....................	8	1	c16
......................................................................uaaaugcacuaucugguaAgac.....................	11	1	c16
......................................................................uaaCugcacuaucugguacgac.....................	3	1	c16
......................................................................uaaaGgcacuaucugguacgac.....................	2	1	c16
......................................................................uaaaugcacuaucugguCcgac.....................	4	1	c16
......................................................................uaaaugUacuaucugguacgaca....................	8	1	c16
......................................................................uaUaugcacuaucugguacgaca....................	3	1	c16
......................................................................uaaaugcacuaucugguCcgaca....................	19	1	c16
......................................................................uaaaugcacuaucugguUcgaca....................	5	1	c16
......................................................................uaaaugcaUuaucugguacgaca....................	12	1	c16
......................................................................uaaaugcacuaGcugguacgaca....................	1	1	c16
......................................................................uaaaugcacuaucugguacgacU....................	957	1	c16
......................................................................uaaaugcUcuaucugguacgaca....................	11	1	c16
......................................................................uaaaugcacuaucAgguacgaca....................	14	1	c16
......................................................................uaaaugcacuaCcugguacgaca....................	2	1	c16
......................................................................uaaaugcacuaucugCuacgaca....................	56	1	c16
......................................................................uaaaugcacuaucugguaUgaca....................	14	1	c16
......................................................................uaaauUcacuaucugguacgaca....................	20	1	c16
......................................................................uaaaugcacuaucugguacgacC....................	247	1	c16
......................................................................uaaUugcacuaucugguacgaca....................	2	1	c16
......................................................................Caaaugcacuaucugguacgaca....................	18	1	c16
......................................................................uaaaugcacuaucuUguacgaca....................	14	1	c16
......................................................................uaaaAgcacuaucugguacgaca....................	21	1	c16
```

```
         Star                                                        Mature
uccggaguucgugccaggagugcguuugcaaagcacgcuacaguuuugcguuaaggcaaaguugacacuguaaaugcacuaucugguacgacaccccggcgcguguagcgacg
......................................................................uaaaugcacuaucuggAacgaca....................    14      1    c16
......................................................................uCaaugcacuaucugguacgaca....................    2       1    c16
......................................................................uaaaugcacuUucugguacgaca....................    3       1    c16
......................................................................uaaaugcGcuaucugguacgaca....................    8       1    c16
......................................................................uaaaugcacuaucuggCacgaca....................    10      1    c16
......................................................................uaaaugcacuaucugguGcgaca....................    5       1    c16
......................................................................uaaaugcacuaucuggGacgaca....................    4       1    c16
......................................................................uaaCugcacuaucugguacgaca....................    14      1    c16
......................................................................uGaaugcacuaucugguacgaca....................    7       1    c16
......................................................................uaaaugcacuaucugUuacgaca....................    18      1    c16
......................................................................uaaaugcacuaucugguacgaGa....................    5       1    c16
......................................................................uaaaugcacuaucuCguacgaca....................    41      1    c16
......................................................................uaaaugcacuaucCgguacgaca....................    7       1    c16
......................................................................uaaaugcacuaucugguacgaUa....................    6       1    c16
......................................................................uaaaugcacuaucGgguacgaca....................    6       1    c16
......................................................................uaaaugcacuaucugguacCaca....................    25      1    c16
......................................................................uaaaugcacAaucugguacgaca....................    8       1    c16
......................................................................uaaaugcacuaucugguaGgaca....................    4       1    c16
......................................................................uaaaugcacuCucugguacgaca....................    5       1    c16
......................................................................uaaaugcacuaAcugguacgaca....................    7       1    c16
......................................................................uaaaugAacuaucugguacgaca....................    47      1    c16
......................................................................uaaaugcaGuaucugguacgaca....................    6       1    c16
......................................................................uaaaugcacuaucugguacUaca....................    27      1    c16
......................................................................uaCaugcacuaucugguacgaca....................    2       1    c16
......................................................................uaaaugcacuaucuAguacgaca....................    4       1    c16
......................................................................uaaaugcacuaucugguacAaca....................    7       1    c16
......................................................................uaaaugcacuaucugguacgUca....................    5       1    c16
......................................................................uaaaugGacuaucugguacgaca....................    6       1    c16
......................................................................uaaGugcacuaucugguacgaca....................    3       1    c16
......................................................................uaaaugcacuaucugguacgGca....................    6       1    c16
......................................................................uaaaugcCcuaucugguacgaca....................    57      1    c16
......................................................................uaGaugcacuaucugguacgaca....................    3       1    c16
......................................................................uaaaugcacuauUugguacgaca....................    4       1    c16
......................................................................uaaaugcacGaucugguacgaca....................    8       1    c16
......................................................................uaaaugcacuaucugguacgacG....................    28      1    c16
......................................................................uaaaugcacuauAugguacgaca....................    22      1    c16
......................................................................uaaaugcacuaucugguacgCca....................    106     1    c16
......................................................................Aaaaugcacuaucugguacgaca....................    169     1    c16
......................................................................uaaaCgcacuaucugguacgaca....................    6       1    c16
......................................................................uaaaugcacuaucugguacgaAa....................    38      1    c16
......................................................................uaaaugcaAuaucugguacgaca....................    32      1    c16
......................................................................uaaaugcacuaucugAuacgaca....................    4       1    c16
......................................................................Gaaaugcacuaucugguacgaca....................    3156    1    c16
......................................................................uaaauCcacuaucugguacgaca....................    26      1    c16
......................................................................uaaaugcacuaucugguaAgaca....................    54      1    c16
......................................................................uUaaugcacuaucugguacgaca....................    5       1    c16
......................................................................uaaauAcacuaucugguacgaca....................    2       1    c16
......................................................................uaaaugcacuGucugguacgaca....................    5       1    c16
......................................................................uaaaugcacuaucugguacgaca....................    27604   0    c16
......................................................................uaaaugcacuaucugguacgacGc...................    1       1    c16
......................................................................Aaaaugcacuaucugguacgacac...................    2       1    c16
......................................................................uaaaugAacuaucugguacgacac...................    1       1    c16
......................................................................uaaaugcacuaucugguacgacUc...................    4       1    c16
......................................................................uaaaugcacuaucugguacgacCc...................    1       1    c16
......................................................................uaaaugcacuaucugguacAacac...................    1       1    c16
......................................................................uaaaugcacuaucugguacgacac...................    314     0    c16
......................................................................uaaaugcacuaucugguacgCcac...................    3       1    c16
......................................................................uaaaugcaAuaucugguacgacac...................    1       1    c16
......................................................................uaaaugcacuauUugguacgacac...................    1       1    c16
......................................................................uaaaugcacuaucugguacgacaG...................    37      1    c16
......................................................................uaaaugcacuaucugguaUgacac...................    1       1    c16
......................................................................Gaaaugcacuaucugguacgacac...................    36      1    c16
......................................................................uaaauCcacuaucugguacgacac...................    1       1    c16
......................................................................uaaaugcacuaucugguaAgacac...................    1       1    c16
......................................................................uaaaugcacuaucugguacgUcac...................    1       1    c16
......................................................................uaaaugcacuaucugguacCacac...................    2       1    c16
......................................................................uaaaugcacuaucugguacgacaU...................    505     1    c16
......................................................................uaaaugcacuaucugguacgacaA...................    498     1    c16
......................................................................uGaaugcacuaucugguacgacac...................    1       1    c16
......................................................................uaaaugcacuaucugguacgacacU..................    44      1    c16
```

Star Mature

uccggaguucgugccaggagugcguuugcaaagcacgcuacaguuuugcguuaaggcaaaguugacacuguaaaugcacuaucugguacgacaccccggcgcguguagcgacg			
....uaaaugcacuaucugguacgacaAc..................	1	1	c16
....Gaaaugcacuaucugguacgacacc..................	2	1	c16
....uaaaugcacuaucugguacgacacc..................	3	0	c16
....uaaaugcacuaucugguacgacacA..................	3	1	c16
....uaaaugcacuaucugguacgacaUc..................	1	1	c16
....uaaaugcacuaucugguacgacaGc..................	1	1	c16
....uaaaugcacuaucugguacgacacccc................	1	0	c16
....aaaugcacuaucugguac........................	2	0	c16
....aaaGgcacuaucugguacg.......................	1	1	c16
....Caaugcacuaucugguacga......................	1	1	c16
....aaaugcacuaucugguacga......................	4	0	c16
....aUaugcacuaucugguacgac.....................	1	1	c16
....Uaaugcacuaucugguacgac.....................	1	1	c16
....aaaugcacuaucugguacgaA.....................	1	1	c16
....aaaugcacuaucugguacgac.....................	25	0	c16
....Caaugcacuaucugguacgac.....................	1	1	c16
....Uaaugcacuaucugguacgaca....................	6	1	c16
....aaaugcacuaucugguacgaca....................	21	0	c16
....aaaugcacuaucugguacgacU....................	4	1	c16
....aaaugcacuaucugguacgacaU...................	1	1	c16
....Caaugcacuaucugguacgacac...................	1	1	c16
....aaaugcacuaucugguacgacac...................	1	0	c16
....aaugcacuaucugguacga......................	1	0	c16
....aUugcacuaucugguacgac.....................	1	1	c16
....aaugcacuaucugguacgac.....................	4	0	c16
....Caugcacuaucugguacgaca....................	6	1	c16
....aaugcacuaucugguacgacU....................	1	1	c16
....aaugcCcuaucugguacgaca....................	1	1	c16
....aaugcacuaucugguacgaca....................	30	0	c16
....aaugcacuaucugguacgacac...................	1	0	c16
....aaugcacuaucugguacgacaA...................	1	1	c16
....aaugcacuaucugguacgacacA..................	1	1	c16
....augcacuaucugguacga......................	63	0	c16
....augcacuaucuCguacga......................	1	1	c16
....auUcacuaucugguacga......................	1	1	c16
....Cugcacuaucugguacga......................	7	1	c16
....augcacuaucuCguacgac.....................	1	1	c16
....augcacuaucugCuacgac.....................	1	1	c16
....Cugcacuaucugguacgac.....................	17	1	c16
....augAacuaucugguacgac.....................	1	1	c16
....augcacuaucugguacgac.....................	114	0	c16
....augcacuaucugguacgacC....................	6	1	c16
....augcacuaucugguacgCca....................	5	1	c16
....Cugcacuaucugguacgaca....................	113	1	c16
....augcacuaAcugguacgaca....................	1	1	c16
....augcaAuaucugguacgaca....................	1	1	c16
....augcacuaucuCguacgaca....................	1	1	c16
....augGacuaucugguacgaca....................	1	1	c16
....augcacuaucugguacCaca....................	1	1	c16
....augcacuaucugguGcgaca....................	1	1	c16
....augcacuaucugguacgacU....................	16	1	c16
....augAacuaucugguacgaca....................	1	1	c16
....augcGcuaucugguacgaca....................	1	1	c16
....augcacuaucuUguacgaca....................	2	1	c16
....augcacuaucugguCcgaca....................	1	1	c16
....augcacuaucugguacgaca....................	447	0	c16
....augUacuaucugguacgaca....................	1	1	c16
....augcacuaucugguaAgaca....................	1	1	c16
....augcacuaucugguacgCcac...................	1	1	c16
....augcacuaucugguacgacaG...................	1	1	c16
....augcacuaucugguacgacaA...................	12	1	c16
....augcacuaucugguacgacac...................	5	0	c16
....Cugcacuaucugguacgacac...................	1	1	c16
....augcacuaucugguacgacaU...................	14	1	c16
....augcacuaucugguacgacacU..................	1	1	c16
....ugcacuaucugguacgac.....................	6	0	c16
....Ggcacuaucugguacgac.....................	1	1	c16
....ugAacuaucugguacgaca....................	1	1	c16
....ugcacuaucugguacgacU....................	2	1	c16
....ugcacuaucugguacgaca....................	19	0	c16

```
        Star                                                      Mature
uccggaguucgugccaggagugcguuugcaaagcacgcuacaguuuugcguuaaggcaaaguugacacuguaaaugcacuaucugguacgacaccccggcgcguguagcgacg
..........................................................................Cgcacuaucugguacgaca....................    1     1     c16
..........................................................................Ggcacuaucugguacgaca....................    2     1     c16
...........................................................................gcacuaucugguacgacU....................    3     1     c16
...........................................................................gcacuaucugguacgaca....................    35    0     c16
...........................................................................gcaUuaucugguacgaca....................    1     1     c16
...........................................................................gcacuaucugguacgacC....................    1     1     c16
...........................................................................gcCcuaucugguacgaca....................    1     1     c16
...........................................................................gcacuaucugguacgacaU...................    1     1     c16
...........................................................................gcacuaucugguacgacaA...................    1     1     c16
............................................................................cacuaucugguacgacaU...................    1     1     c16
............................................................................cacuaucugguacgacaA...................    1     1     c16

........ucgugccaggagugcguuugc....................................................................................    1     0     c04
.........cgugccaggagugcguuugca...................................................................................    1     0     c04
..............................aagcacgcuacaguuuugcguuaaggcaaaguugacacug...........................................    1     0     c04
..................................................................aGuguaaaugcacuaucugguacgaca....................    1     1     c04
...................................................................cuAuaaaugcacuaucugguacgac.....................    1     1     c04
....................................................................uguaaaugcacuaucuggu..........................    1     0     c04
....................................................................Aguaaaugcacuaucugguacga......................    1     1     c04
....................................................................uguaaaGgcacuaucugguacgaca....................    1     1     c04
.....................................................................Cuaaaugcacuaucugguacga......................    1     1     c04
.....................................................................Cuaaaugcacuaucugguacgac.....................    1     1     c04
.....................................................................Uuaaaugcacuaucugguacgaca....................    1     1     c04
.....................................................................Auaaaugcacuaucugguacgaca....................    1     1     c04
......................................................................uaaaugcacuaucuggua.........................    79    0     c04
......................................................................Gaaaugcacuaucuggua.........................    4     1     c04
......................................................................uaaaugcacuaucCggua.........................    1     1     c04
......................................................................uaaauAcacuaucuggua.........................    1     1     c04
......................................................................uaaaugcacuaucugguG.........................    1     1     c04
......................................................................uaaaugcacuaucugUuac........................    1     1     c04
......................................................................uaaaugcacuaucugguCc........................    1     1     c04
......................................................................uaaaugcacuauUugguac........................    1     1     c04
......................................................................uaaaugcacuaucugguaA........................    1     1     c04
......................................................................Gaaaugcacuaucugguac........................    2     1     c04
......................................................................uaaaugcacuaucuCguac........................    2     1     c04
......................................................................uaaaugcacuaucugguac........................    55    0     c04
......................................................................Gaaaugcacuaucugguacg.......................    17    1     c04
......................................................................uaaaugcacuaucugguacg.......................    104   0     c04
......................................................................uaaaugcacuaucugguacUa......................    2     1     c04
......................................................................uaaaugcaAuaucugguacga......................    1     1     c04
......................................................................uaaaugcacuaucugguGcga......................    1     1     c04
......................................................................uaaaugcacuaucugguacga......................    320   0     c04
......................................................................uaaaugcacuaGcugguacga......................    1     1     c04
......................................................................uaaaugcaUuaucugguacga......................    1     1     c04
......................................................................uaaaugcacuaucugCuacga......................    1     1     c04
......................................................................Gaaaugcacuaucugguacga......................    27    1     c04
......................................................................Aaaaugcacuaucugguacga......................    2     1     c04
......................................................................uaaaugcacuaucugguaAga......................    1     1     c04
......................................................................uaaauAcacuaucugguacga......................    1     1     c04
......................................................................uaaauUcacuaucugguacgac.....................    1     1     c04
......................................................................uaaaugcacuaGcugguacgac.....................    1     1     c04
......................................................................uaaaugcacuaucugguacgaG.....................    2     1     c04
......................................................................uaaaugcacuaucCgguacgac.....................    1     1     c04
......................................................................uaaaugcacuGucugguacgac.....................    1     1     c04
......................................................................uaaaugcacuaucugguacgCc.....................    6     1     c04
......................................................................uaaaugcacuaucuCguacgac.....................    3     1     c04
......................................................................uaaaugcacuaucuUguacgac.....................    3     1     c04
......................................................................uaaaugcacuaucugguacgac.....................    1271  0     c04
......................................................................uaaaugcCcuaucugguacgac.....................    3     1     c04
......................................................................uaaaugcacuaucAgguacgac.....................    2     1     c04
......................................................................Aaaaugcacuaucugguacgac.....................    9     1     c04
......................................................................uaaaugcacuauAugguacgac.....................    1     1     c04
......................................................................uaGaugcacuaucugguacgac.....................    1     1     c04
......................................................................uaaaugcacuaucugguaAgac.....................    1     1     c04
......................................................................uaaauCcacuaucugguacgac.....................    1     1     c04
......................................................................uaaaugcacuaucugguCcgac.....................    1     1     c04
......................................................................uaaaugcacuaucugguGcgac.....................    1     1     c04
......................................................................uaaaugcacuaAcugguacgac.....................    2     1     c04
......................................................................uaaaugcacuaucugguacCac.....................    1     1     c04
......................................................................uaaaAgcacuaucugguacgac.....................    2     1     c04
```

Star **Mature**

```
uccggaguucgugccaggagugcguuugcaaagcacgcuacaguuuugcguuaaggcaaaguugacacuguaaaugcacuaucugguacgacaccccggcgcguguagcgacg
......................................................................Gaaaugcacuaucugguacgac.....................   107    1   c04
......................................................................uaaaugcacuaucugguacUac.....................   1      1   c04
......................................................................uaaaugcacuaucugCuacgac.....................   2      1   c04
......................................................................uaaaugcacuaucugguacgUc.....................   1      1   c04
......................................................................uaaaugcacuaucugguacgaA.....................   3      1   c04
......................................................................uaaaugcacuaucuggCacgac.....................   1      1   c04
......................................................................uaaaugcacuaucugguacgaU.....................   3      1   c04
......................................................................uaaaugcacuaucugUuacgac.....................   1      1   c04
......................................................................uaaaugAacuaucugguacgac.....................   1      1   c04
......................................................................uaaaugcacuaucugguaAgaca....................   7      1   c04
......................................................................uaaaugcacuaucugguacgaAa....................   4      1   c04
......................................................................uaaaugcacuaucugCuacgaca....................   11     1   c04
......................................................................uaaaugcacGaucugguacgaca....................   1      1   c04
......................................................................uaaaCgcacuaucugguacgaca....................   1      1   c04
......................................................................uaaaugcacuaucugguacCaca....................   9      1   c04
......................................................................uaaaugcacuaucugguGcgaca....................   4      1   c04
......................................................................Aaaaugcacuaucugguacgaca....................   21     1   c04
......................................................................uaaaugcacuaucuggCacgaca....................   2      1   c04
......................................................................uaaaugcacuaucuUguacgaca....................   2      1   c04
......................................................................uaaaugAacuaucugguacgaca....................   2      1   c04
......................................................................uaaaugcacuaucuggAacgaca....................   2      1   c04
......................................................................uaaaugcaGuaucugguacgaca....................   1      1   c04
......................................................................uaaaugcacuaucugAuacgaca....................   2      1   c04
......................................................................uaaaugcacuaucugUuacgaca....................   2      1   c04
......................................................................uaaaugcacuaucugguacUaca....................   1      1   c04
......................................................................uaaaugcacuaucugguacgacC....................   55     1   c04
......................................................................uaaaugcacuaucugguacgaca....................   3403   0   c04
......................................................................uaaaugcaAuaucugguacgaca....................   3      1   c04
......................................................................uaaaugcacuaGcugguacgaca....................   1      1   c04
......................................................................uaaaugcacuaucAgguacgaca....................   1      1   c04
......................................................................uaaauUcacuaucugguacgaca....................   4      1   c04
......................................................................uaaaugcCcuaucugguacgaca....................   2      1   c04
......................................................................uaaaugcacuaucugguacgacG....................   5      1   c04
......................................................................uaaaugcacuaucugguacgaUa....................   1      1   c04
......................................................................uaaaugcacuaucugguacgacU....................   114    1   c04
......................................................................uaaaugcacuaucugguacAaca....................   1      1   c04
......................................................................uaaaugcacuaucugguacgUca....................   1      1   c04
......................................................................uaaaugcacuaucuCguacgaca....................   3      1   c04
......................................................................uaaaugcacuaucugguacgGca....................   1      1   c04
......................................................................uaaaAgcacuaucugguacgaca....................   3      1   c04
......................................................................uaaaugcacuaucuAguacgaca....................   1      1   c04
......................................................................uaaaugcacuaucugguacgCca....................   10     1   c04
......................................................................uaaauCcacuaucugguacgaca....................   2      1   c04
......................................................................uaaaugcacuaucugguaUgaca....................   1      1   c04
......................................................................Gaaaugcacuaucugguacgaca....................   267    1   c04
......................................................................uaaaugcacuauAugguacgaca....................   3      1   c04
......................................................................uaaauAcacuaucugguacgaca....................   1      1   c04
......................................................................uaaaugUacuaucugguacgaca....................   1      1   c04
......................................................................uaaaugcacAaucugguacgaca....................   1      1   c04
......................................................................uaaaugcCcuaucugguacgacac...................   1      1   c04
......................................................................uaaaugcacuaucugguacgacaA...................   68     1   c04
......................................................................Gaaaugcacuaucugguacgacac...................   1      1   c04
......................................................................uaaaugcacuaucugguacgacCc...................   1      1   c04
......................................................................uaaaugcacuaucugguacgacac...................   43     0   c04
......................................................................uaaaugcacuaucugguacgacUc...................   1      1   c04
......................................................................uaaaugcGcuaucugguacgacac...................   1      1   c04
......................................................................uaaaugcacuaucugguacgacaU...................   65     1   c04
......................................................................uaaaugcacuaucugguacgacaG...................   10     1   c04
......................................................................uaaaugcacuaucugguacgacaGc..................   1      1   c04
......................................................................uaaaugcacuaucugguacgacacU..................   2      1   c04
......................................................................uaaaugcacuaucugguacgacaAc..................   1      1   c04
......................................................................uaaaugcacuaucugguacgacacG..................   1      1   c04
......................................................................uaaaugcacuaucugguacgacacA..................   1      1   c04
......................................................................uaaaugcacuaucugguacgacaGcc.................   1      1   c04
.......................................................................Uaaugcacuaucugguacg.......................   1      1   c04
.......................................................................aaaugcacuaucugguacg.......................   2      0   c04
.......................................................................aUaugcacuaucugguacgac.....................   1      1   c04
.......................................................................aaaugcacuaucugguacgac.....................   4      0   c04
.......................................................................aaaugcacuaucugguacgacU....................   1      1   c04
.......................................................................aaaugcacuaucugguacgaca....................   2      0   c04
```

Star **Mature**

```
uccggaguucgugccaggagugcguuugcaaagcacgcuacaguuuugcguuaaggcaaaguugacacuguaaaugcacuaucugguacgacaccccggcgcguguagcgacg
........................................................................aaugcacuaucugguacg.......................   1    0    c04
........................................................................aaugcacuaucugguacgac.....................   2    0    c04
........................................................................Uaugcacuaucugguacgac.....................   1    1    c04
........................................................................aGugcacuaucugguacgac.....................   1    1    c04
........................................................................Gaugcacuaucugguacgaca....................   2    1    c04
........................................................................aaugcacuaucugguacgaca....................   2    0    c04
........................................................................aaGgcacuaucugguacgaca....................   1    1    c04
.........................................................................augcacuaucugguaUga......................   1    1    c04
.........................................................................augcacuaucugguacga......................   15   0    c04
.........................................................................Cugcacuaucugguacgac.....................   1    1    c04
.........................................................................augcacuaucugguacgac.....................   31   0    c04
.........................................................................augcacuauAugguacgac.....................   1    1    c04
.........................................................................augcacuauGugguacgac.....................   1    1    c04
.........................................................................augcacuaucugguGcgac.....................   1    1    c04
.........................................................................augcacuaucugguacgGca....................   1    1    c04
.........................................................................augcacuaucugguacUaca....................   1    1    c04
.........................................................................augcacuaucugguacgaca....................   97   0    c04
.........................................................................augcacuaucugguaAgaca....................   1    1    c04
.........................................................................Cugcacuaucugguacgaca....................   8    1    c04
.........................................................................augcacuaucugguacgacU....................   1    1    c04
.........................................................................augcacuaucugguacgacac...................   1    0    c04
.........................................................................augcacuaucugguacgacaA...................   4    1    c04
..........................................................................Ggcacuaucugguacgac.....................   2    1    c04
..........................................................................ugcacuaucugguacgac.....................   1    0    c04
..........................................................................Ggcacuaucugguacgaca....................   1    1    c04
...........................................................................gcacuaucugguacgaca....................   10   0    c04

........ucgugccaggagugcguu.......................................................................................   4    0    c09
........ucgugccaggagugcguuu......................................................................................   14   0    c09
........ucguCccaggagugcguuu......................................................................................   1    1    c09
........ucgugccaggaAugcguuu......................................................................................   1    1    c09
........ucgugccaggagugcguuug.....................................................................................   6    0    c09
........ucgugccaggCgugcguuugc....................................................................................   1    1    c09
........ucgugccaggagugcguuugA....................................................................................   1    1    c09
........ucgugccaggagugcgGuugc....................................................................................   1    1    c09
........ucgugccaggaCugcguuugc....................................................................................   1    1    c09
........Gcgugccaggagugcguuugc....................................................................................   7    1    c09
........ucgugccaggagugcguuugc....................................................................................   67   0    c09
........ucgugccaggagugcguuugcC...................................................................................   1    1    c09
........ucgugccaggagugcguuugca...................................................................................   25   0    c09
........ucgugUcaggagugcguuugca...................................................................................   1    1    c09
........Gcgugccaggagugcguuugca...................................................................................   1    1    c09
........ucgugccaggagugcguuugcU...................................................................................   3    1    c09
........Gcgugccaggagugcguuugcaa..................................................................................   1    1    c09
........ucgugccaggagugcguuugcaU..................................................................................   1    1    c09
........ucgugccaggagugcguuugcaC..................................................................................   1    1    c09
.........cgugccaggagugcguuu......................................................................................   40   0    c09
.........cgugccaggagugcguuug.....................................................................................   10   0    c09
.........cgugccaggagugcguuuA.....................................................................................   1    1    c09
.........cgugcGaggagugcguuugc....................................................................................   1    1    c09
.........Agugccaggagugcguuugc....................................................................................   1    1    c09
.........cgugccaggagugcguuugc....................................................................................   53   0    c09
.........cgugccaggagugcguuuAc....................................................................................   1    1    c09
.........cgugccaggagugcguuuUc....................................................................................   1    1    c09
.........cgugccaggagugUguuugca...................................................................................   2    1    c09
.........cgugccaggagugcguuugca...................................................................................   108  0    c09
.........cgugccaggagugcguuugcU...................................................................................   2    1    c09
.........cgugccaggagugcguuugcaC..................................................................................   1    1    c09
.........cgugccaggagugcguuugcUa..................................................................................   1    1    c09
.........cgugccaggagugcguuugcaaa.................................................................................   2    0    c09
.........cgugccaggagugcguuugcaaagc...............................................................................   1    0    c09
.........cgugccaggagugcguuugcaaagca..............................................................................   1    0    c09
.........cgugccaggagugcguuugcaaagcac.............................................................................   3    0    c09
.........cgugccaggagugcguuugcaaagcacgcu..........................................................................   3    0    c09
.........cgugccaggagugcguuugcaaagcacgcuacaguu....................................................................   1    0    c09
.........cgugccaggagugcguuugcaaagcacgcuacaguuu...................................................................   1    0    c09
..........gugccaggagugcguuugc....................................................................................   1    0    c09
..........gugccaggagugcguuugca...................................................................................   1    0    c09
..............caggagugcguuugcaaagc...............................................................................   1    0    c09
..............................aagcacgcuacaguuuugcguuaagg.........................................................   1    0    c09
```

Star Mature

```
uccggaguucgugccaggagugcguuugcaaagcacgcuacaguuuugcguuaaggcaaaguugacacuguaaaugcacuaucugguacgacaccccggcgcguguagcgacg
..............................aagcacgcuacaguuuugcguuaaggcaaag....................................................	1	0	c09
..............................aagcacgcuacaguuuugcguuaaggcaaagu...................................................	1	0	c09
..............................aagcacgcuacaguuuugcguuaaggcaaaguugac...............................................	1	0	c09
..............................aagcacgcuacaguuuugcguuaaggcaaaguugacacu............................................	5	0	c09
..............................aagcacgcuacaguuuugcguuaaggcaaaguugCcacu............................................	1	1	c09
..............................aagcacgcuacaguuuugcguuaaggUaaaguugacacug...........................................	1	1	c09
..............................aagcacgcuacaguuuugcguuaaggcaaaguuUacacug...........................................	1	1	c09
..............................aagcacgcuacaguuuugcguuaaggcaaaguugacacug...........................................	109	0	c09
..............................aagcacgcuacaguuuugcguuaaggcaaaguGgacacug...........................................	1	1	c09
..............................aagcacgcuacaguuuugcguuaaggcaaaguugCcacug...........................................	1	1	c09
..............................Cagcacgcuacaguuuugcguuaaggcaaaguugacacug...........................................	4	1	c09
..................................acgcuacaguuuugcguuaaggcaaaguugacacug...........................................	2	0	c09
......................................Gacaguuuugcguuaaggcaaaguugacacug...........................................	1	1	c09
.......................................acaguuuugcguuaaggcaaaguugacacug...........................................	2	0	c09
.......................................acaguuuugcguuaaggcaaaguugacacugu..........................................	1	0	c09
........................................caguuuugcguuaaggcaaaguugac...............................................	1	0	c09
.........................................aguuuugcguuaaggcaaag....................................................	1	0	c09
.........................................aguuuugcguuaaggcaaaguugacacug...........................................	1	0	c09
..........................................guuuugcguuaaggcaaaguugacacug...........................................	6	0	c09
..........................................guuuugcguuaaggcaaaguugacacugua.........................................	1	0	c09
...........................................uuuugcguuaaggcaaaguugac...............................................	1	0	c09
...........................................uuuugcguuaaggcaaaguugacacug...........................................	7	0	c09
............................................uuugcguuaaggcaaaguugacacu............................................	1	0	c09
............................................uuugcguuaaggcaaaguugacacug...........................................	4	0	c09
.............................................uugcguuaaggcaaaguugaca..............................................	1	0	c09
.............................................uugcguuaaggcaaaguugacacug...........................................	1	0	c09
..............................................ugcguuaaggcaaaguugacacu............................................	1	0	c09
..............................................ugcguuaaggcaaaguugacacug...........................................	1	0	c09
...............................................gcguuaaggcaaaguugaca..............................................	2	0	c09
................................................cguuaaggcaaaguugacacug...........................................	2	0	c09
.................................................guuaaggcaaaguugacacug...........................................	4	0	c09
.................................................guuaaggcaaaguugacacugA..........................................	1	1	c09
..................................................uuaaggcaaaguugacacu............................................	1	0	c09
..................................................uuaaggcaaaguugacacug...........................................	1	0	c09
...................................................uaaggcaaaguugacacuguaaaugcacuaucugg...........................	1	0	c09
....................................................aaggcaaaguugacacug...........................................	2	0	c09
......................................................Ugcaaaguugacacuguaaau......................................	1	1	c09
.......................................................gAcaaaguugacacuguaaaugcacuaucugguacgac.....................	1	1	c09
.......................................................gcaaaguugacacuguaaaugcacuauc..............................	1	0	c09
.......................................................gcaaaguugacacuguaaaugcacuaucugguacgaca....................	3	0	c09
.......................................................gcaaaguugacacuguaaaugcacuaucugguacgacaU...................	1	1	c09
........................................................caaaguugacacuguaaaugcacuaucugguacgaca....................	1	0	c09
.........................................................aaaguugacacuguaaaugcacuau...............................	1	0	c09
.........................................................aaaguugacacuguaaaugcacuaucu.............................	1	0	c09
.........................................................aaaguugacacuguaaaugcacuaucugguA.........................	1	1	c09
.........................................................aaaguugacacuguaaaugcacuaucugguacgac.....................	1	0	c09
.........................................................aaaguugacacuguaaaugcacuaucugguacgaca....................	9	0	c09
..........................................................aaguugacacuguaaaugc....................................	1	0	c09
..........................................................aaguugacacuguaaaugcacuau...............................	1	0	c09
..........................................................aaguugacacuguaaaugcacuaucuggua.........................	1	0	c09
..........................................................aaguugacacuguaaaugcacuaucugguacgac.....................	2	0	c09
..........................................................aaguugacacuguaaaugcacuaucugguacgaca....................	3	0	c09
............................................................guugacacuguaaaugcacuaucugg...........................	1	0	c09
............................................................guugacacuguaaaugcacuaucuggua.........................	1	0	c09
............................................................guugacacuguaaaugcacuaucugguacgac.....................	2	0	c09
............................................................guugacacuguaaaugcacuaucugguacgaca....................	2	0	c09
............................................................guugacacuguaaaugcacuaucugguacgacaccA.................	1	1	c09
..............................................................ugacacuguaaaugcacuaucugg...........................	2	0	c09
..............................................................ugacacuguaaaugcacuaucugguacgaca....................	2	0	c09
...............................................................gacacuguaaaugcacuauc..............................	1	0	c09
...............................................................gacacuguaaaugcacuaucu.............................	1	0	c09
...............................................................gCcacuguaaaugcacuaucugg...........................	1	1	c09
...............................................................gacacuguaaaugcacuaucugg...........................	2	0	c09
...............................................................gacacuguaaaugcacuaucuggu..........................	1	0	c09
...............................................................gacacuguaaaugcacuaucugguacgaca....................	4	0	c09
................................................................acacuguaaaugcacuaucugguacgac.....................	1	0	c09
................................................................acacuguaaaugcacuaucugguacgacC....................	1	1	c09
................................................................acacuguaaaugcacuaucugguacgaca....................	1	0	c09
.................................................................caGuguaaaugcacuaucugguacga......................	1	1	c09
.................................................................cCcuguaaaugcacuaucugguacgaca....................	10	1	c09
```

Star Mature

```
uccggaguucgugccaggagugcguuugcaaagcacgcuacaguuuugcguuaaggcaaaguugacacuguaaaugcacuaucugguacgacaccccggcgcguguagcgacg
.................................................................cacuguaaaugcacuaucugguacgacU....................	1	1	c09
.................................................................cGcuguaaaugcacuaucugguacgaca....................	2	1	c09
..................................................................aGuguaaaugcacuaucuggua.........................	1	1	c09
..................................................................aGuguaaaugcacuaucugguac........................	1	1	c09
..................................................................acuAuaaaugcacuaucugguacg.......................	1	1	c09
..................................................................aGuguaaaugcacuaucugguacg.......................	2	1	c09
..................................................................aGuguaaaugcacuaucugguacga......................	2	1	c09
..................................................................acuCuaaaugcacuaucugguacgac.....................	1	1	c09
..................................................................aGuguaaaugcacuaucugguacgac.....................	6	1	c09
..................................................................acuguaaaugcacuaucugguacgac.....................	1	0	c09
..................................................................Ccuguaaaugcacuaucugguacgaca....................	3	1	c09
..................................................................aGuguaaaugcacuaucugguacgaca....................	29	1	c09
..................................................................acuUuaaaugcacuaucugguacgaca....................	1	1	c09
..................................................................acuguaaaugcacuaucugguacgaca....................	5	0	c09
..................................................................acuCuaaaugcacuaucugguacgaca....................	4	1	c09
..................................................................acGguaaaugcacuaucugguacgaca....................	3	1	c09
..................................................................Ucuguaaaugcacuaucugguacgaca....................	2	1	c09
...................................................................cGguaaaugcacuaucuggu..........................	1	1	c09
...................................................................cuCuaaaugcacuaucuggu..........................	2	1	c09
...................................................................Guguaaaugcacuaucugguac........................	1	1	c09
...................................................................cuguaaaugcacuaucugguac........................	1	0	c09
...................................................................cGguaaaugcacuaucugguac........................	1	1	c09
...................................................................cGguaaaugcacuaucugguacg.......................	1	1	c09
...................................................................cuUuaaaugcacuaucugguacg.......................	1	1	c09
...................................................................Auguaaaugcacuaucugguacga......................	3	1	c09
...................................................................cAguaaaugcacuaucugguacga......................	1	1	c09
...................................................................Guguaaaugcacuaucugguacga......................	3	1	c09
...................................................................cuguaaaugcacuaucugguacga......................	2	0	c09
...................................................................cGguaaaugcacuaucugguacga......................	1	1	c09
...................................................................Auguaaaugcacuaucugguacgac.....................	1	1	c09
...................................................................cAguaaaugcacuaucugguacgac.....................	1	1	c09
...................................................................cuUuaaaugcacuaucugguacgac.....................	4	1	c09
...................................................................cGguaaaugcacuaucugguacgac.....................	14	1	c09
...................................................................cuCuaaaugcacuaucugguacgac.....................	1	1	c09
...................................................................cuguaaaugcacuaucugguacgac.....................	12	0	c09
...................................................................cCguaaaugcacuaucugguacgac.....................	1	1	c09
...................................................................cuguaaaugcacuaucugguacgCc.....................	1	1	c09
...................................................................Guguaaaugcacuaucugguacgac.....................	10	1	c09
...................................................................Uuguaaaugcacuaucugguacgac.....................	1	1	c09
...................................................................cGguaaaugcacuaucugguacgaca....................	45	1	c09
...................................................................cuguaaaugcacuaucugguacgaca....................	38	0	c09
...................................................................Guguaaaugcacuaucugguacgaca....................	29	1	c09
...................................................................cCguaaaugcacuaucugguacgaca....................	5	1	c09
...................................................................cuAuaaaugcacuaucugguacgaca....................	3	1	c09
...................................................................cuUuaaaugcacuaucugguacgaca....................	10	1	c09
...................................................................cAguaaaugcacuaucugguacgaca....................	5	1	c09
...................................................................Auguaaaugcacuaucugguacgaca....................	8	1	c09
...................................................................cuCuaaaugcacuaucugguacgaca....................	13	1	c09
...................................................................Uuguaaaugcacuaucugguacgaca....................	2	1	c09
...................................................................cuCuaaaugcacuaucugguacgacac...................	1	1	c09
...................................................................cuguaaaugcacuaucugguacgacac...................	1	0	c09
....................................................................uUuaaaugcacuaucugg...........................	2	1	c09
....................................................................uCuaaaugcacuaucuggua.........................	1	1	c09
....................................................................Aguaaaugcacuaucuggua.........................	2	1	c09
....................................................................uUuaaaugcacuaucuggua.........................	1	1	c09
....................................................................Gguaaaugcacuaucuggua.........................	2	1	c09
....................................................................Gguaaaugcacuaucugguac........................	3	1	c09
....................................................................uguaaaugcacuaucugguac........................	1	0	c09
....................................................................uAuaaaugcacuaucugguacg.......................	1	1	c09
....................................................................Cguaaaugcacuaucugguacg.......................	1	1	c09
....................................................................Gguaaaugcacuaucugguacg.......................	3	1	c09
....................................................................uCuaaaugcacuaucugguacg.......................	2	1	c09
....................................................................uUuaaaugcacuaucugguacg.......................	1	1	c09
....................................................................uCuaaaugcacuaucugguacga......................	5	1	c09
....................................................................Gguaaaugcacuaucugguacga......................	7	1	c09
....................................................................Aguaaaugcacuaucugguacga......................	7	1	c09
....................................................................uUuaaaugcacuaucugguacga......................	1	1	c09
....................................................................uguaaaugcacuaucugguacga......................	1	0	c09
....................................................................Gguaaaugcacuaucugguacgac.....................	26	1	c09
....................................................................uUuaaaugcacuaucugguacgac.....................	10	1	c09
```

Star Mature

uccggaguucgugccaggagugcguuugcaaagcacgcuacaguuuugcguuaaggcaaaguugacacuguaaaugcacuaucugguacgacaccccggcgcguguagcgacg			
....uAuaaaugcacuaucugguacgac.....................	2	1	c09
....Aguaaaugcacuaucugguacgac.....................	22	1	c09
....uCuaaaugcacuaucugguacgac.....................	12	1	c09
....Cguaaaugcacuaucugguacgac.....................	2	1	c09
....uguaaaugcacuaucugguacgac.....................	4	0	c09
....uguaaaugAacuaucugguacgac.....................	1	1	c09
....uguUaaugcacuaucugguacgaca....................	1	1	c09
....uUuaaaugcacuaucugguacgaca....................	46	1	c09
....uguaaaugcacuaucugguacgaca....................	8	0	c09
....Cguaaaugcacuaucugguacgaca....................	3	1	c09
....uCuaaaugcacuaucugguacgaca....................	35	1	c09
....uguaaaugcacuaucugguUcgaca....................	1	1	c09
....Aguaaaugcacuaucugguacgaca....................	56	1	c09
....Gguaaaugcacuaucugguacgaca....................	113	1	c09
....uAuaaaugcacuaucugguacgaca....................	1	1	c09
....Aguaaaugcacuaucugguacgacac...................	1	1	c09
....Gguaaaugcacuaucugguacgacac...................	1	1	c09
....uCuaaaugcacuaucugguacgacac...................	1	1	c09
....Cuaaaugcacuaucuggu..........................	4	1	c09
....guaaaugcacuaucuggu..........................	1	0	c09
....Auaaaugcacuaucuggu..........................	1	1	c09
....Cuaaaugcacuaucuggua.........................	15	1	c09
....guaaaugcacuaucuggua.........................	1	0	c09
....guaaaGgcacuaucuggua.........................	1	1	c09
....Uuaaaugcacuaucuggua.........................	3	1	c09
....Auaaaugcacuaucuggua.........................	3	1	c09
....Uuaaaugcacuaucugguac........................	2	1	c09
....guaaaugcacuaucugguac........................	2	0	c09
....Cuaaaugcacuaucugguac........................	4	1	c09
....Auaaaugcacuaucugguac........................	1	1	c09
....guaaaugcacuaucugguacg.......................	1	0	c09
....Auaaaugcacuaucugguacg.......................	3	1	c09
....Cuaaaugcacuaucugguacg.......................	10	1	c09
....Uuaaaugcacuaucugguacg.......................	5	1	c09
....guaaaugcacuaucugguacga......................	2	0	c09
....Cuaaaugcacuaucugguacga......................	37	1	c09
....Auaaaugcacuaucugguacga......................	13	1	c09
....Uuaaaugcacuaucugguacga......................	10	1	c09
....guaaaugAacuaucugguacga......................	1	1	c09
....guaaaugcacuaucAgguacgac.....................	1	1	c09
....Auaaaugcacuaucugguacgac.....................	39	1	c09
....gGaaaugcacuaucugguacgac.....................	2	1	c09
....Uuaaaugcacuaucugguacgac.....................	50	1	c09
....guaaaugcacuaucugguacgac.....................	14	0	c09
....guaGaugcacuaucugguacgac.....................	1	1	c09
....Cuaaaugcacuaucugguacgac.....................	130	1	c09
....guUaaugcacuaucugguacgaca....................	1	1	c09
....Uuaaaugcacuaucugguacgaca....................	154	1	c09
....Auaaaugcacuaucugguacgaca....................	69	1	c09
....gGaaaugcacuaucugguacgaca....................	3	1	c09
....gAaaaugcacuaucugguacgaca....................	1	1	c09
....guaaaugcacuaucugguacgCca....................	1	1	c09
....guaaaugcacuaucugguacgaca....................	27	0	c09
....guaaaGgcacuaucugguacgaca....................	1	1	c09
....Cuaaaugcacuaucugguacgaca....................	369	1	c09
....Uuaaaugcacuaucugguacgacac...................	1	1	c09
....Cuaaaugcacuaucugguacgacac...................	5	1	c09
....guaaaugcacuaucugguacgacac...................	1	0	c09
....uaaaugcacGaucuggua.........................	1	1	c09
....uaaaugcacuaucAggua.........................	5	1	c09
....uaaaugcacuaucuCgua.........................	18	1	c09
....uaaaugcacuaucugAua.........................	8	1	c09
....uaaaugcacuaucugguU.........................	40	1	c09
....uaaUugcacuaucuggua.........................	3	1	c09
....uaaaugcUcuaucuggua.........................	1	1	c09
....uaaaugcacuaucuggua.........................	19000	0	c09
....uaaaugcacuUucuggua.........................	4	1	c09
....uaaaugcacuaucuAgua.........................	4	1	c09
....uaaaugGacuaucuggua.........................	5	1	c09
....uaUaugcacuaucuggua.........................	3	1	c09

```
          Star                                                         Mature
uccggaguucgugccaggagugcguuugcaaagcacgcuacaguuuugcguuaaggcaaaguugacacuguaaaugcacuaucugguacgacaccccggcgcguguagcgacg
......................................................................Gaaaugcacuaucuggua.........................   1569   1   c09
......................................................................uaaaugcacuaGcuggua.........................   3      1   c09
......................................................................Aaaaugcacuaucuggua.........................   157    1   c09
......................................................................uaaaAgcacuaucuggua.........................   21     1   c09
......................................................................uaaaugcacuauUuggua.........................   3      1   c09
......................................................................uaaaugcaUuaucuggua.........................   5      1   c09
......................................................................uaaaugcCcuaucuggua.........................   16     1   c09
......................................................................Caaaugcacuaucuggua.........................   11     1   c09
......................................................................uaaaugcacuaucuggGa.........................   7      1   c09
......................................................................uaaaugcacuauAuggua.........................   6      1   c09
......................................................................uaaaugcaAuaucuggua.........................   15     1   c09
......................................................................uaaaugcacuCucuggua.........................   2      1   c09
......................................................................uaaaugcacuaCcuggua.........................   4      1   c09
......................................................................uaaaugcacuauGuggua.........................   3      1   c09
......................................................................uaaCugcacuaucuggua.........................   9      1   c09
......................................................................uaaaugcacuaucugUua.........................   8      1   c09
......................................................................uaaauUcacuaucuggua.........................   5      1   c09
......................................................................uaaaugcacAaucuggua.........................   3      1   c09
......................................................................uaaauAcacuaucuggua.........................   6      1   c09
......................................................................uaaaugcacuaucugCua.........................   23     1   c09
......................................................................uGaaugcacuaucuggua.........................   2      1   c09
......................................................................uaaaugUacuaucuggua.........................   8      1   c09
......................................................................uaaauCcacuaucuggua.........................   19     1   c09
......................................................................uaaaugAacuaucuggua.........................   29     1   c09
......................................................................uaaaugcacuGucuggua.........................   2      1   c09
......................................................................uaaaugcacuaucuggCa.........................   7      1   c09
......................................................................uaaaugcacuaucuUgua.........................   15     1   c09
......................................................................uaaaugcacuaucCggua.........................   8      1   c09
......................................................................uaaaCgcacuaucuggua.........................   3      1   c09
......................................................................uaaaugcacuaucugguC.........................   81     1   c09
......................................................................uaaaugcaGuaucuggua.........................   2      1   c09
......................................................................uaaGugcacuaucuggua.........................   1      1   c09
......................................................................uaaaugcacuaucGggua.........................   1      1   c09
......................................................................uaaaGgcacuaucuggua.........................   1      1   c09
......................................................................uaaaugcGcuaucuggua.........................   3      1   c09
......................................................................uaaaugcacCaucuggua.........................   3      1   c09
......................................................................uaaaugcacuaucuggAa.........................   22     1   c09
......................................................................uaaaugcacuaAcuggua.........................   10     1   c09
......................................................................uaaaugcacuaucugguG.........................   8      1   c09
......................................................................uaaaugcacuaucuggCac........................   14     1   c09
......................................................................Caaaugcacuaucugguac........................   9      1   c09
......................................................................uaaaugcacuauUugguac........................   3      1   c09
......................................................................uaaaugcacuUucugguac........................   1      1   c09
......................................................................uaaaCgcacuaucugguac........................   4      1   c09
......................................................................uaaaugcacuaucugguaG........................   50     1   c09
......................................................................Gaaaugcacuaucugguac........................   1249   1   c09
......................................................................Aaaaugcacuaucugguac........................   116    1   c09
......................................................................uaaaugcacuaCcugguac........................   4      1   c09
......................................................................uaaaugcacuaucGgguac........................   2      1   c09
......................................................................uaaaugcacuCucugguac........................   3      1   c09
......................................................................uaaaugcacuaucCgguac........................   6      1   c09
......................................................................uaaaugcacAaucugguac........................   1      1   c09
......................................................................uaaaugcaUuaucugguac........................   4      1   c09
......................................................................uaaauUcacuaucugguac........................   7      1   c09
......................................................................uaaaugcacuaucugguac........................   14831  0   c09
......................................................................uaaaugcacuaAcugguac........................   5      1   c09
......................................................................uaaaugcacuaucugguGc........................   4      1   c09
......................................................................uaaaugcacuaucugguaA........................   76     1   c09
......................................................................uaUaugcacuaucugguac........................   1      1   c09
......................................................................uCaaugcacuaucugguac........................   1      1   c09
......................................................................uaaaGgcacuaucugguac........................   2      1   c09
......................................................................uaaaugcGcuaucugguac........................   5      1   c09
......................................................................uaGaugcacuaucugguac........................   1      1   c09
......................................................................uaaaugcacuaucAgguac........................   7      1   c09
......................................................................uaaaugcacuaucugguUc........................   18     1   c09
......................................................................uaaaugcacuaucuggGac........................   9      1   c09
......................................................................uaaauAcacuaucugguac........................   5      1   c09
......................................................................uaaCugcacuaucugguac........................   6      1   c09
......................................................................uaaaugcacuGucugguac........................   2      1   c09
......................................................................uaaaugcCcuaucugguac........................   10     1   c09
```

Star **Mature**

```
uccggaguucgugccaggagugcguuugcaaagcacgcuacaguuuugcguuaaggcaaaguugacacuguaaaugcacuaucugguacgacaccccggcgcguguagcgacg
......................................................................uaaUugcacuaucugguac........................ 1     1 c09
......................................................................uaaaugcacuaucuAguac........................ 3     1 c09
......................................................................uaaaugcacuauGugguac........................ 2     1 c09
......................................................................uaaaugcacuaucugguCc........................ 19    1 c09
......................................................................uaaaugcacuaucugUuac........................ 5     1 c09
......................................................................uaaaugcacuaucugAuac........................ 5     1 c09
......................................................................uaaaugUacuaucugguac........................ 6     1 c09
......................................................................uaaaugAacuaucugguac........................ 19    1 c09
......................................................................uaaaugcacuaucugCuac........................ 21    1 c09
......................................................................uaaaAgcacuaucugguac........................ 2     1 c09
......................................................................uaaaugcacuaucuggAac........................ 5     1 c09
......................................................................uGaaugcacuaucugguac........................ 3     1 c09
......................................................................uaaaugcacuauAugguac........................ 13    1 c09
......................................................................uaaaugcacuaucuCguac........................ 16    1 c09
......................................................................uaaGugcacuaucugguac........................ 2     1 c09
......................................................................uaaaugcUcuaucugguac........................ 4     1 c09
......................................................................uaaaugcacuaucuUguac........................ 5     1 c09
......................................................................uaaauCcacuaucugguac........................ 12    1 c09
......................................................................uaaaugcacuaucugguaU........................ 76    1 c09
......................................................................uaaaugcacCaucugguac........................ 2     1 c09
......................................................................uaaaugcaAuaucugguac........................ 11    1 c09
......................................................................uaaaugcacAaucugguacg....................... 3     1 c09
......................................................................uaaaugcacuauAugguacg....................... 14    1 c09
......................................................................uaaaGgcacuaucugguacg....................... 1     1 c09
......................................................................uaaaugcacuaucugAuacg....................... 3     1 c09
......................................................................uaaauAcacuaucugguacg....................... 3     1 c09
......................................................................uaaUugcacuaucugguacg....................... 6     1 c09
......................................................................uUaaugcacuaucugguacg....................... 2     1 c09
......................................................................uaaaugcacuaucugguacA....................... 48    1 c09
......................................................................uaaaugcacuauGugguacg....................... 7     1 c09
......................................................................uaUaugcacuaucugguacg....................... 5     1 c09
......................................................................uaaaCgcacuaucugguacg....................... 2     1 c09
......................................................................uaaaugcaAuaucugguacg....................... 15    1 c09
......................................................................Gaaaugcacuaucugguacg....................... 2186  1 c09
......................................................................uaaaugcacuUucugguacg....................... 2     1 c09
......................................................................uaaaugcacuaucuCguacg....................... 16    1 c09
......................................................................uaaaugcacuGucugguacg....................... 5     1 c09
......................................................................uaaCugcacuaucugguacg....................... 13    1 c09
......................................................................uaaaugcacuaucAgguacg....................... 11    1 c09
......................................................................uaaaugcacuaucugUuacg....................... 15    1 c09
......................................................................uaaaugUacuaucugguacg....................... 12    1 c09
......................................................................uaaaugcGcuaucugguacg....................... 7     1 c09
......................................................................Caaaugcacuaucugguacg....................... 7     1 c09
......................................................................uaaaugcacuaucuUguacg....................... 10    1 c09
......................................................................uaaaugcacuaucugguacU....................... 195   1 c09
......................................................................uaaauCcacuaucugguacg....................... 33    1 c09
......................................................................uaaaugcaGuaucugguacg....................... 5     1 c09
......................................................................uaaaugcacuaucuAguacg....................... 3     1 c09
......................................................................uaGaugcacuaucugguacg....................... 2     1 c09
......................................................................uaaaugcacuaucGguacg........................ 3     1 c09
......................................................................uaaaugcacuaucugguCcg....................... 9     1 c09
......................................................................uaaaugcacuaucugguacC....................... 227   1 c09
......................................................................uaaaugcacuaucugguaAg....................... 38    1 c09
......................................................................uaaaugcacuaucuggAacg....................... 5     1 c09
......................................................................uaaaugcacuauUugguacg....................... 5     1 c09
......................................................................uaaaugGacuaucugguacg....................... 2     1 c09
......................................................................uaaaugcacuaucugguacg....................... 27095 0 c09
......................................................................uaaauUcacuaucugguacg....................... 12    1 c09
......................................................................uaaaugcacuaCcugguacg....................... 10    1 c09
......................................................................uaaaugcUcuaucugguacg....................... 6     1 c09
......................................................................uaaaugcacCaucugguacg....................... 1     1 c09
......................................................................uGaaugcacuaucugguacg....................... 5     1 c09
......................................................................uaaaugcacuaucugguaGg....................... 17    1 c09
......................................................................uaaaugcacuCucugguacg....................... 1     1 c09
......................................................................uaaaugcCcuaucugguacg....................... 31    1 c09
......................................................................uaaaugcacuaAcugguacg....................... 7     1 c09
......................................................................uaaaugcacuaGcugguacg....................... 3     1 c09
......................................................................uaaaugAacuaucugguacg....................... 52    1 c09
......................................................................uaaaAgcacuaucugguacg....................... 32    1 c09
......................................................................uaaaugcacuaucugCuacg....................... 53    1 c09
```

```
         Star                                                        Mature
uccggaguucgugccaggagugcguuugcaaagcacgcuacaguuuugcguuaaggcaaaguugacacuguaaaugcacuaucugguacgacaccccggcgcguguagcgacg
......................................................................Aaaaugcacuaucugguacg.......................    183    1    c09
......................................................................uaaaugcacuaucuggCacg.......................    11    1    c09
......................................................................uaaaugcacuaucugguaUg.......................    20    1    c09
......................................................................uaaGugcacuaucugguacg.......................    5    1    c09
......................................................................uaaaugcacuaucugguGcg.......................    5    1    c09
......................................................................uaaaugcacGaucugguacg.......................    1    1    c09
......................................................................uaaaugcaUuaucugguacg.......................    12    1    c09
......................................................................uaaaugcacuaucCgguacg.......................    18    1    c09
......................................................................uaaaugcacuaCcugguacga......................    25    1    c09
......................................................................uaaaugcacuaucuggCacga......................    33    1    c09
......................................................................uaCaugcacuaucugguacga......................    1    1    c09
......................................................................uaaaugcacuaucugguacgC......................    145    1    c09
......................................................................uaaaugcacuaucugguacgU......................    107    1    c09
......................................................................uaaaGgcacuaucugguacga......................    4    1    c09
......................................................................uaaaugcUcuaucugguacga......................    16    1    c09
......................................................................uaaauUcacuaucugguacga......................    37    1    c09
......................................................................uaaCugcacuaucugguacga......................    33    1    c09
......................................................................uaaaugcaUuaucugguacga......................    36    1    c09
......................................................................uaaaugcCcuaucugguacga......................    81    1    c09
......................................................................uaaaAgcacuaucugguacga......................    60    1    c09
......................................................................uaaaugcacuaucugguacAa......................    35    1    c09
......................................................................uaaaugcacuaucugguacCa......................    56    1    c09
......................................................................uaUaugcacuaucugguacga......................    7    1    c09
......................................................................uaaaugcacuaucAgguacga......................    28    1    c09
......................................................................uaaaugcacuaucugAuacga......................    14    1    c09
......................................................................uaaaCgcacuaucugguacga......................    10    1    c09
......................................................................uaaauCcacuaucugguacga......................    78    1    c09
......................................................................uaaaugcacuauAugguacga......................    56    1    c09
......................................................................uaaaugcacGaucugguacga......................    3    1    c09
......................................................................uaaaugcaGuaucugguacga......................    8    1    c09
......................................................................uaaaugcacuaAcugguacga......................    30    1    c09
......................................................................uaaaugcacuaucugguUcga......................    7    1    c09
......................................................................uaaUugcacuaucugguacga......................    11    1    c09
......................................................................uaaaugcacAaucugguacga......................    16    1    c09
......................................................................uaGaugcacuaucugguacga......................    6    1    c09
......................................................................uaaGugcacuaucugguacga......................    8    1    c09
......................................................................uaaaugcacuaucugguaGga......................    11    1    c09
......................................................................uaaaugcacuauGugguacga......................    7    1    c09
......................................................................Gaaaugcacuaucugguacga......................    5855    1    c09
......................................................................Caaaugcacuaucugguacga......................    24    1    c09
......................................................................uUaaugcacuaucugguacga......................    8    1    c09
......................................................................uaaaugcacuaucuggGacga......................    4    1    c09
......................................................................uaaaugcacuaucuUguacga......................    24    1    c09
......................................................................uaaaugcacuGucugguacga......................    16    1    c09
......................................................................uaaaugcacuaucugguCcga......................    8    1    c09
......................................................................uaaaugcacuUucugguacga......................    8    1    c09
......................................................................uaaaugAacuaucugguacga......................    93    1    c09
......................................................................uaaaugcacuaucugguaUga......................    56    1    c09
......................................................................uaaaugcacuaucCgguacga......................    28    1    c09
......................................................................uaaaugcacuaucugguacUa......................    70    1    c09
......................................................................uaaaugcacuaucuggAacga......................    29    1    c09
......................................................................uaaaugcacuaucugguGcga......................    14    1    c09
......................................................................uaaaugcacuaucugUuacga......................    40    1    c09
......................................................................uaaaugcacuaucugCuacga......................    134    1    c09
......................................................................uaaaugcacuaGcugguacga......................    11    1    c09
......................................................................uaaaugcacuaucGgguacga......................    9    1    c09
......................................................................uaaaugcacuauUugguacga......................    13    1    c09
......................................................................uaaaugcacuaucugguaAga......................    94    1    c09
......................................................................Aaaaugcacuaucugguacga......................    505    1    c09
......................................................................uaaaugcacuaucugguacgG......................    99    1    c09
......................................................................uaaaugcacuaucuAguacga......................    13    1    c09
......................................................................uCaaugcacuaucugguacga......................    1    1    c09
......................................................................uaaaugGacuaucugguacga......................    6    1    c09
......................................................................uaaaugcaAuaucugguacga......................    39    1    c09
......................................................................uGaaugcacuaucugguacga......................    9    1    c09
......................................................................uaaaugcGcuaucugguacga......................    18    1    c09
......................................................................uaaaugcacCaucugguacga......................    8    1    c09
......................................................................uaaaugcacuaucuCguacga......................    86    1    c09
......................................................................uaaaugUacuaucugguacga......................    26    1    c09
......................................................................uaaauAcacuaucugguacga......................    29    1    c09
```

Star **Mature**

```
uccggaguucgugccaggagugcguuugcaaagcacgcuacaguuuugcguuaaggcaaaguugacacuguaaaugcacuaucugguacgacaccccggcgcguguagcgacg
......................................................................uaaaugcacuaucugguacga......................	72713	0	c09
......................................................................uaaaugcacuCucugguacga......................	4	1	c09
......................................................................uaaaugcacuaucuggCacgac.....................	174	1	c09
......................................................................uaaaugcacuaucuAguacgac.....................	73	1	c09
......................................................................uaaaGgcacuaucugguacgac.....................	29	1	c09
......................................................................uaaaugcacuaucugguacCac.....................	232	1	c09
......................................................................uaaaugcUcuaucugguacgac.....................	62	1	c09
......................................................................uaaaugcacuaucugguUcgac.....................	47	1	c09
......................................................................uaaaugcacuaucugguacgGc.....................	110	1	c09
......................................................................uaaaugcacuaucugguaAgac.....................	406	1	c09
......................................................................Caaaugcacuaucugguacgac.....................	140	1	c09
......................................................................uaaaugcacuaucugguacUac.....................	383	1	c09
......................................................................uaaaugcacuaucugguacgaU.....................	323	1	c09
......................................................................uaaUugcacuaucugguacgac.....................	48	1	c09
......................................................................uaaaugcaAuaucugguacgac.....................	210	1	c09
......................................................................uaaaugcacuaucugguacgUc.....................	84	1	c09
......................................................................uCaaugcacuaucugguacgac.....................	5	1	c09
......................................................................uaaaugcacuaucugAuacgac.....................	46	1	c09
......................................................................uaaaugcacuaucuCguacgac.....................	244	1	c09
......................................................................uaUaugcacuaucugguacgac.....................	43	1	c09
......................................................................uaGaugcacuaucugguacgac.....................	41	1	c09
......................................................................uaaaugcacuaucugguGcgac.....................	83	1	c09
......................................................................uaaaugcacAaucugguacgac.....................	70	1	c09
......................................................................uaaaCgcacuaucugguacgac.....................	52	1	c09
......................................................................uaaCugcacuaucugguacgac.....................	106	1	c09
......................................................................uaaaugcacuaucugCuacgac.....................	563	1	c09
......................................................................uaaauUcacuaucugguacgac.....................	120	1	c09
......................................................................uaaaugGacuaucugguacgac.....................	31	1	c09
......................................................................uaaaugcacGaucugguacgac.....................	9	1	c09
......................................................................uaaaugcacuaucugUuacgac.....................	174	1	c09
......................................................................uaaaugcacuGucugguacgac.....................	58	1	c09
......................................................................uaaaugcacuaucugguCcgac.....................	119	1	c09
......................................................................uaaaugcacuaucuUguacgac.....................	107	1	c09
......................................................................uaaaugcacuauGugguacgac.....................	31	1	c09
......................................................................uaaaugcacuaCcugguacgac.....................	52	1	c09
......................................................................uaaaAgcacuaucugguacgac.....................	235	1	c09
......................................................................Gaaaugcacuaucugguacgac.....................	25659	1	c09
......................................................................uaaaugcGcuaucugguacgac.....................	97	1	c09
......................................................................uaCaugcacuaucugguacgac.....................	9	1	c09
......................................................................uaaaugcacuaGcugguacgac.....................	56	1	c09
......................................................................uaaaugAacuaucugguacgac.....................	413	1	c09
......................................................................uaaaugcacuauUugguacgac.....................	85	1	c09
......................................................................uaaaugcCcuaucugguacgac.....................	278	1	c09
......................................................................uaaaugcacuaucugguacgaA.....................	585	1	c09
......................................................................uaaaugcacuaucugguacAac.....................	96	1	c09
......................................................................uaaaugcacuCucugguacgac.....................	16	1	c09
......................................................................uaaaugcacuauAugguacgac.....................	167	1	c09
......................................................................uaaaugcacuaucGgguacgac.....................	30	1	c09
......................................................................uaaaugcacuaucugguaUgac.....................	277	1	c09
......................................................................uaaaugcacuUucugguacgac.....................	46	1	c09
......................................................................uaaaugcacuaucuggGacgac.....................	29	1	c09
......................................................................uaaaugcacuaucugguacgac.....................	321328	0	c09
......................................................................uUaaugcacuaucugguacgac.....................	22	1	c09
......................................................................uaaaugcacCaucugguacgac.....................	49	1	c09
......................................................................uaaauCcacuaucugguacgac.....................	285	1	c09
......................................................................uaaaugcaUuaucugguacgac.....................	86	1	c09
......................................................................Aaaaugcacuaucugguacgac.....................	2013	1	c09
......................................................................uaaauAcacuaucugguacgac.....................	76	1	c09
......................................................................uaaaugcacuaucugguaGgac.....................	55	1	c09
......................................................................uaaaugcacuaucugguacgaG.....................	364	1	c09
......................................................................uaaaugcaGuaucugguacgac.....................	36	1	c09
......................................................................uaaaugcacuaucuggAacgac.....................	107	1	c09
......................................................................uaaaugcacuaucAgguacgac.....................	127	1	c09
......................................................................uaaaugcacuaucCgguacgac.....................	158	1	c09
......................................................................uaaaugUacuaucugguacgac.....................	114	1	c09
......................................................................uaaGugcacuaucugguacgac.....................	54	1	c09
......................................................................uGaaugcacuaucugguacgac.....................	52	1	c09
......................................................................uaaaugcacuaucugguacgCc.....................	1136	1	c09
......................................................................uaaaugcacuaAcugguacgac.....................	120	1	c09
......................................................................uaaGugcacuaucugguacgaca....................	133	1	c09
```

Star **Mature**

```
uccggaguucgugccaggagugcguuugcaaagcacgcuacaguuuugcguuaaggcaaaguugacacuguaaaugcacuaucugguacgacaccccggcgcguguagcgacg
......................................................................uaaaugcacuaucugguacgaca....................   1025642   0   c09
......................................................................uaaaugcacuaucugCuacgaca....................   1738   1   c09
......................................................................uaUaugcacuaucugguacgaca....................   103   1   c09
......................................................................uaaaugcacuaucugguacUaca....................   1075   1   c09
......................................................................uaaaugcacuaucugguacCaca....................   871   1   c09
......................................................................uaaaugcacuaCcugguacgaca....................   191   1   c09
......................................................................uaaaCgcacuaucugguacgaca....................   209   1   c09
......................................................................uaaaugcacuaucuCguacgaca....................   907   1   c09
......................................................................uaaaugcacCaucugguacgaca....................   141   1   c09
......................................................................uaaaugcacuaucugguacgacC....................   32006   1   c09
......................................................................uaaaugcaUuaucugguacgaca....................   449   1   c09
......................................................................Aaaaugcacuaucugguacgaca....................   6833   1   c09
......................................................................uaaUugcacuaucugguacgaca....................   174   1   c09
......................................................................uaaaugcacuCucugguacgaca....................   68   1   c09
......................................................................uaaaGgcacuaucugguacgaca....................   93   1   c09
......................................................................Caaaugcacuaucugguacgaca....................   468   1   c09
......................................................................uUaaugcacuaucugguacgaca....................   65   1   c09
......................................................................uaaaugcacuaucugAuacgaca....................   179   1   c09
......................................................................uaaaugcacuaucugguGcgaca....................   250   1   c09
......................................................................uaaaugcacuauGugguacgaca....................   119   1   c09
......................................................................uaaaugcacuaucugguacgacG....................   1691   1   c09
......................................................................uCaaugcacuaucugguacgaca....................   22   1   c09
......................................................................uaaaugcacuaucuggAacgaca....................   366   1   c09
......................................................................uaaaugcacuaucGgguacgaca....................   116   1   c09
......................................................................uaaaugcacuGucugguacgaca....................   171   1   c09
......................................................................uaaaugUacuaucugguacgaca....................   328   1   c09
......................................................................uaCaugcacuaucugguacgaca....................   35   1   c09
......................................................................Gaaaugcacuaucugguacgaca....................   82913   1   c09
......................................................................uaaaugcUcuaucugguacgaca....................   215   1   c09
......................................................................uaaauCcacuaucugguacgaca....................   981   1   c09
......................................................................uaaaugcacuaucuUguacgaca....................   303   1   c09
......................................................................uaaaugcacuUucugguacgaca....................   133   1   c09
......................................................................uaaaugcacuauUugguacgaca....................   251   1   c09
......................................................................uaaaugcacuaucugUuacgaca....................   442   1   c09
......................................................................uaaaAgcacuaucugguacgaca....................   827   1   c09
......................................................................uaaaugcacuauAugguacgaca....................   572   1   c09
......................................................................uaaaugcacuaucugguaUgaca....................   761   1   c09
......................................................................uaaaugcacuaucugguacgaAa....................   856   1   c09
......................................................................uaaaugcacuaucuggGacgaca....................   117   1   c09
......................................................................uaaaugcacuaGcugguacgaca....................   144   1   c09
......................................................................uaaaugcacuaucugguacgaGa....................   167   1   c09
......................................................................uaaaugcacuaucugguacgacU....................   16403   1   c09
......................................................................uaaaugcacuaucugguacgCca....................   2234   1   c09
......................................................................uGaaugcacuaucugguacgaca....................   174   1   c09
......................................................................uaaaugcacuaucugguacgaUa....................   287   1   c09
......................................................................uaaaugcacuaucAgguacgaca....................   479   1   c09
......................................................................uaaaugcacuaAcugguacgaca....................   349   1   c09
......................................................................uaaaugcacuaucugguaGgaca....................   111   1   c09
......................................................................uaaaugcacAaucugguacgaca....................   250   1   c09
......................................................................uaaaugcacGaucugguacgaca....................   33   1   c09
......................................................................uaaaugcacuaucugguCcgaca....................   379   1   c09
......................................................................uaaaugcaGuaucugguacgaca....................   164   1   c09
......................................................................uaaaugcacuaucugguaAgaca....................   1295   1   c09
......................................................................uaaaugGacuaucugguacgaca....................   90   1   c09
......................................................................uaaaugcacuaucugguacAaca....................   351   1   c09
......................................................................uaaaugcGcuaucugguacgaca....................   304   1   c09
......................................................................uaaaugcacuaucuggCacgaca....................   650   1   c09
......................................................................uaaaugcacuaucugguacgUca....................   227   1   c09
......................................................................uaaaugcCcuaucugguacgaca....................   906   1   c09
......................................................................uaaaugcacuaucugguUcgaca....................   158   1   c09
......................................................................uaaaugcacuaucuAguacgaca....................   198   1   c09
......................................................................uaaaugcacuaucugguacgGca....................   199   1   c09
......................................................................uaaauAcacuaucugguacgaca....................   259   1   c09
......................................................................uaaaugcacuaucCgguacgaca....................   508   1   c09
......................................................................uaaaugAacuaucugguacgaca....................   1263   1   c09
......................................................................uaGaugcacuaucugguacgaca....................   114   1   c09
......................................................................uaaauUcacuaucugguacgaca....................   443   1   c09
......................................................................uaaaugcaAuaucugguacgaca....................   592   1   c09
......................................................................uaaCugcacuaucugguacgaca....................   359   1   c09
......................................................................uaaaugcacuaucugguacgacaG...................   4153   1   c09
```

```
        Star                                                         Mature
uccggaguucgugccaggagugcguuugcaaagcacgcuacaguuuugcguuaaggcaaaguugacacuguaaaugcacuaucugguacgacaccccggcgcguguagcgacg
......................................................................uaaaCgcacuaucugguacgacac...................    1        1     c09
......................................................................uaaaugcacuaucugguacgGcac...................    4        1     c09
......................................................................uaaaugcacuaucugguacAacac...................    4        1     c09
......................................................................uaaaugcacuaucCgguacgacac...................    6        1     c09
......................................................................uaaaugcacuaucugguacgacCc...................    157      1     c09
......................................................................uaaauAcacuaucugguacgacac...................    2        1     c09
......................................................................uaaaugUacuaucugguacgacac...................    5        1     c09
......................................................................uaaaugcacuaucugguCcgacac...................    3        1     c09
......................................................................Gaaaugcacuaucugguacgacac...................    960      1     c09
......................................................................uaaaugcacuaucuUguacgacac...................    2        1     c09
......................................................................uaaaugcacuaucugguacgaUac...................    4        1     c09
......................................................................Caaaugcacuaucugguacgacac...................    2        1     c09
......................................................................uaaaGgcacuaucugguacgacac...................    2        1     c09
......................................................................uaaaugcacuaucugguacgacac...................    11892    0     c09
......................................................................uaaaugcGcuaucugguacgacac...................    4        1     c09
......................................................................uaaaugcacuauGugguacgacac...................    1        1     c09
......................................................................uaaaugcacuaucugUuacgacac...................    4        1     c09
......................................................................uaaaugcCcuaucugguacgacac...................    7        1     c09
......................................................................uaaaugcacuCucugguacgacac...................    2        1     c09
......................................................................uaaCugcacuaucugguacgacac...................    7        1     c09
......................................................................uaaaAgcacuaucugguacgacac...................    8        1     c09
......................................................................uaaaugcacuaucuggGacgacac...................    1        1     c09
......................................................................uGaaugcacuaucugguacgacac...................    2        1     c09
......................................................................uaaaugcacuaucugguacgacUc...................    171      1     c09
......................................................................uaaaugcacuaucuggAacgacac...................    2        1     c09
......................................................................uaaauCcacuaucugguacgacac...................    8        1     c09
......................................................................uaaaugcacuaucugguacUacac...................    5        1     c09
......................................................................uaaaugcacuaucugguacgacGc...................    51       1     c09
......................................................................uaaaugcaGuaucugguacgacac...................    2        1     c09
......................................................................uaaaugcacuaucugguaGgacac...................    1        1     c09
......................................................................uaaaugcacuaucuggCacgacac...................    5        1     c09
......................................................................uaaaugcacuaucGgguacgacac...................    1        1     c09
......................................................................uaaaugcacuaAcugguacgacac...................    2        1     c09
......................................................................uaaaugcacuauAugguacgacac...................    7        1     c09
......................................................................uaaaugcaUuaucugguacgacac...................    1        1     c09
......................................................................uaaaugcUcuaucugguacgacac...................    3        1     c09
......................................................................Aaaaugcacuaucugguacgacac...................    85       1     c09
......................................................................uaaaugcacuaucugguGcgacac...................    4        1     c09
......................................................................uaaaugcacuaucugguacgaAac...................    19       1     c09
......................................................................uaaaugcacCaucugguacgacac...................    2        1     c09
......................................................................uaaaugcacuaucugguaUgacac...................    9        1     c09
......................................................................uaaaugcacuaucugguacgaGac...................    9        1     c09
......................................................................uaaaugcacuaucugAuacgacac...................    2        1     c09
......................................................................uaaaugcacuaucAgguacgacac...................    5        1     c09
......................................................................uaaaugAacuaucugguacgacac...................    12       1     c09
......................................................................uaaaugcacuUucugguacgacac...................    2        1     c09
......................................................................uaaaugcacuaucugguacgCcac...................    56       1     c09
......................................................................uaaaugcacuaCcugguacgacac...................    3        1     c09
......................................................................uaaaugcacuGucugguacgacac...................    3        1     c09
......................................................................uaaaugcacuaucugguacgacaA...................    13077    1     c09
......................................................................uaaaugcaAuaucugguacgacac...................    5        1     c09
......................................................................uaaaugcacuaucuAguacgacac...................    1        1     c09
......................................................................uaaaugcacuaucugguacCacac...................    13       1     c09
......................................................................uaaaugcacuaucugguacgacaU...................    4788     1     c09
......................................................................uaaaugcacuaGcugguacgacac...................    3        1     c09
......................................................................uUaaugcacuaucugguacgacac...................    1        1     c09
......................................................................uaaaugcacuauUugguacgacac...................    5        1     c09
......................................................................uaaaugcacuaucugCuacgacac...................    13       1     c09
......................................................................uaUaugcacuaucugguacgacac...................    2        1     c09
......................................................................uaaaugcacAaucugguacgacac...................    1        1     c09
......................................................................uaaaugGacuaucugguacgacac...................    1        1     c09
......................................................................uaaaugcacuaucugguaAgacac...................    16       1     c09
......................................................................uaaaugcacGaucugguacgacac...................    1        1     c09
......................................................................uaaaugcacuaucugguUcgacac...................    2        1     c09
......................................................................uaaaugcacuaucuCguacgacac...................    6        1     c09
......................................................................uaaauUcacuaucugguacgacac...................    1        1     c09
......................................................................uaaaugcacuaucugguacgacacA..................    253      1     c09
......................................................................Aaaaugcacuaucugguacgacacc..................    4        1     c09
......................................................................uaaaugcacuaucuCguacgacacc..................    1        1     c09
......................................................................uaaaugcacuaucugguacgacaUc..................    168      1     c09
```

Star **Mature**

```
uccggaguucgugccaggagugcguuugcaaagcacgcuacaguuuugcguuaaggcaaaguugacacuguaaaugcacuaucugguacgacaccccggcgcguguagcgacg
......................................................................uaaaugcacuaucugguacgacacG..................   122   1   c09
......................................................................uaaaugcacuaucugguaAgacacc..................   1   1   c09
......................................................................uaaaugcacuaucugguacgGcacc..................   1   1   c09
......................................................................uaaaugcacuaucugguacgacaGc..................   285   1   c09
......................................................................uaaaugcacuaucugguacgacaAc..................   78   1   c09
......................................................................uaaaugcacuaucugguacgacacc..................   320   0   c09
......................................................................uaaaugcacuaucugguacgacUcc..................   21   1   c09
......................................................................uaaaugcacuaucugguacgacacU..................   441   1   c09
......................................................................uaaaugcacuaucugguacgacGcc..................   14   1   c09
......................................................................uaaaugcacuaucugguacgCcacc..................   1   1   c09
......................................................................uaaaugcacuaucugguacgacCcc..................   9   1   c09
......................................................................Caaaugcacuaucugguacgacacc..................   1   1   c09
......................................................................uaaaugcacuaucugguacgaGacc..................   1   1   c09
......................................................................Gaaaugcacuaucugguacgacacc..................   24   1   c09
......................................................................uaaaugcacuaucugguacgacacAc.................   5   1   c09
......................................................................uaaaugcacuaucugguacgacGccc.................   3   1   c09
......................................................................uaaaugcacuaucugguacgacacGc.................   16   1   c09
......................................................................uaaaugcacuaucugguacgacaccG.................   7   1   c09
......................................................................uaaaugcacuaucugguacgacacUc.................   48   1   c09
......................................................................Gaaaugcacuaucugguacgacaccc.................   4   1   c09
......................................................................uaaaugcacuaucugguacgacaAcc.................   16   1   c09
......................................................................uaaaugcacuaucugguacgacaUcc.................   27   1   c09
......................................................................uaaaugcacuaucugguacgacaccU.................   19   1   c09
......................................................................uaaaugcacuaucugguacgacUccc.................   5   1   c09
......................................................................uaaaugcacuaucugguacgacCccc.................   5   1   c09
......................................................................uaaaugcacuaucugguacgacaGcc.................   43   1   c09
......................................................................uaaaugcacuaucugguacgacaccA.................   28   1   c09
......................................................................uaaaugcacuaucugguacgacaccc.................   56   0   c09
......................................................................uaaaugcacuaucugguacgacacccG................   4   1   c09
......................................................................uaaaugcacuaucugguacgCcaccc................   1   1   c09
......................................................................uaaaugcacuaucugguacgacaAccc................   1   1   c09
......................................................................uaaaugcacuaucugguacgacacccA................   3   1   c09
......................................................................uaaaugcacuaucugguacgacacccc................   8   0   c09
......................................................................uaaaugcacuaucugguacgacacccU................   6   1   c09
......................................................................uaaaugcacuaucugguacgacCcccc................   1   1   c09
......................................................................uaaaugcacuaucugguacgacacUcc................   11   1   c09
......................................................................uaaaugcacuaucugguacgacaccGc................   3   1   c09
......................................................................uaaaugcacuaucugguacgacaGccc................   4   1   c09
......................................................................uaaaugcacuaucugguacgacacAcc................   2   1   c09
......................................................................uaaaugcacuaucugguacgacacGcc................   4   1   c09
......................................................................uaaaugcacuaucugguacgacaccUc................   11   1   c09
......................................................................uaaaugcacuaucugguacgacaccccg...............   2   0   c09
......................................................................uaaaugcacuaucugguacgacacccUg...............   1   1   c09
......................................................................uaaaugcacuaucugguacgacaccccC...............   1   1   c09
```
[truncated: 862,465 more chars]
